# Granule Cell Dispersion in Human Temporal Lobe Epilepsy: Proteomics Investigation of Neurodevelopmental Migratory Pathways

**DOI:** 10.3389/fncel.2020.00053

**Published:** 2020-03-17

**Authors:** Joan Y. W. Liu, Natasha Dzurova, Batoul Al-Kaaby, Kevin Mills, Sanjay M. Sisodiya, Maria Thom

**Affiliations:** ^1^Division of Neuropathology, National Hospital for Neurology and Neurosurgery, London, United Kingdom; ^2^Department of Clinical and Experimental Epilepsy, UCL Queen Square Institute of Neurology, London, United Kingdom; ^3^School of Life Sciences, University of Westminster, London, United Kingdom; ^4^Biological Mass Spectrometry Centre, UCL Great Ormond Street Institute of Child Health, University College London, London, United Kingdom; ^5^Chalfont Centre for Epilepsy, Chalfont St Peter, United Kingdom

**Keywords:** proteome, dentate gyrus, epilepsy, Rho GTPases, migration

## Abstract

Granule cell dispersion (GCD) is a common pathological feature observed in the hippocampus of patients with Mesial Temporal Lobe Epilepsy (MTLE). Pathomechanisms underlying GCD remain to be elucidated, but one hypothesis proposes aberrant reactivation of neurodevelopmental migratory pathways, possibly triggered by febrile seizures. This study aims to compare the proteomes of basal and dispersed granule cells in the hippocampus of eight MTLE patients with GCD to identify proteins that may mediate GCD in MTLE. Quantitative proteomics identified 1,882 proteins, of which 29% were found in basal granule cells only, 17% in dispersed only and 54% in both samples. Bioinformatics analyses revealed upregulated proteins in dispersed samples were involved in developmental cellular migratory processes, including cytoskeletal remodeling, axon guidance and signaling by Ras homologous (Rho) family of GTPases (*P* < 0.01). The expression of two Rho GTPases, RhoA and Rac1, was subsequently explored in immunohistochemical and *in situ* hybridization studies involving eighteen MTLE cases with or without GCD, and three normal post mortem cases. In cases with GCD, most dispersed granule cells in the outer-granular and molecular layers have an elongated soma and bipolar processes, with intense RhoA immunolabeling at opposite poles of the cell soma, while most granule cells in the basal granule cell layer were devoid of RhoA. A higher percentage of cells expressing RhoA was observed in cases with GCD than without GCD (*P* < 0.004). In GCD cases, the percentage of cells expressing RhoA was significantly higher in the inner molecular layer than the granule cell layer (*P* < 0.026), supporting proteomic findings. *In situ* hybridization studies using probes against *RHOA* and *RAC1* mRNAs revealed fine peri- and nuclear puncta in granule cells of all cases. The density of cells expressing *RHOA* mRNAs was significantly higher in the inner molecular layer of cases with GCD than without GCD (*P* = 0.05). In summary, our study has found limited evidence for ongoing adult neurogenesis in the hippocampus of patients with MTLE, but evidence of differential dysmaturation between dispersed and basal granule cells has been demonstrated, and elevated expression of Rho GTPases in dispersed granule cells may contribute to the pathomechanisms underpinning GCD in MTLE.

## Introduction

Temporal lobe epilepsy is the most common form of pharmacoresistant epilepsy in adults (Engel, 1998). Up to 80% of patients with a mesial form of temporal lobe epilepsy (MTLE) have structural abnormalities in the hippocampus (de Tisi et al., 2011; Blumcke et al., 2017). Over 60% of patients with MTLE and hippocampal pathologies remained seizure-free for at least a year after surgical resection of the hippocampus as treatment for their epilepsy (de Tisi et al., 2011; Engel et al., 2012; Blumcke et al., 2017), suggesting that the hippocampus is the primary site for epileptogenesis.

Hippocampal Sclerosis ILAE Type 1 (HS Type 1) is the most common pathology observed in patients with pharmacoresistant MTLE, and it is primarily characterized by segmental loss of pyramidal neurons in cornu ammonis subfields CA1, 3, and 4 (Blumcke et al., 2017). Over half of the MTLE patients with HS Type 1 also have abnormalities affecting dentate granule cells (DGCs) including granule cell dispersion (GCD) and mossy fiber sprouting (Wieser, 2004; Blümcke et al., 2009; Thom et al., 2010; Da Costa Neves et al., 2013). In the normal human hippocampus, DGCs are organized into a compact layer of up to ten cells thick or ≤120 μm (Houser, 1990; Wieser, 2004). In GCD, ectopic DGCs in clusters or rows disperse to the inner and outer molecular layers of the sclerotic hippocampus, widening the granule cell layer to up to 400 μm in thickness (Blümcke et al., 2009). The presence, severity, and extent of GCD along the hippocampal body is highly variable amongst patients with MTLE (Thom et al., 2010; Blümcke et al., 2013) and there is no confirmed grading scheme for GCD in MTLE. The exact cause of GCD in human MTLE is unknown; however, animal models of MTLE with or without hippocampal sclerosis have shown that seizures can displace the migration of newly-generated (Parent et al., 2006; Hester and Danzer, 2013) and mature DGCs to CA4 and molecular layer (Murphy and Danzer, 2011; Koyama et al., 2012; Chai et al., 2014). Reelin is an extracellular matrix glycoprotein secreted by Cajal-Retzius cells during neurodevelopment to regulate the correct layering of migrating cells (Frotscher, 1998). Low levels of reelin transcript and protein have been reported in the hippocampus of patients with MTLE (Haas et al., 2002; Haas and Frotscher, 2010) and animal models of MTLE (Heinrich et al., 2006), possibly as a consequence of elevated methylation at the promotor region of *RELN* (Kobow et al., 2009) or loss of reelin-synthesizing neurons in hippocampus (Haas et al., 2002; Orcinha et al., 2016). The loss of reelin in MTLE is believed to lead to the “over-running” of DGCs into the molecular layer. Past studies have shown that pharmacological inhibition of mammalian target of rapamycin (mTOR) pathway can prevent the development of the mossy fiber sprouting (Buckmaster et al., 2009) and reduce the severity of GCD in animal models of MTLE (Lee et al., 2018), suggesting that the mTOR pathway may have a role in the pathomechanisms of these abnormalities. In patients with MTLE, most astroglial cells strongly expressed markers of mTOR signaling activation such as phospho-S6 ribosomal protein in the sclerotic hippocampus, whereas DGCs showed minimal immunohistochemical evidence of mTOR activation (Sha et al., 2012; Sosunov et al., 2012; Liu et al., 2014). Clinicopathological studies reported that the presence of GCD in patients with MTLE was associated with a history of early onset of epilepsy and febrile seizures (<4 years) and longer duration of epilepsy (Lurton et al., 1998; Blümcke et al., 2009) suggesting that GCD may be a consequence of seizures or brain trauma acquired during the first decade of life where dentate neurogenesis is still active. Although it is unclear whether the presence of GCD is associated with positive surgical outcomes for patients with pharmacoresistant MTLE based on existing literature (Blümcke et al., 2009; Thom et al., 2010; Da Costa Neves et al., 2013), there is supportive evidence from animal studies to show that ectopic DGCs increase hippocampal excitability by having a lower activation threshold, forming excess dendritic axonal connections and receiving more excitatory and fewer inhibitory synaptic inputs than normal cells (Zhan et al., 2010; Murphy and Danzer, 2011; Althaus et al., 2019). In patients with MTLE, GCD is often observed in conjunction with mossy fiber sprouting, where mossy fibers of DGCs form excitatory synaptic contact with apical dendrites and spines of neighboring DGCs in the molecular layer (Sutula et al., 1989; Cavazos et al., 2003), thus potentially creating an internal, pro-epileptogenic circuit.

DGCs are functionally important for cognition and memory since they filter the main inputs into the hippocampus, and propagate signals by innervating pyramidal neurons in CA subfields. Electrophysiological *in vivo* animal studies have demonstrated that DGCs normally have low-excitability, and only a small, spatially-defined population of DGCs would fire to allow the execution of fine and spatially-complex activities such as pattern separation, novelty detection and spatial discrimination (Kahn et al., 2019). Stimulated DGCs release vesicles containing glutamate to activate the population firing of interconnected CA3 pyramidal cells (Miles and Wong, 1983; Scharfman and MacLusky, 2014). Consequently, many stimulated DGCs would enhance hippocampal excitability, thus increasing the chances of seizures (Overstreet-Wadiche et al., 2006; Hester and Danzer, 2013), and reducing the ability to perform fine, spatial discrimination tasks (Kahn et al., 2019). Silencing DGCs using ontogenetic manipulation can reduce seizure frequency and reverse cognitive impairments in animal models of MTLE (Krook-Magnuson et al., 2015).

In view of the important role DGCs play in cognition and promoting hyperexcitability, it is important to understand mechanisms and substrates driving abnormal displacement of DGCs in patients with MTLE. The proteome of the human hippocampus has been studied in normal (Edgar et al., 1999a; Föcking et al., 2012; Koopmans et al., 2018), and diseased post mortem human brains, including in the context of schizophrenia (Edgar et al., 1999b), Alzheimer’s disease (Edgar et al., 1999b; Sultana et al., 2007; Begcevic et al., 2013; Hondius et al., 2016), and non-CNS malignancies (Yang et al., 2004a). In epilepsy, the proteomes of surgically-resected hippocampi from patients with refractory TLE (Czech et al., 2004; Yang et al., 2004b, 2006; Persike et al., 2012, 2018; Mériaux et al., 2014) or temporal cortex (Eun et al., 2004; He et al., 2006; Keren-Aviram et al., 2018) have been studied. Most of these past studies investigated the whole hippocampus rather than specific hippocampal subregions, and information about structural hippocampal pathology was not disclosed in three studies (Czech et al., 2004; Persike et al., 2012, 2018). None of the previous human proteomic studies discussed GCD in their samples. We aimed to investigate the proteomes of DGCs located in the basal and dispersed regions of the granule and molecular layers of patients with HS Type 1 and GCD, and to identify the molecular substrates that mediate GCD. Ectopic DGCs are potential substrates for recurrent excitation, and they may be the key to understanding MTLE with hippocampal sclerosis and GCD, and its comorbidities including cognitive and memory impairments.

## Materials and Methods

### Cases

Patients with refractory MTLE who had undergone surgical resection of the hippocampus as a treatment for their epilepsy between 2005 and 2016 were identified from the records of UCL Epilepsy Society Brain and Tissue Bank (Table 1). All patients provided written informed consent for the use of tissue in research studies in accordance with the Declaration of Helsinki, and the study has obtained ethical approval. Eight cases with age at surgery ranged from 20 to 60 years were submitted to proteomic analysis. All cases had HS Type 1 with marked GCD pathology as confirmed by an experienced neuropathologist.

**Table 1:** Clinical details of cases studied.

Age at surgery	Cases	Relevant clinical history under age of 4 years	Age of onset (years)	Antiepileptic medications	Neuro-psychometric findings	DG pathology laterality (other pathology)	Type of studies proteomic (*n* = 8) RhoA IHC (*n* = 17) RhoA ISH (*n* = 10) Rac1 ISH (*n* = 15)
Between 20–30 years	E1	FS	13	Carb, Lam, Lev, Mid, Val	No remarkable cognitive deficits	HS Type 1. Left. GCD. MFS	All studies
	E20	FS, meningitis	12	Carb	Average intellectual abilities and language functions	HS Type 1. Left. GCD. MFS (TS)	RhoA IHC
	E2	FS	14	Carb, Lev, Ox, Val	Weak verbal and recognition functions	HS Type 1. Right. GCD. MFS (TS)	All studies
	E122	none	14	Carb, Lam, Preg	Weak working and verbal memory	HS Type 1. Right. GCD. MFS.	All except proteomics
	E123	FS	1.5	Carb, Lam, Val	Average intellectual abilities and memory. Weak visuospatial processing abilities	HS Type 3 (OH)	All studies except proteomic
	E16	None	9	Lam, Lev	Average cognition and memory functions	HS Type 3. Right	Rac1-ISH
	E3	Head trauma	11	Carb, Lev, Zon	Weak verbal intelligence and verbal and visual memory	HS Type 1. Left. GCD. MFS	Proteomic, RhoA-IHC
	E12	None	15	Lam, Zon	Weak verbal memory	HS Type 3. Left.	Rac1-ISH
Between 31–40 years	E4	None	10	Carb, Lev	Average cognition and memory functions	HS Type 1. Right. GCD. MFS	Proteomic, Rac1-ISH
	E14	B cell non-Hodgkin’s lymphoma	8	Carb, Lev, Dia	Weak verbal and working memory Depression. Psychosis.	HS Type 1. Left. GCD. MFS	All except proteomics
	E13	FS	1	Val, Clob, Carb	Average cognition and memory functions.	HS Type 1. Right. GCD. MFS	All except proteomics
Between 41–50 years	E5P	None	2	Lev, Carb	Weak visual memory	HS Type 3. Right.	All except proteomic
	E11	None	8	Clonazepam, Ox	Weak cognition and visual memory. Depression, Paranoia	HS Type 1. Right. GCD. MFS	All except proteomics
Between 51–60 years	E5	Head trauma	16	Carb, Lam, Pheny	Weak verbal intelligence and working memory. Anxiety. Depression	HS Type 1. Right. GCD. MFS	Proteomic
	E6	FS	Late 30s	Carb, Ox, Lev, Lam, Pheno	Weak verbal and non-verbal memory. Psychosis	HS Type 1. Left. GCD. MFS.	All except RhoA-ISH
	E7	n/a	11	Val, Preg, Carb	Weak verbal memory and spatial recognition. Paranoia	HS Type 1. Left. GCD. MFS	All studies
	E1P	FS	22	Carb, Clob	Impaired intellectual ability and visual and verbal memory	HS Type 1. Left. GCD. MFS	All except proteomic
	E8	Head trauma	8	Carb, Gaba, Lam, Prim	Poor verbal memory	HS Type 1. Left. GCD. MFS	All studies
29	PM44	COD: sudden death	n/a	None	None	None	RhoA-IHC, qualitative only
35	PM45	COD: cardiac arrest	n/a	None	None	None	RhoA-IHC, qualitative only
57	PM46	COD: no notes available	n/a	None	None	None	RhoA-IHC, qualitative only

*Eight cases with Hippocampal Sclerosis Type I [according to the International League Against Epilepsy (ILAE) classification system] and granule cell dispersion were analyzed using proteomics (E1–8, in gray). In addition to these cases, another ten surgical cases with MTLE and three post mortem normal cases were included in RhoA and Rac1 immunohistochemical and/or *in situ* hybridization studies. *In situ* hybridization studies using probes against *CDC42*, or *RELN* transcripts were performed on four cases (E1P, E122, E1, E3). *Abbreviations*: Carb, carbamazepine; COD, cause of death; Clob, clobazam; Dia, diazepam; Gaba, Gabapentin; GCD, granule cell dispersion; GG, ganglioglioma; HS, hippocampal sclerosis; Lam, Lamotrigine; Lev, Levetiracetam; MCD, malformation of cortical development; MFS, mossy fiber sprouting; OH, oligodendroglial hyperplasia in white matter; Ox, Oxcarbazepine; Pheny, Phenytoin; Pheno, Phenobarbitone; Preg, Pregabalin; Prim, Primidone; TS, temporal sclerosis; Tem, temapazem; Top, topiramate; Val, Sodium valproate; Zon, Zonisamide*.

At the initial neuropathological assessment, two 5 mm-thick blocks from each case were coronally sampled from the middle of the hippocampus (2 cm from anterior tip) to ensure that the dentate gyrus was presented for assessment and subsequent experiments. One block of the sampled hippocampus was snap-frozen in liquid nitrogen, stored in −70 freezers and later retrieved for proteomic studies, while the other block was fixed in 10% neutral-buffered formalin, processed and embedded in paraffin wax for histological staining and immunohistochemistry.

### Laser Capture Microdissection

Fourteen sections of 14 μm thickness were sectioned from each frozen hippocampal sample, and sections were collected onto polyethylene terephthalate metal frame slides for laser capture microdissection (Leica, Milton Keynes, UK). Two additional sections were collected onto a microscopic slide (VWR International, UK) and stained briefly in 0.1% toluidine blue (pH 4.5) solution for 5 s to visualize the granule cell layer. Laser capture microdissection (LCM 700; Leica, Milton Keynes, UK) was then carried out along the entire length of the GCL of multiple sections per case to capture basal and dispersed DGCs (Figure 2A). Basal samples included DGCs in the granule cell layer closest to CA4, while the dispersed samples included ectopic DGCs in the outer-granular layer and inner and outer molecular layers. A total tissue area of 9 ± 1 mm^2^ was dissected for each case, and submitted for proteomic analysis.

**Figure 1:**
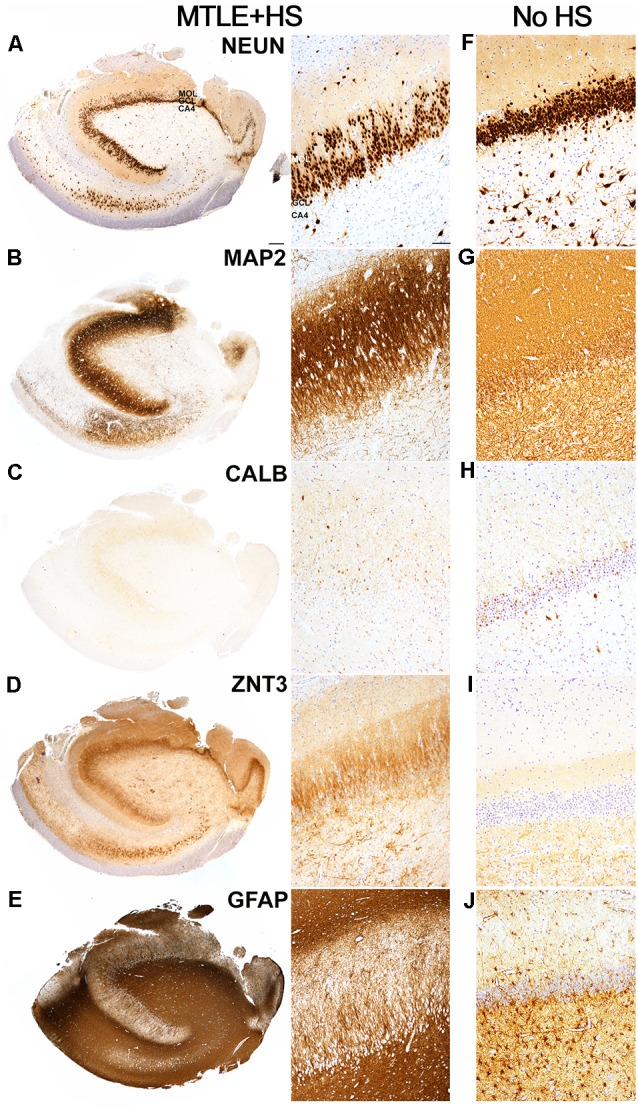
Hippocampal pathology observed in an Mesial Temporal Lobe Epilepsy (MTLE) patient with Hippocampal Sclerosis and Granule Cell Dispersion. **(A)** NeuN-positive granule cells were observed in the granule cell layer of the hippocampal dentate gyrus. The number of dentate granule cells (DGCs) remained abundant despite marked loss of pyramidal neurons in the cornu ammonis (CA) 1, 3 and 4 subfields, which are typical features of Hippocampal Sclerosis Type 1 based on classification scheme established by the task force of International League against Epilepsy (ILAE; Blümcke et al., 2013). However, the majority of the NeuN-positive DGCs appeared to scatter in rows or clusters to the outer-granular layer and molecular layer (MOL), which is consistent with granule cell dispersion pathology. NeuN-positive pyramidal neurons were still observed in the CA2 region. **(B)** I MAP2-positive DGCs were observed in the granule cell layer. MAP2-positive DGCs had immunopositive fibers projected towards the molecular layer, and MAP2-positive labeling appeared to be stronger in the molecular layer than in the basal granule cell layer. MAP2-positive fibers of various lengths and a small number of MAP2-positive cells were observed in the CA4 region. **(C)** Calbindin immunoreactivity was observed in most, but not all, dispersed and elongated DGCs in the granule and molecular layer. In contrast, small, round basal DGCs were immunonegative for calbindin. An intense blush of granular calbindin-positive labeling was also observed in the molecular layer. **(D)** Intense ZnT3-positive labeling was observed in the molecular layer, particularly in the inner molecular layer. This is consistent with mossy fiber sprouting pathology in MTLE. Clusters of ZnT3-positive fibers were observed predominantly in the CA4 region. **(E)** The dense matrix of GFAP-positive fibers was observed in all CA regions. GFAP-positive radially-directed fibers extended between DGCs in the granule cell layer, and GFAP-positive cells were visible in the molecular layer. **(F–J)** Images showing the immunoreactivities of NeuN, MAP2, calbindin, ZnT3, and GFAP in the hippocampus of an MTLE patient with no remarkable hippocampal pathologies. Scale bars in **(A)** left 500 μm, and **(A)** right 50 μm.

**Figure 2:**
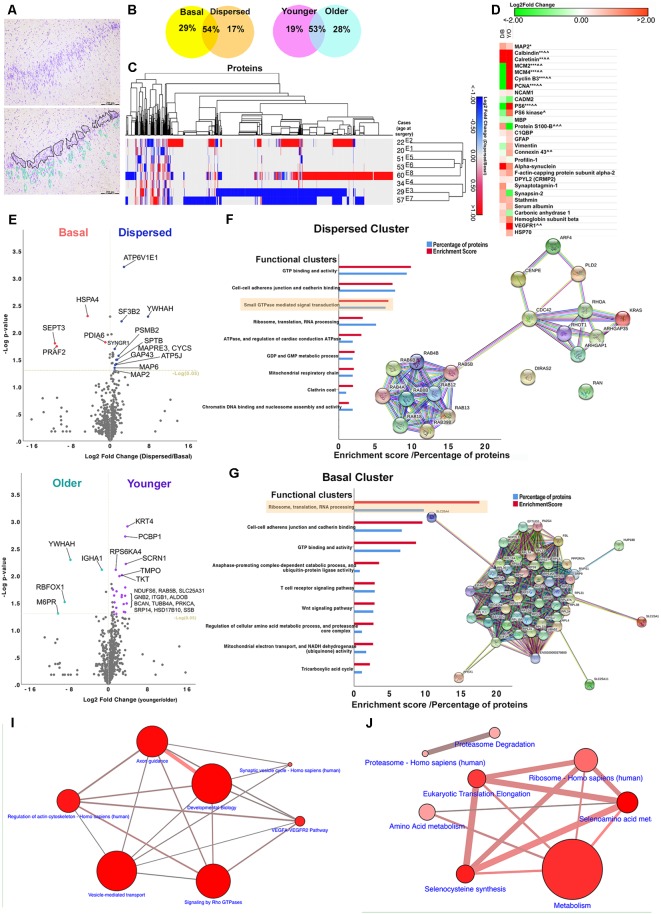
Bioinformatics analyses. **(A)** An image of a toluidine-stained hippocampal tissue section showing marked granule cell dispersion pathology (top). DGCs were extracted from frozen hippocampal tissue sections during laser microdissection. Extracted DGCs (including nuclei and ~10 μm-perinuclear rim as cytoplasm) were divided into either basal or dispersed samples per case based on their location within the dentate gyrus. Basal samples contained DGCs located in the basal layer of the granule cell layer (black outline). These round DGCs were closely situated to neighboring DGCs, and they were aligned along the border of CA4 and granule cell layer. Dispersed samples contained a mixture of round and elongated DGCs located ectopically, in rows or clusters in the outer-granular, inner and outer molecular layers (green outline). Individual, small, round nuclei scattered in the dentate gyrus, sometimes in close proximity to DGCs, were likely to glial cells, and therefore these cells were avoided where possible. **(B)** Finding from proteomic studies show that more than 50% of the identified proteins were shared between basal and dispersed samples and between younger (<35 years) and older cohorts (<50 years). Less than one-third of the identified proteins were uniquely detected in basal, dispersed, younger and older samples. **(C)** A dendrogram showing hierarchical clustering of all differentially expressed proteins in eight MTLE cases with GCD. The pattern of protein changes between dispersed and basal samples was more similar between cases E1 and E2 (younger cohort) and E5, E6, and E8 (older cohort). **(D)** Heat map showing logarithmic-2 fold change between dispersed and basal samples (D/B), or younger and older cohorts (Y/O) of selected proteins expressed in neuronal (MAP2, calbindin, calretinin), glial (GFAP, S100-B, MBP, connexion 43), vascular (VEGFR), immature cell (vimentin) and proliferative populations (MCM2, MCM4, PCNA, cyclin B3), or are proteins associated with cytoskeletal (profiling-1, alpha-synuclein, f-actin capping protein, DPYL2, stathmin) and synapse remodeling (synaptotagmin-1, synapsin-2), mTOR pathway (PS6, PS6 kinase), neurogenic niches (NCAM1, CADM2), vascular changes (carbonic anhydrase 1, hemoglobin) and inflammation (C1QBP, HSP70). Keys: * or ^∧^ significantly overexpressed proteins (*P* < 0.05), **proteins found in dispersed samples of GCD cases only, ***proteins found in basal samples of GCD cases only, ^∧∧^proteins only found in younger cohort only, ^∧∧∧^proteins found in older cohort only. Abbreviations: C1QBP, complement component 1Q subcomponent-binding protein; DPYL2, dihydropyrimidinase-related protein 2; GFAP, glial fibrillary acidic protein; HSP70, heat shock proteins; MAP2, microtubule-associated protein 2; MBP myelin binding protein; MCM2, minichromosomal maintenance 2; NCAM1, neural cell adhesion molecule 1; PCNA, proliferating cell nuclear antigen; PS6 ribosomal protein S6; VEGFR vascular endothelial growth factor receptors. **(E,H)** Volcano plots showing logarithmic-2 fold change against –log *P*-value for proteins that were expressed in both dispersed and basal samples **(E)** or in both younger and older cohorts **(H)**. Significantly differentially expressed proteins that showed over the 1.5-fold change were highlighted in the plots (*P* < 0.05). **(F,G)** The abundance of each protein was compared between basal and dispersed samples, and between younger and older cohorts. Proteins that were uniquely expressed in one group only, and proteins that were significantly expressed by 1.5-fold than comparative group (fold change >1.5, *P* < 0.05) generated the Dispersed, Basal, Younger and Older clusters. Each cluster was submitted to bioinformatics platforms for Gene Ontology and Pathways analyses. Top functional annotation clusters based on Gene Ontology, Uniprot keywords, and sequences and INTERPRO terms for proteins in the Dispersed **(F)** and Basal clusters **(G)** were listed (*P* < 0.01; also refer to Supplementary Material S3). The enrichment scores generated by bioinformatics platforms and the percentage of proteins submitted that were associated with each functional annotation cluster are illustrated. Network maps show the connections between proteins in small GTPase mediated signal transduction **(F)** and ribosomes annotation clusters **(G)**. **(I,J)** Proteins in the Dispersed and Basal clusters were significantly involved in a number of pathways (*P* < 0.01; also refer to Supplementary Material S4). The size of the circle represents the number of proteins submitted that were involved in the pathways. The thickness of connections refers to the number of proteins that belongs to both connecting pathways.

### Proteomic and Bioinformatics Analyses

Samples were denatured using in-solution trypsin digestion and prepared for MSe label-free quantitative proteomics as described previously (Heywood et al., 2013; Manwaring et al., 2013; Liu et al., 2016). Data were processed using ProteinLynx GlobalServer version 2.5. Protein identifications were obtained by searching the UniProt reference human proteomes with the sequence of porcine trypsin (P00761) added. Protein identification from the low/high collision spectra for each sample was processed using a hierarchical approach where more than three fragment ions per peptide, seven fragment ions per protein, and more than two peptides per protein had to be matched. Peptide identification was accepted if they could be established at 95% or greater probability. Statistical analyses of group means were performed using *t*-test in SPSS (v25; IBM, USA) to identify significantly differentially expressed proteins between dispersed and basal samples, or younger and older cohorts (*P* < 0.05). The younger cohort consisted of four cases with an age at surgery ranging from 20 to 34 years, and the older cohort included four cases with an age at surgery ranging from 51 to 60 years. For volcano plots, fold change between groups was transformed using base 2 logarithmic transformations and plotted against the negative logarithmic transformation of *P*-value. List of differentially expressed proteins in Basal, Dispersed, Younger and Older clusters included proteins found only in the respective samples, and proteins that were significantly different between comparison group (fold change >1.5, *P* < 0.05). Enriched lists were analyzed using bioinformatics resources, Database for Annotation, Visualization and Integrated Discovery (DAVID; version 6.8; Huang da et al., 2009a,b) and Enrichr (Chen et al., 2013; Kuleshov et al., 2016), to obtain information regarding biological, cellular and molecular functions of proteins. Interactions between proteins were investigated using STRING analysis^1^, and proteins in functional pathways were explored in established pathway databases, Kyoto Encyclopaedia of Genes and Genomes (KEGG), Reactome, and Wikipathways. *P*-values and adjusted *P*-values (corrected according to Bonferroni’s method and false discovery rate) were calculated by bioinformatics resources. For gene ontology annotation and enriched pathway analyses, the stringency of the criteria was set to high, and *P*-value cut-off was set at 0.01 and only pathways with four proteins of interests or more were presented. The dendrogram was constructed using Morpheus (Broad Institute^2^) and included hierarchical clustering based on Pearson correlation coefficient, and average linkage clustering (Meunier et al., 2007).

Other clinical details, including patients’ epilepsy history and psychometry, were also reviewed and analyzed with proteomic data. Pre-operative MRI sequences, PET, EEG, video-telemetry, and psychometry had been carried out according to the epilepsy surgical protocols at the National Hospital of Neurology and Neurosurgery, and clinical findings were reviewed for each case.

### Immunohistochemistry

Eighteen cases were included in RhoA, Rac1, and Cdc42 immunohistochemistry and/or *in situ* hybridization studies. Cases included eight MTLE cases submitted for proteomics, an additional six surgical MTLE with hippocampal sclerosis and GCD, four surgical MTLE with hippocampal sclerosis but no GCD, and three postmortem healthy controls (Table 1).

Five micrometer-thick formalin-fixed, paraffin-embedded sections from selected cases were examined histologically with Haematoxylin and Eosin, and Luxol Fast Blue stains. Routine automated and manual immunochemistry was performed using antibodies against neuronal nuclei (NeuN), glial fibrillary acidic protein (GFAP), non/phosphorylated neurofilament (SMI32, SMI31), myelin basic protein (SMI94), microtubule associated protein 2 (MAP2), zinc transporter 3 (ZnT3) calbindin, nestin (Table 2). Additional immunohistochemistry using anti-RhoA was performed manually using pretreatment and incubation procedures described in Table 2, and breast carcinoma tissue was used as a positive control (Ma et al., 2010). Negative controls where primary antibodies were omitted showed no specific labeling. A number of commercially-available antibodies against RhoA, RhoB and Cdc42 (Santa Cruz Biotechnology, Germany), RhoC and pan-Rac1 (Cell Signalling Biotechnology, UK) were also tested using formalin-fixed, paraffin-embedded human brain tissue, but no specific immunolabeling was obtained after applying a number of different antigen retrieval methods. These antibodies were excluded from further immunohistochemical studies.

**Table 2:** Antibodies and protocols for RNA and immunohistochemical studies.

Antibodies/Probes Clone, Code	Immunogen or target epitope	Labeled cell or protein type	Pre-treatment (mins)	Antibody supplier, dilution, incubation time (mins) and temperature
Anti-NeuN A60, MAB377	Purified cell nuclei from mouse brain	Nuclei of most neurons, some proximal dendrites	ER1 (20)	Millipore; 1:2K, 15, RT
SMI31 801601	Phosphorylated epitope in neurofilament H and M	Neurons	H-3301 (12)	Sternberger; 1:500, ov, 4°C
SMI32 801707	Non-phosphorylated epitope in neurofilament H	Neurons	H-3301 (12)	Sternberger; 1:500, ov, 4°C
Anti-MAP2 HM-2, M4403	Rat microtubule-associated proteins	Neurons	H-3301 (12)	Sigma; 1:1,500, ov, 4°C
Anti-Calbindin D-28K, 300	Calbindin D-28k	Calbindin-expressing interneurons	H-3301 (12)	Swant; 1:10K, ov, 4°C
Anti-ZnT3 197–003	Recombinant protein of mouse ZnT3 (aa. 2–75)	Synaptic vesicle located zinc-transporter	H-3301 (12)	Synaptic Systems; 1:10K, ov, 4°C
SMI 94 SMI-94R	70–89 aa of human myelin basic protein	Myelinated fiber in white matter	ENZ1 (10)	DAKO; 1:2,000, 20, RT
Anti-Nestin 10C2, AB22035	150 aa recombinant fragment from human nestin conjugated to GST	Immature progenitors, glia, endothelial cells	H-3301 (12)	Abcam; 1:1K, ov, 4°C
Anti-GFAP Z0334	GFAP isolated from cow spinal cord	Astrocytes, some ependymal cells	ENZ1 (10)	DAKO; 1:2.5K, 20, RT
Anti-RhoA OSR00266W	A synthetic peptide from human Transforming protein RhoA conjugated to an immunogenic carrier protein	Small GTPase protein that regulates actin cytoskeleton in the formation of stress fibers, and cell division. Expressed in the cytoplasm of cells undergoing structural changes in preparation for migration	Protease (20, 37°C)	Thermo; 1:500, ov, 4°C
*RHOA* mRNA 416291	NM_001664.2. Probe region from 135 to 1796	Pretreatment 2 and 3*	BioTechne; 120, 40°C
*RAC1* mRNA 419851	NM_006908.4 Probe region from 1194 to 2256		Pretreatment 2 and 3*	BioTechne; 120, 40°C
*CDC42* mRNA 502651	NM_044472.2 Probe region from 2 to 1091	Pretreatment 2 and 3*	BioTechne; 120, 40°C
*RELN* mRNA 413051	NM_173054.2 Probe region from 4401 to 5340	Glycoprotein expressed by Cajal-Retzius cells	Pretreatment 2 and 3*	BioTechne; 120, 40°C

Automated immunohistochemistry was performed using the Bond Max automated immunostainer and reagents (Leica, Milton Keynes, UK). *RNA detection was performed using the RNAscope patent assays system following the supplier’s instruction. Abbreviations: aa, amino acids; Cdc42, Cell division control protein 42 homolog; GFAP, glial fibrillary acidic protein; K, thousands; MAP, microtubule-associated protein; NeuN, neuronal nuclei; ov, overnight; Rac1, Ras-Related C3 Botulinum Toxin Substrate 1; RhoA, Transforming protein RhoA or Ras homolog family member A; RT, room temperature. Antigen retrieval buffers: ENZ1, Leica Bond enzyme concentrate, and diluent; ER1, Leica Bond citrate-based buffer; ER2, Leica Bond EDTA-based buffer; H-3301, Vector’s Tris-based buffer pH 9.0; H-3300, Vector’s citrate-based buffer pH 6.0; SC, Sodium citrate buffer, pH 6.0. Suppliers: Abcam plc., Cambridge, UK; BD Transduction Lab., Oxford, UK; Bio-Techne, Abingdon, UK; DAKO, Cambridgeshire, UK; Millipore, Watford, UK; Sternberger, Maryland, US; Swant, Marly, Switzerland; Thermo Fisher Scientific, Hemel Hempstead, UK; Synaptic Systems, Goettingen, Germany.

### *In situ* Hybridization

Five micrometer-thick formalin-fixed, paraffin-embedded sections from selected surgical cases were processed for *in situ* hybridization following supplier’s instructions (Wang et al., 2019). Probes against human *RHOA, RAC1, CDC42*, and *RELN* mRNAs were used in conjunction with ready-to-use reagents from RNAscope^®^ 2.5 HD Reagent Brown and Duplex kits (Table 2; Bio-Techne, Abingdon, UK). Positive probe control, *UBC*, and negative probe control, *dapB* transcripts, were performed on Hela cell tissue (positive tissue control) and formalin-fixed, paraffin-embedded surgical human brain tissue to ensure specificity of labeling prior to final studies. Labeled sections were counterstained with Gills I hematoxylin solution (VWR, UK) before coverslipped.

### Image Analysis

Labeled sections were assessed qualitatively using a brightfield microscope (Nikon Eclipse 80i) and subsequently scanned at 40x magnification using the whole slide scanner, AxioScan.Z1 (Zeiss, Germany) to obtain high-quality digital images for quantitative analysis and data interpretation. All images were viewed and analyzed using the image analysis software, QuPath (Bankhead et al., 2017). Criteria for GCD: at present, there are no strict criteria to evaluate GCD, or to identify the boundaries amongst the granule cell layer and inner and outer molecular layers. In this study, the extent of GCD in each case was assessed by measuring the thickness of the granule cell layer along with the external and internal limbs on hematoxylin and eosin-stained, whole-slide images at 2.8x magnification. The curved region joining the internal and external limbs of GCL was not included. An average of 19 ± 2 measurements (mean ± SEM) with a mean interval of 400 ± 3 μm were made along the entire length of GCL in each case. Each line measurement was taken from the basal granule cell layer closest to CA4 to the furthest dispersed DGCs in the outer molecular layer. The length of each line was then divided into three groups: ≤120 μm (no GCD, DGCs within granule cell layer as previously reported in Blümcke et al., 2009), between 121 and 215 μm (moderate GCD, scattered DGCs in the inner molecular layer) and ≥216 μm (severe GCD, scattered, clusters or rows of DGCs in the outer molecular layer). The threshold of 215 μm was taken from the longest line measurement to the most dispersed cell minus 120 μm (granule cell layer) to get the thickness of the molecular layer and then divided by two to derive a mid-point to divide the inner and outer molecular layer (Supplementary Material S1). The frequency of lines in each measurement group was used to categorize the extent of GCD in each case.

Automated quantification of RhoA immunopositive labeling, and RhoA and Rac1-positive puncta from *in situ* hybridization studies were performed using QuPath. First, granule cell layer, inner and outer molecular layers spanning 120, 215 and 310 μm from the basal granule cell layer were annotated. The software was then trained to recognize hematoxylin-stained nuclei, positive labeled cells and puncta using positive cell and subcellular detection modules (Bankhead et al., 2017). The number of cells with RhoA-positive labeling and RhoA/Rac1-positive puncta per μm^2^, and the percentage of cells detected with RhoA immunolabeling and RhoA/Rac1-positive puncta were recorded and compared between MTLE cases with or without GCD, three dentate regions and younger or older cohorts using non-parametric Mann–Whiney or Kruskal–Wallis tests in SPSS (IBM, USA; *P* < 0.05). Spearman correlations were performed to examine the relationship between quantitative measures and thickness of granule cell layer, age of onset or age at surgery (*P* < 0.01).

## Results

### Granule Cell Pathology Observed in Cases Submitted for Proteomics

In MTLE cases with GCD, a thick band of NeuN-positive cells was observed in the dentate granule cell layer (Figure 1A). Two morphologies of NeuN-positive DGCs were noted: the basal population of DGCs located closer to CA4 was round and tightly packed with neighboring DGCs (basal DGCs), and a dispersed population of NeuN-positive DGCs with round or elongated soma with uni- or bipolar processes located in outer-granular and molecular layers of the hippocampus (dispersed DGCs). In the case of E6, the bilamination of the granule cell layer was observed, and a gap of approximately 130 μm measured from the bottom of the basal layer to the top of the dispersed layer was noted. No marked difference in NeuN-positive immunolabeling was noted between younger and older cohorts. In contrast, the granule cell layer in the hippocampus of four MTLE cases with no GCD (Figure 1F) and healthy post mortem controls contained round, tightly packed NeuN-positive cells, and no immunopositive cells were observed in the molecular layer. Quantitative measures of granule cell layer revealed a significantly thicker granule cell layer in MTLE cases with GCD than cases without GCD or healthy controls (*P* = 0.004; mean ± SEM, range; *cases with GCD* 133 ± 11 μm, 71–213 μm; *cases without GCD* 64 ± 10 μm, 34–82 μm;* healthy controls*; 70 ± 4 μm, 63–76 μm).

MAP2-positive cells were observed in the granule cell layer of all MTLE cases (Figures 1B,G). Numerous MAP2-positive processes were observed in the granule and molecular layers. Random short MAP2-positive processes were observed in the CA4 around a few large densely-labeled MAP2-positive cells. Calbindin-positive cells expression was observed in DGCs scattered throughout the outer-granule cell layer and molecular layer of MTLE cases with GCD (Figure 1C). In cases with GCD, calbindin-positive dispersed DGCs had round cell bodies, and occasionally, calbindin-positive cells with long processes extending into the molecular layer were noted. Not all DGCs in the outer-granular layer were calbindin-positive, and the majority of basal DGCs in the sclerotic hippocampus were devoid of calbindin immunolabeling. In contrast, calbindin-positive DGCs were often observed in the granule cell layer of cases without GCD (Figure 1H). ZnT3-positive processes were observed in between DGCs in the granule cell layer and in the molecular layer and CA4 subfield of cases with GCD (Figure 1D). In cases without GCD, ZnT3 immunolabeling was primarily observed in CA4 (Figure 1I). GFAP immunolabeling was observed throughout the hippocampus of all cases, with more intense labeling detected in MTLE cases with GCD (Figure 1E) compared to cases without hippocampal sclerosis or GCD (Figure 1J), and healthy controls. DGCs in the granule cell layer were not immunopositive for GFAP, but GFAP-positive processes extended between DGCs in the granule cell layer, and individual GFAP-positive cells were observed in the molecular layer of cases with GCD. In cases with GCD, a very dense matrix of GFAP processes was observed in the CA4. In contrast, distinctive GFAP-positive cells were seen in the CA4 of cases without GCD (Figure 1J) and in healthy postmortem controls.

### Proteomic Analysis

One-thousand eight-hundred and eighty-two proteins were identified in the proteomic analysis of eight patients with HS Type 1 and GCD (Table 1, E1–8). 29% of the extracted proteins were observed in the basal samples only, 17% in the dispersed samples only, and 54% of the proteins were observed in both samples (Figure 2B). 19% of identifiable proteins were observed in the younger cohort only, 28% in older samples only and the remaining proteins were found in both younger and older samples. Some similar changes in protein expression were observed between two cases in the younger cohort (E1 and E2), and amongst three older cases, E5, E6, E8 based on hierarchical clustering (Figure 2C).

Common neuronal markers such as MAP2, calbindin, and calretinin were detected in MTLE samples (Figure 2D). The protein abundance of MAP2, calretinin, and calbindin was significantly higher in dispersed and younger samples than basal and older samples. The quantity of MAP2 detected was higher than calbindin and calretinin in all samples as expected based on earlier immunohistochemical studies (Figures 1B,C). Astroglial (GFAP, vimentin, S100-B) and oligodendroglial markers (Myelin Basic Protein, MBP) were also detected in all samples, consistent with immunohistochemical findings (Figure 1E). The expression of GFAP was comparable between basal and dispersed samples and was slightly higher in younger than older samples; of interest, the expression of vimentin, expressed in immature astrocytes, was predominantly observed in basal and younger samples (Figure 2D) in keeping with our previous studies (Liu et al., 2018). The expression of connexion 43, a gap junction protein expressed in astrocytes, was similar to the expression of GFAP. Common microglial-specific proteins including TREM2, LST1, HLA-DRA, SP11, MMP9 were not found in our samples, but complement component 1q subcomponent binding protein (C1QBP), a protein found to be highly expressed in microglia around site of lesion in a recent animal study (Barna et al., 2019), was found to be expressed in both dispersed and basal samples. We did not detect common markers of neural progenitors or neuroblasts including SOX2, PAX6, TBR1, TBR2, and DCX; however, a higher abundance of cell adhesion molecule 2 (CADM2), a synapse-associated protein found in subventricular neurogenic niche (Lee et al., 2012; Frese et al., 2017), as well as a number of cell cycle markers (MCM2, PCNA, Cyclin B3) were found in higher amount in the basal DGCs than dispersed DGCs in cases with GCD (Figure 2D). Neural cell adhesion molecule 1 (NCAM) was also detected in dispersed samples of cases with GCD. Together these findings suggest that apart from DGCs and astroglial cell types, minimal level of microglia and neural progenitor cells were included in our capture. A number of proteins involved in actin and cytoskeleton remodeling including profilin-1 and 2, alpha-synuclein, f-actin capping proteins, dihydropyrimidinase-related protein 2 (also known as CRMP2), and synapse proteins such as synaptotagmin-1 and synapsin-2, were identified in dispersed and younger samples at a higher level than in basal and older samples. The detection of serum albumin, carbonic anhydrase 1 and hemoglobin subunits was noted in all samples, possibly due to increased permeability of blood brain barrier in the hippocampus of patients with MTLE.

Volcano plots in Figures 2E,H highlighted the most significantly differentially-expressed proteins between dispersed and basal samples, and between younger and older cohorts respectively. Proteins uniquely expressed in dispersed samples, and proteins significantly displaying over 1.5-fold change in dispersed compared to basal samples (Figure 2E; Supplementary Material S2) were first submitted to gene ontology and pathway bioinformatics analyses (Dispersed cluster, 330 proteins). The top functional annotation clustering based on Gene Ontology, Uniprot keywords, and sequences, and INTERPRO terms were GTP-binding and activity (enrichment score, 10; *P* = 3.67 × 10^−8^), cell to cell adhesion (enrichment score, 7; *P* = 2.61 × 10^−6^), and small GTPase-mediated signal transduction (enrichment score, 7; *P* = 5.60 × 10^−4^); Figure 2F and Supplementary Material S3). Under the functional clustering of small GTPase-mediated signal transduction, a number of proteins in the Ras homolog (Rho) GTPase families such as RhoA, Rac1, Cdc42 and ARGAP1, and ARGAP35 were identified (Table 3 and Supplementary Material S5). Key pathways associated with differential proteins in the Dispersed cluster were related to cellular migration and actin cytoskeletal remodeling, including signaling by Rho GTPases pathway (R-HSA194315; *P* = 1.02 × 10^−6^), axon guidance (R-HSA422475; *P* = 1.25 × 10^−6^), regulation of actin cytoskeleton (K-HSA04810; *P* = 1.01 × 10^−4^), and vesicle-mediated transport (R-HSA5653656, *P* = 9.29 × 10^−7^; Figure 2I and Supplementary Material S4). Rho GTPases are small GTPases that regulate cytoskeletal dynamics and cell migration, which maybe of relevance to abnormal migration of DGCs in GCD. The signaling by Rho GTPases pathway shared common proteins with other pathways related to cytoskeletal dynamics as illustrated in Figure 2I. Proteins detected in basal samples only, and proteins that were significantly overexpressed in basal samples by at least 1.5-fold compared to dispersed samples (Figure 2E and Supplementary Material S2) were subsequently submitted to gene ontology and pathway enrichment analyses (Basal cluster; 555 proteins). The top functional annotation clustering included proteins involved in ribosomes, translation and RNA processing (enrichment score, 18; *P* = 1.79 × 10^−14^), cell-cell adherens (enrichment score, 10; *P* = 1.93 × 10^−8^), GTP binding (enrichment score, 9; *P* = 4.31 × 10^−7^), ubiquitin-protein ligase activity (enrichment score, 4; *P* = 4.926 × 10^−2^), and regulation of amino acid metabolic processes and proteasome activity (enrichment score, 3; *P* = 2.07 × 10^−2^; Figure 2G and Supplementary Material S3). The interactions between proteins clustered under ribosomes, translation and RNA processing are shown as a network map in Figure 2G. Pathways associated with Basal cluster were predominantly related to metabolism (R-HSA1430728, *P* = 2.53 × 10^−26^), including amino acid (WP3925, *P* = 234 × 10^−5^) and selenocysteine metabolisms (H-HSA2408557, *P* = 1.71 × 10^−24^), and electron transport chain (WP111, *P* = 4.73 × 10^−3^), and ribosomes (K-HSA03010, *P* = 5.00 × 10^−13^), translational mechanisms (R-HSA156842, *P* = 2.90 × 10^−24^), and proteasomal degradation (WP111, *P* = 4.73 × 10^−3^; Figure 2J and Supplementary Material S4).

**Table 3:** Proteins in the Dispersed cluster that were involved in signaling by the Rho GTPases pathway (Reactome, R-HSA194315; *P* = 4.68 × 10^−9^).

Entry ID	Gene	Protein
P60953	CDC42	Cell division control protein 42 homolog
E7EVJ5	CYFIP2	Cytoplasmic FMR1-interacting protein 2
Q7L576	CYFIP1	Cytoplasmic FMR1-interacting protein 1
F8W7L3	A2M	Alpha-2-macroglobulin
Q12802	AKAP13	A-kinase anchor protein 13
H0YE29	ARHGAP1	Rho GTPase-activating protein 1
Q9NRY4	ARHGAP35	Rho GTPase-activating protein 35
I3L4C2	BAIAP2	Brain-specific angiogenesis inhibitor associated protein 2
P11274	BCR	Breakpoint cluster region protein
A0A087X0P0	CENPE	Kinesin-like protein Dispersed
G3XAM7	CTNNA1	Catenin-alpha-1
P78352	DLG4	Disks large homolog 4
Q99880	HIST1H2BL	Histone H2B type 1-L
Q15691	MAPRE1	Microtubule-associated protein RP/EB family member 1
Q9Y2A7	NCKAP1	Nck-associated protein 1
Q9NZQ3	NCKIPSD	NCK-interacting protein with SH3 domain
P35080	PFN2	Profilin-2
P62140	PPP1CB	Serine/threonine-protein phosphatase PP1-beta catalytic
C9J9C1	PPP2R1A	Serine/threonine-protein phosphatase 2A 65 kDa regulatory subunit A
P63000	RAC1	Ras-related C3 botulinum toxin substrate 1
C9JNR4	RHOA	Transforming protein RhoA/Ras homologous family member A
P62745	RHOB	Rho-related GTP-binding protein RhoB
Q8IXI2	RHOT1	Mitochondrial Rho GTPase 1
P31947	SFN	14–3–3 protein sigma
O43295	SRGAP3	SLIT-ROBO Rho GTPase-activating protein 3
A2IDB2	YWHAH	14–3–3 protein eta

### Immunohistochemical and *in situ* Hybridization Using Markers Against Rho GTPases

Bioinformatics analyses revealed a number of proteins in the signaling by Rho GTPases pathway either uniquely expressed or upregulated in dispersed samples of MTLE cases with GCD. To further investigate the expression of Rho GTPases, immunohistochemistry and *in situ* hybridization using antibodies and probes against RhoA, Rac1 and Cdc42 protein and mRNAs respectively were performed on surgical, formalin-fixed, paraffin-embedded, hippocampal tissue from cases submitted to proteomics, and additional surgical MTLE cases with or without GCD. Post mortem hippocampal tissue from three healthy donors was also included for qualitative assessment.

**Figure 3:**
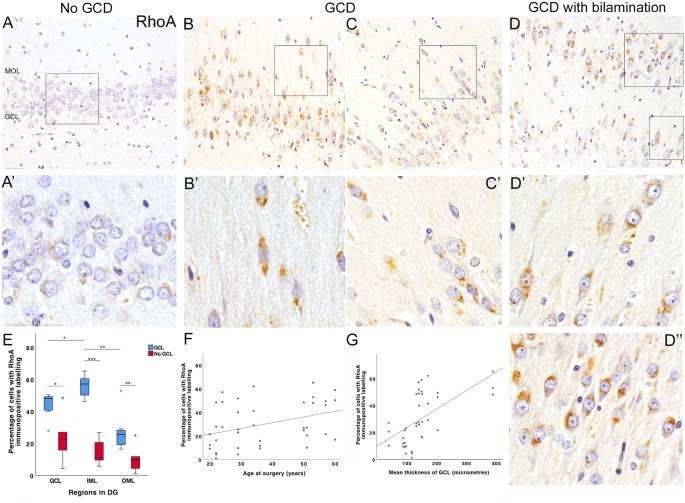
RhoA immunohistochemical studies. **(A)** Most DGCs were immunonegative for RhoA in the granule cell layer of an MTLE case without GCD (E5P). Occasionally, weak perinuclear RhoA-positive labeling was observed in a few DGCs (**A’**, *higher magnification of area outlined in* **A**). No DGCs were observed in the molecular layer of cases without GCD. **(B,C)** Numerous DGCs with intense RhoA immunolabeling were observed in the granule and molecular cell layer of two MTLE cases with GCD (E14, **B**; E3, **C)**. In some DGCs, RhoA immunolabeling was detected in one polar end of the DGC soma directed towards the CA4 or molecular layer. A number of RhoA-positive cells in the outer-granular and molecule layer had elongated soma with uni- or bipolar processes **(B’,C’)**. In bipolar DGCs, RhoA immunolabeling was observed at one or both apices of cell soma. Occasionally, RhoA-positive DGCs were found to align single line formation near blood vessels **(C’)**. In contrast, most basal DGCs closest to the CA4 border had no or minimal RhoA immunolabeling. **(D)** Bilamination of the granule cell layer was observed in a case with MTLE and GCD E6. In this case, most DGCs in the basal granule cell layer appeared to be devoid of RhoA immunolabeling. In contrast, nearly all DGCs in the outer-granule layer **(D’)** and second granule cell layer expressed RhoA **(D”)** either in one or both ends of the cell. The majority of RhoA-positive cells in the second granule cell layer had stronger and distinct RhoA immunoreactivities at the end directed towards the molecular layer. **(E)** A boxplot showing that the percentage of cells with RhoA-positive labeling in the dentate gyrus of MTLE cases with GCD (blue) or without GCD (red). A significantly higher percentage of cells expressing RhoA was observed in granule and molecular layers of cases with GCD than cases without GCD (GCL, *P* = 0.035; IML, *P* = 0.001; OML, *P* = 0.008; see Supplementary Material S6). In cases with GCD, the percentage of cells with RhoA immunolabeling was significantly higher in the inner molecular layer than the granule cell (*P* = 0.026) and outer molecular layer (*P* = 0.003). **(F,G)** The percentage of cells expressing RhoA in the dentate gyrus weakly correlated with the age at surgery of MTLE patients (*r*_s_ = 0.390, *P* = 0.014; **F**) and the mean thickness of granule cell layer (*r*_s_ = 0.675, *P* < 0.001; **G**). Abbreviations: DG, dentate gyrus; GCL, granule cell layer; IML, inner molecular layer; OML, outer molecular layer. Scale bars, 50 μm. *P ≤ 0.05, **P ≤ 0.01, ***P ≤ 0.001.

In cases without GCD, the majority of DGCs in the granule cell layer were immunonegative for RhoA (Figures 3A,A′). In contrast, cases with GCD had numerous RhoA-immunopositive DGCs in the outer-granule and molecular layers. RhoA-immunopositive DGCs in the granule cell layer generally had a large, round nucleus surrounded by a thin, perinuclear “ring” of immunopositive labeling, or a “cone” of localized RhoA immunolabeling at one end of the cell soma, usually facing either towards the molecular layer or CA4 region (Figures 3B,3B′,3C,3C′). Occasionally, RhoA-positive cells in single-chain formation near vascular structures were observed (Figures 3C,C′). Most RhoA-positive cells in the molecular layer had an elongated cell soma with protruding uni- or bipolar processes. In a case with a bilaminar granule cell layer (E6), most DGCs in the basal granule cell layer were devoid of RhoA immunolabeling (Figure 3D), while most DGCs in the second granule cell layer had distinct RhoA immunoreactivities in one or bipolar ends of the cell soma (Figures 3D′,D″). Quantitative studies revealed a significantly higher density of RhoA-positive cells and a higher percentage of cells expressing RhoA in the granule and molecular layer of MTLE cases with GCD than without GCD (mean ± s.e.m;* GCL*, GCD 44 ± 3%, no GCD 23 ± 6%, *P* = 0.035; *IML*, GCD 56 ± 3%, no GCD 14 ± 3%, *P* = 0.001; *OML* GCD 27 ± 5%, no GCD 10 ± 3%, *P* = 0.008; Figure 3E and data in Supplementary Material S6). In cases with GCD, a higher percentage of cells expressing RhoA were detected in the inner molecular cell layer than the granule cell layer and the outer molecular layer (IML-GCL, *P* = 0.026; IML-OML, *P* = 0.003; Figure 3E). In contrast, the percentage of RhoA-positive cells detected were not significantly different between regions in cases without GCD. No significant difference in density or percentage of cells expressing RhoA was noted between younger and older cohorts. A probable relationship was observed between the percentage of cells expressing RhoA and age of surgery (*r*_s_ = 0.390, *P* = 0.014; Figure 3F), and the mean thickness of granule cell layer (*r*_s_ = 0.675, *P* < 0.001; Figure 3G).

**Figure 4:**
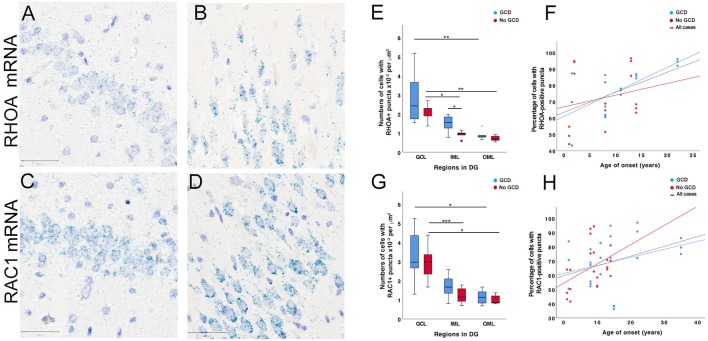
*RHOA* and *RAC1 in situ* hybridization studies. **(A–D)** *RHOA* and *RAC1*-positive puncta (cyan) were observed in the dentate gyrus of MTLE cases without GCD **(A,C)** and cases with GCD **(B,D)**. The majority of positive puncta were found in or around hematoxylin-stained nuclei (purple) in the granule and molecular cell layers. In MTLE cases with GCD, most *RHOA* and *RAC1-*positive puncta were localized peri-nuclearly and at opposite ends of each nucleus **(B,D)**. Many *RAC1-*positive puncta were also observed in the proximal portion of uni- or bipolar processes **(D)**. A number of *RHOA* and *RAC1-*positive puncta were also observed in the neuropil. **(E,G)** In cases without GCD, the density of cells with *RHOA and RAC1-*positive puncta was higher in the granule cell layer than inner (*RHOA* *P* = 0.023; *RAC1* *P* = 0.001) and outer molecular layer (*RHOA* *P* = 0.004; *RAC*1 P = 0.018). In cases with GCD, significantly higher densities of cells with *RHOA* and *RAC1-*positive puncta were observed in the granule than outer molecular layers (*RHOA* *P* = 0.004; *RAC1* *P* = 0.017), but not in the inner molecular layer (*P* > 0.05). The density of cells expressing *RHOA* mRNAs in the inner molecular layer was significantly higher in cases with GCD than cases without GCD (*P* = 0.05), but this difference was not observed in the granule cell layer or outer molecular layer (*P* > 0.05). **(F,H)** The percentage of cells with *RHOA* or *RAC1-*positive puncta weakly correlated with age of onset in patients with GCD (*RHOA*, *r*_s_ = 0.439, *P* = 0.011; *RAC1*, *r*_s_ = 0.295, *P* = 0.049), but not in patients without GCD. Abbreviations: DG, dentate gyrus; GCL, granule cell layer; IML, inner molecular layer; OML, outer molecular layer. Scale bars, 50 μm. *P ≤ 0.05, **P ≤ 0.01, ***P ≤ 0.001.

*In situ* hybridization studies using probes against *RHOA* and* RAC1* mRNA sequences revealed fine nuclear and perinuclear puncta in DGCs in the granule and molecular layers of all cases (Figures 4A–D). *RHOA*-positive and *RAC1*-positive puncta were also observed in the neuropil, likely representing processes of DGC which were not visible in the sections. In cases with GCD, a high number of *RHOA*-positive and *RAC1*-positive puncta clustered at the polar ends of the DGC soma, particularly in DGCs situated in the molecular layer (Figures 4B,D). Some smaller hematoxylin-stained glial cells were associated with none or only a few numbers of *RHOA*-positive or *RAC1*-positive puncta. Further immunohistochemical and *in situ* hybridization double-label studies showed that glutamine synthetase-labeled astrocytes did not have *RHOA*-positive or *RAC1*-positive puncta (Supplementary Material S7).

Quantitative analyses showed that a higher density of cells with *RAC1*-positive puncta than *RHOA* was observed in MTLE with GCD (mean ± SEM, *RAC1* 2.05 × 10^−3^ ± 2.15 × 10^−4^ per μm^2^; *RHOA* 1.74 × 10^−3^ ± 2.71 × 10^−4^; *P* = 0.017), which was not observed in cases without GCD (*RAC1* 1.72 × 10^−3^ ± 2.27 × 10^−4^ per μm^2^; *RHOA* 1.25 × 10^−3^ ± 1.81 × 10^−4^; *P* > 0.05). In all cases, a significantly higher density of cells with *RHOA*-positive puncta was observed in the granule cell layer than outer molecular layer (GCD, GCL 2.83 × 10^−3^ ± 5.61 × 10^−4^ per μm^2^, OML 8.97 × 10^−4^ ± 1.01 × 10^−4^, *P* = 0.004 (blue bars); no GCD, GCL 2.11 × 10^−3^ ± 2.23 × 10^−4^ per μm^2^, OML 7.24 × 10^−4^ ± 6.94 × 10^−5^, *P* = 0.004 (red bars); Figure 4E and Supplementary Material S6). A significantly higher density of *RHOA*-positive puncta was observed in the granule cell layer than inner molecular layer in cases without GCD (GCL 2.11 × 10^−3^ ± 2.23 × 10^−4^ per μm^2^, IML 9.25 × 10^−4^ ± 9.15 × 10^−5^, *P* = 0.023), but not in cases with GCD (GCL 2.83 × 10^−3^ ± 5.61 × 10^−4^ per μm^2^, IML 1.49 × 10^−3^ ± 1.84 × 10^−4^, *P* > 0.05), indicating that the level of *RHOA* gene expression was more similar between granule and inner molecular layers in cases with GCD than in cases without GCD. A significantly higher density of *RHOA*-positive puncta was observed in the inner molecular layer of cases with GCD than without GCD (GCL 1.49 × 10^−3^ ± 1.84 × 10^−4^ per μm^2^ IML; no GCD IML 9.25 × 10^−4^ ± 9.15 × 10^−5^, *P* = 0.05). In all cases, the densities of cells with *RAC1*-positive puncta were higher in granule cell layer than the outer molecular layer (GCD, 3.31 × 10^−3^ ± 4.47 × 10^−4^ per μm^2^ GCL, 1.14 × 10^3^ ± 1.24 × 10^−4^ OML, *P* = 0.017; no GCD, 2.92 × 10^−3^ ± 3.47 × 10^−4^ per μm^2^ GCL, 1.01 × 10^−4^ ± 8.99 × 10^−5^ OML, *P* = 0.018, Figure 4G). A significantly higher density of *RAC1*-positive puncta was noted in the granule cell later compared to inner molecular layer in cases without GCD (GCL 2.92 × 10^−3^ ± 3.47 × 10^−4^ per μm^2^, IML 1.23 × 10^−3^ ± 1.62 × 10^−4^, *P* = 0.001), but not in cases with GCD (GCL 3.31 × 10^−3^ ± 4.47 × 10^−4^ per μm^2^, IML 1.70 × 10^−3^ ± 2.06 × 10^−4^, *P* > 0.05). The percentage of cells with *RHOA*-positive and *RAC1*-positive puncta weakly correlate with the age of onset (*RHOA*, *r*_s_ = 0.439, *P* = 0.011, Figure 4F; *RAC1*, *r*_s_ = 0.294, *p* = 0.049, Figure 4H).

*In situ* hybridization using probes against *CDC42* and *RELN* mRNAs were also performed on surgical cases, and labeling was qualitatively assessed as only four cases were investigated. The regional distribution and density of cells with *CDC4*2-positive puncta were similar to *RHOA*-positive and *RAC1*-positive positive labeling. *RELN*-positive puncta were not observed in any of these cases.

## Discussion

We investigated the proteome of basal and dispersed DGCs in the dentate gyrus of pharmacoresistant MTLE patients with hippocampal sclerosis to explore the neurodevelopmental pathomechanisms of GCD. We have identified differences in the proteomes between basal and dispersed populations of DGCs in the hippocampus of patients with MTLE and GCD. Specifically, dispersed DGCs in cases with GCD highly expressed mature neuronal markers, MAP2, calbindin, and calretinin as well as a number of Rho GTPases and proteins associated with cell migration, cytoskeleton, and synapse remodeling. These results were further supported by findings from immunohistochemical and *in situ* hybridization studies, where a significantly higher density and percentage of cells expressing RhoA mRNA and protein was observed in the dentate gyrus of cases with GCD than cases without GCD. The expression of RhoA protein was localized to the opposite ends of uni- or bipolar of DGCs in the molecular layer of cases with GCD. Consistently, the mRNAs of *RHOA, RAC1*, and *CDC42* were also found in the same intracellular region of DGCs, while *RAC1* mRNA was also detected in the proximal portion of processes. These findings provide evidence supportive of DGCs undergoing neurodevelopment processes relating to cellular migration, which may contribute to the abnormal dispersion of DGCs in patients with MTLE.

### Comparison With Previous Human Proteomics Studies

The current study reported on proteins identified from the dentate gyrus of eight patients with refractory MTLE and HS Type I and GCD (or Type II granule cell pathology according to Blümcke et al., 2009) using protocols established in our previous proteomic study (Liu et al., 2016). There is no specific marker for DGCs in the human brain (unlike Prox1 or NeuroD in rat brains), but other neuronal markers such as MAP2 and calbindin, which are expressed by granule cells, were detected at high levels in our immunohistochemical and proteomic studies. Our samples did not capture microglial cell contaminants, but astroglial markers (GFAP, S100-B) and myelin basic protein were detected, and this is consistent with the intimate intermingling of radial glial and myelinated axons in the dentate gyrus in MTLE with hippocampal sclerosis.

Our proteomic studies did not include surgical or post mortem healthy controls as seen in most previous human proteomic studies of MTLE. This is because (i) surgically-resected “normal” hippocampal tissue from healthy individuals is not available for research; (ii) the process of protein extraction for proteomic analyses is different for surgical and post mortem brain tissue so one standardized protocol cannot be applied to both type of tissue; and (iii) autopsy tissue is likely to be affected by post mortem processes such as rapid protein degradation (especially for tubulins, intermediate filaments, high motility group box protein-1, proapolioprotein, hemoglobin and mutant derivative (He et al., 2006) which may influence our findings. In view of these arguments, we compared the protein profile of basal and dispersed DGCs as well as between younger (<35 years) and older groups (>50 years) to avoid the detection of changes associated with tissue type.

The differential expression of certain proteins in our study is generally consistent with findings from previous human MTLE studies. There is good agreement amongst human TLE proteomics studies that most proteins related to metabolism are significantly upregulated in epilepsy compared to archival normal brain hippocampal samples (Eun et al., 2004; Yang et al., 2006; Persike et al., 2012, 2018). This may be because the generation of epileptiform activity, or response to such activity, consumes a large amount of energy, and rapid glycolysis and oxidative phosphorylation are necessary to fulfill energy demands in the epileptic human brain (Kovac et al., 2017). Consistently, we found 15% of proteins extracted from our epilepsy samples were involved in metabolic processes, and up to 8% of proteins in the Basal cluster were involved in the tricyclic acid cycle and mitochondrial electron chain (Figure 2G and Supplementary Material S4).

A key finding in this study is the identification of proteins in dispersed DGCs of cases with GCD specifically involved in axonal guidance (including beta-spectrin, growth-associated protein 43, postsynaptic density protein 95, profilins), regulation of actin cytoskeleton (cytoplasmic FMR1-interacting proteins, WAVE complex proteins), cytoskeletal and synaptic remodeling (DPYL2, alpha-synuclein, synaptotagmin 1, synapsin II, and stathmin1), as well as Rho GTPases, signaling (RhoA, Cdc42, Rac1, brain-specific angiogenesis inhibitor, Rho GTPases activating proteins; Table 3). These processes are active during neurodevelopment and in neurogenic niches in the adult mammalian brain (Lee et al., 2012; Frese et al., 2017). Our findings are consistent with previous human proteomic studies which have reported increased expression of DPYL2 (also known as collapsing response mediator protein 2, CRMP2), a cytoplasmic phosphoprotein that binds microtubules and promotes neurite outgrowth during neurogenesis and neuronal migration (Inagaki et al., 2001; Fukata et al., 2002), in the hippocampus of MTLE patients compared to controls (Persike et al., 2012, 2018; Keren-Aviram et al., 2018). The CRMP2 antagonist, lacosamide, is an antiepileptic drug often used in patients with drug-resistant epilepsy (Kelemen and Peter, 2010).

### GCD and Neurogenesis: Evidence?

During neurodevelopment, new DGCs migrate from the dentate ventricular zone to the granule cell layer to form an “outside-in” dentate gyrus, where the outer-granular cells are the oldest (and showing earliest NeuN immunoreactivity), and DGCs located in the inner layer (basal granule cells) are the youngest (Altman and Bayer, 1990; Seress et al., 2001). A secondary dentate matrix is formed at gestation week (GW) 10–11 which will later become the subgranular zone that supports ongoing neurogenesis that continues to adulthood so there is also an outside-in gradient for neurogenesis (Cipriani et al., 2017). Previous studies have reported that the dentate neurogenesis is still active postnatally at 1 year of age, but proliferative and neurogenic activities decline sharply between 7–13 years and argued to be minimally detected in adulthood (Sorrells et al., 2018) even in the brains of adult patients with MTLE (Blümcke et al., 2001; Cipriani et al., 2017). The level of hippocampal neurogenesis in the normal and diseased adult human brain is still controversial because the detection of neural progenitor cells and new neurons in adult human brains depends on tissue quality and the immunohistochemical protocols employed in studies (Sorrells et al., 2018; Moreno-Jiménez et al., 2019). Considering that the level of neurogenesis is lower in adults than children, it is more plausible that new DGCs born in early childhood contributes to GCD. Although our current findings did not capture immature neural progenitor cell proteins (SOX1, SOX2, TBR1, TBR2, PAX6), or neuroblast proteins (DCX), even in the youngest MTLE patients with age at surgery of 20 and 22 years, we did detect a number of proliferative cell cycle proteins, minichromosome maintenance protein 2 (MCM2), proliferative cell nuclear antigen (PCNA) and cyclin B3, and immature glial progenitor protein, vimentin, in the basal DGCs of younger MTLE cases and not in the dispersed DGCs, thus providing some supporting evidence of neuroplasticity. Our previous immunohistochemical study found a number of proliferative cells expressing MCM2 in the granule cell layer of patients with MTLE (Thom et al., 2005). In addition, we detected a high abundance of neural cell adhesion molecule 1 in dispersed DGCs of younger MTLE cases. In adult animal neurogenesis studies, calretinin is transiently expressed in newborn DGCs before calbindin (Brandt et al., 2003). Calretinin was detected in the dispersed samples of this study, however as we know marked re-organization of calretinin neurons and networks occurs in the hippocampus of patients with MTLE and hippocampal sclerosis (Thom et al., 2012), calretinin may not be a reliable marker of adult neurogenesis in this context. Calbindin D28K was only detected in dispersed DGCs in our study, which is consistent with previous studies that reported reduced expression of calbindin in basal granule cell layer of patients with MTLE (Maglóczky et al., 1997; Arellano et al., 2004; Abrahám et al., 2009, 2011; Martinian et al., 2012).

We did not detect reelin, a glycoprotein secreted by Cajal-Retzius cells to regulate the migration of DGCs during neurodevelopment, in our samples. This is confirmed in subsequent *in situ* hybridization studies using a probe against *RELN* mRNA, where no positive labeling was observed in the hippocampus of MTLE cases with GCD. This finding is consistent with previous studies that have reported low levels of *RELN* mRNA in MTLE patients with hippocampal sclerosis and GCD (Haas et al., 2002; Frotscher et al., 2003; Kobow et al., 2009). In those studies, reelin was detected in the CA1, 3 and 4, and we did not sample those areas.

### Rho GTPases and Mechanisms for Migration or GCD

Due to the observation of Rho GTPases in the dispersed DGCs, these proteins have become the focus of further investigation as to their role in pathomechanisms underlying GCD.

The main finding in this study is the upregulation of proteins involved in the Rho GTPase signaling pathway in dispersed DGCs of patients with MTLE and GCD. Rho GTPases belong to the Rho family of GTPases, which is a subgroup of the Ras family of small GTP binding protein (Heasman and Ridley, 2008). Ras homologous member A (RhoA), Ras-related C3 (Rac1) and cell division cycle 42 (Cdc42) are the three most-studied Rho GTPases, and the activated form of these Rho GTPases bind with a number of effector molecules (Dia, ROCK, myosin light chain, phosphatase and kinase, WAVE, Arp2/3, PAK, LIMK, Cofilin-P, Mec-3, WASP; see Supplementary Material S5) to regulate actin polymerization, microtubule stabilization, and actomyosin contractility during neurodevelopment when cells undergo active morphogenesis and participate in migratory, proliferative, and survival activities (Heasman and Ridley, 2008; Zarco et al., 2019). Data from the Human Brain Transcriptome project has reported continuous gene expression of *RHOA, RAC1* and *CDC42* in the human hippocampus from embryonic and early fetal periods to adulthood (4 post-conceptional weeks to 60 years of age; Supplementary Material S8; Kang et al., 2011). Similarly, we observed a high number of DGCs expressing *RHOA, RAC1* and *CDC42* mRNAs in the brains of adult MTLE patients with and without GCD, with age at surgery spanning 20–60 years. Whether the protein expression of these Rho GTPases is continuously expressed in DGCs from development to adulthood in the normal and MTLE human brain remain to be investigated as there is currently limited information. In this study, we found significantly higher protein expression of RhoA in uni- or bipolar ends of DGCs in cases with GCD than without GCD, particularly in displaced DGCs in the outer-granule and molecular layers. RhoA was absent in most DGCs located in the basal granule cell layer of MTLE cases. Considering the established role of RhoA in polarized cell migration in neurogenic niches, it is plausible that RhoA has a role in the mismigration of DGCs in cases with DGCs. The distinct localization of RhoA to the apices of dispersed DGC soma may be suggestive of proximal cytoplasmic bulging, a characteristic feature of cells undergoing saltatory migratory during neurodevelopment and in neurogenic zones of adult mammalian brains (Schaar and McConnell, 2005; Wang et al., 2019). During saltatory migration, the centrosome of the migrating cell moves forward to form a transient swelling in the proximal leading process and then the cell body and nucleus move towards the centrosome then pause and this process is repeated again. Previous *in vitro* morphodynamic studies have found spatiotemporal changes to the expression of RhoA in migrating cells as they undergo saltatory migration (Fritz et al., 2013; Martin et al., 2016; Kaneko et al., 2017). These studies detected RhoA initially at the transient swelling of the leading proximal process where RhoA interacted with effector proteins to regulate f-actin and myosin II in the actomyosin contraction to allow cell soma to translocate forward (Solecki et al., 2009; Ota et al., 2014). As the cell moved forward then stalled, RhoA expression was reduced in the front and gradually increased at the rear of the cell to facilitate retraction of cell soma and back processes, allowing the entire cell to move forward. Thus, RhoA expression may be detected at either or both ends of the cell soma depending on the cell’s migratory state. In agreement, we observed varying numbers of DGCs with RhoA accumulation in one or both ends of the cell soma in cases with GCD. Rho GTPase-mediated cell migration is a well-coordinated process during neurodevelopment, tightly regulated by a number of guanine nucleotide exchange factors and dissociation inhibitors, and GTPase-activating proteins to ensure new neurons arrive timely at a specific location (Schmidt and Hall, 2002). Genetic knockout of Rho GTPase-activating proteins, such as Gimp, in animal studies, can lead to the appearance of ectopic cells (Ota et al., 2014). In this study, Gimp was not detected in our samples, but other GTPase-activating proteins, such as ARGAP1, ARGAP35, A-kinase anchor protein 13, SLIT-ROBO Rho GTPase-activating protein 3 were detected, and further studies are needed to investigate their cellular expression and role in regulating DGC migration in MTLE. In our study, a number of *RHOA* and *RAC1*-positive puncta were observed in the neuropil of MTLE cases. It is possible that some of these positive puncta maybe transcripts localized to the processes of DGCs. Previous studies have demonstrated the protein expression of Rac1 and Rho GTPases-exchange factors at the tip of leading processes of migrating cells (Shinohara et al., 2012; Hikita et al., 2014). It is also plausible that *RHOA* and *RAC1* mRNAs are expressed in surrounding glial cells in the granule and molecular layers, although our initial double labeling experiments did not detect *RHOA* and *RAC1* transcripts in astroglial cells expressing glutamine synthetase.

Other modes of cellular migration involving Rho GTPases include “frog leap” and “vessel-based” migrations. Using live imaging techniques, a study reported that 50% of the adult-born new DGCs had leading process pointed tangentially in the subgranular zone of the hippocampal granule cell layer, and these cells underwent lateral migration in small clusters coupled by connexin 43 before migrating radially into the deeper granule cell layer (Wang et al., 2019). During this migratory process, the leading cells in the migratory cluster changed repeatedly (akin to leapfrog), hovering forward and backward as the cluster moved towards their final destination. Other animal studies have also reported that gap junctions are important for neuronal dispersion in the embryonic cortex (Elias et al., 2007, 2010; Yu et al., 2012). In our study, expression of connexin 43 was observed in dispersed DGCs of cases with GCD and we did observe a number of dispersed DGCs in single line formation in outer-granule and molecular layers of cases with GCD, sometimes in close proximity to vascular structures. In microvessel-based migration, DGCs are postulated to migrate tangentially along blood vessels, followed by limited radial migration into the granule cell layer (Sun et al., 2015). In developing animals, it is known that large plexuses of vasculature are developed in the molecular layer and CA4 during postnatal days 0–7, while only short bridges of blood vessels are found to extend through the granule cell layer (Pombero et al., 2018).

## Conclusion

In conclusion, we have shown limited evidence to support ongoing adult neurogenesis in the hippocampus of patients with MTLE, but evidence of differential dysmaturation between dispersed and basal DGCs has been shown. We have provided evidence from proteomic and immunohistochemical studies to demonstrate that DGCs contribute to ongoing structural and synaptic changes in the MTLE human brain, and expression of Rho GTPases in these cells may support abnormal cellular migratory activities that are linked to GCD pathology. Further studies are required to assess the possible contribution of DGCs expressing Rho GTPases to seizure generation and cognitive impairments.

## Data Availability Statement

Peptide identification and quantification data analyzed in this study can be found in the Supplementary Material S9. Any further queries related to data availability should be directed to Professor MT (m.thom@ucl.ac.uk).

## Ethics Statement

The studies involving human participants were reviewed and approved by Epilepsy Society Brain and Tissue Bank (NRES 17/SC/0573). Informed written consents were obtained from all tissue donors.

## Author Contributions

JL, MT, and SS planned and designed the study. KM and this team performed the proteomics analyses. ND and BA-K conducted experiments using *in situ* hybridization and immunohistochemistry, respectively. JL performed bioinformatics and quantitative analyses. All authors contributed to the writing and reviewing of the manuscript.

## Conflict of Interest

The authors declare that the research was conducted in the absence of any commercial or financial relationships that could be construed as a potential conflict of interest.

## Footnotes

^1^https://string-db.org/

^2^https://software.broadinstitute.org/morpheus

## Abbreviations

CA: cornu ammonis
GCD: granule cell dispersion
GCL: granule cell layer
HS ILAE: Type 1 hippocampal sclerosis Type 1 according to International League Against Epilepsy classification system
MOL: molecular layer
MTLE: Mesial Temporal Lobe Epilepsy.
