# Supplementary material for: Granule Cell Dispersion in Human Temporal Lobe Epilepsy: Proteomics Investigation of Neurodevelopmental Migratory Pathways
Source: Front Cell Neurosci. 2020 Mar 17;14:53. doi: 10.3389/fncel.2020.00053 (PMC7090224; doi:10.3389/fncel.2020.00053)
Supplement: Supplementary file 1 [file Data_Sheet_1.pdf]

Supplementary Material 1 Schematic diagram showing basal and dispersed populations of DGCs in the dentate gyrus of MTLE cases with no, moderate or severe (bilaminated) GCD. In this study, the thickness of the GCL was measured in over 19 evenly-spaced points along the GCL. Lines were drawn from basal granule cell layer bordering the CA4 region to furthest dispersed cells, perpendicular to the length of GCL. The distance of 215 µm from basal granule cell layer was arbitrary and was used to denote a midpoint within the molecular cell layer to indicate the inner and outer molecular layers. *Abbreviations:* GCD granule cell dispersion, GCL granule cell layer, MOL molecular layer.

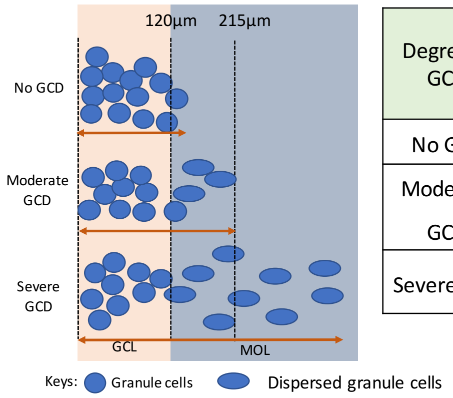

Degree of GCD	Percentage of lines sampled		
	≤120 µm	between 121 and 215 µm	≥ 216 µm
No GCD	Up to 100%	< 10%	0%
Moderate GCD	Up to 90%		< 10%
Severe GCD	Up to 90%		> 10%
