# Supplementary material for: Granule Cell Dispersion in Human Temporal Lobe Epilepsy: Proteomics Investigation of Neurodevelopmental Migratory Pathways
Source: Front Cell Neurosci. 2020 Mar 17;14:53. doi: 10.3389/fncel.2020.00053 (PMC7090224; doi:10.3389/fncel.2020.00053)
Supplement: Supplementary file 2 [file Data_Sheet_2.PDF]

Supplementary Material 2: Proteins that were significantly overexpressed in specific clusters (fold change>1.5, P<0.05) as depicted in Figure 2E and 2H.

**Cluster**	**Entry ID**	**Protein**	**Gene**
Basal	P34932	Heat shock 70 kDa protein 4	HSPA4
Basal	Q15084	Protein disulfide-isomerase A6	PDIA6
Basal	B1AHR1	Neuronal-specific septin-3	SEPT3
Basal	A6NP52	PRA1 family protein 2	PRAF2
Dispersed	P36543	V-type proton ATPase subunit E 1	ATP6V1E1
Dispersed	A0A087WZZ5	Splicing factor 3B subunit 2	SF3B2
Dispersed	A2IDB2	14-3-3 protein eta (Fragment)	YWHAH
Dispersed	P49721	Proteasome subunit beta type-2	PSMB2
Dispersed	B5MCD7	Synaptogyrin-1	SYNGR1
Dispersed	P11277	Spectrin beta chain, erythrocytic	SPTB
Dispersed	P17677	Neuromodulin	GAP43
Dispersed	P18859	ATP synthase-coupling factor 6	ATP5J
Dispersed	Q96JE9	Microtubule-associated protein 6	MAP6
Dispersed	P11137	Microtubule-associated protein 2	MAP2
Dispersed	Q9UPY8	Microtubule-associated protein RP/EB 3	MAPRE3
Dispersed	C9JFR7	Cytochrome C	CYCS
Older	F5GX30	Cation-dependent mannose-6-phosphate receptor	M6PR
Older	A0A087X2B1	RNA binding protein fox-1 homolog	RBFOX1
Older	A2IDB2	14-3-3 protein	YWHAH
Older	P01876	Ig alpha-1 chain C region	IGHA1
Younger	Q15365	Poly(rC)-binding protein 1	PCBP1
Younger	A0A0A6YYC0	Ribosomal protein S6 kinase alpha-4	RPS6KA4
Younger	Q12765	Secernin-1	SCRN1
Younger	P42166	Lamina-associated polypeptide 2, isoform alpha	TMPO
Younger	P29401	Transketolase	TKT
Younger	O75380	NADH dehydrogenase iron-sulfur protein 6	NDUFS6
Younger	P62879	Guanine nucleotide-binding protein subunit beta-2	GNB2
Younger	Q96GW7	Brevican core protein	BCAN
Younger	H0YLA2	Signal recognition particle 14 kDa protein	SRP14
Younger	P17252	Protein kinase C alpha type	PRKCA
Younger	P61020	Ras-related protein Rab-5B	RAB5B
Younger	P05556	Integrin beta-1	ITGB1
Younger	P04350	Tubulin beta-4A chain	TUBB4A
Younger	Q99714	3-hydroxyacyl-CoA dehydrogenase type-2	HSD17B10
Younger	P05455	Lupus La protein	SSB
Younger	Q9H0C2	ADP/ATP translocase 4	SLC25A31
Younger	P05062	Fructose-bisphosphate aldolase B	ALDOB
Younger	P19013	Keratin, type II cytoskeletal 4	KRT4
