# Supplementary material for: Granule Cell Dispersion in Human Temporal Lobe Epilepsy: Proteomics Investigation of Neurodevelopmental Migratory Pathways
Source: Front Cell Neurosci. 2020 Mar 17;14:53. doi: 10.3389/fncel.2020.00053 (PMC7090224; doi:10.3389/fncel.2020.00053)
Supplement: Supplementary file 3 [file Data_Sheet_3.PDF]

Supplementary Material 3: Top eight functional annotation clusters associated with proteins in Dispersed and Basal clusters (Annotation clusters 1-5, P<0.01). Also refer to Figure 2F and 2G.

Annotation cluster	Description	Enrichment Score	P value	Adjusted P values
1	GTP binding and activity	10	1.06E-10	3.67E-08
2	Cell-cell adherens junction and cadherin binding	7	6.75E-09	2.61E-06
3	Small GTPase mediated signal transduction	7	3.19E-07	5.60E-04
4	Ribosome, translation, RNA processing	3	1.46E-05	5.65E-03
5	ATPase, and regulation of cardiac conduction ATPase	3	1.03E-04	4.65E-03
6	GDP and GMP metabolic process	2	7.74E-04	7.44E-01
7	Mitochondrial respiratory chain	2	1.37E-03	4.11E-01
8	Clathrin coat	2	4.06E-03	7.93E-01

Basal cluster

Annotation cluster	Description	Enrichment Score	P value	Adjusted P value
1	Ribosome, translation, RNA processing	18	3.54E-17	1.79E-14
2	Cell-cell adherens junction and cadherin binding	10	3.82E-11	1.93E-08
3	GTP binding and activity	9	1.09E-09	4.31E-07
4	Anaphase-promoting complex-dependent catabolic process, and ubiquitin-protein ligase activity	4	2.16E-05	4.92E-02
5	Regulation of cellular amino acid metabolic process, and proteasome activity	3	1.22E-04	2.07E-02
6	T cell receptor signalling pathway	3	6.86E-05	1.48E-01
7	Wnt signalling pathway	3	4.12E-04	6.18E-01
8	Proteasome core complex	3	1.11E-03	4.30E-01
