# Supplementary material for: Granule Cell Dispersion in Human Temporal Lobe Epilepsy: Proteomics Investigation of Neurodevelopmental Migratory Pathways
Source: Front Cell Neurosci. 2020 Mar 17;14:53. doi: 10.3389/fncel.2020.00053 (PMC7090224; doi:10.3389/fncel.2020.00053)
Supplement: Supplementary file 4 [file Data_Sheet_4.PDF]

Supplementary Material 4: Pathways associated with proteins in Dispersed and Basal clusters (P<0.01).

Databases	Pathways	P-value	Adjusted P-value	Percentage of proteins
**Dispersed Cluster**				
Reactome	VEGFA-VEGFR2 Pathway_R-HSA-4420097	1.64E-09	6.27E-07	7.3
Reactome	Signalling by VEGF_R-HSA-194138	2.64E-09	8.06E-07	7.3
Reactome	Membrane Trafficking_ R-HSA-199991	3.30E-09	8.41E-07	5.0
Reactome	Vesicle-mediated transport_R-HSA-5653656	1.21E-09	9.29E-07	9.3
Reactome	Signalling by Rho GTPases_ R-HSA-194315	4.68E-09	1.02E-06	4.6
Reactome	Axon guidance_R-HSA-422475	8.18E-10	1.25E-06	9.6
Reactome	Developmental Biology_ R-HSA-1266738	1.01E-08	1.92E-06	6.9
KEGG	Huntington disease_K-HSA-05016	1.17E-08	3.62E-06	5.4
KEGG	Alzheimer disease_K-HSA-05010	9.10E-08	1.40E-05	4.8
KEGG	Endocytosis_K-HAS-04144	3.59E-07	3.68E-05	5.4
KEGG	Regulation of actin cytoskeleton_K-HSA-04810	1.63E-06	1.01E-04	4.8
KEGG	Oxidative phosphorylation_K-HSA-00190	1.32E-06	1.02E-04	3.8
KEGG	Parkinson disease	2.65E-06	1.36E-04	2.3
Wikipathway	Cytoplasmic Ribosomal Proteins WP477	1.38E-06	2.17E-04	3.2
Wikipathway	Synaptic Vesicle Pathway WP2267	1.22E-06	2.89E-04	2.6
Wikipathway	Regulation of Actin Cytoskeleton WP51	7.60E-07	3.59E-04	4.2
Wikipathway	Electron Transport Chain WP111	5.27E-06	6.22E-04	3.2
KEGG	Tight junction	1.68E-05	7.40E-04	2.3
Wikipathway	TCA Cycle WP78	1.55E-04	1.00E-02	1.3
**Basal Cluster**				
Reactome	Metabolism R-HSA-1430728	1.65E-29	2.53E-26	24.9
Reactome	Selenocysteine synthesis R-HSA-2408557	3.36E-27	1.71E-24	5.6
Reactome	Eukaryotic Translation Elongation R-HSA-156842	7.58E-27	2.90E-24	5.6
Reactome	Peptide chain elongation R-HSA-156902	2.14E-26	5.46E-24	5.4
Reactome	Viral mRNA Translation R-HSA-192823	2.14E-26	6.55E-24	5.4
Reactome	Selenoamino acid metabolism R-HSA-2408522	5.96E-26	1.30E-23	5.9
Wikipathway	Cytoplasmic Ribosomal Proteins WP477	1.57E-25	7.42E-23	5.4
Reactome	Eukaryotic Translation Termination R-HSA-72764	1.20E-20	2.50E-18	5.9
KEGG	Ribosome K-HSA03010	2.10E-15	5.00E-13	6.1
Wikipathway	Amino Acid metabolism WP3925	9.92E-08	2.34E-05	2.5
Wikipathway	mRNA Processing WP411	1.92E-07	3.02E-05	2.9
Wikipathway	Proteasome Degradation WP183	4.35E-06	5.14E-04	1.8
Wikipathway	Synaptic Vesicle Pathway WP2267	6.11E-06	5.77E-04	1.6
Wikipathway	Electron Transport Chain WP111	8.02E-05	4.73E-03	2.0
Wikipathway	Hippo-Merlin Signaling Dysregulation WP4541	7.34E-05	4.95E-03	2.2
