# Supplementary material for: Granule Cell Dispersion in Human Temporal Lobe Epilepsy: Proteomics Investigation of Neurodevelopmental Migratory Pathways
Source: Front Cell Neurosci. 2020 Mar 17;14:53. doi: 10.3389/fncel.2020.00053 (PMC7090224; doi:10.3389/fncel.2020.00053)

Supplementary Material 5 Diagram illustrating the role of Rho GTPases and their effector proteins in cytoskeletal dynamics. Adapted from Reactome (R-HSA195258.3). Yellow bars refer to the number of proteins in Dispersed cluster that contribute to specific Rho GTPases-effector complex.

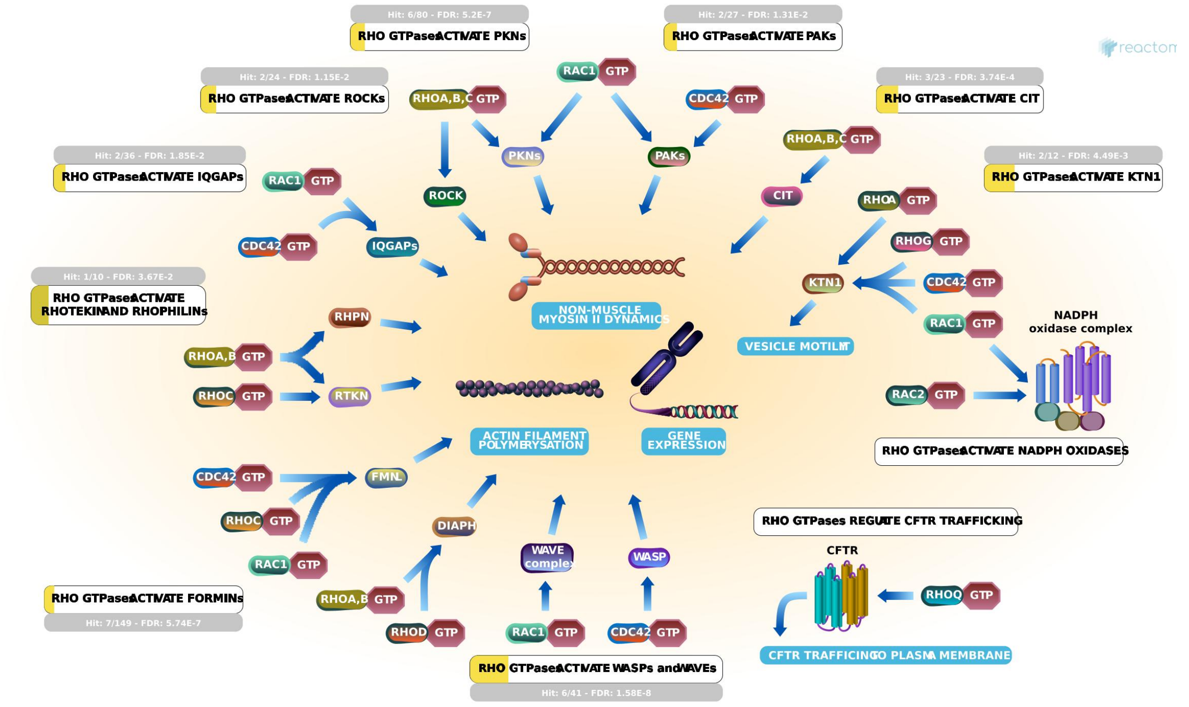

Supplement: Supplementary file 5 [file Data_Sheet_5.PDF]
