# Supplementary material for: Granule Cell Dispersion in Human Temporal Lobe Epilepsy: Proteomics Investigation of Neurodevelopmental Migratory Pathways
Source: Front Cell Neurosci. 2020 Mar 17;14:53. doi: 10.3389/fncel.2020.00053 (PMC7090224; doi:10.3389/fncel.2020.00053)
Supplement: Supplementary file 6 [file Data_Sheet_6.pdf]

Supplementary Material 6: Quantitative data from immunohistochemistry (IHC) and *in situ* hybridisation studies (ISH). *P<0.05.

	RHOA-IHC					RHOA-ISH					RAC-ISH				
Measures	GCD	SEM	No GCD	SEM	P value	GCD	SEM	No GCD	SEM	P value	GCD	SEM	No GCD	SEM	P value
Mean number of labelled cells per μm^2	4.57E-04	8.15E-05	2.04E-04	6.11E-05	0.004*	1.74E-03	2.71E-04	1.25E-03	1.81E-04	0.229	2.05E-03	2.51E-04	1.72E-03	2.27E-04	0.339
GCL	8.80E-04	1.13E-04	4.63E-04	1.25E-04	0.022*	2.83E-03	5.61E-04	2.11E-03	2.23E-04	0.537	3.31E-03	4.47E-04	2.92E-03	3.47E-04	0.779
IML	3.42E-04	3.90E-05	9.11E-05	3.20E-05	0.002*	1.49E-03	1.84E-04	9.25E-04	9.15E-05	0.050*	1.70E-03	2.06E-04	1.23E-03	1.62E-04	0.094
OML	1.49E-04	6.57E-05	5.90E-05	3.42E-05	0.008*	8.97E-04	1.01E-04	7.24E-04	6.94E-05	0.329	1.14E-03	1.24E-04	1.01E-03	8.99E-05	0.23
Mean percentage of cells labelled per μm^2	42.18	3.27	15.92	2.72	0.001*	77.13	3.32	72.29	4.77	0.605	69.39	3.63	65.47	3.35	0.317
GCL	43.73	3.05	23.41	5.96	0.035*	77.75	5.88	73.02	6.07	0.662	78.51	6.80	75.02	5.49	0.397
IML	55.67	2.67	14.11	3.25	0.001*	78.70	4.73	72.91	9.77	0.931	70.25	6.03	62.98	6.41	0.397
OML	27.13	4.69	10.24	3.35	0.008*	74.94	7.37	70.95	10.28	0.933	59.41	4.59	58.41	4.06	0.475
Mean number of ISH-puncta per µm2						7.24E-03	1.36E-03	5.50E-03	1.09E-03	0.275	1.02E-02	2.04E-03	8.85E-03	2.07E-03	0.4
GCL						1.21E-02	3.17E-03	8.21E-03	2.31E-03	0.536	1.78E-02	4.74E-03	1.64E-02	4.96E-03	0.779
IML						6.15E-03	9.41E-04	4.54E-03	1.49E-03	0.429	8.22E-03	2.12E-03	5.90E-03	1.71E-03	0.336
OML						8.85E-04	3.61E-04	3.74E-03	1.42E-03	0.662	4.65E-03	8.74E-04	4.26E-03	9.39E-04	0.417
