# Supplementary material for: Granule Cell Dispersion in Human Temporal Lobe Epilepsy: Proteomics Investigation of Neurodevelopmental Migratory Pathways
Source: Front Cell Neurosci. 2020 Mar 17;14:53. doi: 10.3389/fncel.2020.00053 (PMC7090224; doi:10.3389/fncel.2020.00053)

Supplementary Material 7: Additional results from *in situ* hybridisation studies. (Top) *In situ* hybridisation studies using probes against *CDC42* mRNA in a case with GCD. The image shows *CDC42*-positive puncta (cyan) in DGCs in the outer-granular layer and inner molecular layer. The tissue section was counterstained using haematoxylin (purple). (Bottom) Two images showing glial cells expressing glutamine synthetase (GS; arrowheads) with no or very few *RHOA*-positive puncta. In contrast, numerous *RHOA*-positive puncta were detected in the adjacent larger, neuronal-like cells.

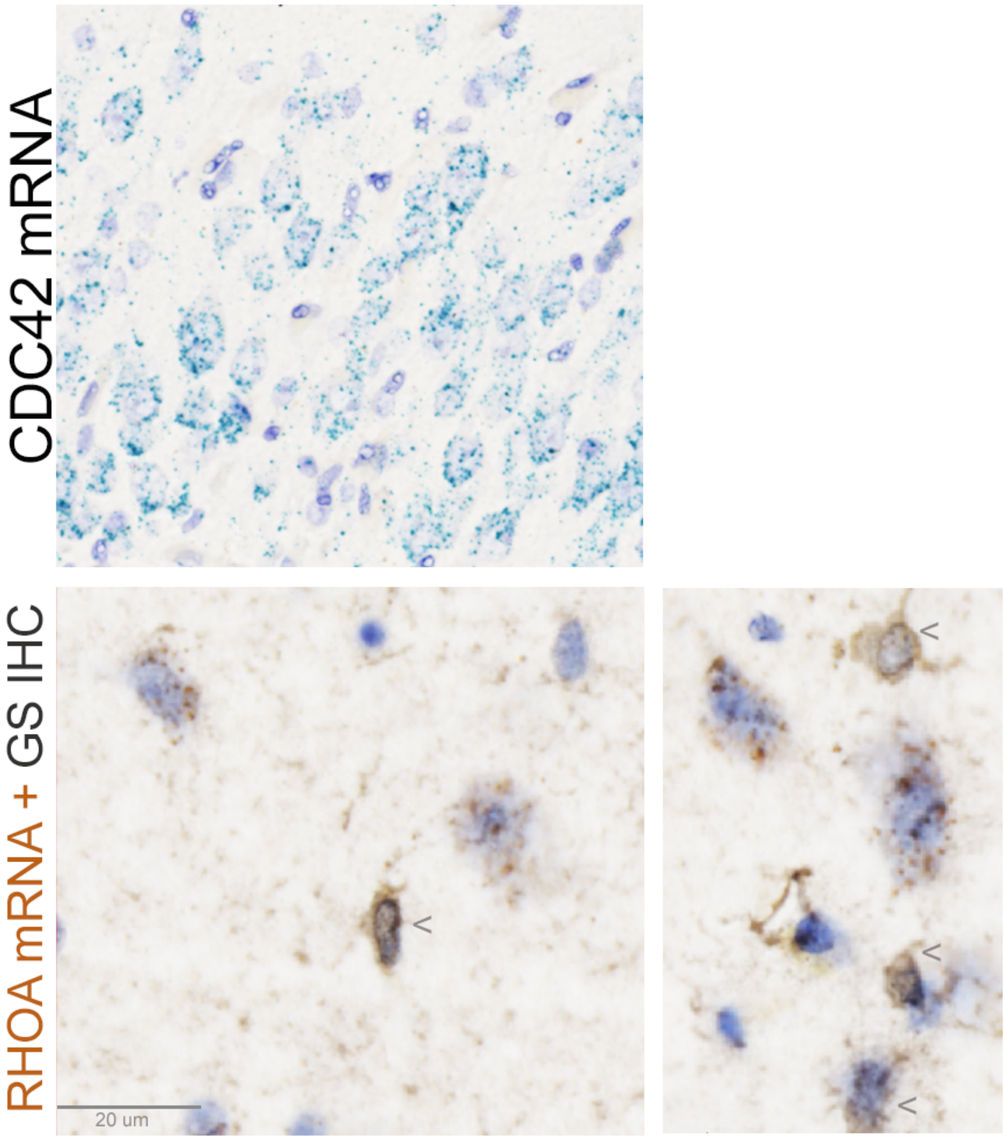

Supplement: Supplementary file 7 [file Data_Sheet_7.PDF]
