# Supplementary material for: Granule Cell Dispersion in Human Temporal Lobe Epilepsy: Proteomics Investigation of Neurodevelopmental Migratory Pathways
Source: Front Cell Neurosci. 2020 Mar 17;14:53. doi: 10.3389/fncel.2020.00053 (PMC7090224; doi:10.3389/fncel.2020.00053)

Supplementary Material 8: Transcriptome expression of *RHOA, RAC1* and *CDC42* in fetal and adult postmortem human brains extracted from the Human Brain Transcriptome (www.hbatlas.org) (Kang et al., 2011). *Periods:* 1 Embryonic 4 PCW; 2,3 Early fetal 8-10 PCW; 4,5 Early mid-fetal 13-16 PCW; 6,7 Late fetal 19-24 PCW; 8 Neonatal and early infancy 0M; 9 Late infancy 6M; 10 Early childhood 1Y; 11 Middle and late childhood 6Y; 12 Adolescence 12Y; 13 Young adulthood 20Y; 14 Middle adulthood 40Y; 15 Late adulthood 60Y. Abbreviations: M postnatal months, PCW post conceptional weeks, Y postnatal years. *Regions:* NCX, neocortex; HIP, hippocampus; AMY, amygdala; STR, striatum; MD, mediodorsal nucleus of the thalamus; CBC, cerebellar cortex.

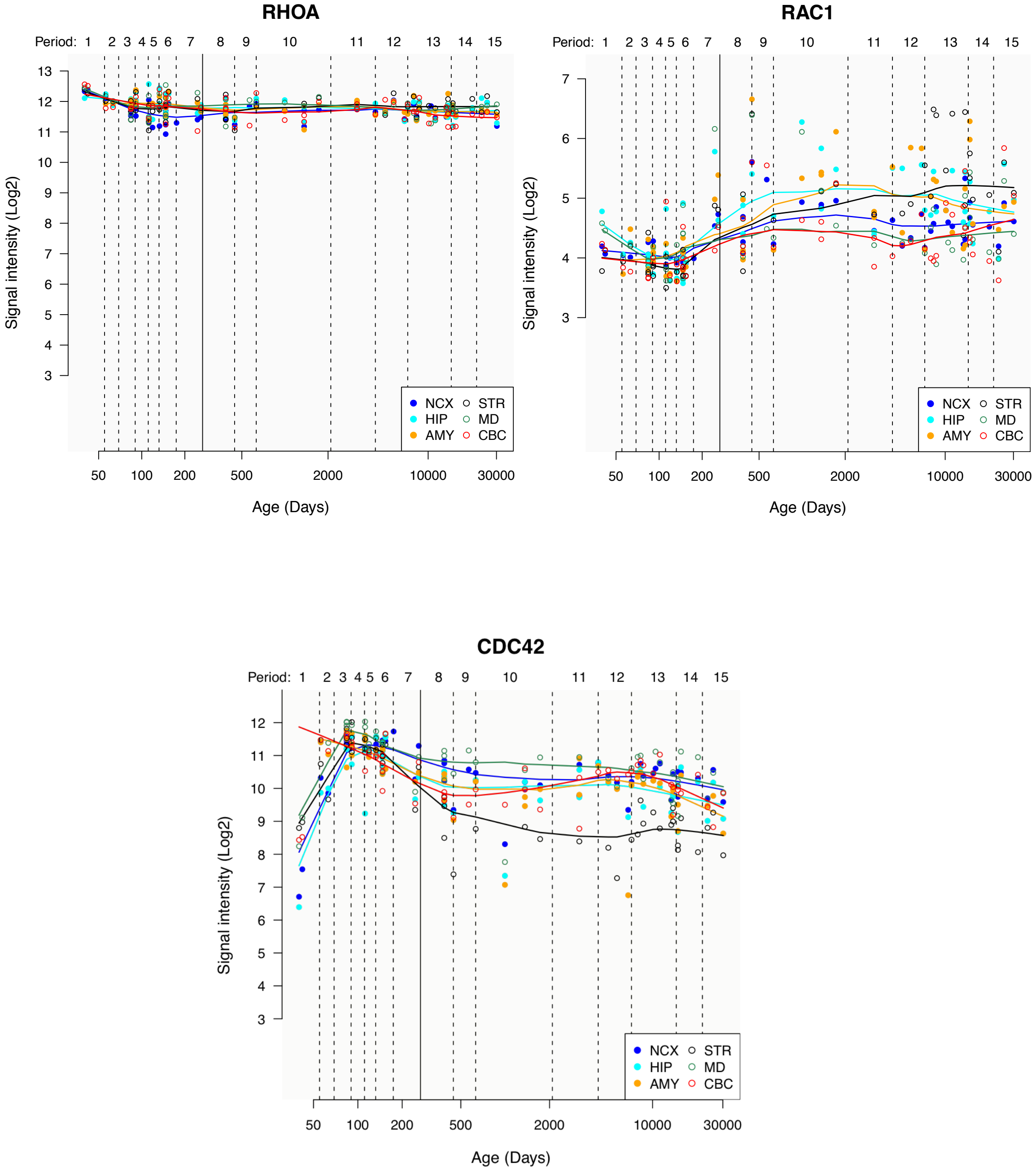

Supplement: Supplementary file 8 [file Data_Sheet_8.PDF]
