# Supplementary material for: Granule Cell Dispersion in Human Temporal Lobe Epilepsy: Proteomics Investigation of Neurodevelopmental Migratory Pathways
Source: Front Cell Neurosci. 2020 Mar 17;14:53. doi: 10.3389/fncel.2020.00053 (PMC7090224; doi:10.3389/fncel.2020.00053)
Supplement: Supplementary file 9 [file Data_Sheet_9.PDF]

| Accession | Description | mW (Da) | pI (pH) | fmol | Basal/Dispersec | Younger/Older |
|---|---|---|---|---|---|---|
| A0A024QZ42 | HCG1985580, isoform CRA_c OS=Homo sapiens GN=PDCD6 PE=1 SV=1 | 14441 | 4.8604 | 0.329 | B | Y |
| A0A024QZX5 | Serpin B6 OS=Homo sapiens GN=SERPINB6 PE=1 SV=1 | 42996 | 5.0039 | 0.003 | B | Y |
| A0A024R3B9 | Alpha-crystallin B chain OS=Homo sapiens GN=CRYAB PE=1 SV=1 | 12245 | 9.2739 | 0.001 | B | Y |
| A0A024R4M0 | 40S ribosomal protein S9 OS=Homo sapiens GN=RPS9 PE=1 SV=1 | 22577 | 11.0874 | 0.001 | B | Y |
| A0A024RA52 | Proteasome subunit alpha type OS=Homo sapiens GN=PSMA2 PE=1 SV=1 | 25882 | 7.2979 | 0.001 | B | Y |
| A0A075B6H6 | Ig kappa chain C region (Fragment) OS=Homo sapiens GN=IGKC PE=1 SV=1 | 11712 | 5.5005 | 0.001 | B | Y |
| A0A075B6K8 | Ig lambda-1 chain C regions (Fragment) OS=Homo sapiens GN=IGLC1 PE=4 SV=1 | 11394 | 7.9878 | 0.001 | B | Y |
| A0A087WT87 | Amino acid transporter OS=Homo sapiens GN=SLC1A3 PE=1 SV=1 | 54172 | 7.2817 | 0.001 | B | Y |
| A0A087WTF6 | Neural cell adhesion molecule 1 OS=Homo sapiens GN=NCAM1 PE=1 SV=1 | 93271 | 4.585 | 1.305 | B | Y |
| A0A087WTG3 | Cullin-3 OS=Homo sapiens GN=CUL3 PE=1 SV=1 | 39122 | 9.8525 | 0.001 | B | Y |
| A0A087WTP3 | Far upstream element-binding protein 2 OS=Homo sapiens GN=KHSRP PE=1 SV=1 | 72982 | 7.5762 | 0.001 | B | Y |
| A0A087WTT1 | Polyadenylate-binding protein OS=Homo sapiens GN=PABPC1 PE=1 SV=1 | 58498 | 9.5977 | 0.175 | B | Y |
| A0A087WUI2 | Heterogeneous nuclear ribonucleoproteins A2/B1 OS=Homo sapiens GN=HNRNPA2B1 PE=1 S | 29816 | 4.7183 | 0.556 | B | Y |
| A0A087WUK2 | Heterogeneous nuclear ribonucleoprotein D-like OS=Homo sapiens GN=HNRNPDL PE=1 SV=1 | 40015 | 10.355 | 0.324 | B | Y |
| A0A087WUL2 | Proteasome subunit beta type-3 (Fragment) OS=Homo sapiens GN=PSMB3 PE=1 SV=1 | 16150 | 8.5708 | 0.001 | B | Y |
| A0A087WUM2 | L-lactate dehydrogenase OS=Homo sapiens GN=LDHAL6A PE=1 SV=1 | 25481 | 7.3198 | 2.049 | B | Y |
| A0A087WUS0 | 40S ribosomal protein S24 OS=Homo sapiens GN=RPS24 PE=1 SV=1 | 15343 | 11.458 | 0.001 | B | Y |
| A0A087WUX6 | Proteasomal ubiquitin receptor ADRM1 (Fragment) OS=Homo sapiens GN=ADRM1 PE=1 SV=1 | 14936 | 6.0674 | 0.156 | B | Y |
| A0A087WUZ3 | Spectrin beta chain, non-erythrocytic 1 OS=Homo sapiens GN=SPTBN1 PE=1 SV=1 | 274657 | 5.2515 | 0.001 | B | Y |
| A0A087WV23 | SH3 domain-binding glutamic acid-rich-like protein 3 OS=Homo sapiens GN=SH3BGRL3 PE=1 | 23771 | 9.3442 | 0.001 | B | Y |
| A0A087WV47 | Ig gamma-1 chain C region OS=Homo sapiens GN=IGHG1 PE=1 SV=1 | 51121 | 7.3813 | 0.093 | B | Y |
| A0A087WV48 | Uncharacterized protein OS=Homo sapiens PE=4 SV=1 | 12672 | 8.5488 | 0.360 | B | Y |
| A0A087WVQ6 | Clathrin heavy chain OS=Homo sapiens GN=CLTC PE=1 SV=1 | 191934 | 5.3555 | 0.001 | B | Y |
| A0A087WW00 | Ubiquitin-conjugating enzyme E2 D1 OS=Homo sapiens GN=UBE2D1 PE=1 SV=1 | 12413 | 7.9834 | 0.001 | B | Y |
| A0A087WW66 | 26S proteasome non-ATPase regulatory subunit 1 OS=Homo sapiens GN=PSMD1 PE=1 SV=1 | 105783 | 5.1123 | 0.001 | B | Y |
| A0A087WWU8 | Tropomyosin alpha-3 chain OS=Homo sapiens GN=TPM3 PE=1 SV=1 | 26404 | 4.5513 | 0.082 | B | Y |
| A0A087WX08 | Gamma-adducin OS=Homo sapiens GN=ADD3 PE=1 SV=1 | 75328 | 6.2915 | 0.269 | B | Y |
| A0A087WX29 | TAR DNA-binding protein 43 (Fragment) OS=Homo sapiens GN=TARDBP PE=1 SV=1 | 26726 | 7.7402 | 0.056 | B | Y |
| A0A087WXC5 | NADH dehydrogenase [ubiquinone] 1 alpha subcomplex subunit 10, mitochondrial OS=Homo sa | 40811 | 8.5957 | 0.001 | B | Y |
| A0A087WXM8 | Basal cell adhesion molecule OS=Homo sapiens GN=BCAM PE=1 SV=1 | 63654 | 5.6763 | 0.001 | B | Y |
| A0A087WY71 | AP-2 complex subunit mu OS=Homo sapiens GN=AP2M1 PE=1 SV=1 | 49495 | 9.8848 | 0.174 | B | Y |
| A0A087WYG8 | Alpha-internexin OS=Homo sapiens GN=INA PE=1 SV=1 | 55031 | 5.1665 | 0.532 | B | Y |
| A0A087WYS1 | UTP--glucose-1-phosphate uridylyltransferase OS=Homo sapiens GN=UGP2 PE=1 SV=1 | 56931 | 7.9468 | 0.001 | B | Y |
| A0A087WYT3 | Prostaglandin E synthase 3 OS=Homo sapiens GN=PTGES3 PE=1 SV=1 | 19142 | 4.147 | 0.001 | B | Y |
| A0A087WZ27 | Zinc finger protein 90 OS=Homo sapiens GN=ZNF90 PE=4 SV=2 | 14385 | 10.5996 | 0.001 | B | Y |
| A0A087WZH7 | Myristoylated alanine-rich C-kinase substrate OS=Homo sapiens GN=MARCKS PE=1 SV=1 | 31577 | 4.5264 | 1.110 | B | Y |
| A0A087WZN1 | Isocitrate dehydrogenase [NAD] subunit, mitochondrial OS=Homo sapiens GN=IDH3B PE=1 SV | 42383 | 8.5854 | 0.001 | B | Y |
| A0A087WZR9 | Pyrroline-5-carboxylate reductase OS=Homo sapiens GN=PYCR2 PE=1 SV=1 | 33616 | 5.5693 | 0.061 | B | Y |
| A0A087WZZ5 | Splicing factor 3B subunit 2 OS=Homo sapiens GN=SF3B2 PE=1 SV=1 | 97524 | 5.3818 | 0.083 | B | Y |
| A0A087X027 | Protein SETSIP OS=Homo sapiens GN=SETSIP PE=3 SV=1 | 33624 | 4.0034 | 0.001 | B | Y |
| A0A087X0X3 | Heterogeneous nuclear ribonucleoprotein M OS=Homo sapiens GN=HNRNPM PE=1 SV=1 | 77518 | 9.1436 | 0.001 | B | Y |
| A0A087X0Z7 | Dehydrogenase/reductase SDR family member 7 OS=Homo sapiens GN=DHRS7 PE=1 SV=1 | 42592 | 8.8726 | 0.001 | B | Y |
| A0A087X1B9 | Ferritin OS=Homo sapiens GN=FTL PE=1 SV=1 | 21169 | 5.6396 | 0.001 | B | Y |
| A0A087X1H6 | Hsc70-interacting protein OS=Homo sapiens GN=ST13 PE=1 SV=1 | 28220 | 9.0073 | 0.001 | B | Y |
| A0A087X1K9 | Acyl-protein thioesterase 1 OS=Homo sapiens GN=LYPLA1 PE=1 SV=1 | 17969 | 4.7021 | 0.001 | B | Y |
| A0A087X294 | Non-specific serine/threonine protein kinase OS=Homo sapiens GN=PAK3 PE=1 SV=1 | 60588 | 5.165 | 0.261 | B | Y |
| A0A087X2B1 | RNA binding protein fox-1 homolog OS=Homo sapiens GN=RBFOX1 PE=4 SV=1 | 40284 | 6.624 | 0.001 | B | Y |
| A0A087X2D0 | Serine/arginine-rich-splicing factor 3 OS=Homo sapiens GN=SRSF3 PE=1 SV=1 | 10313 | 4.7959 | 0.518 | B | Y |
| A0A087X2G1 | ATP-dependent RNA helicase DDX1 OS=Homo sapiens GN=DDX1 PE=1 SV=1 | 73928 | 7.5747 | 0.001 | B | Y |
| A0A087X2H1 | E3 ubiquitin-protein ligase HECTD1 OS=Homo sapiens GN=HECTD1 PE=1 SV=1 | 289449 | 5.1064 | 0.001 | B | Y |
| A0A088AWN7 | Kin of IRRE-like protein 3 OS=Homo sapiens GN=KIRREL3 PE=1 SV=1 | 80331 | 6.0439 | 0.027 | B | Y |
| A0A096LNZ9 | Ubiquitin-like protein ISG15 (Fragment) OS=Homo sapiens GN=ISG15 PE=1 SV=4 | 15552 | 5.855 | 0.146 | B | Y |
| A0A096LP12 | ES1 protein homolog, mitochondrial (Fragment) OS=Homo sapiens GN=LOC102724023 PE=4 S | 23849 | 9.4292 | 0.001 | B | Y |
| A0A096LP52 | Syntaxin-binding protein 1 (Fragment) OS=Homo sapiens GN=STXBP1 PE=1 SV=1 | 13558 | 6.6167 | 0.069 | B | Y |
| A0A0A0MR85 | Glutathione S-transferase Mu 4 OS=Homo sapiens GN=GSTM4 PE=1 SV=1 | 25546 | 5.502 | 0.001 | B | Y |
| A0A0A0MRA8 | Band 4.1-like protein 3 OS=Homo sapiens GN=EPB41L3 PE=1 SV=1 | 102225 | 5.0361 | 0.126 | B | Y |
| A0A0A0MRF6 | A-kinase anchor protein 9 OS=Homo sapiens GN=AKAP9 PE=1 SV=1 | 453212 | 4.7534 | 2.158 | B | Y |
| A0A0A0MRJ6 | Protein-L-isoaspartate O-methyltransferase OS=Homo sapiens GN=PCMT1 PE=1 SV=1 | 30295 | 7.314 | 0.001 | B | Y |
| A0A0A0MRX1 | ELAV-like protein OS=Homo sapiens GN=ELAVL2 PE=1 SV=1 | 42605 | 9.2974 | 0.001 | B | Y |
| A0A0A0MS41 | Sideroflexin OS=Homo sapiens GN=SFXN3 PE=1 SV=1 | 35480 | 9.3223 | 0.270 | B | Y |
| A0A0A0MS51 | Gelsolin OS=Homo sapiens GN=GSN PE=1 SV=1 | 82474 | 5.2896 | 1.012 | B | Y |
| A0A0A0MS54 | cAMP-dependent protein kinase catalytic subunit beta OS=Homo sapiens GN=PRKACB PE=1 S | 41282 | 8.8125 | 0.001 | B | Y |
| A0A0A0MS87 | Protein NDRG2 OS=Homo sapiens GN=NDRG2 PE=1 SV=1 | 39519 | 6.5845 | 0.747 | B | Y |
| A0A0A0MSE2 | Hydroxyacyl-coenzyme A dehydrogenase, mitochondrial OS=Homo sapiens GN=HADH PE=1 S | 42096 | 9.6475 | 0.001 | B | Y |
| A0A0A0MSI0 | Peroxiredoxin-1 (Fragment) OS=Homo sapiens GN=PRDX1 PE=1 SV=1 | 18963 | 6.4907 | 2.852 | B | Y |
| A0A0A0MT26 | Sodium/potassium-transporting ATPase subunit alpha-3 OS=Homo sapiens GN=ATP1A3 PE=1 | 133231 | 5.6865 | 0.001 | B | Y |
| A0A0A0MT35 | Prostamide/prostaglandin F synthase OS=Homo sapiens GN=FAM213B PE=1 SV=1 | 26393 | 6.5874 | 0.001 | B | Y |
| A0A0A0MT83 | Isovaleryl-CoA dehydrogenase, mitochondrial OS=Homo sapiens GN=IVD PE=1 SV=1 | 46620 | 8.001 | 0.001 | B | Y |
| A0A0A0MTI5 | Acyl-CoA-binding protein OS=Homo sapiens GN=DBI PE=1 SV=1 | 15948 | 4.7886 | 0.001 | B | Y |
| A0A0A0MTJ9 | Neutral cholesterol ester hydrolase 1 OS=Homo sapiens GN=NCEH1 PE=1 SV=1 | 49849 | 6.8306 | 0.001 | B | Y |
| A0A0A0MTS2 | Glucose-6-phosphate isomerase (Fragment) OS=Homo sapiens GN=GPI PE=1 SV=1 | 64784 | 9.3516 | 0.001 | B | Y |
| A0A0A6YYA0 | Protein TMED7-TICAM2 OS=Homo sapiens GN=TMED7-TICAM2 PE=3 SV=1 | 21219 | 5.7759 | 0.583 | B | Y |
| A0A0A6YYC0 | Ribosomal protein S6 kinase alpha-4 (Fragment) OS=Homo sapiens GN=RPS6KA4 PE=1 SV=3 | 78317 | 7.3594 | 0.520 | B | Y |
| A0A0A6YYG9 | Protein ARPC4-TTLL3 OS=Homo sapiens GN=ARPC4-TTLL3 PE=4 SV=1 | 71672 | 5.4873 | 1.009 | B | Y |
| A0A0B4J1R6 | Transketolase OS=Homo sapiens GN=TKT PE=1 SV=1 | 49878 | 7.8545 | 0.487 | B | Y |
| A0A0B4J2A4 | 3-ketoacyl-CoA thiolase, mitochondrial OS=Homo sapiens GN=ACAA2 PE=1 SV=1 | 41575 | 7.8501 | 0.392 | B | Y |
| A0A0B4J2C3 | Translationally-controlled tumor protein OS=Homo sapiens GN=TPT1 PE=1 SV=1 | 22559 | 4.9395 | 0.001 | B | Y |
| A0A0C4DFU1 | Superoxide dismutase OS=Homo sapiens GN=SOD2 PE=1 SV=1 | 20710 | 8.4067 | 0.001 | B | Y |
| A0A0C4DFU2 | Superoxide dismutase OS=Homo sapiens GN=SOD2 PE=1 SV=1 | 24734 | 8.4038 | 0.001 | B | Y |
| A0A0C4DG17 | 40S ribosomal protein SA OS=Homo sapiens GN=RPSA PE=1 SV=1 | 33292 | 4.5938 | 0.001 | B | Y |

| | | | | | | |
|---|---|---|---|---|---|---|
| A0A0C4DGS1 | Dolichyl-diphosphooligosaccharide--protein glycosyltransferase 48 kDa subunit OS=Homo sapie | 48768 | 5.3042 | 0.706 | B | Y |
| A0A0C4DGY8 | Enolase-phosphatase E1 OS=Homo sapiens GN=ENOPH1 PE=1 SV=1 | 16522 | 5.9253 | 0.129 | B | Y |
| A0A0C4DGZ9 | Tripeptidyl-peptidase 1 OS=Homo sapiens GN=TPP1 PE=1 SV=1 | 60420 | 5.9268 | 1.003 | B | Y |
| A0A0C4DH22 | Band 4.1-like protein 1 OS=Homo sapiens GN=EPB41L1 PE=1 SV=1 | 98314 | 5.2866 | 0.108 | B | Y |
| A0A0D9SF53 | ATP-dependent RNA helicase DDX3X OS=Homo sapiens GN=DDX3X PE=1 SV=1 | 81426 | 8.0039 | 0.001 | B | Y |
| A0A0D9SF54 | Spectrin alpha chain, non-erythrocytic 1 OS=Homo sapiens GN=SPTAN1 PE=1 SV=1 | 282660 | 5.0493 | 2.425 | B | Y |
| A0A0D9SFB1 | Dynamin-1 OS=Homo sapiens GN=DNM1 PE=1 SV=1 | 93958 | 6.1934 | 0.682 | B | Y |
| A0A0D9SGJ6 | Synaptojanin-1 OS=Homo sapiens GN=SYNJ1 PE=1 SV=1 | 168052 | 6.832 | 0.001 | B | Y |
| A0A0G2JHA9 | Myelin-oligodendrocyte glycoprotein OS=Homo sapiens GN=MOG PE=1 SV=1 | 25337 | 8.4067 | 0.001 | B | Y |
| A0A0G2JQ41 | Active breakpoint cluster region-related protein (Fragment) OS=Homo sapiens GN=ABR PE=1 S | 88802 | 7.1089 | 0.001 | B | Y |
| A0A0J9YWK4 | Hemoglobin subunit beta OS=Homo sapiens GN=HBB PE=4 SV=1 | 5987 | 7.0122 | 3.221 | B | Y |
| A0A0J9YX62 | DnaJ homolog subfamily B member 6 OS=Homo sapiens GN=DNAJB6 PE=4 SV=1 | 36634 | 6.7852 | 0.001 | B | Y |
| A0A0J9YX66 | CUGBP Elav-like family member 2 OS=Homo sapiens GN=CELF2 PE=4 SV=1 | 54879 | 8.9297 | 0.001 | B | Y |
| A0A0J9YY01 | Unconventional myosin-XVB OS=Homo sapiens GN=MYO15B PE=4 SV=1 | 333513 | 7.481 | 6.049 | B | Y |
| A0AVT1 | Ubiquitin-like modifier-activating enzyme 6 OS=Homo sapiens GN=UBA6 PE=1 SV=1 | 117895 | 5.7041 | 0.279 | B | Y |
| A1L0T0 | Acetolactate synthase-like protein OS=Homo sapiens GN=ILVBL PE=1 SV=2 | 67824 | 8.0742 | 0.001 | B | Y |
| A1L390 | Pleckstrin homology domain-containing family G member 3 OS=Homo sapiens GN=PLEKHG3 F | 134329 | 6.104 | 0.080 | B | Y |
| A2A274 | Aconitate hydratase, mitochondrial OS=Homo sapiens GN=ACO2 PE=1 SV=1 | 87765 | 6.9858 | 0.001 | B | Y |
| A2A2D0 | Stathmin (Fragment) OS=Homo sapiens GN=STMN1 PE=1 SV=6 | 9787 | 7.7842 | 0.516 | B | Y |
| A2IDB2 | 14-3-3 protein eta (Fragment) OS=Homo sapiens GN=YWHAH PE=1 SV=1 | 18663 | 7.1704 | 0.040 | B | Y |
| A6NC98 | Coiled-coil domain-containing protein 88B OS=Homo sapiens GN=CCDC88B PE=1 SV=1 | 164709 | 4.9043 | 4.599 | B | Y |
| A6NEC2 | Puromycin-sensitive aminopeptidase-like protein OS=Homo sapiens GN=NPEPPSL1 PE=2 SV= | 53713 | 4.9966 | 0.151 | B | Y |
| A6NG10 | WW domain-binding protein 2 OS=Homo sapiens GN=WBP2 PE=1 SV=2 | 25790 | 5.6279 | 0.001 | B | Y |
| A6NKB8 | Aminopeptidase B OS=Homo sapiens GN=RNPEP PE=1 SV=1 | 68125 | 5.5986 | 0.151 | B | Y |
| A6NL76 | Actin, alpha skeletal muscle OS=Homo sapiens GN=ACTA1 PE=1 SV=3 | 28147 | 5.644 | 0.001 | B | Y |
| A6NLN1 | Polypyrimidine tract binding protein 1, isoform CRA_b OS=Homo sapiens GN=PTBP1 PE=1 SV | 56475 | 9.7324 | 0.001 | B | Y |
| A6NMH8 | Tetraspanin OS=Homo sapiens GN=CD81 PE=1 SV=1 | 29786 | 6.5288 | 0.001 | B | Y |
| A6NNI4 | Tetraspanin OS=Homo sapiens GN=CD9 PE=1 SV=1 | 17751 | 5.877 | 0.404 | B | Y |
| A6NP52 | PRA1 family protein 2 OS=Homo sapiens GN=PRAF2 PE=1 SV=1 | 17177 | 8.9077 | 0.190 | B | Y |
| A8MRB1 | Protein S100-B OS=Homo sapiens GN=S100B PE=1 SV=1 | 10336 | 8.2588 | 0.001 | B | Y |
| A8MT02 | Small nuclear ribonucleoprotein-associated proteins B and B' OS=Homo sapiens GN=SNRPB P | 29955 | 10.5791 | 0.001 | B | Y |
| A8MU27 | Small ubiquitin-related modifier 3 OS=Homo sapiens GN=SUMO3 PE=1 SV=1 | 16945 | 10.106 | 0.001 | B | Y |
| A8MUS3 | 60S ribosomal protein L23a OS=Homo sapiens GN=RPL23A PE=1 SV=1 | 21902 | 10.916 | 0.001 | B | Y |
| A8MXP9 | Matrin-3 OS=Homo sapiens GN=MATR3 PE=1 SV=1 | 99905 | 5.6309 | 0.001 | B | Y |
| A8MZH3 | Myelin basic protein OS=Homo sapiens GN=MBP PE=1 SV=1 | 19717 | 11.7583 | 0.001 | B | Y |
| B1AHC9 | X-ray repair cross-complementing protein 6 OS=Homo sapiens GN=XRCC6 PE=1 SV=1 | 64243 | 9.6416 | 0.245 | B | Y |
| B1AHD1 | Protein SNU13 OS=Homo sapiens GN=SNU13 PE=1 SV=1 | 14617 | 8.6323 | 0.001 | B | Y |
| B1AHR1 | Neuronal-specific septin-3 OS=Homo sapiens GN=SEPT3 PE=1 SV=1 | 33580 | 6.5142 | 0.245 | B | Y |
| B1AJQ6 | Syntaxin-12 (Fragment) OS=Homo sapiens GN=STX12 PE=1 SV=2 | 24561 | 4.8911 | 0.073 | B | Y |
| B1AJZ9 | Forkhead-associated domain-containing protein 1 OS=Homo sapiens GN=FHAD1 PE=2 SV=2 | 161803 | 6.5068 | 1.063 | B | Y |
| B1AK87 | Capping protein (Actin filament) muscle Z-line, beta, isoform CRA_a OS=Homo sapiens GN=CA | 29276 | 6.5098 | 0.089 | B | Y |
| B1AKR6 | Dynein light chain roadblock-type 1 OS=Homo sapiens GN=DYNLRB1 PE=1 SV=1 | 16242 | 6.8276 | 0.001 | B | Y |
| B1AKY9 | Sodium/potassium-transporting ATPase subunit alpha OS=Homo sapiens GN=ATP1A2 PE=1 S | 110791 | 5.2749 | 0.208 | B | Y |
| B1ALA9 | Ribose-phosphate pyrophosphokinase 1 OS=Homo sapiens GN=PRPS1 PE=1 SV=1 | 24068 | 7.6055 | 0.139 | B | Y |
| B1AMS2 | Septin 6, isoform CRA_b OS=Homo sapiens GN=SEPT6 PE=1 SV=1 | 49272 | 6.3589 | 1.190 | B | Y |
| B1B1G2 | Myelin proteolipid protein (Fragment) OS=Homo sapiens GN=PLP1 PE=1 SV=6 | 12203 | 5.4258 | 1.078 | B | Y |
| B2R4S9 | Histone H2B OS=Homo sapiens GN=HIST1H2BC PE=2 SV=1 | 13897 | 10.7402 | 0.001 | B | Y |
| B2R5W2 | Heterogeneous nuclear ribonucleoproteins C1/C2 OS=Homo sapiens GN=HNRNPC PE=1 SV= | 31928 | 4.9087 | 0.811 | B | Y |
| B4DDC6 | Prostaglandin E synthase 3 OS=Homo sapiens GN=PTGES3 PE=1 SV=1 | 14563 | 3.8906 | 0.115 | B | Y |
| B4DDD6 | Drebrin-like protein OS=Homo sapiens GN=DBNL PE=1 SV=1 | 45718 | 4.7373 | 0.058 | B | Y |
| B4DEB1 | Histone H3 OS=Homo sapiens GN=H3F3A PE=1 SV=1 | 14043 | 11.7466 | 0.001 | B | Y |
| B4DGU4 | Catenin beta-1 OS=Homo sapiens GN=CTNNB1 PE=1 SV=1 | 84711 | 5.499 | 0.148 | B | Y |
| B4DJV2 | Citrate synthase OS=Homo sapiens GN=CS PE=1 SV=1 | 50399 | 7.8604 | 0.692 | B | Y |
| B4DR61 | Protein transport protein Sec61 subunit alpha isoform 1 OS=Homo sapiens GN=SEC61A1 PE=' | 52914 | 8.2397 | 0.001 | B | Y |
| B4DUR8 | T-complex protein 1 subunit gamma OS=Homo sapiens GN=CCT3 PE=1 SV=1 | 55638 | 5.3145 | 0.284 | B | Y |
| B4DV12 | Polyubiquitin-B OS=Homo sapiens GN=UBB PE=1 SV=1 | 17204 | 7.3169 | 0.001 | B | Y |
| B4DW73 | Phosphoenolpyruvate carboxykinase [GTP], mitochondrial OS=Homo sapiens GN=PCK2 PE=1 | 55917 | 6.6812 | 0.001 | B | Y |
| B4DY09 | Interleukin enhancer-binding factor 2 OS=Homo sapiens GN=ILF2 PE=1 SV=1 | 38886 | 4.6963 | 2.377 | B | Y |
| B4DYP1 | N(G),N(G)-dimethylarginine dimethylaminohydrolase 1 OS=Homo sapiens GN=DDAH1 PE=2 S' | 20521 | 5.2456 | 0.457 | B | Y |
| B5MCD7 | Synaptogyrin-1 OS=Homo sapiens GN=SYNGR1 PE=1 SV=1 | 18453 | 6.8643 | 0.317 | B | Y |
| B5MD17 | Chromobox protein homolog 1 (Fragment) OS=Homo sapiens GN=CBX1 PE=1 SV=1 | 19313 | 4.7695 | 0.001 | B | Y |
| B7WNR0 | Serum albumin OS=Homo sapiens GN=ALB PE=1 SV=1 | 56175 | 6.8027 | 5.205 | B | Y |
| B7Z264 | Disks large homolog 2 OS=Homo sapiens GN=DLG2 PE=1 SV=1 | 87841 | 5.6089 | 0.252 | B | Y |
| B7Z2L8 | Coiled-coil domain-containing protein 25 OS=Homo sapiens GN=CCDC25 PE=1 SV=1 | 10563 | 5.3203 | 0.001 | B | Y |
| B7Z4C8 | 60S ribosomal protein L31 OS=Homo sapiens GN=RPL31 PE=1 SV=1 | 15109 | 10.7959 | 0.001 | B | Y |
| B7Z4M1 | Reticulon OS=Homo sapiens GN=RTN3 PE=1 SV=1 | 12738 | 7.8545 | 0.230 | B | Y |
| B7Z596 | Tropomyosin alpha-1 chain OS=Homo sapiens GN=TPM1 PE=1 SV=1 | 31733 | 4.6582 | 0.001 | B | Y |
| B7Z613 | Neuronal membrane glycoprotein M6-b OS=Homo sapiens GN=GPM6B PE=1 SV=1 | 33250 | 5.9575 | 0.622 | B | Y |
| B7Z6Z4 | Myosin light polypeptide 6 OS=Homo sapiens GN=MYL6 PE=1 SV=1 | 26689 | 4.8149 | 0.001 | B | Y |
| B7ZAR1 | T-complex protein 1 subunit epsilon OS=Homo sapiens GN=CCT5 PE=1 SV=1 | 55313 | 5.1577 | 0.001 | B | Y |
| B7ZC38 | Endophilin-B2 OS=Homo sapiens GN=SH3GLB2 PE=1 SV=1 | 44333 | 5.4829 | 0.001 | B | Y |
| B8ZZ51 | Malate dehydrogenase, cytoplasmic OS=Homo sapiens GN=MDH1 PE=1 SV=1 | 18677 | 5.5532 | 0.610 | B | Y |
| B8ZZB8 | CB1 cannabinoid receptor-interacting protein 1 OS=Homo sapiens GN=CNRIP1 PE=1 SV=1 | 15158 | 9.7544 | 0.752 | B | Y |
| B9A041 | Malate dehydrogenase, cytoplasmic OS=Homo sapiens GN=MDH1 PE=1 SV=1 | 23023 | 7.2305 | 0.404 | B | Y |
| B9A067 | MICOS complex subunit MIC60 OS=Homo sapiens GN=IMMT PE=1 SV=2 | 78925 | 6.6357 | 0.378 | B | Y |
| B9ZVP7 | 60S ribosomal protein L23 OS=Homo sapiens GN=RPL23 PE=1 SV=1 | 12091 | 11.1475 | 0.001 | B | Y |
| C9IZG4 | Protein CutA OS=Homo sapiens GN=CUTA PE=1 SV=1 | 14391 | 5.1709 | 0.130 | B | Y |
| C9J0K6 | Sorcin OS=Homo sapiens GN=SRI PE=1 SV=1 | 17593 | 5.1929 | 0.315 | B | Y |
| C9J1Z8 | ADP-ribosylation factor 5 (Fragment) OS=Homo sapiens GN=ARF5 PE=1 SV=1 | 17095 | 7.3125 | 1.616 | B | Y |
| C9J2N0 | Profilin OS=Homo sapiens GN=PFN2 PE=1 SV=1 | 13551 | 5.644 | 0.501 | B | Y |
| C9J4P8 | Peptidyl-prolyl cis-trans isomerase (Fragment) OS=Homo sapiens GN=FKBP9 PE=1 SV=6 | 24591 | 5.3145 | 0.132 | B | Y |

| | | | | | | |
|---|---|---|---|---|---|---|
| C9J4Z3 | 60S ribosomal protein L37a OS=Homo sapiens GN=RPL37A PE=1 SV=1 | 7618 | 10.4429 | 4.967 | B | Y |
| C9J634 | Pyruvate dehydrogenase E1 component subunit beta, mitochondrial OS=Homo sapiens GN=PD | 38380 | 7.0518 | 0.207 | B | Y |
| C9J6P4 | Zinc finger CCCH-type antiviral protein 1 OS=Homo sapiens GN=ZC3HAV1 PE=1 SV=1 | 114013 | 8.5151 | 0.001 | B | Y |
| C9J712 | Profilin-2 OS=Homo sapiens GN=PFN2 PE=1 SV=1 | 9791 | 9.7266 | 0.262 | B | Y |
| C9J8I8 | Protein Wnt (Fragment) OS=Homo sapiens GN=WNT5A PE=1 SV=1 | 23958 | 8.6631 | 0.066 | B | Y |
| C9J8P9 | Clathrin light chain A OS=Homo sapiens GN=CLTA PE=1 SV=1 | 21089 | 4.3286 | 0.346 | B | Y |
| C9J9W2 | LIM and SH3 domain protein 1 (Fragment) OS=Homo sapiens GN=LASP1 PE=1 SV=1 | 18968 | 9.3428 | 0.223 | B | Y |
| C9JDR0 | Sterol-4-alpha-carboxylate 3-dehydrogenase, decarboxylating (Fragment) OS=Homo sapiens G | 28128 | 6.0835 | 0.339 | B | Y |
| C9JFR7 | Cytochrome c (Fragment) OS=Homo sapiens GN=CYCS PE=1 SV=1 | 11325 | 10.0488 | 0.796 | B | Y |
| C9JIZ6 | Prosaposin OS=Homo sapiens GN=PSAP PE=1 SV=2 | 58402 | 4.8853 | 0.001 | B | Y |
| C9JJS3 | Phosphoribosyl pyrophosphate synthase-associated protein 2 (Fragment) OS=Homo sapiens G | 21746 | 9.3472 | 0.066 | B | Y |
| C9JJT5 | Protein ATP5J2-PTCD1 OS=Homo sapiens GN=ATP5J2-PTCD1 PE=4 SV=2 | 5911 | 9.7969 | 0.749 | B | Y |
| C9JME2 | FERM, RhoGEF and pleckstrin domain-containing protein 1 OS=Homo sapiens GN=FARP1 PE | 122018 | 8.5327 | 0.001 | B | Y |
| C9JNV3 | U6 snRNA-associated Sm-like protein LSm8 (Fragment) OS=Homo sapiens GN=LSM8 PE=1 S | 8262 | 4.8984 | 0.001 | B | Y |
| C9JNW5 | 60S ribosomal protein L24 OS=Homo sapiens GN=RPL24 PE=1 SV=1 | 17531 | 11.2104 | 0.001 | B | Y |
| C9JQS9 | Propionyl-CoA carboxylase beta chain, mitochondrial OS=Homo sapiens GN=PCCB PE=1 SV= | 60698 | 7.894 | 0.001 | B | Y |
| C9JRG0 | Hemoglobin subunit delta (Fragment) OS=Homo sapiens GN=HBD PE=1 SV=1 | 9800 | 8.4785 | 0.336 | B | Y |
| C9JYY6 | Neuronal cell adhesion molecule OS=Homo sapiens GN=NRCAM PE=1 SV=3 | 133729 | 5.4082 | 0.212 | B | Y |
| C9K0J5 | Ras association (RalGDS/AF-6) and pleckstrin homology domains 1, isoform CRA_b OS=Homo | 141091 | 9.1523 | 8.471 | B | Y |
| D6R9P3 | Heterogeneous nuclear ribonucleoprotein A/B OS=Homo sapiens GN=HNRNPAB PE=1 SV=1 | 30283 | 8.1401 | 0.037 | B | Y |
| D6RA82 | Annexin OS=Homo sapiens GN=ANXA3 PE=1 SV=1 | 32098 | 5.5342 | 0.336 | B | Y |
| D6RAN4 | 60S ribosomal protein L9 (Fragment) OS=Homo sapiens GN=RPL9 PE=1 SV=6 | 20762 | 10.6172 | 0.001 | B | Y |
| D6RAT0 | 40S ribosomal protein S3a OS=Homo sapiens GN=RPS3A PE=1 SV=1 | 25870 | 9.8423 | 0.001 | B | Y |
| D6RDU5 | Septin-11 (Fragment) OS=Homo sapiens GN=SEPT11 PE=1 SV=1 | 43085 | 5.8755 | 0.284 | B | Y |
| D6RE83 | Ubiquitin carboxyl-terminal hydrolase OS=Homo sapiens GN=UCHL1 PE=1 SV=1 | 23159 | 5.1812 | 1.441 | B | Y |
| D6RER5 | Septin-11 OS=Homo sapiens GN=SEPT11 PE=1 SV=1 | 49777 | 6.23 | 0.001 | B | Y |
| D6REX3 | Protein transport protein Sec31A OS=Homo sapiens GN=SEC31A PE=1 SV=1 | 136140 | 6.5083 | 0.001 | B | Y |
| D6RFM5 | Succinate dehydrogenase [ubiquinone] flavoprotein subunit, mitochondrial OS=Homo sapiens G | 63526 | 6.8394 | 0.213 | B | Y |
| D6RGV5 | Cytochrome c oxidase subunit 7A2, mitochondrial (Fragment) OS=Homo sapiens GN=COX7A2 | 11504 | 10.2715 | 0.001 | B | Y |
| D6RHH4 | Guanine nucleotide-binding protein subunit beta-2-like 1 OS=Homo sapiens GN=GNB2L1 PE=1 | 25853 | 9.4731 | 0.146 | B | Y |
| E2QRB3 | Pyrroline-5-carboxylate reductase 1, isoform CRA_c OS=Homo sapiens GN=PYCR1 PE=1 SV= | 30193 | 6.6533 | 0.001 | B | Y |
| E5KLJ5 | Dynamin-like 120 kDa protein, mitochondrial OS=Homo sapiens GN=OPA1 PE=1 SV=1 | 117669 | 7.5952 | 0.001 | B | Y |
| E5RGN3 | Copper transport protein ATOX1 OS=Homo sapiens GN=ATOX1 PE=1 SV=1 | 6326 | 7.9966 | 0.001 | B | Y |
| E5RH81 | Carbonic anhydrase 1 (Fragment) OS=Homo sapiens GN=CA1 PE=1 SV=6 | 19224 | 6.9126 | 0.226 | B | Y |
| E5RHK8 | Dynamin-3 OS=Homo sapiens GN=DNM3 PE=1 SV=1 | 72844 | 8.7495 | 0.049 | B | Y |
| E5RI56 | Uncharacterized protein (Fragment) OS=Homo sapiens PE=1 SV=1 | 10264 | 4.0239 | 0.135 | B | Y |
| E5RI99 | 60S ribosomal protein L30 (Fragment) OS=Homo sapiens GN=RPL30 PE=1 SV=1 | 12647 | 9.873 | 0.110 | B | Y |
| E5RJ29 | PH and SEC7 domain-containing protein 3 OS=Homo sapiens GN=PSD3 PE=1 SV=1 | 108923 | 5.6528 | 0.189 | B | Y |
| E5RJH0 | Betaine--homocysteine S-methyltransferase 1 OS=Homo sapiens GN=BHMT PE=1 SV=1 | 28188 | 9.2139 | 0.609 | B | Y |
| E5RJR5 | S-phase kinase-associated protein 1 OS=Homo sapiens GN=SKP1 PE=1 SV=1 | 18708 | 4.3696 | 0.001 | B | Y |
| E5RJU9 | Protein LYRIC OS=Homo sapiens GN=MTDH PE=1 SV=1 | 57486 | 10.0298 | 0.082 | B | Y |
| E7EM72 | Diacylglycerol kinase OS=Homo sapiens GN=DGKI PE=1 SV=1 | 114978 | 7.6699 | 0.192 | B | Y |
| E7EMB3 | Calmodulin OS=Homo sapiens GN=CALM2 PE=1 SV=1 | 21675 | 4.2524 | 0.001 | B | Y |
| E7EMM4 | Acid ceramidase OS=Homo sapiens GN=ASAH1 PE=1 SV=1 | 41769 | 8.1343 | 0.077 | B | Y |
| E7EMV2 | Neurofilament medium polypeptide OS=Homo sapiens GN=NEFM PE=1 SV=1 | 78834 | 4.5688 | 0.315 | B | Y |
| E7ENQ6 | Uncharacterized protein OS=Homo sapiens PE=4 SV=1 | 30075 | 6.5039 | 0.580 | B | Y |
| E7ENY0 | Alpha-adducin OS=Homo sapiens GN=ADD1 PE=1 SV=1 | 73358 | 6.0938 | 0.001 | B | Y |
| E7EPB3 | 60S ribosomal protein L14 OS=Homo sapiens GN=RPL14 PE=1 SV=1 | 14548 | 10.6597 | 0.363 | B | Y |
| E7EPI0 | Inhibitor of Bruton tyrosine kinase OS=Homo sapiens GN=IBTK PE=1 SV=1 | 148921 | 7.4077 | 0.142 | B | Y |
| E7EPK1 | Septin-7 OS=Homo sapiens GN=SEPT7 PE=1 SV=2 | 50662 | 8.8887 | 0.001 | B | Y |
| E7EPT4 | NADH dehydrogenase [ubiquinone] flavoprotein 2, mitochondrial OS=Homo sapiens GN=NDUF | 27889 | 7.9966 | 0.001 | B | Y |
| E7EPV7 | Alpha-synuclein OS=Homo sapiens GN=SNCA PE=1 SV=1 | 11769 | 9.7354 | 1.229 | B | Y |
| E7EQB8 | Isocitrate dehydrogenase [NAD] subunit, mitochondrial OS=Homo sapiens GN=IDH3G PE=1 S\ | 37043 | 8.7671 | 0.001 | B | Y |
| E7EQR4 | Ezrin OS=Homo sapiens GN=EZR PE=1 SV=3 | 69328 | 5.7876 | 0.001 | B | Y |
| E7EQV9 | Ribosomal protein L15 (Fragment) OS=Homo sapiens GN=RPL15 PE=1 SV=1 | 20497 | 11.6777 | 0.001 | B | Y |
| E7ES68 | Zinc phosphodiesterase ELAC protein 2 (Fragment) OS=Homo sapiens GN=ELAC2 PE=1 SV=2 | 70173 | 6.5464 | 0.054 | B | Y |
| E7ESP9 | Neurofilament medium polypeptide OS=Homo sapiens GN=NEFM PE=1 SV=1 | 98322 | 4.6523 | 0.555 | B | Y |
| E7EVA0 | Microtubule-associated protein OS=Homo sapiens GN=MAP4 PE=1 SV=1 | 245289 | 5.833 | 0.114 | B | Y |
| E7EWE5 | Peroxisomal multifunctional enzyme type 2 OS=Homo sapiens GN=HSD17B4 PE=1 SV=1 | 77538 | 8.9092 | 0.133 | B | Y |
| E9PB61 | THO complex subunit 4 OS=Homo sapiens GN=ALYREF PE=1 SV=1 | 27540 | 11.4551 | 0.001 | B | Y |
| E9PCP0 | Guanine nucleotide-binding protein G(I)/G(S)/G(T) subunit beta-3 OS=Homo sapiens GN=GNB3 | 37093 | 5.2676 | 0.199 | B | Y |
| E9PD68 | Dihydropyrimidinase-related protein 1 OS=Homo sapiens GN=CRMP1 PE=1 SV=1 | 61990 | 6.2578 | 0.298 | B | Y |
| E9PDE8 | Heat shock 70 kDa protein 4L OS=Homo sapiens GN=HSPA4L PE=1 SV=1 | 91895 | 5.6924 | 0.160 | B | Y |
| E9PDG8 | Clathrin coat assembly protein AP180 OS=Homo sapiens GN=SNAP91 PE=1 SV=1 | 91913 | 4.522 | 0.581 | B | Y |
| E9PDL2 | Dipeptidyl aminopeptidase-like protein 6 OS=Homo sapiens GN=DPP6 PE=1 SV=1 | 86272 | 5.8081 | 0.137 | B | Y |
| E9PEX6 | Dihydrolipoyl dehydrogenase OS=Homo sapiens GN=DLD PE=1 SV=1 | 51782 | 7.9233 | 0.273 | B | Y |
| E9PF17 | Versican core protein OS=Homo sapiens GN=VCAN PE=1 SV=2 | 176718 | 4.5176 | 1.465 | B | Y |
| E9PF46 | Acylphosphatase OS=Homo sapiens GN=ACYP2 PE=1 SV=1 | 10291 | 9.0117 | 0.001 | B | Y |
| E9PF58 | Actin-related protein 2/3 complex subunit 1A OS=Homo sapiens GN=ARPC1A PE=1 SV=1 | 30868 | 8.8184 | 0.133 | B | Y |
| E9PHY5 | Band 4.1-like protein 2 OS=Homo sapiens GN=EPB41L2 PE=1 SV=1 | 104296 | 5.1284 | 0.084 | B | Y |
| E9PIM6 | Thy-1 membrane glycoprotein (Fragment) OS=Homo sapiens GN=THY1 PE=1 SV=6 | 16916 | 9.2461 | 1.583 | B | Y |
| E9PJH7 | Mitochondrial glutamate carrier 1 (Fragment) OS=Homo sapiens GN=SLC25A22 PE=1 SV=5 | 33260 | 10.0063 | 0.972 | B | Y |
| E9PK25 | Cofilin-1 OS=Homo sapiens GN=CFL1 PE=1 SV=1 | 22713 | 8.5181 | 0.001 | B | Y |
| E9PKD5 | 26S protease regulatory subunit 6A (Fragment) OS=Homo sapiens GN=PSMC3 PE=1 SV=2 | 34592 | 5.0332 | 0.050 | B | Y |
| E9PKE3 | Heat shock cognate 71 kDa protein OS=Homo sapiens GN=HSPA8 PE=1 SV=1 | 68763 | 5.1973 | 1.628 | B | Y |
| E9PKG1 | Protein arginine N-methyltransferase 1 OS=Homo sapiens GN=PRMT1 PE=1 SV=1 | 37684 | 5.71 | 0.357 | B | Y |
| E9PKH6 | NADH dehydrogenase [ubiquinone] iron-sulfur protein 8, mitochondrial (Fragment) OS=Homo sa | 15881 | 7.5044 | 0.043 | B | Y |
| E9PKU7 | Neutral alpha-glucosidase AB OS=Homo sapiens GN=GANAB PE=1 SV=1 | 96483 | 5.5093 | 0.001 | B | Y |
| E9PL57 | Protein NEDD8-MDP1 (Fragment) OS=Homo sapiens GN=NEDD8-MDP1 PE=4 SV=1 | 19524 | 7.4004 | 0.001 | B | Y |
| E9PLK3 | Puromycin-sensitive aminopeptidase OS=Homo sapiens GN=NPEPPS PE=1 SV=1 | 102922 | 5.2661 | 0.322 | B | Y |
| E9PMV1 | Plectin (Fragment) OS=Homo sapiens GN=PLEC PE=1 SV=1 | 80730 | 6.3018 | 0.375 | B | Y |

| | | | | | | |
|---|---|---|---|---|---|---|
| E9PN17 | ATP synthase subunit g, mitochondrial OS=Homo sapiens GN=ATP5L PE=1 SV=1 | 8446 | 10.7021 | 0.934 | B | Y |
| E9PNW4 | CD59 glycoprotein OS=Homo sapiens GN=CD59 PE=1 SV=1 | 11976 | 5.3672 | 0.298 | B | Y |
| E9PP67 | Toll-interacting protein OS=Homo sapiens GN=TOLLIP PE=1 SV=1 | 20260 | 8.8608 | 0.968 | B | Y |
| E9PPQ4 | Ferritin (Fragment) OS=Homo sapiens GN=FTH1 PE=1 SV=1 | 6659 | 5.4185 | 0.546 | B | Y |
| E9PPV6 | Serpin H1 OS=Homo sapiens GN=SERPINH1 PE=1 SV=1 | 35643 | 9.4629 | 0.331 | B | Y |
| E9PQ59 | Nuclear factor-related to kappa-B-binding protein (Fragment) OS=Homo sapiens GN=NFRKB P | 108173 | 8.3804 | 1.048 | B | Y |
| E9PR44 | Alpha-crystallin B chain (Fragment) OS=Homo sapiens GN=CRYAB PE=1 SV=1 | 20018 | 6.5698 | 1.877 | B | Y |
| E9PRY8 | Elongation factor 1-delta OS=Homo sapiens GN=EEF1D PE=1 SV=1 | 76522 | 6.6196 | 0.104 | B | Y |
| F1T0E5 | Calcium-dependent secretion activator 1 OS=Homo sapiens GN=CADPS PE=1 SV=1 | 151904 | 5.5342 | 0.056 | B | Y |
| F2Z393 | Transaldolase OS=Homo sapiens GN=TALDO1 PE=1 SV=1 | 35306 | 9.2988 | 0.168 | B | Y |
| F5GX30 | Cation-dependent mannose-6-phosphate receptor OS=Homo sapiens GN=M6PR PE=1 SV=2 | 21524 | 6.2358 | 0.001 | B | Y |
| F5GY55 | DNA damage-binding protein 1 OS=Homo sapiens GN=DDB1 PE=1 SV=1 | 121636 | 5.1914 | 0.338 | B | Y |
| F5GYN4 | Ubiquitin thioesterase OTUB1 OS=Homo sapiens GN=OTUB1 PE=1 SV=1 | 28032 | 4.9849 | 0.312 | B | Y |
| F5GYQ1 | V-type proton ATPase subunit d 1 OS=Homo sapiens GN=ATP6V0D1 PE=1 SV=1 | 44631 | 4.8457 | 0.001 | B | Y |
| F5GZS6 | 4F2 cell-surface antigen heavy chain OS=Homo sapiens GN=SLC3A2 PE=1 SV=1 | 64832 | 4.7813 | 0.216 | B | Y |
| F5H006 | Lymphoid-restricted membrane protein OS=Homo sapiens GN=LRMP PE=1 SV=1 | 50393 | 5.8594 | 9.077 | B | Y |
| F5H018 | GTP-binding nuclear protein Ran (Fragment) OS=Homo sapiens GN=RAN PE=1 SV=6 | 22421 | 9.0278 | 0.365 | B | Y |
| F5H0B0 | Uncharacterized protein OS=Homo sapiens PE=4 SV=2 | 27450 | 4.9629 | 0.001 | B | Y |
| F5H1U9 | Multiple PDZ domain protein OS=Homo sapiens GN=MPDZ PE=1 SV=1 | 222986 | 4.7622 | 0.001 | B | Y |
| F5H2F4 | C-1-tetrahydrofolate synthase, cytoplasmic OS=Homo sapiens GN=MTHFD1 PE=1 SV=1 | 110543 | 8.4565 | 0.001 | B | Y |
| F5H481 | Protein N-lysine methyltransferase METTL20 (Fragment) OS=Homo sapiens GN=METTL20 PE | 7875 | 7.0781 | 1.319 | B | Y |
| F5H4R6 | Nucleosome assembly protein 1-like 1 OS=Homo sapiens GN=NAP1L1 PE=1 SV=1 | 44598 | 4.2729 | 0.001 | B | Y |
| F5H5G1 | Limbic system-associated membrane protein OS=Homo sapiens GN=LSAMP PE=1 SV=2 | 31722 | 5.5635 | 0.337 | B | Y |
| F6RFD5 | Destrin OS=Homo sapiens GN=DSTN PE=1 SV=1 | 15386 | 8.748 | 0.560 | B | Y |
| F6TLX2 | Glyoxalase domain-containing protein 4 OS=Homo sapiens GN=GLOD4 PE=1 SV=1 | 54684 | 8.7451 | 0.001 | B | Y |
| F6U236 | Protein kinase C and casein kinase substrate in neurons protein 1 OS=Homo sapiens GN=PAC | 46117 | 5.0786 | 0.180 | B | Y |
| F8VPD4 | CAD protein OS=Homo sapiens GN=CAD PE=1 SV=1 | 235870 | 6.0835 | 0.390 | B | Y |
| F8VSD4 | Ubiquitin-conjugating enzyme E2 N OS=Homo sapiens GN=UBE2N PE=1 SV=1 | 11975 | 9.5171 | 0.181 | B | Y |
| F8VVM2 | Phosphate carrier protein, mitochondrial OS=Homo sapiens GN=SLC25A3 PE=1 SV=1 | 36137 | 9.4966 | 0.676 | B | Y |
| F8VYN9 | ADP-ribosylation factor-like protein 1 OS=Homo sapiens GN=ARL1 PE=1 SV=1 | 21764 | 6.3545 | 0.001 | B | Y |
| F8VZX2 | Poly(rC)-binding protein 2 OS=Homo sapiens GN=PCBP2 PE=1 SV=1 | 33778 | 8.2822 | 1.160 | B | Y |
| F8VZY9 | Keratin, type I cytoskeletal 18 OS=Homo sapiens GN=KRT18 PE=1 SV=1 | 43747 | 5.0815 | 0.246 | B | Y |
| F8W1D1 | Melanocyte protein PMEL (Fragment) OS=Homo sapiens GN=PMEL PE=1 SV=1 | 32282 | 7.2686 | 0.503 | B | Y |
| F8W6I7 | Heterogeneous nuclear ribonucleoprotein A1 OS=Homo sapiens GN=HNRNPA1 PE=1 SV=2 | 33134 | 9.4336 | 1.087 | B | Y |
| F8W726 | Ubiquitin-associated protein 2-like OS=Homo sapiens GN=UBAP2L PE=1 SV=2 | 113558 | 6.2051 | 0.076 | B | Y |
| F8W8T1 | Interferon-induced GTP-binding protein Mx1 OS=Homo sapiens GN=MX1 PE=1 SV=1 | 73290 | 5.4785 | 0.250 | B | Y |
| F8W914 | Reticulon OS=Homo sapiens GN=RTN4 PE=1 SV=1 | 37121 | 4.4941 | 0.164 | B | Y |
| F8W9U4 | Microtubule-associated protein OS=Homo sapiens GN=MAP4 PE=1 SV=1 | 88222 | 9.6035 | 0.094 | B | Y |
| F8WCA0 | Vesicle-associated membrane protein 2 OS=Homo sapiens GN=VAMP2 PE=4 SV=1 | 12925 | 8.7012 | 0.001 | B | Y |
| F8WCF6 | Protein ARPC4-TTLL3 OS=Homo sapiens GN=ARPC4-TTLL3 PE=4 SV=1 | 21045 | 9.186 | 0.001 | B | Y |
| F8WE04 | Heat shock protein beta-1 OS=Homo sapiens GN=HSPB1 PE=1 SV=1 | 20393 | 9.394 | 1.702 | B | Y |
| F8WEJ5 | Asparagine synthetase [glutamine-hydrolyzing] OS=Homo sapiens GN=ASNS PE=1 SV=1 | 45597 | 6.6899 | 0.319 | B | Y |
| G3V0I5 | NADH dehydrogenase (Ubiquinone) flavoprotein 1, 51kDa, isoform CRA_c OS=Homo sapiens ( | 50022 | 8.1533 | 0.187 | B | Y |
| G3V1B3 | 60S ribosomal protein L21 OS=Homo sapiens GN=RPL21 PE=1 SV=1 | 9880 | 10.6567 | 0.234 | B | Y |
| G3V1N2 | HCG1745306, isoform CRA_a OS=Homo sapiens GN=HBA2 PE=1 SV=1 | 11940 | 9.3018 | 2.342 | B | Y |
| G3V200 | Liprin-alpha-2 OS=Homo sapiens GN=PPFIA2 PE=1 SV=2 | 142019 | 5.625 | 0.041 | B | Y |
| G3V2N0 | Guanine nucleotide-binding protein subunit gamma OS=Homo sapiens GN=GNG2 PE=1 SV=1 | 12378 | 9.3047 | 0.001 | B | Y |
| G3V2R9 | Prostaglandin reductase 2 OS=Homo sapiens GN=PTGR2 PE=1 SV=1 | 23364 | 5.7773 | 0.488 | B | Y |
| G3V2S6 | V-type proton ATPase subunit D OS=Homo sapiens GN=ATP6V1D PE=1 SV=1 | 21907 | 5.48 | 0.185 | B | Y |
| G3V2U4 | Protein unc-79 homolog OS=Homo sapiens GN=UNC79 PE=4 SV=1 | 290594 | 5.8228 | 0.050 | B | Y |
| G3V359 | DNA-(apurinic or apyrimidinic site) lyase (Fragment) OS=Homo sapiens GN=APEX1 PE=1 SV= | 19155 | 5.1855 | 0.001 | B | Y |
| G3V4P8 | Glia maturation factor beta (Fragment) OS=Homo sapiens GN=GMFB PE=1 SV=1 | 17500 | 5.0332 | 0.001 | B | Y |
| G3V5Z7 | Proteasome subunit alpha type OS=Homo sapiens GN=PSMA6 PE=1 SV=1 | 28129 | 6.3677 | 0.001 | B | Y |
| G3XAL0 | Malate dehydrogenase OS=Homo sapiens GN=MDH2 PE=1 SV=1 | 24578 | 8.0024 | 0.808 | B | Y |
| G5E9Q6 | Profilin OS=Homo sapiens GN=PFN2 PE=1 SV=1 | 20773 | 7.4106 | 0.095 | B | Y |
| G8JLB6 | Heterogeneous nuclear ribonucleoprotein H OS=Homo sapiens GN=HNRNPH1 PE=1 SV=1 | 51197 | 6.3442 | 1.208 | B | Y |
| G8JLD5 | Dynamin-1-like protein OS=Homo sapiens GN=DNM1L PE=1 SV=1 | 79572 | 6.6519 | 0.159 | B | Y |
| H0Y2P0 | CD44 antigen (Fragment) OS=Homo sapiens GN=CD44 PE=1 SV=1 | 30932 | 5.0156 | 0.001 | B | Y |
| H0Y3Y4 | Septin-7 (Fragment) OS=Homo sapiens GN=SEPT7 PE=1 SV=1 | 43009 | 7.7446 | 0.576 | B | Y |
| H0Y474 | V-type proton ATPase subunit G 2 (Fragment) OS=Homo sapiens GN=ATP6V1G2 PE=1 SV=1 | 16884 | 11.1489 | 0.001 | B | Y |
| H0Y512 | Adipocyte plasma membrane-associated protein (Fragment) OS=Homo sapiens GN=APMAP PI | 45377 | 5.2998 | 0.001 | B | Y |
| H0Y6E7 | RNA-binding motif protein, X chromosome (Fragment) OS=Homo sapiens GN=RBMX PE=1 SV | 31837 | 10.0635 | 0.166 | B | Y |
| H0Y7V4 | Dynein heavy chain 8, axonemal OS=Homo sapiens GN=DNAH8 PE=1 SV=1 | 478565 | 5.7114 | 10.758 | B | Y |
| H0Y8E6 | DNA replication licensing factor MCM2 (Fragment) OS=Homo sapiens GN=MCM2 PE=1 SV=1 | 94050 | 5.8008 | 0.440 | B | Y |
| H0Y8G5 | Heterogeneous nuclear ribonucleoprotein D0 (Fragment) OS=Homo sapiens GN=HNRNPD PE | 29648 | 9.4526 | 0.001 | B | Y |
| H0Y8L3 | Transforming growth factor-beta-induced protein ig-h3 (Fragment) OS=Homo sapiens GN=TGFI | 39880 | 6.4028 | 0.284 | B | Y |
| H0Y8X1 | Succinate dehydrogenase [ubiquinone] flavoprotein subunit, mitochondrial (Fragment) OS=Hom | 13628 | 10.5 | 3.846 | B | Y |
| H0YCG2 | Lysosome-associated membrane glycoprotein 2 (Fragment) OS=Homo sapiens GN=LAMP2 PE | 28247 | 6.0718 | 0.001 | B | Y |
| H0YCY8 | Dipeptidyl peptidase 1 (Fragment) OS=Homo sapiens GN=CTSC PE=1 SV=6 | 27819 | 9.4131 | 0.374 | B | Y |
| H0YDD4 | Acetyltransferase component of pyruvate dehydrogenase complex (Fragment) OS=Homo sapiei | 51169 | 8.6733 | 0.304 | B | Y |
| H0YDN1 | Plectin (Fragment) OS=Homo sapiens GN=PLEC PE=1 SV=6 | 23731 | 4.6934 | 0.241 | B | Y |
| H0YJ21 | Cytoplasmic dynein 1 heavy chain 1 (Fragment) OS=Homo sapiens GN=DYNC1H1 PE=1 SV=1 | 21009 | 7.0942 | 0.141 | B | Y |
| H0YKT8 | Proteasome subunit beta type (Fragment) OS=Homo sapiens GN=PSMA4 PE=1 SV=6 | 19949 | 6.3398 | 0.091 | B | Y |
| H0YLA2 | Signal recognition particle 14 kDa protein OS=Homo sapiens GN=SRP14 PE=1 SV=1 | 13049 | 9.9697 | 0.181 | B | Y |
| H0YLN8 | Transient receptor potential cation channel subfamily M member 7 OS=Homo sapiens GN=TRP | 212488 | 7.7344 | 0.084 | B | Y |
| H0YN26 | Acidic leucine-rich nuclear phosphoprotein 32 family member A OS=Homo sapiens GN=ANP32/ | 19985 | 4.27 | 0.092 | B | Y |
| H0YNE9 | Ras-related protein Rab-8B (Fragment) OS=Homo sapiens GN=RAB8B PE=1 SV=1 | 21854 | 8.7349 | 0.029 | B | Y |
| H3BLZ8 | Probable ATP-dependent RNA helicase DDX17 OS=Homo sapiens GN=DDX17 PE=1 SV=1 | 80389 | 8.3511 | 0.001 | B | Y |
| H3BMH2 | Ras-related protein Rab-11A (Fragment) OS=Homo sapiens GN=RAB11A PE=3 SV=1 | 17657 | 8.9253 | 0.206 | B | Y |
| H3BNQ7 | 4-aminobutyrate aminotransferase, mitochondrial OS=Homo sapiens GN=ABAT PE=1 SV=1 | 57886 | 7.749 | 0.001 | B | Y |
| H3BNX8 | Cytochrome c oxidase subunit 5A, mitochondrial OS=Homo sapiens GN=COX5A PE=1 SV=1 | 17223 | 5.603 | 0.001 | B | Y |

| | | | | | | |
|---|---|---|---|---|---|---|
| H3BPE7 | RNA-binding protein FUS OS=Homo sapiens GN=FUS PE=1 SV=1 | 53464 | 9.4951 | 0.001 | B | Y |
| H3BPJ9 | NADH dehydrogenase [ubiquinone] 1 beta subcomplex subunit 10 OS=Homo sapiens GN=NDU | 19245 | 8.8608 | 0.001 | B | Y |
| H3BPQ4 | Hydroxyacylglutathione hydrolase, mitochondrial OS=Homo sapiens GN=HAGH PE=1 SV=1 | 27895 | 6.9053 | 0.001 | B | Y |
| H3BPS8 | Fructose-bisphosphate aldolase (Fragment) OS=Homo sapiens GN=ALDOA PE=1 SV=1 | 30407 | 7.2949 | 0.156 | B | Y |
| H3BQ23 | Anamorsin (Fragment) OS=Homo sapiens GN=CIAPIN1 PE=1 SV=1 | 13624 | 5.5737 | 0.001 | B | Y |
| H3BQN4 | Fructose-bisphosphate aldolase OS=Homo sapiens GN=ALDOA PE=1 SV=1 | 39315 | 8.4331 | 0.596 | B | Y |
| H3BR70 | Pyruvate kinase OS=Homo sapiens GN=PKM PE=1 SV=1 | 40163 | 8.0171 | 2.204 | B | Y |
| H3BT82 | Syntaxin-1B (Fragment) OS=Homo sapiens GN=STX1B PE=1 SV=2 | 23432 | 7.2378 | 0.332 | B | Y |
| H3BTN5 | Pyruvate kinase (Fragment) OS=Homo sapiens GN=PKM PE=1 SV=1 | 53011 | 6.3926 | 0.204 | B | Y |
| H7BXZ6 | Mitochondrial Rho GTPase OS=Homo sapiens GN=RHOT1 PE=1 SV=2 | 68405 | 5.5869 | 0.065 | B | Y |
| H7BYH4 | Superoxide dismutase [Cu-Zn] OS=Homo sapiens GN=SOD1 PE=1 SV=1 | 13900 | 5.6558 | 0.001 | B | Y |
| H7C1V0 | Cathepsin D (Fragment) OS=Homo sapiens GN=CTSD PE=1 SV=1 | 20358 | 8.5957 | 0.518 | B | Y |
| H7C3T4 | Peroxiredoxin-4 (Fragment) OS=Homo sapiens GN=PRDX4 PE=1 SV=1 | 18314 | 5.7598 | 1.640 | B | Y |
| H9KV31 | Neural cell adhesion molecule 2 OS=Homo sapiens GN=NCAM2 PE=1 SV=2 | 91066 | 5.2939 | 0.306 | B | Y |
| I3L0A0 | HCG2044781 OS=Homo sapiens GN=TMEM189-UBE2V1 PE=4 SV=1 | 42181 | 6.249 | 0.001 | B | Y |
| I3L0N3 | Vesicle-fusing ATPase OS=Homo sapiens GN=NSF PE=1 SV=1 | 82039 | 6.3735 | 0.722 | B | Y |
| I3L1N3 | Phosphorylase b kinase regulatory subunit beta (Fragment) OS=Homo sapiens GN=PHKB PE= | 9139 | 4.3813 | 1.358 | B | Y |
| I3L397 | Eukaryotic translation initiation factor 5A (Fragment) OS=Homo sapiens GN=EIF5A PE=1 SV=6 | 16008 | 4.6567 | 0.410 | B | Y |
| I6L894 | Ankyrin-2 OS=Homo sapiens GN=ANK2 PE=1 SV=1 | 429989 | 4.8413 | 8.233 | B | Y |
| J3KMX5 | 40S ribosomal protein S13 OS=Homo sapiens GN=RPS13 PE=1 SV=1 | 16722 | 10.9395 | 0.059 | B | Y |
| J3KN67 | Tropomyosin alpha-3 chain OS=Homo sapiens GN=TPM3 PE=1 SV=1 | 33201 | 4.5293 | 0.222 | B | Y |
| J3KN75 | TBC1 domain family member 8B OS=Homo sapiens GN=TBC1D8B PE=1 SV=1 | 128026 | 5.6074 | 0.204 | B | Y |
| J3KP15 | Serine/arginine-rich-splicing factor 2 (Fragment) OS=Homo sapiens GN=SRSF2 PE=1 SV=6 | 15361 | 11.2954 | 0.001 | B | Y |
| J3KPX7 | Prohibitin-2 OS=Homo sapiens GN=PHB2 PE=1 SV=2 | 33218 | 10.1895 | 0.464 | B | Y |
| J3KQ32 | Obg-like ATPase 1 OS=Homo sapiens GN=OLA1 PE=1 SV=1 | 46908 | 8.1753 | 0.001 | B | Y |
| J3KQA0 | Synaptotagmin I, isoform CRA_b OS=Homo sapiens GN=SYT1 PE=1 SV=1 | 47230 | 7.9702 | 0.851 | B | Y |
| J3KRE2 | Rho GDP-dissociation inhibitor 1 OS=Homo sapiens GN=ARHGDIA PE=1 SV=1 | 14805 | 4.3799 | 0.283 | B | Y |
| J3KSD8 | Bleomycin hydrolase (Fragment) OS=Homo sapiens GN=BLMH PE=1 SV=6 | 30244 | 7.2393 | 0.001 | B | Y |
| J3KT73 | 60S ribosomal protein L38 OS=Homo sapiens GN=RPL38 PE=1 SV=1 | 7560 | 10.4531 | 0.112 | B | Y |
| J3KTL2 | Serine/arginine-rich-splicing factor 1 OS=Homo sapiens GN=SRSF1 PE=1 SV=1 | 28311 | 10.2861 | 0.001 | B | Y |
| J3QRS3 | Myosin regulatory light chain 12A OS=Homo sapiens GN=MYL12A PE=1 SV=1 | 20443 | 4.4048 | 0.001 | B | Y |
| J3QT27 | Poly(rC)-binding protein 3 (Fragment) OS=Homo sapiens GN=PCBP3 PE=1 SV=1 | 34363 | 7.7461 | 0.030 | B | Y |
| J3QT28 | Mitotic checkpoint protein BUB3 (Fragment) OS=Homo sapiens GN=BUB3 PE=1 SV=1 | 31683 | 8.1548 | 0.079 | B | Y |
| J9JID7 | Lamin B2, isoform CRA_a OS=Homo sapiens GN=LMNB2 PE=1 SV=1 | 69905 | 5.3364 | 0.001 | B | Y |
| K7EK07 | Histone H3 (Fragment) OS=Homo sapiens GN=H3F3B PE=1 SV=1 | 14905 | 11.7026 | 1.034 | B | Y |
| K7EKH9 | Glial fibrillary acidic protein (Fragment) OS=Homo sapiens GN=GFAP PE=1 SV=1 | 29791 | 8.9473 | 44.637 | B | Y |
| K7EKS7 | 60S ribosomal protein L22 OS=Homo sapiens GN=RPL22 PE=1 SV=1 | 5730 | 10.6831 | 0.001 | B | Y |
| K7ELG9 | Protein LSM12 homolog OS=Homo sapiens GN=LSM12 PE=1 SV=1 | 24939 | 7.1689 | 0.001 | B | Y |
| K7ELL7 | Glucosidase 2 subunit beta OS=Homo sapiens GN=PRKCSH PE=1 SV=1 | 60154 | 4.1396 | 0.001 | B | Y |
| K7EN45 | Peptidyl-prolyl cis-trans isomerase NIMA-interacting 1 (Fragment) OS=Homo sapiens GN=PIN1 | 9938 | 4.7373 | 0.304 | B | Y |
| K7EP04 | Heat shock protein beta-6 OS=Homo sapiens GN=HSPB6 PE=1 SV=1 | 14654 | 9.3589 | 0.051 | B | Y |
| K7ER00 | Phenylalanine--tRNA ligase alpha subunit OS=Homo sapiens GN=FARSA PE=1 SV=1 | 62356 | 7.7534 | 0.001 | B | Y |
| K7ERE3 | Keratin, type I cytoskeletal 13 OS=Homo sapiens GN=KRT13 PE=1 SV=1 | 45232 | 4.5615 | 0.731 | B | Y |
| K7N7A8 | Uncharacterized protein (Fragment) OS=Homo sapiens PE=3 SV=2 | 48766 | 4.8501 | 0.001 | B | Y |
| M0QXL5 | rRNA 2'-O-methyltransferase fibrillarin (Fragment) OS=Homo sapiens GN=FBL PE=1 SV=1 | 26622 | 9.3018 | 0.001 | B | Y |
| M0QXS5 | Heterogeneous nuclear ribonucleoprotein L (Fragment) OS=Homo sapiens GN=HNRNPL PE=1 | 58436 | 6.3208 | 0.362 | B | Y |
| M0QYT0 | Uncharacterized protein (Fragment) OS=Homo sapiens PE=1 SV=1 | 35992 | 7.1689 | 0.012 | B | Y |
| M0QYZ2 | AP-2 complex subunit sigma OS=Homo sapiens GN=AP2S1 PE=1 SV=1 | 18916 | 7.834 | 0.001 | B | Y |
| M0QZL7 | Tubulin beta-4A chain (Fragment) OS=Homo sapiens GN=TUBB4A PE=1 SV=6 | 16558 | 5.7173 | 7.721 | B | Y |
| M0R0F0 | 40S ribosomal protein S5 (Fragment) OS=Homo sapiens GN=RPS5 PE=1 SV=1 | 22376 | 9.9214 | 0.244 | B | Y |
| M0R0P7 | 60S ribosomal protein L18a OS=Homo sapiens GN=RPL18A PE=1 SV=1 | 16166 | 11.064 | 0.049 | B | Y |
| M0R116 | Sodium/potassium-transporting ATPase subunit alpha OS=Homo sapiens GN=ATP1A3 PE=1 S | 108209 | 4.9629 | 0.001 | B | Y |
| M0R3B8 | NADH dehydrogenase [ubiquinone] 1 beta subcomplex subunit 7 OS=Homo sapiens GN=NDUF | 7423 | 5.3188 | 0.106 | B | Y |
| O00154 | Cytosolic acyl coenzyme A thioester hydrolase OS=Homo sapiens GN=ACOT7 PE=1 SV=3 | 41769 | 8.5444 | 0.486 | B | Y |
| O00231 | 26S proteasome non-ATPase regulatory subunit 11 OS=Homo sapiens GN=PSMD11 PE=1 SV= | 47434 | 6.0469 | 0.117 | B | Y |
| O00264 | Membrane-associated progesterone receptor component 1 OS=Homo sapiens GN=PGRMC1 P | 21657 | 4.3433 | 0.259 | B | Y |
| O00339 | Matrilin-2 OS=Homo sapiens GN=MATN2 PE=1 SV=4 | 106766 | 5.8257 | 0.520 | B | Y |
| O00468 | Agrin OS=Homo sapiens GN=AGRN PE=1 SV=5 | 217089 | 5.9575 | 0.132 | B | Y |
| O00483 | Cytochrome c oxidase subunit NDUFA4 OS=Homo sapiens GN=NDUFA4 PE=1 SV=1 | 9363 | 9.75 | 0.546 | B | Y |
| O00764 | Pyridoxal kinase OS=Homo sapiens GN=PDXK PE=1 SV=1 | 35079 | 5.6953 | 0.154 | B | Y |
| O14531 | Dihydropyrimidinase-related protein 4 OS=Homo sapiens GN=DPYSL4 PE=1 SV=2 | 61838 | 6.6592 | 0.110 | B | Y |
| O14602 | Eukaryotic translation initiation factor 1A, Y-chromosomal OS=Homo sapiens GN=EIF1AY PE=1 | 16432 | 4.8794 | 0.001 | B | Y |
| O14745 | Na(+)/H(+) exchange regulatory cofactor NHE-RF1 OS=Homo sapiens GN=SLC9A3R1 PE=1 S | 38844 | 5.4331 | 0.220 | B | Y |
| O14880 | Microsomal glutathione S-transferase 3 OS=Homo sapiens GN=MGST3 PE=1 SV=1 | 16505 | 9.6021 | 0.187 | B | Y |
| O14910 | Protein lin-7 homolog A OS=Homo sapiens GN=LIN7A PE=1 SV=2 | 25980 | 9.1201 | 0.147 | B | Y |
| O14936 | Peripheral plasma membrane protein CASK OS=Homo sapiens GN=CASK PE=1 SV=3 | 105056 | 5.9634 | 0.357 | B | Y |
| O14949 | Cytochrome b-c1 complex subunit 8 OS=Homo sapiens GN=UQCRQ PE=1 SV=4 | 9900 | 10.3462 | 0.335 | B | Y |
| O15020 | Spectrin beta chain, non-erythrocytic 2 OS=Homo sapiens GN=SPTBN2 PE=1 SV=3 | 271155 | 5.7144 | 0.530 | B | Y |
| O15075 | Serine/threonine-protein kinase DCLK1 OS=Homo sapiens GN=DCLK1 PE=1 SV=2 | 82172 | 8.7935 | 0.168 | B | Y |
| O15144 | Actin-related protein 2/3 complex subunit 2 OS=Homo sapiens GN=ARPC2 PE=1 SV=1 | 34311 | 6.9785 | 0.319 | B | Y |
| O15145 | Actin-related protein 2/3 complex subunit 3 OS=Homo sapiens GN=ARPC3 PE=1 SV=3 | 20533 | 8.8037 | 0.299 | B | Y |
| O15498 | Synaptobrevin homolog YKT6 OS=Homo sapiens GN=YKT6 PE=1 SV=1 | 22403 | 6.5098 | 0.121 | B | Y |
| O15540 | Fatty acid-binding protein, brain OS=Homo sapiens GN=FABP7 PE=1 SV=3 | 14879 | 5.25 | 0.498 | B | Y |
| O43175 | D-3-phosphoglycerate dehydrogenase OS=Homo sapiens GN=PHGDH PE=1 SV=4 | 56614 | 6.2798 | 0.001 | B | Y |
| O43236 | Septin-4 OS=Homo sapiens GN=SEPT4 PE=1 SV=1 | 55063 | 5.6938 | 0.064 | B | Y |
| O43242 | 26S proteasome non-ATPase regulatory subunit 3 OS=Homo sapiens GN=PSMD3 PE=1 SV=2 | 60939 | 8.6953 | 0.086 | B | Y |
| O43301 | Heat shock 70 kDa protein 12A OS=Homo sapiens GN=HSPA12A PE=1 SV=2 | 74931 | 6.3179 | 0.149 | B | Y |
| O43390 | Heterogeneous nuclear ribonucleoprotein R OS=Homo sapiens GN=HNRNPR PE=1 SV=1 | 70899 | 8.2119 | 0.354 | B | Y |
| O43396 | Thioredoxin-like protein 1 OS=Homo sapiens GN=TXNL1 PE=1 SV=3 | 32230 | 4.6436 | 0.063 | B | Y |
| O43488 | Aflatoxin B1 aldehyde reductase member 2 OS=Homo sapiens GN=AKR7A2 PE=1 SV=3 | 39563 | 6.7485 | 0.257 | B | Y |
| O43707 | Alpha-actinin-4 OS=Homo sapiens GN=ACTN4 PE=1 SV=2 | 104788 | 5.1211 | 0.305 | B | Y |

| | | | | | | |
|---|---|---|---|---|---|---|
| O43865 | Adenosylhomocysteinase 2 OS=Homo sapiens GN=AHCYL1 PE=1 SV=2 | 58913 | 6.4775 | 0.450 | B | Y |
| O60293 | Zinc finger C3H1 domain-containing protein OS=Homo sapiens GN=ZFC3H1 PE=1 SV=3 | 226212 | 8.0698 | 0.741 | B | Y |
| O60506 | Heterogeneous nuclear ribonucleoprotein Q OS=Homo sapiens GN=SYNCRIP PE=1 SV=2 | 69559 | 8.7861 | 0.252 | B | Y |
| O60551 | Glycylpeptide N-tetradecanoyltransferase 2 OS=Homo sapiens GN=NMT2 PE=1 SV=1 | 56944 | 7.3491 | 0.001 | B | Y |
| O60830 | Mitochondrial import inner membrane translocase subunit Tim17-B OS=Homo sapiens GN=TIM | 18261 | 9.2944 | 0.001 | B | Y |
| O60884 | DnaJ homolog subfamily A member 2 OS=Homo sapiens GN=DNAJA2 PE=1 SV=1 | 45716 | 6.0352 | 0.096 | B | Y |
| O75061 | Putative tyrosine-protein phosphatase auxilin OS=Homo sapiens GN=DNAJC6 PE=1 SV=3 | 99933 | 6.8408 | 0.123 | B | Y |
| O75083 | WD repeat-containing protein 1 OS=Homo sapiens GN=WDR1 PE=1 SV=4 | 66151 | 6.1772 | 0.321 | B | Y |
| O75131 | Copine-3 OS=Homo sapiens GN=CPNE3 PE=1 SV=1 | 60092 | 5.4727 | 0.029 | B | Y |
| O75145 | Liprin-alpha-3 OS=Homo sapiens GN=PPFIA3 PE=1 SV=3 | 133413 | 5.3877 | 0.097 | B | Y |
| O75208 | Ubiquinone biosynthesis protein COQ9, mitochondrial OS=Homo sapiens GN=COQ9 PE=1 SV= | 35486 | 5.5342 | 1.659 | B | Y |
| O75306 | NADH dehydrogenase [ubiquinone] iron-sulfur protein 2, mitochondrial OS=Homo sapiens GN=I | 52511 | 7.3008 | 0.120 | B | Y |
| O75323 | Protein NipSnap homolog 2 OS=Homo sapiens GN=GBAS PE=1 SV=1 | 33721 | 9.6211 | 1.666 | B | Y |
| O75335 | Liprin-alpha-4 OS=Homo sapiens GN=PPFIA4 PE=2 SV=3 | 134320 | 6.2739 | 0.013 | B | Y |
| O75367 | Core histone macro-H2A.1 OS=Homo sapiens GN=H2AFY PE=1 SV=4 | 39592 | 10.2305 | 0.095 | B | Y |
| O75368 | SH3 domain-binding glutamic acid-rich-like protein OS=Homo sapiens GN=SH3BGRL PE=1 SV | 12766 | 5.0215 | 0.247 | B | Y |
| O75369 | Filamin-B OS=Homo sapiens GN=FLNB PE=1 SV=2 | 277987 | 5.3555 | 0.152 | B | Y |
| O75380 | NADH dehydrogenase [ubiquinone] iron-sulfur protein 6, mitochondrial OS=Homo sapiens GN=I | 13702 | 8.2939 | 0.383 | B | Y |
| O75396 | Vesicle-trafficking protein SEC22b OS=Homo sapiens GN=SEC22B PE=1 SV=4 | 24577 | 6.501 | 0.101 | B | Y |
| O75436 | Vacuolar protein sorting-associated protein 26A OS=Homo sapiens GN=VPS26A PE=1 SV=2 | 38145 | 6.1172 | 0.760 | B | Y |
| O75475 | PC4 and SFRS1-interacting protein OS=Homo sapiens GN=PSIP1 PE=1 SV=1 | 60066 | 9.605 | 0.170 | B | Y |
| O75489 | NADH dehydrogenase [ubiquinone] iron-sulfur protein 3, mitochondrial OS=Homo sapiens GN=I | 30222 | 7.3623 | 0.270 | B | Y |
| O75533 | Splicing factor 3B subunit 1 OS=Homo sapiens GN=SF3B1 PE=1 SV=3 | 145737 | 6.6533 | 0.402 | B | Y |
| O75569 | Interferon-inducible double-stranded RNA-dependent protein kinase activator A OS=Homo sapie | 34382 | 8.4536 | 0.144 | B | Y |
| O75746 | Calcium-binding mitochondrial carrier protein Aralar1 OS=Homo sapiens GN=SLC25A12 PE=1 | 74714 | 8.4551 | 0.213 | B | Y |
| O75781 | Paralemmin-1 OS=Homo sapiens GN=PALM PE=1 SV=2 | 42050 | 4.7432 | 0.122 | B | Y |
| O75874 | Isocitrate dehydrogenase [NADP] cytoplasmic OS=Homo sapiens GN=IDH1 PE=1 SV=2 | 46629 | 6.5698 | 0.197 | B | Y |
| O75915 | PRA1 family protein 3 OS=Homo sapiens GN=ARL6IP5 PE=1 SV=1 | 21600 | 10.1045 | 0.105 | B | Y |
| O75947 | ATP synthase subunit d, mitochondrial OS=Homo sapiens GN=ATP5H PE=1 SV=3 | 18479 | 5.0244 | 0.782 | B | Y |
| O75955 | Flotillin-1 OS=Homo sapiens GN=FLOT1 PE=1 SV=3 | 47325 | 7.2979 | 0.049 | B | Y |
| O76070 | Gamma-synuclein OS=Homo sapiens GN=SNCG PE=1 SV=2 | 13322 | 4.6626 | 0.269 | B | Y |
| O94760 | N(G),N(G)-dimethylarginine dimethylaminohydrolase 1 OS=Homo sapiens GN=DDAH1 PE=1 S | 31101 | 5.4287 | 0.988 | B | Y |
| O94811 | Tubulin polymerization-promoting protein OS=Homo sapiens GN=TPPP PE=1 SV=1 | 23679 | 9.8892 | 0.322 | B | Y |
| O94819 | Kelch repeat and BTB domain-containing protein 11 OS=Homo sapiens GN=KBTBD11 PE=1 S | 65678 | 5.6909 | 1.055 | B | Y |
| O94826 | Mitochondrial import receptor subunit TOM70 OS=Homo sapiens GN=TOMM70A PE=1 SV=1 | 67412 | 6.772 | 0.189 | B | Y |
| O94856 | Neurofascin OS=Homo sapiens GN=NFASC PE=1 SV=4 | 149933 | 6.208 | 0.001 | B | Y |
| O94925 | Glutaminase kidney isoform, mitochondrial OS=Homo sapiens GN=GLS PE=1 SV=1 | 73413 | 7.6011 | 0.342 | B | Y |
| O94973 | AP-2 complex subunit alpha-2 OS=Homo sapiens GN=AP2A2 PE=1 SV=2 | 103895 | 6.5303 | 0.407 | B | Y |
| O95168 | NADH dehydrogenase [ubiquinone] 1 beta subcomplex subunit 4 OS=Homo sapiens GN=NDUF | 15199 | 10.0942 | 0.210 | B | Y |
| O95202 | LETM1 and EF-hand domain-containing protein 1, mitochondrial OS=Homo sapiens GN=LETM | 83301 | 6.2695 | 0.097 | B | Y |
| O95267 | RAS guanyl-releasing protein 1 OS=Homo sapiens GN=RASGRP1 PE=1 SV=2 | 90344 | 7.834 | 0.164 | B | Y |
| O95294 | RasGAP-activating-like protein 1 OS=Homo sapiens GN=RASAL1 PE=1 SV=3 | 89959 | 6.0601 | 0.101 | B | Y |
| O95336 | 6-phosphogluconolactonase OS=Homo sapiens GN=PGLS PE=1 SV=2 | 27529 | 5.6396 | 0.219 | B | Y |
| O95502 | Neuronal pentraxin receptor OS=Homo sapiens GN=NPTXR PE=3 SV=2 | 52813 | 5.7744 | 0.285 | B | Y |
| O95613 | Pericentrin OS=Homo sapiens GN=PCNT PE=1 SV=4 | 377803 | 5.2588 | 0.089 | B | Y |
| O95716 | Ras-related protein Rab-3D OS=Homo sapiens GN=RAB3D PE=1 SV=1 | 24251 | 4.5674 | 0.482 | B | Y |
| O95741 | Copine-6 OS=Homo sapiens GN=CPNE6 PE=1 SV=3 | 61951 | 5.168 | 0.398 | B | Y |
| O95782 | AP-2 complex subunit alpha-1 OS=Homo sapiens GN=AP2A1 PE=1 SV=3 | 107477 | 6.6255 | 0.664 | B | Y |
| O95865 | N(G),N(G)-dimethylarginine dimethylaminohydrolase 2 OS=Homo sapiens GN=DDAH2 PE=1 S | 29625 | 5.5898 | 0.109 | B | Y |
| O95989 | Diphosphoinositol polyphosphate phosphohydrolase 1 OS=Homo sapiens GN=NUDT3 PE=1 S\ | 19458 | 5.9487 | 0.068 | B | Y |
| P00338 | L-lactate dehydrogenase A chain OS=Homo sapiens GN=LDHA PE=1 SV=2 | 36665 | 8.3657 | 0.675 | B | Y |
| P00352 | Retinal dehydrogenase 1 OS=Homo sapiens GN=ALDH1A1 PE=1 SV=2 | 54826 | 6.2886 | 0.223 | B | Y |
| P00367 | Glutamate dehydrogenase 1, mitochondrial OS=Homo sapiens GN=GLUD1 PE=1 SV=2 | 61359 | 7.7021 | 1.131 | B | Y |
| P00387 | NADH-cytochrome b5 reductase 3 OS=Homo sapiens GN=CYB5R3 PE=1 SV=3 | 34212 | 7.3872 | 0.487 | B | Y |
| P00390 | Glutathione reductase, mitochondrial OS=Homo sapiens GN=GSR PE=1 SV=2 | 56220 | 8.5386 | 0.306 | B | Y |
| P00403 | Cytochrome c oxidase subunit 2 OS=Homo sapiens GN=MT-CO2 PE=1 SV=1 | 25548 | 4.4839 | 1.159 | B | Y |
| P00441 | Superoxide dismutase [Cu-Zn] OS=Homo sapiens GN=SOD1 PE=1 SV=2 | 15925 | 5.666 | 0.464 | B | Y |
| P00491 | Purine nucleoside phosphorylase OS=Homo sapiens GN=PNP PE=1 SV=2 | 32097 | 6.498 | 0.139 | B | Y |
| P00505 | Aspartate aminotransferase, mitochondrial OS=Homo sapiens GN=GOT2 PE=1 SV=3 | 47487 | 9.2637 | 1.303 | B | Y |
| P00558 | Phosphoglycerate kinase 1 OS=Homo sapiens GN=PGK1 PE=1 SV=3 | 44586 | 8.1475 | 1.000 | B | Y |
| P00568 | Adenylate kinase isoenzyme 1 OS=Homo sapiens GN=AK1 PE=1 SV=3 | 21621 | 8.9897 | 0.603 | B | Y |
| P00846 | ATP synthase subunit a OS=Homo sapiens GN=MT-ATP6 PE=1 SV=1 | 24800 | 10.4634 | 1.632 | B | Y |
| P00918 | Carbonic anhydrase 2 OS=Homo sapiens GN=CA2 PE=1 SV=2 | 29227 | 7.0269 | 0.604 | B | Y |
| P00966 | Argininosuccinate synthase OS=Homo sapiens GN=ASS1 PE=1 SV=2 | 46501 | 8.0566 | 0.058 | B | Y |
| P01011 | Alpha-1-antichymotrypsin OS=Homo sapiens GN=SERPINA3 PE=1 SV=2 | 47620 | 5.1812 | 0.049 | B | Y |
| P01034 | Cystatin-C OS=Homo sapiens GN=CST3 PE=1 SV=1 | 15789 | 8.9854 | 0.113 | B | Y |
| P01111 | GTPase NRas OS=Homo sapiens GN=NRAS PE=1 SV=1 | 21215 | 4.8208 | 0.001 | B | Y |
| P01859 | Ig gamma-2 chain C region OS=Homo sapiens GN=IGHG2 PE=1 SV=2 | 35877 | 7.437 | 0.111 | B | Y |
| P01860 | Ig gamma-3 chain C region OS=Homo sapiens GN=IGHG3 PE=1 SV=2 | 41260 | 7.7886 | 0.017 | B | Y |
| P01861 | Ig gamma-4 chain C region OS=Homo sapiens GN=IGHG4 PE=1 SV=1 | 35917 | 7.1104 | 0.027 | B | Y |
| P02008 | Hemoglobin subunit zeta OS=Homo sapiens GN=HBZ PE=1 SV=2 | 15627 | 8.6514 | 5.328 | B | Y |
| P02042 | Hemoglobin subunit delta OS=Homo sapiens GN=HBD PE=1 SV=2 | 16045 | 8.2397 | 0.450 | B | Y |
| P02489 | Alpha-crystallin A chain OS=Homo sapiens GN=CRYAA PE=1 SV=2 | 19896 | 5.7451 | 5.675 | B | Y |
| P02533 | Keratin, type I cytoskeletal 14 OS=Homo sapiens GN=KRT14 PE=1 SV=4 | 51529 | 4.8984 | 0.225 | B | Y |
| P02545 | Prelamin-A/C OS=Homo sapiens GN=LMNA PE=1 SV=1 | 74094 | 6.5815 | 0.198 | B | Y |
| P02649 | Apolipoprotein E OS=Homo sapiens GN=APOE PE=1 SV=1 | 36131 | 5.4829 | 0.095 | B | Y |
| P02768 | Serum albumin OS=Homo sapiens GN=ALB PE=1 SV=2 | 69321 | 5.8608 | 5.382 | B | Y |
| P02769 | Serum albumin OS=Bos taurus GN=ALB PE=1 SV=4 | 69248 | 5.7583 | 3.460 | B | Y |
| P02787 | Serotransferrin OS=Homo sapiens GN=TF PE=1 SV=3 | 77013 | 6.7515 | 0.114 | B | Y |
| P02794 | Ferritin heavy chain OS=Homo sapiens GN=FTH1 PE=1 SV=2 | 21212 | 5.187 | 0.485 | B | Y |
| P04075 | Fructose-bisphosphate aldolase A OS=Homo sapiens GN=ALDOA PE=1 SV=2 | 39395 | 8.0654 | 3.049 | B | Y |

| | | | | | | |
|---|---|---|---|---|---|---|
| P04080 | Cystatin-B OS=Homo sapiens GN=CSTB PE=1 SV=2 | 11132 | 7.5073 | 0.503 | B | Y |
| P04083 | Annexin A1 OS=Homo sapiens GN=ANXA1 PE=1 SV=2 | 38689 | 6.6372 | 0.147 | B | Y |
| P04259 | Keratin, type II cytoskeletal 6B OS=Homo sapiens GN=KRT6B PE=1 SV=5 | 60030 | 8.0537 | 0.068 | B | Y |
| P04264 | Keratin, type II cytoskeletal 1 OS=Homo sapiens GN=KRT1 PE=1 SV=6 | 65998 | 8.2661 | 3.114 | B | Y |
| P04350 | Tubulin beta-4A chain OS=Homo sapiens GN=TUBB4A PE=1 SV=2 | 49553 | 4.5908 | 4.072 | B | Y |
| P04406 | Glyceraldehyde-3-phosphate dehydrogenase OS=Homo sapiens GN=GAPDH PE=1 SV=3 | 36030 | 8.6968 | 7.188 | B | Y |
| P04792 | Heat shock protein beta-1 OS=Homo sapiens GN=HSPB1 PE=1 SV=2 | 22768 | 5.959 | 0.873 | B | Y |
| P04843 | Dolichyl-diphosphooligosaccharide--protein glycosyltransferase subunit 1 OS=Homo sapiens GN | 68526 | 5.9268 | 0.120 | B | Y |
| P04844 | Dolichyl-diphosphooligosaccharide--protein glycosyltransferase subunit 2 OS=Homo sapiens GN | 69241 | 5.332 | 0.523 | B | Y |
| P04899 | Guanine nucleotide-binding protein G(i) subunit alpha-2 OS=Homo sapiens GN=GNAI2 PE=1 S | 40425 | 5.1899 | 0.443 | B | Y |
| P05023 | Sodium/potassium-transporting ATPase subunit alpha-1 OS=Homo sapiens GN=ATP1A1 PE=1 | 112824 | 5.1694 | 1.939 | B | Y |
| P05026 | Sodium/potassium-transporting ATPase subunit beta-1 OS=Homo sapiens GN=ATP1B1 PE=1 S | 35038 | 8.6484 | 2.026 | B | Y |
| P05060 | Secretogranin-1 OS=Homo sapiens GN=CHGB PE=1 SV=2 | 78229 | 4.8354 | 0.076 | B | Y |
| P05062 | Fructose-bisphosphate aldolase B OS=Homo sapiens GN=ALDOB PE=1 SV=2 | 39448 | 7.793 | 0.106 | B | Y |
| P05091 | Aldehyde dehydrogenase, mitochondrial OS=Homo sapiens GN=ALDH2 PE=1 SV=2 | 56345 | 6.6694 | 0.567 | B | Y |
| P05129 | Protein kinase C gamma type OS=Homo sapiens GN=PRKCG PE=1 SV=3 | 78397 | 7.1689 | 0.140 | B | Y |
| P05141 | ADP/ATP translocase 2 OS=Homo sapiens GN=SLC25A5 PE=1 SV=7 | 32831 | 9.9917 | 0.868 | B | Y |
| P05186 | Alkaline phosphatase, tissue-nonspecific isozyme OS=Homo sapiens GN=ALPL PE=1 SV=4 | 57268 | 6.1948 | 0.207 | B | Y |
| P05386 | 60S acidic ribosomal protein P1 OS=Homo sapiens GN=RPLP1 PE=1 SV=1 | 11506 | 4.0005 | 0.171 | B | Y |
| P05388 | 60S acidic ribosomal protein P0 OS=Homo sapiens GN=RPLP0 PE=1 SV=1 | 34251 | 5.606 | 0.525 | B | Y |
| P05413 | Fatty acid-binding protein, heart OS=Homo sapiens GN=FABP3 PE=1 SV=4 | 14848 | 6.3574 | 0.001 | B | Y |
| P05455 | Lupus La protein OS=Homo sapiens GN=SSB PE=1 SV=2 | 46808 | 6.7529 | 0.343 | B | Y |
| P05556 | Integrin beta-1 OS=Homo sapiens GN=ITGB1 PE=1 SV=2 | 88356 | 5.0977 | 0.678 | B | Y |
| P05813 | Beta-crystallin A3 OS=Homo sapiens GN=CRYBA1 PE=1 SV=4 | 25133 | 5.7773 | 0.447 | B | Y |
| P06576 | ATP synthase subunit beta, mitochondrial OS=Homo sapiens GN=ATP5B PE=1 SV=3 | 56524 | 5.0962 | 1.664 | B | Y |
| P06703 | Protein S100-A6 OS=Homo sapiens GN=S100A6 PE=1 SV=1 | 10173 | 5.1665 | 0.756 | B | Y |
| P06733 | Alpha-enolase OS=Homo sapiens GN=ENO1 PE=1 SV=2 | 47139 | 7.1719 | 3.966 | B | Y |
| P06748 | Nucleophosmin OS=Homo sapiens GN=NPM1 PE=1 SV=2 | 32554 | 4.4443 | 0.714 | B | Y |
| P07099 | Epoxide hydrolase 1 OS=Homo sapiens GN=EPHX1 PE=1 SV=1 | 52914 | 6.8364 | 0.323 | B | Y |
| P07195 | L-lactate dehydrogenase B chain OS=Homo sapiens GN=LDHB PE=1 SV=2 | 36615 | 5.6396 | 3.424 | B | Y |
| P07196 | Neurofilament light polypeptide OS=Homo sapiens GN=NEFL PE=1 SV=3 | 61479 | 4.4326 | 1.060 | B | Y |
| P07237 | Protein disulfide-isomerase OS=Homo sapiens GN=P4HB PE=1 SV=3 | 57080 | 4.5645 | 0.220 | B | Y |
| P07339 | Cathepsin D OS=Homo sapiens GN=CTSD PE=1 SV=1 | 44523 | 6.0908 | 1.386 | B | Y |
| P07355 | Annexin A2 OS=Homo sapiens GN=ANXA2 PE=1 SV=2 | 38579 | 7.7842 | 0.419 | B | Y |
| P07437 | Tubulin beta chain OS=Homo sapiens GN=TUBB PE=1 SV=2 | 49638 | 4.5908 | 6.365 | B | Y |
| P07602 | Prosaposin OS=Homo sapiens GN=PSAP PE=1 SV=2 | 58073 | 4.8853 | 1.861 | B | Y |
| P07737 | Profilin-1 OS=Homo sapiens GN=PFN1 PE=1 SV=2 | 15044 | 8.4595 | 0.468 | B | Y |
| P07741 | Adenine phosphoribosyltransferase OS=Homo sapiens GN=APRT PE=1 SV=2 | 19595 | 5.6587 | 0.257 | B | Y |
| P07814 | Bifunctional glutamate/proline--tRNA ligase OS=Homo sapiens GN=EPRS PE=1 SV=5 | 170482 | 6.9756 | 0.228 | B | Y |
| P07858 | Cathepsin B OS=Homo sapiens GN=CTSB PE=1 SV=3 | 37796 | 5.855 | 0.312 | B | Y |
| P07900 | Heat shock protein HSP 90-alpha OS=Homo sapiens GN=HSP90AA1 PE=1 SV=5 | 84606 | 4.7476 | 1.093 | B | Y |
| P07954 | Fumarate hydratase, mitochondrial OS=Homo sapiens GN=FH PE=1 SV=3 | 54602 | 9.0835 | 0.718 | B | Y |
| P08133 | Annexin A6 OS=Homo sapiens GN=ANXA6 PE=1 SV=3 | 75825 | 5.2646 | 0.571 | B | Y |
| P08134 | Rho-related GTP-binding protein RhoC OS=Homo sapiens GN=RHOC PE=1 SV=1 | 21992 | 6.189 | 0.001 | B | Y |
| P08237 | ATP-dependent 6-phosphofructokinase, muscle type OS=Homo sapiens GN=PFKM PE=1 SV=2 | 85128 | 7.8926 | 0.368 | B | Y |
| P08238 | Heat shock protein HSP 90-beta OS=Homo sapiens GN=HSP90AB1 PE=1 SV=4 | 83212 | 4.7739 | 0.459 | B | Y |
| P08247 | Synaptophysin OS=Homo sapiens GN=SYP PE=1 SV=3 | 33823 | 4.4561 | 0.623 | B | Y |
| P08559 | Pyruvate dehydrogenase E1 component subunit alpha, somatic form, mitochondrial OS=Homo s | 43267 | 7.9995 | 0.424 | B | Y |
| P08572 | Collagen alpha-2(IV) chain OS=Homo sapiens GN=COL4A2 PE=1 SV=4 | 167448 | 8.7378 | 0.183 | B | Y |
| P08574 | Cytochrome c1, heme protein, mitochondrial OS=Homo sapiens GN=CYC1 PE=1 SV=3 | 35398 | 9.1831 | 0.307 | B | Y |
| P08582 | Melanotransferrin OS=Homo sapiens GN=MFI2 PE=1 SV=2 | 80163 | 5.5269 | 3.769 | B | Y |
| P08670 | Vimentin OS=Homo sapiens GN=VIM PE=1 SV=4 | 53619 | 4.8633 | 1.889 | B | Y |
| P08708 | 40S ribosomal protein S17 OS=Homo sapiens GN=RPS17 PE=1 SV=2 | 15540 | 10.2407 | 0.181 | B | Y |
| P08754 | Guanine nucleotide-binding protein G(k) subunit alpha OS=Homo sapiens GN=GNAI3 PE=1 SV | 40506 | 5.3584 | 0.430 | B | Y |
| P08758 | Annexin A5 OS=Homo sapiens GN=ANXA5 PE=1 SV=2 | 35914 | 4.7329 | 0.346 | B | Y |
| P08779 | Keratin, type I cytoskeletal 16 OS=Homo sapiens GN=KRT16 PE=1 SV=4 | 51236 | 4.7915 | 0.076 | B | Y |
| P08F94 | Fibrocystin OS=Homo sapiens GN=PKHD1 PE=1 SV=1 | 446417 | 6.1025 | 0.971 | B | Y |
| P09104 | Gamma-enolase OS=Homo sapiens GN=ENO2 PE=1 SV=3 | 47239 | 4.7183 | 0.846 | B | Y |
| P09211 | Glutathione S-transferase P OS=Homo sapiens GN=GSTP1 PE=1 SV=2 | 23341 | 5.2822 | 0.937 | B | Y |
| P09382 | Galectin-1 OS=Homo sapiens GN=LGALS1 PE=1 SV=2 | 14706 | 5.1416 | 0.911 | B | Y |
| P09417 | Dihydropteridine reductase OS=Homo sapiens GN=QDPR PE=1 SV=2 | 25773 | 7.1719 | 0.482 | B | Y |
| P09471 | Guanine nucleotide-binding protein G(o) subunit alpha OS=Homo sapiens GN=GNAO1 PE=1 S | 40024 | 5.1899 | 1.189 | B | Y |
| P09488 | Glutathione S-transferase Mu 1 OS=Homo sapiens GN=GSTM1 PE=1 SV=3 | 25695 | 6.2505 | 0.231 | B | Y |
| P09497 | Clathrin light chain B OS=Homo sapiens GN=CLTB PE=1 SV=1 | 25175 | 4.3726 | 0.198 | B | Y |
| P09543 | 2',3'-cyclic-nucleotide 3'-phosphodiesterase OS=Homo sapiens GN=CNP PE=1 SV=2 | 47548 | 9.3618 | 0.805 | B | Y |
| P09669 | Cytochrome c oxidase subunit 6C OS=Homo sapiens GN=COX6C PE=1 SV=2 | 8775 | 10.752 | 0.302 | B | Y |
| P09874 | Poly [ADP-ribose] polymerase 1 OS=Homo sapiens GN=PARP1 PE=1 SV=4 | 113012 | 9.1611 | 0.488 | B | Y |
| P09936 | Ubiquitin carboxyl-terminal hydrolase isozyme L1 OS=Homo sapiens GN=UCHL1 PE=1 SV=2 | 24808 | 5.1841 | 2.933 | B | Y |
| P09972 | Fructose-bisphosphate aldolase C OS=Homo sapiens GN=ALDOC PE=1 SV=2 | 39431 | 6.4351 | 1.568 | B | Y |
| P0DMV8 | Heat shock 70 kDa protein 1A OS=Homo sapiens GN=HSPA1A PE=1 SV=1 | 70009 | 5.3188 | 3.416 | B | Y |
| P10155 | 60 kDa SS-A/Ro ribonucleoprotein OS=Homo sapiens GN=TROVE2 PE=1 SV=2 | 60631 | 7.9614 | 0.075 | B | Y |
| P10412 | Histone H1.4 OS=Homo sapiens GN=HIST1H1E PE=1 SV=2 | 21852 | 11.5195 | 0.001 | B | Y |
| P10599 | Thioredoxin OS=Homo sapiens GN=TXN PE=1 SV=3 | 11729 | 4.6201 | 0.675 | B | Y |
| P10606 | Cytochrome c oxidase subunit 5B, mitochondrial OS=Homo sapiens GN=COX5B PE=1 SV=2 | 13686 | 9.0688 | 0.384 | B | Y |
| P10636 | Microtubule-associated protein tau OS=Homo sapiens GN=MAPT PE=1 SV=5 | 78879 | 6.2446 | 0.366 | B | Y |
| P10644 | cAMP-dependent protein kinase type I-alpha regulatory subunit OS=Homo sapiens GN=PRKAR | 42955 | 5.0859 | 0.160 | B | Y |
| P10721 | Mast/stem cell growth factor receptor Kit OS=Homo sapiens GN=KIT PE=1 SV=1 | 109794 | 6.5332 | 0.023 | B | Y |
| P10768 | S-formylglutathione hydrolase OS=Homo sapiens GN=ESD PE=1 SV=2 | 31442 | 6.5815 | 0.001 | B | Y |
| P10809 | 60 kDa heat shock protein, mitochondrial OS=Homo sapiens GN=HSPD1 PE=1 SV=2 | 61016 | 5.5503 | 0.678 | B | Y |
| P10909 | Clusterin OS=Homo sapiens GN=CLU PE=1 SV=1 | 52461 | 5.8389 | 0.375 | B | Y |
| P10915 | Hyaluronan and proteoglycan link protein 1 OS=Homo sapiens GN=HAPLN1 PE=2 SV=2 | 40139 | 7.1396 | 0.223 | B | Y |

| | | | | | | |
|---|---|---|---|---|---|---|
| P11021 | 78 kDa glucose-regulated protein OS=Homo sapiens GN=HSPA5 PE=1 SV=2 | 72288 | 4.875 | 1.382 | B | Y |
| P11137 | Microtubule-associated protein 2 OS=Homo sapiens GN=MAP2 PE=1 SV=4 | 199402 | 4.6318 | 0.756 | B | Y |
| P11142 | Heat shock cognate 71 kDa protein OS=Homo sapiens GN=HSPA8 PE=1 SV=1 | 70854 | 5.2002 | 4.862 | B | Y |
| P11177 | Pyruvate dehydrogenase E1 component subunit beta, mitochondrial OS=Homo sapiens GN=PD | 39208 | 6.2021 | 0.466 | B | Y |
| P11216 | Glycogen phosphorylase, brain form OS=Homo sapiens GN=PYGB PE=1 SV=5 | 96634 | 6.3999 | 0.580 | B | Y |
| P11217 | Glycogen phosphorylase, muscle form OS=Homo sapiens GN=PYGM PE=1 SV=6 | 97030 | 6.583 | 0.630 | B | Y |
| P11233 | Ras-related protein Ral-A OS=Homo sapiens GN=RALA PE=1 SV=1 | 23551 | 6.9858 | 0.128 | B | Y |
| P11277 | Spectrin beta chain, erythrocytic OS=Homo sapiens GN=SPTB PE=1 SV=5 | 246313 | 4.9761 | 0.106 | B | Y |
| P11498 | Pyruvate carboxylase, mitochondrial OS=Homo sapiens GN=PC PE=1 SV=2 | 129551 | 6.3706 | 0.055 | B | Y |
| P11766 | Alcohol dehydrogenase class-3 OS=Homo sapiens GN=ADH5 PE=1 SV=4 | 39698 | 7.3125 | 0.177 | B | Y |
| P12004 | Proliferating cell nuclear antigen OS=Homo sapiens GN=PCNA PE=1 SV=1 | 28750 | 4.3755 | 0.285 | B | Y |
| P12036 | Neurofilament heavy polypeptide OS=Homo sapiens GN=NEFH PE=1 SV=4 | 112410 | 5.874 | 0.831 | B | Y |
| P12235 | ADP/ATP translocase 1 OS=Homo sapiens GN=SLC25A4 PE=1 SV=4 | 33043 | 10.0723 | 0.552 | B | Y |
| P12236 | ADP/ATP translocase 3 OS=Homo sapiens GN=SLC25A6 PE=1 SV=4 | 32845 | 10.062 | 1.151 | B | Y |
| P12277 | Creatine kinase B-type OS=Homo sapiens GN=CKB PE=1 SV=1 | 42617 | 5.2178 | 4.716 | B | Y |
| P12532 | Creatine kinase U-type, mitochondrial OS=Homo sapiens GN=CKMT1A PE=1 SV=1 | 47007 | 8.3628 | 0.810 | B | Y |
| P12814 | Alpha-actinin-1 OS=Homo sapiens GN=ACTN1 PE=1 SV=2 | 102992 | 5.0933 | 0.290 | B | Y |
| P13010 | X-ray repair cross-complementing protein 5 OS=Homo sapiens GN=XRCC5 PE=1 SV=3 | 82652 | 5.436 | 0.545 | B | Y |
| P13073 | Cytochrome c oxidase subunit 4 isoform 1, mitochondrial OS=Homo sapiens GN=COX4I1 PE=1 | 19564 | 9.9155 | 0.716 | B | Y |
| P13489 | Ribonuclease inhibitor OS=Homo sapiens GN=RNH1 PE=1 SV=2 | 49941 | 4.5176 | 0.379 | B | Y |
| P13521 | Secretogranin-2 OS=Homo sapiens GN=SCG2 PE=1 SV=2 | 70897 | 4.478 | 0.562 | B | Y |
| P13611 | Versican core protein OS=Homo sapiens GN=VCAN PE=1 SV=3 | 372588 | 4.2349 | 1.905 | B | Y |
| P13639 | Elongation factor 2 OS=Homo sapiens GN=EEF2 PE=1 SV=4 | 95277 | 6.394 | 1.610 | B | Y |
| P13645 | Keratin, type I cytoskeletal 10 OS=Homo sapiens GN=KRT10 PE=1 SV=6 | 58791 | 4.9556 | 2.174 | B | Y |
| P13647 | Keratin, type II cytoskeletal 5 OS=Homo sapiens GN=KRT5 PE=1 SV=3 | 62339 | 7.793 | 0.222 | B | Y |
| P13804 | Electron transfer flavoprotein subunit alpha, mitochondrial OS=Homo sapiens GN=ETFA PE=1 S | 35057 | 8.4858 | 0.290 | B | Y |
| P13861 | cAMP-dependent protein kinase type II-alpha regulatory subunit OS=Homo sapiens GN=PRKAF | 45489 | 4.771 | 0.224 | B | Y |
| P13929 | Beta-enolase OS=Homo sapiens GN=ENO3 PE=1 SV=5 | 46957 | 7.6582 | 0.066 | B | Y |
| P14136 | Glial fibrillary acidic protein OS=Homo sapiens GN=GFAP PE=1 SV=1 | 49849 | 5.2559 | 38.403 | B | Y |
| P14174 | Macrophage migration inhibitory factor OS=Homo sapiens GN=MIF PE=1 SV=4 | 12468 | 7.9922 | 4.125 | B | Y |
| P14415 | Sodium/potassium-transporting ATPase subunit beta-2 OS=Homo sapiens GN=ATP1B2 PE=1 S | 33345 | 8.3438 | 0.335 | B | Y |
| P14618 | Pyruvate kinase PKM OS=Homo sapiens GN=PKM PE=1 SV=4 | 57900 | 7.7534 | 2.711 | B | Y |
| P14625 | Endoplasmin OS=Homo sapiens GN=HSP90B1 PE=1 SV=1 | 92411 | 4.5645 | 0.658 | B | Y |
| P14854 | Cytochrome c oxidase subunit 6B1 OS=Homo sapiens GN=COX6B1 PE=1 SV=2 | 10185 | 6.8657 | 0.509 | B | Y |
| P14868 | Aspartate--tRNA ligase, cytoplasmic OS=Homo sapiens GN=DARS PE=1 SV=2 | 57100 | 6.0894 | 1.192 | B | Y |
| P14923 | Junction plakoglobin OS=Homo sapiens GN=JUP PE=1 SV=3 | 81692 | 5.6982 | 0.180 | B | Y |
| P14927 | Cytochrome b-c1 complex subunit 7 OS=Homo sapiens GN=UQCRB PE=1 SV=2 | 13521 | 9.2446 | 0.425 | B | Y |
| P15104 | Glutamine synthetase OS=Homo sapiens GN=GLUL PE=1 SV=4 | 42037 | 6.4424 | 0.186 | B | Y |
| P15121 | Aldose reductase OS=Homo sapiens GN=AKR1B1 PE=1 SV=3 | 35830 | 6.5508 | 0.280 | B | Y |
| P15311 | Ezrin OS=Homo sapiens GN=EZR PE=1 SV=4 | 69369 | 5.8726 | 0.077 | B | Y |
| P15880 | 40S ribosomal protein S2 OS=Homo sapiens GN=RPS2 PE=1 SV=2 | 31304 | 10.6465 | 0.552 | B | Y |
| P16104 | Histone H2AX OS=Homo sapiens GN=H2AFX PE=1 SV=2 | 15135 | 11.1563 | 0.001 | B | Y |
| P16152 | Carbonyl reductase [NADPH] 1 OS=Homo sapiens GN=CBR1 PE=1 SV=3 | 30355 | 8.417 | 0.708 | B | Y |
| P16234 | Platelet-derived growth factor receptor alpha OS=Homo sapiens GN=PDGFRA PE=1 SV=1 | 122591 | 4.8779 | 0.068 | B | Y |
| P16298 | Serine/threonine-protein phosphatase 2B catalytic subunit beta isoform OS=Homo sapiens GN= | 58986 | 5.5166 | 0.034 | B | Y |
| P16615 | Sarcoplasmic/endoplasmic reticulum calcium ATPase 2 OS=Homo sapiens GN=ATP2A2 PE=1 | 114682 | 5.0522 | 3.187 | B | Y |
| P16870 | Carboxypeptidase E OS=Homo sapiens GN=CPE PE=1 SV=1 | 53117 | 4.8472 | 0.150 | B | Y |
| P17066 | Heat shock 70 kDa protein 6 OS=Homo sapiens GN=HSPA6 PE=1 SV=2 | 70984 | 5.7319 | 1.080 | B | Y |
| P17174 | Aspartate aminotransferase, cytoplasmic OS=Homo sapiens GN=GOT1 PE=1 SV=3 | 46218 | 6.5698 | 0.303 | B | Y |
| P17252 | Protein kinase C alpha type OS=Homo sapiens GN=PRKCA PE=1 SV=4 | 76700 | 6.6138 | 0.250 | B | Y |
| P17302 | Gap junction alpha-1 protein OS=Homo sapiens GN=GJA1 PE=1 SV=2 | 42980 | 8.8887 | 0.267 | B | Y |
| P17600 | Synapsin-1 OS=Homo sapiens GN=SYN1 PE=1 SV=3 | 74065 | 10.1895 | 2.872 | B | Y |
| P17612 | cAMP-dependent protein kinase catalytic subunit alpha OS=Homo sapiens GN=PRKACA PE=1 | 40564 | 9.126 | 0.029 | B | Y |
| P17661 | Desmin OS=Homo sapiens GN=DES PE=1 SV=3 | 53503 | 5.0303 | 0.245 | B | Y |
| P17677 | Neuromodulin OS=Homo sapiens GN=GAP43 PE=1 SV=1 | 24787 | 4.4473 | 0.133 | B | Y |
| P17844 | Probable ATP-dependent RNA helicase DDX5 OS=Homo sapiens GN=DDX5 PE=1 SV=1 | 69104 | 9.0996 | 0.069 | B | Y |
| P17858 | ATP-dependent 6-phosphofructokinase, liver type OS=Homo sapiens GN=PFKL PE=1 SV=6 | 84964 | 7.2261 | 0.230 | B | Y |
| P17948 | Vascular endothelial growth factor receptor 1 OS=Homo sapiens GN=FLT1 PE=1 SV=2 | 150672 | 8.3247 | 0.079 | B | Y |
| P17987 | T-complex protein 1 subunit alpha OS=Homo sapiens GN=TCP1 PE=1 SV=1 | 60305 | 5.7129 | 1.586 | B | Y |
| P18124 | 60S ribosomal protein L7 OS=Homo sapiens GN=RPL7 PE=1 SV=1 | 29207 | 11.0654 | 0.282 | B | Y |
| P18206 | Vinculin OS=Homo sapiens GN=VCL PE=1 SV=4 | 123721 | 5.3481 | 0.179 | B | Y |
| P18669 | Phosphoglycerate mutase 1 OS=Homo sapiens GN=PGAM1 PE=1 SV=2 | 28785 | 6.7866 | 0.800 | B | Y |
| P18859 | ATP synthase-coupling factor 6, mitochondrial OS=Homo sapiens GN=ATP5J PE=1 SV=1 | 12579 | 9.9829 | 0.236 | B | Y |
| P19012 | Keratin, type I cytoskeletal 15 OS=Homo sapiens GN=KRT15 PE=1 SV=3 | 49181 | 4.5132 | 0.422 | B | Y |
| P19013 | Keratin, type II cytoskeletal 4 OS=Homo sapiens GN=KRT4 PE=1 SV=4 | 57249 | 6.2153 | 0.244 | B | Y |
| P19022 | Cadherin-2 OS=Homo sapiens GN=CDH2 PE=1 SV=4 | 99747 | 4.4429 | 1.880 | B | Y |
| P19086 | Guanine nucleotide-binding protein G(z) subunit alpha OS=Homo sapiens GN=GNAZ PE=2 SV= | 40897 | 7.4912 | 0.047 | B | Y |
| P19338 | Nucleolin OS=Homo sapiens GN=NCL PE=1 SV=3 | 76568 | 4.4004 | 1.160 | B | Y |
| P19367 | Hexokinase-1 OS=Homo sapiens GN=HK1 PE=1 SV=3 | 102420 | 6.3472 | 0.786 | B | Y |
| P20020 | Plasma membrane calcium-transporting ATPase 1 OS=Homo sapiens GN=ATP2B1 PE=1 SV=3 | 138667 | 5.644 | 0.209 | B | Y |
| P20073 | Annexin A7 OS=Homo sapiens GN=ANXA7 PE=1 SV=3 | 52705 | 5.3525 | 0.148 | B | Y |
| P20336 | Ras-related protein Rab-3A OS=Homo sapiens GN=RAB3A PE=1 SV=1 | 24968 | 4.6626 | 1.907 | B | Y |
| P20339 | Ras-related protein Rab-5A OS=Homo sapiens GN=RAB5A PE=1 SV=2 | 23643 | 8.2515 | 0.140 | B | Y |
| P20340 | Ras-related protein Rab-6A OS=Homo sapiens GN=RAB6A PE=1 SV=3 | 23577 | 5.2266 | 0.157 | B | Y |
| P20648 | Potassium-transporting ATPase alpha chain 1 OS=Homo sapiens GN=ATP4A PE=2 SV=5 | 114045 | 5.4521 | 0.083 | B | Y |
| P20674 | Cytochrome c oxidase subunit 5A, mitochondrial OS=Homo sapiens GN=COX5A PE=1 SV=2 | 16751 | 6.3574 | 0.533 | B | Y |
| P20916 | Myelin-associated glycoprotein OS=Homo sapiens GN=MAG PE=1 SV=1 | 69024 | 4.7871 | 0.083 | B | Y |
| P21266 | Glutathione S-transferase Mu 3 OS=Homo sapiens GN=GSTM3 PE=1 SV=3 | 26542 | 5.2075 | 0.784 | B | Y |
| P21281 | V-type proton ATPase subunit B, brain isoform OS=Homo sapiens GN=ATP6V1B2 PE=1 SV=3 | 56464 | 5.4492 | 0.658 | B | Y |
| P21283 | V-type proton ATPase subunit C 1 OS=Homo sapiens GN=ATP6V1C1 PE=1 SV=4 | 43914 | 7.3037 | 0.062 | B | Y |
| P21291 | Cysteine and glycine-rich protein 1 OS=Homo sapiens GN=CSRP1 PE=1 SV=3 | 20553 | 8.5532 | 0.267 | B | Y |

| | | | | | | |
|---|---|---|---|---|---|---|
| P21397 | Amine oxidase [flavin-containing] A OS=Homo sapiens GN=MAOA PE=1 SV=1 | 59643 | 7.7842 | 0.160 | B | Y |
| P21579 | Synaptotagmin-1 OS=Homo sapiens GN=SYT1 PE=1 SV=1 | 47542 | 8.2061 | 0.493 | B | Y |
| P21796 | Voltage-dependent anion-selective channel protein 1 OS=Homo sapiens GN=VDAC1 PE=1 SV= | 30753 | 8.8682 | 2.531 | B | Y |
| P22314 | Ubiquitin-like modifier-activating enzyme 1 OS=Homo sapiens GN=UBA1 PE=1 SV=3 | 117774 | 5.3789 | 0.585 | B | Y |
| P22392 | Nucleoside diphosphate kinase B OS=Homo sapiens GN=NME2 PE=1 SV=1 | 17286 | 8.7568 | 0.453 | B | Y |
| P22626 | Heterogeneous nuclear ribonucleoproteins A2/B1 OS=Homo sapiens GN=HNRNPA2B1 PE=1 S | 37406 | 9.1948 | 3.317 | B | Y |
| P22695 | Cytochrome b-c1 complex subunit 2, mitochondrial OS=Homo sapiens GN=UQCRC2 PE=1 SV= | 48412 | 8.8784 | 0.754 | B | Y |
| P23246 | Splicing factor, proline- and glutamine-rich OS=Homo sapiens GN=SFPQ PE=1 SV=2 | 76101 | 9.772 | 0.287 | B | Y |
| P23284 | Peptidyl-prolyl cis-trans isomerase B OS=Homo sapiens GN=PPIB PE=1 SV=2 | 23727 | 9.8511 | 0.337 | B | Y |
| P23297 | Protein S100-A1 OS=Homo sapiens GN=S100A1 PE=1 SV=2 | 10539 | 4.1865 | 1.038 | B | Y |
| P23381 | Tryptophan--tRNA ligase, cytoplasmic OS=Homo sapiens GN=WARS PE=1 SV=2 | 53131 | 5.7803 | 0.102 | B | Y |
| P23396 | 40S ribosomal protein S3 OS=Homo sapiens GN=RPS3 PE=1 SV=2 | 26671 | 10.0415 | 0.303 | B | Y |
| P23471 | Receptor-type tyrosine-protein phosphatase zeta OS=Homo sapiens GN=PTPRZ1 PE=1 SV=4 | 254427 | 4.5718 | 0.507 | B | Y |
| P23634 | Plasma membrane calcium-transporting ATPase 4 OS=Homo sapiens GN=ATP2B4 PE=1 SV=2 | 137832 | 6.1597 | 0.178 | B | Y |
| P24534 | Elongation factor 1-beta OS=Homo sapiens GN=EEF1B2 PE=1 SV=3 | 24748 | 4.3037 | 0.333 | B | Y |
| P24539 | ATP synthase F(0) complex subunit B1, mitochondrial OS=Homo sapiens GN=ATP5F1 PE=1 S\ | 28890 | 9.6563 | 0.532 | B | Y |
| P24752 | Acetyl-CoA acetyltransferase, mitochondrial OS=Homo sapiens GN=ACAT1 PE=1 SV=1 | 45170 | 9.1201 | 0.200 | B | Y |
| P24821 | Tenascin OS=Homo sapiens GN=TNC PE=1 SV=3 | 240697 | 4.5967 | 0.172 | B | Y |
| P25398 | 40S ribosomal protein S12 OS=Homo sapiens GN=RPS12 PE=1 SV=3 | 14505 | 6.9844 | 0.218 | B | Y |
| P25705 | ATP synthase subunit alpha, mitochondrial OS=Homo sapiens GN=ATP5A1 PE=1 SV=1 | 59713 | 9.4321 | 2.537 | B | Y |
| P25786 | Proteasome subunit alpha type-1 OS=Homo sapiens GN=PSMA1 PE=1 SV=1 | 29536 | 6.1523 | 0.188 | B | Y |
| P25788 | Proteasome subunit alpha type-3 OS=Homo sapiens GN=PSMA3 PE=1 SV=2 | 28415 | 5.0171 | 0.117 | B | Y |
| P26038 | Moesin OS=Homo sapiens GN=MSN PE=1 SV=3 | 67777 | 6.0103 | 0.318 | B | Y |
| P26196 | Probable ATP-dependent RNA helicase DDX6 OS=Homo sapiens GN=DDX6 PE=1 SV=2 | 54382 | 8.8359 | 0.067 | B | Y |
| P26373 | 60S ribosomal protein L13 OS=Homo sapiens GN=RPL13 PE=1 SV=4 | 24246 | 12.0586 | 0.161 | B | Y |
| P26641 | Elongation factor 1-gamma OS=Homo sapiens GN=EEF1G PE=1 SV=3 | 50087 | 6.2358 | 0.591 | B | Y |
| P26885 | Peptidyl-prolyl cis-trans isomerase FKBP2 OS=Homo sapiens GN=FKBP2 PE=1 SV=2 | 15639 | 9.5522 | 0.164 | B | Y |
| P27105 | Erythrocyte band 7 integral membrane protein OS=Homo sapiens GN=STOM PE=1 SV=3 | 31710 | 7.9878 | 0.070 | B | Y |
| P27338 | Amine oxidase [flavin-containing] B OS=Homo sapiens GN=MAOB PE=1 SV=3 | 58725 | 7.248 | 0.497 | B | Y |
| P27348 | 14-3-3 protein theta OS=Homo sapiens GN=YWHAQ PE=1 SV=1 | 27746 | 4.4854 | 0.548 | B | Y |
| P27361 | Mitogen-activated protein kinase 3 OS=Homo sapiens GN=MAPK3 PE=1 SV=4 | 43108 | 6.2886 | 0.419 | B | Y |
| P27482 | Calmodulin-like protein 3 OS=Homo sapiens GN=CALML3 PE=1 SV=2 | 16879 | 4.0972 | 1.469 | B | Y |
| P27797 | Calreticulin OS=Homo sapiens GN=CALR PE=1 SV=1 | 48111 | 4.0942 | 1.175 | B | Y |
| P27824 | Calnexin OS=Homo sapiens GN=CANX PE=1 SV=2 | 67525 | 4.2686 | 0.487 | B | Y |
| P28066 | Proteasome subunit alpha type-5 OS=Homo sapiens GN=PSMA5 PE=1 SV=3 | 26394 | 4.5439 | 1.244 | B | Y |
| P28070 | Proteasome subunit beta type-4 OS=Homo sapiens GN=PSMB4 PE=1 SV=4 | 29185 | 5.6001 | 0.782 | B | Y |
| P28072 | Proteasome subunit beta type-6 OS=Homo sapiens GN=PSMB6 PE=1 SV=4 | 25341 | 4.6069 | 0.195 | B | Y |
| P28074 | Proteasome subunit beta type-5 OS=Homo sapiens GN=PSMB5 PE=1 SV=3 | 28462 | 6.5083 | 0.773 | B | Y |
| P28161 | Glutathione S-transferase Mu 2 OS=Homo sapiens GN=GSTM2 PE=1 SV=2 | 25727 | 5.9531 | 0.595 | B | Y |
| P28331 | NADH-ubiquinone oxidoreductase 75 kDa subunit, mitochondrial OS=Homo sapiens GN=NDUF | 79416 | 5.8184 | 0.265 | B | Y |
| P28482 | Mitogen-activated protein kinase 1 OS=Homo sapiens GN=MAPK1 PE=1 SV=3 | 41363 | 6.5317 | 0.661 | B | Y |
| P28838 | Cytosol aminopeptidase OS=Homo sapiens GN=LAP3 PE=1 SV=3 | 56130 | 7.9072 | 0.194 | B | Y |
| P28907 | ADP-ribosyl cyclase/cyclic ADP-ribose hydrolase 1 OS=Homo sapiens GN=CD38 PE=1 SV=2 | 34306 | 7.5264 | 0.074 | B | Y |
| P29401 | Transketolase OS=Homo sapiens GN=TKT PE=1 SV=3 | 67834 | 7.478 | 2.917 | B | Y |
| P30038 | Delta-1-pyrroline-5-carboxylate dehydrogenase, mitochondrial OS=Homo sapiens GN=ALDH4A | 61680 | 8.0566 | 0.190 | B | Y |
| P30040 | Endoplasmic reticulum resident protein 29 OS=Homo sapiens GN=ERP29 PE=1 SV=4 | 28975 | 7.2832 | 0.298 | B | Y |
| P30041 | Peroxiredoxin-6 OS=Homo sapiens GN=PRDX6 PE=1 SV=3 | 25019 | 5.9575 | 1.954 | B | Y |
| P30044 | Peroxiredoxin-5, mitochondrial OS=Homo sapiens GN=PRDX5 PE=1 SV=4 | 22072 | 8.9839 | 0.597 | B | Y |
| P30048 | Thioredoxin-dependent peroxide reductase, mitochondrial OS=Homo sapiens GN=PRDX3 PE=' | 27675 | 7.6934 | 0.507 | B | Y |
| P30049 | ATP synthase subunit delta, mitochondrial OS=Homo sapiens GN=ATP5D PE=1 SV=2 | 17479 | 5.1914 | 0.848 | B | Y |
| P30050 | 60S ribosomal protein L12 OS=Homo sapiens GN=RPL12 PE=1 SV=1 | 17807 | 9.8965 | 0.262 | B | Y |
| P30084 | Enoyl-CoA hydratase, mitochondrial OS=Homo sapiens GN=ECHS1 PE=1 SV=4 | 31367 | 8.0728 | 0.289 | B | Y |
| P30085 | UMP-CMP kinase OS=Homo sapiens GN=CMPK1 PE=1 SV=3 | 22208 | 5.2646 | 0.001 | B | Y |
| P30086 | Phosphatidylethanolamine-binding protein 1 OS=Homo sapiens GN=PEBP1 PE=1 SV=3 | 21043 | 7.3901 | 1.878 | B | Y |
| P30101 | Protein disulfide-isomerase A3 OS=Homo sapiens GN=PDIA3 PE=1 SV=4 | 56746 | 5.9312 | 0.420 | B | Y |
| P30153 | Serine/threonine-protein phosphatase 2A 65 kDa regulatory subunit A alpha isoform OS=Homo | 65267 | 4.8149 | 0.880 | B | Y |
| P30154 | Serine/threonine-protein phosphatase 2A 65 kDa regulatory subunit A beta isoform OS=Homo s | 66171 | 4.645 | 0.214 | B | Y |
| P30622 | CAP-Gly domain-containing linker protein 1 OS=Homo sapiens GN=CLIP1 PE=1 SV=2 | 162146 | 5.1138 | 3.180 | B | Y |
| P31146 | Coronin-1A OS=Homo sapiens GN=CORO1A PE=1 SV=4 | 50993 | 6.2417 | 0.182 | B | Y |
| P31150 | Rab GDP dissociation inhibitor alpha OS=Homo sapiens GN=GDI1 PE=1 SV=2 | 50550 | 4.8135 | 1.131 | B | Y |
| P31153 | S-adenosylmethionine synthase isoform type-2 OS=Homo sapiens GN=MAT2A PE=1 SV=1 | 43633 | 6.0117 | 0.358 | B | Y |
| P31629 | Transcription factor HIVEP2 OS=Homo sapiens GN=HIVEP2 PE=1 SV=2 | 268882 | 6.4951 | 0.103 | B | Y |
| P31930 | Cytochrome b-c1 complex subunit 1, mitochondrial OS=Homo sapiens GN=UQCRC1 PE=1 SV= | 52612 | 5.9092 | 0.620 | B | Y |
| P31939 | Bifunctional purine biosynthesis protein PURH OS=Homo sapiens GN=ATIC PE=1 SV=3 | 64575 | 6.2622 | 0.309 | B | Y |
| P31942 | Heterogeneous nuclear ribonucleoprotein H3 OS=Homo sapiens GN=HNRNPH3 PE=1 SV=2 | 36903 | 6.3999 | 0.319 | B | Y |
| P31946 | 14-3-3 protein beta/alpha OS=Homo sapiens GN=YWHAB PE=1 SV=3 | 28064 | 4.5674 | 3.050 | B | Y |
| P31948 | Stress-induced-phosphoprotein 1 OS=Homo sapiens GN=STIP1 PE=1 SV=1 | 62599 | 6.3867 | 0.429 | B | Y |
| P32119 | Peroxiredoxin-2 OS=Homo sapiens GN=PRDX2 PE=1 SV=5 | 21878 | 5.5679 | 3.116 | B | Y |
| P33778 | Histone H2B type 1-B OS=Homo sapiens GN=HIST1H2BB PE=1 SV=2 | 13941 | 10.7402 | 0.472 | B | Y |
| P33991 | DNA replication licensing factor MCM4 OS=Homo sapiens GN=MCM4 PE=1 SV=5 | 96497 | 6.2725 | 0.245 | B | Y |
| P34932 | Heat shock 70 kDa protein 4 OS=Homo sapiens GN=HSPA4 PE=1 SV=4 | 94271 | 4.9131 | 2.605 | B | Y |
| P35232 | Prohibitin OS=Homo sapiens GN=PHB PE=1 SV=1 | 29785 | 5.4302 | 1.008 | B | Y |
| P35520 | Cystathionine beta-synthase OS=Homo sapiens GN=CBS PE=1 SV=2 | 60548 | 6.1919 | 0.624 | B | Y |
| P35527 | Keratin, type I cytoskeletal 9 OS=Homo sapiens GN=KRT9 PE=1 SV=3 | 62026 | 4.9585 | 0.632 | B | Y |
| P35579 | Myosin-9 OS=Homo sapiens GN=MYH9 PE=1 SV=4 | 226390 | 5.3364 | 0.167 | B | Y |
| P35580 | Myosin-10 OS=Homo sapiens GN=MYH10 PE=1 SV=3 | 228856 | 5.272 | 0.069 | B | Y |
| P35609 | Alpha-actinin-2 OS=Homo sapiens GN=ACTN2 PE=1 SV=1 | 103788 | 5.1533 | 0.029 | B | Y |
| P35612 | Beta-adducin OS=Homo sapiens GN=ADD2 PE=1 SV=3 | 80803 | 5.5635 | 0.181 | B | Y |
| P35637 | RNA-binding protein FUS OS=Homo sapiens GN=FUS PE=1 SV=1 | 53393 | 9.4951 | 0.612 | B | Y |
| P35749 | Myosin-11 OS=Homo sapiens GN=MYH11 PE=1 SV=3 | 227197 | 5.25 | 0.049 | B | Y |
| P35908 | Keratin, type II cytoskeletal 2 epidermal OS=Homo sapiens GN=KRT2 PE=1 SV=2 | 65393 | 8.0537 | 1.079 | B | Y |

| | | | | | | |
|---|---|---|---|---|---|---|
| P35914 | Hydroxymethylglutaryl-CoA lyase, mitochondrial OS=Homo sapiens GN=HMGCL PE=1 SV=2 | 34337 | 8.625 | 0.202 | B | Y |
| P36542 | ATP synthase subunit gamma, mitochondrial OS=Homo sapiens GN=ATP5C1 PE=1 SV=1 | 32975 | 9.561 | 1.391 | B | Y |
| P36543 | V-type proton ATPase subunit E 1 OS=Homo sapiens GN=ATP6V1E1 PE=1 SV=1 | 26128 | 8.4419 | 0.090 | B | Y |
| P36578 | 60S ribosomal protein L4 OS=Homo sapiens GN=RPL4 PE=1 SV=5 | 47667 | 11.4961 | 0.179 | B | Y |
| P36871 | Phosphoglucomutase-1 OS=Homo sapiens GN=PGM1 PE=1 SV=3 | 61410 | 6.3032 | 0.084 | B | Y |
| P36888 | Receptor-type tyrosine-protein kinase FLT3 OS=Homo sapiens GN=FLT3 PE=1 SV=2 | 112830 | 5.3555 | 0.049 | B | Y |
| P36957 | Dihydrolipoyllysine-residue succinyltransferase component of 2-oxoglutarate dehydrogenase co | 48724 | 9.2769 | 0.203 | B | Y |
| P37802 | Transgelin-2 OS=Homo sapiens GN=TAGLN2 PE=1 SV=3 | 22377 | 8.4492 | 0.180 | B | Y |
| P37837 | Transaldolase OS=Homo sapiens GN=TALDO1 PE=1 SV=2 | 37516 | 6.375 | 0.135 | B | Y |
| P38405 | Guanine nucleotide-binding protein G(olf) subunit alpha OS=Homo sapiens GN=GNAL PE=1 S\ | 44280 | 6.2183 | 0.567 | B | Y |
| P38606 | V-type proton ATPase catalytic subunit A OS=Homo sapiens GN=ATP6V1A PE=1 SV=2 | 68260 | 5.1855 | 0.710 | B | Y |
| P38646 | Stress-70 protein, mitochondrial OS=Homo sapiens GN=HSPA9 PE=1 SV=2 | 73634 | 5.7803 | 0.849 | B | Y |
| P38919 | Eukaryotic initiation factor 4A-III OS=Homo sapiens GN=EIF4A3 PE=1 SV=4 | 46841 | 6.2944 | 0.088 | B | Y |
| P39023 | 60S ribosomal protein L3 OS=Homo sapiens GN=RPL3 PE=1 SV=2 | 46079 | 10.6187 | 0.238 | B | Y |
| P40123 | Adenylyl cyclase-associated protein 2 OS=Homo sapiens GN=CAP2 PE=1 SV=1 | 52790 | 5.9253 | 0.260 | B | Y |
| P40227 | T-complex protein 1 subunit zeta OS=Homo sapiens GN=CCT6A PE=1 SV=3 | 57987 | 6.2241 | 0.482 | B | Y |
| P40925 | Malate dehydrogenase, cytoplasmic OS=Homo sapiens GN=MDH1 PE=1 SV=4 | 36403 | 7.1704 | 1.378 | B | Y |
| P40926 | Malate dehydrogenase, mitochondrial OS=Homo sapiens GN=MDH2 PE=1 SV=3 | 35480 | 8.8213 | 3.250 | B | Y |
| P40939 | Trifunctional enzyme subunit alpha, mitochondrial OS=Homo sapiens GN=HADHA PE=1 SV=2 | 82946 | 9.3413 | 0.183 | B | Y |
| P41219 | Peripherin OS=Homo sapiens GN=PRPH PE=1 SV=2 | 53618 | 5.209 | 0.119 | B | Y |
| P41250 | Glycine--tRNA ligase OS=Homo sapiens GN=GARS PE=1 SV=3 | 83112 | 6.6138 | 0.298 | B | Y |
| P42025 | Beta-centractin OS=Homo sapiens GN=ACTR1B PE=1 SV=1 | 42266 | 5.9546 | 0.348 | B | Y |
| P42166 | Lamina-associated polypeptide 2, isoform alpha OS=Homo sapiens GN=TMPO PE=1 SV=2 | 75445 | 7.5132 | 0.234 | B | Y |
| P42262 | Glutamate receptor 2 OS=Homo sapiens GN=GRIA2 PE=1 SV=3 | 98758 | 7.4341 | 0.066 | B | Y |
| P42677 | 40S ribosomal protein S27 OS=Homo sapiens GN=RPS27 PE=1 SV=3 | 9454 | 9.835 | 0.001 | B | Y |
| P43004 | Excitatory amino acid transporter 2 OS=Homo sapiens GN=SLC1A2 PE=1 SV=2 | 62063 | 6.0762 | 1.987 | B | Y |
| P43007 | Neutral amino acid transporter A OS=Homo sapiens GN=SLC1A4 PE=1 SV=1 | 55687 | 5.8315 | 0.100 | B | Y |
| P43034 | Platelet-activating factor acetylhydrolase IB subunit alpha OS=Homo sapiens GN=PAFAH1B1 P | 46608 | 7.0151 | 0.166 | B | Y |
| P43304 | Glycerol-3-phosphate dehydrogenase, mitochondrial OS=Homo sapiens GN=GPD2 PE=1 SV=3 | 80801 | 7.5454 | 0.282 | B | Y |
| P45880 | Voltage-dependent anion-selective channel protein 2 OS=Homo sapiens GN=VDAC2 PE=1 SV= | 31546 | 7.4678 | 1.634 | B | Y |
| P46777 | 60S ribosomal protein L5 OS=Homo sapiens GN=RPL5 PE=1 SV=3 | 34340 | 10.0151 | 0.431 | B | Y |
| P46783 | 40S ribosomal protein S10 OS=Homo sapiens GN=RPS10 PE=1 SV=1 | 18885 | 10.5088 | 0.291 | B | Y |
| P46821 | Microtubule-associated protein 1B OS=Homo sapiens GN=MAP1B PE=1 SV=2 | 270465 | 4.5381 | 0.708 | B | Y |
| P47755 | F-actin-capping protein subunit alpha-2 OS=Homo sapiens GN=CAPZA2 PE=1 SV=3 | 32928 | 5.4756 | 0.345 | B | Y |
| P47914 | 60S ribosomal protein L29 OS=Homo sapiens GN=RPL29 PE=1 SV=2 | 17741 | 12.0791 | 0.089 | B | Y |
| P47985 | Cytochrome b-c1 complex subunit Rieske, mitochondrial OS=Homo sapiens GN=UQCRFS1 PE | 29649 | 8.3936 | 0.405 | B | Y |
| P48047 | ATP synthase subunit O, mitochondrial OS=Homo sapiens GN=ATP5O PE=1 SV=1 | 23262 | 10.3638 | 1.048 | B | Y |
| P48668 | Keratin, type II cytoskeletal 6C OS=Homo sapiens GN=KRT6C PE=1 SV=3 | 59988 | 8.0537 | 0.116 | B | Y |
| P48735 | Isocitrate dehydrogenase [NADP], mitochondrial OS=Homo sapiens GN=IDH2 PE=1 SV=2 | 50876 | 8.855 | 1.329 | B | Y |
| P49189 | 4-trimethylaminobutyraldehyde dehydrogenase OS=Homo sapiens GN=ALDH9A1 PE=1 SV=3 | 53767 | 5.562 | 0.518 | B | Y |
| P49411 | Elongation factor Tu, mitochondrial OS=Homo sapiens GN=TUFM PE=1 SV=2 | 49510 | 7.3726 | 0.483 | B | Y |
| P49418 | Amphiphysin OS=Homo sapiens GN=AMPH PE=1 SV=1 | 76210 | 4.374 | 0.259 | B | Y |
| P49419 | Alpha-aminoadipic semialdehyde dehydrogenase OS=Homo sapiens GN=ALDH7A1 PE=1 SV= | 58450 | 7.938 | 0.689 | B | Y |
| P49721 | Proteasome subunit beta type-2 OS=Homo sapiens GN=PSMB2 PE=1 SV=1 | 22821 | 6.6079 | 0.183 | B | Y |
| P49915 | GMP synthase [glutamine-hydrolyzing] OS=Homo sapiens GN=GMPS PE=1 SV=1 | 76667 | 6.4175 | 0.840 | B | Y |
| P50135 | Histamine N-methyltransferase OS=Homo sapiens GN=HNMT PE=1 SV=1 | 33273 | 5.0127 | 0.186 | B | Y |
| P50148 | Guanine nucleotide-binding protein G(q) subunit alpha OS=Homo sapiens GN=GNAQ PE=1 SV | 42115 | 5.3408 | 0.166 | B | Y |
| P50213 | Isocitrate dehydrogenase [NAD] subunit alpha, mitochondrial OS=Homo sapiens GN=IDH3A PE | 39566 | 6.4907 | 0.583 | B | Y |
| P50395 | Rab GDP dissociation inhibitor beta OS=Homo sapiens GN=GDI2 PE=1 SV=2 | 50630 | 6.0557 | 0.374 | B | Y |
| P50897 | Palmitoyl-protein thioesterase 1 OS=Homo sapiens GN=PPT1 PE=1 SV=1 | 34171 | 6.0659 | 0.271 | B | Y |
| P50990 | T-complex protein 1 subunit theta OS=Homo sapiens GN=CCT8 PE=1 SV=4 | 59582 | 5.272 | 0.476 | B | Y |
| P50991 | T-complex protein 1 subunit delta OS=Homo sapiens GN=CCT4 PE=1 SV=4 | 57887 | 7.7827 | 0.276 | B | Y |
| P50995 | Annexin A11 OS=Homo sapiens GN=ANXA11 PE=1 SV=1 | 54355 | 7.6143 | 0.098 | B | Y |
| P51148 | Ras-related protein Rab-5C OS=Homo sapiens GN=RAB5C PE=1 SV=2 | 23467 | 8.5796 | 0.117 | B | Y |
| P51149 | Ras-related protein Rab-7a OS=Homo sapiens GN=RAB7A PE=1 SV=1 | 23474 | 6.5742 | 0.536 | B | Y |
| P51649 | Succinate-semialdehyde dehydrogenase, mitochondrial OS=Homo sapiens GN=ALDH5A1 PE= | 57178 | 8.2324 | 0.701 | B | Y |
| P51665 | 26S proteasome non-ATPase regulatory subunit 7 OS=Homo sapiens GN=PSMD7 PE=1 SV=2 | 37002 | 6.3003 | 2.638 | B | Y |
| P51674 | Neuronal membrane glycoprotein M6-a OS=Homo sapiens GN=GPM6A PE=1 SV=2 | 31188 | 5.0024 | 1.009 | B | Y |
| P51970 | NADH dehydrogenase [ubiquinone] 1 alpha subcomplex subunit 8 OS=Homo sapiens GN=NDU | 20092 | 7.5483 | 0.296 | B | Y |
| P51991 | Heterogeneous nuclear ribonucleoprotein A3 OS=Homo sapiens GN=HNRNPA3 PE=1 SV=2 | 39570 | 9.2212 | 0.934 | B | Y |
| P52209 | 6-phosphogluconate dehydrogenase, decarboxylating OS=Homo sapiens GN=PGD PE=1 SV=3 | 53105 | 6.8584 | 0.146 | B | Y |
| P52597 | Heterogeneous nuclear ribonucleoprotein F OS=Homo sapiens GN=HNRNPF PE=1 SV=3 | 45642 | 5.2427 | 0.042 | B | Y |
| P52758 | Ribonuclease UK114 OS=Homo sapiens GN=HRSP12 PE=1 SV=1 | 14484 | 9.1289 | 0.223 | B | Y |
| P52907 | F-actin-capping protein subunit alpha-1 OS=Homo sapiens GN=CAPZA1 PE=1 SV=3 | 32902 | 5.3262 | 0.038 | B | Y |
| P53007 | Tricarboxylate transport protein, mitochondrial OS=Homo sapiens GN=SLC25A1 PE=1 SV=2 | 33991 | 10.2231 | 0.141 | B | Y |
| P53396 | ATP-citrate synthase OS=Homo sapiens GN=ACLY PE=1 SV=3 | 120762 | 6.9507 | 0.518 | B | Y |
| P53597 | Succinyl-CoA ligase [ADP/GDP-forming] subunit alpha, mitochondrial OS=Homo sapiens GN=S | 36226 | 8.978 | 0.133 | B | Y |
| P53673 | Beta-crystallin A4 OS=Homo sapiens GN=CRYBA4 PE=1 SV=3 | 22359 | 5.8066 | 2.223 | B | Y |
| P53674 | Beta-crystallin B1 OS=Homo sapiens GN=CRYBB1 PE=1 SV=2 | 28005 | 8.9502 | 0.502 | B | Y |
| P53675 | Clathrin heavy chain 2 OS=Homo sapiens GN=CLTCL1 PE=1 SV=2 | 186909 | 5.4741 | 0.056 | B | Y |
| P54289 | Voltage-dependent calcium channel subunit alpha-2/delta-1 OS=Homo sapiens GN=CACNA2D | 124489 | 4.9365 | 0.838 | B | Y |
| P54652 | Heat shock-related 70 kDa protein 2 OS=Homo sapiens GN=HSPA2 PE=1 SV=1 | 69977 | 5.4082 | 2.025 | B | Y |
| P54707 | Potassium-transporting ATPase alpha chain 2 OS=Homo sapiens GN=ATP12A PE=1 SV=3 | 115437 | 6.0981 | 4.060 | B | Y |
| P54886 | Delta-1-pyrroline-5-carboxylate synthase OS=Homo sapiens GN=ALDH18A1 PE=1 SV=2 | 87247 | 6.6768 | 0.258 | B | Y |
| P54920 | Alpha-soluble NSF attachment protein OS=Homo sapiens GN=NAPA PE=1 SV=3 | 33211 | 5.064 | 0.249 | B | Y |
| P55072 | Transitional endoplasmic reticulum ATPase OS=Homo sapiens GN=VCP PE=1 SV=4 | 89265 | 4.9556 | 0.395 | B | Y |
| P55084 | Trifunctional enzyme subunit beta, mitochondrial OS=Homo sapiens GN=HADHB PE=1 SV=3 | 51261 | 9.75 | 0.344 | B | Y |
| P55087 | Aquaporin-4 OS=Homo sapiens GN=AQP4 PE=1 SV=2 | 34806 | 7.5557 | 0.001 | B | Y |
| P55786 | Puromycin-sensitive aminopeptidase OS=Homo sapiens GN=NPEPPS PE=1 SV=2 | 103210 | 5.3599 | 0.101 | B | Y |
| P55795 | Heterogeneous nuclear ribonucleoprotein H2 OS=Homo sapiens GN=HNRNPH2 PE=1 SV=1 | 49232 | 5.8521 | 0.293 | B | Y |
| P55809 | Succinyl-CoA:3-ketoacid coenzyme A transferase 1, mitochondrial OS=Homo sapiens GN=OXC | 56121 | 7.2202 | 0.292 | B | Y |

| | | | | | | |
|---|---|---|---|---|---|---|
| P56385 | ATP synthase subunit e, mitochondrial OS=Homo sapiens GN=ATP5I PE=1 SV=2 | 7928 | 9.7412 | 0.913 | B | Y |
| P57772 | Selenocysteine-specific elongation factor OS=Homo sapiens GN=EEFSEC PE=1 SV=4 | 65263 | 8.3481 | 0.094 | B | Y |
| P58546 | Myotrophin OS=Homo sapiens GN=MTPN PE=1 SV=2 | 12886 | 5.1343 | 0.226 | B | Y |
| P60174 | Triosephosphate isomerase OS=Homo sapiens GN=TPI1 PE=1 SV=3 | 30771 | 5.5474 | 2.330 | B | Y |
| P60201 | Myelin proteolipid protein OS=Homo sapiens GN=PLP1 PE=1 SV=2 | 30057 | 8.2881 | 2.664 | B | Y |
| P60602 | Reactive oxygen species modulator 1 OS=Homo sapiens GN=ROMO1 PE=1 SV=1 | 8176 | 9.7207 | 0.376 | B | Y |
| P60709 | Actin, cytoplasmic 1 OS=Homo sapiens GN=ACTB PE=1 SV=1 | 41709 | 5.1431 | 8.483 | B | Y |
| P60842 | Eukaryotic initiation factor 4A-I OS=Homo sapiens GN=EIF4A1 PE=1 SV=1 | 46124 | 5.1548 | 0.784 | B | Y |
| P60866 | 40S ribosomal protein S20 OS=Homo sapiens GN=RPS20 PE=1 SV=1 | 13364 | 10.4414 | 0.086 | B | Y |
| P60880 | Synaptosomal-associated protein 25 OS=Homo sapiens GN=SNAP25 PE=1 SV=1 | 23300 | 4.4575 | 1.338 | B | Y |
| P60953 | Cell division control protein 42 homolog OS=Homo sapiens GN=CDC42 PE=1 SV=2 | 21245 | 6.1538 | 0.549 | D | Y |
| P60983 | Glia maturation factor beta OS=Homo sapiens GN=GMFB PE=1 SV=2 | 16702 | 5.0098 | 0.221 | B | Y |
| P61006 | Ras-related protein Rab-8A OS=Homo sapiens GN=RAB8A PE=1 SV=1 | 23653 | 9.4321 | 0.297 | B | Y |
| P61019 | Ras-related protein Rab-2A OS=Homo sapiens GN=RAB2A PE=1 SV=1 | 23530 | 6.0806 | 0.613 | B | Y |
| P61020 | Ras-related protein Rab-5B OS=Homo sapiens GN=RAB5B PE=1 SV=1 | 23691 | 8.2383 | 0.416 | B | Y |
| P61026 | Ras-related protein Rab-10 OS=Homo sapiens GN=RAB10 PE=1 SV=1 | 22526 | 8.5737 | 0.539 | B | Y |
| P61088 | Ubiquitin-conjugating enzyme E2 N OS=Homo sapiens GN=UBE2N PE=1 SV=1 | 17126 | 6.1494 | 0.415 | B | Y |
| P61106 | Ras-related protein Rab-14 OS=Homo sapiens GN=RAB14 PE=1 SV=4 | 23881 | 5.7979 | 0.245 | B | Y |
| P61160 | Actin-related protein 2 OS=Homo sapiens GN=ACTR2 PE=1 SV=1 | 44732 | 6.2959 | 0.207 | B | Y |
| P61163 | Alpha-centractin OS=Homo sapiens GN=ACTR1A PE=1 SV=1 | 42586 | 6.1846 | 0.383 | B | Y |
| P61204 | ADP-ribosylation factor 3 OS=Homo sapiens GN=ARF3 PE=1 SV=2 | 20587 | 7.3887 | 0.001 | B | Y |
| P61224 | Ras-related protein Rap-1b OS=Homo sapiens GN=RAP1B PE=1 SV=1 | 20811 | 5.4653 | 0.001 | B | Y |
| P61266 | Syntaxin-1B OS=Homo sapiens GN=STX1B PE=1 SV=1 | 33223 | 5.0859 | 0.662 | B | Y |
| P61604 | 10 kDa heat shock protein, mitochondrial OS=Homo sapiens GN=HSPE1 PE=1 SV=2 | 10924 | 9.4702 | 0.805 | B | Y |
| P61764 | Syntaxin-binding protein 1 OS=Homo sapiens GN=STXBP1 PE=1 SV=1 | 67525 | 6.5098 | 0.963 | B | Y |
| P61978 | Heterogeneous nuclear ribonucleoprotein K OS=Homo sapiens GN=HNRNPK PE=1 SV=1 | 50944 | 5.2207 | 1.062 | B | Y |
| P61981 | 14-3-3 protein gamma OS=Homo sapiens GN=YWHAG PE=1 SV=2 | 28284 | 4.6069 | 2.698 | B | Y |
| P62081 | 40S ribosomal protein S7 OS=Homo sapiens GN=RPS7 PE=1 SV=1 | 22113 | 10.582 | 0.169 | B | Y |
| P62136 | Serine/threonine-protein phosphatase PP1-alpha catalytic subunit OS=Homo sapiens GN=PPP | 37487 | 5.8931 | 0.098 | B | Y |
| P62241 | 40S ribosomal protein S8 OS=Homo sapiens GN=RPS8 PE=1 SV=2 | 24190 | 10.7153 | 0.001 | B | Y |
| P62244 | 40S ribosomal protein S15a OS=Homo sapiens GN=RPS15A PE=1 SV=2 | 14829 | 10.604 | 0.206 | B | Y |
| P62258 | 14-3-3 protein epsilon OS=Homo sapiens GN=YWHAE PE=1 SV=1 | 29155 | 4.4355 | 6.095 | B | Y |
| P62269 | 40S ribosomal protein S18 OS=Homo sapiens GN=RPS18 PE=1 SV=3 | 17707 | 11.4141 | 0.263 | B | Y |
| P62304 | Small nuclear ribonucleoprotein E OS=Homo sapiens GN=SNRPE PE=1 SV=1 | 10796 | 9.7808 | 0.174 | B | Y |
| P62424 | 60S ribosomal protein L7a OS=Homo sapiens GN=RPL7A PE=1 SV=2 | 29977 | 11.0581 | 0.001 | B | Y |
| P62701 | 40S ribosomal protein S4, X isoform OS=Homo sapiens GN=RPS4X PE=1 SV=2 | 29579 | 10.5864 | 0.302 | B | Y |
| P62736 | Actin, aortic smooth muscle OS=Homo sapiens GN=ACTA2 PE=1 SV=1 | 41981 | 5.0771 | 0.001 | B | Y |
| P62753 | 40S ribosomal protein S6 OS=Homo sapiens GN=RPS6 PE=1 SV=1 | 28663 | 11.2896 | 0.920 | B | Y |
| P62805 | Histone H4 OS=Homo sapiens GN=HIST1H4A PE=1 SV=2 | 11360 | 11.7671 | 3.550 | B | Y |
| P62820 | Ras-related protein Rab-1A OS=Homo sapiens GN=RAB1A PE=1 SV=3 | 22663 | 5.8491 | 0.583 | B | Y |
| P62851 | 40S ribosomal protein S25 OS=Homo sapiens GN=RPS25 PE=1 SV=1 | 13733 | 10.5762 | 0.206 | B | Y |
| P62873 | Guanine nucleotide-binding protein G(I)/G(S)/G(T) subunit beta-1 OS=Homo sapiens GN=GNB | 37353 | 5.5356 | 1.722 | B | Y |
| P62879 | Guanine nucleotide-binding protein G(I)/G(S)/G(T) subunit beta-2 OS=Homo sapiens GN=GNB | 37307 | 5.5356 | 0.179 | B | Y |
| P62906 | 60S ribosomal protein L10a OS=Homo sapiens GN=RPL10A PE=1 SV=2 | 24815 | 10.3564 | 0.123 | B | Y |
| P62937 | Peptidyl-prolyl cis-trans isomerase A OS=Homo sapiens GN=PPIA PE=1 SV=2 | 18000 | 7.853 | 1.787 | B | Y |
| P62942 | Peptidyl-prolyl cis-trans isomerase FKBP1A OS=Homo sapiens GN=FKBP1A PE=1 SV=2 | 11943 | 8.6558 | 0.244 | B | Y |
| P62993 | Growth factor receptor-bound protein 2 OS=Homo sapiens GN=GRB2 PE=1 SV=1 | 25190 | 5.8579 | 0.190 | B | Y |
| P63000 | Ras-related C3 botulinum toxin substrate 1 OS=Homo sapiens GN=RAC1 PE=1 SV=1 | 21436 | 8.5723 | 0.191 | B | Y |
| P63010 | AP-2 complex subunit beta OS=Homo sapiens GN=AP2B1 PE=1 SV=1 | 104486 | 5.0552 | 0.393 | B | Y |
| P63096 | Guanine nucleotide-binding protein G(i) subunit alpha-1 OS=Homo sapiens GN=GNAI1 PE=1 S | 40335 | 5.5913 | 0.134 | B | Y |
| P63104 | 14-3-3 protein zeta/delta OS=Homo sapiens GN=YWHAZ PE=1 SV=1 | 27727 | 4.5278 | 4.524 | B | Y |
| P63151 | Serine/threonine-protein phosphatase 2A 55 kDa regulatory subunit B alpha isoform OS=Homo | 51659 | 5.7642 | 0.048 | B | Y |
| P63267 | Actin, gamma-enteric smooth muscle OS=Homo sapiens GN=ACTG2 PE=1 SV=1 | 41849 | 5.1606 | 0.026 | B | Y |
| P67775 | Serine/threonine-protein phosphatase 2A catalytic subunit alpha isoform OS=Homo sapiens GN | 35571 | 5.168 | 0.168 | B | Y |
| P67936 | Tropomyosin alpha-4 chain OS=Homo sapiens GN=TPM4 PE=1 SV=3 | 28504 | 4.4707 | 0.620 | B | Y |
| P68032 | Actin, alpha cardiac muscle 1 OS=Homo sapiens GN=ACTC1 PE=1 SV=1 | 41991 | 5.0713 | 1.086 | B | Y |
| P68104 | Elongation factor 1-alpha 1 OS=Homo sapiens GN=EEF1A1 PE=1 SV=1 | 50109 | 9.3428 | 1.416 | B | Y |
| P68363 | Tubulin alpha-1B chain OS=Homo sapiens GN=TUBA1B PE=1 SV=1 | 50119 | 4.7622 | 0.365 | B | Y |
| P68366 | Tubulin alpha-4A chain OS=Homo sapiens GN=TUBA4A PE=1 SV=1 | 49892 | 4.752 | 5.176 | B | Y |
| P68371 | Tubulin beta-4B chain OS=Homo sapiens GN=TUBB4B PE=1 SV=1 | 49799 | 4.6025 | 3.897 | B | Y |
| P68431 | Histone H3.1 OS=Homo sapiens GN=HIST1H3A PE=1 SV=2 | 15394 | 11.5415 | 0.619 | B | Y |
| P68871 | Hemoglobin subunit beta OS=Homo sapiens GN=HBB PE=1 SV=2 | 15988 | 6.8804 | 8.794 | B | Y |
| P69905 | Hemoglobin subunit alpha OS=Homo sapiens GN=HBA1 PE=1 SV=2 | 15247 | 9.1787 | 8.419 | B | Y |
| P78324 | Tyrosine-protein phosphatase non-receptor type substrate 1 OS=Homo sapiens GN=SIRPA PE | 54932 | 6.5361 | 0.065 | B | Y |
| P78357 | Contactin-associated protein 1 OS=Homo sapiens GN=CNTNAP1 PE=1 SV=1 | 156166 | 6.5991 | 0.059 | B | Y |
| P78371 | T-complex protein 1 subunit beta OS=Homo sapiens GN=CCT2 PE=1 SV=4 | 57452 | 5.9912 | 0.830 | B | Y |
| P78527 | DNA-dependent protein kinase catalytic subunit OS=Homo sapiens GN=PRKDC PE=1 SV=3 | 468786 | 6.7075 | 0.831 | B | Y |
| P78559 | Microtubule-associated protein 1A OS=Homo sapiens GN=MAP1A PE=1 SV=6 | 305296 | 4.6567 | 0.659 | B | Y |
| P80723 | Brain acid soluble protein 1 OS=Homo sapiens GN=BASP1 PE=1 SV=2 | 22680 | 4.4238 | 0.404 | B | Y |
| P84074 | Neuron-specific calcium-binding protein hippocalcin OS=Homo sapiens GN=HPCA PE=1 SV=2 | 22412 | 4.6758 | 0.804 | B | Y |
| P84077 | ADP-ribosylation factor 1 OS=Homo sapiens GN=ARF1 PE=1 SV=2 | 20683 | 6.375 | 0.786 | B | Y |
| Q00005 | Serine/threonine-protein phosphatase 2A 55 kDa regulatory subunit B beta isoform OS=Homo s | 51677 | 5.9824 | 0.041 | B | Y |
| Q00325 | Phosphate carrier protein, mitochondrial OS=Homo sapiens GN=SLC25A3 PE=1 SV=2 | 40068 | 9.6387 | 1.719 | B | Y |
| Q00577 | Transcriptional activator protein Pur-alpha OS=Homo sapiens GN=PURA PE=1 SV=2 | 34889 | 6.0249 | 0.709 | B | Y |
| Q00839 | Heterogeneous nuclear ribonucleoprotein U OS=Homo sapiens GN=HNRNPU PE=1 SV=6 | 90527 | 5.6484 | 1.123 | B | Y |
| Q01082 | Spectrin beta chain, non-erythrocytic 1 OS=Homo sapiens GN=SPTBN1 PE=1 SV=2 | 274437 | 5.2515 | 0.617 | B | Y |
| Q01469 | Fatty acid-binding protein, epidermal OS=Homo sapiens GN=FABP5 PE=1 SV=3 | 15154 | 6.8042 | 0.540 | B | Y |
| Q01484 | Ankyrin-2 OS=Homo sapiens GN=ANK2 PE=1 SV=4 | 433447 | 4.8516 | 0.310 | B | Y |
| Q01518 | Adenylyl cyclase-associated protein 1 OS=Homo sapiens GN=CAP1 PE=1 SV=5 | 51868 | 8.0581 | 0.436 | B | Y |
| Q01813 | ATP-dependent 6-phosphofructokinase, platelet type OS=Homo sapiens GN=PFKP PE=1 SV=2 | 85541 | 7.3535 | 0.350 | B | Y |
| Q01814 | Plasma membrane calcium-transporting ATPase 2 OS=Homo sapiens GN=ATP2B2 PE=1 SV=2 | 136789 | 5.5474 | 0.031 | B | Y |

| | | | | | | |
|---|---|---|---|---|---|---|
| Q02252 | Methylmalonate-semialdehyde dehydrogenase [acylating], mitochondrial OS=Homo sapiens GN | 57802 | 8.584 | 0.338 | B | Y |
| Q02338 | D-beta-hydroxybutyrate dehydrogenase, mitochondrial OS=Homo sapiens GN=BDH1 PE=1 SV= | 38132 | 9.1523 | 0.075 | B | Y |
| Q02750 | Dual specificity mitogen-activated protein kinase kinase 1 OS=Homo sapiens GN=MAP2K1 PE= | 43411 | 6.1772 | 0.330 | B | Y |
| Q02790 | Peptidyl-prolyl cis-trans isomerase FKBP4 OS=Homo sapiens GN=FKBP4 PE=1 SV=3 | 51772 | 5.1812 | 0.106 | B | Y |
| Q02818 | Nucleobindin-1 OS=Homo sapiens GN=NUCB1 PE=1 SV=4 | 53846 | 4.9805 | 0.155 | B | Y |
| Q02878 | 60S ribosomal protein L6 OS=Homo sapiens GN=RPL6 PE=1 SV=3 | 32707 | 11.0244 | 0.185 | B | Y |
| Q02978 | Mitochondrial 2-oxoglutarate/malate carrier protein OS=Homo sapiens GN=SLC25A11 PE=1 SV | 34039 | 10.2085 | 0.810 | B | Y |
| Q04695 | Keratin, type I cytoskeletal 17 OS=Homo sapiens GN=KRT17 PE=1 SV=2 | 48076 | 4.7769 | 4.094 | B | Y |
| Q04760 | Lactoylglutathione lyase OS=Homo sapiens GN=GLO1 PE=1 SV=4 | 20764 | 4.9424 | 0.374 | B | Y |
| Q04837 | Single-stranded DNA-binding protein, mitochondrial OS=Homo sapiens GN=SSBP1 PE=1 SV=1 | 17249 | 9.9111 | 0.230 | B | Y |
| Q04917 | 14-3-3 protein eta OS=Homo sapiens GN=YWHAH PE=1 SV=4 | 28201 | 4.5615 | 1.332 | B | Y |
| Q05193 | Dynamin-1 OS=Homo sapiens GN=DNM1 PE=1 SV=2 | 97347 | 6.7588 | 0.040 | B | Y |
| Q05639 | Elongation factor 1-alpha 2 OS=Homo sapiens GN=EEF1A2 PE=1 SV=1 | 50438 | 9.3472 | 1.238 | B | Y |
| Q06210 | Glutamine--fructose-6-phosphate aminotransferase [isomerizing] 1 OS=Homo sapiens GN=GFF | 78756 | 6.6694 | 0.341 | B | Y |
| Q06828 | Fibromodulin OS=Homo sapiens GN=FMOD PE=1 SV=2 | 43151 | 5.6045 | 0.480 | B | Y |
| Q06830 | Peroxiredoxin-1 OS=Homo sapiens GN=PRDX1 PE=1 SV=1 | 22096 | 8.2427 | 2.692 | B | Y |
| Q07021 | Complement component 1 Q subcomponent-binding protein, mitochondrial OS=Homo sapiens ( | 31342 | 4.5469 | 0.654 | B | Y |
| Q07065 | Cytoskeleton-associated protein 4 OS=Homo sapiens GN=CKAP4 PE=1 SV=2 | 65982 | 5.5415 | 0.251 | B | Y |
| Q07666 | KH domain-containing, RNA-binding, signal transduction-associated protein 1 OS=Homo sapien | 48197 | 8.9136 | 0.100 | B | Y |
| Q07960 | Rho GTPase-activating protein 1 OS=Homo sapiens GN=ARHGAP1 PE=1 SV=1 | 50404 | 5.8198 | 0.070 | B | Y |
| Q08209 | Serine/threonine-protein phosphatase 2B catalytic subunit alpha isoform OS=Homo sapiens GN | 58650 | 5.4814 | 0.596 | B | Y |
| Q08211 | ATP-dependent RNA helicase A OS=Homo sapiens GN=DHX9 PE=1 SV=4 | 140868 | 6.3955 | 0.488 | B | Y |
| Q08378 | Golgin subfamily A member 3 OS=Homo sapiens GN=GOLGA3 PE=1 SV=2 | 167251 | 5.1724 | 1.165 | B | Y |
| Q08495 | Dematin OS=Homo sapiens GN=DMTN PE=1 SV=3 | 45486 | 9.2549 | 0.082 | B | Y |
| Q08722 | Leukocyte surface antigen CD47 OS=Homo sapiens GN=CD47 PE=1 SV=1 | 35190 | 6.9844 | 0.376 | B | Y |
| Q10567 | AP-1 complex subunit beta-1 OS=Homo sapiens GN=AP1B1 PE=1 SV=2 | 104570 | 4.749 | 0.170 | B | Y |
| Q12765 | Secernin-1 OS=Homo sapiens GN=SCRN1 PE=1 SV=2 | 46352 | 4.4707 | 2.069 | B | Y |
| Q12860 | Contactin-1 OS=Homo sapiens GN=CNTN1 PE=1 SV=1 | 113249 | 5.5137 | 0.714 | B | Y |
| Q12906 | Interleukin enhancer-binding factor 3 OS=Homo sapiens GN=ILF3 PE=1 SV=3 | 95279 | 8.9561 | 0.185 | B | Y |
| Q12931 | Heat shock protein 75 kDa, mitochondrial OS=Homo sapiens GN=TRAP1 PE=1 SV=3 | 80059 | 8.2852 | 0.109 | B | Y |
| Q12955 | Ankyrin-3 OS=Homo sapiens GN=ANK3 PE=1 SV=3 | 480112 | 6.0396 | 0.194 | B | Y |
| Q13011 | Delta(3,5)-Delta(2,4)-dienoyl-CoA isomerase, mitochondrial OS=Homo sapiens GN=ECH1 PE= | 35793 | 7.9995 | 0.501 | B | Y |
| Q13151 | Heterogeneous nuclear ribonucleoprotein A0 OS=Homo sapiens GN=HNRNPA0 PE=1 SV=1 | 30821 | 9.5493 | 0.570 | B | Y |
| Q13177 | Serine/threonine-protein kinase PAK 2 OS=Homo sapiens GN=PAK2 PE=1 SV=3 | 58005 | 5.5913 | 0.298 | B | Y |
| Q13228 | Selenium-binding protein 1 OS=Homo sapiens GN=SELENBP1 PE=1 SV=2 | 52357 | 5.9063 | 0.182 | B | Y |
| Q13263 | Transcription intermediary factor 1-beta OS=Homo sapiens GN=TRIM28 PE=1 SV=5 | 88493 | 5.4126 | 0.588 | B | Y |
| Q13308 | Inactive tyrosine-protein kinase 7 OS=Homo sapiens GN=PTK7 PE=1 SV=2 | 118316 | 6.6606 | 0.235 | B | Y |
| Q13332 | Receptor-type tyrosine-protein phosphatase S OS=Homo sapiens GN=PTPRS PE=1 SV=3 | 216903 | 6.0176 | 0.612 | B | Y |
| Q13423 | NAD(P) transhydrogenase, mitochondrial OS=Homo sapiens GN=NNT PE=1 SV=3 | 113822 | 8.0566 | 0.646 | B | Y |
| Q13492 | Phosphatidylinositol-binding clathrin assembly protein OS=Homo sapiens GN=PICALM PE=1 S' | 70710 | 7.9556 | 0.476 | B | Y |
| Q13509 | Tubulin beta-3 chain OS=Homo sapiens GN=TUBB3 PE=1 SV=2 | 50400 | 4.6392 | 1.821 | B | Y |
| Q13554 | Calcium/calmodulin-dependent protein kinase type II subunit beta OS=Homo sapiens GN=CAMI | 72632 | 6.8818 | 0.717 | B | Y |
| Q13555 | Calcium/calmodulin-dependent protein kinase type II subunit gamma OS=Homo sapiens GN=C/ | 62569 | 7.6714 | 0.039 | B | Y |
| Q13561 | Dynactin subunit 2 OS=Homo sapiens GN=DCTN2 PE=1 SV=4 | 44203 | 4.9248 | 0.125 | B | Y |
| Q13748 | Tubulin alpha-3C/D chain OS=Homo sapiens GN=TUBA3C PE=1 SV=3 | 49927 | 4.7974 | 0.022 | B | Y |
| Q13825 | Methylglutaconyl-CoA hydratase, mitochondrial OS=Homo sapiens GN=AUH PE=1 SV=1 | 35586 | 9.8687 | 0.185 | B | Y |
| Q13838 | Spliceosome RNA helicase DDX39B OS=Homo sapiens GN=DDX39B PE=1 SV=1 | 48960 | 5.313 | 0.290 | B | Y |
| Q13885 | Tubulin beta-2A chain OS=Homo sapiens GN=TUBB2A PE=1 SV=1 | 49874 | 4.5908 | 3.787 | B | Y |
| Q14011 | Cold-inducible RNA-binding protein OS=Homo sapiens GN=CIRBP PE=1 SV=1 | 18636 | 9.7397 | 0.488 | B | Y |
| Q14019 | Coactosin-like protein OS=Homo sapiens GN=COTL1 PE=1 SV=3 | 15935 | 5.3408 | 0.421 | B | Y |
| Q14108 | Lysosome membrane protein 2 OS=Homo sapiens GN=SCARB2 PE=1 SV=2 | 54255 | 4.8179 | 0.071 | B | Y |
| Q14152 | Eukaryotic translation initiation factor 3 subunit A OS=Homo sapiens GN=EIF3A PE=1 SV=1 | 166467 | 6.3428 | 0.932 | B | Y |
| Q14194 | Dihydropyrimidinase-related protein 1 OS=Homo sapiens GN=CRMP1 PE=1 SV=1 | 62144 | 6.5801 | 0.727 | B | Y |
| Q14195 | Dihydropyrimidinase-related protein 3 OS=Homo sapiens GN=DPYSL3 PE=1 SV=1 | 61924 | 6.0249 | 0.766 | B | Y |
| Q14240 | Eukaryotic initiation factor 4A-II OS=Homo sapiens GN=EIF4A2 PE=1 SV=2 | 46372 | 5.1636 | 0.635 | B | Y |
| Q14247 | Src substrate cortactin OS=Homo sapiens GN=CTTN PE=1 SV=2 | 61548 | 5.0771 | 0.057 | B | Y |
| Q14257 | Reticulocalbin-2 OS=Homo sapiens GN=RCN2 PE=1 SV=1 | 36853 | 4.0635 | 0.213 | B | Y |
| Q14344 | Guanine nucleotide-binding protein subunit alpha-13 OS=Homo sapiens GN=GNA13 PE=1 SV= | 44021 | 7.9966 | 0.058 | B | Y |
| Q14568 | Heat shock protein HSP 90-alpha A2 OS=Homo sapiens GN=HSP90AA2P PE=1 SV=2 | 39340 | 4.377 | 0.144 | B | Y |
| Q14894 | Ketimine reductase mu-crystallin OS=Homo sapiens GN=CRYM PE=1 SV=1 | 33754 | 4.8765 | 1.551 | B | Y |
| Q14914 | Prostaglandin reductase 1 OS=Homo sapiens GN=PTGR1 PE=1 SV=2 | 35846 | 8.4712 | 0.260 | B | Y |
| Q14CZ8 | Hepatocyte cell adhesion molecule OS=Homo sapiens GN=HEPACAM PE=1 SV=1 | 45998 | 9.3999 | 0.329 | B | Y |
| Q15019 | Septin-2 OS=Homo sapiens GN=SEPT2 PE=1 SV=1 | 41461 | 6.1392 | 0.474 | B | Y |
| Q15029 | 116 kDa U5 small nuclear ribonucleoprotein component OS=Homo sapiens GN=EFTUD2 PE=1 | 109366 | 4.6582 | 0.024 | B | Y |
| Q15056 | Eukaryotic translation initiation factor 4H OS=Homo sapiens GN=EIF4H PE=1 SV=5 | 27368 | 7.2026 | 0.240 | B | Y |
| Q15084 | Protein disulfide-isomerase A6 OS=Homo sapiens GN=PDIA6 PE=1 SV=1 | 48091 | 4.7622 | 0.409 | B | Y |
| Q15121 | Astrocytic phosphoprotein PEA-15 OS=Homo sapiens GN=PEA15 PE=1 SV=2 | 15030 | 4.7388 | 0.361 | B | Y |
| Q15233 | Non-POU domain-containing octamer-binding protein OS=Homo sapiens GN=NONO PE=1 SV= | 54197 | 9.3691 | 0.171 | B | Y |
| Q15286 | Ras-related protein Rab-35 OS=Homo sapiens GN=RAB35 PE=1 SV=1 | 23010 | 8.3936 | 0.696 | B | Y |
| Q15365 | Poly(rC)-binding protein 1 OS=Homo sapiens GN=PCBP1 PE=1 SV=2 | 37473 | 6.7148 | 1.093 | B | Y |
| Q15435 | Protein phosphatase 1 regulatory subunit 7 OS=Homo sapiens GN=PPP1R7 PE=1 SV=1 | 41538 | 4.6479 | 0.156 | B | Y |
| Q15555 | Microtubule-associated protein RP/EB family member 2 OS=Homo sapiens GN=MAPRE2 PE=1 | 37008 | 5.2324 | 0.208 | B | Y |
| Q15717 | ELAV-like protein 1 OS=Homo sapiens GN=ELAVL1 PE=1 SV=2 | 36069 | 9.4702 | 0.182 | B | Y |
| Q15907 | Ras-related protein Rab-11B OS=Homo sapiens GN=RAB11B PE=1 SV=4 | 24473 | 5.5474 | 0.850 | B | Y |
| Q16143 | Beta-synuclein OS=Homo sapiens GN=SNCB PE=1 SV=1 | 14279 | 4.21 | 0.751 | B | Y |
| Q16352 | Alpha-internexin OS=Homo sapiens GN=INA PE=1 SV=2 | 55357 | 5.1694 | 0.879 | B | Y |
| Q16555 | Dihydropyrimidinase-related protein 2 OS=Homo sapiens GN=DPYSL2 PE=1 SV=1 | 62254 | 5.9238 | 2.227 | B | Y |
| Q16643 | Drebrin OS=Homo sapiens GN=DBN1 PE=1 SV=4 | 71385 | 4.2026 | 0.343 | B | Y |
| Q16658 | Fascin OS=Homo sapiens GN=FSCN1 PE=1 SV=3 | 54496 | 6.876 | 1.710 | B | Y |
| Q16698 | 2,4-dienoyl-CoA reductase, mitochondrial OS=Homo sapiens GN=DECR1 PE=1 SV=1 | 36044 | 9.6606 | 0.153 | B | Y |
| Q16795 | NADH dehydrogenase [ubiquinone] 1 alpha subcomplex subunit 9, mitochondrial OS=Homo sap | 42482 | 10.1558 | 0.182 | B | Y |

| | | | | | | |
|---|---|---|---|---|---|---|
| Q16799 | Reticulon-1 OS=Homo sapiens GN=RTN1 PE=1 SV=1 | 83566 | 4.415 | 0.672 | B | Y |
| Q16864 | V-type proton ATPase subunit F OS=Homo sapiens GN=ATP6V1F PE=1 SV=2 | 13361 | 5.1504 | 0.151 | B | Y |
| Q2TAY7 | WD40 repeat-containing protein SMU1 OS=Homo sapiens GN=SMU1 PE=1 SV=2 | 57507 | 6.7705 | 0.303 | B | Y |
| Q3SXM5 | Inactive hydroxysteroid dehydrogenase-like protein 1 OS=Homo sapiens GN=HSDL1 PE=1 SV= | 36978 | 8.8315 | 0.051 | B | Y |
| Q4G0N4 | NAD kinase 2, mitochondrial OS=Homo sapiens GN=NADK2 PE=1 SV=2 | 49402 | 8.1211 | 0.070 | B | Y |
| Q56VL3 | OCIA domain-containing protein 2 OS=Homo sapiens GN=OCIAD2 PE=1 SV=1 | 16942 | 9.3193 | 0.111 | B | Y |
| Q58FF7 | Putative heat shock protein HSP 90-beta-3 OS=Homo sapiens GN=HSP90AB3P PE=5 SV=1 | 68282 | 4.5132 | 0.134 | B | Y |
| Q58FF8 | Putative heat shock protein HSP 90-beta 2 OS=Homo sapiens GN=HSP90AB2P PE=1 SV=2 | 44321 | 4.5894 | 0.071 | B | Y |
| Q5HY64 | Putative protein FAM47C OS=Homo sapiens GN=FAM47C PE=2 SV=1 | 115266 | 6.7441 | 5.812 | B | Y |
| Q5JWF2 | Guanine nucleotide-binding protein G(s) subunit alpha isoforms XLas OS=Homo sapiens GN=G | 110955 | 4.7256 | 0.188 | B | Y |
| Q5JXI8 | Four and a half LIM domains protein 1 (Fragment) OS=Homo sapiens GN=FHL1 PE=1 SV=1 | 29140 | 8.1519 | 0.257 | B | Y |
| Q5JXJ0 | Mitochondrial ribosome-associated GTPase 2 (Fragment) OS=Homo sapiens GN=MTG2 PE=1 | 24669 | 10.3726 | 0.153 | B | Y |
| Q5SRE5 | Nucleoporin NUP188 homolog OS=Homo sapiens GN=NUP188 PE=1 SV=1 | 195916 | 6.2607 | 0.074 | B | Y |
| Q5SSJ5 | Heterochromatin protein 1-binding protein 3 OS=Homo sapiens GN=HP1BP3 PE=1 SV=1 | 61169 | 10.1016 | 0.164 | B | Y |
| Q5TC82 | Roquin-1 OS=Homo sapiens GN=RC3H1 PE=1 SV=1 | 125657 | 6.8643 | 1.641 | B | Y |
| Q5TEC6 | Histone H3 OS=Homo sapiens GN=HIST2H3PS2 PE=1 SV=1 | 15420 | 11.6807 | 0.045 | B | Y |
| Q5TZA2 | Rootletin OS=Homo sapiens GN=CROCC PE=1 SV=1 | 228385 | 5.2778 | 0.151 | B | Y |
| Q5VT06 | Centrosome-associated protein 350 OS=Homo sapiens GN=CEP350 PE=1 SV=1 | 350714 | 5.8989 | 0.581 | B | Y |
| Q5VTR2 | E3 ubiquitin-protein ligase BRE1A OS=Homo sapiens GN=RNF20 PE=1 SV=2 | 113592 | 5.6104 | 0.473 | B | Y |
| Q5XKE5 | Keratin, type II cytoskeletal 79 OS=Homo sapiens GN=KRT79 PE=1 SV=2 | 57800 | 6.8262 | 0.095 | B | Y |
| Q68D91 | Metallo-beta-lactamase domain-containing protein 2 OS=Homo sapiens GN=MBLAC2 PE=1 SV | 31351 | 6.4468 | 0.062 | B | Y |
| Q6DT37 | Serine/threonine-protein kinase MRCK gamma OS=Homo sapiens GN=CDC42BPG PE=1 SV=2 | 172351 | 5.855 | 0.914 | B | Y |
| Q6PCE3 | Glucose 1,6-bisphosphate synthase OS=Homo sapiens GN=PGM2L1 PE=1 SV=3 | 70396 | 6.8042 | 0.280 | B | Y |
| Q6S8J3 | POTE ankyrin domain family member E OS=Homo sapiens GN=POTEE PE=1 SV=3 | 121285 | 5.7715 | 0.076 | B | Y |
| Q6ZQQ6 | WD repeat-containing protein 87 OS=Homo sapiens GN=WDR87 PE=1 SV=3 | 332973 | 6.9009 | 0.480 | B | Y |
| Q6ZSZ5 | Rho guanine nucleotide exchange factor 18 OS=Homo sapiens GN=ARHGEF18 PE=1 SV=3 | 130700 | 6.6504 | 0.044 | B | Y |
| Q709C8 | Vacuolar protein sorting-associated protein 13C OS=Homo sapiens GN=VPS13C PE=1 SV=1 | 422122 | 6.3647 | 0.214 | B | Y |
| Q71DI3 | Histone H3.2 OS=Homo sapiens GN=HIST2H3A PE=1 SV=3 | 15378 | 11.6807 | 0.329 | B | Y |
| Q71U36 | Tubulin alpha-1A chain OS=Homo sapiens GN=TUBA1A PE=1 SV=1 | 50103 | 4.7622 | 14.880 | B | Y |
| Q7KZF4 | Staphylococcal nuclease domain-containing protein 1 OS=Homo sapiens GN=SND1 PE=1 SV= | 101933 | 6.75 | 0.593 | B | Y |
| Q7L099 | Protein RUFY3 OS=Homo sapiens GN=RUFY3 PE=1 SV=1 | 52931 | 5.2002 | 0.123 | B | Y |
| Q7L0J3 | Synaptic vesicle glycoprotein 2A OS=Homo sapiens GN=SV2A PE=1 SV=1 | 82642 | 5.2354 | 0.252 | B | Y |
| Q7L273 | BTB/POZ domain-containing protein KCTD9 OS=Homo sapiens GN=KCTD9 PE=1 SV=1 | 42539 | 5.9209 | 0.609 | B | Y |
| Q7Z406 | Myosin-14 OS=Homo sapiens GN=MYH14 PE=1 SV=2 | 227730 | 5.3584 | 0.080 | B | Y |
| Q86UY8 | 5'-nucleotidase domain-containing protein 3 OS=Homo sapiens GN=NT5DC3 PE=1 SV=1 | 63379 | 8.3628 | 0.057 | B | Y |
| Q86VP6 | Cullin-associated NEDD8-dissociated protein 1 OS=Homo sapiens GN=CAND1 PE=1 SV=2 | 136288 | 5.4067 | 0.191 | B | Y |
| Q86W61 | VCAN protein OS=Homo sapiens GN=VCAN PE=1 SV=1 | 39239 | 6.7412 | 2.988 | B | Y |
| Q86XW9 | Thioredoxin domain-containing protein 6 OS=Homo sapiens GN=NME9 PE=2 SV=1 | 36832 | 4.623 | 2.732 | B | Y |
| Q86Y39 | NADH dehydrogenase [ubiquinone] 1 alpha subcomplex subunit 11 OS=Homo sapiens GN=ND | 14842 | 8.9077 | 0.178 | B | Y |
| Q86Y46 | Keratin, type II cytoskeletal 73 OS=Homo sapiens GN=KRT73 PE=1 SV=1 | 58886 | 6.9932 | 0.030 | B | Y |
| Q8IUG5 | Unconventional myosin-XVIIIb OS=Homo sapiens GN=MYO18B PE=1 SV=1 | 285006 | 6.4585 | 2.102 | B | Y |
| Q8IVE3 | Pleckstrin homology domain-containing family H member 2 OS=Homo sapiens GN=PLEKHH2 F | 168123 | 7.3184 | 0.027 | B | Y |
| Q8IVL0 | Neuron navigator 3 OS=Homo sapiens GN=NAV3 PE=1 SV=3 | 255488 | 8.9487 | 0.557 | B | Y |
| Q8IX04 | Ubiquitin-conjugating enzyme E2 variant 3 OS=Homo sapiens GN=UEVLD PE=1 SV=2 | 52231 | 6.6577 | 0.322 | B | Y |
| Q8IXQ9 | Protein N-lysine methyltransferase METTL20 OS=Homo sapiens GN=METTL20 PE=1 SV=1 | 29441 | 6.0542 | 3.111 | B | Y |
| Q8N126 | Cell adhesion molecule 3 OS=Homo sapiens GN=CADM3 PE=1 SV=1 | 43272 | 5.6572 | 0.179 | B | Y |
| Q8N1G4 | Leucine-rich repeat-containing protein 47 OS=Homo sapiens GN=LRRC47 PE=1 SV=1 | 63433 | 8.272 | 0.177 | B | Y |
| Q8N3J6 | Cell adhesion molecule 2 OS=Homo sapiens GN=CADM2 PE=2 SV=1 | 47524 | 4.9922 | 0.119 | B | Y |
| Q8N6M0 | OTU domain-containing protein 6B OS=Homo sapiens GN=OTUD6B PE=1 SV=1 | 33791 | 5.6851 | 0.050 | B | Y |
| Q8N6N7 | Acyl-CoA-binding domain-containing protein 7 OS=Homo sapiens GN=ACBD7 PE=1 SV=1 | 9784 | 6.7588 | 0.141 | B | Y |
| Q8NCB2 | CaM kinase-like vesicle-associated protein OS=Homo sapiens GN=CAMKV PE=2 SV=2 | 54320 | 5.2251 | 0.107 | B | Y |
| Q8TAA3 | Proteasome subunit alpha type-7-like OS=Homo sapiens GN=PSMA8 PE=2 SV=3 | 28512 | 9.3354 | 0.127 | B | Y |
| Q8TB36 | Ganglioside-induced differentiation-associated protein 1 OS=Homo sapiens GN=GDAP1 PE=1 S | 41319 | 8.4858 | 0.192 | B | Y |
| Q8TDB8 | Solute carrier family 2, facilitated glucose transporter member 14 OS=Homo sapiens GN=SLC2/ | 56283 | 7.7358 | 0.236 | B | Y |
| Q8TDM6 | Disks large homolog 5 OS=Homo sapiens GN=DLG5 PE=1 SV=4 | 213733 | 7.0547 | 0.229 | B | Y |
| Q8WXF1 | Paraspeckle component 1 OS=Homo sapiens GN=PSPC1 PE=1 SV=1 | 58706 | 6.2344 | 0.159 | B | Y |
| Q8WXF7 | Atlastin-1 OS=Homo sapiens GN=ATL1 PE=1 SV=1 | 63503 | 5.7568 | 0.081 | B | Y |
| Q8WY54 | Protein phosphatase 1E OS=Homo sapiens GN=PPM1E PE=1 SV=2 | 84948 | 4.7666 | 0.143 | B | Y |
| Q92561 | Phytanoyl-CoA hydroxylase-interacting protein OS=Homo sapiens GN=PHYHIP PE=1 SV=1 | 37548 | 6.5566 | 0.182 | B | Y |
| Q92598 | Heat shock protein 105 kDa OS=Homo sapiens GN=HSPH1 PE=1 SV=1 | 96803 | 5.0991 | 0.200 | B | Y |
| Q92599 | Septin-8 OS=Homo sapiens GN=SEPT8 PE=1 SV=4 | 55721 | 5.8418 | 0.632 | B | Y |
| Q92747 | Actin-related protein 2/3 complex subunit 1A OS=Homo sapiens GN=ARPC1A PE=1 SV=2 | 41542 | 8.1343 | 0.103 | B | Y |
| Q92752 | Tenascin-R OS=Homo sapiens GN=TNR PE=1 SV=3 | 149467 | 4.522 | 0.824 | B | Y |
| Q92777 | Synapsin-2 OS=Homo sapiens GN=SYN2 PE=2 SV=3 | 62807 | 8.5474 | 0.743 | B | Y |
| Q93050 | V-type proton ATPase 116 kDa subunit a isoform 1 OS=Homo sapiens GN=ATP6V0A1 PE=1 S | 96350 | 5.981 | 0.381 | B | Y |
| Q93084 | Sarcoplasmic/endoplasmic reticulum calcium ATPase 3 OS=Homo sapiens GN=ATP2A3 PE=1 | 113904 | 5.2632 | 2.780 | B | Y |
| Q969P0 | Immunoglobulin superfamily member 8 OS=Homo sapiens GN=IGSF8 PE=1 SV=1 | 64993 | 7.8999 | 0.098 | B | Y |
| Q96A08 | Histone H2B type 1-A OS=Homo sapiens GN=HIST1H2BA PE=1 SV=3 | 14158 | 10.7402 | 1.313 | B | Y |
| Q96A23 | Copine-4 OS=Homo sapiens GN=CPNE4 PE=1 SV=1 | 62355 | 5.8799 | 0.257 | B | Y |
| Q96AE4 | Far upstream element-binding protein 1 OS=Homo sapiens GN=FUBP1 PE=1 SV=3 | 67518 | 7.4268 | 0.098 | B | Y |
| Q96AY3 | Peptidyl-prolyl cis-trans isomerase FKBP10 OS=Homo sapiens GN=FKBP10 PE=1 SV=1 | 64204 | 5.2397 | 0.086 | B | Y |
| Q96CN7 | Isochorismatase domain-containing protein 1 OS=Homo sapiens GN=ISOC1 PE=1 SV=3 | 32216 | 7.1719 | 0.541 | B | Y |
| Q96D15 | Reticulocalbin-3 OS=Homo sapiens GN=RCN3 PE=1 SV=1 | 37470 | 4.5571 | 0.114 | B | Y |
| Q96E17 | Ras-related protein Rab-3C OS=Homo sapiens GN=RAB3C PE=2 SV=1 | 25935 | 4.9028 | 0.659 | B | Y |
| Q96F85 | CB1 cannabinoid receptor-interacting protein 1 OS=Homo sapiens GN=CNRIP1 PE=1 SV=1 | 18636 | 8.1768 | 0.345 | B | Y |
| Q96FJ2 | Dynein light chain 2, cytoplasmic OS=Homo sapiens GN=DYNLL2 PE=1 SV=1 | 10343 | 7.2422 | 0.252 | B | Y |
| Q96GW7 | Brevican core protein OS=Homo sapiens GN=BCAN PE=1 SV=2 | 99056 | 4.377 | 0.481 | B | Y |
| Q96HN2 | Adenosylhomocysteinase 3 OS=Homo sapiens GN=AHCYL2 PE=1 SV=1 | 66678 | 7.0811 | 0.063 | B | Y |
| Q96IX5 | Up-regulated during skeletal muscle growth protein 5 OS=Homo sapiens GN=USMG5 PE=1 SV | 6453 | 10.0664 | 0.441 | B | Y |
| Q96JE9 | Microtubule-associated protein 6 OS=Homo sapiens GN=MAP6 PE=1 SV=2 | 86451 | 9.5786 | 0.085 | B | Y |
| Q96KN7 | X-linked retinitis pigmentosa GTPase regulator-interacting protein 1 OS=Homo sapiens GN=RP | 146589 | 5.3481 | 2.910 | B | Y |

| | | | | | | |
|---|---|---|---|---|---|---|
| Q96KP4 | Cytosolic non-specific dipeptidase OS=Homo sapiens GN=CNDP2 PE=1 SV=2 | 52844 | 5.5679 | 0.287 | B | Y |
| Q96L93 | Kinesin-like protein KIF16B OS=Homo sapiens GN=KIF16B PE=1 SV=2 | 151916 | 5.7788 | 0.885 | B | Y |
| Q96PK6 | RNA-binding protein 14 OS=Homo sapiens GN=RBM14 PE=1 SV=2 | 69448 | 9.8042 | 0.878 | B | Y |
| Q96QB1 | Rho GTPase-activating protein 7 OS=Homo sapiens GN=DLC1 PE=1 SV=4 | 170484 | 5.9458 | 6.299 | B | Y |
| Q96QK1 | Vacuolar protein sorting-associated protein 35 OS=Homo sapiens GN=VPS35 PE=1 SV=2 | 91649 | 5.168 | 0.096 | B | Y |
| Q99456 | Keratin, type I cytoskeletal 12 OS=Homo sapiens GN=KRT12 PE=1 SV=1 | 53478 | 4.5073 | 0.682 | B | Y |
| Q99497 | Protein deglycase DJ-1 OS=Homo sapiens GN=PARK7 PE=1 SV=2 | 19878 | 6.3721 | 1.114 | B | Y |
| Q99536 | Synaptic vesicle membrane protein VAT-1 homolog OS=Homo sapiens GN=VAT1 PE=1 SV=2 | 41893 | 5.8506 | 0.145 | B | Y |
| Q99584 | Protein S100-A13 OS=Homo sapiens GN=S100A13 PE=1 SV=1 | 11464 | 5.8257 | 0.254 | B | Y |
| Q99714 | 3-hydroxyacyl-CoA dehydrogenase type-2 OS=Homo sapiens GN=HSD17B10 PE=1 SV=3 | 26906 | 7.8384 | 1.066 | B | Y |
| Q99719 | Septin-5 OS=Homo sapiens GN=SEPT5 PE=1 SV=1 | 42749 | 6.2065 | 0.309 | B | Y |
| Q99747 | Gamma-soluble NSF attachment protein OS=Homo sapiens GN=NAPG PE=1 SV=1 | 34724 | 5.1299 | 0.153 | B | Y |
| Q99798 | Aconitate hydratase, mitochondrial OS=Homo sapiens GN=ACO2 PE=1 SV=2 | 85371 | 7.3286 | 1.056 | B | Y |
| Q99832 | T-complex protein 1 subunit eta OS=Homo sapiens GN=CCT7 PE=1 SV=2 | 59328 | 7.519 | 0.824 | B | Y |
| Q99962 | Endophilin-A1 OS=Homo sapiens GN=SH3GL2 PE=1 SV=1 | 39937 | 5.1636 | 0.413 | B | Y |
| Q9BPU6 | Dihydropyrimidinase-related protein 5 OS=Homo sapiens GN=DPYSL5 PE=1 SV=1 | 61382 | 6.7559 | 0.863 | B | Y |
| Q9BPW8 | Protein NipSnap homolog 1 OS=Homo sapiens GN=NIPSNAP1 PE=1 SV=1 | 33288 | 9.5581 | 0.316 | B | Y |
| Q9BPX5 | Actin-related protein 2/3 complex subunit 5-like protein OS=Homo sapiens GN=ARPC5L PE=1 S | 16930 | 6.1729 | 0.344 | B | Y |
| Q9BQE3 | Tubulin alpha-1C chain OS=Homo sapiens GN=TUBA1C PE=1 SV=1 | 49863 | 4.7856 | 1.609 | B | Y |
| Q9BR76 | Coronin-1B OS=Homo sapiens GN=CORO1B PE=1 SV=1 | 54200 | 5.5034 | 0.070 | B | Y |
| Q9BRA2 | Thioredoxin domain-containing protein 17 OS=Homo sapiens GN=TXNDC17 PE=1 SV=1 | 13931 | 5.231 | 0.108 | B | Y |
| Q9BUF5 | Tubulin beta-6 chain OS=Homo sapiens GN=TUBB6 PE=1 SV=1 | 49825 | 4.5791 | 1.193 | B | Y |
| Q9BVA1 | Tubulin beta-2B chain OS=Homo sapiens GN=TUBB2B PE=1 SV=1 | 49920 | 4.5908 | 1.171 | B | Y |
| Q9BVK6 | Transmembrane emp24 domain-containing protein 9 OS=Homo sapiens GN=TMED9 PE=1 SV= | 27260 | 8.1973 | 0.243 | B | Y |
| Q9BW30 | Tubulin polymerization-promoting protein family member 3 OS=Homo sapiens GN=TPPP3 PE= | 18973 | 9.5288 | 0.379 | B | Y |
| Q9BWD1 | Acetyl-CoA acetyltransferase, cytosolic OS=Homo sapiens GN=ACAT2 PE=1 SV=2 | 41324 | 6.4878 | 1.120 | B | Y |
| Q9BYK8 | Helicase with zinc finger domain 2 OS=Homo sapiens GN=HELZ2 PE=1 SV=6 | 294462 | 7.1616 | 0.065 | B | Y |
| Q9BYX7 | Putative beta-actin-like protein 3 OS=Homo sapiens GN=POTEKP PE=5 SV=1 | 41988 | 5.8843 | 6.683 | B | Y |
| Q9H0E2 | Toll-interacting protein OS=Homo sapiens GN=TOLLIP PE=1 SV=1 | 30262 | 5.584 | 0.043 | B | Y |
| Q9H0N0 | Ras-related protein Rab-6C OS=Homo sapiens GN=RAB6C PE=1 SV=2 | 28337 | 7.8032 | 0.136 | B | Y |
| Q9H115 | Beta-soluble NSF attachment protein OS=Homo sapiens GN=NAPB PE=1 SV=2 | 33535 | 5.1694 | 0.298 | B | Y |
| Q9H254 | Spectrin beta chain, non-erythrocytic 4 OS=Homo sapiens GN=SPTBN4 PE=1 SV=2 | 288806 | 5.6323 | 0.134 | B | Y |
| Q9H4B7 | Tubulin beta-1 chain OS=Homo sapiens GN=TUBB1 PE=1 SV=1 | 50294 | 4.8823 | 0.233 | B | Y |
| Q9H6R4 | Nucleolar protein 6 OS=Homo sapiens GN=NOL6 PE=1 SV=2 | 127513 | 7.3477 | 5.566 | B | Y |
| Q9H8Y8 | Golgi reassembly-stacking protein 2 OS=Homo sapiens GN=GORASP2 PE=1 SV=3 | 47116 | 4.5337 | 0.074 | B | Y |
| Q9H9B4 | Sideroflexin-1 OS=Homo sapiens GN=SFXN1 PE=1 SV=4 | 35596 | 9.3457 | 0.451 | B | Y |
| Q9HAV0 | Guanine nucleotide-binding protein subunit beta-4 OS=Homo sapiens GN=GNB4 PE=1 SV=3 | 37543 | 5.5283 | 0.696 | B | Y |
| Q9HCC0 | Methylcrotonoyl-CoA carboxylase beta chain, mitochondrial OS=Homo sapiens GN=MCCC2 PE | 61294 | 7.5293 | 0.064 | B | Y |
| Q9NP72 | Ras-related protein Rab-18 OS=Homo sapiens GN=RAB18 PE=1 SV=1 | 22962 | 4.9263 | 0.073 | B | Y |
| Q9NPJ3 | Acyl-coenzyme A thioesterase 13 OS=Homo sapiens GN=ACOT13 PE=1 SV=1 | 14950 | 9.6343 | 0.126 | B | Y |
| Q9NQ66 | 1-phosphatidylinositol 4,5-bisphosphate phosphodiesterase beta-1 OS=Homo sapiens GN=PLC | 138479 | 5.8257 | 0.067 | B | Y |
| Q9NQC3 | Reticulon-4 OS=Homo sapiens GN=RTN4 PE=1 SV=2 | 129851 | 4.2246 | 0.143 | B | Y |
| Q9NR31 | GTP-binding protein SAR1a OS=Homo sapiens GN=SAR1A PE=1 SV=1 | 22352 | 6.2344 | 0.363 | B | Y |
| Q9NRC6 | Spectrin beta chain, non-erythrocytic 5 OS=Homo sapiens GN=SPTBN5 PE=1 SV=2 | 416491 | 6.1904 | 0.106 | B | Y |
| Q9NRW1 | Ras-related protein Rab-6B OS=Homo sapiens GN=RAB6B PE=1 SV=1 | 23446 | 5.2163 | 0.116 | B | Y |
| Q9NS69 | Mitochondrial import receptor subunit TOM22 homolog OS=Homo sapiens GN=TOMM22 PE=1 | 15511 | 4.0649 | 0.295 | B | Y |
| Q9NSB2 | Keratin, type II cuticular Hb4 OS=Homo sapiens GN=KRT84 PE=2 SV=2 | 64801 | 7.4209 | 0.058 | B | Y |
| Q9NSD9 | Phenylalanine--tRNA ligase beta subunit OS=Homo sapiens GN=FARSB PE=1 SV=3 | 66073 | 6.3955 | 0.643 | B | Y |
| Q9NSE4 | Isoleucine--tRNA ligase, mitochondrial OS=Homo sapiens GN=IARS2 PE=1 SV=2 | 113719 | 6.7852 | 0.380 | B | Y |
| Q9NUJ1 | Mycophenolic acid acyl-glucuronide esterase, mitochondrial OS=Homo sapiens GN=ABHD10 P | 33910 | 8.7056 | 0.234 | B | Y |
| Q9NVJ2 | ADP-ribosylation factor-like protein 8B OS=Homo sapiens GN=ARL8B PE=1 SV=1 | 21525 | 8.6045 | 0.271 | B | Y |
| Q9NY65 | Tubulin alpha-8 chain OS=Homo sapiens GN=TUBA8 PE=1 SV=1 | 50061 | 4.7578 | 0.366 | B | Y |
| Q9NZ45 | CDGSH iron-sulfur domain-containing protein 1 OS=Homo sapiens GN=CISD1 PE=1 SV=1 | 12191 | 9.4468 | 0.503 | B | Y |
| Q9P035 | Very-long-chain (3R)-3-hydroxyacyl-CoA dehydratase 3 OS=Homo sapiens GN=HACD3 PE=1 S | 43131 | 9.1714 | 0.268 | B | Y |
| Q9P0K7 | Ankycorbin OS=Homo sapiens GN=RAI14 PE=1 SV=2 | 109972 | 5.8052 | 0.314 | B | Y |
| Q9P258 | Protein RCC2 OS=Homo sapiens GN=RCC2 PE=1 SV=2 | 56049 | 8.8857 | 0.642 | B | Y |
| Q9P2R3 | Rabankyrin-5 OS=Homo sapiens GN=ANKFY1 PE=1 SV=2 | 128318 | 5.6572 | 0.060 | B | Y |
| Q9P2R7 | Succinyl-CoA ligase [ADP-forming] subunit beta, mitochondrial OS=Homo sapiens GN=SUCLA2 | 50285 | 7.2041 | 0.317 | B | Y |
| Q9UBB6 | Neurochondrin OS=Homo sapiens GN=NCDN PE=1 SV=1 | 78813 | 5.1812 | 0.552 | B | Y |
| Q9UBI6 | Guanine nucleotide-binding protein G(I)/G(S)/G(O) subunit gamma-12 OS=Homo sapiens GN=( | 8001 | 9.4761 | 0.072 | B | Y |
| Q9UBQ7 | Glyoxylate reductase/hydroxypyruvate reductase OS=Homo sapiens GN=GRHPR PE=1 SV=1 | 35645 | 7.1411 | 0.089 | B | Y |
| Q9UBV8 | Peflin OS=Homo sapiens GN=PEF1 PE=1 SV=1 | 30361 | 6.1099 | 0.204 | B | Y |
| Q9UDW1 | Cytochrome b-c1 complex subunit 9 OS=Homo sapiens GN=UQCR10 PE=1 SV=3 | 7303 | 9.8643 | 0.240 | B | Y |
| Q9UFM8 | Neuroplastin (Fragment) OS=Homo sapiens GN=DKFZp566H1924 PE=1 SV=2 | 34793 | 7.6582 | 0.237 | B | Y |
| Q9UFN0 | Protein NipSnap homolog 3A OS=Homo sapiens GN=NIPSNAP3A PE=1 SV=2 | 28448 | 9.4556 | 0.104 | B | Y |
| Q9UH03 | Neuronal-specific septin-3 OS=Homo sapiens GN=SEPT3 PE=1 SV=3 | 40678 | 6.8218 | 0.430 | B | Y |
| Q9UHD8 | Septin-9 OS=Homo sapiens GN=SEPT9 PE=1 SV=2 | 65360 | 9.312 | 0.230 | B | Y |
| Q9UHG2 | ProSAAS OS=Homo sapiens GN=PCSK1N PE=1 SV=1 | 27355 | 6.2227 | 0.228 | B | Y |
| Q9UHG3 | Prenylcysteine oxidase 1 OS=Homo sapiens GN=PCYOX1 PE=1 SV=3 | 56603 | 5.748 | 0.696 | B | Y |
| Q9UI12 | V-type proton ATPase subunit H OS=Homo sapiens GN=ATP6V1H PE=1 SV=1 | 55847 | 6.0396 | 0.245 | B | Y |
| Q9UI15 | Transgelin-3 OS=Homo sapiens GN=TAGLN3 PE=1 SV=2 | 22458 | 7.1704 | 0.037 | B | Y |
| Q9UIJ7 | GTP:AMP phosphotransferase AK3, mitochondrial OS=Homo sapiens GN=AK3 PE=1 SV=4 | 25549 | 9.5303 | 0.171 | B | Y |
| Q9UJC5 | SH3 domain-binding glutamic acid-rich-like protein 2 OS=Homo sapiens GN=SH3BGRL2 PE=1 | 12318 | 6.8013 | 0.168 | B | Y |
| Q9UJS0 | Calcium-binding mitochondrial carrier protein Aralar2 OS=Homo sapiens GN=SLC25A13 PE=1 | 74128 | 8.77 | 0.077 | B | Y |
| Q9UJW0 | Dynactin subunit 4 OS=Homo sapiens GN=DCTN4 PE=1 SV=1 | 52303 | 7.0679 | 0.123 | B | Y |
| Q9UJZ1 | Stomatin-like protein 2, mitochondrial OS=Homo sapiens GN=STOML2 PE=1 SV=1 | 38510 | 7.2642 | 0.504 | B | Y |
| Q9UK22 | F-box only protein 2 OS=Homo sapiens GN=FBXO2 PE=1 SV=2 | 33306 | 4.0957 | 0.168 | B | Y |
| Q9UK61 | Protein TASOR OS=Homo sapiens GN=FAM208A PE=1 SV=3 | 188912 | 5.439 | 0.060 | B | Y |
| Q9ULV4 | Coronin-1C OS=Homo sapiens GN=CORO1C PE=1 SV=1 | 53215 | 6.6724 | 0.593 | B | Y |
| Q9UM22 | Mammalian ependymin-related protein 1 OS=Homo sapiens GN=EPDR1 PE=1 SV=2 | 25420 | 6.4189 | 0.087 | B | Y |
| Q9UMF0 | Intercellular adhesion molecule 5 OS=Homo sapiens GN=ICAM5 PE=1 SV=3 | 97055 | 5.6016 | 0.163 | B | Y |

| | | | | | | |
|---|---|---|---|---|---|---|
| Q9UMX0 | Ubiquilin-1 OS=Homo sapiens GN=UBQLN1 PE=1 SV=2 | 62479 | 4.8413 | 0.179 | B | Y |
| Q9UNZ2 | NSFL1 cofactor p47 OS=Homo sapiens GN=NSFL1C PE=1 SV=2 | 40548 | 4.8076 | 0.106 | B | Y |
| Q9UPY8 | Microtubule-associated protein RP/EB family member 3 OS=Homo sapiens GN=MAPRE3 PE=1 | 31961 | 5.1958 | 0.112 | B | Y |
| Q9UQ80 | Proliferation-associated protein 2G4 OS=Homo sapiens GN=PA2G4 PE=1 SV=3 | 43759 | 6.1069 | 0.126 | B | Y |
| Q9UQM7 | Calcium/calmodulin-dependent protein kinase type II subunit alpha OS=Homo sapiens GN=CAM | 54053 | 6.6284 | 3.290 | B | Y |
| Q9Y266 | Nuclear migration protein nudC OS=Homo sapiens GN=NUDC PE=1 SV=1 | 38219 | 5.0947 | 0.041 | B | Y |
| Q9Y277 | Voltage-dependent anion-selective channel protein 3 OS=Homo sapiens GN=VDAC3 PE=1 SV= | 30639 | 8.8301 | 0.986 | B | Y |
| Q9Y285 | Phenylalanine--tRNA ligase alpha subunit OS=Homo sapiens GN=FARSA PE=1 SV=3 | 57527 | 7.7168 | 0.222 | B | Y |
| Q9Y2J8 | Protein-arginine deiminase type-2 OS=Homo sapiens GN=PADI2 PE=1 SV=2 | 75515 | 5.2588 | 0.190 | B | Y |
| Q9Y2Q0 | Phospholipid-transporting ATPase IA OS=Homo sapiens GN=ATP8A1 PE=1 SV=1 | 131285 | 6.394 | 0.125 | B | Y |
| Q9Y3F4 | Serine-threonine kinase receptor-associated protein OS=Homo sapiens GN=STRAP PE=1 SV= | 38413 | 4.8032 | 0.100 | B | Y |
| Q9Y3Q3 | Transmembrane emp24 domain-containing protein 3 OS=Homo sapiens GN=TMED3 PE=1 SV= | 24761 | 5.269 | 0.395 | B | Y |
| Q9Y4L1 | Hypoxia up-regulated protein 1 OS=Homo sapiens GN=HYOU1 PE=1 SV=1 | 111266 | 4.9717 | 0.244 | B | Y |
| Q9Y512 | Sorting and assembly machinery component 50 homolog OS=Homo sapiens GN=SAMM50 PE= | 51943 | 6.4585 | 0.150 | B | Y |
| Q9Y536 | Peptidyl-prolyl cis-trans isomerase A-like 4A OS=Homo sapiens GN=PPIAL4A PE=2 SV=1 | 18170 | 9.6167 | 0.057 | B | Y |
| Q9Y5L4 | Mitochondrial import inner membrane translocase subunit Tim13 OS=Homo sapiens GN=TIMM1 | 10492 | 8.2822 | 0.170 | B | Y |
| Q9Y5Z4 | Heme-binding protein 2 OS=Homo sapiens GN=HEBP2 PE=1 SV=1 | 22861 | 4.3491 | 0.105 | B | Y |
| Q9Y617 | Phosphoserine aminotransferase OS=Homo sapiens GN=PSAT1 PE=1 SV=2 | 40396 | 7.5879 | 0.342 | B | Y |
| Q9Y678 | Coatomer subunit gamma-1 OS=Homo sapiens GN=COPG1 PE=1 SV=1 | 97655 | 5.1665 | 0.231 | B | Y |
| Q9Y6B6 | GTP-binding protein SAR1b OS=Homo sapiens GN=SAR1B PE=1 SV=1 | 22395 | 5.6909 | 0.461 | B | Y |
| Q9Y6C9 | Mitochondrial carrier homolog 2 OS=Homo sapiens GN=MTCH2 PE=1 SV=1 | 33308 | 7.8926 | 0.302 | B | Y |
| Q9Y6R1 | Electrogenic sodium bicarbonate cotransporter 1 OS=Homo sapiens GN=SLC4A4 PE=1 SV=1 | 121382 | 6.3428 | 0.101 | B | Y |
| S4R435 | Protein RPS10-NUDT3 (Fragment) OS=Homo sapiens GN=RPS10-NUDT3 PE=3 SV=1 | 32575 | 9.479 | 0.019 | B | Y |
| A0A024QZX5 | Serpin B6 OS=Homo sapiens GN=SERPINB6 PE=1 SV=1 | 42996 | 5.0039 | 0.002 | D | Y |
| A0A024R216 | Hepatoma-derived growth factor, related protein 3, isoform CRA_a OS=Homo sapiens GN=HDC | 22606 | 8.4185 | 0.002 | D | Y |
| A0A024R3B9 | Alpha-crystallin B chain OS=Homo sapiens GN=CRYAB PE=1 SV=1 | 12245 | 9.2739 | 0.001 | D | Y |
| A0A075B6K8 | Ig lambda-1 chain C regions (Fragment) OS=Homo sapiens GN=IGLC1 PE=4 SV=1 | 11394 | 7.9878 | 0.001 | D | Y |
| A0A075B6N8 | Ig gamma-3 chain C region (Fragment) OS=Homo sapiens GN=IGHG3 PE=1 SV=1 | 41300 | 7.7886 | 0.001 | D | Y |
| A0A087WT59 | Transthyretin OS=Homo sapiens GN=TTR PE=1 SV=1 | 20132 | 5.0376 | 0.001 | D | Y |
| A0A087WTE4 | Neural cell adhesion molecule 1 OS=Homo sapiens GN=NCAM1 PE=1 SV=1 | 83690 | 4.5542 | 0.001 | D | Y |
| A0A087WTP3 | Far upstream element-binding protein 2 OS=Homo sapiens GN=KHSRP PE=1 SV=1 | 72982 | 7.5762 | 0.001 | D | Y |
| A0A087WUI2 | Heterogeneous nuclear ribonucleoproteins A2/B1 OS=Homo sapiens GN=HNRNPA2B1 PE=1 S | 29816 | 4.7183 | 1.010 | D | Y |
| A0A087WUK2 | Heterogeneous nuclear ribonucleoprotein D-like OS=Homo sapiens GN=HNRNPDL PE=1 SV=1 | 40015 | 10.355 | 0.278 | D | Y |
| A0A087WUZ3 | Spectrin beta chain, non-erythrocytic 1 OS=Homo sapiens GN=SPTBN1 PE=1 SV=1 | 274657 | 5.2515 | 0.001 | D | Y |
| A0A087WV01 | Elongation factor 1-alpha OS=Homo sapiens GN=EEF1A1 PE=1 SV=1 | 46330 | 7.5264 | 0.001 | D | Y |
| A0A087WV23 | SH3 domain-binding glutamic acid-rich-like protein 3 OS=Homo sapiens GN=SH3BGRL3 PE=1 | 23771 | 9.3442 | 0.001 | D | Y |
| A0A087WV47 | Ig gamma-1 chain C region OS=Homo sapiens GN=IGHG1 PE=1 SV=1 | 51121 | 7.3813 | 0.001 | D | Y |
| A0A087WV61 | Regulator of G-protein-signaling 22 OS=Homo sapiens GN=RGS22 PE=4 SV=1 | 145239 | 7.8062 | 0.001 | D | Y |
| A0A087WVQ6 | Clathrin heavy chain OS=Homo sapiens GN=CLTC PE=1 SV=1 | 191934 | 5.3555 | 0.001 | D | Y |
| A0A087WW96 | Synapsin-2 OS=Homo sapiens GN=SYN2 PE=1 SV=1 | 62956 | 8.5474 | 0.174 | D | Y |
| A0A087WWA3 | Kinesin-like protein OS=Homo sapiens GN=KIF1B PE=1 SV=1 | 201881 | 5.1387 | 0.250 | D | Y |
| A0A087WWB6 | Transgelin OS=Homo sapiens GN=TAGLN3 PE=1 SV=1 | 24994 | 8.5649 | 0.001 | D | Y |
| A0A087WWU8 | Tropomyosin alpha-3 chain OS=Homo sapiens GN=TPM3 PE=1 SV=1 | 26404 | 4.5513 | 0.273 | D | Y |
| A0A087WX08 | Gamma-adducin OS=Homo sapiens GN=ADD3 PE=1 SV=1 | 75328 | 6.2915 | 0.072 | D | Y |
| A0A087WXC5 | NADH dehydrogenase [ubiquinone] 1 alpha subcomplex subunit 10, mitochondrial OS=Homo sa | 40811 | 8.5957 | 0.001 | D | Y |
| A0A087WXS7 | ATPase ASNA1 OS=Homo sapiens GN=ASNA1 PE=1 SV=1 | 37094 | 4.8369 | 0.042 | D | Y |
| A0A087WY00 | Unconventional myosin-Va OS=Homo sapiens GN=MYO5A PE=1 SV=1 | 212065 | 8.7144 | 0.427 | D | Y |
| A0A087WY71 | AP-2 complex subunit mu OS=Homo sapiens GN=AP2M1 PE=1 SV=1 | 49495 | 9.8848 | 0.112 | D | Y |
| A0A087WYG8 | Alpha-internexin OS=Homo sapiens GN=INA PE=1 SV=1 | 55031 | 5.1665 | 0.174 | D | Y |
| A0A087WYS6 | Proteasome (Prosome, macropain) subunit, alpha type, 8, isoform CRA_b OS=Homo sapiens G | 25025 | 9.063 | 0.097 | D | Y |
| A0A087WYT3 | Prostaglandin E synthase 3 OS=Homo sapiens GN=PTGES3 PE=1 SV=1 | 19142 | 4.147 | 0.001 | D | Y |
| A0A087WZH7 | Myristoylated alanine-rich C-kinase substrate OS=Homo sapiens GN=MARCKS PE=1 SV=1 | 31577 | 4.5264 | 0.570 | D | Y |
| A0A087X027 | Protein SETSIP OS=Homo sapiens GN=SETSIP PE=3 SV=1 | 33624 | 4.0034 | 0.001 | D | Y |
| A0A087X054 | Hypoxia up-regulated protein 1 OS=Homo sapiens GN=HYOU1 PE=1 SV=1 | 104713 | 5.4243 | 0.067 | D | Y |
| A0A087X057 | Ganglioside-induced differentiation-associated protein 1-like 1 OS=Homo sapiens GN=GDAP1L | 41150 | 6.3867 | 0.081 | D | Y |
| A0A087X0X3 | Heterogeneous nuclear ribonucleoprotein M OS=Homo sapiens GN=HNRNPM PE=1 SV=1 | 77518 | 9.1436 | 0.001 | D | Y |
| A0A087X142 | Septin-8 OS=Homo sapiens GN=SEPT8 PE=1 SV=1 | 49325 | 5.7847 | 0.136 | D | Y |
| A0A087X253 | AP-2 complex subunit beta OS=Homo sapiens GN=AP2B1 PE=1 SV=1 | 101268 | 4.9644 | 0.508 | D | Y |
| A0A087X2B1 | RNA binding protein fox-1 homolog OS=Homo sapiens GN=RBFOX1 PE=4 SV=1 | 40284 | 6.624 | 0.001 | D | Y |
| A0A087X2G1 | ATP-dependent RNA helicase DDX1 OS=Homo sapiens GN=DDX1 PE=1 SV=1 | 73928 | 7.5747 | 0.001 | D | Y |
| A0A087X2H1 | E3 ubiquitin-protein ligase HECTD1 OS=Homo sapiens GN=HECTD1 PE=1 SV=1 | 289449 | 5.1064 | 0.001 | D | Y |
| A0A096LP12 | ES1 protein homolog, mitochondrial (Fragment) OS=Homo sapiens GN=LOC102724023 PE=4 S | 23849 | 9.4292 | 0.001 | D | Y |
| A0A0A0MRA8 | Band 4.1-like protein 3 OS=Homo sapiens GN=EPB41L3 PE=1 SV=1 | 102225 | 5.0361 | 1.299 | D | Y |
| A0A0A0MRF6 | A-kinase anchor protein 9 OS=Homo sapiens GN=AKAP9 PE=1 SV=1 | 453212 | 4.7534 | 0.243 | D | Y |
| A0A0A0MRJ6 | Protein-L-isoaspartate O-methyltransferase OS=Homo sapiens GN=PCMT1 PE=1 SV=1 | 30295 | 7.314 | 0.001 | D | Y |
| A0A0A0MS41 | Sideroflexin OS=Homo sapiens GN=SFXN3 PE=1 SV=1 | 35480 | 9.3223 | 0.071 | D | Y |
| A0A0A0MS51 | Gelsolin OS=Homo sapiens GN=GSN PE=1 SV=1 | 82474 | 5.2896 | 0.267 | D | Y |
| A0A0A0MS87 | Protein NDRG2 OS=Homo sapiens GN=NDRG2 PE=1 SV=1 | 39519 | 6.5845 | 0.001 | D | Y |
| A0A0A0MSE2 | Hydroxyacyl-coenzyme A dehydrogenase, mitochondrial OS=Homo sapiens GN=HADH PE=1 S | 42096 | 9.6475 | 0.001 | D | Y |
| A0A0A0MSI0 | Peroxiredoxin-1 (Fragment) OS=Homo sapiens GN=PRDX1 PE=1 SV=1 | 18963 | 6.4907 | 1.567 | D | Y |
| A0A0A0MT26 | Sodium/potassium-transporting ATPase subunit alpha-3 OS=Homo sapiens GN=ATP1A3 PE=1 | 133231 | 5.6865 | 0.001 | D | Y |
| A0A0A0MTI5 | Acyl-CoA-binding protein OS=Homo sapiens GN=DBI PE=1 SV=1 | 15948 | 4.7886 | 0.001 | D | Y |
| A0A0A0MTN3 | Glutathione S-transferase Mu 3 OS=Homo sapiens GN=GSTM3 PE=1 SV=1 | 24898 | 5.0156 | 0.210 | D | Y |
| A0A0A0MTR1 | Cadherin-13 OS=Homo sapiens GN=CDH13 PE=1 SV=2 | 76921 | 4.585 | 0.085 | D | Y |
| A0A0A0MTS2 | Glucose-6-phosphate isomerase (Fragment) OS=Homo sapiens GN=GPI PE=1 SV=1 | 64784 | 9.3516 | 0.001 | D | Y |
| A0A0A6YYA0 | Protein TMED7-TICAM2 OS=Homo sapiens GN=TMED7-TICAM2 PE=3 SV=1 | 21219 | 5.7759 | 0.122 | D | Y |
| A0A0B4J1R6 | Transketolase OS=Homo sapiens GN=TKT PE=1 SV=1 | 49878 | 7.8545 | 0.637 | D | Y |
| A0A0B4J2A2 | Peptidyl-prolyl cis-trans isomerase A-like 4C OS=Homo sapiens GN=PPIAL4C PE=2 SV=1 | 18144 | 9.6167 | 0.009 | D | Y |
| A0A0C4DFU1 | Superoxide dismutase OS=Homo sapiens GN=SOD2 PE=1 SV=1 | 20710 | 8.4067 | 0.001 | D | Y |
| A0A0C4DFU2 | Superoxide dismutase OS=Homo sapiens GN=SOD2 PE=1 SV=1 | 24734 | 8.4038 | 0.001 | D | Y |
| A0A0C4DGR7 | Liprin-alpha-3 (Fragment) OS=Homo sapiens GN=PPFIA3 PE=1 SV=1 | 31255 | 6.7178 | 0.001 | D | Y |

| | | | | | | |
|---|---|---|---|---|---|---|
| A0A0C4DGS0 | NADH dehydrogenase [ubiquinone] 1 alpha subcomplex subunit 6 OS=Homo sapiens GN=NDU | 15126 | 10.4033 | 0.163 | D | Y |
| A0A0C4DGZ9 | Tripeptidyl-peptidase 1 OS=Homo sapiens GN=TPP1 PE=1 SV=1 | 60420 | 5.9268 | 0.318 | D | Y |
| A0A0D9SF30 | Neural cell adhesion molecule 1 (Fragment) OS=Homo sapiens GN=NCAM1 PE=1 SV=1 | 47099 | 4.3975 | 0.104 | D | Y |
| A0A0D9SF51 | Phosphoinositide phospholipase C (Fragment) OS=Homo sapiens GN=PLCB1 PE=1 SV=1 | 108989 | 5.543 | 0.362 | D | Y |
| A0A0D9SF54 | Spectrin alpha chain, non-erythrocytic 1 OS=Homo sapiens GN=SPTAN1 PE=1 SV=1 | 282660 | 5.0493 | 1.243 | D | Y |
| A0A0D9SFB1 | Dynamin-1 OS=Homo sapiens GN=DNM1 PE=1 SV=1 | 93958 | 6.1934 | 0.570 | D | Y |
| A0A0D9SFF0 | Forkhead box protein M1 OS=Homo sapiens GN=FOXM1 PE=1 SV=1 | 82690 | 8.4199 | 6.330 | D | Y |
| A0A0D9SFL3 | RNA-binding protein EWS OS=Homo sapiens GN=EWSR1 PE=1 SV=1 | 61376 | 9.2607 | 0.001 | D | Y |
| A0A0D9SFS3 | 2-oxoglutarate dehydrogenase, mitochondrial OS=Homo sapiens GN=OGDH PE=1 SV=1 | 113241 | 6.627 | 0.214 | D | Y |
| A0A0D9SGJ6 | Synaptojanin-1 OS=Homo sapiens GN=SYNJ1 PE=1 SV=1 | 168052 | 6.832 | 0.294 | D | Y |
| A0A0G2JIW1 | Heat shock 70 kDa protein 1B OS=Homo sapiens GN=HSPA1B PE=1 SV=1 | 70066 | 5.3188 | 0.001 | D | Y |
| A0A0G2JLD8 | Single-stranded DNA-binding protein, mitochondrial (Fragment) OS=Homo sapiens GN=SSBP1 | 15588 | 10.2671 | 0.117 | D | Y |
| A0A0G2JMX7 | Microtubule-associated protein OS=Homo sapiens GN=MAPT PE=1 SV=1 | 80865 | 6.6577 | 0.001 | D | Y |
| A0A0G2JS82 | AP-2 complex subunit alpha-2 (Fragment) OS=Homo sapiens GN=AP2A2 PE=1 SV=1 | 101588 | 6.4189 | 0.001 | D | Y |
| A0A0J9YWK4 | Hemoglobin subunit beta OS=Homo sapiens GN=HBB PE=4 SV=1 | 5987 | 7.0122 | 10.146 | D | Y |
| A0A0J9YX62 | DnaJ homolog subfamily B member 6 OS=Homo sapiens GN=DNAJB6 PE=4 SV=1 | 36634 | 6.7852 | 0.001 | D | Y |
| A0A0J9YX66 | CUGBP Elav-like family member 2 OS=Homo sapiens GN=CELF2 PE=4 SV=1 | 54879 | 8.9297 | 0.001 | D | Y |
| A0A0J9YY01 | Unconventional myosin-XVB OS=Homo sapiens GN=MYO15B PE=4 SV=1 | 333513 | 7.481 | 2.560 | D | Y |
| A2A274 | Aconitate hydratase, mitochondrial OS=Homo sapiens GN=ACO2 PE=1 SV=1 | 87765 | 6.9858 | 0.001 | D | Y |
| A6NHL2 | Tubulin alpha chain-like 3 OS=Homo sapiens GN=TUBAL3 PE=1 SV=2 | 49876 | 5.6235 | 0.034 | D | Y |
| A6NMH8 | Tetraspanin OS=Homo sapiens GN=CD81 PE=1 SV=1 | 29786 | 6.5288 | 0.001 | D | Y |
| A8MXP9 | Matrin-3 OS=Homo sapiens GN=MATR3 PE=1 SV=1 | 99905 | 5.6309 | 0.001 | D | Y |
| B1AHR1 | Neuronal-specific septin-3 OS=Homo sapiens GN=SEPT3 PE=1 SV=1 | 33580 | 6.5142 | 0.001 | D | Y |
| B1AJQ6 | Syntaxin-12 (Fragment) OS=Homo sapiens GN=STX12 PE=1 SV=2 | 24561 | 4.8911 | 0.070 | D | Y |
| B1AK87 | Capping protein (Actin filament) muscle Z-line, beta, isoform CRA_a OS=Homo sapiens GN=CA | 29276 | 6.5098 | 0.206 | D | Y |
| B1AMS2 | Septin 6, isoform CRA_b OS=Homo sapiens GN=SEPT6 PE=1 SV=1 | 49272 | 6.3589 | 1.195 | D | Y |
| B1B1G2 | Myelin proteolipid protein (Fragment) OS=Homo sapiens GN=PLP1 PE=1 SV=6 | 12203 | 5.4258 | 1.161 | D | Y |
| B2R4S9 | Histone H2B OS=Homo sapiens GN=HIST1H2BC PE=2 SV=1 | 13897 | 10.7402 | 0.001 | D | Y |
| B2R5W2 | Heterogeneous nuclear ribonucleoproteins C1/C2 OS=Homo sapiens GN=HNRNPC PE=1 SV= | 31928 | 4.9087 | 0.184 | D | Y |
| B3KVD8 | Synaptoporin OS=Homo sapiens GN=SYNPR PE=1 SV=1 | 30508 | 6.1611 | 0.001 | D | Y |
| B4DDG0 | Tetraspanin OS=Homo sapiens GN=TSPAN7 PE=2 SV=1 | 29331 | 7.355 | 0.001 | D | Y |
| B4DE93 | NADH dehydrogenase [ubiquinone] flavoprotein 1, mitochondrial OS=Homo sapiens GN=NDUF | 39436 | 6.0103 | 0.217 | D | Y |
| B4DFG2 | WW domain-binding protein 2 OS=Homo sapiens GN=WBP2 PE=1 SV=1 | 23369 | 5.5415 | 0.187 | D | Y |
| B4DJ62 | HCG2002594, isoform CRA_a OS=Homo sapiens GN=SEPT5 PE=1 SV=1 | 37382 | 5.6616 | 0.001 | D | Y |
| B4DJV2 | Citrate synthase OS=Homo sapiens GN=CS PE=1 SV=1 | 50399 | 7.8604 | 0.303 | D | Y |
| B4DV12 | Polyubiquitin-B OS=Homo sapiens GN=UBB PE=1 SV=1 | 17204 | 7.3169 | 0.001 | D | Y |
| B4DY09 | Interleukin enhancer-binding factor 2 OS=Homo sapiens GN=ILF2 PE=1 SV=1 | 38886 | 4.6963 | 2.104 | D | Y |
| B4DYP1 | N(G),N(G)-dimethylarginine dimethylaminohydrolase 1 OS=Homo sapiens GN=DDAH1 PE=2 S | 20521 | 5.2456 | 0.558 | D | Y |
| B5MCD7 | Synaptogyrin-1 OS=Homo sapiens GN=SYNGR1 PE=1 SV=1 | 18453 | 6.8643 | 0.528 | D | Y |
| B5MCX3 | Septin-2 OS=Homo sapiens GN=SEPT2 PE=1 SV=1 | 36916 | 5.7451 | 0.001 | D | Y |
| B5MDF5 | GTP-binding nuclear protein Ran OS=Homo sapiens GN=RAN PE=1 SV=1 | 26207 | 6.5566 | 0.001 | D | Y |
| B7Z2R2 | Cytochrome b-c1 complex subunit 7 OS=Homo sapiens GN=UQCRB PE=1 SV=1 | 18725 | 9.3164 | 0.001 | D | Y |
| B7Z613 | Neuronal membrane glycoprotein M6-b OS=Homo sapiens GN=GPM6B PE=1 SV=1 | 33250 | 5.9575 | 0.814 | D | Y |
| B7ZAR1 | T-complex protein 1 subunit epsilon OS=Homo sapiens GN=CCT5 PE=1 SV=1 | 55313 | 5.1577 | 0.115 | D | Y |
| B7ZC38 | Endophilin-B2 OS=Homo sapiens GN=SH3GLB2 PE=1 SV=1 | 44333 | 5.4829 | 0.001 | D | Y |
| B8ZZ51 | Malate dehydrogenase, cytoplasmic OS=Homo sapiens GN=MDH1 PE=1 SV=1 | 18677 | 5.5532 | 0.610 | D | Y |
| B8ZZ54 | 10 kDa heat shock protein, mitochondrial OS=Homo sapiens GN=HSPE1 PE=1 SV=1 | 5186 | 4.2598 | 0.112 | D | Y |
| B8ZZB8 | CB1 cannabinoid receptor-interacting protein 1 OS=Homo sapiens GN=CNRIP1 PE=1 SV=1 | 15158 | 9.7544 | 0.639 | D | Y |
| B9A041 | Malate dehydrogenase, cytoplasmic OS=Homo sapiens GN=MDH1 PE=1 SV=1 | 23023 | 7.2305 | 1.721 | D | Y |
| B9A067 | MICOS complex subunit MIC60 OS=Homo sapiens GN=IMMT PE=1 SV=2 | 78925 | 6.6357 | 0.001 | D | Y |
| C9J0J7 | Profilin-2 OS=Homo sapiens GN=PFN2 PE=1 SV=1 | 9834 | 9.5332 | 0.001 | D | Y |
| C9J8Q5 | Succinate-semialdehyde dehydrogenase, mitochondrial OS=Homo sapiens GN=ALDH5A1 PE= | 53833 | 8.127 | 0.212 | D | Y |
| C9J9C1 | Serine/threonine-protein phosphatase 2A 65 kDa regulatory subunit A alpha isoform (Fragment) | 43168 | 5.0684 | 0.124 | D | Y |
| C9J9E2 | CaM kinase-like vesicle-associated protein OS=Homo sapiens GN=CAMKV PE=1 SV=1 | 49606 | 8.9795 | 0.001 | D | Y |
| C9JC84 | Fibrinogen gamma chain OS=Homo sapiens GN=FGG PE=1 SV=1 | 52304 | 5.2427 | 0.001 | D | Y |
| C9JFR7 | Cytochrome c (Fragment) OS=Homo sapiens GN=CYCS PE=1 SV=1 | 11325 | 10.0488 | 1.071 | D | Y |
| C9JIZ6 | Prosaposin OS=Homo sapiens GN=PSAP PE=1 SV=2 | 58402 | 4.8853 | 0.001 | D | Y |
| C9JJT5 | Protein ATP5J2-PTCD1 OS=Homo sapiens GN=ATP5J2-PTCD1 PE=4 SV=2 | 5911 | 9.7969 | 0.673 | D | Y |
| C9JNR4 | Transforming protein RhoA (Fragment) OS=Homo sapiens GN=RHOA PE=1 SV=1 | 14659 | 4.5674 | 0.100 | D | Y |
| C9JW96 | Prohibitin (Fragment) OS=Homo sapiens GN=PHB PE=1 SV=2 | 26875 | 5.0947 | 0.001 | D | Y |
| C9JY79 | Non-erythrocytic beta-spectrin 4 OS=Homo sapiens GN=SPTBN4 PE=1 SV=2 | 288882 | 5.6777 | 0.001 | D | Y |
| C9JYN0 | Synaptophysin-like protein 1 OS=Homo sapiens GN=SYPL1 PE=1 SV=1 | 24767 | 6.9199 | 0.160 | D | Y |
| C9JYY6 | Neuronal cell adhesion molecule OS=Homo sapiens GN=NRCAM PE=1 SV=3 | 133729 | 5.4082 | 0.243 | D | Y |
| C9K0J5 | Ras association (RalGDS/AF-6) and pleckstrin homology domains 1, isoform CRA_b OS=Homo | 141091 | 9.1523 | 3.218 | D | Y |
| D6R960 | Complexin-2 (Fragment) OS=Homo sapiens GN=CPLX2 PE=1 SV=1 | 13626 | 4.6948 | 0.001 | D | Y |
| D6R9Z7 | Cytochrome c oxidase subunit 7C, mitochondrial OS=Homo sapiens GN=COX7C PE=1 SV=1 | 6378 | 10.1997 | 0.326 | D | Y |
| D6RA31 | Alpha-synuclein (Fragment) OS=Homo sapiens GN=SNCA PE=1 SV=6 | 6815 | 9.8467 | 4.588 | D | Y |
| D6RA82 | Annexin OS=Homo sapiens GN=ANXA3 PE=1 SV=1 | 32098 | 5.5342 | 0.231 | D | Y |
| D6RAT0 | 40S ribosomal protein S3a OS=Homo sapiens GN=RPS3A PE=1 SV=1 | 25870 | 9.8423 | 0.001 | D | Y |
| D6RDU5 | Septin-11 (Fragment) OS=Homo sapiens GN=SEPT11 PE=1 SV=1 | 43085 | 5.8755 | 0.334 | D | Y |
| D6RE83 | Ubiquitin carboxyl-terminal hydrolase OS=Homo sapiens GN=UCHL1 PE=1 SV=1 | 23159 | 5.1812 | 3.128 | D | Y |
| D6RER5 | Septin-11 OS=Homo sapiens GN=SEPT11 PE=1 SV=1 | 49777 | 6.23 | 0.001 | D | Y |
| D6RF62 | Multifunctional protein ADE2 OS=Homo sapiens GN=PAICS PE=1 SV=1 | 37087 | 5.8638 | 0.057 | D | Y |
| D6RFH4 | Cytochrome b5 type B OS=Homo sapiens GN=CYB5B PE=1 SV=1 | 14836 | 5.7437 | 0.066 | D | Y |
| D6RJD1 | Clathrin light chain B (Fragment) OS=Homo sapiens GN=CLTB PE=1 SV=1 | 13070 | 3.999 | 0.256 | D | Y |
| E5KLJ5 | Dynamin-like 120 kDa protein, mitochondrial OS=Homo sapiens GN=OPA1 PE=1 SV=1 | 117669 | 7.5952 | 0.001 | D | Y |
| E5RGN3 | Copper transport protein ATOX1 OS=Homo sapiens GN=ATOX1 PE=1 SV=1 | 6326 | 7.9966 | 0.158 | D | Y |
| E5RGS4 | Prefoldin subunit 1 OS=Homo sapiens GN=PFDN1 PE=1 SV=1 | 13457 | 5.231 | 0.107 | D | Y |
| E5RHP7 | Carbonic anhydrase 1 (Fragment) OS=Homo sapiens GN=CA1 PE=1 SV=1 | 27736 | 6.1187 | 0.160 | D | Y |
| E5RI56 | Uncharacterized protein (Fragment) OS=Homo sapiens PE=1 SV=1 | 10264 | 4.0239 | 0.021 | D | Y |
| E5RJ29 | PH and SEC7 domain-containing protein 3 OS=Homo sapiens GN=PSD3 PE=1 SV=1 | 108923 | 5.6528 | 0.096 | D | Y |

| | | | | | | |
|---|---|---|---|---|---|---|
| E5RJR5 | S-phase kinase-associated protein 1 OS=Homo sapiens GN=SKP1 PE=1 SV=1 | 18708 | 4.3696 | 0.001 | D | Y |
| E5RJU9 | Protein LYRIC OS=Homo sapiens GN=MTDH PE=1 SV=1 | 57486 | 10.0298 | 0.040 | D | Y |
| E5RJZ1 | Cytochrome c oxidase subunit 7A-related protein, mitochondrial OS=Homo sapiens GN=COX7A | 8834 | 9.4189 | 0.206 | D | Y |
| E7EMB3 | Calmodulin OS=Homo sapiens GN=CALM2 PE=1 SV=1 | 21675 | 4.2524 | 0.001 | D | Y |
| E7EMV2 | Neurofilament medium polypeptide OS=Homo sapiens GN=NEFM PE=1 SV=1 | 78834 | 4.5688 | 0.545 | D | Y |
| E7EPK1 | Septin-7 OS=Homo sapiens GN=SEPT7 PE=1 SV=2 | 50662 | 8.8887 | 0.001 | D | Y |
| E7EPV7 | Alpha-synuclein OS=Homo sapiens GN=SNCA PE=1 SV=1 | 11769 | 9.7354 | 0.149 | D | Y |
| E7EQB2 | Lactotransferrin (Fragment) OS=Homo sapiens GN=LTF PE=1 SV=1 | 76576 | 7.894 | 0.383 | D | Y |
| E7ETC2 | Serine/threonine-protein phosphatase OS=Homo sapiens GN=PPP3CA PE=1 SV=1 | 47784 | 5.3804 | 0.313 | D | Y |
| E7EU96 | Casein kinase II subunit alpha OS=Homo sapiens GN=CSNK2A1 PE=1 SV=1 | 45281 | 7.9512 | 0.136 | D | Y |
| E7EWP0 | NADH dehydrogenase [ubiquinone] 1 beta subcomplex subunit 5, mitochondrial OS=Homo sapi | 20025 | 7.8311 | 0.001 | D | Y |
| E7EX29 | 14-3-3 protein zeta/delta (Fragment) OS=Homo sapiens GN=YWHAZ PE=1 SV=1 | 28018 | 4.6523 | 1.488 | D | Y |
| E9PCY7 | Heterogeneous nuclear ribonucleoprotein H OS=Homo sapiens GN=HNRNPH1 PE=1 SV=1 | 47057 | 5.896 | 0.223 | D | Y |
| E9PD68 | Dihydropyrimidinase-related protein 1 OS=Homo sapiens GN=CRMP1 PE=1 SV=1 | 61990 | 6.2578 | 0.511 | D | Y |
| E9PDE8 | Heat shock 70 kDa protein 4L OS=Homo sapiens GN=HSPA4L PE=1 SV=1 | 91895 | 5.6924 | 0.013 | D | Y |
| E9PDG8 | Clathrin coat assembly protein AP180 OS=Homo sapiens GN=SNAP91 PE=1 SV=1 | 91913 | 4.522 | 0.325 | D | Y |
| E9PEJ4 | Acetyltransferase component of pyruvate dehydrogenase complex OS=Homo sapiens GN=DLA | 57550 | 9.2051 | 0.001 | D | Y |
| E9PFP8 | Poly(rC)-binding protein 3 OS=Homo sapiens GN=PCBP3 PE=1 SV=1 | 38162 | 8.1123 | 0.001 | D | Y |
| E9PFT6 | Hemoglobin subunit delta OS=Homo sapiens GN=HBD PE=1 SV=1 | 15409 | 8.001 | 0.090 | D | Y |
| E9PG32 | Dynein heavy chain 12, axonemal OS=Homo sapiens GN=DNAH12 PE=4 SV=2 | 454033 | 5.4727 | 0.086 | D | Y |
| E9PH64 | NADH dehydrogenase [ubiquinone] 1 beta subcomplex subunit 9 OS=Homo sapiens GN=NDUF | 20370 | 7.9688 | 0.059 | D | Y |
| E9PHN6 | Glutathione S-transferase Mu 2 OS=Homo sapiens GN=GSTM2 PE=1 SV=1 | 22861 | 4.875 | 0.001 | D | Y |
| E9PHN7 | Glutathione S-transferase Mu 2 OS=Homo sapiens GN=GSTM2 PE=1 SV=2 | 21348 | 8.6001 | 0.142 | D | Y |
| E9PIA8 | Palmitoyl-protein thioesterase 1 (Fragment) OS=Homo sapiens GN=PPT1 PE=1 SV=7 | 22894 | 7.207 | 0.001 | D | Y |
| E9PII3 | Band 4.1-like protein 2 OS=Homo sapiens GN=EPB41L2 PE=1 SV=1 | 79208 | 6.3398 | 0.037 | D | Y |
| E9PJH7 | Mitochondrial glutamate carrier 1 (Fragment) OS=Homo sapiens GN=SLC25A22 PE=1 SV=5 | 33260 | 10.0063 | 0.591 | D | Y |
| E9PJL7 | Alpha-crystallin B chain (Fragment) OS=Homo sapiens GN=CRYAB PE=1 SV=6 | 15338 | 6.1362 | 0.001 | D | Y |
| E9PK25 | Cofilin-1 OS=Homo sapiens GN=CFL1 PE=1 SV=1 | 22713 | 8.5181 | 0.001 | D | Y |
| E9PKE3 | Heat shock cognate 71 kDa protein OS=Homo sapiens GN=HSPA8 PE=1 SV=1 | 68763 | 5.1973 | 0.001 | D | Y |
| E9PKL7 | Ras-related protein Rab-2A OS=Homo sapiens GN=RAB2A PE=1 SV=1 | 20765 | 10.2158 | 0.001 | D | Y |
| E9PKU7 | Neutral alpha-glucosidase AB OS=Homo sapiens GN=GANAB PE=1 SV=1 | 96483 | 5.5093 | 0.001 | D | Y |
| E9PL09 | 40S ribosomal protein S3 OS=Homo sapiens GN=RPS3 PE=1 SV=1 | 25354 | 10.1045 | 0.001 | D | Y |
| E9PL57 | Protein NEDD8-MDP1 (Fragment) OS=Homo sapiens GN=NEDD8-MDP1 PE=4 SV=1 | 19524 | 7.4004 | 0.001 | D | Y |
| E9PMV1 | Plectin (Fragment) OS=Homo sapiens GN=PLEC PE=1 SV=1 | 80730 | 6.3018 | 1.584 | D | Y |
| E9PNW4 | CD59 glycoprotein OS=Homo sapiens GN=CD59 PE=1 SV=1 | 11976 | 5.3672 | 0.337 | D | Y |
| E9PPQ4 | Ferritin (Fragment) OS=Homo sapiens GN=FTH1 PE=1 SV=1 | 6659 | 5.4185 | 0.391 | D | Y |
| E9PQD7 | 40S ribosomal protein S2 OS=Homo sapiens GN=RPS2 PE=1 SV=1 | 25195 | 10.6597 | 0.250 | D | Y |
| F5GWA7 | Prohibitin-2 (Fragment) OS=Homo sapiens GN=PHB2 PE=1 SV=1 | 29208 | 9.375 | 0.358 | D | Y |
| F5GX30 | Cation-dependent mannose-6-phosphate receptor OS=Homo sapiens GN=M6PR PE=1 SV=2 | 21524 | 6.2358 | 0.001 | D | Y |
| F5GY37 | Prohibitin-2 OS=Homo sapiens GN=PHB2 PE=1 SV=1 | 29704 | 10.2363 | 0.329 | D | Y |
| F5GYN4 | Ubiquitin thioesterase OTUB1 OS=Homo sapiens GN=OTUB1 PE=1 SV=1 | 28032 | 4.9849 | 0.228 | D | Y |
| F5GYQ1 | V-type proton ATPase subunit d 1 OS=Homo sapiens GN=ATP6V0D1 PE=1 SV=1 | 44631 | 4.8457 | 0.001 | D | Y |
| F5GZS6 | 4F2 cell-surface antigen heavy chain OS=Homo sapiens GN=SLC3A2 PE=1 SV=1 | 64832 | 4.7813 | 0.176 | D | Y |
| F5H006 | Lymphoid-restricted membrane protein OS=Homo sapiens GN=LRMP PE=1 SV=1 | 50393 | 5.8594 | 0.001 | D | Y |
| F5H039 | Gephyrin OS=Homo sapiens GN=GPHN PE=1 SV=1 | 84680 | 5.3643 | 0.001 | D | Y |
| F5H1U9 | Multiple PDZ domain protein OS=Homo sapiens GN=MPDZ PE=1 SV=1 | 222986 | 4.7622 | 0.001 | D | Y |
| F5H423 | Uncharacterized protein OS=Homo sapiens PE=3 SV=1 | 23331 | 9.0981 | 0.001 | D | Y |
| F5H481 | Protein N-lysine methyltransferase METTL20 (Fragment) OS=Homo sapiens GN=METTL20 PE | 7875 | 7.0781 | 2.582 | D | Y |
| F5H5G1 | Limbic system-associated membrane protein OS=Homo sapiens GN=LSAMP PE=1 SV=2 | 31722 | 5.5635 | 0.215 | D | Y |
| F5H608 | ATP synthase subunit d, mitochondrial OS=Homo sapiens GN=ATP5H PE=1 SV=2 | 8909 | 9.835 | 0.242 | D | Y |
| F5H7S3 | Tropomyosin alpha-1 chain OS=Homo sapiens GN=TPM1 PE=1 SV=2 | 28507 | 4.5454 | 0.428 | D | Y |
| F6U236 | Protein kinase C and casein kinase substrate in neurons protein 1 OS=Homo sapiens GN=PAC | 46117 | 5.0786 | 0.001 | D | Y |
| F8VQX6 | Methyltransferase-like protein 7A (Fragment) OS=Homo sapiens GN=METTL7A PE=1 SV=1 | 20648 | 8.0845 | 0.095 | D | Y |
| F8VSD4 | Ubiquitin-conjugating enzyme E2 N OS=Homo sapiens GN=UBE2N PE=1 SV=1 | 11975 | 9.5171 | 0.091 | D | Y |
| F8VUA5 | Ras-related protein Rab-5B (Fragment) OS=Homo sapiens GN=RAB5B PE=1 SV=1 | 12848 | 8.7905 | 0.001 | D | Y |
| F8VVM2 | Phosphate carrier protein, mitochondrial OS=Homo sapiens GN=SLC25A3 PE=1 SV=1 | 36137 | 9.4966 | 0.058 | D | Y |
| F8VXU5 | Vacuolar protein sorting-associated protein 29 OS=Homo sapiens GN=VPS29 PE=1 SV=1 | 23964 | 8.25 | 0.001 | D | Y |
| F8VY51 | Synembryn-B (Fragment) OS=Homo sapiens GN=RIC8B PE=1 SV=1 | 13419 | 5.3613 | 0.256 | D | Y |
| F8VZ49 | Heterogeneous nuclear ribonucleoprotein A1 (Fragment) OS=Homo sapiens GN=HNRNPA1 PE | 25670 | 8.458 | 0.391 | D | Y |
| F8W6P5 | Hemoglobin subunit beta (Fragment) OS=Homo sapiens GN=HBB PE=1 SV=1 | 9664 | 6.3384 | 3.152 | D | Y |
| F8W726 | Ubiquitin-associated protein 2-like OS=Homo sapiens GN=UBAP2L PE=1 SV=2 | 113558 | 6.2051 | 0.082 | D | Y |
| F8W7L3 | Alpha-2-macroglobulin (Fragment) OS=Homo sapiens GN=A2M PE=1 SV=1 | 18711 | 6.0864 | 0.048 | D | Y |
| F8W9U4 | Microtubule-associated protein OS=Homo sapiens GN=MAP4 PE=1 SV=1 | 88222 | 9.6035 | 0.125 | D | Y |
| F8WCA0 | Vesicle-associated membrane protein 2 OS=Homo sapiens GN=VAMP2 PE=4 SV=1 | 12925 | 8.7012 | 0.001 | D | Y |
| F8WE04 | Heat shock protein beta-1 OS=Homo sapiens GN=HSPB1 PE=1 SV=1 | 20393 | 9.394 | 2.542 | D | Y |
| G3V192 | Ferritin OS=Homo sapiens GN=FTH1 PE=1 SV=1 | 17642 | 5.1694 | 0.001 | D | Y |
| G3V1N2 | HCG1745306, isoform CRA_a OS=Homo sapiens GN=HBA2 PE=1 SV=1 | 11940 | 9.3018 | 1.453 | D | Y |
| G3V2N0 | Guanine nucleotide-binding protein subunit gamma OS=Homo sapiens GN=GNG2 PE=1 SV=1 | 12378 | 9.3047 | 0.001 | D | Y |
| G3XAL0 | Malate dehydrogenase OS=Homo sapiens GN=MDH2 PE=1 SV=1 | 24578 | 8.0024 | 1.282 | D | Y |
| G5EA42 | Tropomodulin 2 (Neuronal), isoform CRA_a OS=Homo sapiens GN=TMOD2 PE=1 SV=1 | 34471 | 6.4614 | 0.259 | D | Y |
| H0Y512 | Adipocyte plasma membrane-associated protein (Fragment) OS=Homo sapiens GN=APMAP PI | 45377 | 5.2998 | 0.470 | D | Y |
| H0Y532 | Selenium-binding protein 1 (Fragment) OS=Homo sapiens GN=SELENBP1 PE=1 SV=6 | 33903 | 5.2808 | 0.093 | D | Y |
| H0Y7V4 | Dynein heavy chain 8, axonemal OS=Homo sapiens GN=DNAH8 PE=1 SV=1 | 478565 | 5.7114 | 1.458 | D | Y |
| H0Y8G5 | Heterogeneous nuclear ribonucleoprotein D0 (Fragment) OS=Homo sapiens GN=HNRNPD PE | 29648 | 9.4526 | 0.001 | D | Y |
| H0YDN1 | Plectin (Fragment) OS=Homo sapiens GN=PLEC PE=1 SV=6 | 23731 | 4.6934 | 0.174 | D | Y |
| H0YH81 | ATP synthase subunit beta (Fragment) OS=Homo sapiens GN=ATP5B PE=1 SV=1 | 38226 | 5.2251 | 0.733 | D | Y |
| H0YJG0 | Creatine kinase B-type (Fragment) OS=Homo sapiens GN=CKB PE=1 SV=1 | 20134 | 6.2373 | 0.415 | D | Y |
| H0YK49 | Electron transfer flavoprotein subunit alpha, mitochondrial OS=Homo sapiens GN=ETFA PE=1 S | 24144 | 9.0747 | 0.138 | D | Y |
| H0YL72 | Isocitrate dehydrogenase [NAD] subunit alpha, mitochondrial OS=Homo sapiens GN=IDH3A PE | 35763 | 5.9897 | 0.001 | D | Y |
| H0YLI6 | Isocitrate dehydrogenase [NAD] subunit alpha, mitochondrial (Fragment) OS=Homo sapiens GN | 14799 | 8.584 | 0.219 | D | Y |
| H3BLZ8 | Probable ATP-dependent RNA helicase DDX17 OS=Homo sapiens GN=DDX17 PE=1 SV=1 | 80389 | 8.3511 | 0.001 | D | Y |

| | | | | | | |
|---|---|---|---|---|---|---|
| H3BMH2 | Ras-related protein Rab-11A (Fragment) OS=Homo sapiens GN=RAB11A PE=3 SV=1 | 17657 | 8.9253 | 0.199 | D | Y |
| H3BMN5 | Calretinin (Fragment) OS=Homo sapiens GN=CALB2 PE=1 SV=2 | 18455 | 4.5366 | 0.537 | D | Y |
| H3BN98 | Uncharacterized protein (Fragment) OS=Homo sapiens PE=4 SV=2 | 27150 | 9.8979 | 0.001 | D | Y |
| H3BNQ7 | 4-aminobutyrate aminotransferase, mitochondrial OS=Homo sapiens GN=ABAT PE=1 SV=1 | 57886 | 7.749 | 0.001 | D | Y |
| H3BNX8 | Cytochrome c oxidase subunit 5A, mitochondrial OS=Homo sapiens GN=COX5A PE=1 SV=1 | 17223 | 5.603 | 0.001 | D | Y |
| H3BPE7 | RNA-binding protein FUS OS=Homo sapiens GN=FUS PE=1 SV=1 | 53464 | 9.4951 | 0.001 | D | Y |
| H3BPS8 | Fructose-bisphosphate aldolase (Fragment) OS=Homo sapiens GN=ALDOA PE=1 SV=1 | 30407 | 7.2949 | 0.089 | D | Y |
| H3BQN4 | Fructose-bisphosphate aldolase OS=Homo sapiens GN=ALDOA PE=1 SV=1 | 39315 | 8.4331 | 1.768 | D | Y |
| H3BTN5 | Pyruvate kinase (Fragment) OS=Homo sapiens GN=PKM PE=1 SV=1 | 53011 | 6.3926 | 0.353 | D | Y |
| H7BY57 | Neurofascin (Fragment) OS=Homo sapiens GN=NFASC PE=1 SV=1 | 116625 | 5.8857 | 0.292 | D | Y |
| H7BYH4 | Superoxide dismutase [Cu-Zn] OS=Homo sapiens GN=SOD1 PE=1 SV=1 | 13900 | 5.6558 | 1.408 | D | Y |
| H7BYR8 | Myelin basic protein OS=Homo sapiens GN=MBP PE=1 SV=1 | 25726 | 11.6763 | 0.902 | D | Y |
| H7C1V0 | Cathepsin D (Fragment) OS=Homo sapiens GN=CTSD PE=1 SV=1 | 20358 | 8.5957 | 1.261 | D | Y |
| H7C3P7 | Ras-related protein Ral-A (Fragment) OS=Homo sapiens GN=RALA PE=1 SV=1 | 18360 | 4.396 | 0.001 | D | Y |
| H7C5W9 | Sarcoplasmic/endoplasmic reticulum calcium ATPase 2 (Fragment) OS=Homo sapiens GN=ATI | 102524 | 5.2793 | 0.001 | D | Y |
| H9KV31 | Neural cell adhesion molecule 2 OS=Homo sapiens GN=NCAM2 PE=1 SV=2 | 91066 | 5.2939 | 0.050 | D | Y |
| I3L0A0 | HCG2044781 OS=Homo sapiens GN=TMEM189-UBE2V1 PE=4 SV=1 | 42181 | 6.249 | 0.001 | D | Y |
| I3L0N3 | Vesicle-fusing ATPase OS=Homo sapiens GN=NSF PE=1 SV=1 | 82039 | 6.3735 | 0.827 | D | Y |
| I6L894 | Ankyrin-2 OS=Homo sapiens GN=ANK2 PE=1 SV=1 | 429989 | 4.8413 | 6.456 | D | Y |
| J3KN75 | TBC1 domain family member 8B OS=Homo sapiens GN=TBC1D8B PE=1 SV=1 | 128026 | 5.6074 | 0.523 | D | Y |
| J3KP97 | Coiled-coil domain-containing protein 18 (Fragment) OS=Homo sapiens GN=CCDC18 PE=1 SV | 174784 | 5.4272 | 0.001 | D | Y |
| J3KRE2 | Rho GDP-dissociation inhibitor 1 OS=Homo sapiens GN=ARHGDIA PE=1 SV=1 | 14805 | 4.3799 | 0.001 | D | Y |
| J3KTL2 | Serine/arginine-rich-splicing factor 1 OS=Homo sapiens GN=SRSF1 PE=1 SV=1 | 28311 | 10.2861 | 0.001 | D | Y |
| J3QRS3 | Myosin regulatory light chain 12A OS=Homo sapiens GN=MYL12A PE=1 SV=1 | 20443 | 4.4048 | 0.001 | D | Y |
| J3QSU6 | Tenascin OS=Homo sapiens GN=TNC PE=1 SV=1 | 220710 | 4.6274 | 0.164 | D | Y |
| J9JID7 | Lamin B2, isoform CRA_a OS=Homo sapiens GN=LMNB2 PE=1 SV=1 | 69905 | 5.3364 | 0.001 | D | Y |
| K7EK42 | Tubulin-folding cofactor B OS=Homo sapiens GN=TBCB PE=1 SV=1 | 21282 | 5.0376 | 0.110 | D | Y |
| K7EK78 | Uncharacterized protein OS=Homo sapiens PE=4 SV=1 | 12762 | 8.4814 | 0.001 | D | Y |
| K7EKU3 | FXYD domain-containing ion transport regulator 7 OS=Homo sapiens GN=FXYD7 PE=1 SV=1 | 12638 | 8.0845 | 0.001 | D | Y |
| K7ELL7 | Glucosidase 2 subunit beta OS=Homo sapiens GN=PRKCSH PE=1 SV=1 | 60154 | 4.1396 | 0.001 | D | Y |
| K7ELW0 | Protein deglycase DJ-1 OS=Homo sapiens GN=PARK7 PE=1 SV=1 | 17898 | 7.9937 | 1.055 | D | Y |
| K7EM49 | 6-phosphogluconate dehydrogenase, decarboxylating (Fragment) OS=Homo sapiens GN=PGD | 22243 | 5.9443 | 0.001 | D | Y |
| K7EN45 | Peptidyl-prolyl cis-trans isomerase NIMA-interacting 1 (Fragment) OS=Homo sapiens GN=PIN1 | 9938 | 4.7373 | 0.276 | D | Y |
| K7ER00 | Phenylalanine--tRNA ligase alpha subunit OS=Homo sapiens GN=FARSA PE=1 SV=1 | 62356 | 7.7534 | 0.001 | D | Y |
| K7N7A8 | Uncharacterized protein (Fragment) OS=Homo sapiens PE=3 SV=2 | 48766 | 4.8501 | 0.001 | D | Y |
| M0QXS5 | Heterogeneous nuclear ribonucleoprotein L (Fragment) OS=Homo sapiens GN=HNRNPL PE=1 | 58436 | 6.3208 | 0.131 | D | Y |
| O00154 | Cytosolic acyl coenzyme A thioester hydrolase OS=Homo sapiens GN=ACOT7 PE=1 SV=3 | 41769 | 8.5444 | 0.212 | D | Y |
| O00160 | Unconventional myosin-If OS=Homo sapiens GN=MYO1F PE=1 SV=3 | 124765 | 9.356 | 0.019 | D | Y |
| O00264 | Membrane-associated progesterone receptor component 1 OS=Homo sapiens GN=PGRMC1 P | 21657 | 4.3433 | 0.248 | D | Y |
| O00483 | Cytochrome c oxidase subunit NDUFA4 OS=Homo sapiens GN=NDUFA4 PE=1 SV=1 | 9363 | 9.75 | 0.245 | D | Y |
| O14531 | Dihydropyrimidinase-related protein 4 OS=Homo sapiens GN=DPYSL4 PE=1 SV=2 | 61838 | 6.6592 | 0.092 | D | Y |
| O14594 | Neurocan core protein OS=Homo sapiens GN=NCAN PE=1 SV=3 | 143003 | 5.0698 | 0.209 | D | Y |
| O14745 | Na(+)/H(+) exchange regulatory cofactor NHE-RF1 OS=Homo sapiens GN=SLC9A3R1 PE=1 S | 38844 | 5.4331 | 0.159 | D | Y |
| O14939 | Phospholipase D2 OS=Homo sapiens GN=PLD2 PE=1 SV=2 | 105920 | 7.3564 | 0.027 | D | Y |
| O15075 | Serine/threonine-protein kinase DCLK1 OS=Homo sapiens GN=DCLK1 PE=1 SV=2 | 82172 | 8.7935 | 0.001 | D | Y |
| O15144 | Actin-related protein 2/3 complex subunit 2 OS=Homo sapiens GN=ARPC2 PE=1 SV=1 | 34311 | 6.9785 | 0.061 | D | Y |
| O15212 | Prefoldin subunit 6 OS=Homo sapiens GN=PFDN6 PE=1 SV=1 | 14573 | 9.4263 | 0.189 | D | Y |
| O15240 | Neurosecretory protein VGF OS=Homo sapiens GN=VGF PE=1 SV=2 | 67217 | 4.5645 | 0.001 | D | Y |
| O43181 | NADH dehydrogenase [ubiquinone] iron-sulfur protein 4, mitochondrial OS=Homo sapiens GN=I | 20095 | 10.7593 | 0.527 | D | Y |
| O43295 | SLIT-ROBO Rho GTPase-activating protein 3 OS=Homo sapiens GN=SRGAP3 PE=1 SV=3 | 124425 | 6.2227 | 0.083 | D | Y |
| O43301 | Heat shock 70 kDa protein 12A OS=Homo sapiens GN=HSPA12A PE=1 SV=2 | 74931 | 6.3179 | 0.226 | D | Y |
| O43396 | Thioredoxin-like protein 1 OS=Homo sapiens GN=TXNL1 PE=1 SV=3 | 32230 | 4.6436 | 0.075 | D | Y |
| O43633 | Charged multivesicular body protein 2a OS=Homo sapiens GN=CHMP2A PE=1 SV=1 | 25087 | 5.6895 | 0.105 | D | Y |
| O43761 | Synaptogyrin-3 OS=Homo sapiens GN=SYNGR3 PE=1 SV=2 | 24539 | 8.2632 | 0.097 | D | Y |
| O43865 | Adenosylhomocysteinase 2 OS=Homo sapiens GN=AHCYL1 PE=1 SV=2 | 58913 | 6.4775 | 1.950 | D | Y |
| O43920 | NADH dehydrogenase [ubiquinone] iron-sulfur protein 5 OS=Homo sapiens GN=NDUFS5 PE=1 | 12509 | 9.4775 | 0.372 | D | Y |
| O60242 | Adhesion G protein-coupled receptor B3 OS=Homo sapiens GN=ADGRB3 PE=1 SV=2 | 171406 | 6.6489 | 0.032 | D | Y |
| O75061 | Putative tyrosine-protein phosphatase auxilin OS=Homo sapiens GN=DNAJC6 PE=1 SV=3 | 99933 | 6.8408 | 0.152 | D | Y |
| O75083 | WD repeat-containing protein 1 OS=Homo sapiens GN=WDR1 PE=1 SV=4 | 66151 | 6.1772 | 0.264 | D | Y |
| O75396 | Vesicle-trafficking protein SEC22b OS=Homo sapiens GN=SEC22B PE=1 SV=4 | 24577 | 6.501 | 0.093 | D | Y |
| O75489 | NADH dehydrogenase [ubiquinone] iron-sulfur protein 3, mitochondrial OS=Homo sapiens GN=I | 30222 | 7.3623 | 0.395 | D | Y |
| O75569 | Interferon-inducible double-stranded RNA-dependent protein kinase activator A OS=Homo sapie | 34382 | 8.4536 | 0.150 | D | Y |
| O75746 | Calcium-binding mitochondrial carrier protein Aralar1 OS=Homo sapiens GN=SLC25A12 PE=1 | 74714 | 8.4551 | 0.072 | D | Y |
| O75781 | Paralemmin-1 OS=Homo sapiens GN=PALM PE=1 SV=2 | 42050 | 4.7432 | 0.364 | D | Y |
| O75915 | PRA1 family protein 3 OS=Homo sapiens GN=ARL6IP5 PE=1 SV=1 | 21600 | 10.1045 | 0.151 | D | Y |
| O75947 | ATP synthase subunit d, mitochondrial OS=Homo sapiens GN=ATP5H PE=1 SV=3 | 18479 | 5.0244 | 0.274 | D | Y |
| O75964 | ATP synthase subunit g, mitochondrial OS=Homo sapiens GN=ATP5L PE=1 SV=3 | 11421 | 9.9844 | 0.485 | D | Y |
| O76041 | Nebulette OS=Homo sapiens GN=NEBL PE=1 SV=1 | 116380 | 8.0435 | 0.114 | D | Y |
| O76070 | Gamma-synuclein OS=Homo sapiens GN=SNCG PE=1 SV=2 | 13322 | 4.6626 | 0.459 | D | Y |
| O94811 | Tubulin polymerization-promoting protein OS=Homo sapiens GN=TPPP PE=1 SV=1 | 23679 | 9.8892 | 0.164 | D | Y |
| O94819 | Kelch repeat and BTB domain-containing protein 11 OS=Homo sapiens GN=KBTBD11 PE=1 S' | 65678 | 5.6909 | 0.294 | D | Y |
| O94826 | Mitochondrial import receptor subunit TOM70 OS=Homo sapiens GN=TOMM70A PE=1 SV=1 | 67412 | 6.772 | 0.047 | D | Y |
| O94925 | Glutaminase kidney isoform, mitochondrial OS=Homo sapiens GN=GLS PE=1 SV=1 | 73413 | 7.6011 | 0.211 | D | Y |
| O95171 | Sciellin OS=Homo sapiens GN=SCEL PE=1 SV=2 | 77504 | 9.7427 | 0.324 | D | Y |
| O95298 | NADH dehydrogenase [ubiquinone] 1 subunit C2 OS=Homo sapiens GN=NDUFC2 PE=1 SV=1 | 14178 | 9.2886 | 0.101 | D | Y |
| O95716 | Ras-related protein Rab-3D OS=Homo sapiens GN=RAB3D PE=1 SV=1 | 24251 | 4.5674 | 0.144 | D | Y |
| O95741 | Copine-6 OS=Homo sapiens GN=CPNE6 PE=1 SV=3 | 61951 | 5.168 | 0.155 | D | Y |
| O95782 | AP-2 complex subunit alpha-1 OS=Homo sapiens GN=AP2A1 PE=1 SV=3 | 107477 | 6.6255 | 0.091 | D | Y |
| O95865 | N(G),N(G)-dimethylarginine dimethylaminohydrolase 2 OS=Homo sapiens GN=DDAH2 PE=1 S' | 29625 | 5.5898 | 0.001 | D | Y |
| P00338 | L-lactate dehydrogenase A chain OS=Homo sapiens GN=LDHA PE=1 SV=2 | 36665 | 8.3657 | 0.113 | D | Y |
| P00352 | Retinal dehydrogenase 1 OS=Homo sapiens GN=ALDH1A1 PE=1 SV=2 | 54826 | 6.2886 | 0.184 | D | Y |

| | | | | | | |
|---|---|---|---|---|---|---|
| P00367 | Glutamate dehydrogenase 1, mitochondrial OS=Homo sapiens GN=GLUD1 PE=1 SV=2 | 61359 | 7.7021 | 1.049 | D | Y |
| P00441 | Superoxide dismutase [Cu-Zn] OS=Homo sapiens GN=SOD1 PE=1 SV=2 | 15925 | 5.666 | 1.780 | D | Y |
| P00505 | Aspartate aminotransferase, mitochondrial OS=Homo sapiens GN=GOT2 PE=1 SV=3 | 47487 | 9.2637 | 0.713 | D | Y |
| P00558 | Phosphoglycerate kinase 1 OS=Homo sapiens GN=PGK1 PE=1 SV=3 | 44586 | 8.1475 | 1.000 | D | Y |
| P00568 | Adenylate kinase isoenzyme 1 OS=Homo sapiens GN=AK1 PE=1 SV=3 | 21621 | 8.9897 | 0.040 | D | Y |
| P00918 | Carbonic anhydrase 2 OS=Homo sapiens GN=CA2 PE=1 SV=2 | 29227 | 7.0269 | 0.533 | D | Y |
| P01111 | GTPase NRas OS=Homo sapiens GN=NRAS PE=1 SV=1 | 21215 | 4.8208 | 0.001 | D | Y |
| P01213 | Proenkephalin-B OS=Homo sapiens GN=PDYN PE=1 SV=1 | 28367 | 5.9839 | 0.228 | D | Y |
| P01859 | Ig gamma-2 chain C region OS=Homo sapiens GN=IGHG2 PE=1 SV=2 | 35877 | 7.437 | 0.111 | D | Y |
| P01860 | Ig gamma-3 chain C region OS=Homo sapiens GN=IGHG3 PE=1 SV=2 | 41260 | 7.7886 | 0.007 | D | Y |
| P01861 | Ig gamma-4 chain C region OS=Homo sapiens GN=IGHG4 PE=1 SV=1 | 35917 | 7.1104 | 0.072 | D | Y |
| P01876 | Ig alpha-1 chain C region OS=Homo sapiens GN=IGHA1 PE=1 SV=2 | 37630 | 6.0571 | 0.057 | D | Y |
| P02008 | Hemoglobin subunit zeta OS=Homo sapiens GN=HBZ PE=1 SV=2 | 15627 | 8.6514 | 1.148 | D | Y |
| P02533 | Keratin, type I cytoskeletal 14 OS=Homo sapiens GN=KRT14 PE=1 SV=4 | 51529 | 4.8984 | 0.156 | D | Y |
| P02545 | Prelamin-A/C OS=Homo sapiens GN=LMNA PE=1 SV=1 | 74094 | 6.5815 | 0.107 | D | Y |
| P02647 | Apolipoprotein A-I OS=Homo sapiens GN=APOA1 PE=1 SV=1 | 30758 | 5.4316 | 0.135 | D | Y |
| P02649 | Apolipoprotein E OS=Homo sapiens GN=APOE PE=1 SV=1 | 36131 | 5.4829 | 0.146 | D | Y |
| P02768 | Serum albumin OS=Homo sapiens GN=ALB PE=1 SV=2 | 69321 | 5.8608 | 5.840 | D | Y |
| P02769 | Serum albumin OS=Bos taurus GN=ALB PE=1 SV=4 | 69248 | 5.7583 | 2.116 | D | Y |
| P02787 | Serotransferrin OS=Homo sapiens GN=TF PE=1 SV=3 | 77013 | 6.7515 | 0.315 | D | Y |
| P04083 | Annexin A1 OS=Homo sapiens GN=ANXA1 PE=1 SV=2 | 38689 | 6.6372 | 0.001 | D | Y |
| P04264 | Keratin, type II cytoskeletal 1 OS=Homo sapiens GN=KRT1 PE=1 SV=6 | 65998 | 8.2661 | 0.970 | D | Y |
| P04350 | Tubulin beta-4A chain OS=Homo sapiens GN=TUBB4A PE=1 SV=2 | 49553 | 4.5908 | 3.304 | D | Y |
| P04406 | Glyceraldehyde-3-phosphate dehydrogenase OS=Homo sapiens GN=GAPDH PE=1 SV=3 | 36030 | 8.6968 | 7.249 | D | Y |
| P04792 | Heat shock protein beta-1 OS=Homo sapiens GN=HSPB1 PE=1 SV=2 | 22768 | 5.959 | 0.729 | D | Y |
| P04843 | Dolichyl-diphosphooligosaccharide--protein glycosyltransferase subunit 1 OS=Homo sapiens GN | 68526 | 5.9268 | 0.060 | D | Y |
| P04899 | Guanine nucleotide-binding protein G(i) subunit alpha-2 OS=Homo sapiens GN=GNAI2 PE=1 S | 40425 | 5.1899 | 0.240 | D | Y |
| P05023 | Sodium/potassium-transporting ATPase subunit alpha-1 OS=Homo sapiens GN=ATP1A1 PE=1 | 112824 | 5.1694 | 5.061 | D | Y |
| P05026 | Sodium/potassium-transporting ATPase subunit beta-1 OS=Homo sapiens GN=ATP1B1 PE=1 S | 35038 | 8.6484 | 0.186 | D | Y |
| P05060 | Secretogranin-1 OS=Homo sapiens GN=CHGB PE=1 SV=2 | 78229 | 4.8354 | 0.093 | D | Y |
| P05091 | Aldehyde dehydrogenase, mitochondrial OS=Homo sapiens GN=ALDH2 PE=1 SV=2 | 56345 | 6.6694 | 0.531 | D | Y |
| P05129 | Protein kinase C gamma type OS=Homo sapiens GN=PRKCG PE=1 SV=3 | 78397 | 7.1689 | 0.118 | D | Y |
| P05141 | ADP/ATP translocase 2 OS=Homo sapiens GN=SLC25A5 PE=1 SV=7 | 32831 | 9.9917 | 0.569 | D | Y |
| P05165 | Propionyl-CoA carboxylase alpha chain, mitochondrial OS=Homo sapiens GN=PCCA PE=1 SV= | 80008 | 7.2524 | 0.057 | D | Y |
| P05413 | Fatty acid-binding protein, heart OS=Homo sapiens GN=FABP3 PE=1 SV=4 | 14848 | 6.3574 | 0.001 | D | Y |
| P05937 | Calbindin OS=Homo sapiens GN=CALB1 PE=1 SV=2 | 30006 | 4.5088 | 0.634 | D | Y |
| P06576 | ATP synthase subunit beta, mitochondrial OS=Homo sapiens GN=ATP5B PE=1 SV=3 | 56524 | 5.0962 | 3.469 | D | Y |
| P06733 | Alpha-enolase OS=Homo sapiens GN=ENO1 PE=1 SV=2 | 47139 | 7.1719 | 2.751 | D | Y |
| P06748 | Nucleophosmin OS=Homo sapiens GN=NPM1 PE=1 SV=2 | 32554 | 4.4443 | 0.164 | D | Y |
| P07099 | Epoxide hydrolase 1 OS=Homo sapiens GN=EPHX1 PE=1 SV=1 | 52914 | 6.8364 | 0.099 | D | Y |
| P07195 | L-lactate dehydrogenase B chain OS=Homo sapiens GN=LDHB PE=1 SV=2 | 36615 | 5.6396 | 1.264 | D | Y |
| P07196 | Neurofilament light polypeptide OS=Homo sapiens GN=NEFL PE=1 SV=3 | 61479 | 4.4326 | 0.554 | D | Y |
| P07237 | Protein disulfide-isomerase OS=Homo sapiens GN=P4HB PE=1 SV=3 | 57080 | 4.5645 | 0.107 | D | Y |
| P07339 | Cathepsin D OS=Homo sapiens GN=CTSD PE=1 SV=1 | 44523 | 6.0908 | 0.604 | D | Y |
| P07384 | Calpain-1 catalytic subunit OS=Homo sapiens GN=CAPN1 PE=1 SV=1 | 81838 | 5.3423 | 0.047 | D | Y |
| P07437 | Tubulin beta chain OS=Homo sapiens GN=TUBB PE=1 SV=2 | 49638 | 4.5908 | 2.942 | D | Y |
| P07737 | Profilin-1 OS=Homo sapiens GN=PFN1 PE=1 SV=2 | 15044 | 8.4595 | 0.650 | D | Y |
| P07858 | Cathepsin B OS=Homo sapiens GN=CTSB PE=1 SV=3 | 37796 | 5.855 | 0.097 | D | Y |
| P07900 | Heat shock protein HSP 90-alpha OS=Homo sapiens GN=HSP90AA1 PE=1 SV=5 | 84606 | 4.7476 | 1.248 | D | Y |
| P07919 | Cytochrome b-c1 complex subunit 6, mitochondrial OS=Homo sapiens GN=UQCRH PE=1 SV=2 | 10731 | 4.1865 | 0.235 | D | Y |
| P07954 | Fumarate hydratase, mitochondrial OS=Homo sapiens GN=FH PE=1 SV=3 | 54602 | 9.0835 | 0.648 | D | Y |
| P08133 | Annexin A6 OS=Homo sapiens GN=ANXA6 PE=1 SV=3 | 75825 | 5.2646 | 0.530 | D | Y |
| P08237 | ATP-dependent 6-phosphofructokinase, muscle type OS=Homo sapiens GN=PFKM PE=1 SV=2 | 85128 | 7.8926 | 0.139 | D | Y |
| P08238 | Heat shock protein HSP 90-beta OS=Homo sapiens GN=HSP90AB1 PE=1 SV=4 | 83212 | 4.7739 | 0.676 | D | Y |
| P08247 | Synaptophysin OS=Homo sapiens GN=SYP PE=1 SV=3 | 33823 | 4.4561 | 0.257 | D | Y |
| P08574 | Cytochrome c1, heme protein, mitochondrial OS=Homo sapiens GN=CYC1 PE=1 SV=3 | 35398 | 9.1831 | 0.459 | D | Y |
| P08670 | Vimentin OS=Homo sapiens GN=VIM PE=1 SV=4 | 53619 | 4.8633 | 0.861 | D | Y |
| P08754 | Guanine nucleotide-binding protein G(k) subunit alpha OS=Homo sapiens GN=GNAI3 PE=1 SV | 40506 | 5.3584 | 1.073 | D | Y |
| P08758 | Annexin A5 OS=Homo sapiens GN=ANXA5 PE=1 SV=2 | 35914 | 4.7329 | 0.577 | D | Y |
| P09104 | Gamma-enolase OS=Homo sapiens GN=ENO2 PE=1 SV=3 | 47239 | 4.7183 | 1.065 | D | Y |
| P09211 | Glutathione S-transferase P OS=Homo sapiens GN=GSTP1 PE=1 SV=2 | 23341 | 5.2822 | 0.558 | D | Y |
| P09238 | Stromelysin-2 OS=Homo sapiens GN=MMP10 PE=1 SV=1 | 54116 | 5.3965 | 0.195 | D | Y |
| P09382 | Galectin-1 OS=Homo sapiens GN=LGALS1 PE=1 SV=2 | 14706 | 5.1416 | 0.796 | D | Y |
| P09417 | Dihydropteridine reductase OS=Homo sapiens GN=QDPR PE=1 SV=2 | 25773 | 7.1719 | 0.126 | D | Y |
| P09471 | Guanine nucleotide-binding protein G(o) subunit alpha OS=Homo sapiens GN=GNAO1 PE=1 S | 40024 | 5.1899 | 1.054 | D | Y |
| P09543 | 2',3'-cyclic-nucleotide 3'-phosphodiesterase OS=Homo sapiens GN=CNP PE=1 SV=2 | 47548 | 9.3618 | 0.786 | D | Y |
| P09669 | Cytochrome c oxidase subunit 6C OS=Homo sapiens GN=COX6C PE=1 SV=2 | 8775 | 10.752 | 0.555 | D | Y |
| P09936 | Ubiquitin carboxyl-terminal hydrolase isozyme L1 OS=Homo sapiens GN=UCHL1 PE=1 SV=2 | 24808 | 5.1841 | 1.922 | D | Y |
| P09972 | Fructose-bisphosphate aldolase C OS=Homo sapiens GN=ALDOC PE=1 SV=2 | 39431 | 6.4351 | 1.689 | D | Y |
| P0DMV8 | Heat shock 70 kDa protein 1A OS=Homo sapiens GN=HSPA1A PE=1 SV=1 | 70009 | 5.3188 | 0.578 | D | Y |
| P10599 | Thioredoxin OS=Homo sapiens GN=TXN PE=1 SV=3 | 11729 | 4.6201 | 0.746 | D | Y |
| P10606 | Cytochrome c oxidase subunit 5B, mitochondrial OS=Homo sapiens GN=COX5B PE=1 SV=2 | 13686 | 9.0688 | 0.180 | D | Y |
| P10809 | 60 kDa heat shock protein, mitochondrial OS=Homo sapiens GN=HSPD1 PE=1 SV=2 | 61016 | 5.5503 | 0.793 | D | Y |
| P10909 | Clusterin OS=Homo sapiens GN=CLU PE=1 SV=1 | 52461 | 5.8389 | 0.336 | D | Y |
| P10915 | Hyaluronan and proteoglycan link protein 1 OS=Homo sapiens GN=HAPLN1 PE=2 SV=2 | 40139 | 7.1396 | 0.369 | D | Y |
| P11021 | 78 kDa glucose-regulated protein OS=Homo sapiens GN=HSPA5 PE=1 SV=2 | 72288 | 4.875 | 0.802 | D | Y |
| P11137 | Microtubule-associated protein 2 OS=Homo sapiens GN=MAP2 PE=1 SV=4 | 199402 | 4.6318 | 1.187 | D | Y |
| P11142 | Heat shock cognate 71 kDa protein OS=Homo sapiens GN=HSPA8 PE=1 SV=1 | 70854 | 5.2002 | 3.289 | D | Y |
| P11177 | Pyruvate dehydrogenase E1 component subunit beta, mitochondrial OS=Homo sapiens GN=PD | 39208 | 6.2021 | 0.160 | D | Y |
| P11216 | Glycogen phosphorylase, brain form OS=Homo sapiens GN=PYGB PE=1 SV=5 | 96634 | 6.3999 | 0.214 | D | Y |
| P11217 | Glycogen phosphorylase, muscle form OS=Homo sapiens GN=PYGM PE=1 SV=6 | 97030 | 6.583 | 1.520 | D | Y |

| | | | | | | |
|---|---|---|---|---|---|---|
| P11498 | Pyruvate carboxylase, mitochondrial OS=Homo sapiens GN=PC PE=1 SV=2 | 129551 | 6.3706 | 0.041 | D | Y |
| P12036 | Neurofilament heavy polypeptide OS=Homo sapiens GN=NEFH PE=1 SV=4 | 112410 | 5.874 | 1.320 | D | Y |
| P12236 | ADP/ATP translocase 3 OS=Homo sapiens GN=SLC25A6 PE=1 SV=4 | 32845 | 10.062 | 0.530 | D | Y |
| P12277 | Creatine kinase B-type OS=Homo sapiens GN=CKB PE=1 SV=1 | 42617 | 5.2178 | 2.447 | D | Y |
| P12532 | Creatine kinase U-type, mitochondrial OS=Homo sapiens GN=CKMT1A PE=1 SV=1 | 47007 | 8.3628 | 0.599 | D | Y |
| P13010 | X-ray repair cross-complementing protein 5 OS=Homo sapiens GN=XRCC5 PE=1 SV=3 | 82652 | 5.436 | 0.072 | D | Y |
| P13073 | Cytochrome c oxidase subunit 4 isoform 1, mitochondrial OS=Homo sapiens GN=COX4I1 PE=1 | 19564 | 9.9155 | 0.277 | D | Y |
| P13489 | Ribonuclease inhibitor OS=Homo sapiens GN=RNH1 PE=1 SV=2 | 49941 | 4.5176 | 0.080 | D | Y |
| P13521 | Secretogranin-2 OS=Homo sapiens GN=SCG2 PE=1 SV=2 | 70897 | 4.478 | 0.121 | D | Y |
| P13611 | Versican core protein OS=Homo sapiens GN=VCAN PE=1 SV=3 | 372588 | 4.2349 | 3.132 | D | Y |
| P13645 | Keratin, type I cytoskeletal 10 OS=Homo sapiens GN=KRT10 PE=1 SV=6 | 58791 | 4.9556 | 0.756 | D | Y |
| P13929 | Beta-enolase OS=Homo sapiens GN=ENO3 PE=1 SV=5 | 46957 | 7.6582 | 0.044 | D | Y |
| P14136 | Glial fibrillary acidic protein OS=Homo sapiens GN=GFAP PE=1 SV=1 | 49849 | 5.2559 | 31.749 | D | Y |
| P14174 | Macrophage migration inhibitory factor OS=Homo sapiens GN=MIF PE=1 SV=4 | 12468 | 7.9922 | 0.478 | D | Y |
| P14415 | Sodium/potassium-transporting ATPase subunit beta-2 OS=Homo sapiens GN=ATP1B2 PE=1 S | 33345 | 8.3438 | 0.147 | D | Y |
| P14618 | Pyruvate kinase PKM OS=Homo sapiens GN=PKM PE=1 SV=4 | 57900 | 7.7534 | 2.867 | D | Y |
| P14625 | Endoplasmin OS=Homo sapiens GN=HSP90B1 PE=1 SV=1 | 92411 | 4.5645 | 0.361 | D | Y |
| P14854 | Cytochrome c oxidase subunit 6B1 OS=Homo sapiens GN=COX6B1 PE=1 SV=2 | 10185 | 6.8657 | 1.393 | D | Y |
| P14867 | Gamma-aminobutyric acid receptor subunit alpha-1 OS=Homo sapiens GN=GABRA1 PE=1 SV | 51768 | 9.4731 | 1.492 | D | Y |
| P15104 | Glutamine synthetase OS=Homo sapiens GN=GLUL PE=1 SV=4 | 42037 | 6.4424 | 0.140 | D | Y |
| P15121 | Aldose reductase OS=Homo sapiens GN=AKR1B1 PE=1 SV=3 | 35830 | 6.5508 | 0.080 | D | Y |
| P15311 | Ezrin OS=Homo sapiens GN=EZR PE=1 SV=4 | 69369 | 5.8726 | 0.045 | D | Y |
| P15531 | Nucleoside diphosphate kinase A OS=Homo sapiens GN=NME1 PE=1 SV=1 | 17137 | 5.7671 | 0.248 | D | Y |
| P16152 | Carbonyl reductase [NADPH] 1 OS=Homo sapiens GN=CBR1 PE=1 SV=3 | 30355 | 8.417 | 0.494 | D | Y |
| P16870 | Carboxypeptidase E OS=Homo sapiens GN=CPE PE=1 SV=1 | 53117 | 4.8472 | 0.161 | D | Y |
| P17174 | Aspartate aminotransferase, cytoplasmic OS=Homo sapiens GN=GOT1 PE=1 SV=3 | 46218 | 6.5698 | 0.476 | D | Y |
| P17252 | Protein kinase C alpha type OS=Homo sapiens GN=PRKCA PE=1 SV=4 | 76700 | 6.6138 | 0.182 | D | Y |
| P17600 | Synapsin-1 OS=Homo sapiens GN=SYN1 PE=1 SV=3 | 74065 | 10.1895 | 3.063 | D | Y |
| P17661 | Desmin OS=Homo sapiens GN=DES PE=1 SV=3 | 53503 | 5.0303 | 0.162 | D | Y |
| P17677 | Neuromodulin OS=Homo sapiens GN=GAP43 PE=1 SV=1 | 24787 | 4.4473 | 0.309 | D | Y |
| P17858 | ATP-dependent 6-phosphofructokinase, liver type OS=Homo sapiens GN=PFKL PE=1 SV=6 | 84964 | 7.2261 | 0.054 | D | Y |
| P17948 | Vascular endothelial growth factor receptor 1 OS=Homo sapiens GN=FLT1 PE=1 SV=2 | 150672 | 8.3247 | 0.084 | D | Y |
| P17987 | T-complex protein 1 subunit alpha OS=Homo sapiens GN=TCP1 PE=1 SV=1 | 60305 | 5.7129 | 0.099 | D | Y |
| P18124 | 60S ribosomal protein L7 OS=Homo sapiens GN=RPL7 PE=1 SV=1 | 29207 | 11.0654 | 0.079 | D | Y |
| P18669 | Phosphoglycerate mutase 1 OS=Homo sapiens GN=PGAM1 PE=1 SV=2 | 28785 | 6.7866 | 1.114 | D | Y |
| P18859 | ATP synthase-coupling factor 6, mitochondrial OS=Homo sapiens GN=ATP5J PE=1 SV=1 | 12579 | 9.9829 | 0.463 | D | Y |
| P19338 | Nucleolin OS=Homo sapiens GN=NCL PE=1 SV=3 | 76568 | 4.4004 | 0.273 | D | Y |
| P19367 | Hexokinase-1 OS=Homo sapiens GN=HK1 PE=1 SV=3 | 102420 | 6.3472 | 1.068 | D | Y |
| P20020 | Plasma membrane calcium-transporting ATPase 1 OS=Homo sapiens GN=ATP2B1 PE=1 SV=3 | 138667 | 5.644 | 0.253 | D | Y |
| P20073 | Annexin A7 OS=Homo sapiens GN=ANXA7 PE=1 SV=3 | 52705 | 5.3525 | 0.101 | D | Y |
| P20336 | Ras-related protein Rab-3A OS=Homo sapiens GN=RAB3A PE=1 SV=1 | 24968 | 4.6626 | 1.573 | D | Y |
| P20338 | Ras-related protein Rab-4A OS=Homo sapiens GN=RAB4A PE=1 SV=3 | 24374 | 5.7231 | 0.104 | D | Y |
| P20340 | Ras-related protein Rab-6A OS=Homo sapiens GN=RAB6A PE=1 SV=3 | 23577 | 5.2266 | 0.053 | D | Y |
| P21281 | V-type proton ATPase subunit B, brain isoform OS=Homo sapiens GN=ATP6V1B2 PE=1 SV=3 | 56464 | 5.4492 | 0.438 | D | Y |
| P21579 | Synaptotagmin-1 OS=Homo sapiens GN=SYT1 PE=1 SV=1 | 47542 | 8.2061 | 0.715 | D | Y |
| P21796 | Voltage-dependent anion-selective channel protein 1 OS=Homo sapiens GN=VDAC1 PE=1 SV | 30753 | 8.8682 | 4.632 | D | Y |
| P22314 | Ubiquitin-like modifier-activating enzyme 1 OS=Homo sapiens GN=UBA1 PE=1 SV=3 | 117774 | 5.3789 | 0.247 | D | Y |
| P22626 | Heterogeneous nuclear ribonucleoproteins A2/B1 OS=Homo sapiens GN=HNRNPA2B1 PE=1 S | 37406 | 9.1948 | 1.691 | D | Y |
| P23246 | Splicing factor, proline- and glutamine-rich OS=Homo sapiens GN=SFPQ PE=1 SV=2 | 76101 | 9.772 | 0.245 | D | Y |
| P23284 | Peptidyl-prolyl cis-trans isomerase B OS=Homo sapiens GN=PPIB PE=1 SV=2 | 23727 | 9.8511 | 0.048 | D | Y |
| P23297 | Protein S100-A1 OS=Homo sapiens GN=S100A1 PE=1 SV=2 | 10539 | 4.1865 | 0.517 | D | Y |
| P23471 | Receptor-type tyrosine-protein phosphatase zeta OS=Homo sapiens GN=PTPRZ1 PE=1 SV=4 | 254427 | 4.5718 | 0.673 | D | Y |
| P24752 | Acetyl-CoA acetyltransferase, mitochondrial OS=Homo sapiens GN=ACAT1 PE=1 SV=1 | 45170 | 9.1201 | 0.159 | D | Y |
| P25705 | ATP synthase subunit alpha, mitochondrial OS=Homo sapiens GN=ATP5A1 PE=1 SV=1 | 59713 | 9.4321 | 3.145 | D | Y |
| P26038 | Moesin OS=Homo sapiens GN=MSN PE=1 SV=3 | 67777 | 6.0103 | 0.073 | D | Y |
| P26232 | Catenin alpha-2 OS=Homo sapiens GN=CTNNA2 PE=1 SV=5 | 105246 | 5.3745 | 0.058 | D | Y |
| P26641 | Elongation factor 1-gamma OS=Homo sapiens GN=EEF1G PE=1 SV=3 | 50087 | 6.2358 | 0.334 | D | Y |
| P27338 | Amine oxidase [flavin-containing] B OS=Homo sapiens GN=MAOB PE=1 SV=3 | 58725 | 7.248 | 0.437 | D | Y |
| P27348 | 14-3-3 protein theta OS=Homo sapiens GN=YWHAQ PE=1 SV=1 | 27746 | 4.4854 | 0.762 | D | Y |
| P27482 | Calmodulin-like protein 3 OS=Homo sapiens GN=CALML3 PE=1 SV=2 | 16879 | 4.0972 | 2.185 | D | Y |
| P27797 | Calreticulin OS=Homo sapiens GN=CALR PE=1 SV=1 | 48111 | 4.0942 | 0.512 | D | Y |
| P27816 | Microtubule-associated protein 4 OS=Homo sapiens GN=MAP4 PE=1 SV=3 | 120929 | 5.1387 | 0.065 | D | Y |
| P27824 | Calnexin OS=Homo sapiens GN=CANX PE=1 SV=2 | 67525 | 4.2686 | 0.444 | D | Y |
| P28066 | Proteasome subunit alpha type-5 OS=Homo sapiens GN=PSMA5 PE=1 SV=3 | 26394 | 4.5439 | 0.094 | D | Y |
| P28072 | Proteasome subunit beta type-6 OS=Homo sapiens GN=PSMB6 PE=1 SV=4 | 25341 | 4.6069 | 0.138 | D | Y |
| P28074 | Proteasome subunit beta type-5 OS=Homo sapiens GN=PSMB5 PE=1 SV=3 | 28462 | 6.5083 | 0.139 | D | Y |
| P28482 | Mitogen-activated protein kinase 1 OS=Homo sapiens GN=MAPK1 PE=1 SV=3 | 41363 | 6.5317 | 0.298 | D | Y |
| P30038 | Delta-1-pyrroline-5-carboxylate dehydrogenase, mitochondrial OS=Homo sapiens GN=ALDH4A | 61680 | 8.0566 | 0.247 | D | Y |
| P30040 | Endoplasmic reticulum resident protein 29 OS=Homo sapiens GN=ERP29 PE=1 SV=4 | 28975 | 7.2832 | 0.250 | D | Y |
| P30041 | Peroxiredoxin-6 OS=Homo sapiens GN=PRDX6 PE=1 SV=3 | 25019 | 5.9575 | 0.910 | D | Y |
| P30044 | Peroxiredoxin-5, mitochondrial OS=Homo sapiens GN=PRDX5 PE=1 SV=4 | 22072 | 8.9839 | 0.191 | D | Y |
| P30049 | ATP synthase subunit delta, mitochondrial OS=Homo sapiens GN=ATP5D PE=1 SV=2 | 17479 | 5.1914 | 0.955 | D | Y |
| P30050 | 60S ribosomal protein L12 OS=Homo sapiens GN=RPL12 PE=1 SV=1 | 17807 | 9.8965 | 0.100 | D | Y |
| P30084 | Enoyl-CoA hydratase, mitochondrial OS=Homo sapiens GN=ECHS1 PE=1 SV=4 | 31367 | 8.0728 | 0.174 | D | Y |
| P30086 | Phosphatidylethanolamine-binding protein 1 OS=Homo sapiens GN=PEBP1 PE=1 SV=3 | 21043 | 7.3901 | 1.591 | D | Y |
| P30101 | Protein disulfide-isomerase A3 OS=Homo sapiens GN=PDIA3 PE=1 SV=4 | 56746 | 5.9312 | 0.286 | D | Y |
| P30622 | CAP-Gly domain-containing linker protein 1 OS=Homo sapiens GN=CLIP1 PE=1 SV=2 | 162146 | 5.1138 | 0.598 | D | Y |
| P31040 | Succinate dehydrogenase [ubiquinone] flavoprotein subunit, mitochondrial OS=Homo sapiens G | 72645 | 7.0415 | 0.061 | D | Y |
| P31150 | Rab GDP dissociation inhibitor alpha OS=Homo sapiens GN=GDI1 PE=1 SV=2 | 50550 | 4.8135 | 0.822 | D | Y |
| P31930 | Cytochrome b-c1 complex subunit 1, mitochondrial OS=Homo sapiens GN=UQCRC1 PE=1 SV | 52612 | 5.9092 | 0.580 | D | Y |
| P31942 | Heterogeneous nuclear ribonucleoprotein H3 OS=Homo sapiens GN=HNRNPH3 PE=1 SV=2 | 36903 | 6.3999 | 0.291 | D | Y |

| | | | | | | |
|---|---|---|---|---|---|---|
| P31946 | 14-3-3 protein beta/alpha OS=Homo sapiens GN=YWHAB PE=1 SV=3 | 28064 | 4.5674 | 9.485 | D | Y |
| P32119 | Peroxiredoxin-2 OS=Homo sapiens GN=PRDX2 PE=1 SV=5 | 21878 | 5.5679 | 2.204 | D | Y |
| P33897 | ATP-binding cassette sub-family D member 1 OS=Homo sapiens GN=ABCD1 PE=1 SV=2 | 82884 | 9.1011 | 0.047 | D | Y |
| P34932 | Heat shock 70 kDa protein 4 OS=Homo sapiens GN=HSPA4 PE=1 SV=4 | 94271 | 4.9131 | 0.097 | D | Y |
| P35080 | Profilin-2 OS=Homo sapiens GN=PFN2 PE=1 SV=3 | 15036 | 6.7778 | 0.243 | D | Y |
| P35527 | Keratin, type I cytoskeletal 9 OS=Homo sapiens GN=KRT9 PE=1 SV=3 | 62026 | 4.9585 | 0.803 | D | Y |
| P35611 | Alpha-adducin OS=Homo sapiens GN=ADD1 PE=1 SV=2 | 80904 | 5.4844 | 0.330 | D | Y |
| P35908 | Keratin, type II cytoskeletal 2 epidermal OS=Homo sapiens GN=KRT2 PE=1 SV=2 | 65393 | 8.0537 | 0.445 | D | Y |
| P36871 | Phosphoglucomutase-1 OS=Homo sapiens GN=PGM1 PE=1 SV=3 | 61410 | 6.3032 | 0.126 | D | Y |
| P38117 | Electron transfer flavoprotein subunit beta OS=Homo sapiens GN=ETFB PE=1 SV=3 | 27826 | 8.2456 | 0.281 | D | Y |
| P38159 | RNA-binding motif protein, X chromosome OS=Homo sapiens GN=RBMX PE=1 SV=3 | 42306 | 10.2437 | 0.089 | D | Y |
| P38606 | V-type proton ATPase catalytic subunit A OS=Homo sapiens GN=ATP6V1A PE=1 SV=2 | 68260 | 5.1855 | 0.308 | D | Y |
| P38646 | Stress-70 protein, mitochondrial OS=Homo sapiens GN=HSPA9 PE=1 SV=2 | 73634 | 5.7803 | 0.499 | D | Y |
| P40123 | Adenylyl cyclase-associated protein 2 OS=Homo sapiens GN=CAP2 PE=1 SV=1 | 52790 | 5.9253 | 0.132 | D | Y |
| P40925 | Malate dehydrogenase, cytoplasmic OS=Homo sapiens GN=MDH1 PE=1 SV=4 | 36403 | 7.1704 | 1.803 | D | Y |
| P40926 | Malate dehydrogenase, mitochondrial OS=Homo sapiens GN=MDH2 PE=1 SV=3 | 35480 | 8.8213 | 0.752 | D | Y |
| P40939 | Trifunctional enzyme subunit alpha, mitochondrial OS=Homo sapiens GN=HADHA PE=1 SV=2 | 82946 | 9.3413 | 0.098 | D | Y |
| P43004 | Excitatory amino acid transporter 2 OS=Homo sapiens GN=SLC1A2 PE=1 SV=2 | 62063 | 6.0762 | 0.385 | D | Y |
| P45880 | Voltage-dependent anion-selective channel protein 2 OS=Homo sapiens GN=VDAC2 PE=1 SV= | 31546 | 7.4678 | 2.067 | D | Y |
| P46783 | 40S ribosomal protein S10 OS=Homo sapiens GN=RPS10 PE=1 SV=1 | 18885 | 10.5088 | 0.395 | D | Y |
| P46821 | Microtubule-associated protein 1B OS=Homo sapiens GN=MAP1B PE=1 SV=2 | 270465 | 4.5381 | 0.695 | D | Y |
| P47985 | Cytochrome b-c1 complex subunit Rieske, mitochondrial OS=Homo sapiens GN=UQCRFS1 PE | 29649 | 8.3936 | 0.397 | D | Y |
| P48539 | Purkinje cell protein 4 OS=Homo sapiens GN=PCP4 PE=1 SV=3 | 6787 | 7.248 | 0.070 | D | Y |
| P48735 | Isocitrate dehydrogenase [NADP], mitochondrial OS=Homo sapiens GN=IDH2 PE=1 SV=2 | 50876 | 8.855 | 0.375 | D | Y |
| P49006 | MARCKS-related protein OS=Homo sapiens GN=MARCKSL1 PE=1 SV=2 | 19517 | 4.4531 | 0.352 | D | Y |
| P49411 | Elongation factor Tu, mitochondrial OS=Homo sapiens GN=TUFM PE=1 SV=2 | 49510 | 7.3726 | 0.405 | D | Y |
| P49418 | Amphiphysin OS=Homo sapiens GN=AMPH PE=1 SV=1 | 76210 | 4.374 | 0.508 | D | Y |
| P49419 | Alpha-aminoadipic semialdehyde dehydrogenase OS=Homo sapiens GN=ALDH7A1 PE=1 SV= | 58450 | 7.938 | 0.328 | D | Y |
| P50148 | Guanine nucleotide-binding protein G(q) subunit alpha OS=Homo sapiens GN=GNAQ PE=1 SV | 42115 | 5.3408 | 0.042 | D | Y |
| P50213 | Isocitrate dehydrogenase [NAD] subunit alpha, mitochondrial OS=Homo sapiens GN=IDH3A PE | 39566 | 6.4907 | 0.818 | D | Y |
| P50395 | Rab GDP dissociation inhibitor beta OS=Homo sapiens GN=GDI2 PE=1 SV=2 | 50630 | 6.0557 | 0.092 | D | Y |
| P50570 | Dynamin-2 OS=Homo sapiens GN=DNM2 PE=1 SV=2 | 98003 | 7.1162 | 0.175 | D | Y |
| P50991 | T-complex protein 1 subunit delta OS=Homo sapiens GN=CCT4 PE=1 SV=4 | 57887 | 7.7827 | 0.047 | D | Y |
| P50993 | Sodium/potassium-transporting ATPase subunit alpha-2 OS=Homo sapiens GN=ATP1A2 PE=1 | 112193 | 5.3247 | 0.909 | D | Y |
| P51149 | Ras-related protein Rab-7a OS=Homo sapiens GN=RAB7A PE=1 SV=1 | 23474 | 6.5742 | 0.404 | D | Y |
| P51674 | Neuronal membrane glycoprotein M6-a OS=Homo sapiens GN=GPM6A PE=1 SV=2 | 31188 | 5.0024 | 0.137 | D | Y |
| P51970 | NADH dehydrogenase [ubiquinone] 1 alpha subcomplex subunit 8 OS=Homo sapiens GN=NDU | 20092 | 7.5483 | 0.114 | D | Y |
| P51991 | Heterogeneous nuclear ribonucleoprotein A3 OS=Homo sapiens GN=HNRNPA3 PE=1 SV=2 | 39570 | 9.2212 | 0.457 | D | Y |
| P52597 | Heterogeneous nuclear ribonucleoprotein F OS=Homo sapiens GN=HNRNPF PE=1 SV=3 | 45642 | 5.2427 | 0.352 | D | Y |
| P52907 | F-actin-capping protein subunit alpha-1 OS=Homo sapiens GN=CAPZA1 PE=1 SV=3 | 32902 | 5.3262 | 0.056 | D | Y |
| P52943 | Cysteine-rich protein 2 OS=Homo sapiens GN=CRIP2 PE=1 SV=1 | 22478 | 8.7393 | 0.068 | D | Y |
| P53673 | Beta-crystallin A4 OS=Homo sapiens GN=CRYBA4 PE=1 SV=3 | 22359 | 5.8066 | 1.514 | D | Y |
| P53999 | Activated RNA polymerase II transcriptional coactivator p15 OS=Homo sapiens GN=SUB1 PE= | 14386 | 10.0737 | 0.438 | D | Y |
| P54652 | Heat shock-related 70 kDa protein 2 OS=Homo sapiens GN=HSPA2 PE=1 SV=1 | 69977 | 5.4082 | 2.040 | D | Y |
| P54707 | Potassium-transporting ATPase alpha chain 2 OS=Homo sapiens GN=ATP12A PE=1 SV=3 | 115437 | 6.0981 | 3.853 | D | Y |
| P54886 | Delta-1-pyrroline-5-carboxylate synthase OS=Homo sapiens GN=ALDH18A1 PE=1 SV=2 | 87247 | 6.6768 | 0.015 | D | Y |
| P54920 | Alpha-soluble NSF attachment protein OS=Homo sapiens GN=NAPA PE=1 SV=3 | 33211 | 5.064 | 0.130 | D | Y |
| P55072 | Transitional endoplasmic reticulum ATPase OS=Homo sapiens GN=VCP PE=1 SV=4 | 89265 | 4.9556 | 0.260 | D | Y |
| P55084 | Trifunctional enzyme subunit beta, mitochondrial OS=Homo sapiens GN=HADHB PE=1 SV=3 | 51261 | 9.75 | 0.128 | D | Y |
| P55087 | Aquaporin-4 OS=Homo sapiens GN=AQP4 PE=1 SV=2 | 34806 | 7.5557 | 0.001 | D | Y |
| P55265 | Double-stranded RNA-specific adenosine deaminase OS=Homo sapiens GN=ADAR PE=1 SV= | 135980 | 8.7173 | 0.539 | D | Y |
| P55795 | Heterogeneous nuclear ribonucleoprotein H2 OS=Homo sapiens GN=HNRNPH2 PE=1 SV=1 | 49232 | 5.8521 | 0.181 | D | Y |
| P55809 | Succinyl-CoA:3-ketoacid coenzyme A transferase 1, mitochondrial OS=Homo sapiens GN=OXC | 56121 | 7.2202 | 0.144 | D | Y |
| P58546 | Myotrophin OS=Homo sapiens GN=MTPN PE=1 SV=2 | 12886 | 5.1343 | 0.623 | D | Y |
| P60174 | Triosephosphate isomerase OS=Homo sapiens GN=TPI1 PE=1 SV=3 | 30771 | 5.5474 | 1.560 | D | Y |
| P60201 | Myelin proteolipid protein OS=Homo sapiens GN=PLP1 PE=1 SV=2 | 30057 | 8.2881 | 0.423 | D | Y |
| P60709 | Actin, cytoplasmic 1 OS=Homo sapiens GN=ACTB PE=1 SV=1 | 41709 | 5.1431 | 12.784 | D | Y |
| P60880 | Synaptosomal-associated protein 25 OS=Homo sapiens GN=SNAP25 PE=1 SV=1 | 23300 | 4.4575 | 0.426 | D | Y |
| P60983 | Glia maturation factor beta OS=Homo sapiens GN=GMFB PE=1 SV=2 | 16702 | 5.0098 | 0.182 | D | Y |
| P61006 | Ras-related protein Rab-8A OS=Homo sapiens GN=RAB8A PE=1 SV=1 | 23653 | 9.4321 | 0.079 | D | Y |
| P61019 | Ras-related protein Rab-2A OS=Homo sapiens GN=RAB2A PE=1 SV=1 | 23530 | 6.0806 | 0.762 | D | Y |
| P61026 | Ras-related protein Rab-10 OS=Homo sapiens GN=RAB10 PE=1 SV=1 | 22526 | 8.5737 | 0.055 | D | Y |
| P61106 | Ras-related protein Rab-14 OS=Homo sapiens GN=RAB14 PE=1 SV=4 | 23881 | 5.7979 | 0.178 | D | Y |
| P61163 | Alpha-centractin OS=Homo sapiens GN=ACTR1A PE=1 SV=1 | 42586 | 6.1846 | 0.001 | D | Y |
| P61204 | ADP-ribosylation factor 3 OS=Homo sapiens GN=ARF3 PE=1 SV=2 | 20587 | 7.3887 | 0.001 | D | Y |
| P61266 | Syntaxin-1B OS=Homo sapiens GN=STX1B PE=1 SV=1 | 33223 | 5.0859 | 0.314 | D | Y |
| P61604 | 10 kDa heat shock protein, mitochondrial OS=Homo sapiens GN=HSPE1 PE=1 SV=2 | 10924 | 9.4702 | 0.973 | D | Y |
| P61764 | Syntaxin-binding protein 1 OS=Homo sapiens GN=STXBP1 PE=1 SV=1 | 67525 | 6.5098 | 1.203 | D | Y |
| P61978 | Heterogeneous nuclear ribonucleoprotein K OS=Homo sapiens GN=HNRNPK PE=1 SV=1 | 50944 | 5.2207 | 0.863 | D | Y |
| P61981 | 14-3-3 protein gamma OS=Homo sapiens GN=YWHAG PE=1 SV=2 | 28284 | 4.6069 | 7.829 | D | Y |
| P62158 | Calmodulin OS=Homo sapiens GN=CALM1 PE=1 SV=2 | 16826 | 3.8833 | 4.788 | D | Y |
| P62241 | 40S ribosomal protein S8 OS=Homo sapiens GN=RPS8 PE=1 SV=2 | 24190 | 10.7153 | 0.001 | D | Y |
| P62258 | 14-3-3 protein epsilon OS=Homo sapiens GN=YWHAE PE=1 SV=1 | 29155 | 4.4355 | 6.331 | D | Y |
| P62269 | 40S ribosomal protein S18 OS=Homo sapiens GN=RPS18 PE=1 SV=3 | 17707 | 11.4141 | 0.110 | D | Y |
| P62277 | 40S ribosomal protein S13 OS=Homo sapiens GN=RPS13 PE=1 SV=2 | 17211 | 10.9409 | 0.061 | D | Y |
| P62736 | Actin, aortic smooth muscle OS=Homo sapiens GN=ACTA2 PE=1 SV=1 | 41981 | 5.0771 | 1.361 | D | Y |
| P62805 | Histone H4 OS=Homo sapiens GN=HIST1H4A PE=1 SV=2 | 11360 | 11.7671 | 2.257 | D | Y |
| P62873 | Guanine nucleotide-binding protein G(I)/G(S)/G(T) subunit beta-1 OS=Homo sapiens GN=GNB1 | 37353 | 5.5356 | 2.009 | D | Y |
| P62879 | Guanine nucleotide-binding protein G(I)/G(S)/G(T) subunit beta-2 OS=Homo sapiens GN=GNB2 | 37307 | 5.5356 | 0.145 | D | Y |
| P62937 | Peptidyl-prolyl cis-trans isomerase A OS=Homo sapiens GN=PPIA PE=1 SV=2 | 18000 | 7.853 | 1.520 | D | Y |
| P62942 | Peptidyl-prolyl cis-trans isomerase FKBP1A OS=Homo sapiens GN=FKBP1A PE=1 SV=2 | 11943 | 8.6558 | 0.426 | D | Y |

| | | | | | | |
|---|---|---|---|---|---|---|
| P63000 | Ras-related C3 botulinum toxin substrate 1 OS=Homo sapiens GN=RAC1 PE=1 SV=1 | 21436 | 8.5723 | 0.127 | D | Y |
| P63010 | AP-2 complex subunit beta OS=Homo sapiens GN=AP2B1 PE=1 SV=1 | 104486 | 5.0552 | 0.241 | D | Y |
| P63096 | Guanine nucleotide-binding protein G(i) subunit alpha-1 OS=Homo sapiens GN=GNAI1 PE=1 S | 40335 | 5.5913 | 0.713 | D | Y |
| P67775 | Serine/threonine-protein phosphatase 2A catalytic subunit alpha isoform OS=Homo sapiens GN | 35571 | 5.168 | 0.227 | D | Y |
| P67936 | Tropomyosin alpha-4 chain OS=Homo sapiens GN=TPM4 PE=1 SV=3 | 28504 | 4.4707 | 0.205 | D | Y |
| P68036 | Ubiquitin-conjugating enzyme E2 L3 OS=Homo sapiens GN=UBE2L3 PE=1 SV=1 | 17850 | 8.7993 | 0.205 | D | Y |
| P68104 | Elongation factor 1-alpha 1 OS=Homo sapiens GN=EEF1A1 PE=1 SV=1 | 50109 | 9.3428 | 0.806 | D | Y |
| P68133 | Actin, alpha skeletal muscle OS=Homo sapiens GN=ACTA1 PE=1 SV=1 | 42023 | 5.0713 | 6.063 | D | Y |
| P68363 | Tubulin alpha-1B chain OS=Homo sapiens GN=TUBA1B PE=1 SV=1 | 50119 | 4.7622 | 0.505 | D | Y |
| P68366 | Tubulin alpha-4A chain OS=Homo sapiens GN=TUBA4A PE=1 SV=1 | 49892 | 4.752 | 4.367 | D | Y |
| P68371 | Tubulin beta-4B chain OS=Homo sapiens GN=TUBB4B PE=1 SV=1 | 49799 | 4.6025 | 14.561 | D | Y |
| P68871 | Hemoglobin subunit beta OS=Homo sapiens GN=HBB PE=1 SV=2 | 15988 | 6.8804 | 6.337 | D | Y |
| P69905 | Hemoglobin subunit alpha OS=Homo sapiens GN=HBA1 PE=1 SV=2 | 15247 | 9.1787 | 10.236 | D | Y |
| P78324 | Tyrosine-protein phosphatase non-receptor type substrate 1 OS=Homo sapiens GN=SIRPA PE | 54932 | 6.5361 | 0.145 | D | Y |
| P78559 | Microtubule-associated protein 1A OS=Homo sapiens GN=MAP1A PE=1 SV=6 | 305296 | 4.6567 | 0.428 | D | Y |
| P80723 | Brain acid soluble protein 1 OS=Homo sapiens GN=BASP1 PE=1 SV=2 | 22680 | 4.4238 | 0.553 | D | Y |
| P84074 | Neuron-specific calcium-binding protein hippocalcin OS=Homo sapiens GN=HPCA PE=1 SV=2 | 22412 | 4.6758 | 0.386 | D | Y |
| Q00577 | Transcriptional activator protein Pur-alpha OS=Homo sapiens GN=PURA PE=1 SV=2 | 34889 | 6.0249 | 0.648 | D | Y |
| Q00839 | Heterogeneous nuclear ribonucleoprotein U OS=Homo sapiens GN=HNRNPU PE=1 SV=6 | 90527 | 5.6484 | 0.307 | D | Y |
| Q01082 | Spectrin beta chain, non-erythrocytic 1 OS=Homo sapiens GN=SPTBN1 PE=1 SV=2 | 274437 | 5.2515 | 0.561 | D | Y |
| Q01469 | Fatty acid-binding protein, epidermal OS=Homo sapiens GN=FABP5 PE=1 SV=3 | 15154 | 6.8042 | 0.458 | D | Y |
| Q01518 | Adenylyl cyclase-associated protein 1 OS=Homo sapiens GN=CAP1 PE=1 SV=5 | 51868 | 8.0581 | 0.212 | D | Y |
| Q01813 | ATP-dependent 6-phosphofructokinase, platelet type OS=Homo sapiens GN=PFKP PE=1 SV=2 | 85541 | 7.3535 | 0.102 | D | Y |
| Q02750 | Dual specificity mitogen-activated protein kinase kinase 1 OS=Homo sapiens GN=MAP2K1 PE= | 43411 | 6.1772 | 0.177 | D | Y |
| Q02978 | Mitochondrial 2-oxoglutarate/malate carrier protein OS=Homo sapiens GN=SLC25A11 PE=1 SV | 34039 | 10.2085 | 0.278 | D | Y |
| Q03113 | Guanine nucleotide-binding protein subunit alpha-12 OS=Homo sapiens GN=GNA12 PE=1 SV= | 44251 | 10.2378 | 0.414 | D | Y |
| Q04695 | Keratin, type I cytoskeletal 17 OS=Homo sapiens GN=KRT17 PE=1 SV=2 | 48076 | 4.7769 | 1.478 | D | Y |
| Q04917 | 14-3-3 protein eta OS=Homo sapiens GN=YWHAH PE=1 SV=4 | 28201 | 4.5615 | 1.441 | D | Y |
| Q05639 | Elongation factor 1-alpha 2 OS=Homo sapiens GN=EEF1A2 PE=1 SV=1 | 50438 | 9.3472 | 0.939 | D | Y |
| Q07283 | Trichohyalin OS=Homo sapiens GN=TCHH PE=1 SV=2 | 253775 | 5.562 | 0.808 | D | Y |
| Q07666 | KH domain-containing, RNA-binding, signal transduction-associated protein 1 OS=Homo sapien | 48197 | 8.9136 | 0.236 | D | Y |
| Q08211 | ATP-dependent RNA helicase A OS=Homo sapiens GN=DHX9 PE=1 SV=4 | 140868 | 6.3955 | 0.063 | D | Y |
| Q08378 | Golgin subfamily A member 3 OS=Homo sapiens GN=GOLGA3 PE=1 SV=2 | 167251 | 5.1724 | 0.572 | D | Y |
| Q08722 | Leukocyte surface antigen CD47 OS=Homo sapiens GN=CD47 PE=1 SV=1 | 35190 | 6.9844 | 0.241 | D | Y |
| Q10567 | AP-1 complex subunit beta-1 OS=Homo sapiens GN=AP1B1 PE=1 SV=2 | 104570 | 4.749 | 0.107 | D | Y |
| Q12860 | Contactin-1 OS=Homo sapiens GN=CNTN1 PE=1 SV=1 | 113249 | 5.5137 | 0.993 | D | Y |
| Q12907 | Vesicular integral-membrane protein VIP36 OS=Homo sapiens GN=LMAN2 PE=1 SV=1 | 40203 | 6.4893 | 0.059 | D | Y |
| Q12955 | Ankyrin-3 OS=Homo sapiens GN=ANK3 PE=1 SV=3 | 480112 | 6.0396 | 0.130 | D | Y |
| Q13151 | Heterogeneous nuclear ribonucleoprotein A0 OS=Homo sapiens GN=HNRNPA0 PE=1 SV=1 | 30821 | 9.5493 | 0.113 | D | Y |
| Q13423 | NAD(P) transhydrogenase, mitochondrial OS=Homo sapiens GN=NNT PE=1 SV=3 | 113822 | 8.0566 | 0.164 | D | Y |
| Q13509 | Tubulin beta-3 chain OS=Homo sapiens GN=TUBB3 PE=1 SV=2 | 50400 | 4.6392 | 0.856 | D | Y |
| Q13554 | Calcium/calmodulin-dependent protein kinase type II subunit beta OS=Homo sapiens GN=CAMI | 72632 | 6.8818 | 0.632 | D | Y |
| Q13555 | Calcium/calmodulin-dependent protein kinase type II subunit gamma OS=Homo sapiens GN=C/ | 62569 | 7.6714 | 0.062 | D | Y |
| Q13561 | Dynactin subunit 2 OS=Homo sapiens GN=DCTN2 PE=1 SV=4 | 44203 | 4.9248 | 0.132 | D | Y |
| Q13885 | Tubulin beta-2A chain OS=Homo sapiens GN=TUBB2A PE=1 SV=1 | 49874 | 4.5908 | 8.635 | D | Y |
| Q14011 | Cold-inducible RNA-binding protein OS=Homo sapiens GN=CIRBP PE=1 SV=1 | 18636 | 9.7397 | 0.433 | D | Y |
| Q14195 | Dihydropyrimidinase-related protein 3 OS=Homo sapiens GN=DPYSL3 PE=1 SV=1 | 61924 | 6.0249 | 0.332 | D | Y |
| Q14240 | Eukaryotic initiation factor 4A-II OS=Homo sapiens GN=EIF4A2 PE=1 SV=2 | 46372 | 5.1636 | 0.126 | D | Y |
| Q14247 | Src substrate cortactin OS=Homo sapiens GN=CTTN PE=1 SV=2 | 61548 | 5.0771 | 0.089 | D | Y |
| Q14257 | Reticulocalbin-2 OS=Homo sapiens GN=RCN2 PE=1 SV=1 | 36853 | 4.0635 | 0.159 | D | Y |
| Q14667 | Protein KIAA0100 OS=Homo sapiens GN=KIAA0100 PE=1 SV=3 | 253537 | 6.6973 | 1.982 | D | Y |
| Q14894 | Ketimine reductase mu-crystallin OS=Homo sapiens GN=CRYM PE=1 SV=1 | 33754 | 4.8765 | 0.485 | D | Y |
| Q14CZ8 | Hepatocyte cell adhesion molecule OS=Homo sapiens GN=HEPACAM PE=1 SV=1 | 45998 | 9.3999 | 0.447 | D | Y |
| Q15084 | Protein disulfide-isomerase A6 OS=Homo sapiens GN=PDIA6 PE=1 SV=1 | 48091 | 4.7622 | 0.161 | D | Y |
| Q15121 | Astrocytic phosphoprotein PEA-15 OS=Homo sapiens GN=PEA15 PE=1 SV=2 | 15030 | 4.7388 | 0.056 | D | Y |
| Q15286 | Ras-related protein Rab-35 OS=Homo sapiens GN=RAB35 PE=1 SV=1 | 23010 | 8.3936 | 0.142 | D | Y |
| Q15555 | Microtubule-associated protein RP/EB family member 2 OS=Homo sapiens GN=MAPRE2 PE=1 | 37008 | 5.2324 | 0.152 | D | Y |
| Q15717 | ELAV-like protein 1 OS=Homo sapiens GN=ELAVL1 PE=1 SV=2 | 36069 | 9.4702 | 0.223 | D | Y |
| Q15819 | Ubiquitin-conjugating enzyme E2 variant 2 OS=Homo sapiens GN=UBE2V2 PE=1 SV=4 | 16352 | 8.5781 | 0.047 | D | Y |
| Q16143 | Beta-synuclein OS=Homo sapiens GN=SNCB PE=1 SV=1 | 14279 | 4.21 | 0.900 | D | Y |
| Q16555 | Dihydropyrimidinase-related protein 2 OS=Homo sapiens GN=DPYSL2 PE=1 SV=1 | 62254 | 5.9238 | 2.828 | D | Y |
| Q16643 | Drebrin OS=Homo sapiens GN=DBN1 PE=1 SV=4 | 71385 | 4.2026 | 0.189 | D | Y |
| Q16658 | Fascin OS=Homo sapiens GN=FSCN1 PE=1 SV=3 | 54496 | 6.876 | 0.182 | D | Y |
| Q16698 | 2,4-dienoyl-CoA reductase, mitochondrial OS=Homo sapiens GN=DECR1 PE=1 SV=1 | 36044 | 9.6606 | 0.095 | D | Y |
| Q16798 | NADP-dependent malic enzyme, mitochondrial OS=Homo sapiens GN=ME3 PE=2 SV=2 | 67025 | 7.894 | 0.056 | D | Y |
| Q16799 | Reticulon-1 OS=Homo sapiens GN=RTN1 PE=1 SV=1 | 83566 | 4.415 | 0.487 | D | Y |
| Q3L8U1 | Chromodomain-helicase-DNA-binding protein 9 OS=Homo sapiens GN=CHD9 PE=1 SV=2 | 325817 | 6.5493 | 0.770 | D | Y |
| Q58FF6 | Putative heat shock protein HSP 90-beta 4 OS=Homo sapiens GN=HSP90AB4P PE=5 SV=1 | 58227 | 4.4531 | 0.026 | D | Y |
| Q5F2F8 | Serine/threonine-protein phosphatase OS=Homo sapiens GN=PPP3CB PE=1 SV=1 | 55978 | 5.354 | 0.049 | D | Y |
| Q5H928 | 3-hydroxyacyl-CoA dehydrogenase type-2 OS=Homo sapiens GN=HSD17B10 PE=1 SV=1 | 17212 | 7.7329 | 0.102 | D | Y |
| Q5JRA6 | Melanoma inhibitory activity protein 3 OS=Homo sapiens GN=MIA3 PE=1 SV=1 | 213568 | 4.5688 | 0.120 | D | Y |
| Q5JYX0 | Cell division control protein 42 homolog (Fragment) OS=Homo sapiens GN=CDC42 PE=1 SV=1 | 15170 | 5.1416 | 0.082 | D | Y |
| Q5SSV3 | N(G),N(G)-dimethylarginine dimethylaminohydrolase 2 (Fragment) OS=Homo sapiens GN=DDA | 23554 | 5.4478 | 0.060 | D | Y |
| Q5TC82 | Roquin-1 OS=Homo sapiens GN=RC3H1 PE=1 SV=1 | 125657 | 6.8643 | 1.414 | D | Y |
| Q5XKP0 | MICOS complex subunit MIC13 OS=Homo sapiens GN=MIC13 PE=1 SV=1 | 13078 | 9.7148 | 0.102 | D | Y |
| Q6AI14 | Sodium/hydrogen exchanger 4 OS=Homo sapiens GN=SLC9A4 PE=1 SV=2 | 89756 | 8.603 | 1.120 | D | Y |
| Q6DT37 | Serine/threonine-protein kinase MRCK gamma OS=Homo sapiens GN=CDC42BPG PE=1 SV=2 | 172351 | 5.855 | 0.384 | D | Y |
| Q6IQ22 | Ras-related protein Rab-12 OS=Homo sapiens GN=RAB12 PE=1 SV=3 | 27231 | 8.5107 | 0.074 | D | Y |
| Q6PCE3 | Glucose 1,6-bisphosphate synthase OS=Homo sapiens GN=PGM2L1 PE=1 SV=3 | 70396 | 6.8042 | 0.107 | D | Y |
| Q6R327 | Rapamycin-insensitive companion of mTOR OS=Homo sapiens GN=RICTOR PE=1 SV=1 | 192096 | 7.1323 | 0.068 | D | Y |
| Q6S8J3 | POTE ankyrin domain family member E OS=Homo sapiens GN=POTEE PE=1 SV=3 | 121285 | 5.7715 | 0.010 | D | Y |

| | | | | | | |
|---|---|---|---|---|---|---|
| Q6UXG8 | Butyrophilin-like protein 9 OS=Homo sapiens GN=BTNL9 PE=2 SV=1 | 59678 | 5.9839 | 7.086 | D | Y |
| Q6ZN28 | Metastasis-associated in colon cancer protein 1 OS=Homo sapiens GN=MACC1 PE=1 SV=2 | 96577 | 6.4365 | 1.679 | D | Y |
| Q6ZP82 | Coiled-coil domain-containing protein 141 OS=Homo sapiens GN=CCDC141 PE=1 SV=2 | 166156 | 5.2764 | 0.346 | D | Y |
| Q6ZQQ6 | WD repeat-containing protein 87 OS=Homo sapiens GN=WDR87 PE=1 SV=3 | 332973 | 6.9009 | 0.179 | D | Y |
| Q6ZR08 | Dynein heavy chain 12, axonemal OS=Homo sapiens GN=DNAH12 PE=2 SV=2 | 356711 | 5.7744 | 0.104 | D | Y |
| Q6ZU15 | Septin-14 OS=Homo sapiens GN=SEPT14 PE=1 SV=2 | 49993 | 5.8154 | 0.504 | D | Y |
| Q71U36 | Tubulin alpha-1A chain OS=Homo sapiens GN=TUBA1A PE=1 SV=1 | 50103 | 4.7622 | 14.630 | D | Y |
| Q7L099 | Protein RUFY3 OS=Homo sapiens GN=RUFY3 PE=1 SV=1 | 52931 | 5.2002 | 0.529 | D | Y |
| Q7L0J3 | Synaptic vesicle glycoprotein 2A OS=Homo sapiens GN=SV2A PE=1 SV=1 | 82642 | 5.2354 | 0.093 | D | Y |
| Q7L273 | BTB/POZ domain-containing protein KCTD9 OS=Homo sapiens GN=KCTD9 PE=1 SV=1 | 42539 | 5.9209 | 0.360 | D | Y |
| Q86XW9 | Thioredoxin domain-containing protein 6 OS=Homo sapiens GN=NME9 PE=2 SV=1 | 36832 | 4.623 | 1.553 | D | Y |
| Q86Y46 | Keratin, type II cytoskeletal 73 OS=Homo sapiens GN=KRT73 PE=1 SV=1 | 58886 | 6.9932 | 0.269 | D | Y |
| Q8IWA5 | Choline transporter-like protein 2 OS=Homo sapiens GN=SLC44A2 PE=1 SV=3 | 80070 | 8.521 | 0.055 | D | Y |
| Q8IWJ2 | GRIP and coiled-coil domain-containing protein 2 OS=Homo sapiens GN=GCC2 PE=1 SV=4 | 195787 | 4.916 | 0.823 | D | Y |
| Q8N126 | Cell adhesion molecule 3 OS=Homo sapiens GN=CADM3 PE=1 SV=1 | 43272 | 5.6572 | 0.227 | D | Y |
| Q8N3J6 | Cell adhesion molecule 2 OS=Homo sapiens GN=CADM2 PE=2 SV=1 | 47524 | 4.9922 | 0.100 | D | Y |
| Q8NI77 | Kinesin-like protein KIF18A OS=Homo sapiens GN=KIF18A PE=1 SV=2 | 102216 | 9.1245 | 6.949 | D | Y |
| Q8TB36 | Ganglioside-induced differentiation-associated protein 1 OS=Homo sapiens GN=GDAP1 PE=1 S | 41319 | 8.4858 | 0.224 | D | Y |
| Q8WXF1 | Paraspeckle component 1 OS=Homo sapiens GN=PSPC1 PE=1 SV=1 | 58706 | 6.2344 | 0.199 | D | Y |
| Q8WY54 | Protein phosphatase 1E OS=Homo sapiens GN=PPM1E PE=1 SV=2 | 84948 | 4.7666 | 0.079 | D | Y |
| Q92599 | Septin-8 OS=Homo sapiens GN=SEPT8 PE=1 SV=4 | 55721 | 5.8418 | 0.135 | D | Y |
| Q92747 | Actin-related protein 2/3 complex subunit 1A OS=Homo sapiens GN=ARPC1A PE=1 SV=2 | 41542 | 8.1343 | 0.069 | D | Y |
| Q92752 | Tenascin-R OS=Homo sapiens GN=TNR PE=1 SV=3 | 149467 | 4.522 | 0.967 | D | Y |
| Q92777 | Synapsin-2 OS=Homo sapiens GN=SYN2 PE=2 SV=3 | 62807 | 8.5474 | 0.126 | D | Y |
| Q92930 | Ras-related protein Rab-8B OS=Homo sapiens GN=RAB8B PE=1 SV=2 | 23569 | 9.4321 | 0.072 | D | Y |
| Q93050 | V-type proton ATPase 116 kDa subunit a isoform 1 OS=Homo sapiens GN=ATP6V0A1 PE=1 S' | 96350 | 5.981 | 0.061 | D | Y |
| Q96CX2 | BTB/POZ domain-containing protein KCTD12 OS=Homo sapiens GN=KCTD12 PE=1 SV=1 | 35678 | 5.3232 | 0.187 | D | Y |
| Q96E17 | Ras-related protein Rab-3C OS=Homo sapiens GN=RAB3C PE=2 SV=1 | 25935 | 4.9028 | 1.231 | D | Y |
| Q96FC7 | Phytanoyl-CoA hydroxylase-interacting protein-like OS=Homo sapiens GN=PHYHIPL PE=1 SV= | 42458 | 5.9561 | 0.140 | D | Y |
| Q96FJ2 | Dynein light chain 2, cytoplasmic OS=Homo sapiens GN=DYNLL2 PE=1 SV=1 | 10343 | 7.2422 | 0.607 | D | Y |
| Q96GW7 | Brevican core protein OS=Homo sapiens GN=BCAN PE=1 SV=2 | 99056 | 4.377 | 0.528 | D | Y |
| Q96IX5 | Up-regulated during skeletal muscle growth protein 5 OS=Homo sapiens GN=USMG5 PE=1 SV | 6453 | 10.0664 | 0.362 | D | Y |
| Q96JE9 | Microtubule-associated protein 6 OS=Homo sapiens GN=MAP6 PE=1 SV=2 | 86451 | 9.5786 | 0.194 | D | Y |
| Q96KP4 | Cytosolic non-specific dipeptidase OS=Homo sapiens GN=CNDP2 PE=1 SV=2 | 52844 | 5.5679 | 0.189 | D | Y |
| Q96QB1 | Rho GTPase-activating protein 7 OS=Homo sapiens GN=DLC1 PE=1 SV=4 | 170484 | 5.9458 | 1.489 | D | Y |
| Q96QK1 | Vacuolar protein sorting-associated protein 35 OS=Homo sapiens GN=VPS35 PE=1 SV=2 | 91649 | 5.168 | 0.121 | D | Y |
| Q96S86 | Hyaluronan and proteoglycan link protein 3 OS=Homo sapiens GN=HAPLN3 PE=2 SV=1 | 40868 | 6.0645 | 0.102 | D | Y |
| Q99456 | Keratin, type I cytoskeletal 12 OS=Homo sapiens GN=KRT12 PE=1 SV=1 | 53478 | 4.5073 | 0.636 | D | Y |
| Q99497 | Protein deglycase DJ-1 OS=Homo sapiens GN=PARK7 PE=1 SV=2 | 19878 | 6.3721 | 1.084 | D | Y |
| Q99536 | Synaptic vesicle membrane protein VAT-1 homolog OS=Homo sapiens GN=VAT1 PE=1 SV=2 | 41893 | 5.8506 | 0.138 | D | Y |
| Q99747 | Gamma-soluble NSF attachment protein OS=Homo sapiens GN=NAPG PE=1 SV=1 | 34724 | 5.1299 | 0.152 | D | Y |
| Q99798 | Aconitate hydratase, mitochondrial OS=Homo sapiens GN=ACO2 PE=1 SV=2 | 85371 | 7.3286 | 0.988 | D | Y |
| Q99832 | T-complex protein 1 subunit eta OS=Homo sapiens GN=CCT7 PE=1 SV=2 | 59328 | 7.519 | 0.158 | D | Y |
| Q99962 | Endophilin-A1 OS=Homo sapiens GN=SH3GL2 PE=1 SV=1 | 39937 | 5.1636 | 0.145 | D | Y |
| Q9BPW8 | Protein NipSnap homolog 1 OS=Homo sapiens GN=NIPSNAP1 PE=1 SV=1 | 33288 | 9.5581 | 0.203 | D | Y |
| Q9BS92 | Protein NipSnap homolog 3B OS=Homo sapiens GN=NIPSNAP3B PE=2 SV=1 | 28295 | 9.5698 | 0.048 | D | Y |
| Q9BUF5 | Tubulin beta-6 chain OS=Homo sapiens GN=TUBB6 PE=1 SV=1 | 49825 | 4.5791 | 0.952 | D | Y |
| Q9BVA1 | Tubulin beta-2B chain OS=Homo sapiens GN=TUBB2B PE=1 SV=1 | 49920 | 4.5908 | 1.618 | D | Y |
| Q9BW30 | Tubulin polymerization-promoting protein family member 3 OS=Homo sapiens GN=TPPP3 PE= | 18973 | 9.5288 | 3.584 | D | Y |
| Q9BY12 | S phase cyclin A-associated protein in the endoplasmic reticulum OS=Homo sapiens GN=SCAF | 158187 | 7.1191 | 0.725 | D | Y |
| Q9BYX7 | Putative beta-actin-like protein 3 OS=Homo sapiens GN=POTEKP PE=5 SV=1 | 41988 | 5.8843 | 5.307 | D | Y |
| Q9H0C2 | ADP/ATP translocase 4 OS=Homo sapiens GN=SLC25A31 PE=2 SV=1 | 34999 | 10.2378 | 0.357 | D | Y |
| Q9H115 | Beta-soluble NSF attachment protein OS=Homo sapiens GN=NAPB PE=1 SV=2 | 33535 | 5.1694 | 0.289 | D | Y |
| Q9H3Z4 | DnaJ homolog subfamily C member 5 OS=Homo sapiens GN=DNAJC5 PE=1 SV=1 | 22134 | 4.7446 | 0.166 | D | Y |
| Q9H4G0 | Band 4.1-like protein 1 OS=Homo sapiens GN=EPB41L1 PE=1 SV=2 | 98442 | 5.2866 | 0.153 | D | Y |
| Q9H8Y8 | Golgi reassembly-stacking protein 2 OS=Homo sapiens GN=GORASP2 PE=1 SV=3 | 47116 | 4.5337 | 0.046 | D | Y |
| Q9HCD6 | Protein TANC2 OS=Homo sapiens GN=TANC2 PE=1 SV=3 | 219510 | 7.9614 | 0.642 | D | Y |
| Q9HCK8 | Chromodomain-helicase-DNA-binding protein 8 OS=Homo sapiens GN=CHD8 PE=1 SV=5 | 290335 | 6.0132 | 0.064 | D | Y |
| Q9NQC3 | Reticulon-4 OS=Homo sapiens GN=RTN4 PE=1 SV=2 | 129851 | 4.2246 | 0.062 | D | Y |
| Q9NQX4 | Unconventional myosin-Vc OS=Homo sapiens GN=MYO5C PE=1 SV=2 | 202681 | 7.5103 | 0.277 | D | Y |
| Q9NRY4 | Rho GTPase-activating protein 35 OS=Homo sapiens GN=ARHGAP35 PE=1 SV=3 | 170406 | 6.1758 | 3.028 | D | Y |
| Q9NSD9 | Phenylalanine--tRNA ligase beta subunit OS=Homo sapiens GN=FARSB PE=1 SV=3 | 66073 | 6.3955 | 0.097 | D | Y |
| Q9NVJ2 | ADP-ribosylation factor-like protein 8B OS=Homo sapiens GN=ARL8B PE=1 SV=1 | 21525 | 8.6045 | 0.232 | D | Y |
| Q9NY65 | Tubulin alpha-8 chain OS=Homo sapiens GN=TUBA8 PE=1 SV=1 | 50061 | 4.7578 | 0.075 | D | Y |
| Q9NZ45 | CDGSH iron-sulfur domain-containing protein 1 OS=Homo sapiens GN=CISD1 PE=1 SV=1 | 12191 | 9.4468 | 0.190 | D | Y |
| Q9P035 | Very-long-chain (3R)-3-hydroxyacyl-CoA dehydratase 3 OS=Homo sapiens GN=HACD3 PE=1 S | 43131 | 9.1714 | 0.110 | D | Y |
| Q9P2R7 | Succinyl-CoA ligase [ADP-forming] subunit beta, mitochondrial OS=Homo sapiens GN=SUCLA2 | 50285 | 7.2041 | 0.199 | D | Y |
| Q9P2U7 | Vesicular glutamate transporter 1 OS=Homo sapiens GN=SLC17A7 PE=2 SV=1 | 61573 | 7.2012 | 0.391 | D | Y |
| Q9UBB6 | Neurochondrin OS=Homo sapiens GN=NCDN PE=1 SV=1 | 78813 | 5.1812 | 0.116 | D | Y |
| Q9UDY2 | Tight junction protein ZO-2 OS=Homo sapiens GN=TJP2 PE=1 SV=2 | 133876 | 7.0181 | 0.092 | D | Y |
| Q9UFN0 | Protein NipSnap homolog 3A OS=Homo sapiens GN=NIPSNAP3A PE=1 SV=2 | 28448 | 9.4556 | 0.169 | D | Y |
| Q9UIJ7 | GTP:AMP phosphotransferase AK3, mitochondrial OS=Homo sapiens GN=AK3 PE=1 SV=4 | 25549 | 9.5303 | 0.195 | D | Y |
| Q9UJU6 | Drebrin-like protein OS=Homo sapiens GN=DBNL PE=1 SV=1 | 48177 | 4.8208 | 0.079 | D | Y |
| Q9UJZ1 | Stomatin-like protein 2, mitochondrial OS=Homo sapiens GN=STOML2 PE=1 SV=1 | 38510 | 7.2642 | 0.359 | D | Y |
| Q9UK22 | F-box only protein 2 OS=Homo sapiens GN=FBXO2 PE=1 SV=2 | 33306 | 4.0957 | 0.116 | D | Y |
| Q9ULD0 | 2-oxoglutarate dehydrogenase-like, mitochondrial OS=Homo sapiens GN=OGDHL PE=1 SV=3 | 114408 | 6.1758 | 0.102 | D | Y |
| Q9UPY8 | Microtubule-associated protein RP/EB family member 3 OS=Homo sapiens GN=MAPRE3 PE=1 | 31961 | 5.1958 | 0.137 | D | Y |
| Q9UQM7 | Calcium/calmodulin-dependent protein kinase type II subunit alpha OS=Homo sapiens GN=CAM | 54053 | 6.6284 | 1.881 | D | Y |
| Q9Y277 | Voltage-dependent anion-selective channel protein 3 OS=Homo sapiens GN=VDAC3 PE=1 SV= | 30639 | 8.8301 | 2.348 | D | Y |
| Q9Y2J8 | Protein-arginine deiminase type-2 OS=Homo sapiens GN=PADI2 PE=1 SV=2 | 75515 | 5.2588 | 0.188 | D | Y |
| Q9Y696 | Chloride intracellular channel protein 4 OS=Homo sapiens GN=CLIC4 PE=1 SV=4 | 28753 | 5.291 | 0.026 | D | Y |

| | | | | | | |
|---|---|---|---|---|---|---|
| Q9Y6R1 | Electrogenic sodium bicarbonate cotransporter 1 OS=Homo sapiens GN=SLC4A4 PE=1 SV=1 | 121382 | 6.3428 | 0.108 | D | Y |
| A0A024R216 | Hepatoma-derived growth factor, related protein 3, isoform CRA_a OS=Homo sapiens GN=HDC | 22606 | 8.4185 | 0.001 | B | O |
| A0A024R3B9 | Alpha-crystallin B chain OS=Homo sapiens GN=CRYAB PE=1 SV=1 | 12245 | 9.2739 | 0.001 | B | O |
| A0A024RA52 | Proteasome subunit alpha type OS=Homo sapiens GN=PSMA2 PE=1 SV=1 | 25882 | 7.2979 | 0.001 | B | O |
| A0A075B6H6 | Ig kappa chain C region (Fragment) OS=Homo sapiens GN=IGKC PE=1 SV=1 | 11712 | 5.5005 | 0.001 | B | O |
| A0A087WT12 | Glutathione peroxidase OS=Homo sapiens GN=GPX4 PE=1 SV=1 | 26930 | 10.4531 | 0.001 | B | O |
| A0A087WT87 | Amino acid transporter OS=Homo sapiens GN=SLC1A3 PE=1 SV=1 | 54172 | 7.2817 | 0.001 | B | O |
| A0A087WTE4 | Neural cell adhesion molecule 1 OS=Homo sapiens GN=NCAM1 PE=1 SV=1 | 83690 | 4.5542 | 0.001 | B | O |
| A0A087WTF6 | Neural cell adhesion molecule 1 OS=Homo sapiens GN=NCAM1 PE=1 SV=1 | 93271 | 4.585 | 0.979 | B | O |
| A0A087WTH0 | Enolase-phosphatase E1 OS=Homo sapiens GN=ENOPH1 PE=1 SV=2 | 23349 | 4.7783 | 0.001 | B | O |
| A0A087WTI3 | NADH dehydrogenase [ubiquinone] iron-sulfur protein 7, mitochondrial OS=Homo sapiens GN=I | 23715 | 12.1348 | 0.001 | B | O |
| A0A087WTM7 | Apolipoprotein B-100 OS=Homo sapiens GN=APOB PE=1 SV=1 | 489527 | 6.6899 | 0.267 | B | O |
| A0A087WTP3 | Far upstream element-binding protein 2 OS=Homo sapiens GN=KHSRP PE=1 SV=1 | 72982 | 7.5762 | 0.001 | B | O |
| A0A087WTS8 | Heat shock 70 kDa protein 4 OS=Homo sapiens GN=HSPA4 PE=1 SV=1 | 52566 | 8.2705 | 0.098 | B | O |
| A0A087WTT1 | Polyadenylate-binding protein OS=Homo sapiens GN=PABPC1 PE=1 SV=1 | 58498 | 9.5977 | 0.169 | B | O |
| A0A087WTU9 | Alstrom syndrome protein 1 OS=Homo sapiens GN=ALMS1 PE=1 SV=1 | 456137 | 5.8447 | 0.061 | B | O |
| A0A087WUI2 | Heterogeneous nuclear ribonucleoproteins A2/B1 OS=Homo sapiens GN=HNRNPA2B1 PE=1 S | 29816 | 4.7183 | 1.427 | B | O |
| A0A087WUK2 | Heterogeneous nuclear ribonucleoprotein D-like OS=Homo sapiens GN=HNRNPDL PE=1 SV=1 | 40015 | 10.355 | 0.365 | B | O |
| A0A087WUZ3 | Spectrin beta chain, non-erythrocytic 1 OS=Homo sapiens GN=SPTBN1 PE=1 SV=1 | 274657 | 5.2515 | 0.001 | B | O |
| A0A087WV23 | SH3 domain-binding glutamic acid-rich-like protein 3 OS=Homo sapiens GN=SH3BGRL3 PE=1 | 23771 | 9.3442 | 0.001 | B | O |
| A0A087WV47 | Ig gamma-1 chain C region OS=Homo sapiens GN=IGHG1 PE=1 SV=1 | 51121 | 7.3813 | 0.001 | B | O |
| A0A087WVC4 | cAMP-dependent protein kinase catalytic subunit beta OS=Homo sapiens GN=PRKACB PE=1 S | 39211 | 9.1157 | 0.037 | B | O |
| A0A087WVQ6 | Clathrin heavy chain OS=Homo sapiens GN=CLTC PE=1 SV=1 | 191934 | 5.3555 | 0.001 | B | O |
| A0A087WW96 | Synapsin-2 OS=Homo sapiens GN=SYN2 PE=1 SV=1 | 62956 | 8.5474 | 0.214 | B | O |
| A0A087WWU8 | Tropomyosin alpha-3 chain OS=Homo sapiens GN=TPM3 PE=1 SV=1 | 26404 | 4.5513 | 0.044 | B | O |
| A0A087WX08 | Gamma-adducin OS=Homo sapiens GN=ADD3 PE=1 SV=1 | 75328 | 6.2915 | 0.105 | B | O |
| A0A087WXC5 | NADH dehydrogenase [ubiquinone] 1 alpha subcomplex subunit 10, mitochondrial OS=Homo sa | 40811 | 8.5957 | 0.001 | B | O |
| A0A087WXS7 | ATPase ASNA1 OS=Homo sapiens GN=ASNA1 PE=1 SV=1 | 37094 | 4.8369 | 0.040 | B | O |
| A0A087WXX2 | Fructose-bisphosphate aldolase OS=Homo sapiens GN=ALDOB PE=1 SV=1 | 34832 | 8.1636 | 0.024 | B | O |
| A0A087WY71 | AP-2 complex subunit mu OS=Homo sapiens GN=AP2M1 PE=1 SV=1 | 49495 | 9.8848 | 0.109 | B | O |
| A0A087WYG8 | Alpha-internexin OS=Homo sapiens GN=INA PE=1 SV=1 | 55031 | 5.1665 | 0.190 | B | O |
| A0A087WYS1 | UTP--glucose-1-phosphate uridylyltransferase OS=Homo sapiens GN=UGP2 PE=1 SV=1 | 56931 | 7.9468 | 0.001 | B | O |
| A0A087WYT3 | Prostaglandin E synthase 3 OS=Homo sapiens GN=PTGES3 PE=1 SV=1 | 19142 | 4.147 | 0.001 | B | O |
| A0A087WZH7 | Myristoylated alanine-rich C-kinase substrate OS=Homo sapiens GN=MARCKS PE=1 SV=1 | 31577 | 4.5264 | 0.711 | B | O |
| A0A087WZN1 | Isocitrate dehydrogenase [NAD] subunit, mitochondrial OS=Homo sapiens GN=IDH3B PE=1 SV | 42383 | 8.5854 | 0.001 | B | O |
| A0A087WZQ7 | Beta-soluble NSF attachment protein OS=Homo sapiens GN=NAPB PE=1 SV=1 | 33895 | 5.1694 | 0.001 | B | O |
| A0A087WZZ5 | Splicing factor 3B subunit 2 OS=Homo sapiens GN=SF3B2 PE=1 SV=1 | 97524 | 5.3818 | 0.131 | B | O |
| A0A087X027 | Protein SETSIP OS=Homo sapiens GN=SETSIP PE=3 SV=1 | 33624 | 4.0034 | 0.483 | B | O |
| A0A087X054 | Hypoxia up-regulated protein 1 OS=Homo sapiens GN=HYOU1 PE=1 SV=1 | 104713 | 5.4243 | 0.153 | B | O |
| A0A087X0X3 | Heterogeneous nuclear ribonucleoprotein M OS=Homo sapiens GN=HNRNPM PE=1 SV=1 | 77518 | 9.1436 | 0.001 | B | O |
| A0A087X142 | Septin-8 OS=Homo sapiens GN=SEPT8 PE=1 SV=1 | 49325 | 5.7847 | 0.111 | B | O |
| A0A087X1H6 | Hsc70-interacting protein OS=Homo sapiens GN=ST13 PE=1 SV=1 | 28220 | 9.0073 | 0.001 | B | O |
| A0A087X253 | AP-2 complex subunit beta OS=Homo sapiens GN=AP2B1 PE=1 SV=1 | 101268 | 4.9644 | 0.084 | B | O |
| A0A087X2B1 | RNA binding protein fox-1 homolog OS=Homo sapiens GN=RBFOX1 PE=4 SV=1 | 40284 | 6.624 | 0.057 | B | O |
| A0A087X2D0 | Serine/arginine-rich-splicing factor 3 OS=Homo sapiens GN=SRSF3 PE=1 SV=1 | 10313 | 4.7959 | 0.322 | B | O |
| A0A087X2E3 | Synapsin-2 OS=Homo sapiens GN=SYN2 PE=1 SV=1 | 52277 | 7.6187 | 0.013 | B | O |
| A0A087X2G1 | ATP-dependent RNA helicase DDX1 OS=Homo sapiens GN=DDX1 PE=1 SV=1 | 73928 | 7.5747 | 0.001 | B | O |
| A0A087X2H1 | E3 ubiquitin-protein ligase HECTD1 OS=Homo sapiens GN=HECTD1 PE=1 SV=1 | 289449 | 5.1064 | 0.001 | B | O |
| A0A096LP12 | ES1 protein homolog, mitochondrial (Fragment) OS=Homo sapiens GN=LOC102724023 PE=4 S | 23849 | 9.4292 | 0.161 | B | O |
| A0A0A0MR57 | Zinc finger protein 836 OS=Homo sapiens GN=ZNF836 PE=1 SV=1 | 107659 | 9.3853 | 0.001 | B | O |
| A0A0A0MRA8 | Band 4.1-like protein 3 OS=Homo sapiens GN=EPB41L3 PE=1 SV=1 | 102225 | 5.0361 | 0.001 | B | O |
| A0A0A0MRJ6 | Protein-L-isoaspartate O-methyltransferase OS=Homo sapiens GN=PCMT1 PE=1 SV=1 | 30295 | 7.314 | 0.001 | B | O |
| A0A0A0MS41 | Sideroflexin OS=Homo sapiens GN=SFXN3 PE=1 SV=1 | 35480 | 9.3223 | 0.134 | B | O |
| A0A0A0MS51 | Gelsolin OS=Homo sapiens GN=GSN PE=1 SV=1 | 82474 | 5.2896 | 0.436 | B | O |
| A0A0A0MS87 | Protein NDRG2 OS=Homo sapiens GN=NDRG2 PE=1 SV=1 | 39519 | 6.5845 | 0.166 | B | O |
| A0A0A0MSE2 | Hydroxyacyl-coenzyme A dehydrogenase, mitochondrial OS=Homo sapiens GN=HADH PE=1 S | 42096 | 9.6475 | 0.001 | B | O |
| A0A0A0MSI0 | Peroxiredoxin-1 (Fragment) OS=Homo sapiens GN=PRDX1 PE=1 SV=1 | 18963 | 6.4907 | 1.534 | B | O |
| A0A0A0MSM0 | Heat shock protein 105 kDa OS=Homo sapiens GN=HSPH1 PE=1 SV=1 | 88785 | 5.3101 | 0.010 | B | O |
| A0A0A0MSQ8 | Protein WWC3 OS=Homo sapiens GN=WWC3 PE=1 SV=1 | 136564 | 5.9897 | 0.020 | B | O |
| A0A0A0MSY9 | Histone-lysine N-methyltransferase EZH1 OS=Homo sapiens GN=EZH1 PE=1 SV=1 | 84222 | 7.3784 | 0.001 | B | O |
| A0A0A0MT26 | Sodium/potassium-transporting ATPase subunit alpha-3 OS=Homo sapiens GN=ATP1A3 PE=1 | 133231 | 5.6865 | 0.001 | B | O |
| A0A0A0MTI5 | Acyl-CoA-binding protein OS=Homo sapiens GN=DBI PE=1 SV=1 | 15948 | 4.7886 | 0.001 | B | O |
| A0A0A0MTJ9 | Neutral cholesterol ester hydrolase 1 OS=Homo sapiens GN=NCEH1 PE=1 SV=1 | 49849 | 6.8306 | 0.001 | B | O |
| A0A0A0MTN3 | Glutathione S-transferase Mu 3 OS=Homo sapiens GN=GSTM3 PE=1 SV=1 | 24898 | 5.0156 | 0.043 | B | O |
| A0A0A0MTR1 | Cadherin-13 OS=Homo sapiens GN=CDH13 PE=1 SV=2 | 76921 | 4.585 | 0.118 | B | O |
| A0A0A0MTS2 | Glucose-6-phosphate isomerase (Fragment) OS=Homo sapiens GN=GPI PE=1 SV=1 | 64784 | 9.3516 | 0.001 | B | O |
| A0A0A6YYC0 | Ribosomal protein S6 kinase alpha-4 (Fragment) OS=Homo sapiens GN=RPS6KA4 PE=1 SV=1 | 78317 | 7.3594 | 0.186 | B | O |
| A0A0A6YYG9 | Protein ARPC4-TTLL3 OS=Homo sapiens GN=ARPC4-TTLL3 PE=4 SV=1 | 71672 | 5.4873 | 0.300 | B | O |
| A0A0B4J1R6 | Transketolase OS=Homo sapiens GN=TKT PE=1 SV=1 | 49878 | 7.8545 | 0.330 | B | O |
| A0A0B4J213 | 60S ribosomal protein L30 OS=Homo sapiens GN=RPL30 PE=1 SV=1 | 5551 | 6.0483 | 0.114 | B | O |
| A0A0B4J2A2 | Peptidyl-prolyl cis-trans isomerase A-like 4C OS=Homo sapiens GN=PPIAL4C PE=2 SV=1 | 18144 | 9.6167 | 0.004 | B | O |
| A0A0B4J2C3 | Translationally-controlled tumor protein OS=Homo sapiens GN=TPT1 PE=1 SV=1 | 22559 | 4.9395 | 0.001 | B | O |
| A0A0C4DFU1 | Superoxide dismutase OS=Homo sapiens GN=SOD2 PE=1 SV=1 | 20710 | 8.4067 | 0.001 | B | O |
| A0A0C4DFU2 | Superoxide dismutase OS=Homo sapiens GN=SOD2 PE=1 SV=1 | 24734 | 8.4038 | 0.001 | B | O |
| A0A0C4DFV9 | Protein SET OS=Homo sapiens GN=SET PE=1 SV=1 | 31105 | 3.9272 | 0.937 | B | O |
| A0A0C4DFX2 | Protein furry homolog OS=Homo sapiens GN=FRY PE=1 SV=1 | 337961 | 5.6206 | 0.017 | B | O |
| A0A0C4DFZ1 | Syntaxin-1A OS=Homo sapiens GN=STX1A PE=1 SV=1 | 29554 | 4.5835 | 0.122 | B | O |
| A0A0C4DG17 | 40S ribosomal protein SA OS=Homo sapiens GN=RPSA PE=1 SV=1 | 33292 | 4.5938 | 0.001 | B | O |
| A0A0C4DGI3 | Citrate synthase OS=Homo sapiens GN=CS PE=1 SV=1 | 44674 | 6.7427 | 0.001 | B | O |
| A0A0C4DGK3 | Nesprin-2 OS=Homo sapiens GN=SYNE2 PE=1 SV=1 | 408472 | 4.938 | 0.576 | B | O |
| A0A0C4DGP7 | Nicalin (Fragment) OS=Homo sapiens GN=NCLN PE=1 SV=1 | 39921 | 8.8374 | 0.001 | B | O |

| | | | | | | |
|---|---|---|---|---|---|---|
| A0A0C4DGS0 | NADH dehydrogenase [ubiquinone] 1 alpha subcomplex subunit 6 OS=Homo sapiens GN=NDU | 15126 | 10.4033 | 0.187 | B | O |
| A0A0C4DGZ9 | Tripeptidyl-peptidase 1 OS=Homo sapiens GN=TPP1 PE=1 SV=1 | 60420 | 5.9268 | 0.125 | B | O |
| A0A0C4DH22 | Band 4.1-like protein 1 OS=Homo sapiens GN=EPB41L1 PE=1 SV=1 | 98314 | 5.2866 | 0.072 | B | O |
| A0A0C4DH83 | EH domain-containing protein 3 OS=Homo sapiens GN=EHD3 PE=1 SV=1 | 61857 | 6.0439 | 0.001 | B | O |
| A0A0D9SF30 | Neural cell adhesion molecule 1 (Fragment) OS=Homo sapiens GN=NCAM1 PE=1 SV=1 | 47099 | 4.3975 | 0.172 | B | O |
| A0A0D9SF53 | ATP-dependent RNA helicase DDX3X OS=Homo sapiens GN=DDX3X PE=1 SV=1 | 81426 | 8.0039 | 0.001 | B | O |
| A0A0D9SF54 | Spectrin alpha chain, non-erythrocytic 1 OS=Homo sapiens GN=SPTAN1 PE=1 SV=1 | 282660 | 5.0493 | 1.424 | B | O |
| A0A0D9SFB1 | Dynamin-1 OS=Homo sapiens GN=DNM1 PE=1 SV=1 | 93958 | 6.1934 | 0.383 | B | O |
| A0A0D9SFL3 | RNA-binding protein EWS OS=Homo sapiens GN=EWSR1 PE=1 SV=1 | 61376 | 9.2607 | 0.001 | B | O |
| A0A0D9SG04 | Cordon-bleu protein-like 1 OS=Homo sapiens GN=COBLL1 PE=1 SV=1 | 130090 | 5.9517 | 0.507 | B | O |
| A0A0D9SGF6 | Spectrin alpha chain, non-erythrocytic 1 OS=Homo sapiens GN=SPTAN1 PE=1 SV=1 | 287426 | 5.0728 | 1.898 | B | O |
| A0A0G2JI36 | HLA class I histocompatibility antigen, A-3 alpha chain OS=Homo sapiens GN=HLA-A PE=1 SV | 34194 | 5.792 | 0.069 | B | O |
| A0A0G2JLG5 | Acylglycerol kinase, mitochondrial (Fragment) OS=Homo sapiens GN=AGK PE=1 SV=1 | 25713 | 6.3853 | 0.030 | B | O |
| A0A0G2JLL6 | PAXIP1-associated glutamate-rich protein 1 OS=Homo sapiens GN=PAGR1 PE=4 SV=1 | 36810 | 5.1284 | 0.076 | B | O |
| A0A0G2JMX7 | Microtubule-associated protein OS=Homo sapiens GN=MAPT PE=1 SV=1 | 80865 | 6.6577 | 0.001 | B | O |
| A0A0G2JNQ3 | Heterogeneous nuclear ribonucleoprotein C-like 2 OS=Homo sapiens GN=HNRNPCL2 PE=4 S | 32033 | 5.1021 | 0.001 | B | O |
| A0A0G2JS82 | AP-2 complex subunit alpha-2 (Fragment) OS=Homo sapiens GN=AP2A2 PE=1 SV=1 | 101588 | 6.4189 | 0.103 | B | O |
| A0A0J9YWF7 | Alpha-aminoadipic semialdehyde dehydrogenase OS=Homo sapiens GN=ALDH7A1 PE=4 SV= | 32064 | 7.9072 | 0.001 | B | O |
| A0A0J9YWK4 | Hemoglobin subunit beta OS=Homo sapiens GN=HBB PE=4 SV=1 | 5987 | 7.0122 | 4.192 | B | O |
| A0A0J9YX62 | DnaJ homolog subfamily B member 6 OS=Homo sapiens GN=DNAJB6 PE=4 SV=1 | 36634 | 6.7852 | 0.001 | B | O |
| A0A0J9YX66 | CUGBP Elav-like family member 2 OS=Homo sapiens GN=CELF2 PE=4 SV=1 | 54879 | 8.9297 | 0.001 | B | O |
| A0A0J9YY01 | Unconventional myosin-XVB OS=Homo sapiens GN=MYO15B PE=4 SV=1 | 333513 | 7.481 | 1.304 | B | O |
| A2A2D0 | Stathmin (Fragment) OS=Homo sapiens GN=STMN1 PE=1 SV=6 | 9787 | 7.7842 | 0.076 | B | O |
| A6NG10 | WW domain-binding protein 2 OS=Homo sapiens GN=WBP2 PE=1 SV=2 | 25790 | 5.6279 | 0.001 | B | O |
| A6NL76 | Actin, alpha skeletal muscle OS=Homo sapiens GN=ACTA1 PE=1 SV=3 | 28147 | 5.644 | 0.001 | B | O |
| A6NMH8 | Tetraspanin OS=Homo sapiens GN=CD81 PE=1 SV=1 | 29786 | 6.5288 | 0.001 | B | O |
| A6NMQ3 | Alpha-endosulfine OS=Homo sapiens GN=ENSA PE=1 SV=2 | 15624 | 8.9502 | 0.001 | B | O |
| A6NMY6 | Putative annexin A2-like protein OS=Homo sapiens GN=ANXA2P2 PE=5 SV=2 | 38634 | 6.5479 | 0.001 | B | O |
| A6NP52 | PRA1 family protein 2 OS=Homo sapiens GN=PRAF2 PE=1 SV=1 | 17177 | 8.9077 | 0.193 | B | O |
| A6PVN3 | Carnitine O-acetyltransferase OS=Homo sapiens GN=CRAT PE=1 SV=1 | 18221 | 8.8403 | 0.001 | B | O |
| A6PVN7 | Serine/threonine-protein phosphatase 2A activator (Fragment) OS=Homo sapiens GN=PPP2R4 | 7395 | 9.23 | 0.022 | B | O |
| A8MUS3 | 60S ribosomal protein L23a OS=Homo sapiens GN=RPL23A PE=1 SV=1 | 21902 | 10.916 | 0.001 | B | O |
| A8MXB9 | Sulfatase-modifying factor 2 OS=Homo sapiens GN=SUMF2 PE=1 SV=2 | 33973 | 9.3574 | 0.001 | B | O |
| A8MXP9 | Matrin-3 OS=Homo sapiens GN=MATR3 PE=1 SV=1 | 99905 | 5.6309 | 0.001 | B | O |
| A8MZH3 | Myelin basic protein OS=Homo sapiens GN=MBP PE=1 SV=1 | 19717 | 11.7583 | 0.001 | B | O |
| B1AHD1 | Protein SNU13 OS=Homo sapiens GN=SNU13 PE=1 SV=1 | 14617 | 8.6323 | 0.001 | B | O |
| B1AHR1 | Neuronal-specific septin-3 OS=Homo sapiens GN=SEPT3 PE=1 SV=1 | 33580 | 6.5142 | 0.252 | B | O |
| B1AKD8 | Rootletin (Fragment) OS=Homo sapiens GN=CROCC PE=1 SV=2 | 149086 | 5.1563 | 5.920 | B | O |
| B1AKR6 | Dynein light chain roadblock-type 1 OS=Homo sapiens GN=DYNLRB1 PE=1 SV=1 | 16242 | 6.8276 | 0.001 | B | O |
| B1AKY9 | Sodium/potassium-transporting ATPase subunit alpha OS=Homo sapiens GN=ATP1A2 PE=1 S | 110791 | 5.2749 | 0.095 | B | O |
| B1AKZ5 | Astrocytic phosphoprotein PEA-15 OS=Homo sapiens GN=PEA15 PE=1 SV=1 | 12522 | 4.897 | 0.472 | B | O |
| B1ALC2 | Zinc finger protein 618 (Fragment) OS=Homo sapiens GN=ZNF618 PE=1 SV=6 | 33595 | 6.7148 | 0.054 | B | O |
| B1ALJ5 | Uncharacterized protein C1orf194 OS=Homo sapiens GN=C1orf194 PE=4 SV=1 | 14677 | 9.6592 | 0.029 | B | O |
| B1AMS2 | Septin 6, isoform CRA_b OS=Homo sapiens GN=SEPT6 PE=1 SV=1 | 49272 | 6.3589 | 0.749 | B | O |
| B1ANG9 | Guanylate kinase OS=Homo sapiens GN=GUK1 PE=1 SV=2 | 25320 | 7.3228 | 0.001 | B | O |
| B1B1G2 | Myelin proteolipid protein (Fragment) OS=Homo sapiens GN=PLP1 PE=1 SV=6 | 12203 | 5.4258 | 0.456 | B | O |
| B2R4S9 | Histone H2B OS=Homo sapiens GN=HIST1H2BC PE=2 SV=1 | 13897 | 10.7402 | 0.001 | B | O |
| B2R5W2 | Heterogeneous nuclear ribonucleoproteins C1/C2 OS=Homo sapiens GN=HNRNPC PE=1 SV= | 31928 | 4.9087 | 0.243 | B | O |
| B3KVD8 | Synaptoporin OS=Homo sapiens GN=SYNPR PE=1 SV=1 | 30508 | 6.1611 | 0.001 | B | O |
| B4DEB1 | Histone H3 OS=Homo sapiens GN=H3F3A PE=1 SV=1 | 14043 | 11.7466 | 0.001 | B | O |
| B4DJ62 | HCG2002594, isoform CRA_a OS=Homo sapiens GN=SEPT5 PE=1 SV=1 | 37382 | 5.6616 | 0.001 | B | O |
| B4DJ81 | NADH-ubiquinone oxidoreductase 75 kDa subunit, mitochondrial OS=Homo sapiens GN=NDUF | 66879 | 5.0303 | 0.118 | B | O |
| B4DJV2 | Citrate synthase OS=Homo sapiens GN=CS PE=1 SV=1 | 50399 | 7.8604 | 0.212 | B | O |
| B4DLR8 | NAD(P)H dehydrogenase [quinone] 1 OS=Homo sapiens GN=NQO1 PE=1 SV=1 | 22778 | 8.9531 | 0.001 | B | O |
| B4DUR8 | T-complex protein 1 subunit gamma OS=Homo sapiens GN=CCT3 PE=1 SV=1 | 55638 | 5.3145 | 0.074 | B | O |
| B4DV12 | Polyubiquitin-B OS=Homo sapiens GN=UBB PE=1 SV=1 | 17204 | 7.3169 | 0.001 | B | O |
| B4DV51 | GTP-binding nuclear protein Ran OS=Homo sapiens GN=RAN PE=1 SV=1 | 14721 | 5.8682 | 0.333 | B | O |
| B4DYP1 | N(G),N(G)-dimethylarginine dimethylaminohydrolase 1 OS=Homo sapiens GN=DDAH1 PE=2 S | 20521 | 5.2456 | 0.376 | B | O |
| B4E3L3 | Ubiquitin carboxyl-terminal hydrolase OS=Homo sapiens GN=USP28 PE=1 SV=1 | 105750 | 5.0815 | 0.717 | B | O |
| B5MCD7 | Synaptogyrin-1 OS=Homo sapiens GN=SYNGR1 PE=1 SV=1 | 18453 | 6.8643 | 0.251 | B | O |
| B5MCP9 | 40S ribosomal protein S7 OS=Homo sapiens GN=RPS7 PE=1 SV=1 | 21298 | 10.7769 | 0.161 | B | O |
| B5MD38 | Trifunctional enzyme subunit beta, mitochondrial OS=Homo sapiens GN=HADHB PE=1 SV=1 | 37899 | 9.7866 | 0.155 | B | O |
| B7Z2R2 | Cytochrome b-c1 complex subunit 7 OS=Homo sapiens GN=UQCRB PE=1 SV=1 | 18725 | 9.3164 | 0.001 | B | O |
| B7Z4M1 | Reticulon OS=Homo sapiens GN=RTN3 PE=1 SV=1 | 12738 | 7.8545 | 0.098 | B | O |
| B7Z613 | Neuronal membrane glycoprotein M6-b OS=Homo sapiens GN=GPM6B PE=1 SV=1 | 33250 | 5.9575 | 0.350 | B | O |
| B7Z836 | Abl interactor 2 OS=Homo sapiens GN=ABI2 PE=1 SV=1 | 43061 | 7.5498 | 8.098 | B | O |
| B7Z9C2 | Nucleosome assembly protein 1-like 1 OS=Homo sapiens GN=NAP1L1 PE=1 SV=1 | 40486 | 4.2686 | 0.001 | B | O |
| B7ZAR1 | T-complex protein 1 subunit epsilon OS=Homo sapiens GN=CCT5 PE=1 SV=1 | 55313 | 5.1577 | 0.075 | B | O |
| B8ZZ51 | Malate dehydrogenase, cytoplasmic OS=Homo sapiens GN=MDH1 PE=1 SV=1 | 18677 | 5.5532 | 0.443 | B | O |
| B8ZZB8 | CB1 cannabinoid receptor-interacting protein 1 OS=Homo sapiens GN=CNRIP1 PE=1 SV=1 | 15158 | 9.7544 | 0.454 | B | O |
| B8ZZP4 | Secernin-1 OS=Homo sapiens GN=SCRN1 PE=1 SV=1 | 15631 | 5.2822 | 0.173 | B | O |
| B9A041 | Malate dehydrogenase, cytoplasmic OS=Homo sapiens GN=MDH1 PE=1 SV=1 | 23023 | 7.2305 | 1.009 | B | O |
| B9A067 | MICOS complex subunit MIC60 OS=Homo sapiens GN=IMMT PE=1 SV=2 | 78925 | 6.6357 | 0.277 | B | O |
| C9IZG4 | Protein CutA OS=Homo sapiens GN=CUTA PE=1 SV=1 | 14391 | 5.1709 | 0.048 | B | O |
| C9IZN5 | NADH dehydrogenase [ubiquinone] 1 alpha subcomplex subunit 5 (Fragment) OS=Homo sapier | 10687 | 5.5781 | 0.001 | B | O |
| C9J0D1 | Histone H2A OS=Homo sapiens GN=H2AFV PE=3 SV=1 | 13155 | 10.3301 | 0.686 | B | O |
| C9J0J7 | Profilin-2 OS=Homo sapiens GN=PFN2 PE=1 SV=1 | 9834 | 9.5332 | 0.929 | B | O |
| C9J0K6 | Sorcin OS=Homo sapiens GN=SRI PE=1 SV=1 | 17593 | 5.1929 | 0.277 | B | O |
| C9J1V9 | HCG2043275 OS=Homo sapiens GN=EEF1E1-BLOC1S5 PE=4 SV=2 | 17007 | 7.7388 | 0.001 | B | O |
| C9J1Z8 | ADP-ribosylation factor 5 (Fragment) OS=Homo sapiens GN=ARF5 PE=1 SV=1 | 17095 | 7.3125 | 0.001 | B | O |
| C9J2N0 | Profilin OS=Homo sapiens GN=PFN2 PE=1 SV=1 | 13551 | 5.644 | 0.001 | B | O |

| | | | | | | |
|---|---|---|---|---|---|---|
| C9J8Q5 | Succinate-semialdehyde dehydrogenase, mitochondrial OS=Homo sapiens GN=ALDH5A1 PE= | 53833 | 8.127 | 0.054 | B | O |
| C9J9W2 | LIM and SH3 domain protein 1 (Fragment) OS=Homo sapiens GN=LASP1 PE=1 SV=1 | 18968 | 9.3428 | 0.098 | B | O |
| C9JFR7 | Cytochrome c (Fragment) OS=Homo sapiens GN=CYCS PE=1 SV=1 | 11325 | 10.0488 | 0.571 | B | O |
| C9JIZ0 | LSM8 homolog, U6 small nuclear RNA associated (S. cerevisiae), isoform CRA_a OS=Homo sa | 8104 | 4.0005 | 0.082 | B | O |
| C9JIZ6 | Prosaposin OS=Homo sapiens GN=PSAP PE=1 SV=2 | 58402 | 4.8853 | 0.001 | B | O |
| C9JJT5 | Protein ATP5J2-PTCD1 OS=Homo sapiens GN=ATP5J2-PTCD1 PE=4 SV=2 | 5911 | 9.7969 | 0.379 | B | O |
| C9JL73 | V-type proton ATPase subunit B, kidney isoform OS=Homo sapiens GN=ATP6V1B1 PE=1 SV= | 55046 | 5.3086 | 0.079 | B | O |
| C9JLI6 | 40S ribosomal protein S27 OS=Homo sapiens GN=RPS27L PE=1 SV=1 | 10878 | 8.2866 | 0.001 | B | O |
| C9JQS3 | Syntaxin-binding protein 5-like (Fragment) OS=Homo sapiens GN=STXBP5L PE=1 SV=1 | 125766 | 6.2754 | 0.178 | B | O |
| C9JQS9 | Propionyl-CoA carboxylase beta chain, mitochondrial OS=Homo sapiens GN=PCCB PE=1 SV= | 60698 | 7.894 | 0.001 | B | O |
| C9JQU6 | PRA1 family protein 3 OS=Homo sapiens GN=ARL6IP5 PE=1 SV=1 | 10244 | 10.0225 | 0.001 | B | O |
| C9JRD2 | DnaJ homolog subfamily B member 2 (Fragment) OS=Homo sapiens GN=DNAJB2 PE=1 SV=1 | 25395 | 5.3965 | 0.090 | B | O |
| C9JRZ6 | MICOS complex subunit MIC19 OS=Homo sapiens GN=CHCHD3 PE=1 SV=1 | 26677 | 8.6221 | 0.001 | B | O |
| C9JU14 | Ras-related protein Rab-6B (Fragment) OS=Homo sapiens GN=RAB6B PE=1 SV=3 | 9660 | 5.0303 | 0.355 | B | O |
| C9JXQ9 | NADH dehydrogenase [ubiquinone] 1 beta subcomplex subunit 4 OS=Homo sapiens GN=NDUF | 9530 | 10.0576 | 0.104 | B | O |
| C9JYY6 | Neuronal cell adhesion molecule OS=Homo sapiens GN=NRCAM PE=1 SV=3 | 133729 | 5.4082 | 0.295 | B | O |
| D3YTA9 | Calcineurin subunit B type 1 OS=Homo sapiens GN=PPP3R1 PE=1 SV=1 | 21431 | 4.6377 | 0.001 | B | O |
| D6R904 | Tropomyosin alpha-3 chain OS=Homo sapiens GN=TPM3 PE=1 SV=1 | 11010 | 4.5688 | 0.378 | B | O |
| D6R956 | Ubiquitin carboxyl-terminal hydrolase OS=Homo sapiens GN=UCHL1 PE=1 SV=1 | 26822 | 5.4434 | 0.001 | B | O |
| D6R9D2 | Neuronal membrane glycoprotein M6-a (Fragment) OS=Homo sapiens GN=GPM6A PE=1 SV=1 | 23986 | 4.7476 | 0.494 | B | O |
| D6R9Z7 | Cytochrome c oxidase subunit 7C, mitochondrial OS=Homo sapiens GN=COX7C PE=1 SV=1 | 6378 | 10.1997 | 0.176 | B | O |
| D6RA31 | Alpha-synuclein (Fragment) OS=Homo sapiens GN=SNCA PE=1 SV=6 | 6815 | 9.8467 | 0.587 | B | O |
| D6RAN4 | 60S ribosomal protein L9 (Fragment) OS=Homo sapiens GN=RPL9 PE=1 SV=6 | 20762 | 10.6172 | 0.001 | B | O |
| D6RC06 | Histidine triad nucleotide-binding protein 1 OS=Homo sapiens GN=HINT1 PE=1 SV=1 | 7318 | 10.043 | 0.491 | B | O |
| D6RDG7 | Sideroflexin-1 (Fragment) OS=Homo sapiens GN=SFXN1 PE=1 SV=1 | 16544 | 7.3931 | 0.151 | B | O |
| D6RDU5 | Septin-11 (Fragment) OS=Homo sapiens GN=SEPT11 PE=1 SV=1 | 43085 | 5.8755 | 0.182 | B | O |
| D6RE83 | Ubiquitin carboxyl-terminal hydrolase OS=Homo sapiens GN=UCHL1 PE=1 SV=1 | 23159 | 5.1812 | 0.590 | B | O |
| D6RER5 | Septin-11 OS=Homo sapiens GN=SEPT11 PE=1 SV=1 | 49777 | 6.23 | 0.001 | B | O |
| D6RFH4 | Cytochrome b5 type B OS=Homo sapiens GN=CYB5B PE=1 SV=1 | 14836 | 5.7437 | 0.060 | B | O |
| D6RFM5 | Succinate dehydrogenase [ubiquinone] flavoprotein subunit, mitochondrial OS=Homo sapiens G | 63526 | 6.8394 | 0.055 | B | O |
| D6RGZ6 | Versican core protein (Fragment) OS=Homo sapiens GN=VCAN PE=1 SV=1 | 135660 | 4.2305 | 0.001 | B | O |
| E5RGN3 | Copper transport protein ATOX1 OS=Homo sapiens GN=ATOX1 PE=1 SV=1 | 6326 | 7.9966 | 0.001 | B | O |
| E5RHG6 | Tubulin-specific chaperone A OS=Homo sapiens GN=TBCA PE=1 SV=2 | 15052 | 4.9189 | 0.001 | B | O |
| E5RHP7 | Carbonic anhydrase 1 (Fragment) OS=Homo sapiens GN=CA1 PE=1 SV=1 | 27736 | 6.1187 | 0.176 | B | O |
| E5RID5 | Carbonic anhydrase 2 OS=Homo sapiens GN=CA2 PE=1 SV=1 | 10940 | 5.8271 | 0.319 | B | O |
| E5RJ29 | PH and SEC7 domain-containing protein 3 OS=Homo sapiens GN=PSD3 PE=1 SV=1 | 108923 | 5.6528 | 0.072 | B | O |
| E5RJR5 | S-phase kinase-associated protein 1 OS=Homo sapiens GN=SKP1 PE=1 SV=1 | 18708 | 4.3696 | 0.001 | B | O |
| E5RJU9 | Protein LYRIC OS=Homo sapiens GN=MTDH PE=1 SV=1 | 57486 | 10.0298 | 0.108 | B | O |
| E5RJZ1 | Cytochrome c oxidase subunit 7A-related protein, mitochondrial OS=Homo sapiens GN=COX7A | 8834 | 9.4189 | 0.078 | B | O |
| E7EMB3 | Calmodulin OS=Homo sapiens GN=CALM2 PE=1 SV=1 | 21675 | 4.2524 | 0.001 | B | O |
| E7EMM4 | Acid ceramidase OS=Homo sapiens GN=ASAH1 PE=1 SV=1 | 41769 | 8.1343 | 0.183 | B | O |
| E7EMV2 | Neurofilament medium polypeptide OS=Homo sapiens GN=NEFM PE=1 SV=1 | 78834 | 4.5688 | 0.270 | B | O |
| E7ENJ6 | AP-1 complex subunit mu-1 OS=Homo sapiens GN=AP1M1 PE=1 SV=1 | 42608 | 6.186 | 0.001 | B | O |
| E7ENQ6 | Uncharacterized protein OS=Homo sapiens PE=4 SV=1 | 30075 | 6.5039 | 0.814 | B | O |
| E7ENY0 | Alpha-adducin OS=Homo sapiens GN=ADD1 PE=1 SV=1 | 73358 | 6.0938 | 0.001 | B | O |
| E7EPB3 | 60S ribosomal protein L14 OS=Homo sapiens GN=RPL14 PE=1 SV=1 | 14548 | 10.6597 | 0.122 | B | O |
| E7EPK1 | Septin-7 OS=Homo sapiens GN=SEPT7 PE=1 SV=2 | 50662 | 8.8887 | 0.001 | B | O |
| E7EPT4 | NADH dehydrogenase [ubiquinone] flavoprotein 2, mitochondrial OS=Homo sapiens GN=NDUF | 27889 | 7.9966 | 0.001 | B | O |
| E7EPV7 | Alpha-synuclein OS=Homo sapiens GN=SNCA PE=1 SV=1 | 11769 | 9.7354 | 0.944 | B | O |
| E7EQB2 | Lactotransferrin (Fragment) OS=Homo sapiens GN=LTF PE=1 SV=1 | 76576 | 7.894 | 0.011 | B | O |
| E7EQB8 | Isocitrate dehydrogenase [NAD] subunit, mitochondrial OS=Homo sapiens GN=IDH3G PE=1 SV | 37043 | 8.7671 | 0.001 | B | O |
| E7EQR4 | Ezrin OS=Homo sapiens GN=EZR PE=1 SV=3 | 69328 | 5.7876 | 0.001 | B | O |
| E7ER68 | Protein FAM91A1 OS=Homo sapiens GN=FAM91A1 PE=1 SV=1 | 88931 | 6.2842 | 0.024 | B | O |
| E7ESC9 | WD repeat-containing protein 17 OS=Homo sapiens GN=WDR17 PE=1 SV=2 | 144810 | 5.9282 | 0.214 | B | O |
| E7ESP9 | Neurofilament medium polypeptide OS=Homo sapiens GN=NEFM PE=1 SV=1 | 98322 | 4.6523 | 0.390 | B | O |
| E7ESZ3 | Pleckstrin homology domain-containing family G member 2 OS=Homo sapiens GN=PLEKHG2 F | 133349 | 5.1064 | 0.001 | B | O |
| E7ETC2 | Serine/threonine-protein phosphatase OS=Homo sapiens GN=PPP3CA PE=1 SV=1 | 47784 | 5.3804 | 0.096 | B | O |
| E7EU96 | Casein kinase II subunit alpha OS=Homo sapiens GN=CSNK2A1 PE=1 SV=1 | 45281 | 7.9512 | 0.067 | B | O |
| E7EVA0 | Microtubule-associated protein OS=Homo sapiens GN=MAP4 PE=1 SV=1 | 245289 | 5.833 | 0.001 | B | O |
| E7EVC7 | Autophagy-related protein 16-1 OS=Homo sapiens GN=ATG16L1 PE=1 SV=1 | 69967 | 6.1685 | 0.001 | B | O |
| E7EWP0 | NADH dehydrogenase [ubiquinone] 1 beta subcomplex subunit 5, mitochondrial OS=Homo sapi | 20025 | 7.8311 | 0.078 | B | O |
| E7EX29 | 14-3-3 protein zeta/delta (Fragment) OS=Homo sapiens GN=YWHAZ PE=1 SV=1 | 28018 | 4.6523 | 0.381 | B | O |
| E9PAV3 | Nascent polypeptide-associated complex subunit alpha, muscle-specific form OS=Homo sapiens | 205293 | 10.0386 | 0.262 | B | O |
| E9PB61 | THO complex subunit 4 OS=Homo sapiens GN=ALYREF PE=1 SV=1 | 27540 | 11.4551 | 0.001 | B | O |
| E9PCR7 | 2-oxoglutarate dehydrogenase, mitochondrial OS=Homo sapiens GN=OGDH PE=1 SV=1 | 117590 | 6.4482 | 0.001 | B | O |
| E9PCY7 | Heterogeneous nuclear ribonucleoprotein H OS=Homo sapiens GN=HNRNPH1 PE=1 SV=1 | 47057 | 5.896 | 0.505 | B | O |
| E9PD68 | Dihydropyrimidinase-related protein 1 OS=Homo sapiens GN=CRMP1 PE=1 SV=1 | 61990 | 6.2578 | 0.471 | B | O |
| E9PDE8 | Heat shock 70 kDa protein 4L OS=Homo sapiens GN=HSPA4L PE=1 SV=1 | 91895 | 5.6924 | 0.066 | B | O |
| E9PDG8 | Clathrin coat assembly protein AP180 OS=Homo sapiens GN=SNAP91 PE=1 SV=1 | 91913 | 4.522 | 0.233 | B | O |
| E9PDI2 | Adenylyl cyclase-associated protein OS=Homo sapiens GN=CAP2 PE=1 SV=1 | 49791 | 5.9106 | 0.019 | B | O |
| E9PDL2 | Dipeptidyl aminopeptidase-like protein 6 OS=Homo sapiens GN=DPP6 PE=1 SV=1 | 86272 | 5.8081 | 0.212 | B | O |
| E9PEJ4 | Acetyltransferase component of pyruvate dehydrogenase complex OS=Homo sapiens GN=DLA | 57550 | 9.2051 | 0.078 | B | O |
| E9PEW8 | Hemoglobin subunit delta (Fragment) OS=Homo sapiens GN=HBD PE=1 SV=1 | 11338 | 5.8945 | 0.001 | B | O |
| E9PEX6 | Dihydrolipoyl dehydrogenase OS=Homo sapiens GN=DLD PE=1 SV=1 | 51782 | 7.9233 | 0.248 | B | O |
| E9PF46 | Acylphosphatase OS=Homo sapiens GN=ACYP2 PE=1 SV=1 | 10291 | 9.0117 | 0.420 | B | O |
| E9PG15 | 14-3-3 protein theta (Fragment) OS=Homo sapiens GN=YWHAQ PE=1 SV=1 | 17037 | 5.5093 | 0.329 | B | O |
| E9PGC8 | Microtubule-associated protein 1A OS=Homo sapiens GN=MAP1A PE=1 SV=1 | 331054 | 4.6729 | 0.001 | B | O |
| E9PH64 | NADH dehydrogenase [ubiquinone] 1 beta subcomplex subunit 9 OS=Homo sapiens GN=NDUF | 20370 | 7.9688 | 0.090 | B | O |
| E9PHN6 | Glutathione S-transferase Mu 2 OS=Homo sapiens GN=GSTM2 PE=1 SV=1 | 22861 | 4.875 | 0.107 | B | O |
| E9PIA8 | Palmitoyl-protein thioesterase 1 (Fragment) OS=Homo sapiens GN=PPT1 PE=1 SV=7 | 22894 | 7.207 | 0.001 | B | O |
| E9PIM6 | Thy-1 membrane glycoprotein (Fragment) OS=Homo sapiens GN=THY1 PE=1 SV=6 | 16916 | 9.2461 | 0.874 | B | O |

| | | | | | | |
|---|---|---|---|---|---|---|
| E9PJH7 | Mitochondrial glutamate carrier 1 (Fragment) OS=Homo sapiens GN=SLC25A22 PE=1 SV=5 | 33260 | 10.0063 | 0.420 | B | O |
| E9PJL7 | Alpha-crystallin B chain (Fragment) OS=Homo sapiens GN=CRYAB PE=1 SV=6 | 15338 | 6.1362 | 0.001 | B | O |
| E9PK25 | Cofilin-1 OS=Homo sapiens GN=CFL1 PE=1 SV=1 | 22713 | 8.5181 | 0.001 | B | O |
| E9PKE3 | Heat shock cognate 71 kDa protein OS=Homo sapiens GN=HSPA8 PE=1 SV=1 | 68763 | 5.1973 | 1.199 | B | O |
| E9PKG1 | Protein arginine N-methyltransferase 1 OS=Homo sapiens GN=PRMT1 PE=1 SV=1 | 37684 | 5.71 | 0.038 | B | O |
| E9PKL7 | Ras-related protein Rab-2A OS=Homo sapiens GN=RAB2A PE=1 SV=1 | 20765 | 10.2158 | 0.396 | B | O |
| E9PKU7 | Neutral alpha-glucosidase AB OS=Homo sapiens GN=GANAB PE=1 SV=1 | 96483 | 5.5093 | 0.001 | B | O |
| E9PL57 | Protein NEDD8-MDP1 (Fragment) OS=Homo sapiens GN=NEDD8-MDP1 PE=4 SV=1 | 19524 | 7.4004 | 0.001 | B | O |
| E9PLK3 | Puromycin-sensitive aminopeptidase OS=Homo sapiens GN=NPEPPS PE=1 SV=1 | 102922 | 5.2661 | 0.122 | B | O |
| E9PMV1 | Plectin (Fragment) OS=Homo sapiens GN=PLEC PE=1 SV=1 | 80730 | 6.3018 | 2.347 | B | O |
| E9PNW4 | CD59 glycoprotein OS=Homo sapiens GN=CD59 PE=1 SV=1 | 11976 | 5.3672 | 0.253 | B | O |
| E9PP67 | Toll-interacting protein OS=Homo sapiens GN=TOLLIP PE=1 SV=1 | 20260 | 8.8608 | 0.101 | B | O |
| E9PPU1 | 40S ribosomal protein S3 OS=Homo sapiens GN=RPS3 PE=1 SV=1 | 17396 | 9.9609 | 0.184 | B | O |
| E9PQY2 | Prefoldin subunit 4 OS=Homo sapiens GN=PFDN4 PE=1 SV=1 | 15588 | 4.2803 | 0.001 | B | O |
| E9PR44 | Alpha-crystallin B chain (Fragment) OS=Homo sapiens GN=CRYAB PE=1 SV=1 | 20018 | 6.5698 | 0.709 | B | O |
| E9PRY8 | Elongation factor 1-delta OS=Homo sapiens GN=EEF1D PE=1 SV=1 | 76522 | 6.6196 | 0.157 | B | O |
| F2Z2Y4 | Pyridoxal kinase OS=Homo sapiens GN=PDXK PE=1 SV=1 | 30618 | 6.189 | 0.056 | B | O |
| F2Z393 | Transaldolase OS=Homo sapiens GN=TALDO1 PE=1 SV=1 | 35306 | 9.2988 | 0.065 | B | O |
| F2Z3J9 | Prostaglandin reductase 1 (Fragment) OS=Homo sapiens GN=PTGR1 PE=1 SV=2 | 13435 | 5.2515 | 0.022 | B | O |
| F5GWF6 | T-complex protein 1 subunit beta OS=Homo sapiens GN=CCT2 PE=1 SV=2 | 56770 | 5.9868 | 0.082 | B | O |
| F5GX19 | Ragulator complex protein LAMTOR1 OS=Homo sapiens GN=LAMTOR1 PE=1 SV=1 | 15677 | 4.7476 | 0.001 | B | O |
| F5GX30 | Cation-dependent mannose-6-phosphate receptor OS=Homo sapiens GN=M6PR PE=1 SV=2 | 21524 | 6.2358 | 0.143 | B | O |
| F5GYJ8 | Ubiquitin thioesterase OTUB1 OS=Homo sapiens GN=OTUB1 PE=1 SV=1 | 32338 | 4.6641 | 0.001 | B | O |
| F5GYQ1 | V-type proton ATPase subunit d 1 OS=Homo sapiens GN=ATP6V0D1 PE=1 SV=1 | 44631 | 4.8457 | 0.001 | B | O |
| F5GYX7 | Stromelysin-2 (Fragment) OS=Homo sapiens GN=MMP10 PE=1 SV=1 | 20468 | 8.4023 | 0.098 | B | O |
| F5GZS6 | 4F2 cell-surface antigen heavy chain OS=Homo sapiens GN=SLC3A2 PE=1 SV=1 | 64832 | 4.7813 | 0.183 | B | O |
| F5GZY7 | Gamma-aminobutyric acid receptor-associated protein-like 1 (Fragment) OS=Homo sapiens GN | 8571 | 10.3184 | 0.052 | B | O |
| F5H006 | Lymphoid-restricted membrane protein OS=Homo sapiens GN=LRMP PE=1 SV=1 | 50393 | 5.8594 | 0.001 | B | O |
| F5H018 | GTP-binding nuclear protein Ran (Fragment) OS=Homo sapiens GN=RAN PE=1 SV=6 | 22421 | 9.0278 | 0.194 | B | O |
| F5H0B0 | Uncharacterized protein OS=Homo sapiens PE=4 SV=2 | 27450 | 4.9629 | 0.001 | B | O |
| F5H157 | Ras-related protein Rab-35 (Fragment) OS=Homo sapiens GN=RAB35 PE=1 SV=1 | 21200 | 8.4126 | 0.120 | B | O |
| F5H1U9 | Multiple PDZ domain protein OS=Homo sapiens GN=MPDZ PE=1 SV=1 | 222986 | 4.7622 | 0.001 | B | O |
| F5H212 | Zinc finger protein 40 OS=Homo sapiens GN=HIVEP1 PE=1 SV=2 | 296487 | 7.5894 | 0.299 | B | O |
| F5H2V8 | Transcriptional repressor CTCFL OS=Homo sapiens GN=CTCFL PE=1 SV=1 | 51459 | 6.356 | 0.001 | B | O |
| F5H423 | Uncharacterized protein OS=Homo sapiens PE=3 SV=1 | 23331 | 9.0981 | 0.001 | B | O |
| F5H481 | Protein N-lysine methyltransferase METTL20 (Fragment) OS=Homo sapiens GN=METTL20 PE | 7875 | 7.0781 | 1.165 | B | O |
| F5H5G1 | Limbic system-associated membrane protein OS=Homo sapiens GN=LSAMP PE=1 SV=2 | 31722 | 5.5635 | 0.354 | B | O |
| F5H6T1 | ARP2 actin-related protein 2 homolog (Yeast), isoform CRA_d OS=Homo sapiens GN=ACTR2 I | 38817 | 5.8521 | 0.180 | B | O |
| F5H7S3 | Tropomyosin alpha-1 chain OS=Homo sapiens GN=TPM1 PE=1 SV=2 | 28507 | 4.5454 | 0.320 | B | O |
| F6RFD5 | Destrin OS=Homo sapiens GN=DSTN PE=1 SV=1 | 15386 | 8.748 | 0.606 | B | O |
| F6TLX2 | Glyoxalase domain-containing protein 4 OS=Homo sapiens GN=GLOD4 PE=1 SV=1 | 54684 | 8.7451 | 0.001 | B | O |
| F6U236 | Protein kinase C and casein kinase substrate in neurons protein 1 OS=Homo sapiens GN=PAC | 46117 | 5.0786 | 0.057 | B | O |
| F6X2W2 | Neuronal growth regulator 1 OS=Homo sapiens GN=NEGR1 PE=1 SV=1 | 32829 | 6.4731 | 0.303 | B | O |
| F8VQX6 | Methyltransferase-like protein 7A (Fragment) OS=Homo sapiens GN=METTL7A PE=1 SV=1 | 20648 | 8.0845 | 0.039 | B | O |
| F8VS02 | Alpha-aminoadipic semialdehyde dehydrogenase OS=Homo sapiens GN=ALDH7A1 PE=1 SV= | 51378 | 7.7271 | 1.480 | B | O |
| F8VSD4 | Ubiquitin-conjugating enzyme E2 N OS=Homo sapiens GN=UBE2N PE=1 SV=1 | 11975 | 9.5171 | 0.001 | B | O |
| F8VSL7 | Erythrocyte band 7 integral membrane protein OS=Homo sapiens GN=STOM PE=1 SV=1 | 18686 | 9.772 | 0.103 | B | O |
| F8VU65 | 60S acidic ribosomal protein P0 (Fragment) OS=Homo sapiens GN=RPLP0 PE=1 SV=1 | 27170 | 9.3574 | 0.001 | B | O |
| F8VVM2 | Phosphate carrier protein, mitochondrial OS=Homo sapiens GN=SLC25A3 PE=1 SV=1 | 36137 | 9.4966 | 0.486 | B | O |
| F8VWZ8 | Rho GTPase-activating protein 29 OS=Homo sapiens GN=ARHGAP29 PE=1 SV=1 | 117350 | 6.2036 | 0.017 | B | O |
| F8VYE8 | Serine/threonine-protein phosphatase OS=Homo sapiens GN=PPP1CC PE=1 SV=1 | 34927 | 4.9204 | 0.056 | B | O |
| F8VZ49 | Heterogeneous nuclear ribonucleoprotein A1 (Fragment) OS=Homo sapiens GN=HNRNPA1 PE | 25670 | 8.458 | 0.544 | B | O |
| F8VZX2 | Poly(rC)-binding protein 2 OS=Homo sapiens GN=PCBP2 PE=1 SV=1 | 33778 | 8.2822 | 0.001 | B | O |
| F8W031 | Uncharacterized protein (Fragment) OS=Homo sapiens PE=1 SV=1 | 29205 | 6.6079 | 0.030 | B | O |
| F8W726 | Ubiquitin-associated protein 2-like OS=Homo sapiens GN=UBAP2L PE=1 SV=2 | 113558 | 6.2051 | 0.091 | B | O |
| F8W8W4 | Prenylcysteine oxidase 1 OS=Homo sapiens GN=PCYOX1 PE=1 SV=1 | 32237 | 6.2446 | 0.114 | B | O |
| F8W9U4 | Microtubule-associated protein OS=Homo sapiens GN=MAP4 PE=1 SV=1 | 88222 | 9.6035 | 0.054 | B | O |
| F8WCA0 | Vesicle-associated membrane protein 2 OS=Homo sapiens GN=VAMP2 PE=4 SV=1 | 12925 | 8.7012 | 0.001 | B | O |
| F8WCF6 | Protein ARPC4-TTLL3 OS=Homo sapiens GN=ARPC4-TTLL3 PE=4 SV=1 | 21045 | 9.186 | 0.001 | B | O |
| F8WE04 | Heat shock protein beta-1 OS=Homo sapiens GN=HSPB1 PE=1 SV=1 | 20393 | 9.394 | 0.001 | B | O |
| G3V0I5 | NADH dehydrogenase (Ubiquinone) flavoprotein 1, 51kDa, isoform CRA_c OS=Homo sapiens ( | 50022 | 8.1533 | 0.123 | B | O |
| G3V153 | Caprin-1 OS=Homo sapiens GN=CAPRIN1 PE=1 SV=1 | 70310 | 4.7534 | 0.057 | B | O |
| G3V192 | Ferritin OS=Homo sapiens GN=FTH1 PE=1 SV=1 | 17642 | 5.1694 | 0.001 | B | O |
| G3V1B6 | MICOS complex subunit MIC26 OS=Homo sapiens GN=APOO PE=1 SV=1 | 13092 | 7.1616 | 0.138 | B | O |
| G3V1N2 | HCG1745306, isoform CRA_a OS=Homo sapiens GN=HBA2 PE=1 SV=1 | 11940 | 9.3018 | 1.770 | B | O |
| G3V2N0 | Guanine nucleotide-binding protein subunit gamma OS=Homo sapiens GN=GNG2 PE=1 SV=1 | 12378 | 9.3047 | 0.001 | B | O |
| G3V2S6 | V-type proton ATPase subunit D OS=Homo sapiens GN=ATP6V1D PE=1 SV=1 | 21907 | 5.48 | 0.107 | B | O |
| G3V3H7 | A-kinase anchor protein 6 OS=Homo sapiens GN=AKAP6 PE=1 SV=1 | 139552 | 5.0889 | 0.279 | B | O |
| G3V3M6 | DNA-(apurinic or apyrimidinic site) lyase (Fragment) OS=Homo sapiens GN=APEX1 PE=1 SV= | 29171 | 7.1265 | 0.001 | B | O |
| G3V4P8 | Glia maturation factor beta (Fragment) OS=Homo sapiens GN=GMFB PE=1 SV=1 | 17500 | 5.0332 | 0.001 | B | O |
| G3V5M7 | Pre-mRNA-processing factor 39 (Fragment) OS=Homo sapiens GN=PRPF39 PE=1 SV=1 | 16220 | 4.3096 | 0.381 | B | O |
| G3XAH0 | HCG2002594, isoform CRA_c OS=Homo sapiens GN=SEPT5 PE=1 SV=1 | 43817 | 6.2065 | 0.001 | B | O |
| G5EA42 | Tropomodulin 2 (Neuronal), isoform CRA_a OS=Homo sapiens GN=TMOD2 PE=1 SV=1 | 34471 | 6.4614 | 0.253 | B | O |
| G8JLB6 | Heterogeneous nuclear ribonucleoprotein H OS=Homo sapiens GN=HNRNPH1 PE=1 SV=1 | 51197 | 6.3442 | 0.001 | B | O |
| H0Y2P0 | CD44 antigen (Fragment) OS=Homo sapiens GN=CD44 PE=1 SV=1 | 30932 | 5.0156 | 0.001 | B | O |
| H0Y2P2 | Peptide-N(4)-(N-acetyl-beta-glucosaminyl)asparagine amidase (Fragment) OS=Homo sapiens ( | 26320 | 8.2661 | 0.095 | B | O |
| H0Y3Y4 | Septin-7 (Fragment) OS=Homo sapiens GN=SEPT7 PE=1 SV=1 | 43009 | 7.7446 | 0.381 | B | O |
| H0Y474 | V-type proton ATPase subunit G 2 (Fragment) OS=Homo sapiens GN=ATP6V1G2 PE=1 SV=1 | 16884 | 11.1489 | 0.001 | B | O |
| H0Y512 | Adipocyte plasma membrane-associated protein (Fragment) OS=Homo sapiens GN=APMAP PI | 45377 | 5.2998 | 0.243 | B | O |
| H0Y5C0 | Adhesion G protein-coupled receptor L2 (Fragment) OS=Homo sapiens GN=ADGRL2 PE=1 SV | 151152 | 6.1304 | 0.017 | B | O |
| H0Y614 | Ubiquitin-fold modifier 1 (Fragment) OS=Homo sapiens GN=UFM1 PE=1 SV=1 | 8656 | 9.8643 | 0.068 | B | O |

| | | | | | | |
|---|---|---|---|---|---|---|
| H0Y6E7 | RNA-binding motif protein, X chromosome (Fragment) OS=Homo sapiens GN=RBMX PE=1 SV | 31837 | 10.0635 | 0.278 | B | O |
| H0Y6I0 | Golgin subfamily A member 4 (Fragment) OS=Homo sapiens GN=GOLGA4 PE=1 SV=1 | 246454 | 5.1621 | 0.127 | B | O |
| H0Y875 | Calumenin (Fragment) OS=Homo sapiens GN=CALU PE=1 SV=1 | 17360 | 4.0796 | 0.052 | B | O |
| H0Y8G5 | Heterogeneous nuclear ribonucleoprotein D0 (Fragment) OS=Homo sapiens GN=HNRNPD PE= | 29648 | 9.4526 | 0.001 | B | O |
| H0Y8X1 | Succinate dehydrogenase [ubiquinone] flavoprotein subunit, mitochondrial (Fragment) OS=Hom | 13628 | 10.5 | 1.752 | B | O |
| H0YDD4 | Acetyltransferase component of pyruvate dehydrogenase complex (Fragment) OS=Homo sapie | 51169 | 8.6733 | 0.124 | B | O |
| H0YFS2 | 4F2 cell-surface antigen heavy chain (Fragment) OS=Homo sapiens GN=SLC3A2 PE=1 SV=1 | 26142 | 5.0098 | 0.054 | B | O |
| H0YH81 | ATP synthase subunit beta (Fragment) OS=Homo sapiens GN=ATP5B PE=1 SV=1 | 38226 | 5.2251 | 0.403 | B | O |
| H0YI98 | Dynactin subunit 2 (Fragment) OS=Homo sapiens GN=DCTN2 PE=1 SV=1 | 29308 | 6.3633 | 0.001 | B | O |
| H0YJ21 | Cytoplasmic dynein 1 heavy chain 1 (Fragment) OS=Homo sapiens GN=DYNC1H1 PE=1 SV=1 | 21009 | 7.0942 | 0.290 | B | O |
| H0YK48 | Tropomyosin alpha-1 chain OS=Homo sapiens GN=TPM1 PE=1 SV=1 | 28562 | 4.5396 | 0.443 | B | O |
| H0YL12 | Electron transfer flavoprotein subunit alpha, mitochondrial (Fragment) OS=Homo sapiens GN=E | 24893 | 6.0967 | 0.155 | B | O |
| H0YL18 | Beta-2-microglobulin OS=Homo sapiens GN=B2M PE=1 SV=1 | 13963 | 7.3081 | 0.001 | B | O |
| H0YL72 | Isocitrate dehydrogenase [NAD] subunit alpha, mitochondrial OS=Homo sapiens GN=IDH3A PE | 35763 | 5.9897 | 0.322 | B | O |
| H0YLA2 | Signal recognition particle 14 kDa protein OS=Homo sapiens GN=SRP14 PE=1 SV=1 | 13049 | 9.9697 | 0.079 | B | O |
| H0YNE9 | Ras-related protein Rab-8B (Fragment) OS=Homo sapiens GN=RAB8B PE=1 SV=1 | 21854 | 8.7349 | 0.079 | B | O |
| H3BLU2 | Limbic system-associated membrane protein (Fragment) OS=Homo sapiens GN=LSAMP PE=1 | 37990 | 6.4263 | 0.001 | B | O |
| H3BLU7 | Aflatoxin B1 aldehyde reductase member 2 (Fragment) OS=Homo sapiens GN=AKR7A2 PE=1 | 34662 | 6.7588 | 0.162 | B | O |
| H3BM89 | 60S ribosomal protein L4 OS=Homo sapiens GN=RPL4 PE=1 SV=1 | 37633 | 11.4712 | 0.079 | B | O |
| H3BMH2 | Ras-related protein Rab-11A (Fragment) OS=Homo sapiens GN=RAB11A PE=3 SV=1 | 17657 | 8.9253 | 0.217 | B | O |
| H3BMM9 | RNA-binding protein with serine-rich domain 1 (Fragment) OS=Homo sapiens GN=RNPS1 PE= | 31681 | 12.0308 | 0.001 | B | O |
| H3BNQ7 | 4-aminobutyrate aminotransferase, mitochondrial OS=Homo sapiens GN=ABAT PE=1 SV=1 | 57886 | 7.749 | 0.001 | B | O |
| H3BNX8 | Cytochrome c oxidase subunit 5A, mitochondrial OS=Homo sapiens GN=COX5A PE=1 SV=1 | 17223 | 5.603 | 0.001 | B | O |
| H3BPE7 | RNA-binding protein FUS OS=Homo sapiens GN=FUS PE=1 SV=1 | 53464 | 9.4951 | 0.001 | B | O |
| H3BPJ9 | NADH dehydrogenase [ubiquinone] 1 beta subcomplex subunit 10 OS=Homo sapiens GN=NDU | 19245 | 8.8608 | 0.001 | B | O |
| H3BPK3 | Hydroxyacylglutathione hydrolase, mitochondrial (Fragment) OS=Homo sapiens GN=HAGH PE | 26399 | 8.2705 | 0.725 | B | O |
| H3BPS8 | Fructose-bisphosphate aldolase (Fragment) OS=Homo sapiens GN=ALDOA PE=1 SV=1 | 30407 | 7.2949 | 0.691 | B | O |
| H3BRG4 | Cytochrome b-c1 complex subunit 2, mitochondrial OS=Homo sapiens GN=UQCRC2 PE=1 SV= | 44607 | 9.2827 | 0.001 | B | O |
| H3BT82 | Syntaxin-1B (Fragment) OS=Homo sapiens GN=STX1B PE=1 SV=2 | 23432 | 7.2378 | 0.491 | B | O |
| H3BTE6 | Erythrocyte membrane protein band 4.2 (Fragment) OS=Homo sapiens GN=EPB42 PE=1 SV=1 | 59796 | 7.4678 | 0.022 | B | O |
| H3BTN5 | Pyruvate kinase (Fragment) OS=Homo sapiens GN=PKM PE=1 SV=1 | 53011 | 6.3926 | 0.190 | B | O |
| H7BY36 | RNA-binding protein EWS (Fragment) OS=Homo sapiens GN=EWSR1 PE=1 SV=1 | 32161 | 10.1997 | 0.029 | B | O |
| H7BY57 | Neurofascin (Fragment) OS=Homo sapiens GN=NFASC PE=1 SV=1 | 116625 | 5.8857 | 0.172 | B | O |
| H7BYH4 | Superoxide dismutase [Cu-Zn] OS=Homo sapiens GN=SOD1 PE=1 SV=1 | 13900 | 5.6558 | 0.001 | B | O |
| H7BYR8 | Myelin basic protein OS=Homo sapiens GN=MBP PE=1 SV=1 | 25726 | 11.6763 | 1.188 | B | O |
| H7BZ94 | Protein disulfide-isomerase OS=Homo sapiens GN=P4HB PE=1 SV=2 | 52469 | 4.5703 | 0.121 | B | O |
| H7C0R7 | NADH-cytochrome b5 reductase 1 (Fragment) OS=Homo sapiens GN=CYB5R1 PE=1 SV=1 | 24889 | 9.2681 | 0.044 | B | O |
| H7C342 | D-dopachrome decarboxylase (Fragment) OS=Homo sapiens GN=DDT PE=1 SV=1 | 5697 | 9.7559 | 0.098 | B | O |
| H7C3P7 | Ras-related protein Ral-A (Fragment) OS=Homo sapiens GN=RALA PE=1 SV=1 | 18360 | 4.396 | 0.001 | B | O |
| H7C469 | Uncharacterized protein (Fragment) OS=Homo sapiens PE=1 SV=5 | 27654 | 6.1553 | 0.438 | B | O |
| H9KV31 | Neural cell adhesion molecule 2 OS=Homo sapiens GN=NCAM2 PE=1 SV=2 | 91066 | 5.2939 | 0.364 | B | O |
| I3L0A0 | HCG2044781 OS=Homo sapiens GN=TMEM189-UBE2V1 PE=4 SV=1 | 42181 | 6.249 | 0.152 | B | O |
| I3L0K2 | Thioredoxin domain-containing protein 17 OS=Homo sapiens GN=TXNDC17 PE=1 SV=1 | 8365 | 5.4185 | 0.111 | B | O |
| I3L0N3 | Vesicle-fusing ATPase OS=Homo sapiens GN=NSF PE=1 SV=1 | 82039 | 6.3735 | 0.461 | B | O |
| I3L1P8 | Mitochondrial 2-oxoglutarate/malate carrier protein (Fragment) OS=Homo sapiens GN=SLC25A | 32161 | 10.0503 | 0.246 | B | O |
| I3L397 | Eukaryotic translation initiation factor 5A (Fragment) OS=Homo sapiens GN=EIF5A PE=1 SV=6 | 16008 | 4.6567 | 0.088 | B | O |
| I3L3B0 | Complement component 1 Q subcomponent-binding protein, mitochondrial OS=Homo sapiens ( | 20121 | 3.8965 | 0.001 | B | O |
| J3KN75 | TBC1 domain family member 8B OS=Homo sapiens GN=TBC1D8B PE=1 SV=1 | 128026 | 5.6074 | 0.099 | B | O |
| J3KND3 | Myosin light polypeptide 6 OS=Homo sapiens GN=MYL6 PE=1 SV=1 | 17047 | 4.3608 | 0.001 | B | O |
| J3KPE3 | Guanine nucleotide-binding protein subunit beta-2-like 1 OS=Homo sapiens GN=GNB2L1 PE=1 | 30085 | 7.5469 | 0.090 | B | O |
| J3KPX7 | Prohibitin-2 OS=Homo sapiens GN=PHB2 PE=1 SV=2 | 33218 | 10.1895 | 0.462 | B | O |
| J3KQA0 | Synaptotagmin I, isoform CRA_b OS=Homo sapiens GN=SYT1 PE=1 SV=1 | 47230 | 7.9702 | 0.412 | B | O |
| J3KRI4 | Cytoplasmic dynein 1 light intermediate chain 2 (Fragment) OS=Homo sapiens GN=DYNC1LI2 | 17766 | 9.1348 | 0.137 | B | O |
| J3KSS7 | ADP-ribosylation factor-binding protein GGA3 OS=Homo sapiens GN=GGA3 PE=1 SV=1 | 67874 | 6.2168 | 1.844 | B | O |
| J3KTF8 | Rho GDP-dissociation inhibitor 1 (Fragment) OS=Homo sapiens GN=ARHGDIA PE=1 SV=6 | 21503 | 5.2104 | 1.261 | B | O |
| J3KTL2 | Serine/arginine-rich-splicing factor 1 OS=Homo sapiens GN=SRSF1 PE=1 SV=1 | 28311 | 10.2861 | 0.001 | B | O |
| J3QRS3 | Myosin regulatory light chain 12A OS=Homo sapiens GN=MYL12A PE=1 SV=1 | 20443 | 4.4048 | 0.001 | B | O |
| J3QRY4 | 26S proteasome non-ATPase regulatory subunit 11 (Fragment) OS=Homo sapiens GN=PSMD1 | 21095 | 5.1357 | 0.051 | B | O |
| J3QT27 | Poly(rC)-binding protein 3 (Fragment) OS=Homo sapiens GN=PCBP3 PE=1 SV=1 | 34363 | 7.7461 | 0.024 | B | O |
| J9JID7 | Lamin B2, isoform CRA_a OS=Homo sapiens GN=LMNB2 PE=1 SV=1 | 69905 | 5.3364 | 0.001 | B | O |
| K7EIR2 | MICOS complex subunit MIC13 OS=Homo sapiens GN=C19orf70 PE=1 SV=1 | 9694 | 9.1992 | 0.187 | B | O |
| K7EJN0 | Zinc finger protein 383 (Fragment) OS=Homo sapiens GN=ZNF383 PE=4 SV=1 | 13152 | 4.2524 | 0.249 | B | O |
| K7EK42 | Tubulin-folding cofactor B OS=Homo sapiens GN=TBCB PE=1 SV=1 | 21282 | 5.0376 | 0.049 | B | O |
| K7EK78 | Uncharacterized protein OS=Homo sapiens PE=4 SV=1 | 12762 | 8.4814 | 0.001 | B | O |
| K7EKH6 | Glial fibrillary acidic protein (Fragment) OS=Homo sapiens GN=GFAP PE=1 SV=1 | 22523 | 4.6362 | 0.568 | B | O |
| K7EKI8 | Periplakin OS=Homo sapiens GN=PPL PE=1 SV=1 | 204364 | 5.3364 | 0.042 | B | O |
| K7EKU3 | FXYD domain-containing ion transport regulator 7 OS=Homo sapiens GN=FXYD7 PE=1 SV=1 | 12638 | 8.0845 | 0.001 | B | O |
| K7ELL7 | Glucosidase 2 subunit beta OS=Homo sapiens GN=PRKCSH PE=1 SV=1 | 60154 | 4.1396 | 0.001 | B | O |
| K7EM49 | 6-phosphogluconate dehydrogenase, decarboxylating (Fragment) OS=Homo sapiens GN=PGD | 22243 | 5.9443 | 0.001 | B | O |
| K7EN45 | Peptidyl-prolyl cis-trans isomerase NIMA-interacting 1 (Fragment) OS=Homo sapiens GN=PIN1 | 9938 | 4.7373 | 0.133 | B | O |
| K7EQA1 | Programmed cell death protein 5 OS=Homo sapiens GN=PDCD5 PE=1 SV=1 | 9998 | 9.5171 | 0.094 | B | O |
| K7ER00 | Phenylalanine--tRNA ligase alpha subunit OS=Homo sapiens GN=FARSA PE=1 SV=1 | 62356 | 7.7534 | 0.001 | B | O |
| K7N7A8 | Uncharacterized protein (Fragment) OS=Homo sapiens PE=3 SV=2 | 48766 | 4.8501 | 0.001 | B | O |
| M0QXK4 | 40S ribosomal protein S19 (Fragment) OS=Homo sapiens GN=RPS19 PE=1 SV=1 | 8474 | 10.1528 | 0.001 | B | O |
| M0QXS5 | Heterogeneous nuclear ribonucleoprotein L (Fragment) OS=Homo sapiens GN=HNRNPL PE=1 | 58436 | 6.3208 | 0.001 | B | O |
| M0QY67 | Electron transfer flavoprotein subunit beta (Fragment) OS=Homo sapiens GN=ETFB PE=1 SV= | 19510 | 7.8267 | 0.213 | B | O |
| M0QZL1 | Flavin reductase (NADPH) OS=Homo sapiens GN=BLVRB PE=1 SV=1 | 15659 | 5.0771 | 0.098 | B | O |
| M0QZN5 | Alpha-soluble NSF attachment protein OS=Homo sapiens GN=NAPA PE=1 SV=1 | 6515 | 4.6743 | 0.014 | B | O |
| M0R0I3 | Endophilin-A2 (Fragment) OS=Homo sapiens GN=SH3GL1 PE=1 SV=1 | 15742 | 9.1318 | 0.018 | B | O |
| M0R0Y2 | Alpha-soluble NSF attachment protein OS=Homo sapiens GN=NAPA PE=1 SV=1 | 29144 | 4.8955 | 0.068 | B | O |
| M0R116 | Sodium/potassium-transporting ATPase subunit alpha OS=Homo sapiens GN=ATP1A3 PE=1 S | 108209 | 4.9629 | 0.422 | B | O |

| | | | | | | |
|---|---|---|---|---|---|---|
| M0R1T5 | Charged multivesicular body protein 2a (Fragment) OS=Homo sapiens GN=CHMP2A PE=1 SV= | 24876 | 4.9644 | 0.001 | B | O |
| M0R3D6 | 60S ribosomal protein L18a (Fragment) OS=Homo sapiens GN=RPL18A PE=1 SV=1 | 16703 | 11.1621 | 0.019 | B | O |
| O00148 | ATP-dependent RNA helicase DDX39A OS=Homo sapiens GN=DDX39A PE=1 SV=2 | 49098 | 5.3291 | 0.244 | B | O |
| O00154 | Cytosolic acyl coenzyme A thioester hydrolase OS=Homo sapiens GN=ACOT7 PE=1 SV=3 | 41769 | 8.5444 | 0.234 | B | O |
| O00264 | Membrane-associated progesterone receptor component 1 OS=Homo sapiens GN=PGRMC1 P | 21657 | 4.3433 | 0.273 | B | O |
| O00330 | Pyruvate dehydrogenase protein X component, mitochondrial OS=Homo sapiens GN=PDHX PE | 54088 | 9.0073 | 0.027 | B | O |
| O00429 | Dynamin-1-like protein OS=Homo sapiens GN=DNM1L PE=1 SV=2 | 81826 | 6.3633 | 0.125 | B | O |
| O00499 | Myc box-dependent-interacting protein 1 OS=Homo sapiens GN=BIN1 PE=1 SV=1 | 64659 | 4.7886 | 0.033 | B | O |
| O14594 | Neurocan core protein OS=Homo sapiens GN=NCAN PE=1 SV=3 | 143003 | 5.0698 | 0.198 | B | O |
| O14745 | Na(+)/H(+) exchange regulatory cofactor NHE-RF1 OS=Homo sapiens GN=SLC9A3R1 PE=1 S | 38844 | 5.4331 | 0.251 | B | O |
| O14807 | Ras-related protein M-Ras OS=Homo sapiens GN=MRAS PE=1 SV=2 | 23831 | 9.2227 | 0.043 | B | O |
| O14818 | Proteasome subunit alpha type-7 OS=Homo sapiens GN=PSMA7 PE=1 SV=1 | 27869 | 8.7114 | 0.138 | B | O |
| O14880 | Microsomal glutathione S-transferase 3 OS=Homo sapiens GN=MGST3 PE=1 SV=1 | 16505 | 9.6021 | 0.119 | B | O |
| O15020 | Spectrin beta chain, non-erythrocytic 2 OS=Homo sapiens GN=SPTBN2 PE=1 SV=3 | 271155 | 5.7144 | 0.221 | B | O |
| O15144 | Actin-related protein 2/3 complex subunit 2 OS=Homo sapiens GN=ARPC2 PE=1 SV=1 | 34311 | 6.9785 | 1.695 | B | O |
| O15145 | Actin-related protein 2/3 complex subunit 3 OS=Homo sapiens GN=ARPC3 PE=1 SV=3 | 20533 | 8.8037 | 0.140 | B | O |
| O15212 | Prefoldin subunit 6 OS=Homo sapiens GN=PFDN6 PE=1 SV=1 | 14573 | 9.4263 | 0.245 | B | O |
| O15240 | Neurosecretory protein VGF OS=Homo sapiens GN=VGF PE=1 SV=2 | 67217 | 4.5645 | 0.166 | B | O |
| O15400 | Syntaxin-7 OS=Homo sapiens GN=STX7 PE=1 SV=4 | 29797 | 5.2559 | 0.062 | B | O |
| O15498 | Synaptobrevin homolog YKT6 OS=Homo sapiens GN=YKT6 PE=1 SV=1 | 22403 | 6.5098 | 0.066 | B | O |
| O43175 | D-3-phosphoglycerate dehydrogenase OS=Homo sapiens GN=PHGDH PE=1 SV=4 | 56614 | 6.2798 | 0.059 | B | O |
| O43181 | NADH dehydrogenase [ubiquinone] iron-sulfur protein 4, mitochondrial OS=Homo sapiens GN=I | 20095 | 10.7593 | 0.450 | B | O |
| O43236 | Septin-4 OS=Homo sapiens GN=SEPT4 PE=1 SV=1 | 55063 | 5.6938 | 0.012 | B | O |
| O43242 | 26S proteasome non-ATPase regulatory subunit 3 OS=Homo sapiens GN=PSMD3 PE=1 SV=2 | 60939 | 8.6953 | 0.047 | B | O |
| O43301 | Heat shock 70 kDa protein 12A OS=Homo sapiens GN=HSPA12A PE=1 SV=2 | 74931 | 6.3179 | 0.174 | B | O |
| O43390 | Heterogeneous nuclear ribonucleoprotein R OS=Homo sapiens GN=HNRNPR PE=1 SV=1 | 70899 | 8.2119 | 0.073 | B | O |
| O43396 | Thioredoxin-like protein 1 OS=Homo sapiens GN=TXNL1 PE=1 SV=3 | 32230 | 4.6436 | 0.050 | B | O |
| O43490 | Prominin-1 OS=Homo sapiens GN=PROM1 PE=1 SV=1 | 97140 | 6.9346 | 1.055 | B | O |
| O43491 | Band 4.1-like protein 2 OS=Homo sapiens GN=EPB41L2 PE=1 SV=1 | 112519 | 5.1753 | 0.050 | B | O |
| O43707 | Alpha-actinin-4 OS=Homo sapiens GN=ACTN4 PE=1 SV=2 | 104788 | 5.1211 | 0.053 | B | O |
| O43852 | Calumenin OS=Homo sapiens GN=CALU PE=1 SV=2 | 37083 | 4.2773 | 0.131 | B | O |
| O43865 | Adenosylhomocysteinase 2 OS=Homo sapiens GN=AHCYL1 PE=1 SV=2 | 58913 | 6.4775 | 0.084 | B | O |
| O43920 | NADH dehydrogenase [ubiquinone] iron-sulfur protein 5 OS=Homo sapiens GN=NDUFS5 PE=1 | 12509 | 9.4775 | 0.203 | B | O |
| O60268 | Uncharacterized protein KIAA0513 OS=Homo sapiens GN=KIAA0513 PE=2 SV=1 | 46609 | 4.7856 | 0.076 | B | O |
| O60308 | Centrosomal protein of 104 kDa OS=Homo sapiens GN=CEP104 PE=1 SV=1 | 104381 | 7.3857 | 0.061 | B | O |
| O60506 | Heterogeneous nuclear ribonucleoprotein Q OS=Homo sapiens GN=SYNCRIP PE=1 SV=2 | 69559 | 8.7861 | 0.072 | B | O |
| O75037 | Kinesin-like protein KIF21B OS=Homo sapiens GN=KIF21B PE=1 SV=2 | 182546 | 6.665 | 1.008 | B | O |
| O75083 | WD repeat-containing protein 1 OS=Homo sapiens GN=WDR1 PE=1 SV=4 | 66151 | 6.1772 | 0.164 | B | O |
| O75131 | Copine-3 OS=Homo sapiens GN=CPNE3 PE=1 SV=1 | 60092 | 5.4727 | 0.122 | B | O |
| O75145 | Liprin-alpha-3 OS=Homo sapiens GN=PPFIA3 PE=1 SV=3 | 133413 | 5.3877 | 11.475 | B | O |
| O75223 | Gamma-glutamylcyclotransferase OS=Homo sapiens GN=GGCT PE=1 SV=1 | 20994 | 4.8779 | 0.055 | B | O |
| O75306 | NADH dehydrogenase [ubiquinone] iron-sulfur protein 2, mitochondrial OS=Homo sapiens GN=I | 52511 | 7.3008 | 0.057 | B | O |
| O75323 | Protein NipSnap homolog 2 OS=Homo sapiens GN=GBAS PE=1 SV=1 | 33721 | 9.6211 | 0.043 | B | O |
| O75368 | SH3 domain-binding glutamic acid-rich-like protein OS=Homo sapiens GN=SH3BGRL PE=1 SV | 12766 | 5.0215 | 0.318 | B | O |
| O75380 | NADH dehydrogenase [ubiquinone] iron-sulfur protein 6, mitochondrial OS=Homo sapiens GN=I | 13702 | 8.2939 | 0.051 | B | O |
| O75436 | Vacuolar protein sorting-associated protein 26A OS=Homo sapiens GN=VPS26A PE=1 SV=2 | 38145 | 6.1172 | 0.031 | B | O |
| O75489 | NADH dehydrogenase [ubiquinone] iron-sulfur protein 3, mitochondrial OS=Homo sapiens GN=I | 30222 | 7.3623 | 0.306 | B | O |
| O75531 | Barrier-to-autointegration factor OS=Homo sapiens GN=BANF1 PE=1 SV=1 | 10052 | 5.7231 | 0.062 | B | O |
| O75569 | Interferon-inducible double-stranded RNA-dependent protein kinase activator A OS=Homo sapie | 34382 | 8.4536 | 0.110 | B | O |
| O75648 | Mitochondrial tRNA-specific 2-thiouridylase 1 OS=Homo sapiens GN=TRMU PE=1 SV=2 | 47714 | 7.957 | 0.213 | B | O |
| O75746 | Calcium-binding mitochondrial carrier protein Aralar1 OS=Homo sapiens GN=SLC25A12 PE=1 | 74714 | 8.4551 | 0.115 | B | O |
| O75781 | Paralemmin-1 OS=Homo sapiens GN=PALM PE=1 SV=2 | 42050 | 4.7432 | 0.382 | B | O |
| O75787 | Renin receptor OS=Homo sapiens GN=ATP6AP2 PE=1 SV=2 | 38983 | 5.6821 | 0.028 | B | O |
| O75821 | Eukaryotic translation initiation factor 3 subunit G OS=Homo sapiens GN=EIF3G PE=1 SV=2 | 35588 | 5.7642 | 0.138 | B | O |
| O75828 | Carbonyl reductase [NADPH] 3 OS=Homo sapiens GN=CBR3 PE=1 SV=3 | 30830 | 5.7554 | 0.052 | B | O |
| O75947 | ATP synthase subunit d, mitochondrial OS=Homo sapiens GN=ATP5H PE=1 SV=3 | 18479 | 5.0244 | 0.682 | B | O |
| O75964 | ATP synthase subunit g, mitochondrial OS=Homo sapiens GN=ATP5L PE=1 SV=3 | 11421 | 9.9844 | 0.839 | B | O |
| O76041 | Nebulette OS=Homo sapiens GN=NEBL PE=1 SV=1 | 116380 | 8.0435 | 0.186 | B | O |
| O76070 | Gamma-synuclein OS=Homo sapiens GN=SNCG PE=1 SV=2 | 13322 | 4.6626 | 0.231 | B | O |
| O94760 | N(G),N(G)-dimethylarginine dimethylaminohydrolase 1 OS=Homo sapiens GN=DDAH1 PE=1 S | 31101 | 5.4287 | 0.574 | B | O |
| O94811 | Tubulin polymerization-promoting protein OS=Homo sapiens GN=TPPP PE=1 SV=1 | 23679 | 9.8892 | 0.522 | B | O |
| O94856 | Neurofascin OS=Homo sapiens GN=NFASC PE=1 SV=4 | 149933 | 6.208 | 0.001 | B | O |
| O94915 | Protein furry homolog-like OS=Homo sapiens GN=FRYL PE=1 SV=2 | 339381 | 5.2354 | 0.016 | B | O |
| O94925 | Glutaminase kidney isoform, mitochondrial OS=Homo sapiens GN=GLS PE=1 SV=1 | 73413 | 7.6011 | 0.176 | B | O |
| O95168 | NADH dehydrogenase [ubiquinone] 1 beta subcomplex subunit 4 OS=Homo sapiens GN=NDUF | 15199 | 10.0942 | 0.107 | B | O |
| O95169 | NADH dehydrogenase [ubiquinone] 1 beta subcomplex subunit 8, mitochondrial OS=Homo sapi | 21751 | 6.334 | 0.092 | B | O |
| O95202 | LETM1 and EF-hand domain-containing protein 1, mitochondrial OS=Homo sapiens GN=LETM | 83301 | 6.2695 | 1.354 | B | O |
| O95292 | Vesicle-associated membrane protein-associated protein B/C OS=Homo sapiens GN=VAPB PE | 27211 | 7.1206 | 0.028 | B | O |
| O95298 | NADH dehydrogenase [ubiquinone] 1 subunit C2 OS=Homo sapiens GN=NDUFC2 PE=1 SV=1 | 14178 | 9.2886 | 0.036 | B | O |
| O95716 | Ras-related protein Rab-3D OS=Homo sapiens GN=RAB3D PE=1 SV=1 | 24251 | 4.5674 | 0.317 | B | O |
| O95741 | Copine-6 OS=Homo sapiens GN=CPNE6 PE=1 SV=3 | 61951 | 5.168 | 0.195 | B | O |
| O95782 | AP-2 complex subunit alpha-1 OS=Homo sapiens GN=AP2A1 PE=1 SV=3 | 107477 | 6.6255 | 0.054 | B | O |
| O95817 | BAG family molecular chaperone regulator 3 OS=Homo sapiens GN=BAG3 PE=1 SV=3 | 61556 | 6.4775 | 0.075 | B | O |
| O95865 | N(G),N(G)-dimethylarginine dimethylaminohydrolase 2 OS=Homo sapiens GN=DDAH2 PE=1 S | 29625 | 5.5898 | 0.081 | B | O |
| O95989 | Diphosphoinositol polyphosphate phosphohydrolase 1 OS=Homo sapiens GN=NUDT3 PE=1 S\ | 19458 | 5.9487 | 0.145 | B | O |
| P00338 | L-lactate dehydrogenase A chain OS=Homo sapiens GN=LDHA PE=1 SV=2 | 36665 | 8.3657 | 0.225 | B | O |
| P00352 | Retinal dehydrogenase 1 OS=Homo sapiens GN=ALDH1A1 PE=1 SV=2 | 54826 | 6.2886 | 0.150 | B | O |
| P00367 | Glutamate dehydrogenase 1, mitochondrial OS=Homo sapiens GN=GLUD1 PE=1 SV=2 | 61359 | 7.7021 | 0.652 | B | O |
| P00441 | Superoxide dismutase [Cu-Zn] OS=Homo sapiens GN=SOD1 PE=1 SV=2 | 15925 | 5.666 | 1.528 | B | O |
| P00492 | Hypoxanthine-guanine phosphoribosyltransferase OS=Homo sapiens GN=HPRT1 PE=1 SV=2 | 24563 | 6.2314 | 0.095 | B | O |
| P00505 | Aspartate aminotransferase, mitochondrial OS=Homo sapiens GN=GOT2 PE=1 SV=3 | 47487 | 9.2637 | 0.815 | B | O |

| | | | | | | |
|---|---|---|---|---|---|---|
| P00558 | Phosphoglycerate kinase 1 OS=Homo sapiens GN=PGK1 PE=1 SV=3 | 44586 | 8.1475 | 1.000 | B | O |
| P00568 | Adenylate kinase isoenzyme 1 OS=Homo sapiens GN=AK1 PE=1 SV=3 | 21621 | 8.9897 | 0.401 | B | O |
| P00918 | Carbonic anhydrase 2 OS=Homo sapiens GN=CA2 PE=1 SV=2 | 29227 | 7.0269 | 0.275 | B | O |
| P00966 | Argininosuccinate synthase OS=Homo sapiens GN=ASS1 PE=1 SV=2 | 46501 | 8.0566 | 0.033 | B | O |
| P01034 | Cystatin-C OS=Homo sapiens GN=CST3 PE=1 SV=1 | 15789 | 8.9854 | 0.061 | B | O |
| P01111 | GTPase NRas OS=Homo sapiens GN=NRAS PE=1 SV=1 | 21215 | 4.8208 | 0.001 | B | O |
| P01112 | GTPase HRas OS=Homo sapiens GN=HRAS PE=1 SV=1 | 21284 | 4.9805 | 0.054 | B | O |
| P01213 | Proenkephalin-B OS=Homo sapiens GN=PDYN PE=1 SV=1 | 28367 | 5.9839 | 0.102 | B | O |
| P01859 | Ig gamma-2 chain C region OS=Homo sapiens GN=IGHG2 PE=1 SV=2 | 35877 | 7.437 | 0.107 | B | O |
| P01860 | Ig gamma-3 chain C region OS=Homo sapiens GN=IGHG3 PE=1 SV=2 | 41260 | 7.7886 | 0.007 | B | O |
| P01876 | Ig alpha-1 chain C region OS=Homo sapiens GN=IGHA1 PE=1 SV=2 | 37630 | 6.0571 | 0.145 | B | O |
| P02008 | Hemoglobin subunit zeta OS=Homo sapiens GN=HBZ PE=1 SV=2 | 15627 | 8.6514 | 0.011 | B | O |
| P02545 | Prelamin-A/C OS=Homo sapiens GN=LMNA PE=1 SV=1 | 74094 | 6.5815 | 0.242 | B | O |
| P02647 | Apolipoprotein A-I OS=Homo sapiens GN=APOA1 PE=1 SV=1 | 30758 | 5.4316 | 0.140 | B | O |
| P02649 | Apolipoprotein E OS=Homo sapiens GN=APOE PE=1 SV=1 | 36131 | 5.4829 | 0.094 | B | O |
| P02686 | Myelin basic protein OS=Homo sapiens GN=MBP PE=1 SV=3 | 33097 | 10.2217 | 1.243 | B | O |
| P02768 | Serum albumin OS=Homo sapiens GN=ALB PE=1 SV=2 | 69321 | 5.8608 | 2.845 | B | O |
| P02787 | Serotransferrin OS=Homo sapiens GN=TF PE=1 SV=3 | 77013 | 6.7515 | 0.022 | B | O |
| P02792 | Ferritin light chain OS=Homo sapiens GN=FTL PE=1 SV=2 | 20007 | 5.4038 | 0.622 | B | O |
| P02794 | Ferritin heavy chain OS=Homo sapiens GN=FTH1 PE=1 SV=2 | 21212 | 5.187 | 0.680 | B | O |
| P04075 | Fructose-bisphosphate aldolase A OS=Homo sapiens GN=ALDOA PE=1 SV=2 | 39395 | 8.0654 | 1.644 | B | O |
| P04080 | Cystatin-B OS=Homo sapiens GN=CSTB PE=1 SV=2 | 11132 | 7.5073 | 0.354 | B | O |
| P04083 | Annexin A1 OS=Homo sapiens GN=ANXA1 PE=1 SV=2 | 38689 | 6.6372 | 0.069 | B | O |
| P04264 | Keratin, type II cytoskeletal 1 OS=Homo sapiens GN=KRT1 PE=1 SV=6 | 65998 | 8.2661 | 1.280 | B | O |
| P04271 | Protein S100-B OS=Homo sapiens GN=S100B PE=1 SV=2 | 10706 | 4.3257 | 0.377 | B | O |
| P04350 | Tubulin beta-4A chain OS=Homo sapiens GN=TUBB4A PE=1 SV=2 | 49553 | 4.5908 | 1.728 | B | O |
| P04406 | Glyceraldehyde-3-phosphate dehydrogenase OS=Homo sapiens GN=GAPDH PE=1 SV=3 | 36030 | 8.6968 | 5.529 | B | O |
| P04792 | Heat shock protein beta-1 OS=Homo sapiens GN=HSPB1 PE=1 SV=2 | 22768 | 5.959 | 0.940 | B | O |
| P04843 | Dolichyl-diphosphooligosaccharide--protein glycosyltransferase subunit 1 OS=Homo sapiens GN | 68526 | 5.9268 | 0.040 | B | O |
| P04844 | Dolichyl-diphosphooligosaccharide--protein glycosyltransferase subunit 2 OS=Homo sapiens GN | 69241 | 5.332 | 0.069 | B | O |
| P04899 | Guanine nucleotide-binding protein G(i) subunit alpha-2 OS=Homo sapiens GN=GNAI2 PE=1 S | 40425 | 5.1899 | 0.200 | B | O |
| P04908 | Histone H2A type 1-B/E OS=Homo sapiens GN=HIST1H2AB PE=1 SV=2 | 14126 | 11.4712 | 0.001 | B | O |
| P05023 | Sodium/potassium-transporting ATPase subunit alpha-1 OS=Homo sapiens GN=ATP1A1 PE=1 | 112824 | 5.1694 | 6.108 | B | O |
| P05026 | Sodium/potassium-transporting ATPase subunit beta-1 OS=Homo sapiens GN=ATP1B1 PE=1 S | 35038 | 8.6484 | 1.529 | B | O |
| P05060 | Secretogranin-1 OS=Homo sapiens GN=CHGB PE=1 SV=2 | 78229 | 4.8354 | 0.178 | B | O |
| P05062 | Fructose-bisphosphate aldolase B OS=Homo sapiens GN=ALDOB PE=1 SV=2 | 39448 | 7.793 | 0.006 | B | O |
| P05091 | Aldehyde dehydrogenase, mitochondrial OS=Homo sapiens GN=ALDH2 PE=1 SV=2 | 56345 | 6.6694 | 0.515 | B | O |
| P05141 | ADP/ATP translocase 2 OS=Homo sapiens GN=SLC25A5 PE=1 SV=7 | 32831 | 9.9917 | 0.438 | B | O |
| P05165 | Propionyl-CoA carboxylase alpha chain, mitochondrial OS=Homo sapiens GN=PCCA PE=1 SV= | 80008 | 7.2524 | 0.055 | B | O |
| P05386 | 60S acidic ribosomal protein P1 OS=Homo sapiens GN=RPLP1 PE=1 SV=1 | 11506 | 4.0005 | 0.123 | B | O |
| P05413 | Fatty acid-binding protein, heart OS=Homo sapiens GN=FABP3 PE=1 SV=4 | 14848 | 6.3574 | 0.001 | B | O |
| P05455 | Lupus La protein OS=Homo sapiens GN=SSB PE=1 SV=2 | 46808 | 6.7529 | 0.039 | B | O |
| P05556 | Integrin beta-1 OS=Homo sapiens GN=ITGB1 PE=1 SV=2 | 88356 | 5.0977 | 0.078 | B | O |
| P05787 | Keratin, type II cytoskeletal 8 OS=Homo sapiens GN=KRT8 PE=1 SV=7 | 53671 | 5.3408 | 0.016 | B | O |
| P06396 | Gelsolin OS=Homo sapiens GN=GSN PE=1 SV=1 | 85644 | 5.8418 | 0.198 | B | O |
| P06576 | ATP synthase subunit beta, mitochondrial OS=Homo sapiens GN=ATP5B PE=1 SV=3 | 56524 | 5.0962 | 3.995 | B | O |
| P06733 | Alpha-enolase OS=Homo sapiens GN=ENO1 PE=1 SV=2 | 47139 | 7.1719 | 3.443 | B | O |
| P06748 | Nucleophosmin OS=Homo sapiens GN=NPM1 PE=1 SV=2 | 32554 | 4.4443 | 0.285 | B | O |
| P06753 | Tropomyosin alpha-3 chain OS=Homo sapiens GN=TPM3 PE=1 SV=2 | 32929 | 4.4868 | 0.049 | B | O |
| P06899 | Histone H2B type 1-J OS=Homo sapiens GN=HIST1H2BJ PE=1 SV=3 | 13895 | 10.7402 | 0.001 | B | O |
| P07195 | L-lactate dehydrogenase B chain OS=Homo sapiens GN=LDHB PE=1 SV=2 | 36615 | 5.6396 | 1.051 | B | O |
| P07196 | Neurofilament light polypeptide OS=Homo sapiens GN=NEFL PE=1 SV=3 | 61479 | 4.4326 | 0.582 | B | O |
| P07339 | Cathepsin D OS=Homo sapiens GN=CTSD PE=1 SV=1 | 44523 | 6.0908 | 1.056 | B | O |
| P07437 | Tubulin beta chain OS=Homo sapiens GN=TUBB PE=1 SV=2 | 49638 | 4.5908 | 1.768 | B | O |
| P07737 | Profilin-1 OS=Homo sapiens GN=PFN1 PE=1 SV=2 | 15044 | 8.4595 | 0.571 | B | O |
| P07858 | Cathepsin B OS=Homo sapiens GN=CTSB PE=1 SV=3 | 37796 | 5.855 | 0.132 | B | O |
| P07900 | Heat shock protein HSP 90-alpha OS=Homo sapiens GN=HSP90AA1 PE=1 SV=5 | 84606 | 4.7476 | 0.996 | B | O |
| P07919 | Cytochrome b-c1 complex subunit 6, mitochondrial OS=Homo sapiens GN=UQCRH PE=1 SV=2 | 10731 | 4.1865 | 0.240 | B | O |
| P07954 | Fumarate hydratase, mitochondrial OS=Homo sapiens GN=FH PE=1 SV=3 | 54602 | 9.0835 | 1.215 | B | O |
| P08133 | Annexin A6 OS=Homo sapiens GN=ANXA6 PE=1 SV=3 | 75825 | 5.2646 | 1.464 | B | O |
| P08195 | 4F2 cell-surface antigen heavy chain OS=Homo sapiens GN=SLC3A2 PE=1 SV=3 | 67951 | 4.6992 | 0.095 | B | O |
| P08237 | ATP-dependent 6-phosphofructokinase, muscle type OS=Homo sapiens GN=PFKM PE=1 SV=2 | 85128 | 7.8926 | 0.058 | B | O |
| P08238 | Heat shock protein HSP 90-beta OS=Homo sapiens GN=HSP90AB1 PE=1 SV=4 | 83212 | 4.7739 | 0.383 | B | O |
| P08247 | Synaptophysin OS=Homo sapiens GN=SYP PE=1 SV=3 | 33823 | 4.4561 | 0.284 | B | O |
| P08559 | Pyruvate dehydrogenase E1 component subunit alpha, somatic form, mitochondrial OS=Homo s | 43267 | 7.9995 | 0.197 | B | O |
| P08572 | Collagen alpha-2(IV) chain OS=Homo sapiens GN=COL4A2 PE=1 SV=4 | 167448 | 8.7378 | 0.071 | B | O |
| P08574 | Cytochrome c1, heme protein, mitochondrial OS=Homo sapiens GN=CYC1 PE=1 SV=3 | 35398 | 9.1831 | 0.170 | B | O |
| P08670 | Vimentin OS=Homo sapiens GN=VIM PE=1 SV=4 | 53619 | 4.8633 | 0.753 | B | O |
| P08727 | Keratin, type I cytoskeletal 19 OS=Homo sapiens GN=KRT19 PE=1 SV=4 | 44079 | 4.8604 | 3.635 | B | O |
| P08754 | Guanine nucleotide-binding protein G(k) subunit alpha OS=Homo sapiens GN=GNAI3 PE=1 SV | 40506 | 5.3584 | 0.202 | B | O |
| P08758 | Annexin A5 OS=Homo sapiens GN=ANXA5 PE=1 SV=2 | 35914 | 4.7329 | 0.498 | B | O |
| P09104 | Gamma-enolase OS=Homo sapiens GN=ENO2 PE=1 SV=3 | 47239 | 4.7183 | 0.880 | B | O |
| P09211 | Glutathione S-transferase P OS=Homo sapiens GN=GSTP1 PE=1 SV=2 | 23341 | 5.2822 | 0.503 | B | O |
| P09382 | Galectin-1 OS=Homo sapiens GN=LGALS1 PE=1 SV=2 | 14706 | 5.1416 | 0.470 | B | O |
| P09417 | Dihydropteridine reductase OS=Homo sapiens GN=QDPR PE=1 SV=2 | 25773 | 7.1719 | 0.126 | B | O |
| P09471 | Guanine nucleotide-binding protein G(o) subunit alpha OS=Homo sapiens GN=GNAO1 PE=1 S | 40024 | 5.1899 | 0.543 | B | O |
| P09497 | Clathrin light chain B OS=Homo sapiens GN=CLTB PE=1 SV=1 | 25175 | 4.3726 | 0.274 | B | O |
| P09543 | 2',3'-cyclic-nucleotide 3'-phosphodiesterase OS=Homo sapiens GN=CNP PE=1 SV=2 | 47548 | 9.3618 | 0.358 | B | O |
| P09669 | Cytochrome c oxidase subunit 6C OS=Homo sapiens GN=COX6C PE=1 SV=2 | 8775 | 10.752 | 0.196 | B | O |
| P09936 | Ubiquitin carboxyl-terminal hydrolase isozyme L1 OS=Homo sapiens GN=UCHL1 PE=1 SV=2 | 24808 | 5.1841 | 0.775 | B | O |
| P09972 | Fructose-bisphosphate aldolase C OS=Homo sapiens GN=ALDOC PE=1 SV=2 | 39431 | 6.4351 | 0.612 | B | O |

| | | | | | | |
|---|---|---|---|---|---|---|
| P0CG38 | POTE ankyrin domain family member I OS=Homo sapiens GN=POTEI PE=3 SV=1 | 121204 | 5.7817 | 2.459 | B | O |
| P0DME0 | Protein SETSIP OS=Homo sapiens GN=SETSIP PE=1 SV=1 | 34861 | 3.9917 | 0.413 | B | O |
| P0DMV8 | Heat shock 70 kDa protein 1A OS=Homo sapiens GN=HSPA1A PE=1 SV=1 | 70009 | 5.3188 | 0.613 | B | O |
| P10114 | Ras-related protein Rap-2a OS=Homo sapiens GN=RAP2A PE=1 SV=1 | 20602 | 4.5278 | 0.142 | B | O |
| P10155 | 60 kDa SS-A/Ro ribonucleoprotein OS=Homo sapiens GN=TROVE2 PE=1 SV=2 | 60631 | 7.9614 | 0.023 | B | O |
| P10253 | Lysosomal alpha-glucosidase OS=Homo sapiens GN=GAA PE=1 SV=4 | 105256 | 5.5649 | 0.055 | B | O |
| P10599 | Thioredoxin OS=Homo sapiens GN=TXN PE=1 SV=3 | 11729 | 4.6201 | 0.554 | B | O |
| P10606 | Cytochrome c oxidase subunit 5B, mitochondrial OS=Homo sapiens GN=COX5B PE=1 SV=2 | 13686 | 9.0688 | 0.324 | B | O |
| P10636 | Microtubule-associated protein tau OS=Homo sapiens GN=MAPT PE=1 SV=5 | 78879 | 6.2446 | 0.254 | B | O |
| P10768 | S-formylglutathione hydrolase OS=Homo sapiens GN=ESD PE=1 SV=2 | 31442 | 6.5815 | 0.096 | B | O |
| P10809 | 60 kDa heat shock protein, mitochondrial OS=Homo sapiens GN=HSPD1 PE=1 SV=2 | 61016 | 5.5503 | 0.804 | B | O |
| P10909 | Clusterin OS=Homo sapiens GN=CLU PE=1 SV=1 | 52461 | 5.8389 | 0.412 | B | O |
| P10915 | Hyaluronan and proteoglycan link protein 1 OS=Homo sapiens GN=HAPLN1 PE=2 SV=2 | 40139 | 7.1396 | 0.236 | B | O |
| P11021 | 78 kDa glucose-regulated protein OS=Homo sapiens GN=HSPA5 PE=1 SV=2 | 72288 | 4.875 | 0.779 | B | O |
| P11137 | Microtubule-associated protein 2 OS=Homo sapiens GN=MAP2 PE=1 SV=4 | 199402 | 4.6318 | 0.416 | B | O |
| P11142 | Heat shock cognate 71 kDa protein OS=Homo sapiens GN=HSPA8 PE=1 SV=1 | 70854 | 5.2002 | 2.287 | B | O |
| P11177 | Pyruvate dehydrogenase E1 component subunit beta, mitochondrial OS=Homo sapiens GN=PD | 39208 | 6.2021 | 0.407 | B | O |
| P11216 | Glycogen phosphorylase, brain form OS=Homo sapiens GN=PYGB PE=1 SV=5 | 96634 | 6.3999 | 0.131 | B | O |
| P11233 | Ras-related protein Ral-A OS=Homo sapiens GN=RALA PE=1 SV=1 | 23551 | 6.9858 | 0.095 | B | O |
| P11277 | Spectrin beta chain, erythrocytic OS=Homo sapiens GN=SPTB PE=1 SV=5 | 246313 | 4.9761 | 0.088 | B | O |
| P11279 | Lysosome-associated membrane glycoprotein 1 OS=Homo sapiens GN=LAMP1 PE=1 SV=3 | 44853 | 8.8374 | 0.076 | B | O |
| P11488 | Guanine nucleotide-binding protein G(t) subunit alpha-1 OS=Homo sapiens GN=GNAT1 PE=1 S | 40015 | 5.2705 | 0.119 | B | O |
| P11498 | Pyruvate carboxylase, mitochondrial OS=Homo sapiens GN=PC PE=1 SV=2 | 129551 | 6.3706 | 0.019 | B | O |
| P11766 | Alcohol dehydrogenase class-3 OS=Homo sapiens GN=ADH5 PE=1 SV=4 | 39698 | 7.3125 | 0.033 | B | O |
| P12036 | Neurofilament heavy polypeptide OS=Homo sapiens GN=NEFH PE=1 SV=4 | 112410 | 5.874 | 0.042 | B | O |
| P12235 | ADP/ATP translocase 1 OS=Homo sapiens GN=SLC25A4 PE=1 SV=4 | 33043 | 10.0723 | 0.001 | B | O |
| P12236 | ADP/ATP translocase 3 OS=Homo sapiens GN=SLC25A6 PE=1 SV=4 | 32845 | 10.062 | 0.413 | B | O |
| P12277 | Creatine kinase B-type OS=Homo sapiens GN=CKB PE=1 SV=1 | 42617 | 5.2178 | 2.043 | B | O |
| P12532 | Creatine kinase U-type, mitochondrial OS=Homo sapiens GN=CKMT1A PE=1 SV=1 | 47007 | 8.3628 | 0.398 | B | O |
| P12814 | Alpha-actinin-1 OS=Homo sapiens GN=ACTN1 PE=1 SV=2 | 102992 | 5.0933 | 0.037 | B | O |
| P12956 | X-ray repair cross-complementing protein 6 OS=Homo sapiens GN=XRCC6 PE=1 SV=2 | 69799 | 6.2036 | 0.084 | B | O |
| P13010 | X-ray repair cross-complementing protein 5 OS=Homo sapiens GN=XRCC5 PE=1 SV=3 | 82652 | 5.436 | 0.042 | B | O |
| P13073 | Cytochrome c oxidase subunit 4 isoform 1, mitochondrial OS=Homo sapiens GN=COX4I1 PE=1 | 19564 | 9.9155 | 0.216 | B | O |
| P13473 | Lysosome-associated membrane glycoprotein 2 OS=Homo sapiens GN=LAMP2 PE=1 SV=2 | 44932 | 5.231 | 0.040 | B | O |
| P13521 | Secretogranin-2 OS=Homo sapiens GN=SCG2 PE=1 SV=2 | 70897 | 4.478 | 0.313 | B | O |
| P13611 | Versican core protein OS=Homo sapiens GN=VCAN PE=1 SV=3 | 372588 | 4.2349 | 2.846 | B | O |
| P13639 | Elongation factor 2 OS=Homo sapiens GN=EEF2 PE=1 SV=4 | 95277 | 6.394 | 0.060 | B | O |
| P13645 | Keratin, type I cytoskeletal 10 OS=Homo sapiens GN=KRT10 PE=1 SV=6 | 58791 | 4.9556 | 1.531 | B | O |
| P13647 | Keratin, type II cytoskeletal 5 OS=Homo sapiens GN=KRT5 PE=1 SV=3 | 62339 | 7.793 | 0.078 | B | O |
| P13667 | Protein disulfide-isomerase A4 OS=Homo sapiens GN=PDIA4 PE=1 SV=2 | 72887 | 4.7681 | 0.039 | B | O |
| P13716 | Delta-aminolevulinic acid dehydratase OS=Homo sapiens GN=ALAD PE=1 SV=1 | 36271 | 6.3325 | 0.043 | B | O |
| P13929 | Beta-enolase OS=Homo sapiens GN=ENO3 PE=1 SV=5 | 46957 | 7.6582 | 0.005 | B | O |
| P14136 | Glial fibrillary acidic protein OS=Homo sapiens GN=GFAP PE=1 SV=1 | 49849 | 5.2559 | 22.854 | B | O |
| P14174 | Macrophage migration inhibitory factor OS=Homo sapiens GN=MIF PE=1 SV=4 | 12468 | 7.9922 | 0.165 | B | O |
| P14415 | Sodium/potassium-transporting ATPase subunit beta-2 OS=Homo sapiens GN=ATP1B2 PE=1 S | 33345 | 8.3438 | 0.503 | B | O |
| P14618 | Pyruvate kinase PKM OS=Homo sapiens GN=PKM PE=1 SV=4 | 57900 | 7.7534 | 2.103 | B | O |
| P14625 | Endoplasmin OS=Homo sapiens GN=HSP90B1 PE=1 SV=1 | 92411 | 4.5645 | 0.389 | B | O |
| P14854 | Cytochrome c oxidase subunit 6B1 OS=Homo sapiens GN=COX6B1 PE=1 SV=2 | 10185 | 6.8657 | 1.034 | B | O |
| P14868 | Aspartate--tRNA ligase, cytoplasmic OS=Homo sapiens GN=DARS PE=1 SV=2 | 57100 | 6.0894 | 0.065 | B | O |
| P14927 | Cytochrome b-c1 complex subunit 7 OS=Homo sapiens GN=UQCRB PE=1 SV=2 | 13521 | 9.2446 | 0.857 | B | O |
| P15104 | Glutamine synthetase OS=Homo sapiens GN=GLUL PE=1 SV=4 | 42037 | 6.4424 | 0.072 | B | O |
| P15121 | Aldose reductase OS=Homo sapiens GN=AKR1B1 PE=1 SV=3 | 35830 | 6.5508 | 0.043 | B | O |
| P15531 | Nucleoside diphosphate kinase A OS=Homo sapiens GN=NME1 PE=1 SV=1 | 17137 | 5.7671 | 0.254 | B | O |
| P15880 | 40S ribosomal protein S2 OS=Homo sapiens GN=RPS2 PE=1 SV=2 | 31304 | 10.6465 | 0.140 | B | O |
| P15954 | Cytochrome c oxidase subunit 7C, mitochondrial OS=Homo sapiens GN=COX7C PE=1 SV=1 | 7240 | 10.6626 | 0.256 | B | O |
| P16152 | Carbonyl reductase [NADPH] 1 OS=Homo sapiens GN=CBR1 PE=1 SV=3 | 30355 | 8.417 | 0.504 | B | O |
| P16870 | Carboxypeptidase E OS=Homo sapiens GN=CPE PE=1 SV=1 | 53117 | 4.8472 | 0.067 | B | O |
| P17066 | Heat shock 70 kDa protein 6 OS=Homo sapiens GN=HSPA6 PE=1 SV=2 | 70984 | 5.7319 | 0.755 | B | O |
| P17174 | Aspartate aminotransferase, cytoplasmic OS=Homo sapiens GN=GOT1 PE=1 SV=3 | 46218 | 6.5698 | 0.305 | B | O |
| P17252 | Protein kinase C alpha type OS=Homo sapiens GN=PRKCA PE=1 SV=4 | 76700 | 6.6138 | 0.077 | B | O |
| P17302 | Gap junction alpha-1 protein OS=Homo sapiens GN=GJA1 PE=1 SV=2 | 42980 | 8.8887 | 0.117 | B | O |
| P17540 | Creatine kinase S-type, mitochondrial OS=Homo sapiens GN=CKMT2 PE=1 SV=2 | 47474 | 8.1782 | 0.024 | B | O |
| P17600 | Synapsin-1 OS=Homo sapiens GN=SYN1 PE=1 SV=3 | 74065 | 10.1895 | 1.953 | B | O |
| P17661 | Desmin OS=Homo sapiens GN=DES PE=1 SV=3 | 53503 | 5.0303 | 0.131 | B | O |
| P17677 | Neuromodulin OS=Homo sapiens GN=GAP43 PE=1 SV=1 | 24787 | 4.4473 | 0.159 | B | O |
| P17844 | Probable ATP-dependent RNA helicase DDX5 OS=Homo sapiens GN=DDX5 PE=1 SV=1 | 69104 | 9.0996 | 0.033 | B | O |
| P17858 | ATP-dependent 6-phosphofructokinase, liver type OS=Homo sapiens GN=PFKL PE=1 SV=6 | 84964 | 7.2261 | 0.122 | B | O |
| P17987 | T-complex protein 1 subunit alpha OS=Homo sapiens GN=TCP1 PE=1 SV=1 | 60305 | 5.7129 | 0.168 | B | O |
| P18124 | 60S ribosomal protein L7 OS=Homo sapiens GN=RPL7 PE=1 SV=1 | 29207 | 11.0654 | 0.083 | B | O |
| P18669 | Phosphoglycerate mutase 1 OS=Homo sapiens GN=PGAM1 PE=1 SV=2 | 28785 | 6.7866 | 1.196 | B | O |
| P18859 | ATP synthase-coupling factor 6, mitochondrial OS=Homo sapiens GN=ATP5J PE=1 SV=1 | 12579 | 9.9829 | 0.387 | B | O |
| P19013 | Keratin, type II cytoskeletal 4 OS=Homo sapiens GN=KRT4 PE=1 SV=4 | 57249 | 6.2153 | 0.016 | B | O |
| P19022 | Cadherin-2 OS=Homo sapiens GN=CDH2 PE=1 SV=4 | 99747 | 4.4429 | 2.876 | B | O |
| P19086 | Guanine nucleotide-binding protein G(z) subunit alpha OS=Homo sapiens GN=GNAZ PE=2 SV= | 40897 | 7.4912 | 0.026 | B | O |
| P19338 | Nucleolin OS=Homo sapiens GN=NCL PE=1 SV=3 | 76568 | 4.4004 | 0.242 | B | O |
| P19367 | Hexokinase-1 OS=Homo sapiens GN=HK1 PE=1 SV=3 | 102420 | 6.3472 | 0.718 | B | O |
| P20073 | Annexin A7 OS=Homo sapiens GN=ANXA7 PE=1 SV=3 | 52705 | 5.3525 | 0.119 | B | O |
| P20336 | Ras-related protein Rab-3A OS=Homo sapiens GN=RAB3A PE=1 SV=1 | 24968 | 4.6626 | 1.392 | B | O |
| P20337 | Ras-related protein Rab-3B OS=Homo sapiens GN=RAB3B PE=1 SV=2 | 24742 | 4.6538 | 0.083 | B | O |
| P20339 | Ras-related protein Rab-5A OS=Homo sapiens GN=RAB5A PE=1 SV=2 | 23643 | 8.2515 | 0.082 | B | O |
| P20340 | Ras-related protein Rab-6A OS=Homo sapiens GN=RAB6A PE=1 SV=3 | 23577 | 5.2266 | 0.120 | B | O |

| | | | | | | |
|---|---|---|---|---|---|---|
| P20648 | Potassium-transporting ATPase alpha chain 1 OS=Homo sapiens GN=ATP4A PE=2 SV=5 | 114045 | 5.4521 | 0.230 | B | O |
| P20674 | Cytochrome c oxidase subunit 5A, mitochondrial OS=Homo sapiens GN=COX5A PE=1 SV=2 | 16751 | 6.3574 | 0.543 | B | O |
| P20718 | Granzyme H OS=Homo sapiens GN=GZMH PE=1 SV=1 | 27297 | 10.1704 | 0.155 | B | O |
| P21281 | V-type proton ATPase subunit B, brain isoform OS=Homo sapiens GN=ATP6V1B2 PE=1 SV=3 | 56464 | 5.4492 | 0.436 | B | O |
| P21291 | Cysteine and glycine-rich protein 1 OS=Homo sapiens GN=CSRP1 PE=1 SV=3 | 20553 | 8.5532 | 0.147 | B | O |
| P21397 | Amine oxidase [flavin-containing] A OS=Homo sapiens GN=MAOA PE=1 SV=1 | 59643 | 7.7842 | 0.118 | B | O |
| P21579 | Synaptotagmin-1 OS=Homo sapiens GN=SYT1 PE=1 SV=1 | 47542 | 8.2061 | 0.190 | B | O |
| P21796 | Voltage-dependent anion-selective channel protein 1 OS=Homo sapiens GN=VDAC1 PE=1 SV= | 30753 | 8.8682 | 2.396 | B | O |
| P22314 | Ubiquitin-like modifier-activating enzyme 1 OS=Homo sapiens GN=UBA1 PE=1 SV=3 | 117774 | 5.3789 | 0.198 | B | O |
| P22392 | Nucleoside diphosphate kinase B OS=Homo sapiens GN=NME2 PE=1 SV=1 | 17286 | 8.7568 | 0.366 | B | O |
| P22626 | Heterogeneous nuclear ribonucleoproteins A2/B1 OS=Homo sapiens GN=HNRNPA2B1 PE=1 S | 37406 | 9.1948 | 1.372 | B | O |
| P22695 | Cytochrome b-c1 complex subunit 2, mitochondrial OS=Homo sapiens GN=UQCRC2 PE=1 SV= | 48412 | 8.8784 | 0.356 | B | O |
| P23246 | Splicing factor, proline- and glutamine-rich OS=Homo sapiens GN=SFPQ PE=1 SV=2 | 76101 | 9.772 | 0.174 | B | O |
| P23284 | Peptidyl-prolyl cis-trans isomerase B OS=Homo sapiens GN=PPIB PE=1 SV=2 | 23727 | 9.8511 | 0.370 | B | O |
| P23297 | Protein S100-A1 OS=Homo sapiens GN=S100A1 PE=1 SV=2 | 10539 | 4.1865 | 1.063 | B | O |
| P23434 | Glycine cleavage system H protein, mitochondrial OS=Homo sapiens GN=GCSH PE=1 SV=2 | 18872 | 4.6714 | 0.026 | B | O |
| P23471 | Receptor-type tyrosine-protein phosphatase zeta OS=Homo sapiens GN=PTPRZ1 PE=1 SV=4 | 254427 | 4.5718 | 0.510 | B | O |
| P24534 | Elongation factor 1-beta OS=Homo sapiens GN=EEF1B2 PE=1 SV=3 | 24748 | 4.3037 | 0.186 | B | O |
| P24539 | ATP synthase F(0) complex subunit B1, mitochondrial OS=Homo sapiens GN=ATP5F1 PE=1 S | 28890 | 9.6563 | 0.001 | B | O |
| P24752 | Acetyl-CoA acetyltransferase, mitochondrial OS=Homo sapiens GN=ACAT1 PE=1 SV=1 | 45170 | 9.1201 | 0.218 | B | O |
| P25398 | 40S ribosomal protein S12 OS=Homo sapiens GN=RPS12 PE=1 SV=3 | 14505 | 6.9844 | 0.133 | B | O |
| P25705 | ATP synthase subunit alpha, mitochondrial OS=Homo sapiens GN=ATP5A1 PE=1 SV=1 | 59713 | 9.4321 | 2.059 | B | O |
| P25786 | Proteasome subunit alpha type-1 OS=Homo sapiens GN=PSMA1 PE=1 SV=1 | 29536 | 6.1523 | 0.050 | B | O |
| P25788 | Proteasome subunit alpha type-3 OS=Homo sapiens GN=PSMA3 PE=1 SV=2 | 28415 | 5.0171 | 0.159 | B | O |
| P26038 | Moesin OS=Homo sapiens GN=MSN PE=1 SV=3 | 67777 | 6.0103 | 0.039 | B | O |
| P26196 | Probable ATP-dependent RNA helicase DDX6 OS=Homo sapiens GN=DDX6 PE=1 SV=2 | 54382 | 8.8359 | 0.001 | B | O |
| P26641 | Elongation factor 1-gamma OS=Homo sapiens GN=EEF1G PE=1 SV=3 | 50087 | 6.2358 | 0.213 | B | O |
| P27338 | Amine oxidase [flavin-containing] B OS=Homo sapiens GN=MAOB PE=1 SV=3 | 58725 | 7.248 | 0.333 | B | O |
| P27348 | 14-3-3 protein theta OS=Homo sapiens GN=YWHAQ PE=1 SV=1 | 27746 | 4.4854 | 0.307 | B | O |
| P27482 | Calmodulin-like protein 3 OS=Homo sapiens GN=CALML3 PE=1 SV=2 | 16879 | 4.0972 | 0.884 | B | O |
| P27797 | Calreticulin OS=Homo sapiens GN=CALR PE=1 SV=1 | 48111 | 4.0942 | 0.407 | B | O |
| P27824 | Calnexin OS=Homo sapiens GN=CANX PE=1 SV=2 | 67525 | 4.2686 | 0.338 | B | O |
| P28066 | Proteasome subunit alpha type-5 OS=Homo sapiens GN=PSMA5 PE=1 SV=3 | 26394 | 4.5439 | 0.039 | B | O |
| P28072 | Proteasome subunit beta type-6 OS=Homo sapiens GN=PSMB6 PE=1 SV=4 | 25341 | 4.6069 | 0.133 | B | O |
| P28074 | Proteasome subunit beta type-5 OS=Homo sapiens GN=PSMB5 PE=1 SV=3 | 28462 | 6.5083 | 0.058 | B | O |
| P28482 | Mitogen-activated protein kinase 1 OS=Homo sapiens GN=MAPK1 PE=1 SV=3 | 41363 | 6.5317 | 0.236 | B | O |
| P28838 | Cytosol aminopeptidase OS=Homo sapiens GN=LAP3 PE=1 SV=3 | 56130 | 7.9072 | 0.100 | B | O |
| P29218 | Inositol monophosphatase 1 OS=Homo sapiens GN=IMPA1 PE=1 SV=1 | 30169 | 4.9644 | 0.084 | B | O |
| P29401 | Transketolase OS=Homo sapiens GN=TKT PE=1 SV=3 | 67834 | 7.478 | 0.407 | B | O |
| P29992 | Guanine nucleotide-binding protein subunit alpha-11 OS=Homo sapiens GN=GNA11 PE=1 SV= | 42096 | 5.3687 | 0.025 | B | O |
| P30038 | Delta-1-pyrroline-5-carboxylate dehydrogenase, mitochondrial OS=Homo sapiens GN=ALDH4A | 61680 | 8.0566 | 0.104 | B | O |
| P30040 | Endoplasmic reticulum resident protein 29 OS=Homo sapiens GN=ERP29 PE=1 SV=4 | 28975 | 7.2832 | 0.301 | B | O |
| P30041 | Peroxiredoxin-6 OS=Homo sapiens GN=PRDX6 PE=1 SV=3 | 25019 | 5.9575 | 1.793 | B | O |
| P30044 | Peroxiredoxin-5, mitochondrial OS=Homo sapiens GN=PRDX5 PE=1 SV=4 | 22072 | 8.9839 | 0.676 | B | O |
| P30048 | Thioredoxin-dependent peroxide reductase, mitochondrial OS=Homo sapiens GN=PRDX3 PE= | 27675 | 7.6934 | 0.190 | B | O |
| P30049 | ATP synthase subunit delta, mitochondrial OS=Homo sapiens GN=ATP5D PE=1 SV=2 | 17479 | 5.1914 | 1.001 | B | O |
| P30050 | 60S ribosomal protein L12 OS=Homo sapiens GN=RPL12 PE=1 SV=1 | 17807 | 9.8965 | 0.112 | B | O |
| P30084 | Enoyl-CoA hydratase, mitochondrial OS=Homo sapiens GN=ECHS1 PE=1 SV=4 | 31367 | 8.0728 | 0.180 | B | O |
| P30085 | UMP-CMP kinase OS=Homo sapiens GN=CMPK1 PE=1 SV=3 | 22208 | 5.2646 | 0.056 | B | O |
| P30086 | Phosphatidylethanolamine-binding protein 1 OS=Homo sapiens GN=PEBP1 PE=1 SV=3 | 21043 | 7.3901 | 1.665 | B | O |
| P30101 | Protein disulfide-isomerase A3 OS=Homo sapiens GN=PDIA3 PE=1 SV=4 | 56746 | 5.9312 | 0.432 | B | O |
| P31146 | Coronin-1A OS=Homo sapiens GN=CORO1A PE=1 SV=4 | 50993 | 6.2417 | 0.028 | B | O |
| P31150 | Rab GDP dissociation inhibitor alpha OS=Homo sapiens GN=GDI1 PE=1 SV=2 | 50550 | 4.8135 | 1.161 | B | O |
| P31153 | S-adenosylmethionine synthase isoform type-2 OS=Homo sapiens GN=MAT2A PE=1 SV=1 | 43633 | 6.0117 | 0.119 | B | O |
| P31689 | DnaJ homolog subfamily A member 1 OS=Homo sapiens GN=DNAJA1 PE=1 SV=2 | 44839 | 6.6709 | 0.038 | B | O |
| P31930 | Cytochrome b-c1 complex subunit 1, mitochondrial OS=Homo sapiens GN=UQCRC1 PE=1 SV= | 52612 | 5.9092 | 0.280 | B | O |
| P31939 | Bifunctional purine biosynthesis protein PURH OS=Homo sapiens GN=ATIC PE=1 SV=3 | 64575 | 6.2622 | 0.027 | B | O |
| P31942 | Heterogeneous nuclear ribonucleoprotein H3 OS=Homo sapiens GN=HNRNPH3 PE=1 SV=2 | 36903 | 6.3999 | 0.340 | B | O |
| P31946 | 14-3-3 protein beta/alpha OS=Homo sapiens GN=YWHAB PE=1 SV=3 | 28064 | 4.5674 | 1.558 | B | O |
| P31948 | Stress-induced-phosphoprotein 1 OS=Homo sapiens GN=STIP1 PE=1 SV=1 | 62599 | 6.3867 | 0.179 | B | O |
| P32119 | Peroxiredoxin-2 OS=Homo sapiens GN=PRDX2 PE=1 SV=5 | 21878 | 5.5679 | 1.469 | B | O |
| P33778 | Histone H2B type 1-B OS=Homo sapiens GN=HIST1H2BB PE=1 SV=2 | 13941 | 10.7402 | 0.040 | B | O |
| P34931 | Heat shock 70 kDa protein 1-like OS=Homo sapiens GN=HSPA1L PE=1 SV=2 | 70331 | 5.6455 | 0.956 | B | O |
| P35232 | Prohibitin OS=Homo sapiens GN=PHB PE=1 SV=1 | 29785 | 5.4302 | 0.639 | B | O |
| P35527 | Keratin, type I cytoskeletal 9 OS=Homo sapiens GN=KRT9 PE=1 SV=3 | 62026 | 4.9585 | 0.175 | B | O |
| P35609 | Alpha-actinin-2 OS=Homo sapiens GN=ACTN2 PE=1 SV=1 | 103788 | 5.1533 | 0.042 | B | O |
| P35612 | Beta-adducin OS=Homo sapiens GN=ADD2 PE=1 SV=3 | 80803 | 5.5635 | 0.093 | B | O |
| P35637 | RNA-binding protein FUS OS=Homo sapiens GN=FUS PE=1 SV=1 | 53393 | 9.4951 | 0.123 | B | O |
| P35908 | Keratin, type II cytoskeletal 2 epidermal OS=Homo sapiens GN=KRT2 PE=1 SV=2 | 65393 | 8.0537 | 0.215 | B | O |
| P36542 | ATP synthase subunit gamma, mitochondrial OS=Homo sapiens GN=ATP5C1 PE=1 SV=1 | 32975 | 9.561 | 0.356 | B | O |
| P36543 | V-type proton ATPase subunit E 1 OS=Homo sapiens GN=ATP6V1E1 PE=1 SV=1 | 26128 | 8.4419 | 0.089 | B | O |
| P36871 | Phosphoglucomutase-1 OS=Homo sapiens GN=PGM1 PE=1 SV=3 | 61410 | 6.3032 | 0.109 | B | O |
| P36957 | Dihydrolipoyllysine-residue succinyltransferase component of 2-oxoglutarate dehydrogenase co | 48724 | 9.2769 | 0.338 | B | O |
| P37802 | Transgelin-2 OS=Homo sapiens GN=TAGLN2 PE=1 SV=3 | 22377 | 8.4492 | 0.105 | B | O |
| P38606 | V-type proton ATPase catalytic subunit A OS=Homo sapiens GN=ATP6V1A PE=1 SV=2 | 68260 | 5.1855 | 0.339 | B | O |
| P38646 | Stress-70 protein, mitochondrial OS=Homo sapiens GN=HSPA9 PE=1 SV=2 | 73634 | 5.7803 | 0.935 | B | O |
| P38919 | Eukaryotic initiation factor 4A-III OS=Homo sapiens GN=EIF4A3 PE=1 SV=4 | 46841 | 6.2944 | 0.055 | B | O |
| P39019 | 40S ribosomal protein S19 OS=Homo sapiens GN=RPS19 PE=1 SV=2 | 16050 | 10.7329 | 0.145 | B | O |
| P40121 | Macrophage-capping protein OS=Homo sapiens GN=CAPG PE=1 SV=2 | 38474 | 5.7715 | 0.037 | B | O |
| P40227 | T-complex protein 1 subunit zeta OS=Homo sapiens GN=CCT6A PE=1 SV=3 | 57987 | 6.2241 | 0.191 | B | O |
| P40925 | Malate dehydrogenase, cytoplasmic OS=Homo sapiens GN=MDH1 PE=1 SV=4 | 36403 | 7.1704 | 1.138 | B | O |

| | | | | | | |
|---|---|---|---|---|---|---|
| P40926 | Malate dehydrogenase, mitochondrial OS=Homo sapiens GN=MDH2 PE=1 SV=3 | 35480 | 8.8213 | 0.925 | B | O |
| P40939 | Trifunctional enzyme subunit alpha, mitochondrial OS=Homo sapiens GN=HADHA PE=1 SV=2 | 82946 | 9.3413 | 0.081 | B | O |
| P41219 | Peripherin OS=Homo sapiens GN=PRPH PE=1 SV=2 | 53618 | 5.209 | 0.077 | B | O |
| P41222 | Prostaglandin-H2 D-isomerase OS=Homo sapiens GN=PTGDS PE=1 SV=1 | 21015 | 7.8325 | 0.255 | B | O |
| P41240 | Tyrosine-protein kinase CSK OS=Homo sapiens GN=CSK PE=1 SV=1 | 50671 | 6.6431 | 0.098 | B | O |
| P41250 | Glycine--tRNA ligase OS=Homo sapiens GN=GARS PE=1 SV=3 | 83112 | 6.6138 | 0.056 | B | O |
| P42126 | Enoyl-CoA delta isomerase 1, mitochondrial OS=Homo sapiens GN=ECI1 PE=1 SV=1 | 32795 | 8.6821 | 0.028 | B | O |
| P42166 | Lamina-associated polypeptide 2, isoform alpha OS=Homo sapiens GN=TMPO PE=1 SV=2 | 75445 | 7.5132 | 0.048 | B | O |
| P42262 | Glutamate receptor 2 OS=Homo sapiens GN=GRIA2 PE=1 SV=3 | 98758 | 7.4341 | 0.022 | B | O |
| P43004 | Excitatory amino acid transporter 2 OS=Homo sapiens GN=SLC1A2 PE=1 SV=2 | 62063 | 6.0762 | 0.431 | B | O |
| P43007 | Neutral amino acid transporter A OS=Homo sapiens GN=SLC1A4 PE=1 SV=1 | 55687 | 5.8315 | 0.030 | B | O |
| P43304 | Glycerol-3-phosphate dehydrogenase, mitochondrial OS=Homo sapiens GN=GPD2 PE=1 SV=3 | 80801 | 7.5454 | 0.101 | B | O |
| P45880 | Voltage-dependent anion-selective channel protein 2 OS=Homo sapiens GN=VDAC2 PE=1 SV= | 31546 | 7.4678 | 1.002 | B | O |
| P46531 | Neurogenic locus notch homolog protein 1 OS=Homo sapiens GN=NOTCH1 PE=1 SV=4 | 272320 | 4.7798 | 0.097 | B | O |
| P46777 | 60S ribosomal protein L5 OS=Homo sapiens GN=RPL5 PE=1 SV=3 | 34340 | 10.0151 | 0.108 | B | O |
| P46783 | 40S ribosomal protein S10 OS=Homo sapiens GN=RPS10 PE=1 SV=1 | 18885 | 10.5088 | 0.442 | B | O |
| P46821 | Microtubule-associated protein 1B OS=Homo sapiens GN=MAP1B PE=1 SV=2 | 270465 | 4.5381 | 0.361 | B | O |
| P47755 | F-actin-capping protein subunit alpha-2 OS=Homo sapiens GN=CAPZA2 PE=1 SV=3 | 32928 | 5.4756 | 0.115 | B | O |
| P47985 | Cytochrome b-c1 complex subunit Rieske, mitochondrial OS=Homo sapiens GN=UQCRFS1 PE | 29649 | 8.3936 | 0.351 | B | O |
| P48047 | ATP synthase subunit O, mitochondrial OS=Homo sapiens GN=ATP5O PE=1 SV=1 | 23262 | 10.3638 | 0.730 | B | O |
| P48735 | Isocitrate dehydrogenase [NADP], mitochondrial OS=Homo sapiens GN=IDH2 PE=1 SV=2 | 50876 | 8.855 | 0.267 | B | O |
| P49006 | MARCKS-related protein OS=Homo sapiens GN=MARCKSL1 PE=1 SV=2 | 19517 | 4.4531 | 0.166 | B | O |
| P49189 | 4-trimethylaminobutyraldehyde dehydrogenase OS=Homo sapiens GN=ALDH9A1 PE=1 SV=3 | 53767 | 5.562 | 0.167 | B | O |
| P49411 | Elongation factor Tu, mitochondrial OS=Homo sapiens GN=TUFM PE=1 SV=2 | 49510 | 7.3726 | 0.259 | B | O |
| P49418 | Amphiphysin OS=Homo sapiens GN=AMPH PE=1 SV=1 | 76210 | 4.374 | 0.242 | B | O |
| P49419 | Alpha-aminoadipic semialdehyde dehydrogenase OS=Homo sapiens GN=ALDH7A1 PE=1 SV= | 58450 | 7.938 | 0.408 | B | O |
| P49458 | Signal recognition particle 9 kDa protein OS=Homo sapiens GN=SRP9 PE=1 SV=2 | 10105 | 8.2163 | 0.134 | B | O |
| P49721 | Proteasome subunit beta type-2 OS=Homo sapiens GN=PSMB2 PE=1 SV=1 | 22821 | 6.6079 | 0.119 | B | O |
| P49748 | Very long-chain specific acyl-CoA dehydrogenase, mitochondrial OS=Homo sapiens GN=ACAD | 70345 | 8.9912 | 0.020 | B | O |
| P50135 | Histamine N-methyltransferase OS=Homo sapiens GN=HNMT PE=1 SV=1 | 33273 | 5.0127 | 0.072 | B | O |
| P50150 | Guanine nucleotide-binding protein G(I)/G(S)/G(O) subunit gamma-4 OS=Homo sapiens GN=G | 8383 | 6.9375 | 0.060 | B | O |
| P50395 | Rab GDP dissociation inhibitor beta OS=Homo sapiens GN=GDI2 PE=1 SV=2 | 50630 | 6.0557 | 0.186 | B | O |
| P50502 | Hsc70-interacting protein OS=Homo sapiens GN=ST13 PE=1 SV=2 | 41305 | 4.9966 | 0.169 | B | O |
| P50570 | Dynamin-2 OS=Homo sapiens GN=DNM2 PE=1 SV=2 | 98003 | 7.1162 | 0.178 | B | O |
| P50897 | Palmitoyl-protein thioesterase 1 OS=Homo sapiens GN=PPT1 PE=1 SV=1 | 34171 | 6.0659 | 0.366 | B | O |
| P50990 | T-complex protein 1 subunit theta OS=Homo sapiens GN=CCT8 PE=1 SV=4 | 59582 | 5.272 | 0.564 | B | O |
| P50991 | T-complex protein 1 subunit delta OS=Homo sapiens GN=CCT4 PE=1 SV=4 | 57887 | 7.7827 | 0.124 | B | O |
| P51148 | Ras-related protein Rab-5C OS=Homo sapiens GN=RAB5C PE=1 SV=2 | 23467 | 8.5796 | 0.178 | B | O |
| P51149 | Ras-related protein Rab-7a OS=Homo sapiens GN=RAB7A PE=1 SV=1 | 23474 | 6.5742 | 0.536 | B | O |
| P51665 | 26S proteasome non-ATPase regulatory subunit 7 OS=Homo sapiens GN=PSMD7 PE=1 SV=2 | 37002 | 6.3003 | 1.616 | B | O |
| P51970 | NADH dehydrogenase [ubiquinone] 1 alpha subcomplex subunit 8 OS=Homo sapiens GN=NDU | 20092 | 7.5483 | 0.210 | B | O |
| P51991 | Heterogeneous nuclear ribonucleoprotein A3 OS=Homo sapiens GN=HNRNPA3 PE=1 SV=2 | 39570 | 9.2212 | 0.546 | B | O |
| P52209 | 6-phosphogluconate dehydrogenase, decarboxylating OS=Homo sapiens GN=PGD PE=1 SV=3 | 53105 | 6.8584 | 0.106 | B | O |
| P52333 | Tyrosine-protein kinase JAK3 OS=Homo sapiens GN=JAK3 PE=1 SV=2 | 125019 | 6.7529 | 0.724 | B | O |
| P52565 | Rho GDP-dissociation inhibitor 1 OS=Homo sapiens GN=ARHGDIA PE=1 SV=3 | 23192 | 4.8179 | 0.412 | B | O |
| P52758 | Ribonuclease UK114 OS=Homo sapiens GN=HRSP12 PE=1 SV=1 | 14484 | 9.1289 | 0.165 | B | O |
| P52907 | F-actin-capping protein subunit alpha-1 OS=Homo sapiens GN=CAPZA1 PE=1 SV=3 | 32902 | 5.3262 | 0.050 | B | O |
| P52943 | Cysteine-rich protein 2 OS=Homo sapiens GN=CRIP2 PE=1 SV=1 | 22478 | 8.7393 | 0.053 | B | O |
| P53597 | Succinyl-CoA ligase [ADP/GDP-forming] subunit alpha, mitochondrial OS=Homo sapiens GN=S | 36226 | 8.978 | 0.139 | B | O |
| P53999 | Activated RNA polymerase II transcriptional coactivator p15 OS=Homo sapiens GN=SUB1 PE= | 14386 | 10.0737 | 0.890 | B | O |
| P54289 | Voltage-dependent calcium channel subunit alpha-2/delta-1 OS=Homo sapiens GN=CACNA2D | 124489 | 4.9365 | 0.063 | B | O |
| P54652 | Heat shock-related 70 kDa protein 2 OS=Homo sapiens GN=HSPA2 PE=1 SV=1 | 69977 | 5.4082 | 1.132 | B | O |
| P54707 | Potassium-transporting ATPase alpha chain 2 OS=Homo sapiens GN=ATP12A PE=1 SV=3 | 115437 | 6.0981 | 3.101 | B | O |
| P55072 | Transitional endoplasmic reticulum ATPase OS=Homo sapiens GN=VCP PE=1 SV=4 | 89265 | 4.9556 | 0.227 | B | O |
| P55087 | Aquaporin-4 OS=Homo sapiens GN=AQP4 PE=1 SV=2 | 34806 | 7.5557 | 0.001 | B | O |
| P55327 | Tumor protein D52 OS=Homo sapiens GN=TPD52 PE=1 SV=2 | 24312 | 4.5908 | 0.050 | B | O |
| P55786 | Puromycin-sensitive aminopeptidase OS=Homo sapiens GN=NPEPPS PE=1 SV=2 | 103210 | 5.3599 | 0.204 | B | O |
| P55795 | Heterogeneous nuclear ribonucleoprotein H2 OS=Homo sapiens GN=HNRNPH2 PE=1 SV=1 | 49232 | 5.8521 | 0.232 | B | O |
| P55809 | Succinyl-CoA:3-ketoacid coenzyme A transferase 1, mitochondrial OS=Homo sapiens GN=OXC | 56121 | 7.2202 | 0.101 | B | O |
| P56385 | ATP synthase subunit e, mitochondrial OS=Homo sapiens GN=ATP5I PE=1 SV=2 | 7928 | 9.7412 | 2.173 | B | O |
| P58546 | Myotrophin OS=Homo sapiens GN=MTPN PE=1 SV=2 | 12886 | 5.1343 | 0.518 | B | O |
| P60174 | Triosephosphate isomerase OS=Homo sapiens GN=TPI1 PE=1 SV=3 | 30771 | 5.5474 | 1.476 | B | O |
| P60201 | Myelin proteolipid protein OS=Homo sapiens GN=PLP1 PE=1 SV=2 | 30057 | 8.2881 | 0.949 | B | O |
| P60709 | Actin, cytoplasmic 1 OS=Homo sapiens GN=ACTB PE=1 SV=1 | 41709 | 5.1431 | 7.802 | B | O |
| P60763 | Ras-related C3 botulinum toxin substrate 3 OS=Homo sapiens GN=RAC3 PE=1 SV=1 | 21365 | 8.1592 | 0.001 | B | O |
| P60880 | Synaptosomal-associated protein 25 OS=Homo sapiens GN=SNAP25 PE=1 SV=1 | 23300 | 4.4575 | 0.404 | B | O |
| P60953 | Cell division control protein 42 homolog OS=Homo sapiens GN=CDC42 PE=1 SV=2 | 21245 | 6.1538 | 0.070 | B | O |
| P60981 | Destrin OS=Homo sapiens GN=DSTN PE=1 SV=3 | 18493 | 7.8384 | 0.623 | B | O |
| P61019 | Ras-related protein Rab-2A OS=Homo sapiens GN=RAB2A PE=1 SV=1 | 23530 | 6.0806 | 0.519 | B | O |
| P61020 | Ras-related protein Rab-5B OS=Homo sapiens GN=RAB5B PE=1 SV=1 | 23691 | 8.2383 | 0.129 | B | O |
| P61026 | Ras-related protein Rab-10 OS=Homo sapiens GN=RAB10 PE=1 SV=1 | 22526 | 8.5737 | 0.135 | B | O |
| P61088 | Ubiquitin-conjugating enzyme E2 N OS=Homo sapiens GN=UBE2N PE=1 SV=1 | 17126 | 6.1494 | 0.455 | B | O |
| P61160 | Actin-related protein 2 OS=Homo sapiens GN=ACTR2 PE=1 SV=1 | 44732 | 6.2959 | 0.094 | B | O |
| P61163 | Alpha-centractin OS=Homo sapiens GN=ACTR1A PE=1 SV=1 | 42586 | 6.1846 | 0.045 | B | O |
| P61204 | ADP-ribosylation factor 3 OS=Homo sapiens GN=ARF3 PE=1 SV=2 | 20587 | 7.3887 | 0.001 | B | O |
| P61224 | Ras-related protein Rap-1b OS=Homo sapiens GN=RAP1B PE=1 SV=1 | 20811 | 5.4653 | 0.001 | B | O |
| P61225 | Ras-related protein Rap-2b OS=Homo sapiens GN=RAP2B PE=1 SV=1 | 20491 | 4.5278 | 0.079 | B | O |
| P61266 | Syntaxin-1B OS=Homo sapiens GN=STX1B PE=1 SV=1 | 33223 | 5.0859 | 0.345 | B | O |
| P61586 | Transforming protein RhoA OS=Homo sapiens GN=RHOA PE=1 SV=1 | 21754 | 5.7305 | 0.147 | D | Y |
| P61604 | 10 kDa heat shock protein, mitochondrial OS=Homo sapiens GN=HSPE1 PE=1 SV=2 | 10924 | 9.4702 | 0.421 | B | O |
| P61764 | Syntaxin-binding protein 1 OS=Homo sapiens GN=STXBP1 PE=1 SV=1 | 67525 | 6.5098 | 0.785 | B | O |

| | | | | | | |
|---|---|---|---|---|---|---|
| P61978 | Heterogeneous nuclear ribonucleoprotein K OS=Homo sapiens GN=HNRNPK PE=1 SV=1 | 50944 | 5.2207 | 0.613 | B | O |
| P61981 | 14-3-3 protein gamma OS=Homo sapiens GN=YWHAG PE=1 SV=2 | 28284 | 4.6069 | 2.942 | B | O |
| P62081 | 40S ribosomal protein S7 OS=Homo sapiens GN=RPS7 PE=1 SV=1 | 22113 | 10.582 | 0.172 | B | O |
| P62158 | Calmodulin OS=Homo sapiens GN=CALM1 PE=1 SV=2 | 16826 | 3.8833 | 2.179 | B | O |
| P62166 | Neuronal calcium sensor 1 OS=Homo sapiens GN=NCS1 PE=1 SV=2 | 21864 | 4.5088 | 0.242 | B | O |
| P62191 | 26S protease regulatory subunit 4 OS=Homo sapiens GN=PSMC1 PE=1 SV=1 | 49153 | 5.7993 | 0.042 | B | O |
| P62241 | 40S ribosomal protein S8 OS=Homo sapiens GN=RPS8 PE=1 SV=2 | 24190 | 10.7153 | 0.001 | B | O |
| P62258 | 14-3-3 protein epsilon OS=Homo sapiens GN=YWHAE PE=1 SV=1 | 29155 | 4.4355 | 2.157 | B | O |
| P62269 | 40S ribosomal protein S18 OS=Homo sapiens GN=RPS18 PE=1 SV=3 | 17707 | 11.4141 | 0.082 | B | O |
| P62304 | Small nuclear ribonucleoprotein E OS=Homo sapiens GN=SNRPE PE=1 SV=1 | 10796 | 9.7808 | 0.091 | B | O |
| P62330 | ADP-ribosylation factor 6 OS=Homo sapiens GN=ARF6 PE=1 SV=2 | 20069 | 9.293 | 0.055 | B | O |
| P62424 | 60S ribosomal protein L7a OS=Homo sapiens GN=RPL7A PE=1 SV=2 | 29977 | 11.0581 | 1.989 | B | O |
| P62701 | 40S ribosomal protein S4, X isoform OS=Homo sapiens GN=RPS4X PE=1 SV=2 | 29579 | 10.5864 | 0.196 | B | O |
| P62714 | Serine/threonine-protein phosphatase 2A catalytic subunit beta isoform OS=Homo sapiens GN= | 35552 | 5.061 | 0.001 | B | O |
| P62745 | Rho-related GTP-binding protein RhoB OS=Homo sapiens GN=RHOB PE=1 SV=1 | 22109 | 4.9146 | 0.135 | B | O |
| P62760 | Visinin-like protein 1 OS=Homo sapiens GN=VSNL1 PE=1 SV=2 | 22127 | 4.8164 | 0.106 | B | O |
| P62805 | Histone H4 OS=Homo sapiens GN=HIST1H4A PE=1 SV=2 | 11360 | 11.7671 | 1.206 | B | O |
| P62807 | Histone H2B type 1-C/E/F/G/I OS=Homo sapiens GN=HIST1H2BC PE=1 SV=4 | 13897 | 10.7402 | 0.352 | B | O |
| P62857 | 40S ribosomal protein S28 OS=Homo sapiens GN=RPS28 PE=1 SV=1 | 7836 | 11.1504 | 0.641 | B | O |
| P62873 | Guanine nucleotide-binding protein G(I)/G(S)/G(T) subunit beta-1 OS=Homo sapiens GN=GNB1 | 37353 | 5.5356 | 0.476 | B | O |
| P62879 | Guanine nucleotide-binding protein G(I)/G(S)/G(T) subunit beta-2 OS=Homo sapiens GN=GNB2 | 37307 | 5.5356 | 0.074 | B | O |
| P62937 | Peptidyl-prolyl cis-trans isomerase A OS=Homo sapiens GN=PPIA PE=1 SV=2 | 18000 | 7.853 | 1.157 | B | O |
| P62942 | Peptidyl-prolyl cis-trans isomerase FKBP1A OS=Homo sapiens GN=FKBP1A PE=1 SV=2 | 11943 | 8.6558 | 1.382 | B | O |
| P62987 | Ubiquitin-60S ribosomal protein L40 OS=Homo sapiens GN=UBA52 PE=1 SV=2 | 14718 | 10.2656 | 1.081 | B | O |
| P62993 | Growth factor receptor-bound protein 2 OS=Homo sapiens GN=GRB2 PE=1 SV=1 | 25190 | 5.8579 | 0.085 | B | O |
| P63092 | Guanine nucleotide-binding protein G(s) subunit alpha isoforms short OS=Homo sapiens GN=G | 45635 | 5.4668 | 0.013 | B | O |
| P63096 | Guanine nucleotide-binding protein G(i) subunit alpha-1 OS=Homo sapiens GN=GNAI1 PE=1 S | 40335 | 5.5913 | 0.081 | B | O |
| P63151 | Serine/threonine-protein phosphatase 2A 55 kDa regulatory subunit B alpha isoform OS=Homo | 51659 | 5.7642 | 0.381 | B | O |
| P63167 | Dynein light chain 1, cytoplasmic OS=Homo sapiens GN=DYNLL1 PE=1 SV=1 | 10359 | 7.2246 | 0.278 | B | O |
| P63267 | Actin, gamma-enteric smooth muscle OS=Homo sapiens GN=ACTG2 PE=1 SV=1 | 41849 | 5.1606 | 0.186 | B | O |
| P67936 | Tropomyosin alpha-4 chain OS=Homo sapiens GN=TPM4 PE=1 SV=3 | 28504 | 4.4707 | 0.253 | B | O |
| P68032 | Actin, alpha cardiac muscle 1 OS=Homo sapiens GN=ACTC1 PE=1 SV=1 | 41991 | 5.0713 | 0.426 | B | O |
| P68036 | Ubiquitin-conjugating enzyme E2 L3 OS=Homo sapiens GN=UBE2L3 PE=1 SV=1 | 17850 | 8.7993 | 0.082 | B | O |
| P68104 | Elongation factor 1-alpha 1 OS=Homo sapiens GN=EEF1A1 PE=1 SV=1 | 50109 | 9.3428 | 0.178 | B | O |
| P68363 | Tubulin alpha-1B chain OS=Homo sapiens GN=TUBA1B PE=1 SV=1 | 50119 | 4.7622 | 2.627 | B | O |
| P68366 | Tubulin alpha-4A chain OS=Homo sapiens GN=TUBA4A PE=1 SV=1 | 49892 | 4.752 | 2.398 | B | O |
| P68371 | Tubulin beta-4B chain OS=Homo sapiens GN=TUBB4B PE=1 SV=1 | 49799 | 4.6025 | 2.550 | B | O |
| P68871 | Hemoglobin subunit beta OS=Homo sapiens GN=HBB PE=1 SV=2 | 15988 | 6.8804 | 2.695 | B | O |
| P69905 | Hemoglobin subunit alpha OS=Homo sapiens GN=HBA1 PE=1 SV=2 | 15247 | 9.1787 | 5.382 | B | O |
| P78324 | Tyrosine-protein phosphatase non-receptor type substrate 1 OS=Homo sapiens GN=SIRPA PE | 54932 | 6.5361 | 0.197 | B | O |
| P78357 | Contactin-associated protein 1 OS=Homo sapiens GN=CNTNAP1 PE=1 SV=1 | 156166 | 6.5991 | 0.064 | B | O |
| P78559 | Microtubule-associated protein 1A OS=Homo sapiens GN=MAP1A PE=1 SV=6 | 305296 | 4.6567 | 0.108 | B | O |
| P80723 | Brain acid soluble protein 1 OS=Homo sapiens GN=BASP1 PE=1 SV=2 | 22680 | 4.4238 | 2.552 | B | O |
| P98179 | RNA-binding protein 3 OS=Homo sapiens GN=RBM3 PE=1 SV=1 | 17159 | 9.1919 | 0.084 | B | O |
| Q00325 | Phosphate carrier protein, mitochondrial OS=Homo sapiens GN=SLC25A3 PE=1 SV=2 | 40068 | 9.6387 | 0.486 | B | O |
| Q00577 | Transcriptional activator protein Pur-alpha OS=Homo sapiens GN=PURA PE=1 SV=2 | 34889 | 6.0249 | 0.455 | B | O |
| Q00688 | Peptidyl-prolyl cis-trans isomerase FKBP3 OS=Homo sapiens GN=FKBP3 PE=1 SV=1 | 25161 | 9.7324 | 0.091 | B | O |
| Q00839 | Heterogeneous nuclear ribonucleoprotein U OS=Homo sapiens GN=HNRNPU PE=1 SV=6 | 90527 | 5.6484 | 0.178 | B | O |
| Q01082 | Spectrin beta chain, non-erythrocytic 1 OS=Homo sapiens GN=SPTBN1 PE=1 SV=2 | 274437 | 5.2515 | 0.850 | B | O |
| Q01469 | Fatty acid-binding protein, epidermal OS=Homo sapiens GN=FABP5 PE=1 SV=3 | 15154 | 6.8042 | 0.138 | B | O |
| Q01484 | Ankyrin-2 OS=Homo sapiens GN=ANK2 PE=1 SV=4 | 433447 | 4.8516 | 1.041 | B | O |
| Q01518 | Adenylyl cyclase-associated protein 1 OS=Homo sapiens GN=CAP1 PE=1 SV=5 | 51868 | 8.0581 | 0.136 | B | O |
| Q01546 | Keratin, type II cytoskeletal 2 oral OS=Homo sapiens GN=KRT76 PE=1 SV=2 | 65800 | 8.0991 | 0.044 | B | O |
| Q01813 | ATP-dependent 6-phosphofructokinase, platelet type OS=Homo sapiens GN=PFKP PE=1 SV=2 | 85541 | 7.3535 | 0.213 | B | O |
| Q02252 | Methylmalonate-semialdehyde dehydrogenase [acylating], mitochondrial OS=Homo sapiens GN | 57802 | 8.584 | 0.259 | B | O |
| Q02750 | Dual specificity mitogen-activated protein kinase kinase 1 OS=Homo sapiens GN=MAP2K1 PE= | 43411 | 6.1772 | 0.226 | B | O |
| Q02818 | Nucleobindin-1 OS=Homo sapiens GN=NUCB1 PE=1 SV=4 | 53846 | 4.9805 | 0.075 | B | O |
| Q02978 | Mitochondrial 2-oxoglutarate/malate carrier protein OS=Homo sapiens GN=SLC25A11 PE=1 SV | 34039 | 10.2085 | 0.431 | B | O |
| Q03013 | Glutathione S-transferase Mu 4 OS=Homo sapiens GN=GSTM4 PE=1 SV=3 | 25544 | 5.502 | 0.055 | B | O |
| Q03252 | Lamin-B2 OS=Homo sapiens GN=LMNB2 PE=1 SV=3 | 67647 | 5.1064 | 0.126 | B | O |
| Q04760 | Lactoylglutathione lyase OS=Homo sapiens GN=GLO1 PE=1 SV=4 | 20764 | 4.9424 | 0.126 | B | O |
| Q04837 | Single-stranded DNA-binding protein, mitochondrial OS=Homo sapiens GN=SSBP1 PE=1 SV=1 | 17249 | 9.9111 | 0.468 | B | O |
| Q04917 | 14-3-3 protein eta OS=Homo sapiens GN=YWHAH PE=1 SV=4 | 28201 | 4.5615 | 1.299 | B | O |
| Q05639 | Elongation factor 1-alpha 2 OS=Homo sapiens GN=EEF1A2 PE=1 SV=1 | 50438 | 9.3472 | 0.371 | B | O |
| Q07021 | Complement component 1 Q subcomponent-binding protein, mitochondrial OS=Homo sapiens ( | 31342 | 4.5469 | 0.336 | B | O |
| Q07065 | Cytoskeleton-associated protein 4 OS=Homo sapiens GN=CKAP4 PE=1 SV=2 | 65982 | 5.5415 | 0.114 | B | O |
| Q07666 | KH domain-containing, RNA-binding, signal transduction-associated protein 1 OS=Homo sapien | 48197 | 8.9136 | 0.192 | B | O |
| Q08211 | ATP-dependent RNA helicase A OS=Homo sapiens GN=DHX9 PE=1 SV=4 | 140868 | 6.3955 | 0.042 | B | O |
| Q08257 | Quinone oxidoreductase OS=Homo sapiens GN=CRYZ PE=1 SV=1 | 35184 | 8.6602 | 0.037 | B | O |
| Q08722 | Leukocyte surface antigen CD47 OS=Homo sapiens GN=CD47 PE=1 SV=1 | 35190 | 6.9844 | 0.181 | B | O |
| Q10567 | AP-1 complex subunit beta-1 OS=Homo sapiens GN=AP1B1 PE=1 SV=2 | 104570 | 4.749 | 0.045 | B | O |
| Q12765 | Secernin-1 OS=Homo sapiens GN=SCRN1 PE=1 SV=2 | 46352 | 4.4707 | 0.101 | B | O |
| Q12772 | Sterol regulatory element-binding protein 2 OS=Homo sapiens GN=SREBF2 PE=1 SV=2 | 123609 | 8.332 | 0.122 | B | O |
| Q12860 | Contactin-1 OS=Homo sapiens GN=CNTN1 PE=1 SV=1 | 113249 | 5.5137 | 0.522 | B | O |
| Q12931 | Heat shock protein 75 kDa, mitochondrial OS=Homo sapiens GN=TRAP1 PE=1 SV=3 | 80059 | 8.2852 | 0.793 | B | O |
| Q12955 | Ankyrin-3 OS=Homo sapiens GN=ANK3 PE=1 SV=3 | 480112 | 6.0396 | 0.058 | B | O |
| Q13011 | Delta(3,5)-Delta(2,4)-dienoyl-CoA isomerase, mitochondrial OS=Homo sapiens GN=ECH1 PE= | 35793 | 7.9995 | 0.241 | B | O |
| Q13151 | Heterogeneous nuclear ribonucleoprotein A0 OS=Homo sapiens GN=HNRNPA0 PE=1 SV=1 | 30821 | 9.5493 | 0.258 | B | O |
| Q13228 | Selenium-binding protein 1 OS=Homo sapiens GN=SELENBP1 PE=1 SV=2 | 52357 | 5.9063 | 0.090 | B | O |
| Q13315 | Serine-protein kinase ATM OS=Homo sapiens GN=ATM PE=1 SV=4 | 350460 | 6.3647 | 3.412 | B | O |
| Q13332 | Receptor-type tyrosine-protein phosphatase S OS=Homo sapiens GN=PTPRS PE=1 SV=3 | 216903 | 6.0176 | 0.442 | B | O |

| | | | | | | |
|---|---|---|---|---|---|---|
| Q13347 | Eukaryotic translation initiation factor 3 subunit I OS=Homo sapiens GN=EIF3I PE=1 SV=1 | 36478 | 5.2617 | 0.027 | B | O |
| Q13423 | NAD(P) transhydrogenase, mitochondrial OS=Homo sapiens GN=NNT PE=1 SV=3 | 113822 | 8.0566 | 0.169 | B | O |
| Q13492 | Phosphatidylinositol-binding clathrin assembly protein OS=Homo sapiens GN=PICALM PE=1 SV | 70710 | 7.9556 | 0.243 | B | O |
| Q13509 | Tubulin beta-3 chain OS=Homo sapiens GN=TUBB3 PE=1 SV=2 | 50400 | 4.6392 | 0.922 | B | O |
| Q13554 | Calcium/calmodulin-dependent protein kinase type II subunit beta OS=Homo sapiens GN=CAMI | 72632 | 6.8818 | 0.115 | B | O |
| Q13561 | Dynactin subunit 2 OS=Homo sapiens GN=DCTN2 PE=1 SV=4 | 44203 | 4.9248 | 0.063 | B | O |
| Q13740 | CD166 antigen OS=Homo sapiens GN=ALCAM PE=1 SV=2 | 65061 | 5.8477 | 0.041 | B | O |
| Q13825 | Methylglutaconyl-CoA hydratase, mitochondrial OS=Homo sapiens GN=AUH PE=1 SV=1 | 35586 | 9.8687 | 0.080 | B | O |
| Q13838 | Spliceosome RNA helicase DDX39B OS=Homo sapiens GN=DDX39B PE=1 SV=1 | 48960 | 5.313 | 0.199 | B | O |
| Q13885 | Tubulin beta-2A chain OS=Homo sapiens GN=TUBB2A PE=1 SV=1 | 49874 | 4.5908 | 3.255 | B | O |
| Q14011 | Cold-inducible RNA-binding protein OS=Homo sapiens GN=CIRBP PE=1 SV=1 | 18636 | 9.7397 | 0.303 | B | O |
| Q14019 | Coactosin-like protein OS=Homo sapiens GN=COTL1 PE=1 SV=3 | 15935 | 5.3408 | 0.132 | B | O |
| Q14108 | Lysosome membrane protein 2 OS=Homo sapiens GN=SCARB2 PE=1 SV=2 | 54255 | 4.8179 | 0.053 | B | O |
| Q14195 | Dihydropyrimidinase-related protein 3 OS=Homo sapiens GN=DPYSL3 PE=1 SV=1 | 61924 | 6.0249 | 0.261 | B | O |
| Q14240 | Eukaryotic initiation factor 4A-II OS=Homo sapiens GN=EIF4A2 PE=1 SV=2 | 46372 | 5.1636 | 0.169 | B | O |
| Q14247 | Src substrate cortactin OS=Homo sapiens GN=CTTN PE=1 SV=2 | 61548 | 5.0771 | 0.072 | B | O |
| Q14257 | Reticulocalbin-2 OS=Homo sapiens GN=RCN2 PE=1 SV=1 | 36853 | 4.0635 | 0.158 | B | O |
| Q14568 | Heat shock protein HSP 90-alpha A2 OS=Homo sapiens GN=HSP90AA2P PE=1 SV=2 | 39340 | 4.377 | 0.070 | B | O |
| Q14894 | Ketimine reductase mu-crystallin OS=Homo sapiens GN=CRYM PE=1 SV=1 | 33754 | 4.8765 | 1.819 | B | O |
| Q14CZ8 | Hepatocyte cell adhesion molecule OS=Homo sapiens GN=HEPACAM PE=1 SV=1 | 45998 | 9.3999 | 0.474 | B | O |
| Q15019 | Septin-2 OS=Homo sapiens GN=SEPT2 PE=1 SV=1 | 41461 | 6.1392 | 0.146 | B | O |
| Q15029 | 116 kDa U5 small nuclear ribonucleoprotein component OS=Homo sapiens GN=EFTUD2 PE=1 | 109366 | 4.6582 | 0.074 | B | O |
| Q15056 | Eukaryotic translation initiation factor 4H OS=Homo sapiens GN=EIF4H PE=1 SV=5 | 27368 | 7.2026 | 0.307 | B | O |
| Q15084 | Protein disulfide-isomerase A6 OS=Homo sapiens GN=PDIA6 PE=1 SV=1 | 48091 | 4.7622 | 0.344 | B | O |
| Q15181 | Inorganic pyrophosphatase OS=Homo sapiens GN=PPA1 PE=1 SV=2 | 32639 | 5.4419 | 0.038 | B | O |
| Q15233 | Non-POU domain-containing octamer-binding protein OS=Homo sapiens GN=NONO PE=1 SV= | 54197 | 9.3691 | 0.043 | B | O |
| Q15286 | Ras-related protein Rab-35 OS=Homo sapiens GN=RAB35 PE=1 SV=1 | 23010 | 8.3936 | 0.142 | B | O |
| Q15293 | Reticulocalbin-1 OS=Homo sapiens GN=RCN1 PE=1 SV=1 | 38866 | 4.6729 | 0.131 | B | O |
| Q15365 | Poly(rC)-binding protein 1 OS=Homo sapiens GN=PCBP1 PE=1 SV=2 | 37473 | 6.7148 | 0.101 | B | O |
| Q15366 | Poly(rC)-binding protein 2 OS=Homo sapiens GN=PCBP2 PE=1 SV=1 | 38555 | 6.3472 | 0.139 | B | O |
| Q15555 | Microtubule-associated protein RP/EB family member 2 OS=Homo sapiens GN=MAPRE2 PE=1 | 37008 | 5.2324 | 0.077 | B | O |
| Q15717 | ELAV-like protein 1 OS=Homo sapiens GN=ELAVL1 PE=1 SV=2 | 36069 | 9.4702 | 0.040 | B | O |
| Q16143 | Beta-synuclein OS=Homo sapiens GN=SNCB PE=1 SV=1 | 14279 | 4.21 | 0.631 | B | O |
| Q16555 | Dihydropyrimidinase-related protein 2 OS=Homo sapiens GN=DPYSL2 PE=1 SV=1 | 62254 | 5.9238 | 2.073 | B | O |
| Q16643 | Drebrin OS=Homo sapiens GN=DBN1 PE=1 SV=4 | 71385 | 4.2026 | 0.111 | B | O |
| Q16658 | Fascin OS=Homo sapiens GN=FSCN1 PE=1 SV=3 | 54496 | 6.876 | 0.079 | B | O |
| Q16698 | 2,4-dienoyl-CoA reductase, mitochondrial OS=Homo sapiens GN=DECR1 PE=1 SV=1 | 36044 | 9.6606 | 0.038 | B | O |
| Q16720 | Plasma membrane calcium-transporting ATPase 3 OS=Homo sapiens GN=ATP2B3 PE=1 SV=3 | 134112 | 5.2954 | 0.052 | B | O |
| Q16795 | NADH dehydrogenase [ubiquinone] 1 alpha subcomplex subunit 9, mitochondrial OS=Homo sap | 42482 | 10.1558 | 0.116 | B | O |
| Q16798 | NADP-dependent malic enzyme, mitochondrial OS=Homo sapiens GN=ME3 PE=2 SV=2 | 67025 | 7.894 | 0.050 | B | O |
| Q16799 | Reticulon-1 OS=Homo sapiens GN=RTN1 PE=1 SV=1 | 83566 | 4.415 | 0.117 | B | O |
| Q2TB90 | Putative hexokinase HKDC1 OS=Homo sapiens GN=HKDC1 PE=1 SV=3 | 102478 | 6.7412 | 0.031 | B | O |
| Q3ZCW2 | Galectin-related protein OS=Homo sapiens GN=LGALSL PE=1 SV=2 | 18973 | 4.96 | 0.001 | B | O |
| Q4G0N4 | NAD kinase 2, mitochondrial OS=Homo sapiens GN=NADK2 PE=1 SV=2 | 49402 | 8.1211 | 0.028 | B | O |
| Q58FF6 | Putative heat shock protein HSP 90-beta 4 OS=Homo sapiens GN=HSP90AB4P PE=5 SV=1 | 58227 | 4.4531 | 0.039 | B | O |
| Q58FF8 | Putative heat shock protein HSP 90-beta 2 OS=Homo sapiens GN=HSP90AB2P PE=1 SV=2 | 44321 | 4.5894 | 0.059 | B | O |
| Q5F2F8 | Serine/threonine-protein phosphatase OS=Homo sapiens GN=PPP3CB PE=1 SV=1 | 55978 | 5.354 | 0.024 | B | O |
| Q5H9L2 | Transcription elongation factor A protein-like 5 OS=Homo sapiens GN=TCEAL5 PE=1 SV=1 | 23292 | 4.5454 | 0.105 | B | O |
| Q5HYI5 | Putative uncharacterized protein DKFZp313C1541 OS=Homo sapiens GN=DKFZp313C1541 PI | 23404 | 7.3623 | 0.079 | B | O |
| Q5JTJ3 | Cytochrome c oxidase assembly factor 6 homolog OS=Homo sapiens GN=COA6 PE=1 SV=1 | 14106 | 8.2866 | 0.060 | B | O |
| Q5T3I3 | NAD(P)H-hydrate epimerase OS=Homo sapiens GN=APOA1BP PE=1 SV=1 | 28319 | 8.1138 | 0.001 | B | O |
| Q5T655 | Cilia- and flagella-associated protein 58 OS=Homo sapiens GN=CFAP58 PE=1 SV=1 | 103353 | 8.7319 | 0.018 | B | O |
| Q5TZA2 | Rootletin OS=Homo sapiens GN=CROCC PE=1 SV=1 | 228385 | 5.2778 | 0.769 | B | O |
| Q5TZF3 | Ankyrin repeat domain-containing protein 45 OS=Homo sapiens GN=ANKRD45 PE=1 SV=1 | 31789 | 4.3945 | 0.027 | B | O |
| Q5VT06 | Centrosome-associated protein 350 OS=Homo sapiens GN=CEP350 PE=1 SV=1 | 350714 | 5.8989 | 0.506 | B | O |
| Q5XKP0 | MICOS complex subunit MIC13 OS=Homo sapiens GN=MIC13 PE=1 SV=1 | 13078 | 9.7148 | 0.086 | B | O |
| Q66GS9 | Centrosomal protein of 135 kDa OS=Homo sapiens GN=CEP135 PE=1 SV=2 | 133407 | 5.8081 | 0.356 | B | O |
| Q68D91 | Metallo-beta-lactamase domain-containing protein 2 OS=Homo sapiens GN=MBLAC2 PE=1 SV | 31351 | 6.4468 | 0.052 | B | O |
| Q6DKI1 | 60S ribosomal protein L7-like 1 OS=Homo sapiens GN=RPL7L1 PE=1 SV=1 | 28643 | 10.9644 | 0.292 | B | O |
| Q6P587 | Acylpyruvase FAHD1, mitochondrial OS=Homo sapiens GN=FAHD1 PE=1 SV=2 | 24826 | 7.1689 | 0.070 | B | O |
| Q6PCE3 | Glucose 1,6-bisphosphate synthase OS=Homo sapiens GN=PGM2L1 PE=1 SV=3 | 70396 | 6.8042 | 0.040 | B | O |
| Q6R327 | Rapamycin-insensitive companion of mTOR OS=Homo sapiens GN=RICTOR PE=1 SV=1 | 192096 | 7.1323 | 0.351 | B | O |
| Q6S8J3 | POTE ankyrin domain family member E OS=Homo sapiens GN=POTEE PE=1 SV=3 | 121285 | 5.7715 | 0.030 | B | O |
| Q709C8 | Vacuolar protein sorting-associated protein 13C OS=Homo sapiens GN=VPS13C PE=1 SV=1 | 422122 | 6.3647 | 0.026 | B | O |
| Q70EL4 | Ubiquitin carboxyl-terminal hydrolase 43 OS=Homo sapiens GN=USP43 PE=1 SV=2 | 122732 | 9.394 | 0.048 | B | O |
| Q70J99 | Protein unc-13 homolog D OS=Homo sapiens GN=UNC13D PE=1 SV=1 | 123204 | 6.1875 | 3.997 | B | O |
| Q71U36 | Tubulin alpha-1A chain OS=Homo sapiens GN=TUBA1A PE=1 SV=1 | 50103 | 4.7622 | 2.999 | B | O |
| Q7L099 | Protein RUFY3 OS=Homo sapiens GN=RUFY3 PE=1 SV=1 | 52931 | 5.2002 | 0.057 | B | O |
| Q7L0J3 | Synaptic vesicle glycoprotein 2A OS=Homo sapiens GN=SV2A PE=1 SV=1 | 82642 | 5.2354 | 0.294 | B | O |
| Q7L266 | Isoaspartyl peptidase/L-asparaginase OS=Homo sapiens GN=ASRGL1 PE=1 SV=2 | 32034 | 5.7993 | 0.064 | B | O |
| Q7L273 | BTB/POZ domain-containing protein KCTD9 OS=Homo sapiens GN=KCTD9 PE=1 SV=1 | 42539 | 5.9209 | 0.354 | B | O |
| Q7RTS7 | Keratin, type II cytoskeletal 74 OS=Homo sapiens GN=KRT74 PE=1 SV=2 | 57829 | 7.6113 | 0.085 | B | O |
| Q7Z2Z1 | Treslin OS=Homo sapiens GN=TICRR PE=1 SV=2 | 210723 | 8.8828 | 0.205 | B | O |
| Q86TJ2 | Transcriptional adapter 2-beta OS=Homo sapiens GN=TADA2B PE=1 SV=2 | 48439 | 7.7402 | 0.415 | B | O |
| Q86UY8 | 5'-nucleotidase domain-containing protein 3 OS=Homo sapiens GN=NT5DC3 PE=1 SV=1 | 63379 | 8.3628 | 0.047 | B | O |
| Q86VP6 | Cullin-associated NEDD8-dissociated protein 1 OS=Homo sapiens GN=CAND1 PE=1 SV=2 | 136288 | 5.4067 | 0.192 | B | O |
| Q86XW9 | Thioredoxin domain-containing protein 6 OS=Homo sapiens GN=NME9 PE=2 SV=1 | 36832 | 4.623 | 1.538 | B | O |
| Q86Y46 | Keratin, type II cytoskeletal 73 OS=Homo sapiens GN=KRT73 PE=1 SV=1 | 58886 | 6.9932 | 0.051 | B | O |
| Q8IV08 | Phospholipase D3 OS=Homo sapiens GN=PLD3 PE=1 SV=1 | 54670 | 6.0073 | 0.158 | B | O |
| Q8IWA5 | Choline transporter-like protein 2 OS=Homo sapiens GN=SLC44A2 PE=1 SV=3 | 80070 | 8.521 | 0.046 | B | O |
| Q8IWJ2 | GRIP and coiled-coil domain-containing protein 2 OS=Homo sapiens GN=GCC2 PE=1 SV=4 | 195787 | 4.916 | 0.509 | B | O |

| | | | | | | |
|---|---|---|---|---|---|---|
| Q8N126 | Cell adhesion molecule 3 OS=Homo sapiens GN=CADM3 PE=1 SV=1 | 43272 | 5.6572 | 0.244 | B | O |
| Q8N3J6 | Cell adhesion molecule 2 OS=Homo sapiens GN=CADM2 PE=2 SV=1 | 47524 | 4.9922 | 0.263 | B | O |
| Q8N6N7 | Acyl-CoA-binding domain-containing protein 7 OS=Homo sapiens GN=ACBD7 PE=1 SV=1 | 9784 | 6.7588 | 0.201 | B | O |
| Q8N7X1 | RNA-binding motif protein, X-linked-like-3 OS=Homo sapiens GN=RBMXL3 PE=2 SV=2 | 114868 | 9.1538 | 0.176 | B | O |
| Q8N998 | Coiled-coil domain-containing protein 89 OS=Homo sapiens GN=CCDC89 PE=2 SV=1 | 43781 | 5.1533 | 0.150 | B | O |
| Q8NCM8 | Cytoplasmic dynein 2 heavy chain 1 OS=Homo sapiens GN=DYNC2H1 PE=1 SV=4 | 492311 | 6.0908 | 0.266 | B | O |
| Q8NE28 | Serine/threonine kinase-like domain-containing protein STKLD1 OS=Homo sapiens GN=STKLD | 75627 | 5.0742 | 1.423 | B | O |
| Q8NFZ8 | Cell adhesion molecule 4 OS=Homo sapiens GN=CADM4 PE=1 SV=1 | 42758 | 5.8755 | 0.270 | B | O |
| Q8NI77 | Kinesin-like protein KIF18A OS=Homo sapiens GN=KIF18A PE=1 SV=2 | 102216 | 9.1245 | 17.572 | B | O |
| Q8TAA3 | Proteasome subunit alpha type-7-like OS=Homo sapiens GN=PSMA8 PE=2 SV=3 | 28512 | 9.3354 | 0.104 | B | O |
| Q8TB36 | Ganglioside-induced differentiation-associated protein 1 OS=Homo sapiens GN=GDAP1 PE=1 S | 41319 | 8.4858 | 0.115 | B | O |
| Q8TC05 | Nuclear protein MDM1 OS=Homo sapiens GN=MDM1 PE=1 SV=2 | 80686 | 9.4878 | 3.311 | B | O |
| Q8WWL7 | G2/mitotic-specific cyclin-B3 OS=Homo sapiens GN=CCNB3 PE=1 SV=2 | 157815 | 6.2534 | 0.130 | B | O |
| Q8WXF1 | Paraspeckle component 1 OS=Homo sapiens GN=PSPC1 PE=1 SV=1 | 58706 | 6.2344 | 0.213 | B | O |
| Q8WXX0 | Dynein heavy chain 7, axonemal OS=Homo sapiens GN=DNAH7 PE=1 SV=2 | 460861 | 5.6074 | 0.085 | B | O |
| Q8WY54 | Protein phosphatase 1E OS=Homo sapiens GN=PPM1E PE=1 SV=2 | 84948 | 4.7666 | 0.075 | B | O |
| Q92599 | Septin-8 OS=Homo sapiens GN=SEPT8 PE=1 SV=4 | 55721 | 5.8418 | 0.086 | B | O |
| Q92752 | Tenascin-R OS=Homo sapiens GN=TNR PE=1 SV=3 | 149467 | 4.522 | 0.609 | B | O |
| Q92841 | Probable ATP-dependent RNA helicase DDX17 OS=Homo sapiens GN=DDX17 PE=1 SV=2 | 80222 | 8.2397 | 0.012 | B | O |
| Q93050 | V-type proton ATPase 116 kDa subunit a isoform 1 OS=Homo sapiens GN=ATP6V0A1 PE=1 S | 96350 | 5.981 | 0.068 | B | O |
| Q969P0 | Immunoglobulin superfamily member 8 OS=Homo sapiens GN=IGSF8 PE=1 SV=1 | 64993 | 7.8999 | 0.140 | B | O |
| Q96A08 | Histone H2B type 1-A OS=Homo sapiens GN=HIST1H2BA PE=1 SV=3 | 14158 | 10.7402 | 1.804 | B | O |
| Q96CX2 | BTB/POZ domain-containing protein KCTD12 OS=Homo sapiens GN=KCTD12 PE=1 SV=1 | 35678 | 5.3232 | 0.115 | B | O |
| Q96E17 | Ras-related protein Rab-3C OS=Homo sapiens GN=RAB3C PE=2 SV=1 | 25935 | 4.9028 | 0.728 | B | O |
| Q96F85 | CB1 cannabinoid receptor-interacting protein 1 OS=Homo sapiens GN=CNRIP1 PE=1 SV=1 | 18636 | 8.1768 | 0.322 | B | O |
| Q96GW7 | Brevican core protein OS=Homo sapiens GN=BCAN PE=1 SV=2 | 99056 | 4.377 | 0.275 | B | O |
| Q96IX5 | Up-regulated during skeletal muscle growth protein 5 OS=Homo sapiens GN=USMG5 PE=1 SV | 6453 | 10.0664 | 0.349 | B | O |
| Q96IZ5 | RNA-binding protein 41 OS=Homo sapiens GN=RBM41 PE=1 SV=2 | 47070 | 8.9912 | 2.094 | B | O |
| Q96JE9 | Microtubule-associated protein 6 OS=Homo sapiens GN=MAP6 PE=1 SV=2 | 86451 | 9.5786 | 0.130 | B | O |
| Q96KP4 | Cytosolic non-specific dipeptidase OS=Homo sapiens GN=CNDP2 PE=1 SV=2 | 52844 | 5.5679 | 0.317 | B | O |
| Q96L91 | E1A-binding protein p400 OS=Homo sapiens GN=EP400 PE=1 SV=4 | 343275 | 9.4834 | 0.006 | B | O |
| Q96PK6 | RNA-binding protein 14 OS=Homo sapiens GN=RBM14 PE=1 SV=2 | 69448 | 9.8042 | 0.268 | B | O |
| Q96QB1 | Rho GTPase-activating protein 7 OS=Homo sapiens GN=DLC1 PE=1 SV=4 | 170484 | 5.9458 | 0.587 | B | O |
| Q96QF0 | Rab-3A-interacting protein OS=Homo sapiens GN=RAB3IP PE=1 SV=1 | 52987 | 5.6235 | 0.026 | B | O |
| Q96QK1 | Vacuolar protein sorting-associated protein 35 OS=Homo sapiens GN=VPS35 PE=1 SV=2 | 91649 | 5.168 | 0.048 | B | O |
| Q99497 | Protein deglycase DJ-1 OS=Homo sapiens GN=PARK7 PE=1 SV=2 | 19878 | 6.3721 | 0.902 | B | O |
| Q99536 | Synaptic vesicle membrane protein VAT-1 homolog OS=Homo sapiens GN=VAT1 PE=1 SV=2 | 41893 | 5.8506 | 0.119 | B | O |
| Q99584 | Protein S100-A13 OS=Homo sapiens GN=S100A13 PE=1 SV=1 | 11464 | 5.8257 | 0.440 | B | O |
| Q99661 | Kinesin-like protein KIF2C OS=Homo sapiens GN=KIF2C PE=1 SV=2 | 81261 | 7.7153 | 0.269 | B | O |
| Q99714 | 3-hydroxyacyl-CoA dehydrogenase type-2 OS=Homo sapiens GN=HSD17B10 PE=1 SV=3 | 26906 | 7.8384 | 0.113 | B | O |
| Q99747 | Gamma-soluble NSF attachment protein OS=Homo sapiens GN=NAPG PE=1 SV=1 | 34724 | 5.1299 | 0.063 | B | O |
| Q99798 | Aconitate hydratase, mitochondrial OS=Homo sapiens GN=ACO2 PE=1 SV=2 | 85371 | 7.3286 | 0.697 | B | O |
| Q99832 | T-complex protein 1 subunit eta OS=Homo sapiens GN=CCT7 PE=1 SV=2 | 59328 | 7.519 | 0.112 | B | O |
| Q99962 | Endophilin-A1 OS=Homo sapiens GN=SH3GL2 PE=1 SV=1 | 39937 | 5.1636 | 0.287 | B | O |
| Q9BPW8 | Protein NipSnap homolog 1 OS=Homo sapiens GN=NIPSNAP1 PE=1 SV=1 | 33288 | 9.5581 | 0.142 | B | O |
| Q9BRQ6 | MICOS complex subunit MIC25 OS=Homo sapiens GN=CHCHD6 PE=1 SV=1 | 26441 | 9.1494 | 0.020 | B | O |
| Q9BS92 | Protein NipSnap homolog 3B OS=Homo sapiens GN=NIPSNAP3B PE=2 SV=1 | 28295 | 9.5698 | 0.015 | B | O |
| Q9BUF5 | Tubulin beta-6 chain OS=Homo sapiens GN=TUBB6 PE=1 SV=1 | 49825 | 4.5791 | 0.475 | B | O |
| Q9BUJ2 | Heterogeneous nuclear ribonucleoprotein U-like protein 1 OS=Homo sapiens GN=HNRNPUL1 F | 95679 | 6.4878 | 1.975 | B | O |
| Q9BVA1 | Tubulin beta-2B chain OS=Homo sapiens GN=TUBB2B PE=1 SV=1 | 49920 | 4.5908 | 0.786 | B | O |
| Q9BW30 | Tubulin polymerization-promoting protein family member 3 OS=Homo sapiens GN=TPPP3 PE= | 18973 | 9.5288 | 0.294 | B | O |
| Q9BX68 | Histidine triad nucleotide-binding protein 2, mitochondrial OS=Homo sapiens GN=HINT2 PE=1 S | 17151 | 9.5874 | 0.090 | B | O |
| Q9GZP4 | PITH domain-containing protein 1 OS=Homo sapiens GN=PITHD1 PE=1 SV=1 | 24162 | 5.376 | 0.051 | B | O |
| Q9H0C2 | ADP/ATP translocase 4 OS=Homo sapiens GN=SLC25A31 PE=2 SV=1 | 34999 | 10.2378 | 0.036 | B | O |
| Q9H0E2 | Toll-interacting protein OS=Homo sapiens GN=TOLLIP PE=1 SV=1 | 30262 | 5.584 | 0.018 | B | O |
| Q9H0N0 | Ras-related protein Rab-6C OS=Homo sapiens GN=RAB6C PE=1 SV=2 | 28337 | 7.8032 | 0.093 | B | O |
| Q9H0U4 | Ras-related protein Rab-1B OS=Homo sapiens GN=RAB1B PE=1 SV=1 | 22157 | 5.395 | 0.670 | B | O |
| Q9H115 | Beta-soluble NSF attachment protein OS=Homo sapiens GN=NAPB PE=1 SV=2 | 33535 | 5.1694 | 0.221 | B | O |
| Q9H254 | Spectrin beta chain, non-erythrocytic 4 OS=Homo sapiens GN=SPTBN4 PE=1 SV=2 | 288806 | 5.6323 | 0.241 | B | O |
| Q9H3Z4 | DnaJ homolog subfamily C member 5 OS=Homo sapiens GN=DNAJC5 PE=1 SV=1 | 22134 | 4.7446 | 0.167 | B | O |
| Q9H4B7 | Tubulin beta-1 chain OS=Homo sapiens GN=TUBB1 PE=1 SV=1 | 50294 | 4.8823 | 0.113 | B | O |
| Q9H6S3 | Epidermal growth factor receptor kinase substrate 8-like protein 2 OS=Homo sapiens GN=EPS8 | 80570 | 6.3838 | 0.084 | B | O |
| Q9H8M7 | Protein FAM188A OS=Homo sapiens GN=FAM188A PE=1 SV=1 | 49692 | 4.4927 | 0.556 | B | O |
| Q9H9B4 | Sideroflexin-1 OS=Homo sapiens GN=SFXN1 PE=1 SV=4 | 35596 | 9.3457 | 0.220 | B | O |
| Q9HB66 | Alternative protein MKKS OS=Homo sapiens GN=MKKS PE=1 SV=1 | 7258 | 10.0313 | 0.030 | B | O |
| Q9HB71 | Calcyclin-binding protein OS=Homo sapiens GN=CACYBP PE=1 SV=2 | 26193 | 8.5825 | 0.084 | B | O |
| Q9HCH3 | Copine-5 OS=Homo sapiens GN=CPNE5 PE=1 SV=2 | 65691 | 5.562 | 0.037 | B | O |
| Q9HCK8 | Chromodomain-helicase-DNA-binding protein 8 OS=Homo sapiens GN=CHD8 PE=1 SV=5 | 290335 | 6.0132 | 0.031 | B | O |
| Q9NP72 | Ras-related protein Rab-18 OS=Homo sapiens GN=RAB18 PE=1 SV=1 | 22962 | 4.9263 | 0.086 | B | O |
| Q9NP81 | Serine--tRNA ligase, mitochondrial OS=Homo sapiens GN=SARS2 PE=1 SV=1 | 58245 | 8.0786 | 0.178 | B | O |
| Q9NQ39 | Putative 40S ribosomal protein S10-like OS=Homo sapiens GN=RPS10P5 PE=5 SV=1 | 20107 | 10.5117 | 0.257 | B | O |
| Q9NQC3 | Reticulon-4 OS=Homo sapiens GN=RTN4 PE=1 SV=2 | 129851 | 4.2246 | 0.162 | B | O |
| Q9NQR4 | Omega-amidase NIT2 OS=Homo sapiens GN=NIT2 PE=1 SV=1 | 30588 | 6.9858 | 0.021 | B | O |
| Q9NRC6 | Spectrin beta chain, non-erythrocytic 5 OS=Homo sapiens GN=SPTBN5 PE=1 SV=2 | 416491 | 6.1904 | 0.624 | B | O |
| Q9NRW1 | Ras-related protein Rab-6B OS=Homo sapiens GN=RAB6B PE=1 SV=1 | 23446 | 5.2163 | 0.104 | B | O |
| Q9NSD9 | Phenylalanine--tRNA ligase beta subunit OS=Homo sapiens GN=FARSB PE=1 SV=3 | 66073 | 6.3955 | 0.146 | B | O |
| Q9NUJ1 | Mycophenolic acid acyl-glucuronide esterase, mitochondrial OS=Homo sapiens GN=ABHD10 P | 33910 | 8.7056 | 0.127 | B | O |
| Q9NVJ2 | ADP-ribosylation factor-like protein 8B OS=Homo sapiens GN=ARL8B PE=1 SV=1 | 21525 | 8.6045 | 0.103 | B | O |
| Q9NY65 | Tubulin alpha-8 chain OS=Homo sapiens GN=TUBA8 PE=1 SV=1 | 50061 | 4.7578 | 0.061 | B | O |
| Q9NYI0 | PH and SEC7 domain-containing protein 3 OS=Homo sapiens GN=PSD3 PE=1 SV=2 | 115961 | 5.5986 | 0.251 | B | O |
| Q9NZ45 | CDGSH iron-sulfur domain-containing protein 1 OS=Homo sapiens GN=CISD1 PE=1 SV=1 | 12191 | 9.4468 | 0.286 | B | O |

| | | | | | | |
|---|---|---|---|---|---|---|
| Q9NZL9 | Methionine adenosyltransferase 2 subunit beta OS=Homo sapiens GN=MAT2B PE=1 SV=1 | 37528 | 6.9858 | 0.034 | B | O |
| Q9P035 | Very-long-chain (3R)-3-hydroxyacyl-CoA dehydratase 3 OS=Homo sapiens GN=HACD3 PE=1 S | 43131 | 9.1714 | 0.058 | B | O |
| Q9P0J0 | NADH dehydrogenase [ubiquinone] 1 alpha subcomplex subunit 13 OS=Homo sapiens GN=ND | 16687 | 9.0513 | 0.254 | B | O |
| Q9P121 | Neurotrimin OS=Homo sapiens GN=NTM PE=1 SV=1 | 37947 | 7.7314 | 0.139 | B | O |
| Q9P2D1 | Chromodomain-helicase-DNA-binding protein 7 OS=Homo sapiens GN=CHD7 PE=1 SV=3 | 335715 | 5.9033 | 0.085 | B | O |
| Q9P2R7 | Succinyl-CoA ligase [ADP-forming] subunit beta, mitochondrial OS=Homo sapiens GN=SUCLA2 | 50285 | 7.2041 | 0.427 | B | O |
| Q9UBB6 | Neurochondrin OS=Homo sapiens GN=NCDN PE=1 SV=1 | 78813 | 5.1812 | 0.114 | B | O |
| Q9UBI6 | Guanine nucleotide-binding protein G(I)/G(S)/G(O) subunit gamma-12 OS=Homo sapiens GN=( | 8001 | 9.4761 | 0.060 | B | O |
| Q9UBQ7 | Glyoxylate reductase/hydroxypyruvate reductase OS=Homo sapiens GN=GRHPR PE=1 SV=1 | 35645 | 7.1411 | 0.035 | B | O |
| Q9UDW1 | Cytochrome b-c1 complex subunit 9 OS=Homo sapiens GN=UQCR10 PE=1 SV=3 | 7303 | 9.8643 | 0.165 | B | O |
| Q9UFM8 | Neuroplastin (Fragment) OS=Homo sapiens GN=DKFZp566H1924 PE=1 SV=2 | 34793 | 7.6582 | 0.124 | B | O |
| Q9UFN0 | Protein NipSnap homolog 3A OS=Homo sapiens GN=NIPSNAP3A PE=1 SV=2 | 28448 | 9.4556 | 0.127 | B | O |
| Q9UH03 | Neuronal-specific septin-3 OS=Homo sapiens GN=SEPT3 PE=1 SV=3 | 40678 | 6.8218 | 0.073 | B | O |
| Q9UHD8 | Septin-9 OS=Homo sapiens GN=SEPT9 PE=1 SV=2 | 65360 | 9.312 | 0.049 | B | O |
| Q9UHD9 | Ubiquilin-2 OS=Homo sapiens GN=UBQLN2 PE=1 SV=2 | 65654 | 4.9717 | 0.025 | B | O |
| Q9UHG2 | ProSAAS OS=Homo sapiens GN=PCSK1N PE=1 SV=1 | 27355 | 6.2227 | 0.164 | B | O |
| Q9UI15 | Transgelin-3 OS=Homo sapiens GN=TAGLN3 PE=1 SV=2 | 22458 | 7.1704 | 0.093 | B | O |
| Q9UIJ7 | GTP:AMP phosphotransferase AK3, mitochondrial OS=Homo sapiens GN=AK3 PE=1 SV=4 | 25549 | 9.5303 | 0.215 | B | O |
| Q9UJS0 | Calcium-binding mitochondrial carrier protein Aralar2 OS=Homo sapiens GN=SLC25A13 PE=1 | 74128 | 8.77 | 12.790 | B | O |
| Q9UJZ1 | Stomatin-like protein 2, mitochondrial OS=Homo sapiens GN=STOML2 PE=1 SV=1 | 38510 | 7.2642 | 0.541 | B | O |
| Q9UK22 | F-box only protein 2 OS=Homo sapiens GN=FBXO2 PE=1 SV=2 | 33306 | 4.0957 | 0.125 | B | O |
| Q9UL25 | Ras-related protein Rab-21 OS=Homo sapiens GN=RAB21 PE=1 SV=3 | 24332 | 7.916 | 0.076 | B | O |
| Q9UM22 | Mammalian ependymin-related protein 1 OS=Homo sapiens GN=EPDR1 PE=1 SV=2 | 25420 | 6.4189 | 0.073 | B | O |
| Q9UMX0 | Ubiquilin-1 OS=Homo sapiens GN=UBQLN1 PE=1 SV=2 | 62479 | 4.8413 | 0.074 | B | O |
| Q9UMX5 | Neudesin OS=Homo sapiens GN=NENF PE=1 SV=1 | 18844 | 5.3613 | 0.076 | B | O |
| Q9UN86 | Ras GTPase-activating protein-binding protein 2 OS=Homo sapiens GN=G3BP2 PE=1 SV=2 | 54087 | 5.2632 | 0.116 | B | O |
| Q9UNW9 | RNA-binding protein Nova-2 OS=Homo sapiens GN=NOVA2 PE=1 SV=1 | 48978 | 8.3511 | 0.091 | B | O |
| Q9UNZ2 | NSFL1 cofactor p47 OS=Homo sapiens GN=NSFL1C PE=1 SV=2 | 40548 | 4.8076 | 0.138 | B | O |
| Q9UPV7 | PHD finger protein 24 OS=Homo sapiens GN=PHF24 PE=1 SV=2 | 45163 | 5.3496 | 0.014 | B | O |
| Q9UPX8 | SH3 and multiple ankyrin repeat domains protein 2 OS=Homo sapiens GN=SHANK2 PE=1 SV= | 158722 | 6.4658 | 0.060 | B | O |
| Q9UPY8 | Microtubule-associated protein RP/EB family member 3 OS=Homo sapiens GN=MAPRE3 PE=1 | 31961 | 5.1958 | 0.083 | B | O |
| Q9UQ16 | Dynamin-3 OS=Homo sapiens GN=DNM3 PE=1 SV=4 | 97685 | 8.458 | 0.406 | B | O |
| Q9UQ35 | Serine/arginine repetitive matrix protein 2 OS=Homo sapiens GN=SRRM2 PE=1 SV=2 | 299434 | 12.4541 | 0.054 | B | O |
| Q9UQ80 | Proliferation-associated protein 2G4 OS=Homo sapiens GN=PA2G4 PE=1 SV=3 | 43759 | 6.1069 | 0.040 | B | O |
| Q9UQM7 | Calcium/calmodulin-dependent protein kinase type II subunit alpha OS=Homo sapiens GN=CAM | 54053 | 6.6284 | 0.449 | B | O |
| Q9Y230 | RuvB-like 2 OS=Homo sapiens GN=RUVBL2 PE=1 SV=3 | 51124 | 5.335 | 0.040 | B | O |
| Q9Y266 | Nuclear migration protein nudC OS=Homo sapiens GN=NUDC PE=1 SV=1 | 38219 | 5.0947 | 0.085 | B | O |
| Q9Y277 | Voltage-dependent anion-selective channel protein 3 OS=Homo sapiens GN=VDAC3 PE=1 SV= | 30639 | 8.8301 | 1.096 | B | O |
| Q9Y281 | Cofilin-2 OS=Homo sapiens GN=CFL2 PE=1 SV=1 | 18724 | 8.1592 | 0.582 | B | O |
| Q9Y2J8 | Protein-arginine deiminase type-2 OS=Homo sapiens GN=PADI2 PE=1 SV=2 | 75515 | 5.2588 | 0.415 | B | O |
| Q9Y2T3 | Guanine deaminase OS=Homo sapiens GN=GDA PE=1 SV=1 | 50970 | 5.3262 | 0.151 | B | O |
| Q9Y333 | U6 snRNA-associated Sm-like protein LSm2 OS=Homo sapiens GN=LSM2 PE=1 SV=1 | 10827 | 6.082 | 0.091 | B | O |
| Q9Y3B3 | Transmembrane emp24 domain-containing protein 7 OS=Homo sapiens GN=TMED7 PE=1 SV= | 25155 | 6.4893 | 0.076 | B | O |
| Q9Y3F4 | Serine-threonine kinase receptor-associated protein OS=Homo sapiens GN=STRAP PE=1 SV= | 38413 | 4.8032 | 0.083 | B | O |
| Q9Y3I0 | tRNA-splicing ligase RtcB homolog OS=Homo sapiens GN=RTCB PE=1 SV=1 | 55174 | 6.8071 | 0.033 | B | O |
| Q9Y4D7 | Plexin-D1 OS=Homo sapiens GN=PLXND1 PE=1 SV=3 | 211870 | 6.75 | 0.024 | B | O |
| Q9Y5K8 | V-type proton ATPase subunit D OS=Homo sapiens GN=ATP6V1D PE=1 SV=1 | 28245 | 9.7983 | 0.337 | B | O |
| Q9Y5Z4 | Heme-binding protein 2 OS=Homo sapiens GN=HEBP2 PE=1 SV=1 | 22861 | 4.3491 | 0.081 | B | O |
| Q9Y617 | Phosphoserine aminotransferase OS=Homo sapiens GN=PSAT1 PE=1 SV=2 | 40396 | 7.5879 | 0.158 | B | O |
| Q9Y639 | Neuroplastin OS=Homo sapiens GN=NPTN PE=1 SV=2 | 44359 | 7.9834 | 0.176 | B | O |
| Q9Y6C9 | Mitochondrial carrier homolog 2 OS=Homo sapiens GN=MTCH2 PE=1 SV=1 | 33308 | 7.8926 | 0.044 | B | O |
| Q9Y6K8 | Adenylate kinase isoenzyme 5 OS=Homo sapiens GN=AK5 PE=1 SV=2 | 63293 | 4.7695 | 0.039 | B | O |
| V9GYG0 | ADP/ATP translocase 1 OS=Homo sapiens GN=SLC25A4 PE=1 SV=1 | 22871 | 9.8496 | 0.083 | B | O |
| X6RFL8 | Ras-related protein Rab-14 (Fragment) OS=Homo sapiens GN=RAB14 PE=1 SV=1 | 20396 | 5.9121 | 0.268 | B | O |
| A0A024QZX5 | Serpin B6 OS=Homo sapiens GN=SERPINB6 PE=1 SV=1 | 42996 | 5.0039 | 0.001 | D | O |
| A0A024R3B9 | Alpha-crystallin B chain OS=Homo sapiens GN=CRYAB PE=1 SV=1 | 12245 | 9.2739 | 0.001 | D | O |
| A0A024RA52 | Proteasome subunit alpha type OS=Homo sapiens GN=PSMA2 PE=1 SV=1 | 25882 | 7.2979 | 0.001 | D | O |
| A0A075B6H6 | Ig kappa chain C region (Fragment) OS=Homo sapiens GN=IGKC PE=1 SV=1 | 11712 | 5.5005 | 0.001 | D | O |
| A0A075B6K8 | Ig lambda-1 chain C regions (Fragment) OS=Homo sapiens GN=IGLC1 PE=4 SV=1 | 11394 | 7.9878 | 0.001 | D | O |
| A0A075B716 | 40S ribosomal protein S17 OS=Homo sapiens GN=RPS17 PE=1 SV=1 | 21629 | 9.1802 | 0.001 | D | O |
| A0A075B762 | Neuroblastoma breakpoint family member 10 OS=Homo sapiens GN=NBPF10 PE=4 SV=3 | 435361 | 4.3843 | 0.177 | D | O |
| A0A087WT59 | Transthyretin OS=Homo sapiens GN=TTR PE=1 SV=1 | 20132 | 5.0376 | 0.001 | D | O |
| A0A087WT87 | Amino acid transporter OS=Homo sapiens GN=SLC1A3 PE=1 SV=1 | 54172 | 7.2817 | 0.657 | D | O |
| A0A087WT95 | Acyl-coenzyme A thioesterase 2, mitochondrial OS=Homo sapiens GN=ACOT2 PE=1 SV=1 | 50897 | 8.1504 | 0.001 | D | O |
| A0A087WTF6 | Neural cell adhesion molecule 1 OS=Homo sapiens GN=NCAM1 PE=1 SV=1 | 93271 | 4.585 | 1.262 | D | O |
| A0A087WTH0 | Enolase-phosphatase E1 OS=Homo sapiens GN=ENOPH1 PE=1 SV=2 | 23349 | 4.7783 | 0.001 | D | O |
| A0A087WTM7 | Apolipoprotein B-100 OS=Homo sapiens GN=APOB PE=1 SV=1 | 489527 | 6.6899 | 0.057 | D | O |
| A0A087WTP3 | Far upstream element-binding protein 2 OS=Homo sapiens GN=KHSRP PE=1 SV=1 | 72982 | 7.5762 | 0.001 | D | O |
| A0A087WTT1 | Polyadenylate-binding protein OS=Homo sapiens GN=PABPC1 PE=1 SV=1 | 58498 | 9.5977 | 0.141 | D | O |
| A0A087WUI2 | Heterogeneous nuclear ribonucleoproteins A2/B1 OS=Homo sapiens GN=HNRNPA2B1 PE=1 S | 29816 | 4.7183 | 0.450 | D | O |
| A0A087WUK2 | Heterogeneous nuclear ribonucleoprotein D-like OS=Homo sapiens GN=HNRNPDL PE=1 SV=1 | 40015 | 10.355 | 0.108 | D | O |
| A0A087WUL2 | Proteasome subunit beta type-3 (Fragment) OS=Homo sapiens GN=PSMB3 PE=1 SV=1 | 16150 | 8.5708 | 0.001 | D | O |
| A0A087WUS0 | 40S ribosomal protein S24 OS=Homo sapiens GN=RPS24 PE=1 SV=1 | 15343 | 11.458 | 0.001 | D | O |
| A0A087WUZ3 | Spectrin beta chain, non-erythrocytic 1 OS=Homo sapiens GN=SPTBN1 PE=1 SV=1 | 274657 | 5.2515 | 0.001 | D | O |
| A0A087WV23 | SH3 domain-binding glutamic acid-rich-like protein 3 OS=Homo sapiens GN=SH3BGRL3 PE=1 | 23771 | 9.3442 | 0.001 | D | O |
| A0A087WV47 | Ig gamma-1 chain C region OS=Homo sapiens GN=IGHG1 PE=1 SV=1 | 51121 | 7.3813 | 0.001 | D | O |
| A0A087WVQ6 | Clathrin heavy chain OS=Homo sapiens GN=CLTC PE=1 SV=1 | 191934 | 5.3555 | 0.001 | D | O |
| A0A087WVQ9 | Elongation factor 1-alpha 1 OS=Homo sapiens GN=EEF1A1 PE=1 SV=1 | 47852 | 9.3691 | 0.001 | D | O |
| A0A087WW66 | 26S proteasome non-ATPase regulatory subunit 1 OS=Homo sapiens GN=PSMD1 PE=1 SV=1 | 105783 | 5.1123 | 0.001 | D | O |
| A0A087WW96 | Synapsin-2 OS=Homo sapiens GN=SYN2 PE=1 SV=1 | 62956 | 8.5474 | 0.712 | D | O |
| A0A087WWB6 | Transgelin OS=Homo sapiens GN=TAGLN3 PE=1 SV=1 | 24994 | 8.5649 | 0.001 | D | O |

| | | | | | | |
|---|---|---|---|---|---|---|
| A0A087WWU8 | Tropomyosin alpha-3 chain OS=Homo sapiens GN=TPM3 PE=1 SV=1 | 26404 | 4.5513 | 0.001 | D | O |
| A0A087WX29 | TAR DNA-binding protein 43 (Fragment) OS=Homo sapiens GN=TARDBP PE=1 SV=1 | 26726 | 7.7402 | 0.051 | D | O |
| A0A087WXC5 | NADH dehydrogenase [ubiquinone] 1 alpha subcomplex subunit 10, mitochondrial OS=Homo sa | 40811 | 8.5957 | 0.001 | D | O |
| A0A087WXF0 | Methyl-CpG-binding protein 2 OS=Homo sapiens GN=MECP2 PE=1 SV=1 | 54796 | 10.4224 | 0.001 | D | O |
| A0A087WXM6 | 60S ribosomal protein L17 (Fragment) OS=Homo sapiens GN=RPL17 PE=3 SV=1 | 19573 | 10.4443 | 0.099 | D | O |
| A0A087WXS7 | ATPase ASNA1 OS=Homo sapiens GN=ASNA1 PE=1 SV=1 | 37094 | 4.8369 | 0.066 | D | O |
| A0A087WY00 | Unconventional myosin-Va OS=Homo sapiens GN=MYO5A PE=1 SV=1 | 212065 | 8.7144 | 0.078 | D | O |
| A0A087WY71 | AP-2 complex subunit mu OS=Homo sapiens GN=AP2M1 PE=1 SV=1 | 49495 | 9.8848 | 0.350 | D | O |
| A0A087WYT3 | Prostaglandin E synthase 3 OS=Homo sapiens GN=PTGES3 PE=1 SV=1 | 19142 | 4.147 | 0.001 | D | O |
| A0A087WZ11 | Putative ATP-dependent RNA helicase DHX57 OS=Homo sapiens GN=DHX57 PE=1 SV=1 | 54048 | 4.9072 | 1.472 | D | O |
| A0A087WZ27 | Zinc finger protein 90 OS=Homo sapiens GN=ZNF90 PE=4 SV=2 | 14385 | 10.5996 | 0.001 | D | O |
| A0A087WZH7 | Myristoylated alanine-rich C-kinase substrate OS=Homo sapiens GN=MARCKS PE=1 SV=1 | 31577 | 4.5264 | 0.443 | D | O |
| A0A087WZN1 | Isocitrate dehydrogenase [NAD] subunit, mitochondrial OS=Homo sapiens GN=IDH3B PE=1 SV | 42383 | 8.5854 | 0.001 | D | O |
| A0A087WZZ5 | Splicing factor 3B subunit 2 OS=Homo sapiens GN=SF3B2 PE=1 SV=1 | 97524 | 5.3818 | 0.582 | D | O |
| A0A087X0P0 | Kinesin-like protein OS=Homo sapiens GN=CENPE PE=1 SV=1 | 311911 | 5.3101 | 0.309 | D | O |
| A0A087X0X3 | Heterogeneous nuclear ribonucleoprotein M OS=Homo sapiens GN=HNRNPM PE=1 SV=1 | 77518 | 9.1436 | 0.001 | D | O |
| A0A087X1B9 | Ferritin OS=Homo sapiens GN=FTL PE=1 SV=1 | 21169 | 5.6396 | 0.001 | D | O |
| A0A087X1H6 | Hsc70-interacting protein OS=Homo sapiens GN=ST13 PE=1 SV=1 | 28220 | 9.0073 | 0.001 | D | O |
| A0A087X2B1 | RNA binding protein fox-1 homolog OS=Homo sapiens GN=RBFOX1 PE=4 SV=1 | 40284 | 6.624 | 0.040 | D | O |
| A0A087X2E9 | Glutathione S-transferase P (Fragment) OS=Homo sapiens GN=GSTP1 PE=1 SV=1 | 8891 | 7.1646 | 0.029 | D | O |
| A0A087X2G1 | ATP-dependent RNA helicase DDX1 OS=Homo sapiens GN=DDX1 PE=1 SV=1 | 73928 | 7.5747 | 0.001 | D | O |
| A0A087X2H1 | E3 ubiquitin-protein ligase HECTD1 OS=Homo sapiens GN=HECTD1 PE=1 SV=1 | 289449 | 5.1064 | 0.001 | D | O |
| A0A096LNH5 | Protein LOC102724023 OS=Homo sapiens GN=LOC102724023 PE=4 SV=1 | 19404 | 7.7798 | 0.001 | D | O |
| A0A096LP52 | Syntaxin-binding protein 1 (Fragment) OS=Homo sapiens GN=STXBP1 PE=1 SV=1 | 13558 | 6.6167 | 0.046 | D | O |
| A0A0A0MR85 | Glutathione S-transferase Mu 4 OS=Homo sapiens GN=GSTM4 PE=1 SV=1 | 25546 | 5.502 | 0.015 | D | O |
| A0A0A0MRA8 | Band 4.1-like protein 3 OS=Homo sapiens GN=EPB41L3 PE=1 SV=1 | 102225 | 5.0361 | 0.253 | D | O |
| A0A0A0MRD9 | Haptoglobin-related protein OS=Homo sapiens GN=HPR PE=1 SV=1 | 31478 | 7.9219 | 0.014 | D | O |
| A0A0A0MRJ6 | Protein-L-isoaspartate O-methyltransferase OS=Homo sapiens GN=PCMT1 PE=1 SV=1 | 30295 | 7.314 | 0.001 | D | O |
| A0A0A0MRX1 | ELAV-like protein OS=Homo sapiens GN=ELAVL2 PE=1 SV=1 | 42605 | 9.2974 | 0.001 | D | O |
| A0A0A0MS41 | Sideroflexin OS=Homo sapiens GN=SFXN3 PE=1 SV=1 | 35480 | 9.3223 | 0.314 | D | O |
| A0A0A0MS51 | Gelsolin OS=Homo sapiens GN=GSN PE=1 SV=1 | 82474 | 5.2896 | 0.642 | D | O |
| A0A0A0MS54 | cAMP-dependent protein kinase catalytic subunit beta OS=Homo sapiens GN=PRKACB PE=1 S | 41282 | 8.8125 | 0.001 | D | O |
| A0A0A0MS87 | Protein NDRG2 OS=Homo sapiens GN=NDRG2 PE=1 SV=1 | 39519 | 6.5845 | 0.534 | D | O |
| A0A0A0MSE2 | Hydroxyacyl-coenzyme A dehydrogenase, mitochondrial OS=Homo sapiens GN=HADH PE=1 S | 42096 | 9.6475 | 0.001 | D | O |
| A0A0A0MSI0 | Peroxiredoxin-1 (Fragment) OS=Homo sapiens GN=PRDX1 PE=1 SV=1 | 18963 | 6.4907 | 1.510 | D | O |
| A0A0A0MT26 | Sodium/potassium-transporting ATPase subunit alpha-3 OS=Homo sapiens GN=ATP1A3 PE=1 | 133231 | 5.6865 | 0.001 | D | O |
| A0A0A0MT30 | Aldo-keto reductase family 1 member C1 OS=Homo sapiens GN=AKR1C1 PE=1 SV=1 | 37092 | 7.8398 | 0.001 | D | O |
| A0A0A0MT35 | Prostamide/prostaglandin F synthase OS=Homo sapiens GN=FAM213B PE=1 SV=1 | 26393 | 6.5874 | 0.001 | D | O |
| A0A0A0MTI5 | Acyl-CoA-binding protein OS=Homo sapiens GN=DBI PE=1 SV=1 | 15948 | 4.7886 | 0.001 | D | O |
| A0A0A0MTR1 | Cadherin-13 OS=Homo sapiens GN=CDH13 PE=1 SV=2 | 76921 | 4.585 | 0.031 | D | O |
| A0A0A0MTS2 | Glucose-6-phosphate isomerase (Fragment) OS=Homo sapiens GN=GPI PE=1 SV=1 | 64784 | 9.3516 | 0.001 | D | O |
| A0A0A6YYA0 | Protein TMED7-TICAM2 OS=Homo sapiens GN=TMED7-TICAM2 PE=3 SV=1 | 21219 | 5.7759 | 0.034 | D | O |
| A0A0A6YYC0 | Ribosomal protein S6 kinase alpha-4 (Fragment) OS=Homo sapiens GN=RPS6KA4 PE=1 SV=: | 78317 | 7.3594 | 0.181 | D | O |
| A0A0B4J296 | Transcription elongation factor B polypeptide 2 OS=Homo sapiens GN=TCEB2 PE=1 SV=1 | 7751 | 10.4458 | 0.095 | D | O |
| A0A0B4J2C3 | Translationally-controlled tumor protein OS=Homo sapiens GN=TPT1 PE=1 SV=1 | 22559 | 4.9395 | 0.001 | D | O |
| A0A0C4DFT3 | Disks large homolog 1 OS=Homo sapiens GN=DLG1 PE=1 SV=1 | 99752 | 5.6309 | 0.006 | D | O |
| A0A0C4DFU1 | Superoxide dismutase OS=Homo sapiens GN=SOD2 PE=1 SV=1 | 20710 | 8.4067 | 0.001 | D | O |
| A0A0C4DFU2 | Superoxide dismutase OS=Homo sapiens GN=SOD2 PE=1 SV=1 | 24734 | 8.4038 | 0.001 | D | O |
| A0A0C4DFV9 | Protein SET OS=Homo sapiens GN=SET PE=1 SV=1 | 31105 | 3.9272 | 0.541 | D | O |
| A0A0C4DG17 | 40S ribosomal protein SA OS=Homo sapiens GN=RPSA PE=1 SV=1 | 33292 | 4.5938 | 0.001 | D | O |
| A0A0C4DG71 | Serologically defined colon cancer antigen 8 (Fragment) OS=Homo sapiens GN=SDCCAG8 PE | 48585 | 5.584 | 0.165 | D | O |
| A0A0C4DGN6 | ARF GTPase-activating protein GIT1 OS=Homo sapiens GN=GIT1 PE=1 SV=1 | 83080 | 6.4043 | 0.001 | D | O |
| A0A0C4DGS0 | NADH dehydrogenase [ubiquinone] 1 alpha subcomplex subunit 6 OS=Homo sapiens GN=NDU | 15126 | 10.4033 | 0.001 | D | O |
| A0A0C4DGS1 | Dolichyl-diphosphooligosaccharide--protein glycosyltransferase 48 kDa subunit OS=Homo sapie | 48768 | 5.3042 | 0.001 | D | O |
| A0A0C4DH22 | Band 4.1-like protein 1 OS=Homo sapiens GN=EPB41L1 PE=1 SV=1 | 98314 | 5.2866 | 0.127 | D | O |
| A0A0C4DH83 | EH domain-containing protein 3 OS=Homo sapiens GN=EHD3 PE=1 SV=1 | 61857 | 6.0439 | 0.001 | D | O |
| A0A0D9SF30 | Neural cell adhesion molecule 1 (Fragment) OS=Homo sapiens GN=NCAM1 PE=1 SV=1 | 47099 | 4.3975 | 0.277 | D | O |
| A0A0D9SF54 | Spectrin alpha chain, non-erythrocytic 1 OS=Homo sapiens GN=SPTAN1 PE=1 SV=1 | 282660 | 5.0493 | 1.380 | D | O |
| A0A0D9SFB1 | Dynamin-1 OS=Homo sapiens GN=DNM1 PE=1 SV=1 | 93958 | 6.1934 | 0.867 | D | O |
| A0A0D9SFL3 | RNA-binding protein EWS OS=Homo sapiens GN=EWSR1 PE=1 SV=1 | 61376 | 9.2607 | 0.001 | D | O |
| A0A0D9SGJ6 | Synaptojanin-1 OS=Homo sapiens GN=SYNJ1 PE=1 SV=1 | 168052 | 6.832 | 0.168 | D | O |
| A0A0G2JIW1 | Heat shock 70 kDa protein 1B OS=Homo sapiens GN=HSPA1B PE=1 SV=1 | 70066 | 5.3188 | 0.001 | D | O |
| A0A0G2JLL6 | PAXIP1-associated glutamate-rich protein 1 OS=Homo sapiens GN=PAGR1 PE=4 SV=1 | 36810 | 5.1284 | 0.001 | D | O |
| A0A0G2JMX7 | Microtubule-associated protein OS=Homo sapiens GN=MAPT PE=1 SV=1 | 80865 | 6.6577 | 0.001 | D | O |
| A0A0J9YWK4 | Hemoglobin subunit beta OS=Homo sapiens GN=HBB PE=4 SV=1 | 5987 | 7.0122 | 1.785 | D | O |
| A0A0J9YX34 | Tripartite motif-containing protein 2 OS=Homo sapiens GN=TRIM2 PE=4 SV=1 | 58417 | 5.7261 | 0.173 | D | O |
| A0A0J9YX62 | DnaJ homolog subfamily B member 6 OS=Homo sapiens GN=DNAJB6 PE=4 SV=1 | 36634 | 6.7852 | 0.001 | D | O |
| A0A0J9YX66 | CUGBP Elav-like family member 2 OS=Homo sapiens GN=CELF2 PE=4 SV=1 | 54879 | 8.9297 | 0.001 | D | O |
| A0A0J9YY01 | Unconventional myosin-XVB OS=Homo sapiens GN=MYO15B PE=4 SV=1 | 333513 | 7.481 | 0.451 | D | O |
| A1L390 | Pleckstrin homology domain-containing family G member 3 OS=Homo sapiens GN=PLEKHG3 F | 134329 | 6.104 | 0.065 | D | O |
| A2A274 | Aconitate hydratase, mitochondrial OS=Homo sapiens GN=ACO2 PE=1 SV=1 | 87765 | 6.9858 | 0.001 | D | O |
| A2A2D0 | Stathmin (Fragment) OS=Homo sapiens GN=STMN1 PE=1 SV=6 | 9787 | 7.7842 | 0.540 | D | O |
| A2A3R5 | 40S ribosomal protein S6 OS=Homo sapiens GN=RPS6 PE=1 SV=1 | 24953 | 11.562 | 0.018 | D | O |
| A2IDB2 | 14-3-3 protein eta (Fragment) OS=Homo sapiens GN=YWHAH PE=1 SV=1 | 18663 | 7.1704 | 8.591 | D | O |
| A6NE09 | 40S ribosomal protein SA OS=Homo sapiens GN=RPSAP58 PE=1 SV=1 | 32888 | 4.5923 | 0.328 | D | O |
| A6NHL2 | Tubulin alpha chain-like 3 OS=Homo sapiens GN=TUBAL3 PE=1 SV=2 | 49876 | 5.6235 | 0.008 | D | O |
| A6NMH8 | Tetraspanin OS=Homo sapiens GN=CD81 PE=1 SV=1 | 29786 | 6.5288 | 0.001 | D | O |
| A6NMY6 | Putative annexin A2-like protein OS=Homo sapiens GN=ANXA2P2 PE=5 SV=2 | 38634 | 6.5479 | 0.001 | D | O |
| A6NNI4 | Tetraspanin OS=Homo sapiens GN=CD9 PE=1 SV=1 | 17751 | 5.877 | 1.024 | D | O |
| A6NP52 | PRA1 family protein 2 OS=Homo sapiens GN=PRAF2 PE=1 SV=1 | 17177 | 8.9077 | 0.001 | D | O |
| A6XGL0 | YjeF N-terminal domain-containing protein 3 OS=Homo sapiens GN=YJEFN3 PE=1 SV=1 | 32564 | 5.6836 | 0.506 | D | O |

| | | | | | | |
|---|---|---|---|---|---|---|
| A8MT02 | Small nuclear ribonucleoprotein-associated proteins B and B' OS=Homo sapiens GN=SNRPB P | 29955 | 10.5791 | 0.001 | D | O |
| A8MUS3 | 60S ribosomal protein L23a OS=Homo sapiens GN=RPL23A PE=1 SV=1 | 21902 | 10.916 | 0.001 | D | O |
| A8MVZ9 | Fructose-bisphosphate aldolase OS=Homo sapiens GN=ALDOC PE=1 SV=1 | 36272 | 7.6567 | 1.216 | D | O |
| A8MX94 | Glutathione S-transferase P OS=Homo sapiens GN=GSTP1 PE=1 SV=1 | 19468 | 5.5737 | 0.680 | D | O |
| A8MXP9 | Matrin-3 OS=Homo sapiens GN=MATR3 PE=1 SV=1 | 99905 | 5.6309 | 0.001 | D | O |
| A8MZH3 | Myelin basic protein OS=Homo sapiens GN=MBP PE=1 SV=1 | 19717 | 11.7583 | 0.001 | D | O |
| B0QZ43 | Erlin-1 (Fragment) OS=Homo sapiens GN=ERLIN1 PE=1 SV=1 | 31057 | 8.127 | 0.011 | D | O |
| B1AJQ6 | Syntaxin-12 (Fragment) OS=Homo sapiens GN=STX12 PE=1 SV=2 | 24561 | 4.8911 | 0.107 | D | O |
| B1AKR6 | Dynein light chain roadblock-type 1 OS=Homo sapiens GN=DYNLRB1 PE=1 SV=1 | 16242 | 6.8276 | 0.187 | D | O |
| B1AKY9 | Sodium/potassium-transporting ATPase subunit alpha OS=Homo sapiens GN=ATP1A2 PE=1 S | 110791 | 5.2749 | 0.538 | D | O |
| B1AMS2 | Septin 6, isoform CRA_b OS=Homo sapiens GN=SEPT6 PE=1 SV=1 | 49272 | 6.3589 | 1.958 | D | O |
| B1ANG9 | Guanylate kinase OS=Homo sapiens GN=GUK1 PE=1 SV=2 | 25320 | 7.3228 | 0.001 | D | O |
| B1B1G3 | Myelin proteolipid protein (Fragment) OS=Homo sapiens GN=PLP1 PE=1 SV=6 | 13203 | 6.0586 | 0.317 | D | O |
| B2R4S9 | Histone H2B OS=Homo sapiens GN=HIST1H2BC PE=2 SV=1 | 13897 | 10.7402 | 0.001 | D | O |
| B2R5W2 | Heterogeneous nuclear ribonucleoproteins C1/C2 OS=Homo sapiens GN=HNRNPC PE=1 SV= | 31928 | 4.9087 | 0.001 | D | O |
| B4DDD6 | Drebrin-like protein OS=Homo sapiens GN=DBNL PE=1 SV=1 | 45718 | 4.7373 | 0.075 | D | O |
| B4DEB1 | Histone H3 OS=Homo sapiens GN=H3F3A PE=1 SV=1 | 14043 | 11.7466 | 0.001 | D | O |
| B4DGU4 | Catenin beta-1 OS=Homo sapiens GN=CTNNB1 PE=1 SV=1 | 84711 | 5.499 | 0.081 | D | O |
| B4DJ62 | HCG2002594, isoform CRA_a OS=Homo sapiens GN=SEPT5 PE=1 SV=1 | 37382 | 5.6616 | 0.091 | D | O |
| B4DJV2 | Citrate synthase OS=Homo sapiens GN=CS PE=1 SV=1 | 50399 | 7.8604 | 0.881 | D | O |
| B4DT28 | Heterogeneous nuclear ribonucleoprotein R OS=Homo sapiens GN=HNRNPR PE=1 SV=1 | 55683 | 9.4541 | 0.117 | D | O |
| B4DUR8 | T-complex protein 1 subunit gamma OS=Homo sapiens GN=CCT3 PE=1 SV=1 | 55638 | 5.3145 | 0.111 | D | O |
| B4DV12 | Polyubiquitin-B OS=Homo sapiens GN=UBB PE=1 SV=1 | 17204 | 7.3169 | 0.001 | D | O |
| B5MCD7 | Synaptogyrin-1 OS=Homo sapiens GN=SYNGR1 PE=1 SV=1 | 18453 | 6.8643 | 0.565 | D | O |
| B5MDF5 | GTP-binding nuclear protein Ran OS=Homo sapiens GN=RAN PE=1 SV=1 | 26207 | 6.5566 | 0.001 | D | O |
| B7WNR0 | Serum albumin OS=Homo sapiens GN=ALB PE=1 SV=1 | 56175 | 6.8027 | 0.001 | D | O |
| B7Z2R2 | Cytochrome b-c1 complex subunit 7 OS=Homo sapiens GN=UQCRB PE=1 SV=1 | 18725 | 9.3164 | 0.001 | D | O |
| B7Z4Y8 | Butyrophilin-like protein 9 OS=Homo sapiens GN=BTNL9 PE=1 SV=1 | 27525 | 9.876 | 3.287 | D | O |
| B7Z613 | Neuronal membrane glycoprotein M6-b OS=Homo sapiens GN=GPM6B PE=1 SV=1 | 33250 | 5.9575 | 0.450 | D | O |
| B7Z6D5 | Probable ATP-dependent RNA helicase DDX27 OS=Homo sapiens GN=DDX27 PE=1 SV=1 | 86550 | 9.6665 | 0.336 | D | O |
| B7Z6Z4 | Myosin light polypeptide 6 OS=Homo sapiens GN=MYL6 PE=1 SV=1 | 26689 | 4.8149 | 0.001 | D | O |
| B7Z9C2 | Nucleosome assembly protein 1-like 1 OS=Homo sapiens GN=NAP1L1 PE=1 SV=1 | 40486 | 4.2686 | 0.001 | D | O |
| B7Z9X4 | Protein NDRG4 OS=Homo sapiens GN=NDRG4 PE=1 SV=1 | 30742 | 5.603 | 0.001 | D | O |
| B7ZAR1 | T-complex protein 1 subunit epsilon OS=Homo sapiens GN=CCT5 PE=1 SV=1 | 55313 | 5.1577 | 0.001 | D | O |
| B7ZC38 | Endophilin-B2 OS=Homo sapiens GN=SH3GLB2 PE=1 SV=1 | 44333 | 5.4829 | 0.001 | D | O |
| B8ZZ51 | Malate dehydrogenase, cytoplasmic OS=Homo sapiens GN=MDH1 PE=1 SV=1 | 18677 | 5.5532 | 0.859 | D | O |
| B8ZZB8 | CB1 cannabinoid receptor-interacting protein 1 OS=Homo sapiens GN=CNRIP1 PE=1 SV=1 | 15158 | 9.7544 | 0.586 | D | O |
| B8ZZL8 | 10 kDa heat shock protein, mitochondrial OS=Homo sapiens GN=HSPE1 PE=1 SV=1 | 10682 | 10.0679 | 1.026 | D | O |
| B9A067 | MICOS complex subunit MIC60 OS=Homo sapiens GN=IMMT PE=1 SV=2 | 78925 | 6.6357 | 2.699 | D | O |
| C9J0J7 | Profilin-2 OS=Homo sapiens GN=PFN2 PE=1 SV=1 | 9834 | 9.5332 | 0.721 | D | O |
| C9J0K6 | Sorcin OS=Homo sapiens GN=SRI PE=1 SV=1 | 17593 | 5.1929 | 0.269 | D | O |
| C9J1Z8 | ADP-ribosylation factor 5 (Fragment) OS=Homo sapiens GN=ARF5 PE=1 SV=1 | 17095 | 7.3125 | 1.062 | D | O |
| C9J712 | Profilin-2 OS=Homo sapiens GN=PFN2 PE=1 SV=1 | 9791 | 9.7266 | 0.054 | D | O |
| C9J9E2 | CaM kinase-like vesicle-associated protein OS=Homo sapiens GN=CAMKV PE=1 SV=1 | 49606 | 8.9795 | 0.001 | D | O |
| C9J9W2 | LIM and SH3 domain protein 1 (Fragment) OS=Homo sapiens GN=LASP1 PE=1 SV=1 | 18968 | 9.3428 | 0.157 | D | O |
| C9JC84 | Fibrinogen gamma chain OS=Homo sapiens GN=FGG PE=1 SV=1 | 52304 | 5.2427 | 0.001 | D | O |
| C9JFR7 | Cytochrome c (Fragment) OS=Homo sapiens GN=CYCS PE=1 SV=1 | 11325 | 10.0488 | 1.052 | D | O |
| C9JIZ6 | Prosaposin OS=Homo sapiens GN=PSAP PE=1 SV=2 | 58402 | 4.8853 | 0.001 | D | O |
| C9JL73 | V-type proton ATPase subunit B, kidney isoform OS=Homo sapiens GN=ATP6V1B1 PE=1 SV= | 55046 | 5.3086 | 0.036 | D | O |
| C9JPM4 | ADP-ribosylation factor 4 (Fragment) OS=Homo sapiens GN=ARF4 PE=1 SV=1 | 14543 | 8.6411 | 0.007 | D | O |
| C9JQS9 | Propionyl-CoA carboxylase beta chain, mitochondrial OS=Homo sapiens GN=PCCB PE=1 SV= | 60698 | 7.894 | 0.041 | D | O |
| C9JRD2 | DnaJ homolog subfamily B member 2 (Fragment) OS=Homo sapiens GN=DNAJB2 PE=1 SV=1 | 25395 | 5.3965 | 0.018 | D | O |
| C9JRZ6 | MICOS complex subunit MIC19 OS=Homo sapiens GN=CHCHD3 PE=1 SV=1 | 26677 | 8.6221 | 0.001 | D | O |
| C9JRZ8 | Aldo-keto reductase family 1 member B15 OS=Homo sapiens GN=AKR1B15 PE=1 SV=2 | 36513 | 6.2373 | 1.861 | D | O |
| C9JYY6 | Neuronal cell adhesion molecule OS=Homo sapiens GN=NRCAM PE=1 SV=3 | 133729 | 5.4082 | 0.291 | D | O |
| D6R960 | Complexin-2 (Fragment) OS=Homo sapiens GN=CPLX2 PE=1 SV=1 | 13626 | 4.6948 | 0.001 | D | O |
| D6R9Z7 | Cytochrome c oxidase subunit 7C, mitochondrial OS=Homo sapiens GN=COX7C PE=1 SV=1 | 6378 | 10.1997 | 0.001 | D | O |
| D6RA31 | Alpha-synuclein (Fragment) OS=Homo sapiens GN=SNCA PE=1 SV=6 | 6815 | 9.8467 | 3.249 | D | O |
| D6RAN4 | 60S ribosomal protein L9 (Fragment) OS=Homo sapiens GN=RPL9 PE=1 SV=6 | 20762 | 10.6172 | 0.001 | D | O |
| D6RBW1 | Eukaryotic translation initiation factor 4E OS=Homo sapiens GN=EIF4E PE=1 SV=1 | 28494 | 8.228 | 0.001 | D | O |
| D6RC06 | Histidine triad nucleotide-binding protein 1 OS=Homo sapiens GN=HINT1 PE=1 SV=1 | 7318 | 10.043 | 0.934 | D | O |
| D6RE83 | Ubiquitin carboxyl-terminal hydrolase OS=Homo sapiens GN=UCHL1 PE=1 SV=1 | 23159 | 5.1812 | 1.666 | D | O |
| D6RER5 | Septin-11 OS=Homo sapiens GN=SEPT11 PE=1 SV=1 | 49777 | 6.23 | 0.001 | D | O |
| D6RF62 | Multifunctional protein ADE2 OS=Homo sapiens GN=PAICS PE=1 SV=1 | 37087 | 5.8638 | 0.126 | D | O |
| D6RFM5 | Succinate dehydrogenase [ubiquinone] flavoprotein subunit, mitochondrial OS=Homo sapiens G | 63526 | 6.8394 | 0.237 | D | O |
| D6RG13 | 40S ribosomal protein S3a (Fragment) OS=Homo sapiens GN=RPS3A PE=1 SV=1 | 25591 | 10.0884 | 0.001 | D | O |
| E5KLJ5 | Dynamin-like 120 kDa protein, mitochondrial OS=Homo sapiens GN=OPA1 PE=1 SV=1 | 117669 | 7.5952 | 0.001 | D | O |
| E5RGS4 | Prefoldin subunit 1 OS=Homo sapiens GN=PFDN1 PE=1 SV=1 | 13457 | 5.231 | 0.086 | D | O |
| E5RHK8 | Dynamin-3 OS=Homo sapiens GN=DNM3 PE=1 SV=1 | 72844 | 8.7495 | 0.169 | D | O |
| E5RHP7 | Carbonic anhydrase 1 (Fragment) OS=Homo sapiens GN=CA1 PE=1 SV=1 | 27736 | 6.1187 | 0.359 | D | O |
| E5RHW4 | Erlin-2 (Fragment) OS=Homo sapiens GN=ERLIN2 PE=1 SV=1 | 37701 | 5.3232 | 0.059 | D | O |
| E5RJ29 | PH and SEC7 domain-containing protein 3 OS=Homo sapiens GN=PSD3 PE=1 SV=1 | 108923 | 5.6528 | 0.001 | D | O |
| E5RJR5 | S-phase kinase-associated protein 1 OS=Homo sapiens GN=SKP1 PE=1 SV=1 | 18708 | 4.3696 | 0.001 | D | O |
| E7EMB3 | Calmodulin OS=Homo sapiens GN=CALM2 PE=1 SV=1 | 21675 | 4.2524 | 0.001 | D | O |
| E7EMM4 | Acid ceramidase OS=Homo sapiens GN=ASAH1 PE=1 SV=1 | 41769 | 8.1343 | 0.163 | D | O |
| E7ENQ6 | Uncharacterized protein OS=Homo sapiens PE=4 SV=1 | 30075 | 6.5039 | 0.744 | D | O |
| E7ENV7 | Copine-8 OS=Homo sapiens GN=CPNE8 PE=1 SV=2 | 62194 | 5.5327 | 0.011 | D | O |
| E7ENY0 | Alpha-adducin OS=Homo sapiens GN=ADD1 PE=1 SV=1 | 73358 | 6.0938 | 0.001 | D | O |
| E7EPB3 | 60S ribosomal protein L14 OS=Homo sapiens GN=RPL14 PE=1 SV=1 | 14548 | 10.6597 | 0.224 | D | O |
| E7EPK1 | Septin-7 OS=Homo sapiens GN=SEPT7 PE=1 SV=2 | 50662 | 8.8887 | 0.001 | D | O |
| E7EPT4 | NADH dehydrogenase [ubiquinone] flavoprotein 2, mitochondrial OS=Homo sapiens GN=NDUF | 27889 | 7.9966 | 0.001 | D | O |

| | | | | | | |
|---|---|---|---|---|---|---|
| E7EPV7 | Alpha-synuclein OS=Homo sapiens GN=SNCA PE=1 SV=1 | 11769 | 9.7354 | 1.872 | D | O |
| E7EQB8 | Isocitrate dehydrogenase [NAD] subunit, mitochondrial OS=Homo sapiens GN=IDH3G PE=1 S\ | 37043 | 8.7671 | 0.001 | D | O |
| E7EQR4 | Ezrin OS=Homo sapiens GN=EZR PE=1 SV=3 | 69328 | 5.7876 | 0.001 | D | O |
| E7ESP9 | Neurofilament medium polypeptide OS=Homo sapiens GN=NEFM PE=1 SV=1 | 98322 | 4.6523 | 0.630 | D | O |
| E7EU96 | Casein kinase II subunit alpha OS=Homo sapiens GN=CSNK2A1 PE=1 SV=1 | 45281 | 7.9512 | 0.104 | D | O |
| E7EUL7 | Sperm-specific antigen 2 OS=Homo sapiens GN=SSFA2 PE=1 SV=1 | 84539 | 5.1973 | 0.060 | D | O |
| E7EVA0 | Microtubule-associated protein OS=Homo sapiens GN=MAP4 PE=1 SV=1 | 245289 | 5.833 | 0.317 | D | O |
| E7EVC7 | Autophagy-related protein 16-1 OS=Homo sapiens GN=ATG16L1 PE=1 SV=1 | 69967 | 6.1685 | 0.001 | D | O |
| E7EVJ5 | Cytoplasmic FMR1-interacting protein 2 OS=Homo sapiens GN=CYFIP2 PE=1 SV=1 | 142484 | 6.7661 | 0.142 | D | O |
| E7EW69 | Septin-10 OS=Homo sapiens GN=SEPT10 PE=1 SV=1 | 52112 | 6.4717 | 0.007 | D | O |
| E7EX29 | 14-3-3 protein zeta/delta (Fragment) OS=Homo sapiens GN=YWHAZ PE=1 SV=1 | 28018 | 4.6523 | 0.001 | D | O |
| E9PAV3 | Nascent polypeptide-associated complex subunit alpha, muscle-specific form OS=Homo sapien | 205293 | 10.0386 | 0.318 | D | O |
| E9PB61 | THO complex subunit 4 OS=Homo sapiens GN=ALYREF PE=1 SV=1 | 27540 | 11.4551 | 0.001 | D | O |
| E9PBG7 | Calcium/calmodulin-dependent protein kinase type II subunit delta OS=Homo sapiens GN=CAM | 57726 | 6.8745 | 0.049 | D | O |
| E9PC15 | Acylglycerol kinase, mitochondrial OS=Homo sapiens GN=AGK PE=1 SV=1 | 43769 | 5.9312 | 0.055 | D | O |
| E9PCR7 | 2-oxoglutarate dehydrogenase, mitochondrial OS=Homo sapiens GN=OGDH PE=1 SV=1 | 117590 | 6.4482 | 0.001 | D | O |
| E9PDE8 | Heat shock 70 kDa protein 4L OS=Homo sapiens GN=HSPA4L PE=1 SV=1 | 91895 | 5.6924 | 0.089 | D | O |
| E9PDG8 | Clathrin coat assembly protein AP180 OS=Homo sapiens GN=SNAP91 PE=1 SV=1 | 91913 | 4.522 | 0.704 | D | O |
| E9PDL2 | Dipeptidyl aminopeptidase-like protein 6 OS=Homo sapiens GN=DPP6 PE=1 SV=1 | 86272 | 5.8081 | 0.189 | D | O |
| E9PEJ4 | Acetyltransferase component of pyruvate dehydrogenase complex OS=Homo sapiens GN=DLA | 57550 | 9.2051 | 0.198 | D | O |
| E9PEX6 | Dihydrolipoyl dehydrogenase OS=Homo sapiens GN=DLD PE=1 SV=1 | 51782 | 7.9233 | 0.370 | D | O |
| E9PF17 | Versican core protein OS=Homo sapiens GN=VCAN PE=1 SV=2 | 176718 | 4.5176 | 0.659 | D | O |
| E9PF58 | Actin-related protein 2/3 complex subunit 1A OS=Homo sapiens GN=ARPC1A PE=1 SV=1 | 30868 | 8.8184 | 0.146 | D | O |
| E9PGT6 | COP9 signalosome complex subunit 8 OS=Homo sapiens GN=COPS8 PE=1 SV=1 | 19331 | 5.2295 | 0.001 | D | O |
| E9PH64 | NADH dehydrogenase [ubiquinone] 1 beta subcomplex subunit 9 OS=Homo sapiens GN=NDUF | 20370 | 7.9688 | 0.105 | D | O |
| E9PHB5 | Catenin delta-2 OS=Homo sapiens GN=CTNND2 PE=1 SV=1 | 99341 | 7.0034 | 0.052 | D | O |
| E9PIA8 | Palmitoyl-protein thioesterase 1 (Fragment) OS=Homo sapiens GN=PPT1 PE=1 SV=7 | 22894 | 7.207 | 0.001 | D | O |
| E9PIE4 | Mitochondrial carrier homolog 2 (Fragment) OS=Homo sapiens GN=MTCH2 PE=1 SV=6 | 28530 | 7.4839 | 0.262 | D | O |
| E9PII3 | Band 4.1-like protein 2 OS=Homo sapiens GN=EPB41L2 PE=1 SV=1 | 79208 | 6.3398 | 0.076 | D | O |
| E9PIM6 | Thy-1 membrane glycoprotein (Fragment) OS=Homo sapiens GN=THY1 PE=1 SV=6 | 16916 | 9.2461 | 0.001 | D | O |
| E9PJH7 | Mitochondrial glutamate carrier 1 (Fragment) OS=Homo sapiens GN=SLC25A22 PE=1 SV=5 | 33260 | 10.0063 | 0.001 | D | O |
| E9PJL7 | Alpha-crystallin B chain (Fragment) OS=Homo sapiens GN=CRYAB PE=1 SV=6 | 15338 | 6.1362 | 0.001 | D | O |
| E9PK25 | Cofilin-1 OS=Homo sapiens GN=CFL1 PE=1 SV=1 | 22713 | 8.5181 | 0.001 | D | O |
| E9PKD5 | 26S protease regulatory subunit 6A (Fragment) OS=Homo sapiens GN=PSMC3 PE=1 SV=2 | 34592 | 5.0332 | 0.076 | D | O |
| E9PL57 | Protein NEDD8-MDP1 (Fragment) OS=Homo sapiens GN=NEDD8-MDP1 PE=4 SV=1 | 19524 | 7.4004 | 0.001 | D | O |
| E9PMV1 | Plectin (Fragment) OS=Homo sapiens GN=PLEC PE=1 SV=1 | 80730 | 6.3018 | 0.260 | D | O |
| E9PNK6 | Tumor protein D53 OS=Homo sapiens GN=TPD52L1 PE=1 SV=1 | 18649 | 5.4888 | 0.081 | D | O |
| E9PPQ4 | Ferritin (Fragment) OS=Homo sapiens GN=FTH1 PE=1 SV=1 | 6659 | 5.4185 | 0.394 | D | O |
| E9PPU1 | 40S ribosomal protein S3 OS=Homo sapiens GN=RPS3 PE=1 SV=1 | 17396 | 9.9609 | 0.001 | D | O |
| E9PQD7 | 40S ribosomal protein S2 OS=Homo sapiens GN=RPS2 PE=1 SV=1 | 25195 | 10.6597 | 0.124 | D | O |
| E9PQY2 | Prefoldin subunit 4 OS=Homo sapiens GN=PFDN4 PE=1 SV=1 | 15588 | 4.2803 | 0.001 | D | O |
| E9PR46 | ATP-dependent RNA helicase DDX25 (Fragment) OS=Homo sapiens GN=DDX25 PE=1 SV=2 | 46484 | 6.5537 | 0.142 | D | O |
| E9PRK8 | Ferritin OS=Homo sapiens GN=FTH1 PE=1 SV=1 | 11161 | 9.022 | 0.554 | D | O |
| F1T0E5 | Calcium-dependent secretion activator 1 OS=Homo sapiens GN=CADPS PE=1 SV=1 | 151904 | 5.5342 | 0.121 | D | O |
| F2Z2Y4 | Pyridoxal kinase OS=Homo sapiens GN=PDXK PE=1 SV=1 | 30618 | 6.189 | 0.091 | D | O |
| F5GX30 | Cation-dependent mannose-6-phosphate receptor OS=Homo sapiens GN=M6PR PE=1 SV=2 | 21524 | 6.2358 | 0.088 | D | O |
| F5GXR3 | Parathymosin OS=Homo sapiens GN=PTMS PE=1 SV=1 | 12066 | 11.4668 | 0.001 | D | O |
| F5GY55 | DNA damage-binding protein 1 OS=Homo sapiens GN=DDB1 PE=1 SV=1 | 121636 | 5.1914 | 0.016 | D | O |
| F5GYN4 | Ubiquitin thioesterase OTUB1 OS=Homo sapiens GN=OTUB1 PE=1 SV=1 | 28032 | 4.9849 | 0.313 | D | O |
| F5GYQ1 | V-type proton ATPase subunit d 1 OS=Homo sapiens GN=ATP6V0D1 PE=1 SV=1 | 44631 | 4.8457 | 0.001 | D | O |
| F5GZC2 | Synaptotagmin-7 OS=Homo sapiens GN=SYT7 PE=1 SV=1 | 50668 | 9.3501 | 0.001 | D | O |
| F5GZS6 | 4F2 cell-surface antigen heavy chain OS=Homo sapiens GN=SLC3A2 PE=1 SV=1 | 64832 | 4.7813 | 0.272 | D | O |
| F5GZY7 | Gamma-aminobutyric acid receptor-associated protein-like 1 (Fragment) OS=Homo sapiens GN | 8571 | 10.3184 | 0.048 | D | O |
| F5H157 | Ras-related protein Rab-35 (Fragment) OS=Homo sapiens GN=RAB35 PE=1 SV=1 | 21200 | 8.4126 | 0.513 | D | O |
| F5H1U9 | Multiple PDZ domain protein OS=Homo sapiens GN=MPDZ PE=1 SV=1 | 222986 | 4.7622 | 0.001 | D | O |
| F5H423 | Uncharacterized protein OS=Homo sapiens PE=3 SV=1 | 23331 | 9.0981 | 0.001 | D | O |
| F5H481 | Protein N-lysine methyltransferase METTL20 (Fragment) OS=Homo sapiens GN=METTL20 PE | 7875 | 7.0781 | 1.471 | D | O |
| F5H5D3 | Tubulin alpha-1C chain OS=Homo sapiens GN=TUBA1C PE=1 SV=1 | 57693 | 4.7637 | 0.001 | D | O |
| F5H5G1 | Limbic system-associated membrane protein OS=Homo sapiens GN=LSAMP PE=1 SV=2 | 31722 | 5.5635 | 0.488 | D | O |
| F5H608 | ATP synthase subunit d, mitochondrial OS=Homo sapiens GN=ATP5H PE=1 SV=2 | 8909 | 9.835 | 0.432 | D | O |
| F5H6T1 | ARP2 actin-related protein 2 homolog (Yeast), isoform CRA_d OS=Homo sapiens GN=ACTR2 I | 38817 | 5.8521 | 0.001 | D | O |
| F5H6X6 | Neutral alpha-glucosidase AB OS=Homo sapiens GN=GANAB PE=1 SV=1 | 96155 | 5.3262 | 0.131 | D | O |
| F5H7S3 | Tropomyosin alpha-1 chain OS=Homo sapiens GN=TPM1 PE=1 SV=2 | 28507 | 4.5454 | 0.642 | D | O |
| F6RFD5 | Destrin OS=Homo sapiens GN=DSTN PE=1 SV=1 | 15386 | 8.748 | 0.575 | D | O |
| F6TLX2 | Glyoxalase domain-containing protein 4 OS=Homo sapiens GN=GLOD4 PE=1 SV=1 | 54684 | 8.7451 | 0.001 | D | O |
| F6U236 | Protein kinase C and casein kinase substrate in neurons protein 1 OS=Homo sapiens GN=PAC | 46117 | 5.0786 | 0.001 | D | O |
| F6VDH7 | Hsc70-interacting protein (Fragment) OS=Homo sapiens GN=ST13 PE=1 SV=1 | 18074 | 4.4707 | 0.136 | D | O |
| F6X2W2 | Neuronal growth regulator 1 OS=Homo sapiens GN=NEGR1 PE=1 SV=1 | 32829 | 6.4731 | 0.139 | D | O |
| F8VSD4 | Ubiquitin-conjugating enzyme E2 N OS=Homo sapiens GN=UBE2N PE=1 SV=1 | 11975 | 9.5171 | 0.263 | D | O |
| F8VXU5 | Vacuolar protein sorting-associated protein 29 OS=Homo sapiens GN=VPS29 PE=1 SV=1 | 23964 | 8.25 | 0.001 | D | O |
| F8VZX2 | Poly(rC)-binding protein 2 OS=Homo sapiens GN=PCBP2 PE=1 SV=1 | 33778 | 8.2822 | 0.125 | D | O |
| F8W6I7 | Heterogeneous nuclear ribonucleoprotein A1 OS=Homo sapiens GN=HNRNPA1 PE=1 SV=2 | 33134 | 9.4336 | 0.001 | D | O |
| F8W726 | Ubiquitin-associated protein 2-like OS=Homo sapiens GN=UBAP2L PE=1 SV=2 | 113558 | 6.2051 | 0.076 | D | O |
| F8W9L4 | Fibroblast growth factor receptor OS=Homo sapiens GN=FGFR3 PE=1 SV=1 | 85157 | 6.8042 | 0.006 | D | O |
| F8W9U4 | Microtubule-associated protein OS=Homo sapiens GN=MAP4 PE=1 SV=1 | 88222 | 9.6035 | 0.102 | D | O |
| F8WCA0 | Vesicle-associated membrane protein 2 OS=Homo sapiens GN=VAMP2 PE=4 SV=1 | 12925 | 8.7012 | 0.001 | D | O |
| F8WCF6 | Protein ARPC4-TTLL3 OS=Homo sapiens GN=ARPC4-TTLL3 PE=4 SV=1 | 21045 | 9.186 | 0.001 | D | O |
| G3V0I5 | NADH dehydrogenase (Ubiquinone) flavoprotein 1, 51kDa, isoform CRA_c OS=Homo sapiens ( | 50022 | 8.1533 | 0.142 | D | O |
| G3V126 | ATPase, H+ transporting, lysosomal 50/57kDa, V1 subunit H, isoform CRA_c OS=Homo sapien | 51536 | 5.8037 | 0.221 | D | O |
| G3V192 | Ferritin OS=Homo sapiens GN=FTH1 PE=1 SV=1 | 17642 | 5.1694 | 0.001 | D | O |
| G3V1N2 | HCG1745306, isoform CRA_a OS=Homo sapiens GN=HBA2 PE=1 SV=1 | 11940 | 9.3018 | 3.335 | D | O |

| | | | | | | |
|---|---|---|---|---|---|---|
| G3V200 | Liprin-alpha-2 OS=Homo sapiens GN=PPFIA2 PE=1 SV=2 | 142019 | 5.625 | 0.052 | D | O |
| G3V295 | Proteasome subunit alpha type OS=Homo sapiens GN=PSMA6 PE=1 SV=1 | 22827 | 8.3555 | 0.100 | D | O |
| G3V2N0 | Guanine nucleotide-binding protein subunit gamma OS=Homo sapiens GN=GNG2 PE=1 SV=1 | 12378 | 9.3047 | 0.001 | D | O |
| G3V325 | Protein ATP5J2-PTCD1 OS=Homo sapiens GN=ATP5J2-PTCD1 PE=4 SV=1 | 84056 | 8.8682 | 0.304 | D | O |
| G3V4P8 | Glia maturation factor beta (Fragment) OS=Homo sapiens GN=GMFB PE=1 SV=1 | 17500 | 5.0332 | 0.001 | D | O |
| G3V5X7 | Telomerase protein component 1 OS=Homo sapiens GN=TEP1 PE=1 SV=1 | 277845 | 7.3198 | 0.099 | D | O |
| G3V5Z7 | Proteasome subunit alpha type OS=Homo sapiens GN=PSMA6 PE=1 SV=1 | 28129 | 6.3677 | 0.001 | D | O |
| G3XAL0 | Malate dehydrogenase OS=Homo sapiens GN=MDH2 PE=1 SV=1 | 24578 | 8.0024 | 1.273 | D | O |
| G3XAM7 | Catenin (Cadherin-associated protein), alpha 1, 102kDa, isoform CRA_a OS=Homo sapiens GN | 92663 | 5.1855 | 0.001 | D | O |
| G8JLB6 | Heterogeneous nuclear ribonucleoprotein H OS=Homo sapiens GN=HNRNPH1 PE=1 SV=1 | 51197 | 6.3442 | 0.001 | D | O |
| H0Y3Y4 | Septin-7 (Fragment) OS=Homo sapiens GN=SEPT7 PE=1 SV=1 | 43009 | 7.7446 | 0.523 | D | O |
| H0Y614 | Ubiquitin-fold modifier 1 (Fragment) OS=Homo sapiens GN=UFM1 PE=1 SV=1 | 8656 | 9.8643 | 0.062 | D | O |
| H0Y650 | Dynein assembly factor 5, axonemal (Fragment) OS=Homo sapiens GN=DNAAF5 PE=1 SV=1 | 72538 | 5.9912 | 0.063 | D | O |
| H0Y6T8 | Ras-related protein Rab-18 (Fragment) OS=Homo sapiens GN=RAB18 PE=1 SV=1 | 32954 | 9.1875 | 0.020 | D | O |
| H0Y7S3 | Plasma membrane calcium-transporting ATPase 2 (Fragment) OS=Homo sapiens GN=ATP2B2 | 121188 | 5.6323 | 0.050 | D | O |
| H0Y8G5 | Heterogeneous nuclear ribonucleoprotein D0 (Fragment) OS=Homo sapiens GN=HNRNPD PE= | 29648 | 9.4526 | 0.001 | D | O |
| H0YAM0 | Carboxypeptidase E (Fragment) OS=Homo sapiens GN=CPE PE=1 SV=1 | 12047 | 5.1812 | 0.108 | D | O |
| H0YD93 | RUN and FYVE domain-containing protein 2 (Fragment) OS=Homo sapiens GN=RUFY2 PE=1 S | 71390 | 5.6265 | 0.029 | D | O |
| H0YDD4 | Acetyltransferase component of pyruvate dehydrogenase complex (Fragment) OS=Homo sapien | 51169 | 8.6733 | 0.201 | D | O |
| H0YDN1 | Plectin (Fragment) OS=Homo sapiens GN=PLEC PE=1 SV=6 | 23731 | 4.6934 | 0.232 | D | O |
| H0YDU8 | Serine/threonine-protein phosphatase (Fragment) OS=Homo sapiens GN=PPP5C PE=1 SV=1 | 55172 | 5.7773 | 0.081 | D | O |
| H0YE29 | Rho GTPase-activating protein 1 (Fragment) OS=Homo sapiens GN=ARHGAP1 PE=1 SV=1 | 38363 | 5.792 | 0.095 | D | O |
| H0YH81 | ATP synthase subunit beta (Fragment) OS=Homo sapiens GN=ATP5B PE=1 SV=1 | 38226 | 5.2251 | 0.454 | D | O |
| H0YHD8 | Cysteine-rich protein 2 (Fragment) OS=Homo sapiens GN=CRIP2 PE=1 SV=1 | 11152 | 8.3994 | 0.107 | D | O |
| H0YJ21 | Cytoplasmic dynein 1 heavy chain 1 (Fragment) OS=Homo sapiens GN=DYNC1H1 PE=1 SV=1 | 21009 | 7.0942 | 0.107 | D | O |
| H0YK48 | Tropomyosin alpha-1 chain OS=Homo sapiens GN=TPM1 PE=1 SV=1 | 28562 | 4.5396 | 0.098 | D | O |
| H0YL12 | Electron transfer flavoprotein subunit alpha, mitochondrial (Fragment) OS=Homo sapiens GN=E | 24893 | 6.0967 | 0.188 | D | O |
| H0YLA2 | Signal recognition particle 14 kDa protein OS=Homo sapiens GN=SRP14 PE=1 SV=1 | 13049 | 9.9697 | 0.083 | D | O |
| H0YN26 | Acidic leucine-rich nuclear phosphoprotein 32 family member A OS=Homo sapiens GN=ANP32 | 19985 | 4.27 | 0.120 | D | O |
| H3BLU7 | Aflatoxin B1 aldehyde reductase member 2 (Fragment) OS=Homo sapiens GN=AKR7A2 PE=1 | 34662 | 6.7588 | 0.201 | D | O |
| H3BMH2 | Ras-related protein Rab-11A (Fragment) OS=Homo sapiens GN=RAB11A PE=3 SV=1 | 17657 | 8.9253 | 0.305 | D | O |
| H3BN14 | Calretinin (Fragment) OS=Homo sapiens GN=CALB2 PE=1 SV=2 | 27299 | 5.1519 | 0.126 | D | O |
| H3BNX8 | Cytochrome c oxidase subunit 5A, mitochondrial OS=Homo sapiens GN=COX5A PE=1 SV=1 | 17223 | 5.603 | 0.001 | D | O |
| H3BPJ9 | NADH dehydrogenase [ubiquinone] 1 beta subcomplex subunit 10 OS=Homo sapiens GN=NDU | 19245 | 8.8608 | 0.447 | D | O |
| H3BPK3 | Hydroxyacylglutathione hydrolase, mitochondrial (Fragment) OS=Homo sapiens GN=HAGH PE | 26399 | 8.2705 | 0.158 | D | O |
| H3BQI7 | Inactive hydroxysteroid dehydrogenase-like protein 1 (Fragment) OS=Homo sapiens GN=HSDL | 12102 | 8.8579 | 0.058 | D | O |
| H3BQN4 | Fructose-bisphosphate aldolase OS=Homo sapiens GN=ALDOA PE=1 SV=1 | 39315 | 8.4331 | 0.977 | D | O |
| H3BR70 | Pyruvate kinase OS=Homo sapiens GN=PKM PE=1 SV=1 | 40163 | 8.0171 | 1.724 | D | O |
| H3BRG4 | Cytochrome b-c1 complex subunit 2, mitochondrial OS=Homo sapiens GN=UQCRC2 PE=1 SV= | 44607 | 9.2827 | 0.001 | D | O |
| H3BRN4 | 4-aminobutyrate aminotransferase, mitochondrial OS=Homo sapiens GN=ABAT PE=1 SV=1 | 58103 | 8.0171 | 0.001 | D | O |
| H3BRV9 | Nuclear transport factor 2 (Fragment) OS=Homo sapiens GN=NUTF2 PE=1 SV=1 | 12150 | 4.9321 | 0.284 | D | O |
| H3BTN5 | Pyruvate kinase (Fragment) OS=Homo sapiens GN=PKM PE=1 SV=1 | 53011 | 6.3926 | 0.298 | D | O |
| H7BYD0 | NADH dehydrogenase [ubiquinone] 1 alpha subcomplex subunit 5 (Fragment) OS=Homo sapien | 13091 | 4.9189 | 0.108 | D | O |
| H7BYH4 | Superoxide dismutase [Cu-Zn] OS=Homo sapiens GN=SOD1 PE=1 SV=1 | 13900 | 5.6558 | 0.221 | D | O |
| H7BYW5 | cAMP-dependent protein kinase type I-beta regulatory subunit (Fragment) OS=Homo sapiens G | 27032 | 8.6836 | 0.060 | D | O |
| H7C1V0 | Cathepsin D (Fragment) OS=Homo sapiens GN=CTSD PE=1 SV=1 | 20358 | 8.5957 | 0.484 | D | O |
| H9KV31 | Neural cell adhesion molecule 2 OS=Homo sapiens GN=NCAM2 PE=1 SV=2 | 91066 | 5.2939 | 0.334 | D | O |
| I3L0A0 | HCG2044781 OS=Homo sapiens GN=TMEM189-UBE2V1 PE=4 SV=1 | 42181 | 6.249 | 0.001 | D | O |
| I3L0K7 | Heat shock protein 75 kDa, mitochondrial OS=Homo sapiens GN=TRAP1 PE=1 SV=1 | 57183 | 7.3989 | 0.100 | D | O |
| I3L0N3 | Vesicle-fusing ATPase OS=Homo sapiens GN=NSF PE=1 SV=1 | 82039 | 6.3735 | 1.085 | D | O |
| I3L397 | Eukaryotic translation initiation factor 5A (Fragment) OS=Homo sapiens GN=EIF5A PE=1 SV=6 | 16008 | 4.6567 | 0.314 | D | O |
| I3L4C2 | Brain-specific angiogenesis inhibitor 1-associated protein 2 OS=Homo sapiens GN=BAIAP2 PE | 61342 | 9.2329 | 0.001 | D | O |
| I6L894 | Ankyrin-2 OS=Homo sapiens GN=ANK2 PE=1 SV=1 | 429989 | 4.8413 | 6.900 | D | O |
| J3KN75 | TBC1 domain family member 8B OS=Homo sapiens GN=TBC1D8B PE=1 SV=1 | 128026 | 5.6074 | 0.352 | D | O |
| J3KPX7 | Prohibitin-2 OS=Homo sapiens GN=PHB2 PE=1 SV=2 | 33218 | 10.1895 | 0.502 | D | O |
| J3KQ32 | Obg-like ATPase 1 OS=Homo sapiens GN=OLA1 PE=1 SV=1 | 46908 | 8.1753 | 0.001 | D | O |
| J3KR73 | Ras-related protein Rab-6B (Fragment) OS=Homo sapiens GN=RAB6B PE=1 SV=1 | 14320 | 4.4019 | 0.248 | D | O |
| J3KRF5 | Clathrin heavy chain 1 (Fragment) OS=Homo sapiens GN=CLTC PE=1 SV=1 | 15590 | 4.9526 | 0.357 | D | O |
| J3KTL2 | Serine/arginine-rich-splicing factor 1 OS=Homo sapiens GN=SRSF1 PE=1 SV=1 | 28311 | 10.2861 | 0.001 | D | O |
| J3QLC9 | Haptoglobin (Fragment) OS=Homo sapiens GN=HP PE=1 SV=1 | 40754 | 5.436 | 0.067 | D | O |
| J3QRS3 | Myosin regulatory light chain 12A OS=Homo sapiens GN=MYL12A PE=1 SV=1 | 20443 | 4.4048 | 0.001 | D | O |
| J3QSU6 | Tenascin OS=Homo sapiens GN=TNC PE=1 SV=1 | 220710 | 4.6274 | 0.050 | D | O |
| J3QT27 | Poly(rC)-binding protein 3 (Fragment) OS=Homo sapiens GN=PCBP3 PE=1 SV=1 | 34363 | 7.7461 | 0.025 | D | O |
| J3QTA6 | MICOS complex subunit MIC25 OS=Homo sapiens GN=CHCHD6 PE=1 SV=1 | 28799 | 9.7939 | 0.001 | D | O |
| J9JID7 | Lamin B2, isoform CRA_a OS=Homo sapiens GN=LMNB2 PE=1 SV=1 | 69905 | 5.3364 | 0.257 | D | O |
| K7EK07 | Histone H3 (Fragment) OS=Homo sapiens GN=H3F3B PE=1 SV=1 | 14905 | 11.7026 | 1.904 | D | O |
| K7EKU3 | FXYD domain-containing ion transport regulator 7 OS=Homo sapiens GN=FXYD7 PE=1 SV=1 | 12638 | 8.0845 | 0.001 | D | O |
| K7ELW0 | Protein deglycase DJ-1 OS=Homo sapiens GN=PARK7 PE=1 SV=1 | 17898 | 7.9937 | 1.674 | D | O |
| K7EMU7 | Peptidyl-prolyl cis-trans isomerase NIMA-interacting 1 OS=Homo sapiens GN=PIN1 PE=1 SV=1 | 16121 | 9.4863 | 0.001 | D | O |
| K7EQG4 | Phospholemman (Fragment) OS=Homo sapiens GN=FXYD1 PE=1 SV=2 | 12886 | 9.9126 | 0.001 | D | O |
| K7ER15 | Haloacid dehalogenase-like hydrolase domain-containing protein 2 (Fragment) OS=Homo sapie | 22475 | 6.5215 | 0.089 | D | O |
| K7ERI8 | Uncharacterized protein OS=Homo sapiens PE=3 SV=1 | 17841 | 9.7559 | 0.144 | D | O |
| K7N7A8 | Uncharacterized protein (Fragment) OS=Homo sapiens PE=3 SV=2 | 48766 | 4.8501 | 0.001 | D | O |
| M0QYS1 | 60S ribosomal protein L13a (Fragment) OS=Homo sapiens GN=RPL13A PE=1 SV=2 | 24200 | 11.269 | 0.001 | D | O |
| M0QYZ2 | AP-2 complex subunit sigma OS=Homo sapiens GN=AP2S1 PE=1 SV=1 | 18916 | 7.834 | 0.001 | D | O |
| M0R0Y2 | Alpha-soluble NSF attachment protein OS=Homo sapiens GN=NAPA PE=1 SV=1 | 29144 | 4.8955 | 0.208 | D | O |
| M0R1H5 | 40S ribosomal protein S11 OS=Homo sapiens GN=RPS11 PE=1 SV=1 | 9480 | 11.0698 | 0.064 | D | O |
| M0R2G0 | Leucine-rich repeat-containing protein 4B (Fragment) OS=Homo sapiens GN=LRRC4B PE=1 SV | 26129 | 8.7979 | 4.522 | D | O |
| M0R389 | Platelet-activating factor acetylhydrolase IB subunit gamma (Fragment) OS=Homo sapiens GN= | 18344 | 6.561 | 0.095 | D | O |
| O00148 | ATP-dependent RNA helicase DDX39A OS=Homo sapiens GN=DDX39A PE=1 SV=2 | 49098 | 5.3291 | 0.022 | D | O |
| O00154 | Cytosolic acyl coenzyme A thioester hydrolase OS=Homo sapiens GN=ACOT7 PE=1 SV=3 | 41769 | 8.5444 | 0.529 | D | O |

| | | | | | | |
|---|---|---|---|---|---|---|
| O00231 | 26S proteasome non-ATPase regulatory subunit 11 OS=Homo sapiens GN=PSMD11 PE=1 SV= | 47434 | 6.0469 | 0.072 | D | O |
| O00264 | Membrane-associated progesterone receptor component 1 OS=Homo sapiens GN=PGRMC1 P | 21657 | 4.3433 | 0.182 | D | O |
| O00330 | Pyruvate dehydrogenase protein X component, mitochondrial OS=Homo sapiens GN=PDHX PE | 54088 | 9.0073 | 0.087 | D | O |
| O00429 | Dynamin-1-like protein OS=Homo sapiens GN=DNM1L PE=1 SV=2 | 81826 | 6.3633 | 0.232 | D | O |
| O00483 | Cytochrome c oxidase subunit NDUFA4 OS=Homo sapiens GN=NDUFA4 PE=1 SV=1 | 9363 | 9.75 | 1.414 | D | O |
| O00499 | Myc box-dependent-interacting protein 1 OS=Homo sapiens GN=BIN1 PE=1 SV=1 | 64659 | 4.7886 | 0.138 | D | O |
| O14531 | Dihydropyrimidinase-related protein 4 OS=Homo sapiens GN=DPYSL4 PE=1 SV=2 | 61838 | 6.6592 | 0.121 | D | O |
| O14548 | Cytochrome c oxidase subunit 7A-related protein, mitochondrial OS=Homo sapiens GN=COX7A | 12606 | 9.7031 | 0.092 | D | O |
| O14594 | Neurocan core protein OS=Homo sapiens GN=NCAN PE=1 SV=3 | 143003 | 5.0698 | 0.239 | D | O |
| O14745 | Na(+)/H(+) exchange regulatory cofactor NHE-RF1 OS=Homo sapiens GN=SLC9A3R1 PE=1 S | 38844 | 5.4331 | 0.205 | D | O |
| O14773 | Tripeptidyl-peptidase 1 OS=Homo sapiens GN=TPP1 PE=1 SV=2 | 61209 | 6.0059 | 0.294 | D | O |
| O14818 | Proteasome subunit alpha type-7 OS=Homo sapiens GN=PSMA7 PE=1 SV=1 | 27869 | 8.7114 | 0.098 | D | O |
| O14880 | Microsomal glutathione S-transferase 3 OS=Homo sapiens GN=MGST3 PE=1 SV=1 | 16505 | 9.6021 | 0.091 | D | O |
| O14949 | Cytochrome b-c1 complex subunit 8 OS=Homo sapiens GN=UQCRQ PE=1 SV=4 | 9900 | 10.3462 | 0.415 | D | O |
| O14983 | Sarcoplasmic/endoplasmic reticulum calcium ATPase 1 OS=Homo sapiens GN=ATP2A1 PE=1 | 110181 | 4.8809 | 0.143 | D | O |
| O14994 | Synapsin-3 OS=Homo sapiens GN=SYN3 PE=1 SV=2 | 63262 | 9.6987 | 0.085 | D | O |
| O15020 | Spectrin beta chain, non-erythrocytic 2 OS=Homo sapiens GN=SPTBN2 PE=1 SV=3 | 271155 | 5.7144 | 0.364 | D | O |
| O15075 | Serine/threonine-protein kinase DCLK1 OS=Homo sapiens GN=DCLK1 PE=1 SV=2 | 82172 | 8.7935 | 0.171 | D | O |
| O15078 | Centrosomal protein of 290 kDa OS=Homo sapiens GN=CEP290 PE=1 SV=2 | 290205 | 5.6294 | 0.043 | D | O |
| O15144 | Actin-related protein 2/3 complex subunit 2 OS=Homo sapiens GN=ARPC2 PE=1 SV=1 | 34311 | 6.9785 | 0.190 | D | O |
| O15212 | Prefoldin subunit 6 OS=Homo sapiens GN=PFDN6 PE=1 SV=1 | 14573 | 9.4263 | 0.103 | D | O |
| O15240 | Neurosecretory protein VGF OS=Homo sapiens GN=VGF PE=1 SV=2 | 67217 | 4.5645 | 0.214 | D | O |
| O15400 | Syntaxin-7 OS=Homo sapiens GN=STX7 PE=1 SV=4 | 29797 | 5.2559 | 0.105 | D | O |
| O43175 | D-3-phosphoglycerate dehydrogenase OS=Homo sapiens GN=PHGDH PE=1 SV=4 | 56614 | 6.2798 | 0.001 | D | O |
| O43181 | NADH dehydrogenase [ubiquinone] iron-sulfur protein 4, mitochondrial OS=Homo sapiens GN=I | 20095 | 10.7593 | 0.377 | D | O |
| O43295 | SLIT-ROBO Rho GTPase-activating protein 3 OS=Homo sapiens GN=SRGAP3 PE=1 SV=3 | 124425 | 6.2227 | 0.924 | D | O |
| O43301 | Heat shock 70 kDa protein 12A OS=Homo sapiens GN=HSPA12A PE=1 SV=2 | 74931 | 6.3179 | 0.163 | D | O |
| O43678 | NADH dehydrogenase [ubiquinone] 1 alpha subcomplex subunit 2 OS=Homo sapiens GN=NDU | 10914 | 9.9756 | 0.181 | D | O |
| O43707 | Alpha-actinin-4 OS=Homo sapiens GN=ACTN4 PE=1 SV=2 | 104788 | 5.1211 | 0.041 | D | O |
| O43813 | LanC-like protein 1 OS=Homo sapiens GN=LANCL1 PE=1 SV=1 | 45253 | 7.6201 | 0.150 | D | O |
| O43852 | Calumenin OS=Homo sapiens GN=CALU PE=1 SV=2 | 37083 | 4.2773 | 0.116 | D | O |
| O43865 | Adenosylhomocysteinase 2 OS=Homo sapiens GN=AHCYL1 PE=1 SV=2 | 58913 | 6.4775 | 0.182 | D | O |
| O43920 | NADH dehydrogenase [ubiquinone] iron-sulfur protein 5 OS=Homo sapiens GN=NDUFS5 PE=1 | 12509 | 9.4775 | 0.074 | D | O |
| O60256 | Phosphoribosyl pyrophosphate synthase-associated protein 2 OS=Homo sapiens GN=PRPSAP | 40899 | 7.2012 | 0.263 | D | O |
| O60268 | Uncharacterized protein KIAA0513 OS=Homo sapiens GN=KIAA0513 PE=2 SV=1 | 46609 | 4.7856 | 0.190 | D | O |
| O60361 | Putative nucleoside diphosphate kinase OS=Homo sapiens GN=NME2P1 PE=5 SV=1 | 15518 | 8.8594 | 0.243 | D | O |
| O60506 | Heterogeneous nuclear ribonucleoprotein Q OS=Homo sapiens GN=SYNCRIP PE=1 SV=2 | 69559 | 8.7861 | 0.159 | D | O |
| O60645 | Exocyst complex component 3 OS=Homo sapiens GN=EXOC3 PE=1 SV=2 | 86789 | 5.7129 | 0.265 | D | O |
| O60884 | DnaJ homolog subfamily A member 2 OS=Homo sapiens GN=DNAJA2 PE=1 SV=1 | 45716 | 6.0352 | 0.086 | D | O |
| O75061 | Putative tyrosine-protein phosphatase auxilin OS=Homo sapiens GN=DNAJC6 PE=1 SV=3 | 99933 | 6.8408 | 0.114 | D | O |
| O75083 | WD repeat-containing protein 1 OS=Homo sapiens GN=WDR1 PE=1 SV=4 | 66151 | 6.1772 | 0.334 | D | O |
| O75131 | Copine-3 OS=Homo sapiens GN=CPNE3 PE=1 SV=1 | 60092 | 5.4727 | 0.083 | D | O |
| O75145 | Liprin-alpha-3 OS=Homo sapiens GN=PPFIA3 PE=1 SV=3 | 133413 | 5.3877 | 0.998 | D | O |
| O75223 | Gamma-glutamylcyclotransferase OS=Homo sapiens GN=GGCT PE=1 SV=1 | 20994 | 4.8779 | 0.110 | D | O |
| O75306 | NADH dehydrogenase [ubiquinone] iron-sulfur protein 2, mitochondrial OS=Homo sapiens GN=I | 52511 | 7.3008 | 0.134 | D | O |
| O75323 | Protein NipSnap homolog 2 OS=Homo sapiens GN=GBAS PE=1 SV=1 | 33721 | 9.6211 | 0.278 | D | O |
| O75367 | Core histone macro-H2A.1 OS=Homo sapiens GN=H2AFY PE=1 SV=4 | 39592 | 10.2305 | 0.080 | D | O |
| O75368 | SH3 domain-binding glutamic acid-rich-like protein OS=Homo sapiens GN=SH3BGRL PE=1 SV | 12766 | 5.0215 | 0.214 | D | O |
| O75380 | NADH dehydrogenase [ubiquinone] iron-sulfur protein 6, mitochondrial OS=Homo sapiens GN=I | 13702 | 8.2939 | 0.064 | D | O |
| O75396 | Vesicle-trafficking protein SEC22b OS=Homo sapiens GN=SEC22B PE=1 SV=4 | 24577 | 6.501 | 0.063 | D | O |
| O75489 | NADH dehydrogenase [ubiquinone] iron-sulfur protein 3, mitochondrial OS=Homo sapiens GN=I | 30222 | 7.3623 | 0.353 | D | O |
| O75531 | Barrier-to-autointegration factor OS=Homo sapiens GN=BANF1 PE=1 SV=1 | 10052 | 5.7231 | 0.049 | D | O |
| O75569 | Interferon-inducible double-stranded RNA-dependent protein kinase activator A OS=Homo sapie | 34382 | 8.4536 | 0.074 | D | O |
| O75746 | Calcium-binding mitochondrial carrier protein Aralar1 OS=Homo sapiens GN=SLC25A12 PE=1 | 74714 | 8.4551 | 0.303 | D | O |
| O75781 | Paralemmin-1 OS=Homo sapiens GN=PALM PE=1 SV=2 | 42050 | 4.7432 | 0.249 | D | O |
| O75821 | Eukaryotic translation initiation factor 3 subunit G OS=Homo sapiens GN=EIF3G PE=1 SV=2 | 35588 | 5.7642 | 0.103 | D | O |
| O75915 | PRA1 family protein 3 OS=Homo sapiens GN=ARL6IP5 PE=1 SV=1 | 21600 | 10.1045 | 0.088 | D | O |
| O75947 | ATP synthase subunit d, mitochondrial OS=Homo sapiens GN=ATP5H PE=1 SV=3 | 18479 | 5.0244 | 0.814 | D | O |
| O75955 | Flotillin-1 OS=Homo sapiens GN=FLOT1 PE=1 SV=3 | 47325 | 7.2979 | 0.164 | D | O |
| O75964 | ATP synthase subunit g, mitochondrial OS=Homo sapiens GN=ATP5L PE=1 SV=3 | 11421 | 9.9844 | 0.877 | D | O |
| O76070 | Gamma-synuclein OS=Homo sapiens GN=SNCG PE=1 SV=2 | 13322 | 4.6626 | 0.280 | D | O |
| O94760 | N(G),N(G)-dimethylarginine dimethylaminohydrolase 1 OS=Homo sapiens GN=DDAH1 PE=1 S | 31101 | 5.4287 | 0.511 | D | O |
| O94811 | Tubulin polymerization-promoting protein OS=Homo sapiens GN=TPPP PE=1 SV=1 | 23679 | 9.8892 | 0.565 | D | O |
| O94819 | Kelch repeat and BTB domain-containing protein 11 OS=Homo sapiens GN=KBTBD11 PE=1 S | 65678 | 5.6909 | 0.141 | D | O |
| O94826 | Mitochondrial import receptor subunit TOM70 OS=Homo sapiens GN=TOMM70A PE=1 SV=1 | 67412 | 6.772 | 0.173 | D | O |
| O94856 | Neurofascin OS=Homo sapiens GN=NFASC PE=1 SV=4 | 149933 | 6.208 | 0.366 | D | O |
| O94925 | Glutaminase kidney isoform, mitochondrial OS=Homo sapiens GN=GLS PE=1 SV=1 | 73413 | 7.6011 | 0.455 | D | O |
| O94964 | Protein SOGA1 OS=Homo sapiens GN=SOGA1 PE=1 SV=2 | 159660 | 6.0234 | 0.307 | D | O |
| O94973 | AP-2 complex subunit alpha-2 OS=Homo sapiens GN=AP2A2 PE=1 SV=2 | 103895 | 6.5303 | 0.461 | D | O |
| O95168 | NADH dehydrogenase [ubiquinone] 1 beta subcomplex subunit 4 OS=Homo sapiens GN=NDUF | 15199 | 10.0942 | 0.265 | D | O |
| O95182 | NADH dehydrogenase [ubiquinone] 1 alpha subcomplex subunit 7 OS=Homo sapiens GN=NDU | 12543 | 10.5674 | 0.064 | D | O |
| O95202 | LETM1 and EF-hand domain-containing protein 1, mitochondrial OS=Homo sapiens GN=LETM | 83301 | 6.2695 | 0.136 | D | O |
| O95235 | Kinesin-like protein KIF20A OS=Homo sapiens GN=KIF20A PE=1 SV=1 | 100215 | 6.4805 | 0.049 | D | O |
| O95292 | Vesicle-associated membrane protein-associated protein B/C OS=Homo sapiens GN=VAPB PE | 27211 | 7.1206 | 0.116 | D | O |
| O95294 | RasGAP-activating-like protein 1 OS=Homo sapiens GN=RASAL1 PE=1 SV=3 | 89959 | 6.0601 | 0.068 | D | O |
| O95502 | Neuronal pentraxin receptor OS=Homo sapiens GN=NPTXR PE=3 SV=2 | 52813 | 5.7744 | 0.309 | D | O |
| O95568 | Histidine protein methyltransferase 1 homolog OS=Homo sapiens GN=METTL18 PE=1 SV=1 | 42121 | 6.2959 | 0.690 | D | O |
| O95613 | Pericentrin OS=Homo sapiens GN=PCNT PE=1 SV=4 | 377803 | 5.2588 | 0.396 | D | O |
| O95716 | Ras-related protein Rab-3D OS=Homo sapiens GN=RAB3D PE=1 SV=1 | 24251 | 4.5674 | 0.695 | D | O |
| O95741 | Copine-6 OS=Homo sapiens GN=CPNE6 PE=1 SV=3 | 61951 | 5.168 | 0.222 | D | O |
| O95782 | AP-2 complex subunit alpha-1 OS=Homo sapiens GN=AP2A1 PE=1 SV=3 | 107477 | 6.6255 | 0.453 | D | O |

| | | | | | | |
|---|---|---|---|---|---|---|
| O95817 | BAG family molecular chaperone regulator 3 OS=Homo sapiens GN=BAG3 PE=1 SV=3 | 61556 | 6.4775 | 0.046 | D | O |
| O95865 | N(G),N(G)-dimethylarginine dimethylaminohydrolase 2 OS=Homo sapiens GN=DDAH2 PE=1 S | 29625 | 5.5898 | 0.089 | D | O |
| O95989 | Diphosphoinositol polyphosphate phosphohydrolase 1 OS=Homo sapiens GN=NUDT3 PE=1 S\ | 19458 | 5.9487 | 0.177 | D | O |
| P00338 | L-lactate dehydrogenase A chain OS=Homo sapiens GN=LDHA PE=1 SV=2 | 36665 | 8.3657 | 0.901 | D | O |
| P00352 | Retinal dehydrogenase 1 OS=Homo sapiens GN=ALDH1A1 PE=1 SV=2 | 54826 | 6.2886 | 0.189 | D | O |
| P00367 | Glutamate dehydrogenase 1, mitochondrial OS=Homo sapiens GN=GLUD1 PE=1 SV=2 | 61359 | 7.7021 | 1.113 | D | O |
| P00387 | NADH-cytochrome b5 reductase 3 OS=Homo sapiens GN=CYB5R3 PE=1 SV=3 | 34212 | 7.3872 | 0.368 | D | O |
| P00390 | Glutathione reductase, mitochondrial OS=Homo sapiens GN=GSR PE=1 SV=2 | 56220 | 8.5386 | 0.117 | D | O |
| P00403 | Cytochrome c oxidase subunit 2 OS=Homo sapiens GN=MT-CO2 PE=1 SV=1 | 25548 | 4.4839 | 1.016 | D | O |
| P00441 | Superoxide dismutase [Cu-Zn] OS=Homo sapiens GN=SOD1 PE=1 SV=2 | 15925 | 5.666 | 0.931 | D | O |
| P00505 | Aspartate aminotransferase, mitochondrial OS=Homo sapiens GN=GOT2 PE=1 SV=3 | 47487 | 9.2637 | 1.447 | D | O |
| P00558 | Phosphoglycerate kinase 1 OS=Homo sapiens GN=PGK1 PE=1 SV=3 | 44586 | 8.1475 | 1.000 | D | O |
| P00568 | Adenylate kinase isoenzyme 1 OS=Homo sapiens GN=AK1 PE=1 SV=3 | 21621 | 8.9897 | 0.467 | D | O |
| P00846 | ATP synthase subunit a OS=Homo sapiens GN=MT-ATP6 PE=1 SV=1 | 24800 | 10.4634 | 0.823 | D | O |
| P00918 | Carbonic anhydrase 2 OS=Homo sapiens GN=CA2 PE=1 SV=2 | 29227 | 7.0269 | 0.322 | D | O |
| P00966 | Argininosuccinate synthase OS=Homo sapiens GN=ASS1 PE=1 SV=2 | 46501 | 8.0566 | 0.001 | D | O |
| P01009 | Alpha-1-antitrypsin OS=Homo sapiens GN=SERPINA1 PE=1 SV=3 | 46707 | 5.2412 | 0.289 | D | O |
| P01034 | Cystatin-C OS=Homo sapiens GN=CST3 PE=1 SV=1 | 15789 | 8.9854 | 0.184 | D | O |
| P01112 | GTPase HRas OS=Homo sapiens GN=HRAS PE=1 SV=1 | 21284 | 4.9805 | 0.042 | D | O |
| P01116 | GTPase KRas OS=Homo sapiens GN=KRAS PE=1 SV=1 | 21642 | 6.3677 | 0.093 | D | O |
| P01730 | T-cell surface glycoprotein CD4 OS=Homo sapiens GN=CD4 PE=1 SV=1 | 51078 | 9.9844 | 0.759 | D | O |
| P01876 | Ig alpha-1 chain C region OS=Homo sapiens GN=IGHA1 PE=1 SV=2 | 37630 | 6.0571 | 0.144 | D | O |
| P02008 | Hemoglobin subunit zeta OS=Homo sapiens GN=HBZ PE=1 SV=2 | 15627 | 8.6514 | 0.030 | D | O |
| P02042 | Hemoglobin subunit delta OS=Homo sapiens GN=HBD PE=1 SV=2 | 16045 | 8.2397 | 0.575 | D | O |
| P02533 | Keratin, type I cytoskeletal 14 OS=Homo sapiens GN=KRT14 PE=1 SV=4 | 51529 | 4.8984 | 0.741 | D | O |
| P02545 | Prelamin-A/C OS=Homo sapiens GN=LMNA PE=1 SV=1 | 74094 | 6.5815 | 0.216 | D | O |
| P02647 | Apolipoprotein A-I OS=Homo sapiens GN=APOA1 PE=1 SV=1 | 30758 | 5.4316 | 0.156 | D | O |
| P02686 | Myelin basic protein OS=Homo sapiens GN=MBP PE=1 SV=3 | 33097 | 10.2217 | 1.624 | D | O |
| P02768 | Serum albumin OS=Homo sapiens GN=ALB PE=1 SV=2 | 69321 | 5.8608 | 3.588 | D | O |
| P02769 | Serum albumin OS=Bos taurus GN=ALB PE=1 SV=4 | 69248 | 5.7583 | 0.815 | D | O |
| P02787 | Serotransferrin OS=Homo sapiens GN=TF PE=1 SV=3 | 77013 | 6.7515 | 0.072 | D | O |
| P04075 | Fructose-bisphosphate aldolase A OS=Homo sapiens GN=ALDOA PE=1 SV=2 | 39395 | 8.0654 | 2.687 | D | O |
| P04080 | Cystatin-B OS=Homo sapiens GN=CSTB PE=1 SV=2 | 11132 | 7.5073 | 0.593 | D | O |
| P04259 | Keratin, type II cytoskeletal 6B OS=Homo sapiens GN=KRT6B PE=1 SV=5 | 60030 | 8.0537 | 0.009 | D | O |
| P04264 | Keratin, type II cytoskeletal 1 OS=Homo sapiens GN=KRT1 PE=1 SV=6 | 65998 | 8.2661 | 0.195 | D | O |
| P04271 | Protein S100-B OS=Homo sapiens GN=S100B PE=1 SV=2 | 10706 | 4.3257 | 0.831 | D | O |
| P04350 | Tubulin beta-4A chain OS=Homo sapiens GN=TUBB4A PE=1 SV=2 | 49553 | 4.5908 | 0.892 | D | O |
| P04406 | Glyceraldehyde-3-phosphate dehydrogenase OS=Homo sapiens GN=GAPDH PE=1 SV=3 | 36030 | 8.6968 | 6.002 | D | O |
| P04792 | Heat shock protein beta-1 OS=Homo sapiens GN=HSPB1 PE=1 SV=2 | 22768 | 5.959 | 0.474 | D | O |
| P04843 | Dolichyl-diphosphooligosaccharide--protein glycosyltransferase subunit 1 OS=Homo sapiens GN | 68526 | 5.9268 | 0.053 | D | O |
| P04844 | Dolichyl-diphosphooligosaccharide--protein glycosyltransferase subunit 2 OS=Homo sapiens GN | 69241 | 5.332 | 0.001 | D | O |
| P04899 | Guanine nucleotide-binding protein G(i) subunit alpha-2 OS=Homo sapiens GN=GNAI2 PE=1 S | 40425 | 5.1899 | 0.490 | D | O |
| P05023 | Sodium/potassium-transporting ATPase subunit alpha-1 OS=Homo sapiens GN=ATP1A1 PE=1 | 112824 | 5.1694 | 2.543 | D | O |
| P05026 | Sodium/potassium-transporting ATPase subunit beta-1 OS=Homo sapiens GN=ATP1B1 PE=1 S | 35038 | 8.6484 | 2.036 | D | O |
| P05060 | Secretogranin-1 OS=Homo sapiens GN=CHGB PE=1 SV=2 | 78229 | 4.8354 | 0.114 | D | O |
| P05062 | Fructose-bisphosphate aldolase B OS=Homo sapiens GN=ALDOB PE=1 SV=2 | 39448 | 7.793 | 0.014 | D | O |
| P05091 | Aldehyde dehydrogenase, mitochondrial OS=Homo sapiens GN=ALDH2 PE=1 SV=2 | 56345 | 6.6694 | 0.676 | D | O |
| P05141 | ADP/ATP translocase 2 OS=Homo sapiens GN=SLC25A5 PE=1 SV=7 | 32831 | 9.9917 | 0.498 | D | O |
| P05165 | Propionyl-CoA carboxylase alpha chain, mitochondrial OS=Homo sapiens GN=PCCA PE=1 SV= | 80008 | 7.2524 | 0.078 | D | O |
| P05386 | 60S acidic ribosomal protein P1 OS=Homo sapiens GN=RPLP1 PE=1 SV=1 | 11506 | 4.0005 | 0.069 | D | O |
| P05388 | 60S acidic ribosomal protein P0 OS=Homo sapiens GN=RPLP0 PE=1 SV=1 | 34251 | 5.606 | 0.329 | D | O |
| P05413 | Fatty acid-binding protein, heart OS=Homo sapiens GN=FABP3 PE=1 SV=4 | 14848 | 6.3574 | 0.001 | D | O |
| P05455 | Lupus La protein OS=Homo sapiens GN=SSB PE=1 SV=2 | 46808 | 6.7529 | 0.026 | D | O |
| P06241 | Tyrosine-protein kinase Fyn OS=Homo sapiens GN=FYN PE=1 SV=3 | 60723 | 6.2197 | 0.056 | D | O |
| P06576 | ATP synthase subunit beta, mitochondrial OS=Homo sapiens GN=ATP5B PE=1 SV=3 | 56524 | 5.0962 | 1.972 | D | O |
| P06733 | Alpha-enolase OS=Homo sapiens GN=ENO1 PE=1 SV=2 | 47139 | 7.1719 | 2.576 | D | O |
| P06748 | Nucleophosmin OS=Homo sapiens GN=NPM1 PE=1 SV=2 | 32554 | 4.4443 | 0.121 | D | O |
| P07099 | Epoxide hydrolase 1 OS=Homo sapiens GN=EPHX1 PE=1 SV=1 | 52914 | 6.8364 | 0.163 | D | O |
| P07195 | L-lactate dehydrogenase B chain OS=Homo sapiens GN=LDHB PE=1 SV=2 | 36615 | 5.6396 | 1.174 | D | O |
| P07196 | Neurofilament light polypeptide OS=Homo sapiens GN=NEFL PE=1 SV=3 | 61479 | 4.4326 | 0.894 | D | O |
| P07237 | Protein disulfide-isomerase OS=Homo sapiens GN=P4HB PE=1 SV=3 | 57080 | 4.5645 | 0.167 | D | O |
| P07339 | Cathepsin D OS=Homo sapiens GN=CTSD PE=1 SV=1 | 44523 | 6.0908 | 1.223 | D | O |
| P07437 | Tubulin beta chain OS=Homo sapiens GN=TUBB PE=1 SV=2 | 49638 | 4.5908 | 1.626 | D | O |
| P07737 | Profilin-1 OS=Homo sapiens GN=PFN1 PE=1 SV=2 | 15044 | 8.4595 | 0.616 | D | O |
| P07814 | Bifunctional glutamate/proline--tRNA ligase OS=Homo sapiens GN=EPRS PE=1 SV=5 | 170482 | 6.9756 | 0.099 | D | O |
| P07858 | Cathepsin B OS=Homo sapiens GN=CTSB PE=1 SV=3 | 37796 | 5.855 | 0.218 | D | O |
| P07900 | Heat shock protein HSP 90-alpha OS=Homo sapiens GN=HSP90AA1 PE=1 SV=5 | 84606 | 4.7476 | 0.866 | D | O |
| P07919 | Cytochrome b-c1 complex subunit 6, mitochondrial OS=Homo sapiens GN=UQCRH PE=1 SV=2 | 10731 | 4.1865 | 0.134 | D | O |
| P07948 | Tyrosine-protein kinase Lyn OS=Homo sapiens GN=LYN PE=1 SV=3 | 58536 | 6.75 | 0.008 | D | O |
| P07954 | Fumarate hydratase, mitochondrial OS=Homo sapiens GN=FH PE=1 SV=3 | 54602 | 9.0835 | 0.522 | D | O |
| P08133 | Annexin A6 OS=Homo sapiens GN=ANXA6 PE=1 SV=3 | 75825 | 5.2646 | 0.817 | D | O |
| P08134 | Rho-related GTP-binding protein RhoC OS=Homo sapiens GN=RHOC PE=1 SV=1 | 21992 | 6.189 | 0.001 | D | O |
| P08237 | ATP-dependent 6-phosphofructokinase, muscle type OS=Homo sapiens GN=PFKM PE=1 SV=2 | 85128 | 7.8926 | 0.283 | D | O |
| P08238 | Heat shock protein HSP 90-beta OS=Homo sapiens GN=HSP90AB1 PE=1 SV=4 | 83212 | 4.7739 | 0.565 | D | O |
| P08247 | Synaptophysin OS=Homo sapiens GN=SYP PE=1 SV=3 | 33823 | 4.4561 | 0.359 | D | O |
| P08559 | Pyruvate dehydrogenase E1 component subunit alpha, somatic form, mitochondrial OS=Homo s | 43267 | 7.9995 | 0.987 | D | O |
| P08572 | Collagen alpha-2(IV) chain OS=Homo sapiens GN=COL4A2 PE=1 SV=4 | 167448 | 8.7378 | 0.148 | D | O |
| P08574 | Cytochrome c1, heme protein, mitochondrial OS=Homo sapiens GN=CYC1 PE=1 SV=3 | 35398 | 9.1831 | 0.346 | D | O |
| P08670 | Vimentin OS=Homo sapiens GN=VIM PE=1 SV=4 | 53619 | 4.8633 | 0.774 | D | O |
| P08727 | Keratin, type I cytoskeletal 19 OS=Homo sapiens GN=KRT19 PE=1 SV=4 | 44079 | 4.8604 | 0.707 | D | O |
| P08754 | Guanine nucleotide-binding protein G(k) subunit alpha OS=Homo sapiens GN=GNAI3 PE=1 SV | 40506 | 5.3584 | 0.350 | D | O |

| | | | | | | |
|---|---|---|---|---|---|---|
| P08758 | Annexin A5 OS=Homo sapiens GN=ANXA5 PE=1 SV=2 | 35914 | 4.7329 | 0.368 | D | O |
| P09104 | Gamma-enolase OS=Homo sapiens GN=ENO2 PE=1 SV=3 | 47239 | 4.7183 | 0.980 | D | O |
| P09211 | Glutathione S-transferase P OS=Homo sapiens GN=GSTP1 PE=1 SV=2 | 23341 | 5.2822 | 0.553 | D | O |
| P09238 | Stromelysin-2 OS=Homo sapiens GN=MMP10 PE=1 SV=1 | 54116 | 5.3965 | 0.145 | D | O |
| P09382 | Galectin-1 OS=Homo sapiens GN=LGALS1 PE=1 SV=2 | 14706 | 5.1416 | 0.596 | D | O |
| P09417 | Dihydropteridine reductase OS=Homo sapiens GN=QDPR PE=1 SV=2 | 25773 | 7.1719 | 0.232 | D | O |
| P09471 | Guanine nucleotide-binding protein G(o) subunit alpha OS=Homo sapiens GN=GNAO1 PE=1 S | 40024 | 5.1899 | 1.631 | D | O |
| P09496 | Clathrin light chain A OS=Homo sapiens GN=CLTA PE=1 SV=1 | 27060 | 4.2349 | 0.203 | D | O |
| P09497 | Clathrin light chain B OS=Homo sapiens GN=CLTB PE=1 SV=1 | 25175 | 4.3726 | 0.287 | D | O |
| P09543 | 2',3'-cyclic-nucleotide 3'-phosphodiesterase OS=Homo sapiens GN=CNP PE=1 SV=2 | 47548 | 9.3618 | 0.882 | D | O |
| P09669 | Cytochrome c oxidase subunit 6C OS=Homo sapiens GN=COX6C PE=1 SV=2 | 8775 | 10.752 | 0.542 | D | O |
| P09936 | Ubiquitin carboxyl-terminal hydrolase isozyme L1 OS=Homo sapiens GN=UCHL1 PE=1 SV=2 | 24808 | 5.1841 | 2.311 | D | O |
| P09972 | Fructose-bisphosphate aldolase C OS=Homo sapiens GN=ALDOC PE=1 SV=2 | 39431 | 6.4351 | 1.271 | D | O |
| P0DMV8 | Heat shock 70 kDa protein 1A OS=Homo sapiens GN=HSPA1A PE=1 SV=1 | 70009 | 5.3188 | 0.697 | D | O |
| P10114 | Ras-related protein Rap-2a OS=Homo sapiens GN=RAP2A PE=1 SV=1 | 20602 | 4.5278 | 0.020 | D | O |
| P10599 | Thioredoxin OS=Homo sapiens GN=TXN PE=1 SV=3 | 11729 | 4.6201 | 0.507 | D | O |
| P10606 | Cytochrome c oxidase subunit 5B, mitochondrial OS=Homo sapiens GN=COX5B PE=1 SV=2 | 13686 | 9.0688 | 0.779 | D | O |
| P10768 | S-formylglutathione hydrolase OS=Homo sapiens GN=ESD PE=1 SV=2 | 31442 | 6.5815 | 0.001 | D | O |
| P10809 | 60 kDa heat shock protein, mitochondrial OS=Homo sapiens GN=HSPD1 PE=1 SV=2 | 61016 | 5.5503 | 0.840 | D | O |
| P10909 | Clusterin OS=Homo sapiens GN=CLU PE=1 SV=1 | 52461 | 5.8389 | 0.206 | D | O |
| P10915 | Hyaluronan and proteoglycan link protein 1 OS=Homo sapiens GN=HAPLN1 PE=2 SV=2 | 40139 | 7.1396 | 0.134 | D | O |
| P11021 | 78 kDa glucose-regulated protein OS=Homo sapiens GN=HSPA5 PE=1 SV=2 | 72288 | 4.875 | 1.333 | D | O |
| P11137 | Microtubule-associated protein 2 OS=Homo sapiens GN=MAP2 PE=1 SV=4 | 199402 | 4.6318 | 1.048 | D | O |
| P11142 | Heat shock cognate 71 kDa protein OS=Homo sapiens GN=HSPA8 PE=1 SV=1 | 70854 | 5.2002 | 2.502 | D | O |
| P11169 | Solute carrier family 2, facilitated glucose transporter member 3 OS=Homo sapiens GN=SLC2A | 53889 | 6.9683 | 0.001 | D | O |
| P11177 | Pyruvate dehydrogenase E1 component subunit beta, mitochondrial OS=Homo sapiens GN=PD | 39208 | 6.2021 | 0.671 | D | O |
| P11216 | Glycogen phosphorylase, brain form OS=Homo sapiens GN=PYGB PE=1 SV=5 | 96634 | 6.3999 | 0.385 | D | O |
| P11217 | Glycogen phosphorylase, muscle form OS=Homo sapiens GN=PYGM PE=1 SV=6 | 97030 | 6.583 | 0.462 | D | O |
| P11233 | Ras-related protein Ral-A OS=Homo sapiens GN=RALA PE=1 SV=1 | 23551 | 6.9858 | 0.172 | D | O |
| P11274 | Breakpoint cluster region protein OS=Homo sapiens GN=BCR PE=1 SV=2 | 142729 | 6.6064 | 0.154 | D | O |
| P11277 | Spectrin beta chain, erythrocytic OS=Homo sapiens GN=SPTB PE=1 SV=5 | 246313 | 4.9761 | 0.255 | D | O |
| P12036 | Neurofilament heavy polypeptide OS=Homo sapiens GN=NEFH PE=1 SV=4 | 112410 | 5.874 | 0.112 | D | O |
| P12236 | ADP/ATP translocase 3 OS=Homo sapiens GN=SLC25A6 PE=1 SV=4 | 32845 | 10.062 | 0.959 | D | O |
| P12277 | Creatine kinase B-type OS=Homo sapiens GN=CKB PE=1 SV=1 | 42617 | 5.2178 | 3.694 | D | O |
| P12532 | Creatine kinase U-type, mitochondrial OS=Homo sapiens GN=CKMT1A PE=1 SV=1 | 47007 | 8.3628 | 0.743 | D | O |
| P12814 | Alpha-actinin-1 OS=Homo sapiens GN=ACTN1 PE=1 SV=2 | 102992 | 5.0933 | 0.187 | D | O |
| P12956 | X-ray repair cross-complementing protein 6 OS=Homo sapiens GN=XRCC6 PE=1 SV=2 | 69799 | 6.2036 | 0.196 | D | O |
| P13010 | X-ray repair cross-complementing protein 5 OS=Homo sapiens GN=XRCC5 PE=1 SV=3 | 82652 | 5.436 | 0.083 | D | O |
| P13073 | Cytochrome c oxidase subunit 4 isoform 1, mitochondrial OS=Homo sapiens GN=COX4I1 PE=1 | 19564 | 9.9155 | 0.861 | D | O |
| P13521 | Secretogranin-2 OS=Homo sapiens GN=SCG2 PE=1 SV=2 | 70897 | 4.478 | 0.581 | D | O |
| P13611 | Versican core protein OS=Homo sapiens GN=VCAN PE=1 SV=3 | 372588 | 4.2349 | 1.900 | D | O |
| P13639 | Elongation factor 2 OS=Homo sapiens GN=EEF2 PE=1 SV=4 | 95277 | 6.394 | 0.227 | D | O |
| P13645 | Keratin, type I cytoskeletal 10 OS=Homo sapiens GN=KRT10 PE=1 SV=6 | 58791 | 4.9556 | 0.406 | D | O |
| P13861 | cAMP-dependent protein kinase type II-alpha regulatory subunit OS=Homo sapiens GN=PRKAF | 45489 | 4.771 | 0.213 | D | O |
| P13987 | CD59 glycoprotein OS=Homo sapiens GN=CD59 PE=1 SV=1 | 14167 | 6.0337 | 0.231 | D | O |
| P14136 | Glial fibrillary acidic protein OS=Homo sapiens GN=GFAP PE=1 SV=1 | 49849 | 5.2559 | 27.647 | D | O |
| P14174 | Macrophage migration inhibitory factor OS=Homo sapiens GN=MIF PE=1 SV=4 | 12468 | 7.9922 | 0.640 | D | O |
| P14415 | Sodium/potassium-transporting ATPase subunit beta-2 OS=Homo sapiens GN=ATP1B2 PE=1 S | 33345 | 8.3438 | 0.489 | D | O |
| P14550 | Alcohol dehydrogenase [NADP(+)] OS=Homo sapiens GN=AKR1A1 PE=1 SV=3 | 36549 | 6.3384 | 0.033 | D | O |
| P14618 | Pyruvate kinase PKM OS=Homo sapiens GN=PKM PE=1 SV=4 | 57900 | 7.7534 | 2.482 | D | O |
| P14621 | Acylphosphatase-2 OS=Homo sapiens GN=ACYP2 PE=1 SV=2 | 11132 | 9.8701 | 0.322 | D | O |
| P14625 | Endoplasmin OS=Homo sapiens GN=HSP90B1 PE=1 SV=1 | 92411 | 4.5645 | 0.453 | D | O |
| P14854 | Cytochrome c oxidase subunit 6B1 OS=Homo sapiens GN=COX6B1 PE=1 SV=2 | 10185 | 6.8657 | 0.555 | D | O |
| P14868 | Aspartate--tRNA ligase, cytoplasmic OS=Homo sapiens GN=DARS PE=1 SV=2 | 57100 | 6.0894 | 0.186 | D | O |
| P14927 | Cytochrome b-c1 complex subunit 7 OS=Homo sapiens GN=UQCRB PE=1 SV=2 | 13521 | 9.2446 | 0.598 | D | O |
| P15104 | Glutamine synthetase OS=Homo sapiens GN=GLUL PE=1 SV=4 | 42037 | 6.4424 | 0.171 | D | O |
| P15121 | Aldose reductase OS=Homo sapiens GN=AKR1B1 PE=1 SV=3 | 35830 | 6.5508 | 0.149 | D | O |
| P15531 | Nucleoside diphosphate kinase A OS=Homo sapiens GN=NME1 PE=1 SV=1 | 17137 | 5.7671 | 0.859 | D | O |
| P15954 | Cytochrome c oxidase subunit 7C, mitochondrial OS=Homo sapiens GN=COX7C PE=1 SV=1 | 7240 | 10.6626 | 0.307 | D | O |
| P16104 | Histone H2AX OS=Homo sapiens GN=H2AFX PE=1 SV=2 | 15135 | 11.1563 | 0.001 | D | O |
| P16152 | Carbonyl reductase [NADPH] 1 OS=Homo sapiens GN=CBR1 PE=1 SV=3 | 30355 | 8.417 | 0.644 | D | O |
| P16298 | Serine/threonine-protein phosphatase 2B catalytic subunit beta isoform OS=Homo sapiens GN= | 58986 | 5.5166 | 0.029 | D | O |
| P16402 | Histone H1.3 OS=Homo sapiens GN=HIST1H1D PE=1 SV=2 | 22336 | 11.5122 | 0.452 | D | O |
| P16403 | Histone H1.2 OS=Homo sapiens GN=HIST1H1C PE=1 SV=2 | 21351 | 11.4287 | 0.232 | D | O |
| P16615 | Sarcoplasmic/endoplasmic reticulum calcium ATPase 2 OS=Homo sapiens GN=ATP2A2 PE=1 | 114682 | 5.0522 | 0.223 | D | O |
| P16870 | Carboxypeptidase E OS=Homo sapiens GN=CPE PE=1 SV=1 | 53117 | 4.8472 | 0.090 | D | O |
| P17066 | Heat shock 70 kDa protein 6 OS=Homo sapiens GN=HSPA6 PE=1 SV=2 | 70984 | 5.7319 | 0.810 | D | O |
| P17174 | Aspartate aminotransferase, cytoplasmic OS=Homo sapiens GN=GOT1 PE=1 SV=3 | 46218 | 6.5698 | 0.320 | D | O |
| P17252 | Protein kinase C alpha type OS=Homo sapiens GN=PRKCA PE=1 SV=4 | 76700 | 6.6138 | 0.121 | D | O |
| P17302 | Gap junction alpha-1 protein OS=Homo sapiens GN=GJA1 PE=1 SV=2 | 42980 | 8.8887 | 0.170 | D | O |
| P17540 | Creatine kinase S-type, mitochondrial OS=Homo sapiens GN=CKMT2 PE=1 SV=2 | 47474 | 8.1782 | 0.076 | D | O |
| P17600 | Synapsin-1 OS=Homo sapiens GN=SYN1 PE=1 SV=3 | 74065 | 10.1895 | 3.444 | D | O |
| P17612 | cAMP-dependent protein kinase catalytic subunit alpha OS=Homo sapiens GN=PRKACA PE=1 | 40564 | 9.126 | 0.017 | D | O |
| P17661 | Desmin OS=Homo sapiens GN=DES PE=1 SV=3 | 53503 | 5.0303 | 0.024 | D | O |
| P17677 | Neuromodulin OS=Homo sapiens GN=GAP43 PE=1 SV=1 | 24787 | 4.4473 | 0.386 | D | O |
| P17844 | Probable ATP-dependent RNA helicase DDX5 OS=Homo sapiens GN=DDX5 PE=1 SV=1 | 69104 | 9.0996 | 0.008 | D | O |
| P17858 | ATP-dependent 6-phosphofructokinase, liver type OS=Homo sapiens GN=PFKL PE=1 SV=6 | 84964 | 7.2261 | 0.083 | D | O |
| P17987 | T-complex protein 1 subunit alpha OS=Homo sapiens GN=TCP1 PE=1 SV=1 | 60305 | 5.7129 | 0.245 | D | O |
| P18124 | 60S ribosomal protein L7 OS=Homo sapiens GN=RPL7 PE=1 SV=1 | 29207 | 11.0654 | 0.216 | D | O |
| P18669 | Phosphoglycerate mutase 1 OS=Homo sapiens GN=PGAM1 PE=1 SV=2 | 28785 | 6.7866 | 1.034 | D | O |
| P18859 | ATP synthase-coupling factor 6, mitochondrial OS=Homo sapiens GN=ATP5J PE=1 SV=1 | 12579 | 9.9829 | 0.706 | D | O |

| | | | | | | |
|---|---|---|---|---|---|---|
| P19013 | Keratin, type II cytoskeletal 4 OS=Homo sapiens GN=KRT4 PE=1 SV=4 | 57249 | 6.2153 | 0.017 | D | O |
| P19021 | Peptidyl-glycine alpha-amidating monooxygenase OS=Homo sapiens GN=PAM PE=1 SV=2 | 108263 | 5.9575 | 0.113 | D | O |
| P19086 | Guanine nucleotide-binding protein G(z) subunit alpha OS=Homo sapiens GN=GNAZ PE=2 SV= | 40897 | 7.4912 | 0.121 | D | O |
| P19087 | Guanine nucleotide-binding protein G(t) subunit alpha-2 OS=Homo sapiens GN=GNAT2 PE=2 S | 40150 | 4.9263 | 0.018 | D | O |
| P19338 | Nucleolin OS=Homo sapiens GN=NCL PE=1 SV=3 | 76568 | 4.4004 | 0.226 | D | O |
| P19367 | Hexokinase-1 OS=Homo sapiens GN=HK1 PE=1 SV=3 | 102420 | 6.3472 | 0.943 | D | O |
| P20020 | Plasma membrane calcium-transporting ATPase 1 OS=Homo sapiens GN=ATP2B1 PE=1 SV=3 | 138667 | 5.644 | 0.240 | D | O |
| P20073 | Annexin A7 OS=Homo sapiens GN=ANXA7 PE=1 SV=3 | 52705 | 5.3525 | 0.116 | D | O |
| P20336 | Ras-related protein Rab-3A OS=Homo sapiens GN=RAB3A PE=1 SV=1 | 24968 | 4.6626 | 1.755 | D | O |
| P20337 | Ras-related protein Rab-3B OS=Homo sapiens GN=RAB3B PE=1 SV=2 | 24742 | 4.6538 | 0.115 | D | O |
| P20338 | Ras-related protein Rab-4A OS=Homo sapiens GN=RAB4A PE=1 SV=3 | 24374 | 5.7231 | 0.217 | D | O |
| P20339 | Ras-related protein Rab-5A OS=Homo sapiens GN=RAB5A PE=1 SV=2 | 23643 | 8.2515 | 0.186 | D | O |
| P20340 | Ras-related protein Rab-6A OS=Homo sapiens GN=RAB6A PE=1 SV=3 | 23577 | 5.2266 | 0.215 | D | O |
| P20648 | Potassium-transporting ATPase alpha chain 1 OS=Homo sapiens GN=ATP4A PE=2 SV=5 | 114045 | 5.4521 | 0.016 | D | O |
| P20674 | Cytochrome c oxidase subunit 5A, mitochondrial OS=Homo sapiens GN=COX5A PE=1 SV=2 | 16751 | 6.3574 | 0.624 | D | O |
| P20916 | Myelin-associated glycoprotein OS=Homo sapiens GN=MAG PE=1 SV=1 | 69024 | 4.7871 | 0.047 | D | O |
| P21266 | Glutathione S-transferase Mu 3 OS=Homo sapiens GN=GSTM3 PE=1 SV=3 | 26542 | 5.2075 | 0.212 | D | O |
| P21281 | V-type proton ATPase subunit B, brain isoform OS=Homo sapiens GN=ATP6V1B2 PE=1 SV=3 | 56464 | 5.4492 | 0.854 | D | O |
| P21283 | V-type proton ATPase subunit C 1 OS=Homo sapiens GN=ATP6V1C1 PE=1 SV=4 | 43914 | 7.3037 | 0.023 | D | O |
| P21291 | Cysteine and glycine-rich protein 1 OS=Homo sapiens GN=CSRP1 PE=1 SV=3 | 20553 | 8.5532 | 0.228 | D | O |
| P21579 | Synaptotagmin-1 OS=Homo sapiens GN=SYT1 PE=1 SV=1 | 47542 | 8.2061 | 0.772 | D | O |
| P21796 | Voltage-dependent anion-selective channel protein 1 OS=Homo sapiens GN=VDAC1 PE=1 SV= | 30753 | 8.8682 | 2.871 | D | O |
| P21802 | Fibroblast growth factor receptor 2 OS=Homo sapiens GN=FGFR2 PE=1 SV=1 | 91965 | 5.5034 | 0.047 | D | O |
| P21912 | Succinate dehydrogenase [ubiquinone] iron-sulfur subunit, mitochondrial OS=Homo sapiens GN | 31608 | 8.8125 | 0.189 | D | O |
| P22314 | Ubiquitin-like modifier-activating enzyme 1 OS=Homo sapiens GN=UBA1 PE=1 SV=3 | 117774 | 5.3789 | 0.385 | D | O |
| P22626 | Heterogeneous nuclear ribonucleoproteins A2/B1 OS=Homo sapiens GN=HNRNPA2B1 PE=1 S | 37406 | 9.1948 | 1.288 | D | O |
| P23246 | Splicing factor, proline- and glutamine-rich OS=Homo sapiens GN=SFPQ PE=1 SV=2 | 76101 | 9.772 | 0.150 | D | O |
| P23284 | Peptidyl-prolyl cis-trans isomerase B OS=Homo sapiens GN=PPIB PE=1 SV=2 | 23727 | 9.8511 | 0.257 | D | O |
| P23297 | Protein S100-A1 OS=Homo sapiens GN=S100A1 PE=1 SV=2 | 10539 | 4.1865 | 0.992 | D | O |
| P23381 | Tryptophan--tRNA ligase, cytoplasmic OS=Homo sapiens GN=WARS PE=1 SV=2 | 53131 | 5.7803 | 0.138 | D | O |
| P23471 | Receptor-type tyrosine-protein phosphatase zeta OS=Homo sapiens GN=PTPRZ1 PE=1 SV=4 | 254427 | 4.5718 | 0.626 | D | O |
| P23634 | Plasma membrane calcium-transporting ATPase 4 OS=Homo sapiens GN=ATP2B4 PE=1 SV=2 | 137832 | 6.1597 | 0.115 | D | O |
| P24534 | Elongation factor 1-beta OS=Homo sapiens GN=EEF1B2 PE=1 SV=3 | 24748 | 4.3037 | 0.127 | D | O |
| P24539 | ATP synthase F(0) complex subunit B1, mitochondrial OS=Homo sapiens GN=ATP5F1 PE=1 S | 28890 | 9.6563 | 0.655 | D | O |
| P24752 | Acetyl-CoA acetyltransferase, mitochondrial OS=Homo sapiens GN=ACAT1 PE=1 SV=1 | 45170 | 9.1201 | 0.331 | D | O |
| P25398 | 40S ribosomal protein S12 OS=Homo sapiens GN=RPS12 PE=1 SV=3 | 14505 | 6.9844 | 0.169 | D | O |
| P25705 | ATP synthase subunit alpha, mitochondrial OS=Homo sapiens GN=ATP5A1 PE=1 SV=1 | 59713 | 9.4321 | 3.082 | D | O |
| P26038 | Moesin OS=Homo sapiens GN=MSN PE=1 SV=3 | 67777 | 6.0103 | 0.218 | D | O |
| P26196 | Probable ATP-dependent RNA helicase DDX6 OS=Homo sapiens GN=DDX6 PE=1 SV=2 | 54382 | 8.8359 | 0.075 | D | O |
| P26232 | Catenin alpha-2 OS=Homo sapiens GN=CTNNA2 PE=1 SV=5 | 105246 | 5.3745 | 0.136 | D | O |
| P26641 | Elongation factor 1-gamma OS=Homo sapiens GN=EEF1G PE=1 SV=3 | 50087 | 6.2358 | 0.451 | D | O |
| P26885 | Peptidyl-prolyl cis-trans isomerase FKBP2 OS=Homo sapiens GN=FKBP2 PE=1 SV=2 | 15639 | 9.5522 | 0.105 | D | O |
| P27105 | Erythrocyte band 7 integral membrane protein OS=Homo sapiens GN=STOM PE=1 SV=3 | 31710 | 7.9878 | 0.085 | D | O |
| P27338 | Amine oxidase [flavin-containing] B OS=Homo sapiens GN=MAOB PE=1 SV=3 | 58725 | 7.248 | 0.750 | D | O |
| P27348 | 14-3-3 protein theta OS=Homo sapiens GN=YWHAQ PE=1 SV=1 | 27746 | 4.4854 | 0.564 | D | O |
| P27361 | Mitogen-activated protein kinase 3 OS=Homo sapiens GN=MAPK3 PE=1 SV=4 | 43108 | 6.2886 | 0.151 | D | O |
| P27482 | Calmodulin-like protein 3 OS=Homo sapiens GN=CALML3 PE=1 SV=2 | 16879 | 4.0972 | 1.356 | D | O |
| P27797 | Calreticulin OS=Homo sapiens GN=CALR PE=1 SV=1 | 48111 | 4.0942 | 0.407 | D | O |
| P27824 | Calnexin OS=Homo sapiens GN=CANX PE=1 SV=2 | 67525 | 4.2686 | 0.406 | D | O |
| P28066 | Proteasome subunit alpha type-5 OS=Homo sapiens GN=PSMA5 PE=1 SV=3 | 26394 | 4.5439 | 0.143 | D | O |
| P28072 | Proteasome subunit beta type-6 OS=Homo sapiens GN=PSMB6 PE=1 SV=4 | 25341 | 4.6069 | 0.073 | D | O |
| P28161 | Glutathione S-transferase Mu 2 OS=Homo sapiens GN=GSTM2 PE=1 SV=2 | 25727 | 5.9531 | 0.334 | D | O |
| P28331 | NADH-ubiquinone oxidoreductase 75 kDa subunit, mitochondrial OS=Homo sapiens GN=NDUF | 79416 | 5.8184 | 0.423 | D | O |
| P28482 | Mitogen-activated protein kinase 1 OS=Homo sapiens GN=MAPK1 PE=1 SV=3 | 41363 | 6.5317 | 0.553 | D | O |
| P28838 | Cytosol aminopeptidase OS=Homo sapiens GN=LAP3 PE=1 SV=3 | 56130 | 7.9072 | 0.171 | D | O |
| P29120 | Neuroendocrine convertase 1 OS=Homo sapiens GN=PCSK1 PE=1 SV=2 | 84099 | 5.5752 | 0.051 | D | O |
| P29401 | Transketolase OS=Homo sapiens GN=TKT PE=1 SV=3 | 67834 | 7.478 | 0.451 | D | O |
| P29992 | Guanine nucleotide-binding protein subunit alpha-11 OS=Homo sapiens GN=GNA11 PE=1 SV= | 42096 | 5.3687 | 0.054 | D | O |
| P30038 | Delta-1-pyrroline-5-carboxylate dehydrogenase, mitochondrial OS=Homo sapiens GN=ALDH4A | 61680 | 8.0566 | 0.195 | D | O |
| P30040 | Endoplasmic reticulum resident protein 29 OS=Homo sapiens GN=ERP29 PE=1 SV=4 | 28975 | 7.2832 | 0.228 | D | O |
| P30041 | Peroxiredoxin-6 OS=Homo sapiens GN=PRDX6 PE=1 SV=3 | 25019 | 5.9575 | 1.240 | D | O |
| P30043 | Flavin reductase (NADPH) OS=Homo sapiens GN=BLVRB PE=1 SV=3 | 22105 | 7.4927 | 0.215 | D | O |
| P30044 | Peroxiredoxin-5, mitochondrial OS=Homo sapiens GN=PRDX5 PE=1 SV=4 | 22072 | 8.9839 | 0.791 | D | O |
| P30048 | Thioredoxin-dependent peroxide reductase, mitochondrial OS=Homo sapiens GN=PRDX3 PE=1 | 27675 | 7.6934 | 0.730 | D | O |
| P30049 | ATP synthase subunit delta, mitochondrial OS=Homo sapiens GN=ATP5D PE=1 SV=2 | 17479 | 5.1914 | 1.005 | D | O |
| P30050 | 60S ribosomal protein L12 OS=Homo sapiens GN=RPL12 PE=1 SV=1 | 17807 | 9.8965 | 0.200 | D | O |
| P30084 | Enoyl-CoA hydratase, mitochondrial OS=Homo sapiens GN=ECHS1 PE=1 SV=4 | 31367 | 8.0728 | 0.298 | D | O |
| P30085 | UMP-CMP kinase OS=Homo sapiens GN=CMPK1 PE=1 SV=3 | 22208 | 5.2646 | 0.139 | D | O |
| P30086 | Phosphatidylethanolamine-binding protein 1 OS=Homo sapiens GN=PEBP1 PE=1 SV=3 | 21043 | 7.3901 | 1.492 | D | O |
| P30101 | Protein disulfide-isomerase A3 OS=Homo sapiens GN=PDIA3 PE=1 SV=4 | 56746 | 5.9312 | 0.421 | D | O |
| P30153 | Serine/threonine-protein phosphatase 2A 65 kDa regulatory subunit A alpha isoform OS=Homo | 65267 | 4.8149 | 0.229 | D | O |
| P30405 | Peptidyl-prolyl cis-trans isomerase F, mitochondrial OS=Homo sapiens GN=PPIF PE=1 SV=1 | 22026 | 9.7734 | 1.169 | D | O |
| P30613 | Pyruvate kinase PKLR OS=Homo sapiens GN=PKLR PE=1 SV=2 | 61791 | 7.6348 | 0.952 | D | O |
| P31146 | Coronin-1A OS=Homo sapiens GN=CORO1A PE=1 SV=4 | 50993 | 6.2417 | 0.222 | D | O |
| P31150 | Rab GDP dissociation inhibitor alpha OS=Homo sapiens GN=GDI1 PE=1 SV=2 | 50550 | 4.8135 | 0.950 | D | O |
| P31323 | cAMP-dependent protein kinase type II-beta regulatory subunit OS=Homo sapiens GN=PRKAR2 | 46272 | 4.6318 | 0.023 | D | O |
| P31689 | DnaJ homolog subfamily A member 1 OS=Homo sapiens GN=DNAJA1 PE=1 SV=2 | 44839 | 6.6709 | 0.070 | D | O |
| P31930 | Cytochrome b-c1 complex subunit 1, mitochondrial OS=Homo sapiens GN=UQCRC1 PE=1 SV= | 52612 | 5.9092 | 0.653 | D | O |
| P31937 | 3-hydroxyisobutyrate dehydrogenase, mitochondrial OS=Homo sapiens GN=HIBADH PE=1 SV= | 35305 | 8.1475 | 0.022 | D | O |
| P31939 | Bifunctional purine biosynthesis protein PURH OS=Homo sapiens GN=ATIC PE=1 SV=3 | 64575 | 6.2622 | 0.056 | D | O |
| P31942 | Heterogeneous nuclear ribonucleoprotein H3 OS=Homo sapiens GN=HNRNPH3 PE=1 SV=2 | 36903 | 6.3999 | 0.221 | D | O |

| | | | | | | |
|---|---|---|---|---|---|---|
| P31946 | 14-3-3 protein beta/alpha OS=Homo sapiens GN=YWHAB PE=1 SV=3 | 28064 | 4.5674 | 3.481 | D | O |
| P31947 | 14-3-3 protein sigma OS=Homo sapiens GN=SFN PE=1 SV=1 | 27756 | 4.4824 | 0.273 | D | O |
| P31948 | Stress-induced-phosphoprotein 1 OS=Homo sapiens GN=STIP1 PE=1 SV=1 | 62599 | 6.3867 | 0.218 | D | O |
| P32119 | Peroxiredoxin-2 OS=Homo sapiens GN=PRDX2 PE=1 SV=5 | 21878 | 5.5679 | 1.690 | D | O |
| P33778 | Histone H2B type 1-B OS=Homo sapiens GN=HIST1H2BB PE=1 SV=2 | 13941 | 10.7402 | 0.300 | D | O |
| P34931 | Heat shock 70 kDa protein 1-like OS=Homo sapiens GN=HSPA1L PE=1 SV=2 | 70331 | 5.6455 | 0.547 | D | O |
| P34932 | Heat shock 70 kDa protein 4 OS=Homo sapiens GN=HSPA4 PE=1 SV=4 | 94271 | 4.9131 | 0.119 | D | O |
| P35232 | Prohibitin OS=Homo sapiens GN=PHB PE=1 SV=1 | 29785 | 5.4302 | 0.994 | D | O |
| P35241 | Radixin OS=Homo sapiens GN=RDX PE=1 SV=1 | 68521 | 5.9692 | 0.306 | D | O |
| P35527 | Keratin, type I cytoskeletal 9 OS=Homo sapiens GN=KRT9 PE=1 SV=3 | 62026 | 4.9585 | 0.144 | D | O |
| P35579 | Myosin-9 OS=Homo sapiens GN=MYH9 PE=1 SV=4 | 226390 | 5.3364 | 0.209 | D | O |
| P35580 | Myosin-10 OS=Homo sapiens GN=MYH10 PE=1 SV=3 | 228856 | 5.272 | 0.126 | D | O |
| P35609 | Alpha-actinin-2 OS=Homo sapiens GN=ACTN2 PE=1 SV=1 | 103788 | 5.1533 | 0.115 | D | O |
| P35612 | Beta-adducin OS=Homo sapiens GN=ADD2 PE=1 SV=3 | 80803 | 5.5635 | 0.254 | D | O |
| P35613 | Basigin OS=Homo sapiens GN=BSG PE=1 SV=2 | 42174 | 5.2822 | 0.157 | D | O |
| P35749 | Myosin-11 OS=Homo sapiens GN=MYH11 PE=1 SV=3 | 227197 | 5.25 | 0.107 | D | O |
| P35908 | Keratin, type II cytoskeletal 2 epidermal OS=Homo sapiens GN=KRT2 PE=1 SV=2 | 65393 | 8.0537 | 0.016 | D | O |
| P36542 | ATP synthase subunit gamma, mitochondrial OS=Homo sapiens GN=ATP5C1 PE=1 SV=1 | 32975 | 9.561 | 1.283 | D | O |
| P36543 | V-type proton ATPase subunit E 1 OS=Homo sapiens GN=ATP6V1E1 PE=1 SV=1 | 26128 | 8.4419 | 0.640 | D | O |
| P36578 | 60S ribosomal protein L4 OS=Homo sapiens GN=RPL4 PE=1 SV=5 | 47667 | 11.4961 | 0.165 | D | O |
| P36871 | Phosphoglucomutase-1 OS=Homo sapiens GN=PGM1 PE=1 SV=3 | 61410 | 6.3032 | 0.111 | D | O |
| P36957 | Dihydrolipoyllysine-residue succinyltransferase component of 2-oxoglutarate dehydrogenase co | 48724 | 9.2769 | 0.586 | D | O |
| P37235 | Hippocalcin-like protein 1 OS=Homo sapiens GN=HPCAL1 PE=1 SV=3 | 22298 | 5.0303 | 0.036 | D | O |
| P37837 | Transaldolase OS=Homo sapiens GN=TALDO1 PE=1 SV=2 | 37516 | 6.375 | 0.082 | D | O |
| P38117 | Electron transfer flavoprotein subunit beta OS=Homo sapiens GN=ETFB PE=1 SV=3 | 27826 | 8.2456 | 0.257 | D | O |
| P38159 | RNA-binding motif protein, X chromosome OS=Homo sapiens GN=RBMX PE=1 SV=3 | 42306 | 10.2437 | 0.236 | D | O |
| P38606 | V-type proton ATPase catalytic subunit A OS=Homo sapiens GN=ATP6V1A PE=1 SV=2 | 68260 | 5.1855 | 0.737 | D | O |
| P38646 | Stress-70 protein, mitochondrial OS=Homo sapiens GN=HSPA9 PE=1 SV=2 | 73634 | 5.7803 | 0.860 | D | O |
| P38919 | Eukaryotic initiation factor 4A-III OS=Homo sapiens GN=EIF4A3 PE=1 SV=4 | 46841 | 6.2944 | 0.029 | D | O |
| P39019 | 40S ribosomal protein S19 OS=Homo sapiens GN=RPS19 PE=1 SV=2 | 16050 | 10.7329 | 0.169 | D | O |
| P39023 | 60S ribosomal protein L3 OS=Homo sapiens GN=RPL3 PE=1 SV=2 | 46079 | 10.6187 | 0.183 | D | O |
| P40121 | Macrophage-capping protein OS=Homo sapiens GN=CAPG PE=1 SV=2 | 38474 | 5.7715 | 0.045 | D | O |
| P40123 | Adenylyl cyclase-associated protein 2 OS=Homo sapiens GN=CAP2 PE=1 SV=1 | 52790 | 5.9253 | 0.388 | D | O |
| P40227 | T-complex protein 1 subunit zeta OS=Homo sapiens GN=CCT6A PE=1 SV=3 | 57987 | 6.2241 | 0.293 | D | O |
| P40925 | Malate dehydrogenase, cytoplasmic OS=Homo sapiens GN=MDH1 PE=1 SV=4 | 36403 | 7.1704 | 0.997 | D | O |
| P40926 | Malate dehydrogenase, mitochondrial OS=Homo sapiens GN=MDH2 PE=1 SV=3 | 35480 | 8.8213 | 2.897 | D | O |
| P40939 | Trifunctional enzyme subunit alpha, mitochondrial OS=Homo sapiens GN=HADHA PE=1 SV=2 | 82946 | 9.3413 | 0.155 | D | O |
| P41219 | Peripherin OS=Homo sapiens GN=PRPH PE=1 SV=2 | 53618 | 5.209 | 0.018 | D | O |
| P41222 | Prostaglandin-H2 D-isomerase OS=Homo sapiens GN=PTGDS PE=1 SV=1 | 21015 | 7.8325 | 0.248 | D | O |
| P41250 | Glycine--tRNA ligase OS=Homo sapiens GN=GARS PE=1 SV=3 | 83112 | 6.6138 | 0.254 | D | O |
| P42025 | Beta-centractin OS=Homo sapiens GN=ACTR1B PE=1 SV=1 | 42266 | 5.9546 | 0.128 | D | O |
| P42166 | Lamina-associated polypeptide 2, isoform alpha OS=Homo sapiens GN=TMPO PE=1 SV=2 | 75445 | 7.5132 | 0.051 | D | O |
| P42262 | Glutamate receptor 2 OS=Homo sapiens GN=GRIA2 PE=1 SV=3 | 98758 | 7.4341 | 0.070 | D | O |
| P43004 | Excitatory amino acid transporter 2 OS=Homo sapiens GN=SLC1A2 PE=1 SV=2 | 62063 | 6.0762 | 1.532 | D | O |
| P43007 | Neutral amino acid transporter A OS=Homo sapiens GN=SLC1A4 PE=1 SV=1 | 55687 | 5.8315 | 0.112 | D | O |
| P43034 | Platelet-activating factor acetylhydrolase IB subunit alpha OS=Homo sapiens GN=PAFAH1B1 P | 46608 | 7.0151 | 0.113 | D | O |
| P43304 | Glycerol-3-phosphate dehydrogenase, mitochondrial OS=Homo sapiens GN=GPD2 PE=1 SV=3 | 80801 | 7.5454 | 0.226 | D | O |
| P45880 | Voltage-dependent anion-selective channel protein 2 OS=Homo sapiens GN=VDAC2 PE=1 SV= | 31546 | 7.4678 | 1.819 | D | O |
| P46783 | 40S ribosomal protein S10 OS=Homo sapiens GN=RPS10 PE=1 SV=1 | 18885 | 10.5088 | 0.292 | D | O |
| P46821 | Microtubule-associated protein 1B OS=Homo sapiens GN=MAP1B PE=1 SV=2 | 270465 | 4.5381 | 0.587 | D | O |
| P47755 | F-actin-capping protein subunit alpha-2 OS=Homo sapiens GN=CAPZA2 PE=1 SV=3 | 32928 | 5.4756 | 0.292 | D | O |
| P47914 | 60S ribosomal protein L29 OS=Homo sapiens GN=RPL29 PE=1 SV=2 | 17741 | 12.0791 | 0.067 | D | O |
| P47985 | Cytochrome b-c1 complex subunit Rieske, mitochondrial OS=Homo sapiens GN=UQCRFS1 PE | 29649 | 8.3936 | 0.510 | D | O |
| P48047 | ATP synthase subunit O, mitochondrial OS=Homo sapiens GN=ATP5O PE=1 SV=1 | 23262 | 10.3638 | 0.985 | D | O |
| P48735 | Isocitrate dehydrogenase [NADP], mitochondrial OS=Homo sapiens GN=IDH2 PE=1 SV=2 | 50876 | 8.855 | 0.592 | D | O |
| P49006 | MARCKS-related protein OS=Homo sapiens GN=MARCKSL1 PE=1 SV=2 | 19517 | 4.4531 | 0.071 | D | O |
| P49189 | 4-trimethylaminobutyraldehyde dehydrogenase OS=Homo sapiens GN=ALDH9A1 PE=1 SV=3 | 53767 | 5.562 | 0.351 | D | O |
| P49411 | Elongation factor Tu, mitochondrial OS=Homo sapiens GN=TUFM PE=1 SV=2 | 49510 | 7.3726 | 0.639 | D | O |
| P49418 | Amphiphysin OS=Homo sapiens GN=AMPH PE=1 SV=1 | 76210 | 4.374 | 0.357 | D | O |
| P49419 | Alpha-aminoadipic semialdehyde dehydrogenase OS=Homo sapiens GN=ALDH7A1 PE=1 SV= | 58450 | 7.938 | 0.338 | D | O |
| P49721 | Proteasome subunit beta type-2 OS=Homo sapiens GN=PSMB2 PE=1 SV=1 | 22821 | 6.6079 | 0.466 | D | O |
| P49748 | Very long-chain specific acyl-CoA dehydrogenase, mitochondrial OS=Homo sapiens GN=ACAD | 70345 | 8.9912 | 0.054 | D | O |
| P50148 | Guanine nucleotide-binding protein G(q) subunit alpha OS=Homo sapiens GN=GNAQ PE=1 SV | 42115 | 5.3408 | 0.113 | D | O |
| P50150 | Guanine nucleotide-binding protein G(I)/G(S)/G(O) subunit gamma-4 OS=Homo sapiens GN=G | 8383 | 6.9375 | 0.071 | D | O |
| P50213 | Isocitrate dehydrogenase [NAD] subunit alpha, mitochondrial OS=Homo sapiens GN=IDH3A PE | 39566 | 6.4907 | 0.830 | D | O |
| P50395 | Rab GDP dissociation inhibitor beta OS=Homo sapiens GN=GDI2 PE=1 SV=2 | 50630 | 6.0557 | 0.392 | D | O |
| P50570 | Dynamin-2 OS=Homo sapiens GN=DNM2 PE=1 SV=2 | 98003 | 7.1162 | 0.029 | D | O |
| P50990 | T-complex protein 1 subunit theta OS=Homo sapiens GN=CCT8 PE=1 SV=4 | 59582 | 5.272 | 0.456 | D | O |
| P50991 | T-complex protein 1 subunit delta OS=Homo sapiens GN=CCT4 PE=1 SV=4 | 57887 | 7.7827 | 0.210 | D | O |
| P50995 | Annexin A11 OS=Homo sapiens GN=ANXA11 PE=1 SV=1 | 54355 | 7.6143 | 0.076 | D | O |
| P51149 | Ras-related protein Rab-7a OS=Homo sapiens GN=RAB7A PE=1 SV=1 | 23474 | 6.5742 | 0.587 | D | O |
| P51153 | Ras-related protein Rab-13 OS=Homo sapiens GN=RAB13 PE=1 SV=1 | 22759 | 9.5698 | 0.059 | D | O |
| P51452 | Dual specificity protein phosphatase 3 OS=Homo sapiens GN=DUSP3 PE=1 SV=1 | 20465 | 7.8325 | 0.070 | D | O |
| P51649 | Succinate-semialdehyde dehydrogenase, mitochondrial OS=Homo sapiens GN=ALDH5A1 PE= | 57178 | 8.2324 | 0.621 | D | O |
| P51665 | 26S proteasome non-ATPase regulatory subunit 7 OS=Homo sapiens GN=PSMD7 PE=1 SV=2 | 37002 | 6.3003 | 1.584 | D | O |
| P51674 | Neuronal membrane glycoprotein M6-a OS=Homo sapiens GN=GPM6A PE=1 SV=2 | 31188 | 5.0024 | 0.818 | D | O |
| P51970 | NADH dehydrogenase [ubiquinone] 1 alpha subcomplex subunit 8 OS=Homo sapiens GN=NDU | 20092 | 7.5483 | 0.194 | D | O |
| P51991 | Heterogeneous nuclear ribonucleoprotein A3 OS=Homo sapiens GN=HNRNPA3 PE=1 SV=2 | 39570 | 9.2212 | 0.559 | D | O |
| P52209 | 6-phosphogluconate dehydrogenase, decarboxylating OS=Homo sapiens GN=PGD PE=1 SV=3 | 53105 | 6.8584 | 0.263 | D | O |
| P52565 | Rho GDP-dissociation inhibitor 1 OS=Homo sapiens GN=ARHGDIA PE=1 SV=3 | 23192 | 4.8179 | 0.367 | D | O |
| P52758 | Ribonuclease UK114 OS=Homo sapiens GN=HRSP12 PE=1 SV=1 | 14484 | 9.1289 | 0.258 | D | O |

| | | | | | | |
|---|---|---|---|---|---|---|
| P52789 | Hexokinase-2 OS=Homo sapiens GN=HK2 PE=1 SV=2 | 102313 | 5.6367 | 0.249 | D | O |
| P52790 | Hexokinase-3 OS=Homo sapiens GN=HK3 PE=1 SV=2 | 98962 | 5.0771 | 0.078 | D | O |
| P52907 | F-actin-capping protein subunit alpha-1 OS=Homo sapiens GN=CAPZA1 PE=1 SV=3 | 32902 | 5.3262 | 0.086 | D | O |
| P53396 | ATP-citrate synthase OS=Homo sapiens GN=ACLY PE=1 SV=3 | 120762 | 6.9507 | 0.083 | D | O |
| P53675 | Clathrin heavy chain 2 OS=Homo sapiens GN=CLTCL1 PE=1 SV=2 | 186909 | 5.4741 | 0.088 | D | O |
| P53999 | Activated RNA polymerase II transcriptional coactivator p15 OS=Homo sapiens GN=SUB1 PE= | 14386 | 10.0737 | 0.412 | D | O |
| P54289 | Voltage-dependent calcium channel subunit alpha-2/delta-1 OS=Homo sapiens GN=CACNA2D | 124489 | 4.9365 | 0.125 | D | O |
| P54652 | Heat shock-related 70 kDa protein 2 OS=Homo sapiens GN=HSPA2 PE=1 SV=1 | 69977 | 5.4082 | 1.192 | D | O |
| P54707 | Potassium-transporting ATPase alpha chain 2 OS=Homo sapiens GN=ATP12A PE=1 SV=3 | 115437 | 6.0981 | 0.453 | D | O |
| P55072 | Transitional endoplasmic reticulum ATPase OS=Homo sapiens GN=VCP PE=1 SV=4 | 89265 | 4.9556 | 0.351 | D | O |
| P55084 | Trifunctional enzyme subunit beta, mitochondrial OS=Homo sapiens GN=HADHB PE=1 SV=3 | 51261 | 9.75 | 0.294 | D | O |
| P55087 | Aquaporin-4 OS=Homo sapiens GN=AQP4 PE=1 SV=2 | 34806 | 7.5557 | 0.001 | D | O |
| P55327 | Tumor protein D52 OS=Homo sapiens GN=TPD52 PE=1 SV=2 | 24312 | 4.5908 | 0.113 | D | O |
| P55786 | Puromycin-sensitive aminopeptidase OS=Homo sapiens GN=NPEPPS PE=1 SV=2 | 103210 | 5.3599 | 0.232 | D | O |
| P55809 | Succinyl-CoA:3-ketoacid coenzyme A transferase 1, mitochondrial OS=Homo sapiens GN=OXC | 56121 | 7.2202 | 0.194 | D | O |
| P56381 | ATP synthase subunit epsilon, mitochondrial OS=Homo sapiens GN=ATP5E PE=1 SV=2 | 5776 | 10.3154 | 0.001 | D | O |
| P56385 | ATP synthase subunit e, mitochondrial OS=Homo sapiens GN=ATP5I PE=1 SV=2 | 7928 | 9.7412 | 0.883 | D | O |
| P58546 | Myotrophin OS=Homo sapiens GN=MTPN PE=1 SV=2 | 12886 | 5.1343 | 0.424 | D | O |
| P60174 | Triosephosphate isomerase OS=Homo sapiens GN=TPI1 PE=1 SV=3 | 30771 | 5.5474 | 1.432 | D | O |
| P60201 | Myelin proteolipid protein OS=Homo sapiens GN=PLP1 PE=1 SV=2 | 30057 | 8.2881 | 2.072 | D | O |
| P60709 | Actin, cytoplasmic 1 OS=Homo sapiens GN=ACTB PE=1 SV=1 | 41709 | 5.1431 | 6.656 | D | O |
| P60880 | Synaptosomal-associated protein 25 OS=Homo sapiens GN=SNAP25 PE=1 SV=1 | 23300 | 4.4575 | 0.386 | D | O |
| P60953 | Cell division control protein 42 homolog OS=Homo sapiens GN=CDC42 PE=1 SV=2 | 21245 | 6.1538 | 0.712 | D | O |
| P61006 | Ras-related protein Rab-8A OS=Homo sapiens GN=RAB8A PE=1 SV=1 | 23653 | 9.4321 | 0.040 | D | O |
| P61019 | Ras-related protein Rab-2A OS=Homo sapiens GN=RAB2A PE=1 SV=1 | 23530 | 6.0806 | 0.561 | D | O |
| P61020 | Ras-related protein Rab-5B OS=Homo sapiens GN=RAB5B PE=1 SV=1 | 23691 | 8.2383 | 0.151 | D | O |
| P61026 | Ras-related protein Rab-10 OS=Homo sapiens GN=RAB10 PE=1 SV=1 | 22526 | 8.5737 | 0.327 | D | O |
| P61088 | Ubiquitin-conjugating enzyme E2 N OS=Homo sapiens GN=UBE2N PE=1 SV=1 | 17126 | 6.1494 | 0.459 | D | O |
| P61163 | Alpha-centractin OS=Homo sapiens GN=ACTR1A PE=1 SV=1 | 42586 | 6.1846 | 0.163 | D | O |
| P61224 | Ras-related protein Rap-1b OS=Homo sapiens GN=RAP1B PE=1 SV=1 | 20811 | 5.4653 | 0.001 | D | O |
| P61225 | Ras-related protein Rap-2b OS=Homo sapiens GN=RAP2B PE=1 SV=1 | 20491 | 4.5278 | 0.095 | D | O |
| P61266 | Syntaxin-1B OS=Homo sapiens GN=STX1B PE=1 SV=1 | 33223 | 5.0859 | 0.812 | D | O |
| P61604 | 10 kDa heat shock protein, mitochondrial OS=Homo sapiens GN=HSPE1 PE=1 SV=2 | 10924 | 9.4702 | 1.791 | D | O |
| P61764 | Syntaxin-binding protein 1 OS=Homo sapiens GN=STXBP1 PE=1 SV=1 | 67525 | 6.5098 | 0.813 | D | O |
| P61978 | Heterogeneous nuclear ribonucleoprotein K OS=Homo sapiens GN=HNRNPK PE=1 SV=1 | 50944 | 5.2207 | 0.618 | D | O |
| P61981 | 14-3-3 protein gamma OS=Homo sapiens GN=YWHAG PE=1 SV=2 | 28284 | 4.6069 | 2.332 | D | O |
| P62081 | 40S ribosomal protein S7 OS=Homo sapiens GN=RPS7 PE=1 SV=1 | 22113 | 10.582 | 0.195 | D | O |
| P62140 | Serine/threonine-protein phosphatase PP1-beta catalytic subunit OS=Homo sapiens GN=PPP1 | 37162 | 5.7715 | 0.061 | D | O |
| P62166 | Neuronal calcium sensor 1 OS=Homo sapiens GN=NCS1 PE=1 SV=2 | 21864 | 4.5088 | 0.150 | D | O |
| P62241 | 40S ribosomal protein S8 OS=Homo sapiens GN=RPS8 PE=1 SV=2 | 24190 | 10.7153 | 0.001 | D | O |
| P62258 | 14-3-3 protein epsilon OS=Homo sapiens GN=YWHAE PE=1 SV=1 | 29155 | 4.4355 | 4.698 | D | O |
| P62266 | 40S ribosomal protein S23 OS=Homo sapiens GN=RPS23 PE=1 SV=3 | 15797 | 10.9585 | 0.193 | D | O |
| P62269 | 40S ribosomal protein S18 OS=Homo sapiens GN=RPS18 PE=1 SV=3 | 17707 | 11.4141 | 0.174 | D | O |
| P62277 | 40S ribosomal protein S13 OS=Homo sapiens GN=RPS13 PE=1 SV=2 | 17211 | 10.9409 | 0.057 | D | O |
| P62330 | ADP-ribosylation factor 6 OS=Homo sapiens GN=ARF6 PE=1 SV=2 | 20069 | 9.293 | 0.375 | D | O |
| P62701 | 40S ribosomal protein S4, X isoform OS=Homo sapiens GN=RPS4X PE=1 SV=2 | 29579 | 10.5864 | 0.199 | D | O |
| P62714 | Serine/threonine-protein phosphatase 2A catalytic subunit beta isoform OS=Homo sapiens GN= | 35552 | 5.061 | 0.001 | D | O |
| P62805 | Histone H4 OS=Homo sapiens GN=HIST1H4A PE=1 SV=2 | 11360 | 11.7671 | 2.802 | D | O |
| P62851 | 40S ribosomal protein S25 OS=Homo sapiens GN=RPS25 PE=1 SV=1 | 13733 | 10.5762 | 0.190 | D | O |
| P62873 | Guanine nucleotide-binding protein G(I)/G(S)/G(T) subunit beta-1 OS=Homo sapiens GN=GNB | 37353 | 5.5356 | 0.556 | D | O |
| P62879 | Guanine nucleotide-binding protein G(I)/G(S)/G(T) subunit beta-2 OS=Homo sapiens GN=GNB2 | 37307 | 5.5356 | 0.025 | D | O |
| P62937 | Peptidyl-prolyl cis-trans isomerase A OS=Homo sapiens GN=PPIA PE=1 SV=2 | 18000 | 7.853 | 2.169 | D | O |
| P62942 | Peptidyl-prolyl cis-trans isomerase FKBP1A OS=Homo sapiens GN=FKBP1A PE=1 SV=2 | 11943 | 8.6558 | 0.429 | D | O |
| P62993 | Growth factor receptor-bound protein 2 OS=Homo sapiens GN=GRB2 PE=1 SV=1 | 25190 | 5.8579 | 0.198 | D | O |
| P62995 | Transformer-2 protein homolog beta OS=Homo sapiens GN=TRA2B PE=1 SV=1 | 33645 | 11.6177 | 0.049 | D | O |
| P63000 | Ras-related C3 botulinum toxin substrate 1 OS=Homo sapiens GN=RAC1 PE=1 SV=1 | 21436 | 8.5723 | 0.547 | D | O |
| P63010 | AP-2 complex subunit beta OS=Homo sapiens GN=AP2B1 PE=1 SV=1 | 104486 | 5.0552 | 0.582 | D | O |
| P63096 | Guanine nucleotide-binding protein G(i) subunit alpha-1 OS=Homo sapiens GN=GNAI1 PE=1 S | 40335 | 5.5913 | 0.214 | D | O |
| P63267 | Actin, gamma-enteric smooth muscle OS=Homo sapiens GN=ACTG2 PE=1 SV=1 | 41849 | 5.1606 | 0.021 | D | O |
| P67936 | Tropomyosin alpha-4 chain OS=Homo sapiens GN=TPM4 PE=1 SV=3 | 28504 | 4.4707 | 0.393 | D | O |
| P68032 | Actin, alpha cardiac muscle 1 OS=Homo sapiens GN=ACTC1 PE=1 SV=1 | 41991 | 5.0713 | 0.125 | D | O |
| P68036 | Ubiquitin-conjugating enzyme E2 L3 OS=Homo sapiens GN=UBE2L3 PE=1 SV=1 | 17850 | 8.7993 | 0.116 | D | O |
| P68104 | Elongation factor 1-alpha 1 OS=Homo sapiens GN=EEF1A1 PE=1 SV=1 | 50109 | 9.3428 | 0.721 | D | O |
| P68363 | Tubulin alpha-1B chain OS=Homo sapiens GN=TUBA1B PE=1 SV=1 | 50119 | 4.7622 | 0.196 | D | O |
| P68366 | Tubulin alpha-4A chain OS=Homo sapiens GN=TUBA4A PE=1 SV=1 | 49892 | 4.752 | 2.996 | D | O |
| P68371 | Tubulin beta-4B chain OS=Homo sapiens GN=TUBB4B PE=1 SV=1 | 49799 | 4.6025 | 3.450 | D | O |
| P68402 | Platelet-activating factor acetylhydrolase IB subunit beta OS=Homo sapiens GN=PAFAH1B2 PE | 25553 | 5.5005 | 0.131 | D | O |
| P68871 | Hemoglobin subunit beta OS=Homo sapiens GN=HBB PE=1 SV=2 | 15988 | 6.8804 | 5.905 | D | O |
| P69905 | Hemoglobin subunit alpha OS=Homo sapiens GN=HBA1 PE=1 SV=2 | 15247 | 9.1787 | 5.144 | D | O |
| P78324 | Tyrosine-protein phosphatase non-receptor type substrate 1 OS=Homo sapiens GN=SIRPA PE | 54932 | 6.5361 | 0.183 | D | O |
| P78352 | Disks large homolog 4 OS=Homo sapiens GN=DLG4 PE=1 SV=3 | 80445 | 5.4858 | 0.052 | D | O |
| P78357 | Contactin-associated protein 1 OS=Homo sapiens GN=CNTNAP1 PE=1 SV=1 | 156166 | 6.5991 | 0.110 | D | O |
| P78371 | T-complex protein 1 subunit beta OS=Homo sapiens GN=CCT2 PE=1 SV=4 | 57452 | 5.9912 | 0.230 | D | O |
| P78559 | Microtubule-associated protein 1A OS=Homo sapiens GN=MAP1A PE=1 SV=6 | 305296 | 4.6567 | 0.336 | D | O |
| P80723 | Brain acid soluble protein 1 OS=Homo sapiens GN=BASP1 PE=1 SV=2 | 22680 | 4.4238 | 0.562 | D | O |
| P84074 | Neuron-specific calcium-binding protein hippocalcin OS=Homo sapiens GN=HPCA PE=1 SV=2 | 22412 | 4.6758 | 0.673 | D | O |
| P84103 | Serine/arginine-rich splicing factor 3 OS=Homo sapiens GN=SRSF3 PE=1 SV=1 | 19317 | 12.0425 | 0.231 | D | O |
| Q00169 | Phosphatidylinositol transfer protein alpha isoform OS=Homo sapiens GN=PITPNA PE=1 SV=2 | 31786 | 6.0981 | 0.065 | D | O |
| Q00325 | Phosphate carrier protein, mitochondrial OS=Homo sapiens GN=SLC25A3 PE=1 SV=2 | 40068 | 9.6387 | 1.662 | D | O |
| Q00577 | Transcriptional activator protein Pur-alpha OS=Homo sapiens GN=PURA PE=1 SV=2 | 34889 | 6.0249 | 0.421 | D | O |
| Q00610 | Clathrin heavy chain 1 OS=Homo sapiens GN=CLTC PE=1 SV=5 | 191491 | 5.3525 | 0.944 | D | O |

| | | | | | | |
|---|---|---|---|---|---|---|
| Q00839 | Heterogeneous nuclear ribonucleoprotein U OS=Homo sapiens GN=HNRNPU PE=1 SV=6 | 90527 | 5.6484 | 0.309 | D | O |
| Q01082 | Spectrin beta chain, non-erythrocytic 1 OS=Homo sapiens GN=SPTBN1 PE=1 SV=2 | 274437 | 5.2515 | 1.975 | D | O |
| Q01469 | Fatty acid-binding protein, epidermal OS=Homo sapiens GN=FABP5 PE=1 SV=3 | 15154 | 6.8042 | 0.329 | D | O |
| Q01484 | Ankyrin-2 OS=Homo sapiens GN=ANK2 PE=1 SV=4 | 433447 | 4.8516 | 0.434 | D | O |
| Q01518 | Adenylyl cyclase-associated protein 1 OS=Homo sapiens GN=CAP1 PE=1 SV=5 | 51868 | 8.0581 | 0.290 | D | O |
| Q01546 | Keratin, type II cytoskeletal 2 oral OS=Homo sapiens GN=KRT76 PE=1 SV=2 | 65800 | 8.0991 | 0.591 | D | O |
| Q01813 | ATP-dependent 6-phosphofructokinase, platelet type OS=Homo sapiens GN=PFKP PE=1 SV=2 | 85541 | 7.3535 | 0.424 | D | O |
| Q02252 | Methylmalonate-semialdehyde dehydrogenase [acylating], mitochondrial OS=Homo sapiens GN | 57802 | 8.584 | 0.177 | D | O |
| Q02539 | Histone H1.1 OS=Homo sapiens GN=HIST1H1A PE=1 SV=3 | 21828 | 11.4829 | 0.110 | D | O |
| Q02750 | Dual specificity mitogen-activated protein kinase kinase 1 OS=Homo sapiens GN=MAP2K1 PE= | 43411 | 6.1772 | 0.352 | D | O |
| Q02790 | Peptidyl-prolyl cis-trans isomerase FKBP4 OS=Homo sapiens GN=FKBP4 PE=1 SV=3 | 51772 | 5.1812 | 0.024 | D | O |
| Q02818 | Nucleobindin-1 OS=Homo sapiens GN=NUCB1 PE=1 SV=4 | 53846 | 4.9805 | 0.062 | D | O |
| Q02878 | 60S ribosomal protein L6 OS=Homo sapiens GN=RPL6 PE=1 SV=3 | 32707 | 11.0244 | 0.127 | D | O |
| Q02978 | Mitochondrial 2-oxoglutarate/malate carrier protein OS=Homo sapiens GN=SLC25A11 PE=1 SV | 34039 | 10.2085 | 0.629 | D | O |
| Q04760 | Lactoylglutathione lyase OS=Homo sapiens GN=GLO1 PE=1 SV=4 | 20764 | 4.9424 | 0.187 | D | O |
| Q04837 | Single-stranded DNA-binding protein, mitochondrial OS=Homo sapiens GN=SSBP1 PE=1 SV=1 | 17249 | 9.9111 | 0.465 | D | O |
| Q04917 | 14-3-3 protein eta OS=Homo sapiens GN=YWHAH PE=1 SV=4 | 28201 | 4.5615 | 0.849 | D | O |
| Q05639 | Elongation factor 1-alpha 2 OS=Homo sapiens GN=EEF1A2 PE=1 SV=1 | 50438 | 9.3472 | 1.089 | D | O |
| Q06830 | Peroxiredoxin-1 OS=Homo sapiens GN=PRDX1 PE=1 SV=1 | 22096 | 8.2427 | 2.718 | D | O |
| Q07021 | Complement component 1 Q subcomponent-binding protein, mitochondrial OS=Homo sapiens G | 31342 | 4.5469 | 0.590 | D | O |
| Q07065 | Cytoskeleton-associated protein 4 OS=Homo sapiens GN=CKAP4 PE=1 SV=2 | 65982 | 5.5415 | 0.040 | D | O |
| Q07666 | KH domain-containing, RNA-binding, signal transduction-associated protein 1 OS=Homo sapien | 48197 | 8.9136 | 0.100 | D | O |
| Q08043 | Alpha-actinin-3 OS=Homo sapiens GN=ACTN3 PE=1 SV=2 | 103176 | 5.2163 | 0.024 | D | O |
| Q08174 | Protocadherin-1 OS=Homo sapiens GN=PCDH1 PE=1 SV=2 | 114673 | 4.7139 | 0.113 | D | O |
| Q08209 | Serine/threonine-protein phosphatase 2B catalytic subunit alpha isoform OS=Homo sapiens GN | 58650 | 5.4814 | 0.701 | D | O |
| Q08211 | ATP-dependent RNA helicase A OS=Homo sapiens GN=DHX9 PE=1 SV=4 | 140868 | 6.3955 | 0.114 | D | O |
| Q08378 | Golgin subfamily A member 3 OS=Homo sapiens GN=GOLGA3 PE=1 SV=2 | 167251 | 5.1724 | 1.643 | D | O |
| Q08722 | Leukocyte surface antigen CD47 OS=Homo sapiens GN=CD47 PE=1 SV=1 | 35190 | 6.9844 | 0.417 | D | O |
| Q10567 | AP-1 complex subunit beta-1 OS=Homo sapiens GN=AP1B1 PE=1 SV=2 | 104570 | 4.749 | 0.129 | D | O |
| Q12765 | Secernin-1 OS=Homo sapiens GN=SCRN1 PE=1 SV=2 | 46352 | 4.4707 | 0.215 | D | O |
| Q12802 | A-kinase anchor protein 13 OS=Homo sapiens GN=AKAP13 PE=1 SV=2 | 307357 | 4.9438 | 0.957 | D | O |
| Q12860 | Contactin-1 OS=Homo sapiens GN=CNTN1 PE=1 SV=1 | 113249 | 5.5137 | 0.700 | D | O |
| Q12906 | Interleukin enhancer-binding factor 3 OS=Homo sapiens GN=ILF3 PE=1 SV=3 | 95279 | 8.9561 | 0.124 | D | O |
| Q12955 | Ankyrin-3 OS=Homo sapiens GN=ANK3 PE=1 SV=3 | 480112 | 6.0396 | 0.220 | D | O |
| Q13011 | Delta(3,5)-Delta(2,4)-dienoyl-CoA isomerase, mitochondrial OS=Homo sapiens GN=ECH1 PE= | 35793 | 7.9995 | 0.234 | D | O |
| Q13151 | Heterogeneous nuclear ribonucleoprotein A0 OS=Homo sapiens GN=HNRNPA0 PE=1 SV=1 | 30821 | 9.5493 | 0.138 | D | O |
| Q13228 | Selenium-binding protein 1 OS=Homo sapiens GN=SELENBP1 PE=1 SV=2 | 52357 | 5.9063 | 0.134 | D | O |
| Q13347 | Eukaryotic translation initiation factor 3 subunit I OS=Homo sapiens GN=EIF3I PE=1 SV=1 | 36478 | 5.2617 | 0.031 | D | O |
| Q13363 | C-terminal-binding protein 1 OS=Homo sapiens GN=CTBP1 PE=1 SV=2 | 47505 | 6.293 | 0.137 | D | O |
| Q13423 | NAD(P) transhydrogenase, mitochondrial OS=Homo sapiens GN=NNT PE=1 SV=3 | 113822 | 8.0566 | 0.367 | D | O |
| Q13492 | Phosphatidylinositol-binding clathrin assembly protein OS=Homo sapiens GN=PICALM PE=1 S' | 70710 | 7.9556 | 0.025 | D | O |
| Q13509 | Tubulin beta-3 chain OS=Homo sapiens GN=TUBB3 PE=1 SV=2 | 50400 | 4.6392 | 2.347 | D | O |
| Q13554 | Calcium/calmodulin-dependent protein kinase type II subunit beta OS=Homo sapiens GN=CAMI | 72632 | 6.8818 | 0.594 | D | O |
| Q13561 | Dynactin subunit 2 OS=Homo sapiens GN=DCTN2 PE=1 SV=4 | 44203 | 4.9248 | 0.134 | D | O |
| Q13733 | Sodium/potassium-transporting ATPase subunit alpha-4 OS=Homo sapiens GN=ATP1A4 PE=1 | 114093 | 6.2007 | 0.674 | D | O |
| Q13740 | CD166 antigen OS=Homo sapiens GN=ALCAM PE=1 SV=2 | 65061 | 5.8477 | 0.183 | D | O |
| Q13748 | Tubulin alpha-3C/D chain OS=Homo sapiens GN=TUBA3C PE=1 SV=3 | 49927 | 4.7974 | 0.238 | D | O |
| Q13825 | Methylglutaconyl-CoA hydratase, mitochondrial OS=Homo sapiens GN=AUH PE=1 SV=1 | 35586 | 9.8687 | 0.160 | D | O |
| Q13838 | Spliceosome RNA helicase DDX39B OS=Homo sapiens GN=DDX39B PE=1 SV=1 | 48960 | 5.313 | 0.249 | D | O |
| Q13885 | Tubulin beta-2A chain OS=Homo sapiens GN=TUBB2A PE=1 SV=1 | 49874 | 4.5908 | 3.961 | D | O |
| Q14011 | Cold-inducible RNA-binding protein OS=Homo sapiens GN=CIRBP PE=1 SV=1 | 18636 | 9.7397 | 0.286 | D | O |
| Q14019 | Coactosin-like protein OS=Homo sapiens GN=COTL1 PE=1 SV=3 | 15935 | 5.3408 | 0.250 | D | O |
| Q14194 | Dihydropyrimidinase-related protein 1 OS=Homo sapiens GN=CRMP1 PE=1 SV=1 | 62144 | 6.5801 | 0.738 | D | O |
| Q14195 | Dihydropyrimidinase-related protein 3 OS=Homo sapiens GN=DPYSL3 PE=1 SV=1 | 61924 | 6.0249 | 0.240 | D | O |
| Q14240 | Eukaryotic initiation factor 4A-II OS=Homo sapiens GN=EIF4A2 PE=1 SV=2 | 46372 | 5.1636 | 0.361 | D | O |
| Q14247 | Src substrate cortactin OS=Homo sapiens GN=CTTN PE=1 SV=2 | 61548 | 5.0771 | 0.099 | D | O |
| Q14257 | Reticulocalbin-2 OS=Homo sapiens GN=RCN2 PE=1 SV=1 | 36853 | 4.0635 | 0.153 | D | O |
| Q14344 | Guanine nucleotide-binding protein subunit alpha-13 OS=Homo sapiens GN=GNA13 PE=1 SV= | 44021 | 7.9966 | 0.039 | D | O |
| Q14568 | Heat shock protein HSP 90-alpha A2 OS=Homo sapiens GN=HSP90AA2P PE=1 SV=2 | 39340 | 4.377 | 0.034 | D | O |
| Q14690 | Protein RRP5 homolog OS=Homo sapiens GN=PDCD11 PE=1 SV=3 | 208569 | 9.1084 | 0.081 | D | O |
| Q14894 | Ketimine reductase mu-crystallin OS=Homo sapiens GN=CRYM PE=1 SV=1 | 33754 | 4.8765 | 1.434 | D | O |
| Q14974 | Importin subunit beta-1 OS=Homo sapiens GN=KPNB1 PE=1 SV=2 | 97108 | 4.4868 | 0.121 | D | O |
| Q14CZ8 | Hepatocyte cell adhesion molecule OS=Homo sapiens GN=HEPACAM PE=1 SV=1 | 45998 | 9.3999 | 0.405 | D | O |
| Q15019 | Septin-2 OS=Homo sapiens GN=SEPT2 PE=1 SV=1 | 41461 | 6.1392 | 0.298 | D | O |
| Q15056 | Eukaryotic translation initiation factor 4H OS=Homo sapiens GN=EIF4H PE=1 SV=5 | 27368 | 7.2026 | 0.162 | D | O |
| Q15084 | Protein disulfide-isomerase A6 OS=Homo sapiens GN=PDIA6 PE=1 SV=1 | 48091 | 4.7622 | 0.211 | D | O |
| Q15121 | Astrocytic phosphoprotein PEA-15 OS=Homo sapiens GN=PEA15 PE=1 SV=2 | 15030 | 4.7388 | 0.507 | D | O |
| Q15181 | Inorganic pyrophosphatase OS=Homo sapiens GN=PPA1 PE=1 SV=2 | 32639 | 5.4419 | 0.062 | D | O |
| Q15365 | Poly(rC)-binding protein 1 OS=Homo sapiens GN=PCBP1 PE=1 SV=2 | 37473 | 6.7148 | 0.103 | D | O |
| Q15435 | Protein phosphatase 1 regulatory subunit 7 OS=Homo sapiens GN=PPP1R7 PE=1 SV=1 | 41538 | 4.6479 | 0.178 | D | O |
| Q15555 | Microtubule-associated protein RP/EB family member 2 OS=Homo sapiens GN=MAPRE2 PE=1 | 37008 | 5.2324 | 0.250 | D | O |
| Q15691 | Microtubule-associated protein RP/EB family member 1 OS=Homo sapiens GN=MAPRE1 PE=1 | 29980 | 4.8296 | 0.054 | D | O |
| Q15700 | Disks large homolog 2 OS=Homo sapiens GN=DLG2 PE=1 SV=3 | 97491 | 6.0015 | 0.075 | D | O |
| Q16143 | Beta-synuclein OS=Homo sapiens GN=SNCB PE=1 SV=1 | 14279 | 4.21 | 1.033 | D | O |
| Q16352 | Alpha-internexin OS=Homo sapiens GN=INA PE=1 SV=2 | 55357 | 5.1694 | 0.331 | D | O |
| Q16555 | Dihydropyrimidinase-related protein 2 OS=Homo sapiens GN=DPYSL2 PE=1 SV=1 | 62254 | 5.9238 | 2.286 | D | O |
| Q16623 | Syntaxin-1A OS=Homo sapiens GN=STX1A PE=1 SV=1 | 33002 | 4.9556 | 0.169 | D | O |
| Q16643 | Drebrin OS=Homo sapiens GN=DBN1 PE=1 SV=4 | 71385 | 4.2026 | 0.241 | D | O |
| Q16658 | Fascin OS=Homo sapiens GN=FSCN1 PE=1 SV=3 | 54496 | 6.876 | 0.214 | D | O |
| Q16659 | Mitogen-activated protein kinase 6 OS=Homo sapiens GN=MAPK6 PE=1 SV=1 | 82628 | 4.73 | 0.016 | D | O |
| Q16698 | 2,4-dienoyl-CoA reductase, mitochondrial OS=Homo sapiens GN=DECR1 PE=1 SV=1 | 36044 | 9.6606 | 0.135 | D | O |

| | | | | | | |
|---|---|---|---|---|---|---|
| Q16720 | Plasma membrane calcium-transporting ATPase 3 OS=Homo sapiens GN=ATP2B3 PE=1 SV=3 | 134112 | 5.2954 | 0.026 | D | O |
| Q16795 | NADH dehydrogenase [ubiquinone] 1 alpha subcomplex subunit 9, mitochondrial OS=Homo sap | 42482 | 10.1558 | 0.162 | D | O |
| Q16799 | Reticulon-1 OS=Homo sapiens GN=RTN1 PE=1 SV=1 | 83566 | 4.415 | 0.396 | D | O |
| Q16864 | V-type proton ATPase subunit F OS=Homo sapiens GN=ATP6V1F PE=1 SV=2 | 13361 | 5.1504 | 0.110 | D | O |
| Q2TB90 | Putative hexokinase HKDC1 OS=Homo sapiens GN=HKDC1 PE=1 SV=3 | 102478 | 6.7412 | 0.036 | D | O |
| Q32Q12 | Nucleoside diphosphate kinase OS=Homo sapiens GN=NME1-NME2 PE=1 SV=1 | 32620 | 8.647 | 0.891 | D | O |
| Q3L8U1 | Chromodomain-helicase-DNA-binding protein 9 OS=Homo sapiens GN=CHD9 PE=1 SV=2 | 325817 | 6.5493 | 0.025 | D | O |
| Q3ZCW2 | Galectin-related protein OS=Homo sapiens GN=LGALSL PE=1 SV=2 | 18973 | 4.96 | 0.056 | D | O |
| Q53FP2 | Transmembrane protein 35 OS=Homo sapiens GN=TMEM35 PE=2 SV=2 | 18428 | 10.4766 | 0.102 | D | O |
| Q56VL3 | OCIA domain-containing protein 2 OS=Homo sapiens GN=OCIAD2 PE=1 SV=1 | 16942 | 9.3193 | 0.067 | D | O |
| Q58FF6 | Putative heat shock protein HSP 90-beta 4 OS=Homo sapiens GN=HSP90AB4P PE=5 SV=1 | 58227 | 4.4531 | 0.035 | D | O |
| Q58FF8 | Putative heat shock protein HSP 90-beta 2 OS=Homo sapiens GN=HSP90AB2P PE=1 SV=2 | 44321 | 4.5894 | 0.020 | D | O |
| Q58FG1 | Putative heat shock protein HSP 90-alpha A4 OS=Homo sapiens GN=HSP90AA4P PE=5 SV=1 | 47682 | 4.8896 | 0.021 | D | O |
| Q5BJH1 | PSAP protein OS=Homo sapiens GN=PSAP PE=1 SV=1 | 26291 | 5.6338 | 1.178 | D | O |
| Q5JRA6 | Melanoma inhibitory activity protein 3 OS=Homo sapiens GN=MIA3 PE=1 SV=1 | 213568 | 4.5688 | 2.125 | D | O |
| Q5JTJ3 | Cytochrome c oxidase assembly factor 6 homolog OS=Homo sapiens GN=COA6 PE=1 SV=1 | 14106 | 8.2866 | 0.091 | D | O |
| Q5JWF2 | Guanine nucleotide-binding protein G(s) subunit alpha isoforms XLas OS=Homo sapiens GN=G | 110955 | 4.7256 | 0.061 | D | O |
| Q5SWX3 | Calcium/calmodulin-dependent protein kinase (CaM kinase) II gamma, isoform CRA_n OS=Hom | 57760 | 6.9009 | 0.030 | D | O |
| Q5TC82 | Roquin-1 OS=Homo sapiens GN=RC3H1 PE=1 SV=1 | 125657 | 6.8643 | 0.394 | D | O |
| Q5TZA2 | Rootletin OS=Homo sapiens GN=CROCC PE=1 SV=1 | 228385 | 5.2778 | 1.001 | D | O |
| Q5XKE5 | Keratin, type II cytoskeletal 79 OS=Homo sapiens GN=KRT79 PE=1 SV=2 | 57800 | 6.8262 | 0.016 | D | O |
| Q66GS9 | Centrosomal protein of 135 kDa OS=Homo sapiens GN=CEP135 PE=1 SV=2 | 133407 | 5.8081 | 0.182 | D | O |
| Q68D91 | Metallo-beta-lactamase domain-containing protein 2 OS=Homo sapiens GN=MBLAC2 PE=1 SV | 31351 | 6.4468 | 0.075 | D | O |
| Q6DT37 | Serine/threonine-protein kinase MRCK gamma OS=Homo sapiens GN=CDC42BPG PE=1 SV=2 | 172351 | 5.855 | 0.140 | D | O |
| Q6IAA8 | Ragulator complex protein LAMTOR1 OS=Homo sapiens GN=LAMTOR1 PE=1 SV=2 | 17733 | 4.8325 | 0.074 | D | O |
| Q6IQ22 | Ras-related protein Rab-12 OS=Homo sapiens GN=RAB12 PE=1 SV=3 | 27231 | 8.5107 | 0.083 | D | O |
| Q6PCE3 | Glucose 1,6-bisphosphate synthase OS=Homo sapiens GN=PGM2L1 PE=1 SV=3 | 70396 | 6.8042 | 0.285 | D | O |
| Q6PEY2 | Tubulin alpha-3E chain OS=Homo sapiens GN=TUBA3E PE=1 SV=2 | 49826 | 4.8296 | 5.548 | D | O |
| Q6PIK3 | HCG1995540, isoform CRA_b OS=Homo sapiens GN=RAB4B PE=1 SV=1 | 13527 | 9.4951 | 0.015 | D | O |
| Q6S8J3 | POTE ankyrin domain family member E OS=Homo sapiens GN=POTEE PE=1 SV=3 | 121285 | 5.7715 | 0.027 | D | O |
| Q6UXG8 | Butyrophilin-like protein 9 OS=Homo sapiens GN=BTNL9 PE=2 SV=1 | 59678 | 5.9839 | 9.552 | D | O |
| Q709C8 | Vacuolar protein sorting-associated protein 13C OS=Homo sapiens GN=VPS13C PE=1 SV=1 | 422122 | 6.3647 | 0.484 | D | O |
| Q70CQ2 | Ubiquitin carboxyl-terminal hydrolase 34 OS=Homo sapiens GN=USP34 PE=1 SV=2 | 403972 | 5.4258 | 0.096 | D | O |
| Q70EL4 | Ubiquitin carboxyl-terminal hydrolase 43 OS=Homo sapiens GN=USP43 PE=1 SV=2 | 122732 | 9.394 | 0.001 | D | O |
| Q70J99 | Protein unc-13 homolog D OS=Homo sapiens GN=UNC13D PE=1 SV=1 | 123204 | 6.1875 | 3.065 | D | O |
| Q71U36 | Tubulin alpha-1A chain OS=Homo sapiens GN=TUBA1A PE=1 SV=1 | 50103 | 4.7622 | 7.968 | D | O |
| Q7KZF4 | Staphylococcal nuclease domain-containing protein 1 OS=Homo sapiens GN=SND1 PE=1 SV= | 101933 | 6.75 | 0.118 | D | O |
| Q7L099 | Protein RUFY3 OS=Homo sapiens GN=RUFY3 PE=1 SV=1 | 52931 | 5.2002 | 0.144 | D | O |
| Q7L0J3 | Synaptic vesicle glycoprotein 2A OS=Homo sapiens GN=SV2A PE=1 SV=1 | 82642 | 5.2354 | 0.360 | D | O |
| Q7L1I2 | Synaptic vesicle glycoprotein 2B OS=Homo sapiens GN=SV2B PE=1 SV=1 | 77393 | 5.1152 | 0.134 | D | O |
| Q7L266 | Isoaspartyl peptidase/L-asparaginase OS=Homo sapiens GN=ASRGL1 PE=1 SV=2 | 32034 | 5.7993 | 0.079 | D | O |
| Q7L576 | Cytoplasmic FMR1-interacting protein 1 OS=Homo sapiens GN=CYFIP1 PE=1 SV=1 | 145088 | 6.4541 | 0.155 | D | O |
| Q7Z406 | Myosin-14 OS=Homo sapiens GN=MYH14 PE=1 SV=2 | 227730 | 5.3584 | 0.044 | D | O |
| Q7Z494 | Nephrocystin-3 OS=Homo sapiens GN=NPHP3 PE=1 SV=1 | 150768 | 6.3018 | 0.083 | D | O |
| Q7Z7A1 | Centriolin OS=Homo sapiens GN=CNTRL PE=1 SV=2 | 268718 | 5.2837 | 0.270 | D | O |
| Q86VP6 | Cullin-associated NEDD8-dissociated protein 1 OS=Homo sapiens GN=CAND1 PE=1 SV=2 | 136288 | 5.4067 | 0.181 | D | O |
| Q86WA6 | Valacyclovir hydrolase OS=Homo sapiens GN=BPHL PE=1 SV=1 | 32521 | 9.4673 | 0.025 | D | O |
| Q86XW9 | Thioredoxin domain-containing protein 6 OS=Homo sapiens GN=NME9 PE=2 SV=1 | 36832 | 4.623 | 1.379 | D | O |
| Q86Y39 | NADH dehydrogenase [ubiquinone] 1 alpha subcomplex subunit 11 OS=Homo sapiens GN=ND | 14842 | 8.9077 | 0.180 | D | O |
| Q86Y46 | Keratin, type II cytoskeletal 73 OS=Homo sapiens GN=KRT73 PE=1 SV=1 | 58886 | 6.9932 | 0.070 | D | O |
| Q8IV08 | Phospholipase D3 OS=Homo sapiens GN=PLD3 PE=1 SV=1 | 54670 | 6.0073 | 0.205 | D | O |
| Q8IXI2 | Mitochondrial Rho GTPase 1 OS=Homo sapiens GN=RHOT1 PE=1 SV=2 | 70739 | 5.8271 | 0.049 | D | O |
| Q8IYE1 | Coiled-coil domain-containing protein 13 OS=Homo sapiens GN=CCDC13 PE=1 SV=2 | 80834 | 9.334 | 0.078 | D | O |
| Q8N126 | Cell adhesion molecule 3 OS=Homo sapiens GN=CADM3 PE=1 SV=1 | 43272 | 5.6572 | 0.226 | D | O |
| Q8N1G4 | Leucine-rich repeat-containing protein 47 OS=Homo sapiens GN=LRRC47 PE=1 SV=1 | 63433 | 8.272 | 0.041 | D | O |
| Q8N1N4 | Keratin, type II cytoskeletal 78 OS=Homo sapiens GN=KRT78 PE=2 SV=2 | 56830 | 5.6821 | 0.064 | D | O |
| Q8N3J6 | Cell adhesion molecule 2 OS=Homo sapiens GN=CADM2 PE=2 SV=1 | 47524 | 4.9922 | 0.212 | D | O |
| Q8N4C8 | Misshapen-like kinase 1 OS=Homo sapiens GN=MINK1 PE=1 SV=2 | 149728 | 7.7168 | 0.077 | D | O |
| Q8N6N7 | Acyl-CoA-binding domain-containing protein 7 OS=Homo sapiens GN=ACBD7 PE=1 SV=1 | 9784 | 6.7588 | 0.296 | D | O |
| Q8N7X1 | RNA-binding motif protein, X-linked-like-3 OS=Homo sapiens GN=RBMXL3 PE=2 SV=2 | 114868 | 9.1538 | 0.116 | D | O |
| Q8N988 | Zinc finger protein 557 OS=Homo sapiens GN=ZNF557 PE=2 SV=2 | 48595 | 9.082 | 0.025 | D | O |
| Q8N9I0 | Synaptotagmin-2 OS=Homo sapiens GN=SYT2 PE=1 SV=2 | 46842 | 7.9834 | 0.867 | D | O |
| Q8NBM8 | Prenylcysteine oxidase-like OS=Homo sapiens GN=PCYOX1L PE=1 SV=2 | 54611 | 6.8892 | 0.087 | D | O |
| Q8NFZ8 | Cell adhesion molecule 4 OS=Homo sapiens GN=CADM4 PE=1 SV=1 | 42758 | 5.8755 | 0.056 | D | O |
| Q8NHH9 | Atlastin-2 OS=Homo sapiens GN=ATL2 PE=1 SV=2 | 66187 | 5.1489 | 0.085 | D | O |
| Q8TB36 | Ganglioside-induced differentiation-associated protein 1 OS=Homo sapiens GN=GDAP1 PE=1 S | 41319 | 8.4858 | 0.275 | D | O |
| Q8TC12 | Retinol dehydrogenase 11 OS=Homo sapiens GN=RDH11 PE=1 SV=2 | 35363 | 9.0015 | 0.038 | D | O |
| Q8TD26 | Chromodomain-helicase-DNA-binding protein 6 OS=Homo sapiens GN=CHD6 PE=1 SV=4 | 305218 | 5.8403 | 0.037 | D | O |
| Q8WUM4 | Programmed cell death 6-interacting protein OS=Homo sapiens GN=PDCD6IP PE=1 SV=1 | 95963 | 6.0864 | 0.061 | D | O |
| Q8WWZ7 | ATP-binding cassette sub-family A member 5 OS=Homo sapiens GN=ABCA5 PE=2 SV=2 | 186386 | 6.501 | 0.026 | D | O |
| Q8WXF1 | Paraspeckle component 1 OS=Homo sapiens GN=PSPC1 PE=1 SV=1 | 58706 | 6.2344 | 0.088 | D | O |
| Q8WXF7 | Atlastin-1 OS=Homo sapiens GN=ATL1 PE=1 SV=1 | 63503 | 5.7568 | 0.058 | D | O |
| Q8WY54 | Protein phosphatase 1E OS=Homo sapiens GN=PPM1E PE=1 SV=2 | 84948 | 4.7666 | 0.136 | D | O |
| Q92526 | T-complex protein 1 subunit zeta-2 OS=Homo sapiens GN=CCT6B PE=1 SV=5 | 57784 | 6.8906 | 0.196 | D | O |
| Q92561 | Phytanoyl-CoA hydroxylase-interacting protein OS=Homo sapiens GN=PHYHIP PE=1 SV=1 | 37548 | 6.5566 | 0.217 | D | O |
| Q92598 | Heat shock protein 105 kDa OS=Homo sapiens GN=HSPH1 PE=1 SV=1 | 96803 | 5.0991 | 0.027 | D | O |
| Q92599 | Septin-8 OS=Homo sapiens GN=SEPT8 PE=1 SV=4 | 55721 | 5.8418 | 0.231 | D | O |
| Q92752 | Tenascin-R OS=Homo sapiens GN=TNR PE=1 SV=3 | 149467 | 4.522 | 0.662 | D | O |
| Q92777 | Synapsin-2 OS=Homo sapiens GN=SYN2 PE=2 SV=3 | 62807 | 8.5474 | 0.068 | D | O |
| Q92841 | Probable ATP-dependent RNA helicase DDX17 OS=Homo sapiens GN=DDX17 PE=1 SV=2 | 80222 | 8.2397 | 0.092 | D | O |
| Q92930 | Ras-related protein Rab-8B OS=Homo sapiens GN=RAB8B PE=1 SV=2 | 23569 | 9.4321 | 0.250 | D | O |

| | | | | | | |
|---|---|---|---|---|---|---|
| Q92973 | Transportin-1 OS=Homo sapiens GN=TNPO1 PE=1 SV=2 | 102288 | 4.6479 | 0.150 | D | O |
| Q93050 | V-type proton ATPase 116 kDa subunit a isoform 1 OS=Homo sapiens GN=ATP6V0A1 PE=1 S | 96350 | 5.981 | 0.770 | D | O |
| Q969P0 | Immunoglobulin superfamily member 8 OS=Homo sapiens GN=IGSF8 PE=1 SV=1 | 64993 | 7.8999 | 0.156 | D | O |
| Q96A08 | Histone H2B type 1-A OS=Homo sapiens GN=HIST1H2BA PE=1 SV=3 | 14158 | 10.7402 | 1.199 | D | O |
| Q96A23 | Copine-4 OS=Homo sapiens GN=CPNE4 PE=1 SV=1 | 62355 | 5.8799 | 0.043 | D | O |
| Q96C19 | EF-hand domain-containing protein D2 OS=Homo sapiens GN=EFHD2 PE=1 SV=1 | 26680 | 4.9556 | 0.084 | D | O |
| Q96CV9 | Optineurin OS=Homo sapiens GN=OPTN PE=1 SV=2 | 65880 | 4.998 | 0.040 | D | O |
| Q96DA2 | Ras-related protein Rab-39B OS=Homo sapiens GN=RAB39B PE=1 SV=1 | 24606 | 7.8472 | 0.015 | D | O |
| Q96E17 | Ras-related protein Rab-3C OS=Homo sapiens GN=RAB3C PE=2 SV=1 | 25935 | 4.9028 | 0.523 | D | O |
| Q96FC7 | Phytanoyl-CoA hydroxylase-interacting protein-like OS=Homo sapiens GN=PHYHIPL PE=1 SV= | 42458 | 5.9561 | 0.073 | D | O |
| Q96FJ2 | Dynein light chain 2, cytoplasmic OS=Homo sapiens GN=DYNLL2 PE=1 SV=1 | 10343 | 7.2422 | 0.552 | D | O |
| Q96GD0 | Pyridoxal phosphate phosphatase OS=Homo sapiens GN=PDXP PE=1 SV=2 | 31678 | 6.0981 | 0.072 | D | O |
| Q96GW7 | Brevican core protein OS=Homo sapiens GN=BCAN PE=1 SV=2 | 99056 | 4.377 | 0.251 | D | O |
| Q96HU8 | GTP-binding protein Di-Ras2 OS=Homo sapiens GN=DIRAS2 PE=1 SV=1 | 22470 | 8.9678 | 0.041 | D | O |
| Q96IX5 | Up-regulated during skeletal muscle growth protein 5 OS=Homo sapiens GN=USMG5 PE=1 SV | 6453 | 10.0664 | 0.457 | D | O |
| Q96IZ5 | RNA-binding protein 41 OS=Homo sapiens GN=RBM41 PE=1 SV=2 | 47070 | 8.9912 | 0.768 | D | O |
| Q96JE9 | Microtubule-associated protein 6 OS=Homo sapiens GN=MAP6 PE=1 SV=2 | 86451 | 9.5786 | 0.242 | D | O |
| Q96KP4 | Cytosolic non-specific dipeptidase OS=Homo sapiens GN=CNDP2 PE=1 SV=2 | 52844 | 5.5679 | 0.239 | D | O |
| Q96QB1 | Rho GTPase-activating protein 7 OS=Homo sapiens GN=DLC1 PE=1 SV=4 | 170484 | 5.9458 | 0.791 | D | O |
| Q96QK1 | Vacuolar protein sorting-associated protein 35 OS=Homo sapiens GN=VPS35 PE=1 SV=2 | 91649 | 5.168 | 0.092 | D | O |
| Q96RT7 | Gamma-tubulin complex component 6 OS=Homo sapiens GN=TUBGCP6 PE=1 SV=3 | 200370 | 5.8579 | 0.414 | D | O |
| Q99426 | Tubulin-folding cofactor B OS=Homo sapiens GN=TBCB PE=1 SV=2 | 27308 | 4.8647 | 0.066 | D | O |
| Q99497 | Protein deglycase DJ-1 OS=Homo sapiens GN=PARK7 PE=1 SV=2 | 19878 | 6.3721 | 0.746 | D | O |
| Q99536 | Synaptic vesicle membrane protein VAT-1 homolog OS=Homo sapiens GN=VAT1 PE=1 SV=2 | 41893 | 5.8506 | 0.095 | D | O |
| Q99584 | Protein S100-A13 OS=Homo sapiens GN=S100A13 PE=1 SV=1 | 11464 | 5.8257 | 0.584 | D | O |
| Q99714 | 3-hydroxyacyl-CoA dehydrogenase type-2 OS=Homo sapiens GN=HSD17B10 PE=1 SV=3 | 26906 | 7.8384 | 0.084 | D | O |
| Q99747 | Gamma-soluble NSF attachment protein OS=Homo sapiens GN=NAPG PE=1 SV=1 | 34724 | 5.1299 | 0.245 | D | O |
| Q99798 | Aconitate hydratase, mitochondrial OS=Homo sapiens GN=ACO2 PE=1 SV=2 | 85371 | 7.3286 | 1.161 | D | O |
| Q99832 | T-complex protein 1 subunit eta OS=Homo sapiens GN=CCT7 PE=1 SV=2 | 59328 | 7.519 | 0.480 | D | O |
| Q99880 | Histone H2B type 1-L OS=Homo sapiens GN=HIST1H2BL PE=1 SV=3 | 13943 | 10.7402 | 2.150 | D | O |
| Q99961 | Endophilin-A2 OS=Homo sapiens GN=SH3GL1 PE=1 SV=1 | 41464 | 5.1387 | 0.261 | D | O |
| Q99962 | Endophilin-A1 OS=Homo sapiens GN=SH3GL2 PE=1 SV=1 | 39937 | 5.1636 | 0.623 | D | O |
| Q9BPW8 | Protein NipSnap homolog 1 OS=Homo sapiens GN=NIPSNAP1 PE=1 SV=1 | 33288 | 9.5581 | 0.202 | D | O |
| Q9BPX5 | Actin-related protein 2/3 complex subunit 5-like protein OS=Homo sapiens GN=ARPC5L PE=1 S | 16930 | 6.1729 | 0.235 | D | O |
| Q9BRA2 | Thioredoxin domain-containing protein 17 OS=Homo sapiens GN=TXNDC17 PE=1 SV=1 | 13931 | 5.231 | 0.104 | D | O |
| Q9BTC0 | Death-inducer obliterator 1 OS=Homo sapiens GN=DIDO1 PE=1 SV=5 | 243720 | 7.7563 | 0.098 | D | O |
| Q9BUF5 | Tubulin beta-6 chain OS=Homo sapiens GN=TUBB6 PE=1 SV=1 | 49825 | 4.5791 | 1.725 | D | O |
| Q9BVA1 | Tubulin beta-2B chain OS=Homo sapiens GN=TUBB2B PE=1 SV=1 | 49920 | 4.5908 | 1.252 | D | O |
| Q9BW30 | Tubulin polymerization-promoting protein family member 3 OS=Homo sapiens GN=TPPP3 PE= | 18973 | 9.5288 | 0.310 | D | O |
| Q9BX68 | Histidine triad nucleotide-binding protein 2, mitochondrial OS=Homo sapiens GN=HINT2 PE=1 S | 17151 | 9.5874 | 0.086 | D | O |
| Q9BY89 | Uncharacterized protein KIAA1671 OS=Homo sapiens GN=KIAA1671 PE=1 SV=2 | 196589 | 8.5474 | 9.041 | D | O |
| Q9BYX7 | Putative beta-actin-like protein 3 OS=Homo sapiens GN=POTEKP PE=5 SV=1 | 41988 | 5.8843 | 4.995 | D | O |
| Q9GZP4 | PITH domain-containing protein 1 OS=Homo sapiens GN=PITHD1 PE=1 SV=1 | 24162 | 5.376 | 0.069 | D | O |
| Q9H0C2 | ADP/ATP translocase 4 OS=Homo sapiens GN=SLC25A31 PE=2 SV=1 | 34999 | 10.2378 | 0.017 | D | O |
| Q9H0E2 | Toll-interacting protein OS=Homo sapiens GN=TOLLIP PE=1 SV=1 | 30262 | 5.584 | 0.090 | D | O |
| Q9H0U4 | Ras-related protein Rab-1B OS=Homo sapiens GN=RAB1B PE=1 SV=1 | 22157 | 5.395 | 0.749 | D | O |
| Q9H115 | Beta-soluble NSF attachment protein OS=Homo sapiens GN=NAPB PE=1 SV=2 | 33535 | 5.1694 | 0.360 | D | O |
| Q9H1K4 | Mitochondrial glutamate carrier 2 OS=Homo sapiens GN=SLC25A18 PE=1 SV=1 | 33826 | 9.5039 | 0.804 | D | O |
| Q9H254 | Spectrin beta chain, non-erythrocytic 4 OS=Homo sapiens GN=SPTBN4 PE=1 SV=2 | 288806 | 5.6323 | 0.302 | D | O |
| Q9H3F6 | BTB/POZ domain-containing adapter for CUL3-mediated RhoA degradation protein 3 OS=Homo | 35409 | 5.9238 | 0.109 | D | O |
| Q9H3Z4 | DnaJ homolog subfamily C member 5 OS=Homo sapiens GN=DNAJC5 PE=1 SV=1 | 22134 | 4.7446 | 0.340 | D | O |
| Q9H4B7 | Tubulin beta-1 chain OS=Homo sapiens GN=TUBB1 PE=1 SV=1 | 50294 | 4.8823 | 0.047 | D | O |
| Q9H4M9 | EH domain-containing protein 1 OS=Homo sapiens GN=EHD1 PE=1 SV=2 | 60588 | 6.356 | 0.034 | D | O |
| Q9H6K4 | Optic atrophy 3 protein OS=Homo sapiens GN=OPA3 PE=1 SV=1 | 19983 | 9.2402 | 0.051 | D | O |
| Q9H6R4 | Nucleolar protein 6 OS=Homo sapiens GN=NOL6 PE=1 SV=2 | 127513 | 7.3477 | 2.453 | D | O |
| Q9H9B4 | Sideroflexin-1 OS=Homo sapiens GN=SFXN1 PE=1 SV=4 | 35596 | 9.3457 | 0.532 | D | O |
| Q9HB63 | Netrin-4 OS=Homo sapiens GN=NTN4 PE=1 SV=2 | 70024 | 7.9014 | 0.055 | D | O |
| Q9HB71 | Calcyclin-binding protein OS=Homo sapiens GN=CACYBP PE=1 SV=2 | 26193 | 8.5825 | 0.028 | D | O |
| Q9HCC0 | Methylcrotonoyl-CoA carboxylase beta chain, mitochondrial OS=Homo sapiens GN=MCCC2 PE | 61294 | 7.5293 | 0.024 | D | O |
| Q9HCK8 | Chromodomain-helicase-DNA-binding protein 8 OS=Homo sapiens GN=CHD8 PE=1 SV=5 | 290335 | 6.0132 | 0.013 | D | O |
| Q9HDC9 | Adipocyte plasma membrane-associated protein OS=Homo sapiens GN=APMAP PE=1 SV=2 | 46450 | 5.7466 | 0.360 | D | O |
| Q9NP72 | Ras-related protein Rab-18 OS=Homo sapiens GN=RAB18 PE=1 SV=1 | 22962 | 4.9263 | 0.049 | D | O |
| Q9NPJ3 | Acyl-coenzyme A thioesterase 13 OS=Homo sapiens GN=ACOT13 PE=1 SV=1 | 14950 | 9.6343 | 0.250 | D | O |
| Q9NQ66 | 1-phosphatidylinositol 4,5-bisphosphate phosphodiesterase beta-1 OS=Homo sapiens GN=PLC | 138479 | 5.8257 | 0.064 | D | O |
| Q9NQC3 | Reticulon-4 OS=Homo sapiens GN=RTN4 PE=1 SV=2 | 129851 | 4.2246 | 0.307 | D | O |
| Q9NQR4 | Omega-amidase NIT2 OS=Homo sapiens GN=NIT2 PE=1 SV=1 | 30588 | 6.9858 | 0.077 | D | O |
| Q9NQX4 | Unconventional myosin-Vc OS=Homo sapiens GN=MYO5C PE=1 SV=2 | 202681 | 7.5103 | 0.040 | D | O |
| Q9NR31 | GTP-binding protein SAR1a OS=Homo sapiens GN=SAR1A PE=1 SV=1 | 22352 | 6.2344 | 0.333 | D | O |
| Q9NS69 | Mitochondrial import receptor subunit TOM22 homolog OS=Homo sapiens GN=TOMM22 PE=1 | 15511 | 4.0649 | 0.019 | D | O |
| Q9NS86 | LanC-like protein 2 OS=Homo sapiens GN=LANCL2 PE=1 SV=1 | 50821 | 7.1499 | 0.093 | D | O |
| Q9NSD9 | Phenylalanine--tRNA ligase beta subunit OS=Homo sapiens GN=FARSB PE=1 SV=3 | 66073 | 6.3955 | 0.313 | D | O |
| Q9NUJ1 | Mycophenolic acid acyl-glucuronide esterase, mitochondrial OS=Homo sapiens GN=ABHD10 P | 33910 | 8.7056 | 0.236 | D | O |
| Q9NUQ9 | Protein FAM49B OS=Homo sapiens GN=FAM49B PE=1 SV=1 | 36724 | 5.6792 | 0.037 | D | O |
| Q9NVJ2 | ADP-ribosylation factor-like protein 8B OS=Homo sapiens GN=ARL8B PE=1 SV=1 | 21525 | 8.6045 | 0.251 | D | O |
| Q9NY65 | Tubulin alpha-8 chain OS=Homo sapiens GN=TUBA8 PE=1 SV=1 | 50061 | 4.7578 | 0.545 | D | O |
| Q9NZ45 | CDGSH iron-sulfur domain-containing protein 1 OS=Homo sapiens GN=CISD1 PE=1 SV=1 | 12191 | 9.4468 | 0.201 | D | O |
| Q9NZJ7 | Mitochondrial carrier homolog 1 OS=Homo sapiens GN=MTCH1 PE=1 SV=1 | 41517 | 9.5801 | 0.058 | D | O |
| Q9NZL9 | Methionine adenosyltransferase 2 subunit beta OS=Homo sapiens GN=MAT2B PE=1 SV=1 | 37528 | 6.9858 | 0.047 | D | O |
| Q9NZQ3 | NCK-interacting protein with SH3 domain OS=Homo sapiens GN=NCKIPSD PE=1 SV=1 | 78910 | 5.9209 | 0.034 | D | O |
| Q9NZR1 | Tropomodulin-2 OS=Homo sapiens GN=TMOD2 PE=1 SV=1 | 39570 | 5.0186 | 0.382 | D | O |
| Q9P035 | Very-long-chain (3R)-3-hydroxyacyl-CoA dehydratase 3 OS=Homo sapiens GN=HACD3 PE=1 S | 43131 | 9.1714 | 0.211 | D | O |

| | | | | | | |
|---|---|---|---|---|---|---|
| Q9P0J0 | NADH dehydrogenase [ubiquinone] 1 alpha subcomplex subunit 13 OS=Homo sapiens GN=ND | 16687 | 9.0513 | 0.435 | D | O |
| Q9P121 | Neurotrimin OS=Homo sapiens GN=NTM PE=1 SV=1 | 37947 | 7.7314 | 0.190 | D | O |
| Q9P2D1 | Chromodomain-helicase-DNA-binding protein 7 OS=Homo sapiens GN=CHD7 PE=1 SV=3 | 335715 | 5.9033 | 0.178 | D | O |
| Q9P2R7 | Succinyl-CoA ligase [ADP-forming] subunit beta, mitochondrial OS=Homo sapiens GN=SUCLA2 | 50285 | 7.2041 | 0.490 | D | O |
| Q9P2U7 | Vesicular glutamate transporter 1 OS=Homo sapiens GN=SLC17A7 PE=2 SV=1 | 61573 | 7.2012 | 0.200 | D | O |
| Q9UBB6 | Neurochondrin OS=Homo sapiens GN=NCDN PE=1 SV=1 | 78813 | 5.1812 | 0.418 | D | O |
| Q9UBI6 | Guanine nucleotide-binding protein G(I)/G(S)/G(O) subunit gamma-12 OS=Homo sapiens GN=( | 8001 | 9.4761 | 0.048 | D | O |
| Q9UDW1 | Cytochrome b-c1 complex subunit 9 OS=Homo sapiens GN=UQCR10 PE=1 SV=3 | 7303 | 9.8643 | 0.258 | D | O |
| Q9UEY8 | Gamma-adducin OS=Homo sapiens GN=ADD3 PE=1 SV=1 | 79105 | 5.8755 | 0.230 | D | O |
| Q9UFN0 | Protein NipSnap homolog 3A OS=Homo sapiens GN=NIPSNAP3A PE=1 SV=2 | 28448 | 9.4556 | 0.062 | D | O |
| Q9UH03 | Neuronal-specific septin-3 OS=Homo sapiens GN=SEPT3 PE=1 SV=3 | 40678 | 6.8218 | 0.475 | D | O |
| Q9UHD8 | Septin-9 OS=Homo sapiens GN=SEPT9 PE=1 SV=2 | 65360 | 9.312 | 0.192 | D | O |
| Q9UHG2 | ProSAAS OS=Homo sapiens GN=PCSK1N PE=1 SV=1 | 27355 | 6.2227 | 0.202 | D | O |
| Q9UHV9 | Prefoldin subunit 2 OS=Homo sapiens GN=PFDN2 PE=1 SV=1 | 16637 | 6.208 | 0.074 | D | O |
| Q9UI09 | NADH dehydrogenase [ubiquinone] 1 alpha subcomplex subunit 12 OS=Homo sapiens GN=ND | 17103 | 9.9419 | 0.155 | D | O |
| Q9UIJ7 | GTP:AMP phosphotransferase AK3, mitochondrial OS=Homo sapiens GN=AK3 PE=1 SV=4 | 25549 | 9.5303 | 0.176 | D | O |
| Q9UJC5 | SH3 domain-binding glutamic acid-rich-like protein 2 OS=Homo sapiens GN=SH3BGRL2 PE=1 | 12318 | 6.8013 | 0.205 | D | O |
| Q9UJS0 | Calcium-binding mitochondrial carrier protein Aralar2 OS=Homo sapiens GN=SLC25A13 PE=1 | 74128 | 8.77 | 0.184 | D | O |
| Q9UJW0 | Dynactin subunit 4 OS=Homo sapiens GN=DCTN4 PE=1 SV=1 | 52303 | 7.0679 | 0.104 | D | O |
| Q9UJZ1 | Stomatin-like protein 2, mitochondrial OS=Homo sapiens GN=STOML2 PE=1 SV=1 | 38510 | 7.2642 | 0.312 | D | O |
| Q9UK22 | F-box only protein 2 OS=Homo sapiens GN=FBXO2 PE=1 SV=2 | 33306 | 4.0957 | 0.176 | D | O |
| Q9UKE5 | TRAF2 and NCK-interacting protein kinase OS=Homo sapiens GN=TNIK PE=1 SV=1 | 154847 | 6.7119 | 0.024 | D | O |
| Q9ULD0 | 2-oxoglutarate dehydrogenase-like, mitochondrial OS=Homo sapiens GN=OGDHL PE=1 SV=3 | 114408 | 6.1758 | 0.135 | D | O |
| Q9ULV4 | Coronin-1C OS=Homo sapiens GN=CORO1C PE=1 SV=1 | 53215 | 6.6724 | 0.232 | D | O |
| Q9UMF0 | Intercellular adhesion molecule 5 OS=Homo sapiens GN=ICAM5 PE=1 SV=3 | 97055 | 5.6016 | 0.153 | D | O |
| Q9UMX0 | Ubiquilin-1 OS=Homo sapiens GN=UBQLN1 PE=1 SV=2 | 62479 | 4.8413 | 0.089 | D | O |
| Q9UMX5 | Neudesin OS=Homo sapiens GN=NENF PE=1 SV=1 | 18844 | 5.3613 | 0.063 | D | O |
| Q9UNZ2 | NSFL1 cofactor p47 OS=Homo sapiens GN=NSFL1C PE=1 SV=2 | 40548 | 4.8076 | 0.066 | D | O |
| Q9UPV7 | PHD finger protein 24 OS=Homo sapiens GN=PHF24 PE=1 SV=2 | 45163 | 5.3496 | 0.044 | D | O |
| Q9UPY8 | Microtubule-associated protein RP/EB family member 3 OS=Homo sapiens GN=MAPRE3 PE=1 | 31961 | 5.1958 | 0.138 | D | O |
| Q9UQ16 | Dynamin-3 OS=Homo sapiens GN=DNM3 PE=1 SV=4 | 97685 | 8.458 | 0.245 | D | O |
| Q9UQM7 | Calcium/calmodulin-dependent protein kinase type II subunit alpha OS=Homo sapiens GN=CAM | 54053 | 6.6284 | 2.150 | D | O |
| Q9Y277 | Voltage-dependent anion-selective channel protein 3 OS=Homo sapiens GN=VDAC3 PE=1 SV= | 30639 | 8.8301 | 1.313 | D | O |
| Q9Y281 | Cofilin-2 OS=Homo sapiens GN=CFL2 PE=1 SV=1 | 18724 | 8.1592 | 1.545 | D | O |
| Q9Y285 | Phenylalanine--tRNA ligase alpha subunit OS=Homo sapiens GN=FARSA PE=1 SV=3 | 57527 | 7.7168 | 0.145 | D | O |
| Q9Y2A7 | Nck-associated protein 1 OS=Homo sapiens GN=NCKAP1 PE=1 SV=1 | 128706 | 6.1685 | 0.096 | D | O |
| Q9Y2J0 | Rabphilin-3A OS=Homo sapiens GN=RPH3A PE=1 SV=1 | 76824 | 8.3174 | 0.074 | D | O |
| Q9Y2J8 | Protein-arginine deiminase type-2 OS=Homo sapiens GN=PADI2 PE=1 SV=2 | 75515 | 5.2588 | 0.330 | D | O |
| Q9Y2Q0 | Phospholipid-transporting ATPase IA OS=Homo sapiens GN=ATP8A1 PE=1 SV=1 | 131285 | 6.394 | 0.097 | D | O |
| Q9Y2T3 | Guanine deaminase OS=Homo sapiens GN=GDA PE=1 SV=1 | 50970 | 5.3262 | 0.296 | D | O |
| Q9Y3D6 | Mitochondrial fission 1 protein OS=Homo sapiens GN=FIS1 PE=1 SV=2 | 16927 | 9.2124 | 0.075 | D | O |
| Q9Y3F4 | Serine-threonine kinase receptor-associated protein OS=Homo sapiens GN=STRAP PE=1 SV= | 38413 | 4.8032 | 0.062 | D | O |
| Q9Y4J8 | Dystrobrevin alpha OS=Homo sapiens GN=DTNA PE=1 SV=2 | 83847 | 6.4263 | 0.024 | D | O |
| Q9Y4L1 | Hypoxia up-regulated protein 1 OS=Homo sapiens GN=HYOU1 PE=1 SV=1 | 111266 | 4.9717 | 0.090 | D | O |
| Q9Y5K8 | V-type proton ATPase subunit D OS=Homo sapiens GN=ATP6V1D PE=1 SV=1 | 28245 | 9.7983 | 0.314 | D | O |
| Q9Y5L4 | Mitochondrial import inner membrane translocase subunit Tim13 OS=Homo sapiens GN=TIMM1 | 10492 | 8.2822 | 0.207 | D | O |
| Q9Y617 | Phosphoserine aminotransferase OS=Homo sapiens GN=PSAT1 PE=1 SV=2 | 40396 | 7.5879 | 0.236 | D | O |
| Q9Y639 | Neuroplastin OS=Homo sapiens GN=NPTN PE=1 SV=2 | 44359 | 7.9834 | 0.536 | D | O |
| Q9Y678 | Coatomer subunit gamma-1 OS=Homo sapiens GN=COPG1 PE=1 SV=1 | 97655 | 5.1665 | 0.030 | D | O |
| Q9Y6K8 | Adenylate kinase isoenzyme 5 OS=Homo sapiens GN=AK5 PE=1 SV=2 | 63293 | 4.7695 | 0.293 | D | O |
| Q9Y6R1 | Electrogenic sodium bicarbonate cotransporter 1 OS=Homo sapiens GN=SLC4A4 PE=1 SV=1 | 121382 | 6.3428 | 0.069 | D | O |
| S4R3Z2 | Aldo-keto reductase family 1 member C3 OS=Homo sapiens GN=AKR1C3 PE=1 SV=1 | 34317 | 8.2339 | 0.012 | D | O |
| V9GYG0 | ADP/ATP translocase 1 OS=Homo sapiens GN=SLC25A4 PE=1 SV=1 | 22871 | 9.8496 | 0.308 | D | O |
| X6RFL8 | Ras-related protein Rab-14 (Fragment) OS=Homo sapiens GN=RAB14 PE=1 SV=1 | 20396 | 5.9121 | 0.349 | D | O |
